# Distinct genomic profiles of gestational choriocarcinoma, a unique cancer of pregnant tissues

**DOI:** 10.1038/s12276-020-00544-0

**Published:** 2020-12-15

**Authors:** Seung-Hyun Jung, Youn Jin Choi, Min Sung Kim, Hyeon-Chun Park, Mi-Ryung Han, Soo Young Hur, Ah Won Lee, Ok Ran Shin, Jeana Kim, Sung Hak Lee, Dongwan Hong, Sang Yong Song, Yeun-Jun Chung, Sug Hyung Lee

**Affiliations:** 1 Cancer Evolution Research Center, The Catholic University of Korea, Seoul, Korea; 2 Department of Biochemistry, The Catholic University of Korea, Seoul, Korea; 3 Department of Obstetrics and Gynecology, The Catholic University of Korea, Seoul, Korea; 4 Cancer Research Institute, The Catholic University of Korea, Seoul, Korea; 5 Department of Pathology, The Catholic University of Korea, Seoul, Korea; 6 Integrated Research Center for Genome Polymorphism, The Catholic University of Korea, Seoul, Korea; 7 Department of Microbiology, The Catholic University of Korea, Seoul, Korea; 8 Division of Life Sciences, College of Life Sciences and Bioengineering, Incheon National University, Incheon, Korea; 9 Department of Hospital Pathology, College of Medicine, The Catholic University of Korea, Seoul, Korea; 10 Department of Pathology and Translational Genomics, Samsung Medical Center, Sungkyunkwan University School of Medicine, Seoul, Korea

**Keywords:** Cancer genomics, Endometrial cancer

## Abstract

Little is known about genomic alterations of gestational choriocarcinoma (GC), unique cancer that originates in pregnant tissues, and the progression mechanisms from the nonmalignant complete hydatidiform mole (CHM) to GC. Whole-exome sequencing (20 GCs) and/or single-nucleotide polymorphism microarray (29 GCs) were performed. We analyzed copy-neutral loss-of-heterozygosity (CN-LOH) in 29 GCs that exhibited androgenetic CN-LOHs (20 monospermic, 8 dispermic) and no CN-LOH (one with *NLRP7* mutation). Most GCs (25/29) harboring recurrent copy number alterations (CNAs) and gains on 1q21.1-q44 were significantly associated with poor prognosis. We detected five driver mutations in the GCs, most of which were chromatin remodeling gene (*ARID1A*, *SMARCD1*, and *EP300*) mutations but not in common cancer genes such as *TP53* and *KRAS*. One patient’s serial CHM/invasive mole/GC showed consistent CN-LOHs, but only the GC harbored CNAs, indicating that CN-LOH is an early pivotal event in HM-IM-GC development, and CNAs may be a late event that promotes CHM progression to GC. Our data indicate that GCs have unique profiles of CN-LOHs, mutations and CNAs that together differentiate GCs from non-GCs. Practically, CN-LOH and CNA profiles are useful for the molecular diagnosis of GC and the selection of GC patients with poor prognosis for more intensive treatments, respectively.

## Cancer: Mapping markers of maternal malignancy

Genomic analysis reveals chromosomal alterations that drive disease progression in a poorly understood class of tumors that form in placental tissue. Gestational choriocarcinoma (GC) arises during pregnancy and can quickly develop into lethal metastatic disease if not treated promptly. To identify the origins of such malignancies, researchers led by Sug Hyung Lee and Yeun-Jun Chung at The Catholic University of Korea, Seoul, profiled genetic aberrations in 29 GC specimens. The researchers did not observe any consistent link between these malignancies and a particular set of ‘driver mutations’ underlying tumor progression as has been seen in other solid tumors. However, these GC samples exhibited striking levels of rearrangement between chromosomes. The researchers propose that the gain or loss of genes resulting from these chromosomal abnormalities may be an important contributor to rapidly progressing forms of this disease.

## Introduction

Gestational trophoblastic diseases (GTDs), including hydatidiform mole (HM), invasive mole (IM), and gestational choriocarcinoma (GC), encompass a spectrum of tumorous conditions featuring proliferation of a woman’s pregnant tissues (placental trophoblasts) but not of a woman’s own tissues^1,2^. HMs are further subdivided into complete and partial HMs^3^. Chromosomal polymorphism analyses have identified that most complete HMs (CHMs) arise when an ovum without maternal chromosomes is fertilized by one sperm that duplicates its DNA (monospermic diploid or uniparental diploid), and only some CHMs (~10%) arise from fertilization by two sperm (dispermic diploid)^1^. Both CHM types could produce copy-neutral loss-of-heterozygosity (CN-LOH) due to errors in meiosis I or meiosis II, which would result in two copies of a chromosome from one parent and no copies from the other parent with no change in the copy number^4–6^. In contrast, partial HM arises from two sperm with an ovum (69XXX, 69XXY, or 69XYY)^4–6^. Many CHMs progress to IM, which invades the uterine wall or blood vessels and rarely metastasizes to distant sites^7^. Eventually, some CHMs (~3%) give rise to GCs, which occupy the malignant end of the GTD spectrum^7^. GCs are known to be preceded by CHMs (50%), previous abortions (25%), or normal/ectopic pregnancies (25%)^1^. They are highly sensitive to chemotherapy, but untreated cases can rapidly metastasize to distant organs and may be fatal^1,8^. Some GCs frequently present with metastasis in the brain or lung even without a diagnosis of uterine CHM or GC^1,9^. However, little is known about predictive biomarkers for poor prognosis.

Preneoplastic conditions such as Barrett’s esophagus frequently harbor genetic alterations even before the progression to a frank cancer^10^. CHM is considered a preneoplastic condition of GC, and IM has neoplastic characteristics such as tissue invasion^1–4^. However, it is not known what triggers CHM/IM to progress to GC^11^. Genomic alterations of GCs, especially with next-generation sequencing (NGS), are not well studied due to the rare availability of tumor tissues (frequent tissue necrosis, infrequent surgery). One study reported whole-exome sequencing (WES) data on a single case of GC, but it did not disclose any well-known driver mutation, probably due to the small case number^12^. To date, cytogenetic analyses of GCs have revealed aneuploidy with a diverse range of chromosomal alterations, including frequent 7p12-q11.2 and 8p12-p21 losses and 7q21-q31 gains^13–15^. An array comparative genomic hybridization study identified frequent gains of 1p36 and 17p25 and losses of 9q33, 17q21 and 18q22^16^. However, remarkable driver gene mutations have not yet been identified in GCs by gene-to-gene analyses^17,18^. The lack of genome-wide alteration data on HM/IM/GC, even in this NGS era, led us to analyze GC genomes by WES, copy number alteration (CNA), and CN-LOH profiles in this study.

## Materials and methods

### Hydatidiform mole, invasive mole, and choriocarcinoma tissues

Formalin-fixed and paraffin-embedded (FFPE) tissues of 31 GTDs in 29 patients (CHM/IM/GC tissues in 1 patient (GC15) and GCs in 28 patients) were used for this study. All of these patients were Korean women, and approval for this study was obtained from the Institutional Review Board at The Catholic University of Korea, College of Medicine (KC16TISE0342). The diagnosis of GC was made based on the clinical or histopathologic criteria of the International Federation of Gynecology and Obstetrics (FIGO)^7^. Histologically, GC is a malignancy characterized by trophoblast proliferation, absence of chorionic villi, and tissue necrosis with bleeding, which were confirmed by two pathologists under microscopic examination. The 29 GCs consisted of 21 primary GCs in the uterus or fallopian tube and eight metastatic GCs (six in the lungs, one in the rectum, and one in the brain). Pregnancy-related history was available in 25 patients (14 with CHMs and 11 with nonmolar pregnancies [six with abortion, one with ectopic pregnancy, and four with term pregnancy]) but was not available in four patients. The clinicopathologic features of the cases are summarized in Table 1. CHM, IM, and GC cells as well as matched normal cells in each case were procured from hematoxylin and eosin-stained slides using microdissection by a board-certified pathologist, as described previously^19^. Tumor cell purities were ~50–80%, which were confirmed by two pathologists under microscopic examination. For genomic DNA extraction, the DNeasy Blood & Tissue Kit (Qiagen) was used. None of the presumed partners’ DNA was available for analyses.

**Table 1:** Clinicopathologic characteristics of 29 choriocarcinoma patients.

Case	Age	Diagnosis	Tissues analyzed^a^	Antecedent pregnancy	LOH pattern^b^	STR^c^	Survival status^d^
GC01	23	Choriocarcinoma	Uterus	CHM	Monospermic	Single nonmaternal peak	Poor survival
GC02	33	Choriocarcinoma	Uterus	Term pregnancy	Biparental	N/A	Good survival
GC03	37	Choriocarcinoma	Uterus	CHM	Monospermic	N/A	Poor survival
GC04	31	Choriocarcinoma	Uterus	CHM	Monospermic	Single nonmaternal peak	Good survival
GC05	30	Choriocarcinoma	Uterus	Term pregnancy	Monospermic	Single nonmaternal peak	Good survival
GC06	48	Choriocarcinoma	Uterus	CHM	Monospermic	N/A	Good survival
GC07	39	Choriocarcinoma	Uterus	CHM	Monospermic	Single nonmaternal peak	Good survival
GC08	26	Choriocarcinoma	Uterus	Ectopic pregnancy	Dispermic	Double nonmaternal peak	Poor survival
GC09	46	Choriocarcinoma	Uterus	Abortion	Monospermic	Single nonmaternal peak	Good survival
GC10	42	Choriocarcinoma	Uterus	Abortion	Monospermic	Single nonmaternal peak	Good survival
GC11	52	Choriocarcinoma	Uterus	Abortion	Monospermic	Single nonmaternal peak	Good survival
GC12	51	Choriocarcinoma	Uterus	CHM	Dispermic	Double nonmaternal peak	Good survival
GC13	39	Choriocarcinoma	Lung	Abortion	Dispermic	Double nonmaternal peak	Poor survival
GC14	43	Choriocarcinoma	Lung	Abortion	Monospermic	Single nonmaternal peak	Good survival
GC15	54	Choriocarcinoma	Brain	CHM	Monospermic	Single nonmaternal peak	Poor survival
GC16	68	Choriocarcinoma	Uterus	CHM	Dispermic	Double nonmaternal peak	Good survival
GC17	48	Choriocarcinoma	Fallopian tube	Unknown	Monospermic	Single nonmaternal peak	Good survival
GC18	34	Choriocarcinoma	Lung	CHM	Monospermic	Single nonmaternal peak	Poor survival
GC19	35	Choriocarcinoma	Uterus	CHM	Monospermic	Single nonmaternal peak	Good survival
GC20	43	Choriocarcinoma	Uterus	Unknown	Monospermic	Single nonmaternal peak	Good survival
GC21	58	Choriocarcinoma	Rectum	Unknown	Monospermic	Single nonmaternal peak	Good survival
GC22	33	Choriocarcinoma	Uterus	CHM	Dispermic	N/A	Poor survival
GC23	32	Choriocarcinoma	Uterus	CHM	Monospermic	Single nonmaternal peak	Poor survival
GC24	28	Choriocarcinoma	Lung	Unknown	Monospermic	Single nonmaternal peak	Poor survival
GC25	31	Choriocarcinoma	Lung	Term pregnancy	Dispermic	Double nonmaternal peak	Good survival
GC26	34	Choriocarcinoma	Uterus	Abortion	Dispermic	N/A	Good survival
GC27	57	Choriocarcinoma	Lung	CHM	Monospermic	Single nonmaternal peak	Good survival
GC28	39	Choriocarcinoma	Uterus	Term pregnancy	Dispermic	Single nonmaternal peak	Poor survival
GC29	45	Choriocarcinoma	Uterus	CHM	Monospermic	Single nonmaternal peak	Poor survival

*CHM* complete hydatidiform mole, *LOH* loss of heterozygosity, *N/A* not available.

^a^Primary cancer: the uterus and fallopian tube, metastatic cancer: lung, rectum, and brain.

^b^LOH pattern was determined using SNP microarray.

^c^STR markers were analyzed using the AmpFLSTR PCR Amplification Kit.

^d^The patients who survived <5 years after GC diagnosis were considered “poor survival”, and those who survived >5 years after GC diagnosis were considered “good survival”.

### Short tandem repeat (STR) marker analysis

To identify the genetic sources of GTDs, STR analysis was performed using genomic DNA from GTD tissues and matched normal cells using the AmpFLSTR Identifiler Plus PCR Amplification Kit (Thermo Fisher Scientific) that covered 16 STR loci (polymorphic DNA markers): *D8S1179, D21S11, D7S820, CSF1PO, D3S1358, TH01, D13S317, D16S539, D2S1338, D19S433, vWA, TPOX, D18S51*, Amelogenin*, D5S818*, and *FGA*. The fluorescent PCR products were analyzed using the ABI 3130XL Genetic Analyzer (Applied Biosystems). Each fluorescent peak was quantified by its size (base pairs), peak height, and peak area, as previously reported^15^, and analyzed by GeneMapper 4.1 software (Applied Biosystems).

### Copy number alteration and loss-of-heterozygosity analyses

Both CNA and LOH of the 29 GTDs were analyzed using a SNP microarray with an Affymetrix OncoScan FFPE assay (Affymetrix) according to the manufacturer’s instructions. OncoScan OSCHP files processed by the TuScan algorithm and the SNP-FASST2 segmentation algorithm in NEXUS software v9.0 (BioDiscovery) were used to define CNAs and LOHs for each sample^20^. Segments were classified as copy gains and losses when the log_2_ ratio was >0.2 and <−0.2, respectively. A homozygous value larger than 0.7 was defined as LOH. B-allele frequencies could be attenuated depending on the levels of normal cell contamination from patients (Supplementary Table 1), which was considered in the interpretation.

### WES analysis

WES libraries were constructed using the Agilent SureSelect Human All Exome 50 Mb kit (Agilent Technologies) according to the manufacturer’s instructions. Input quantities of genomic DNA were equally adjusted for WES analyses. Per WES reaction, we used 800 ng of double-stranded genomic DNA each for normal, CHM, IM, and GC samples. WES libraries were sequenced using the Illumina HiSeq2000 platform to generate 101-bp paired-end reads. Burrows-Wheeler Aligner was used to align the sequencing reads to the human reference genome (hg19). Processing and management of sequencing data were performed as described elsewhere^21^. In brief, somatic genomic variants were identified using MuTect^22^ and SomaticIndelDetector^23^ for point mutations and indels, respectively. The ANNOVAR package was used to select somatic variants located in coding sequences and predict their functional consequences, such as silent or nonsilent variants^24^. To identify somatic mutations in GTD, WES was performed in 22 cases (20 GCs, 1 CHM, and 1 IM). WES was not available for the other 9 cases due to low quality. To obtain reliable and robust mutation calling, the following somatic variants were eliminated: (i) read depth <20 in either tumor or matched normal tissue; (ii) any polymorphisms referenced in either the 1000 Genomes Project or Exome Aggregation Consortium with a minor allele frequency of 1% or more in East Asians; and (iii) polymorphisms that showed >1% minor allele frequency in our in-house normal database (397 whole-genome sequencing data from Korean populations). Because the majority of variants detected in GCs were likely to be paternal polymorphisms^1,2^, they were stringently filtered by dbSNP (version 147). Subsequently, variants reported in the clinically relevant variant (ClinVar) database were rescued to prevent the exclusion of true pathogenic variants.

## Results

### Identification of GC origins

**Fig. 1:**
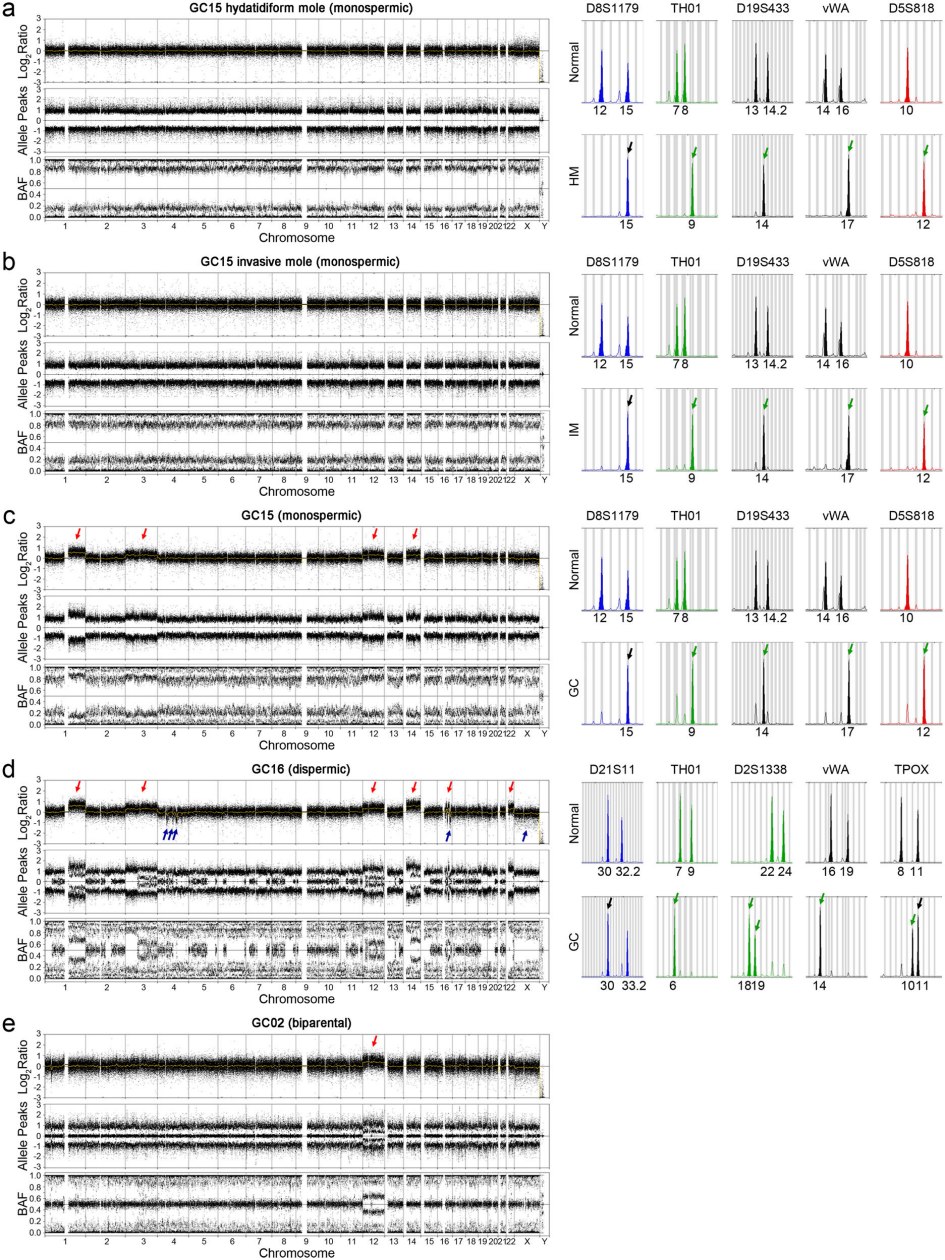
Representative patterns of gestational choriocarcinoma genomes. Monospermic (**a**–**c**), dispermic (**d**), and biparental (**e**) patterns are featured by SNP microarray (left column) and STR marker analysis (right). One patient (GC15) had serial CHM, IM, and GC, and each of these tissues was analyzed (**a**–**c**). In the SNP microarray, logR ratio plots (upper), allele peak plots (middle), and B-allele frequency (BAF) plots (bottom) are shown. Red and blue arrows in the logR ratio plot represent the copy number gain and loss, respectively. Green and black arrows in the STR marker analysis represent the informative alleles that are not identified in matched normal and noninformative alleles, respectively. BAF plots of GC15 (CHM, IM and GC) and GC16 represent two tracks (BB allele (100%) and AA allele (0%)) without the AB allele (50%), suggesting CN-LOH.

Since GCs are known to arise from androgenetic CHMs harboring CN-LOH^16^, we first attempted to address the origins of the GCs. For this, the CN-LOH patterns of 29 GCs were analyzed by single-nucleotide polymorphism (SNP) microarray, which identified three different B-allele patterns (monospermic, dispermic, and biparental). The B-allele frequency (BAF) is calculated by dividing the number of minor (B) alleles by the sum of major (A) and minor alleles. BAF can have values of 100% (BB allele), 50% (AB allele), and 0% (AA allele) in a diploid individual. In CN-LOH, a two-track BAF plot (100 and 0%) representing one haplotype was observed without copy number change. From this point of view, twenty GCs (69%) showed a monospermic pattern (Table 1 and Supplementary Fig. 1) that displayed simple LOHs with only two haplotypes (0 and 100%) across the entire genome (Fig. 1a–c). Another 8 GCs (28%) were identified as the dispermic pattern that showed LOHs across the entire genome with intermittent additional haplotypes (50%), which might be an oscillation of two-haplotype status reflecting the haplotype composition of each sperm (Fig. 1d and Supplementary Fig. 1). The diploidic B-allele pattern of the other GC (GC02) (Fig. 1e) and abundant SNP mismatches between this GC and the patient’s normal cells (*n* = 2353) suggested its nonandrogenetic gestational origin. This patient had a heterozygous *NLRP7* germline mutation (p.Y209C). Very rarely, nonandrogenetic biparental CHMs develop in the family with *NLRP7* germline mutations^25^. Unfortunately, however, her history of molar pregnancy was not available in the hospital record. STR analyses using GCs and matched normal tissues corresponded to those of the B-allele patterns (the monospermic patterns in Fig. 1a–c and Supplementary Table 2; the dispermic patterns in Fig. 1d and Supplementary Table 2).

### Copy number alterations

**Fig. 2:**
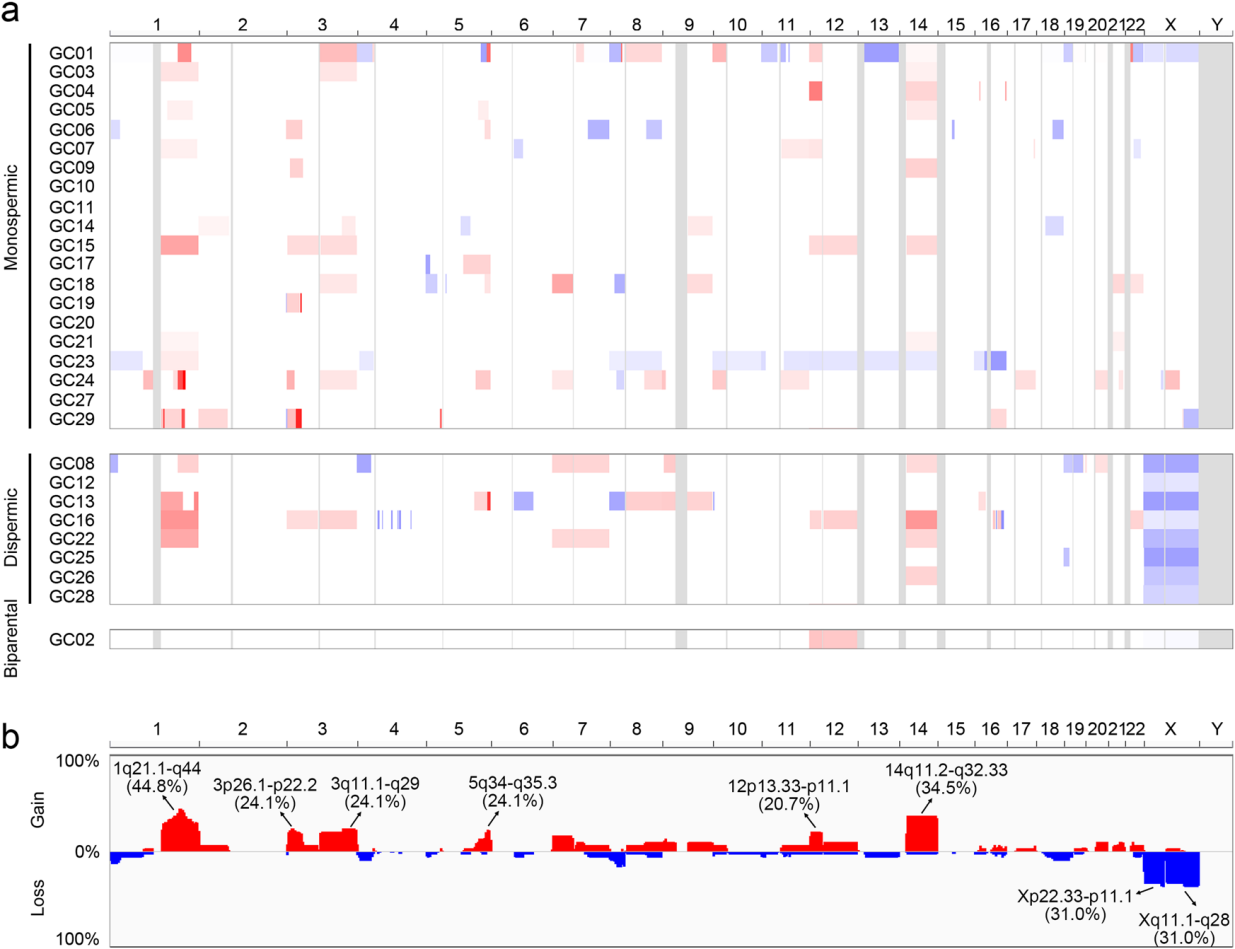
Copy number profiles of GCs. **a** Heat map showing the chromosomal copy gains (red) and losses (blue) in each sample. Boundaries of individual chromosomes are indicated by vertical bars. **b** Frequencies (*y*-axis) of copy number gains and losses across the whole genomes of GC genomes. Red denotes copy number gains, and blue denotes copy number losses.

A total of 185 CNAs (109 gains and 76 losses) were identified in the 29 GCs (Fig. 2a and Supplementary Table 3), corresponding to a median 7.4% fraction of the genome altered (FGA). These results are similar to the rate of uterine endometrial carcinoma (7.2% FGA) but lower than those observed in nongestational germ cell tumors in the testis (37.3% FGA) and other cancers (Supplementary Fig. 2a). Next, we compared our CNA calls from the SNP array with those from WES using the ngCGH module in NEXUS software. The concordance level was estimated by calculating the ratio of overlapping lengths of alterations against total lengths of alterations between the SNP array and WES. We identified that the concordance between them reached 93.6% (range, 79.8–99.9%), suggesting that the CNAs estimated by two independent platforms were in agreement (Supplementary Fig. 3). A majority of the GCs (*n* = 25, 86.2%) harbored at least two CNAs (median of 5 CNAs, range 2–22), whereas four GCs (GC10, 11, 20, and 27) did not harbor any CNAs. Of note, the poor survival group (<5-year survival from GC diagnosis) harbored significantly higher numbers of CNAs than the good survival group (>5-year survival from GC diagnosis) (median of 12.0 vs 3.0 CNAs, *P* = 0.001). There was no significant difference in the number of CNAs between monospermic and dispermic GCs, metastatic and nonmetastatic GCs, or chemotherapy-treated and chemotherapy-naive GCs (*P* > 0.05).

Of the CNAs detected, eight CNA regions (six gains and two losses) were recurrently identified (>6 GCs, 20%) (Fig. 2b and Supplementary Table 4). Compared to other cancers, in which both gains and losses occur at similar rates, GCs harbor more gains than losses (Supplementary Fig. 2b). The most recurrent CNA was an arm-level copy gain on 1q21.1-q44 (44.8%). Arm-level copy gain on 12p13.33-p11.1, where *KRAS* resides, was also detected recurrently (20.7%). The most well-known CNAs for GCs, a gain on 7q21-q31 and a loss on 8p21-p12, were also detected but were not the most common CNAs in our study. Of note, copy gains on 1q21.1-q44 were significantly associated with poor survival (*P* = 0.003). In addition, copy gains on 9q21.11-q34.3 and 21q21.3-q22.3 were associated with metastasis (*P* = 0.015), and chromosome X deletions (p22.33-p11.1 and q11.1-q28) were associated with dispermic origin (*P* = 1.4 × 10^−5^). The frequent chromosome X deletions in the dispermic origin could be explained by the tumor originally being the XY chromosome.

### Mutation profiles

**Fig. 3:**
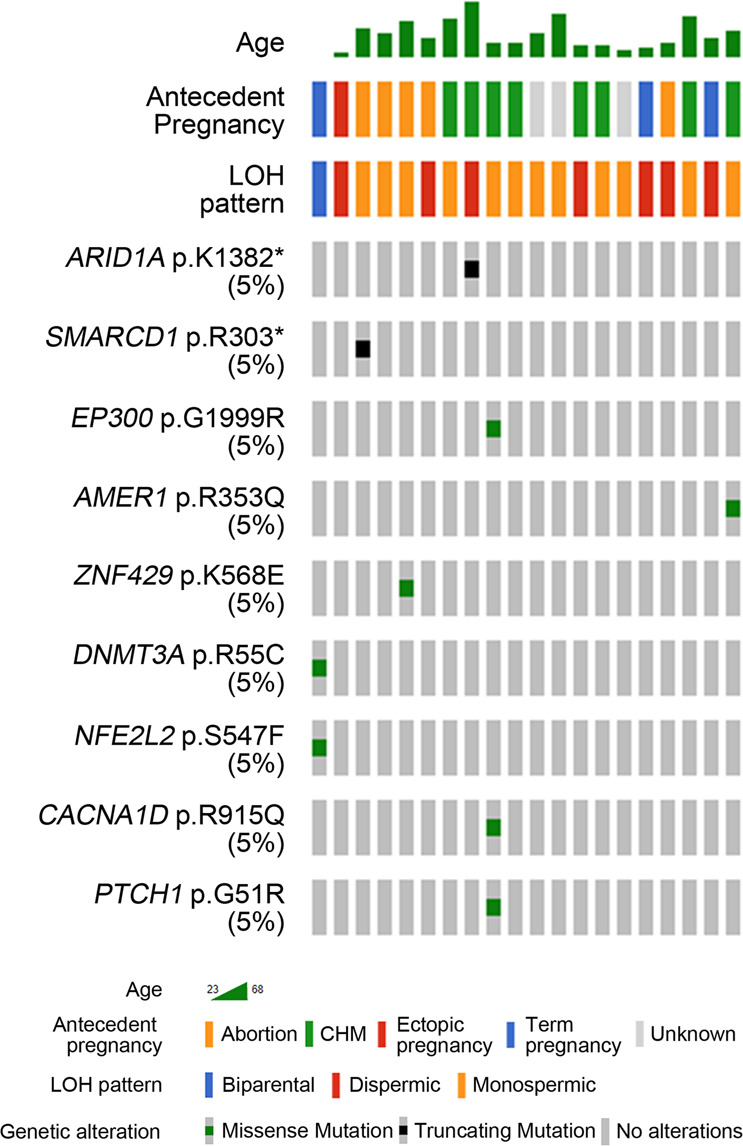
Cancer-related mutations in the GC genomes. Each row represents the mutated gene, and each column represents an individual patient.

Due to the low quality of GC DNA, not all cases (20 of the 29 GCs) were analyzed by WES (107X (range 49–154X) for GC tissues and 122X (range 55–150X) for matched normal tissues) (Supplementary Table 5). A total of 10,964 nonsilent mutations (10,707 SNVs and 257 indels) were identified by applying stringent germline variant filter criteria (Supplementary Table 6). The number of mutations was significantly enriched in GCs with a history of chemotherapy (*P* = 0.036), and 2 GCs (GC08 and 13) obtained after chemotherapy exhibited exceptionally high numbers of mutations. Of the mutated genes detected in our study, nine genes, including *ARID1A* (p.K1382*), *SMARCD1* (p.R303*), *EP300* (p.G1999R), *AMER1* (p.R353Q), and *ZNF429* (p.K568E) have been reported in the COSMIC database and could be considered driver mutations (Fig. 3). Mutations in *ARID1A, SMARCD1*, and *ZNF429* were either hotspot mutations (*ARID1A* and *ZNF429*) or had functional relevance (inactivating mutations in tumor suppressor *SMARCD1*) (https://cancer.sanger.ac.uk/cosmic/mutation)^26^. All of the driver mutations were successfully validated with either digital PCR or Sanger sequencing (Supplementary Fig. 4). These data show that most well-known driver genes in solid cancers, including *TP53* and *KRAS*, may not be common in GCs, but other genes, such as those involved in chromatin remodeling (*ARID1A*, *SMARCD1*, and *EP300*), may represent mutational drivers in GCs.

### Sequential analyses in one patient; hydatidiform mole—invasive mole—choriocarcinoma

One patient (GC15) had consecutive CHM, IM, and GC development, each of which was studied in this study using SNP microarray, STR markers, and WES. The CHM harbored CN-LOH with the two-track BAF plot (100 and 0%) in the SNP array across the whole genome, indicating a monospermic pattern (Fig. 1a). This was confirmed by the STR assay, which showed a single nonmaternal peak for all informative alleles (Fig. 1a and Supplementary Table 2). Both SNP and STR assays also revealed constant patterns (monospermic) among the IM and GC (Fig. 1b, c). Of note, CNAs were evident in GC (Fig. 1c; copy gains on 1q, 3, 12, and 14q) but not in CHM or IM. The CHM, IM, and GC of this patient harbored similar numbers of nonsilent somatic mutations (48, 45, and 57 mutations, respectively) (Supplementary Table 6) without any driver mutations. These results suggest that CNA may play a critical role in HM/IM progression to GC.

## Discussion

Our analyses of GC genomes had two goals. First, we attempted to disclose the genomic alteration profiles of GCs (CN-LOH, CNA, and somatic mutation) that drive tumorigenesis. Second, we wanted to identify any differences in genomic alterations of GCs from those of common (i.e., GC vs. nongestational) cancers. Our data show that CNAs are common genomic alterations in GCs, suggesting that most GCs may occur on the basis of CN-LOH-harboring CHM that may progress to GC by accumulating CNAs. We identified some driver mutations in GCs, but none of them were recurrent. Together, our results indicate that genomic alteration profiles of GCs are different from those of nongestational cancers and suggest that recurrent CNAs appear to be drivers of GC development.

Compared to the reports of HM history to GC (27–80%)^27^, CN-LOH in our study (96.6%) was relatively high. There could be several possible explanations for this disagreement. First, earlier studies to find a causative type of pregnancy might depend on patients’ history but not on pathologic examination, which could underestimate molar pregnancies^28^. In our data, 10 GCs without a history of molar pregnancies but with term/ectopic/abortion pregnancies showed monospermic or dispermic CN-LOH. Second, the discrepancy could arise from sampling bias because our sample size was relatively small.

One GC without any CN-LOH exhibited a diploidic B-allele pattern with abundant SNP mismatches with the patient’s genome, strongly suggesting its biparental origin. This patient had a heterozygous germline mutation in the *NLRP7* gene. However, the *NLRP7* mutation was a novel variant that had not been reported previously (OMIM ID: 609661). In addition, based on the notion that only biallelic mutations in *NLRP7* have been associated with recurrent biparental HMs^25^, this heterozygous mutation may not be associated with GC development. This GC showed no discernible difference in CNAs except a copy gain on chromosome 12.

The present study discovered novel CNAs (gains on 1q, 3p26.1-p22.2, 3q11.1-q29, 5q34-q35.3, 12p, and 14q) as well as previously known CNAs (7q21-q31 gain and 8p21-p12 loss) in GCs. Despite the high sensitivity to chemotherapy for GC, distant metastasis is still an indicator for poor survival^1^. In the present study, we discovered that CNAs might be a biomarker for both poor survival and metastasis of GCs. To our knowledge, our data are the first molecular data for GC prognosis.

GC is a bona fide cancer, but definite cancer genes for GCs are not known^18^, suggesting that GC mutations might not occur in well-known cancer genes. By NGS-based WES, we discovered that GCs harbored a small number of driver mutations, which included chromatin remodeling genes. Our results indicate that the contribution of somatic mutations to GC development may be different in quantity (driver mutation numbers) and quality (mutated gene functions) compared to other cancers. Recurrent inactivating mutations or losses/deletions of *ARID1A* have been found in many cancers (https://cancer.sanger.ac.uk/cosmic/mutation)^29^, suggesting the role of *ARID1A* as a tumor suppressor gene (TSG). In addition to an *ARID1A* frameshift mutation in one case (Fig. 3), another GC (GC06 and 23) showed copy losses at the *ARID1A* locus (2/29 GCs, Fig. 2a), suggesting that *ARID1A* inactivation by genetic alterations might be a common event in GCs (3/29, 10.3%). *ZNF429* encodes a transcription factor^30^, but its exact role in cancer pathogenesis remains unknown. The *ZNF429* p.K568E mutation detected in our study is the second most common variant (35/312 missense mutations) in the COSMIC database and has been identified solely in glioblastomas (https://cancer.sanger.ac.uk). *SMARCD1* interacts with p53, and their uncoupling results in inhibition of p53-dependent apoptosis and cell cycle arrest, suggesting that *SMARCD1* is a putative TSG^31^. An inactivating mutation of *SMARCD1* (p.R303*) detected in the present study might inactivate the TSG functions of *SMARCD1*. Of note, mutations in *ARID1A*, *SMARCD1,* and *EP300* detected in the present study are chromatin remodeling genes^31^, suggesting that alterations of chromatin remodeling might be involved in GC tumorigenesis.

In sequential samples (HM-IM-GC), we found evidence that GTDs might be clonal with a constant CN-LOH pattern. Neither the HM nor IM in this patient harbored CNAs, whereas the GC harbored several CNAs but not driver mutations. Taken together, these findings indicate that CN-LOH is an early pivotal event in HM-IM-GC development and that CNA is a late event that may promote HM/IM progression to GC. In addition, common genomic profiles between the HM and IM (constant CN-LOH, no CNAs, and no driver mutations) suggest that the progressed phenotype of IM (invasion and dissemination) might be caused by nongenetic factors.

The present study identified integrative genetic characteristics of GCs for the first time. We also evaluated genetic alterations in relation to clinical data and found that CNAs could be an important factor for disease progression or for poor prognosis in GCs. The present study provides clues for understanding the etiology of GC, properly diagnosing HM-IM-GC, and selecting patients with GC who might require intensive treatments against poor prognosis.

## Supplementary information

Supplementary information

## Data Availability

The datasets supporting the conclusions of this articleare included within the article and its additional files. Rawdata (sequences) were deposited in the SRA database(Project ID: PRJNA636516).
