# Supplementary material for: Distinct genomic profiles of gestational choriocarcinoma, a unique cancer of pregnant tissues
Source: Exp Mol Med. 2020 Dec 15;52(12):2046–54. doi: 10.1038/s12276-020-00544-0 (PMC8080714; doi:10.1038/s12276-020-00544-0)
Supplement: Supplementary file 1 — Supplementary information [file 12276_2020_544_MOESM1_ESM.pdf]

Supplementary Figures

GC01 (Type1: mono-spermic CHM)

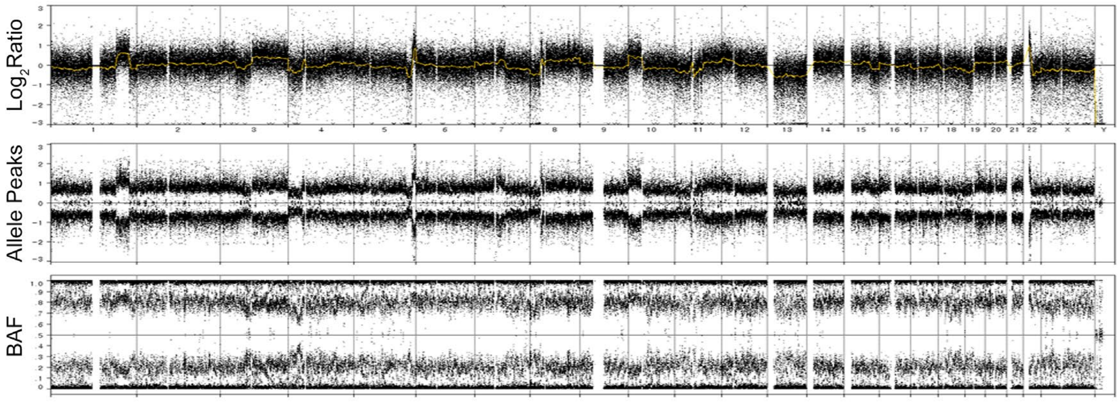

GC02 (Type3: bi-parental)

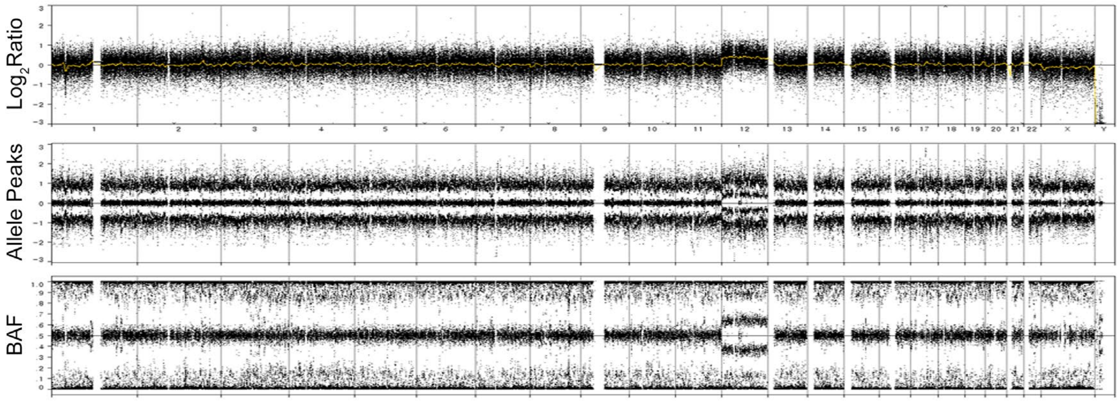

GC03 (Type1: mono-spermic CHM)

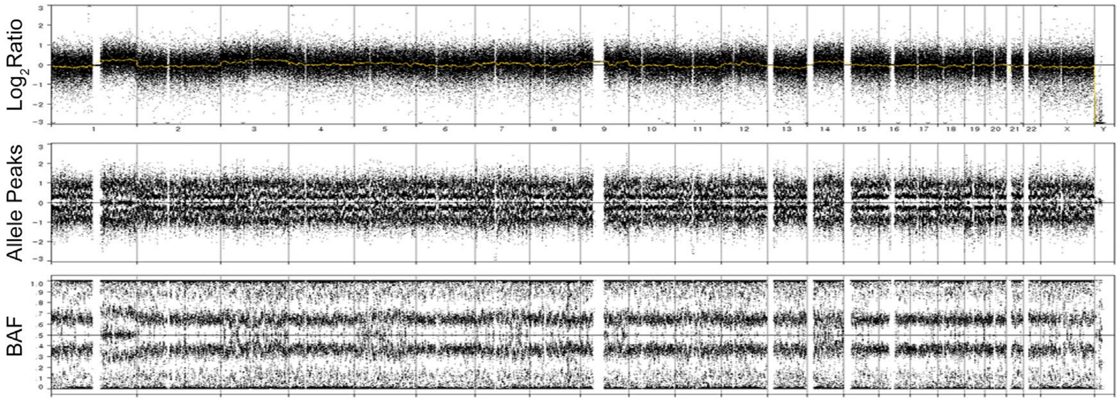

GC04 (Type1: mono-spermic CHM)

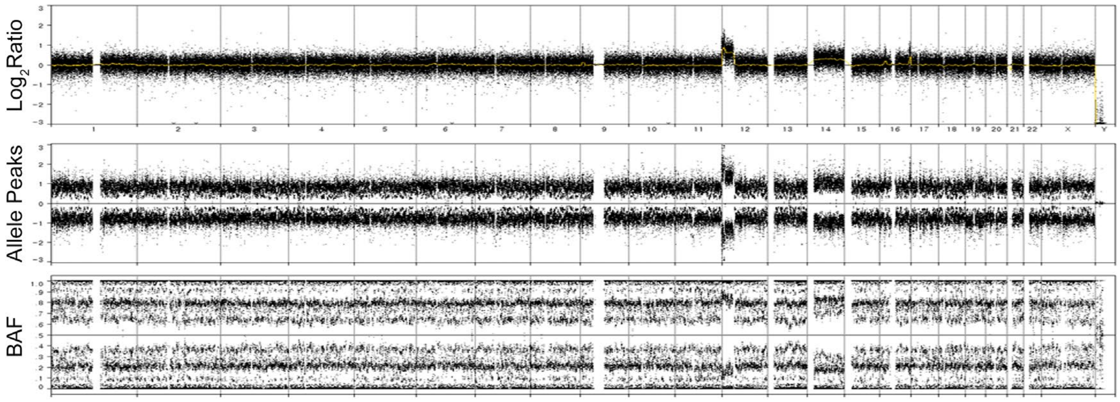

GC05 (Type1: mono-spermic CHM)

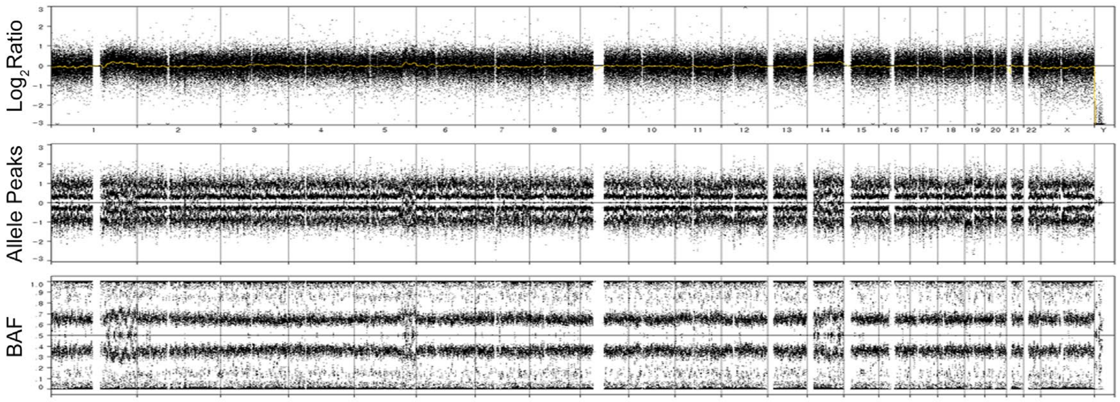

GC06 (Type1: mono-spermic CHM)

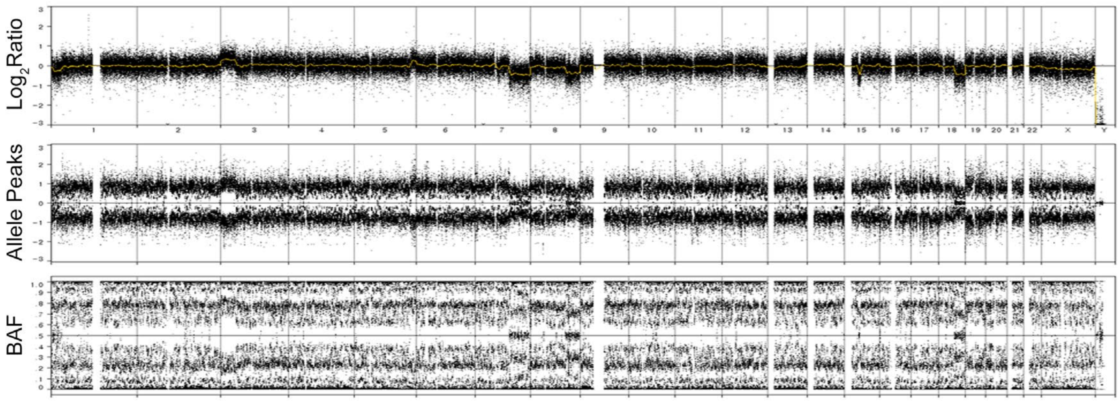

GC07 (Type1: mono-spermic CHM)

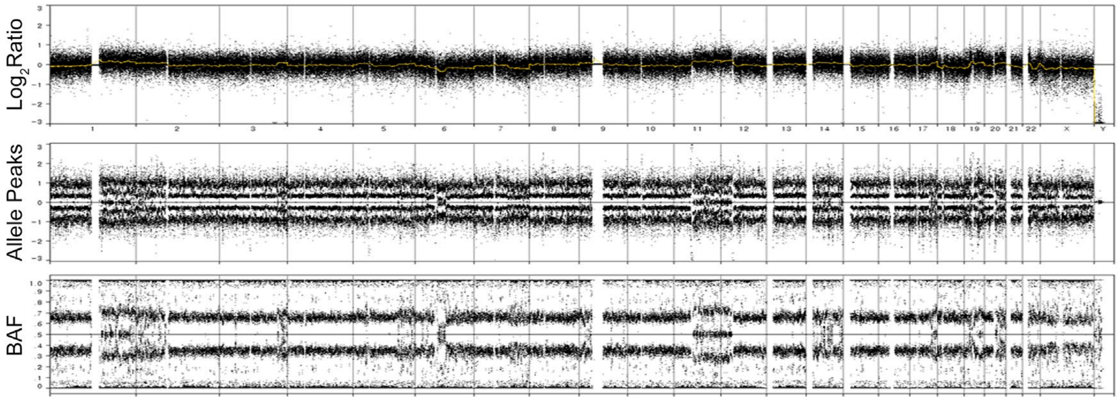

GC08 (Type2: di-spermic CHM)

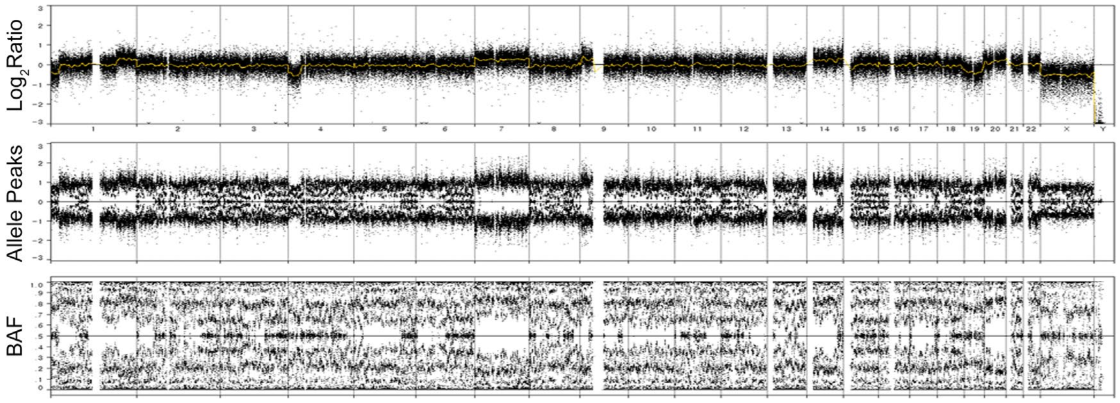

GC09 (Type1: mono-spermic CHM)

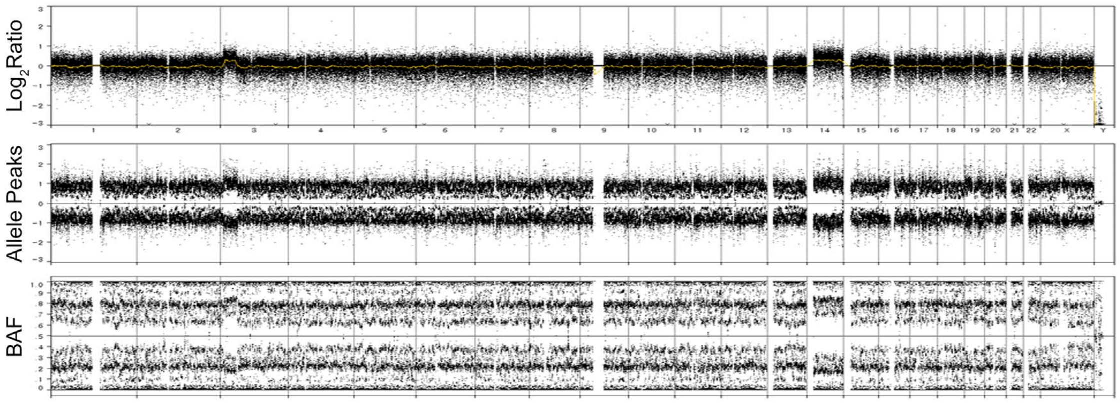

GC10 (Type1: mono-spermic CHM)

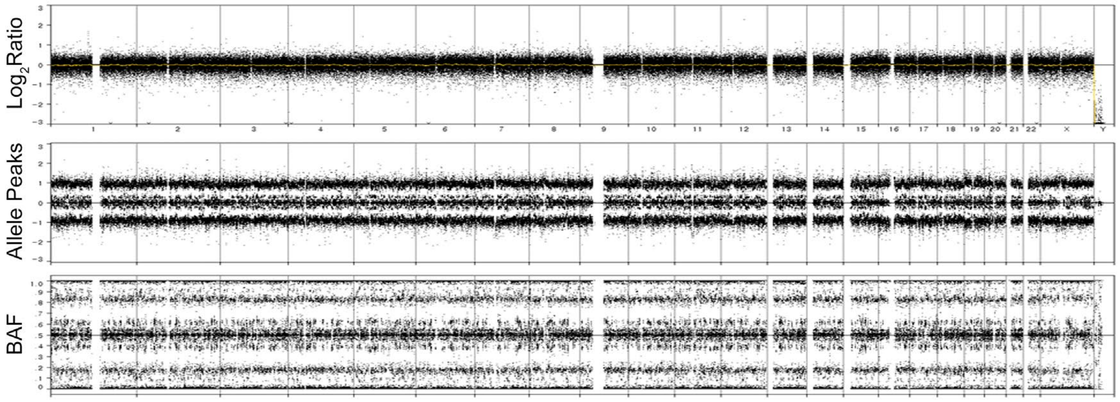

GC11 (Type1: mono-spermic CHM)

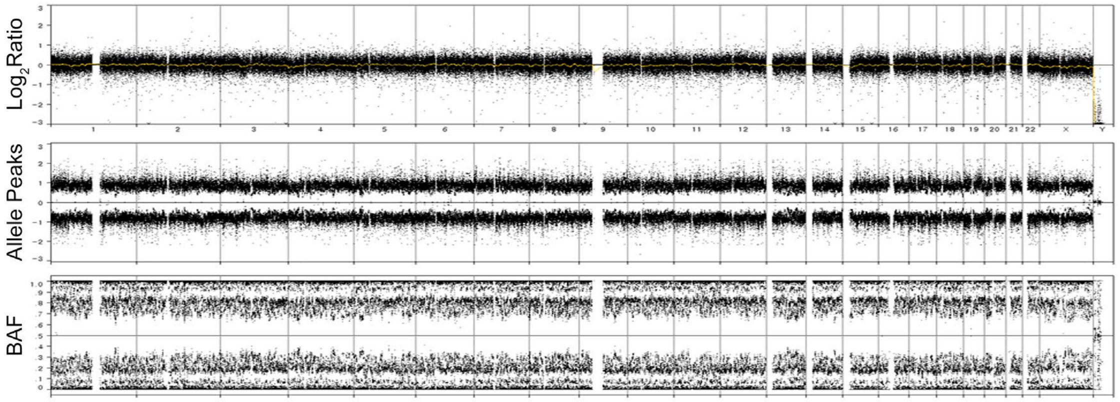

GC12 (Type2: di-spermic CHM)

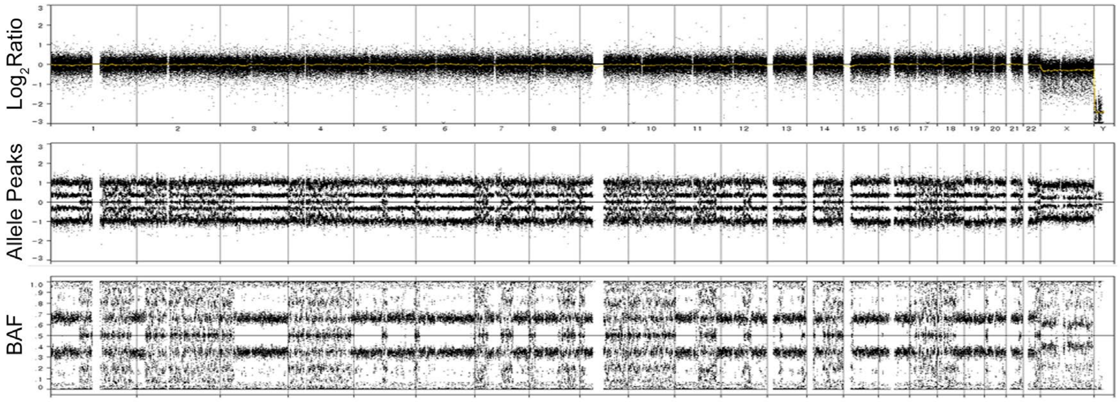

GC13 (Type2: di-spermic CHM)

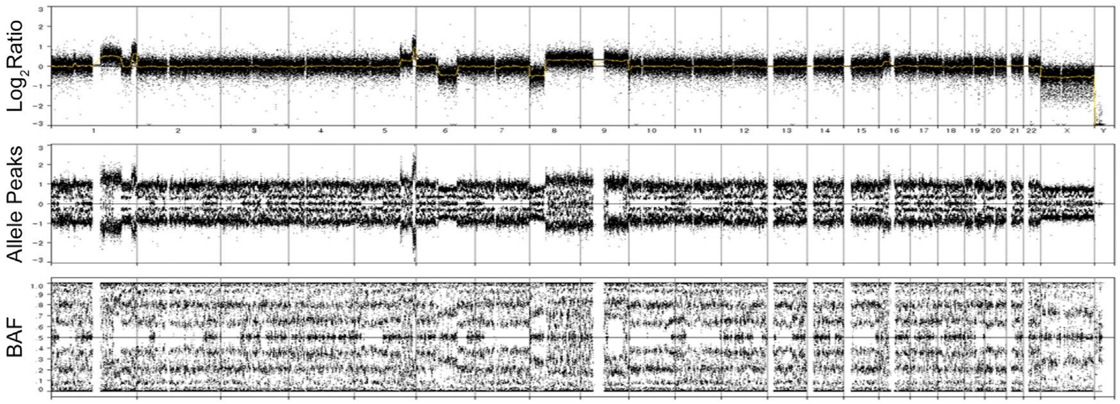

GC14 (Type1: mono-spermic CHM)

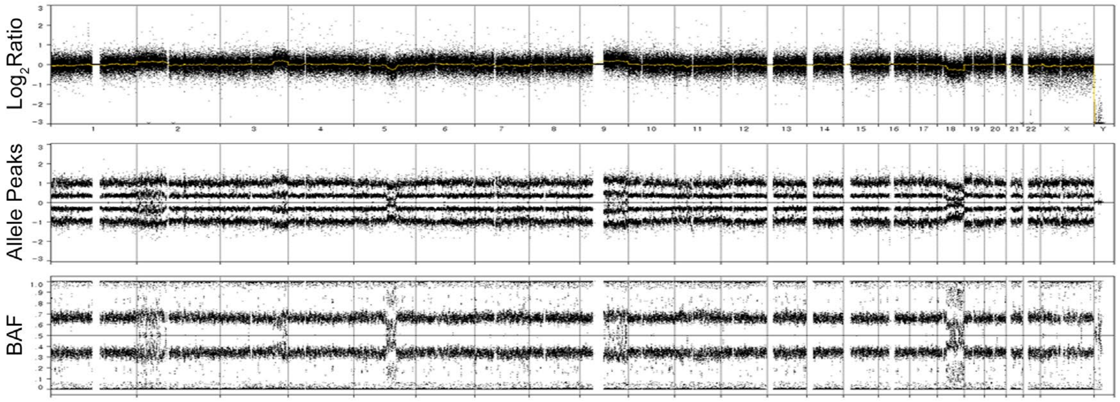

GC15 (Type1: mono-spermic CHM)

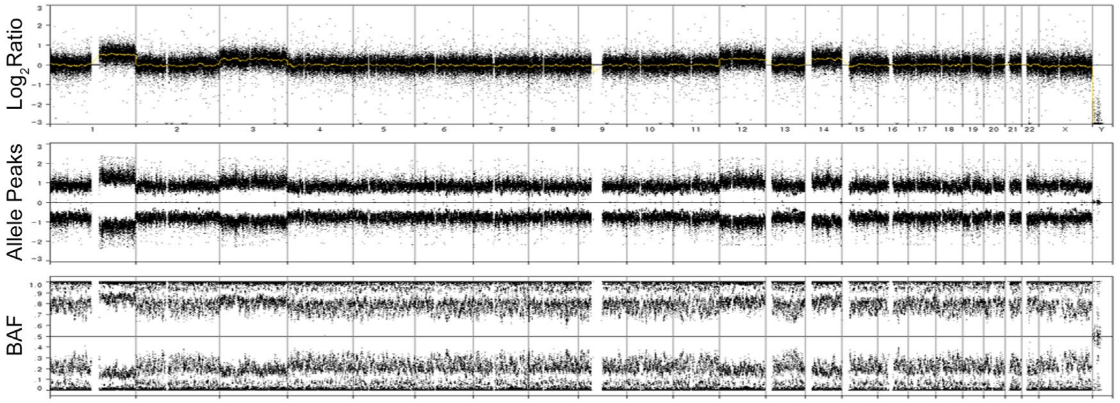

GC16 (Type2: di-spermic CHM)

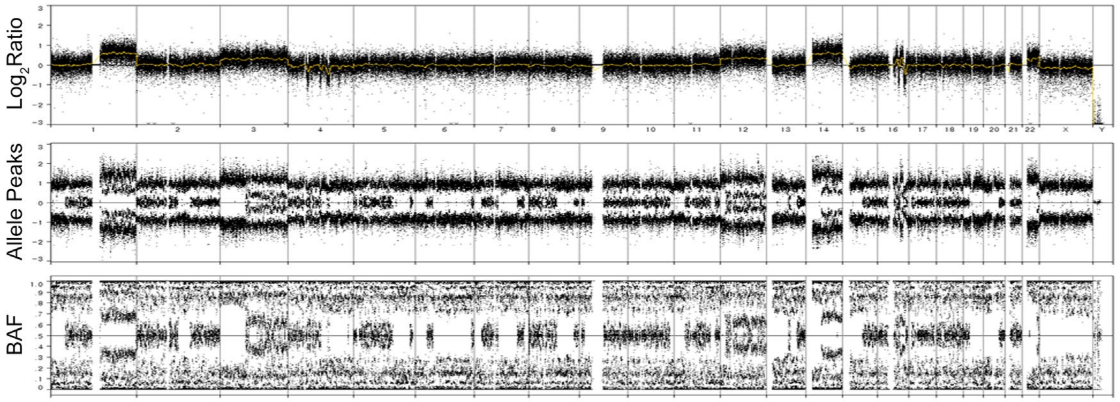

GC17 (Type1: mon-ospermic CHM)

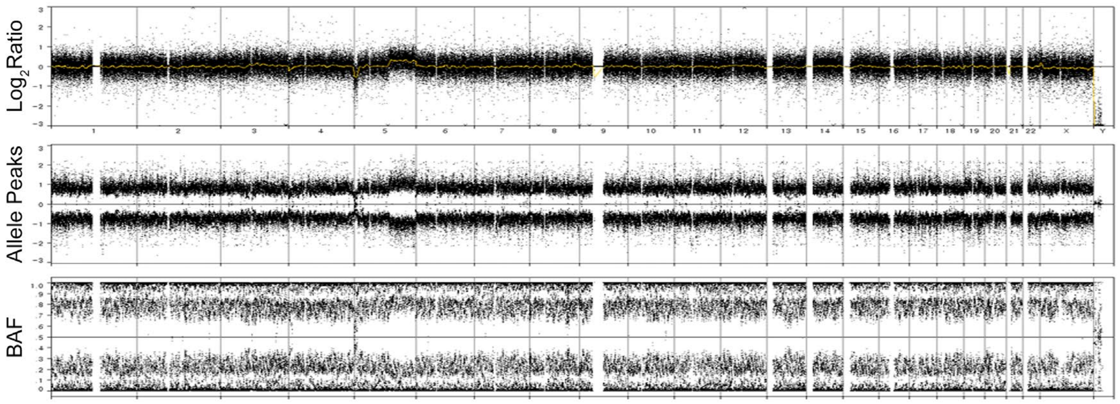

GC18 (Type1: mono-spermic CHM)

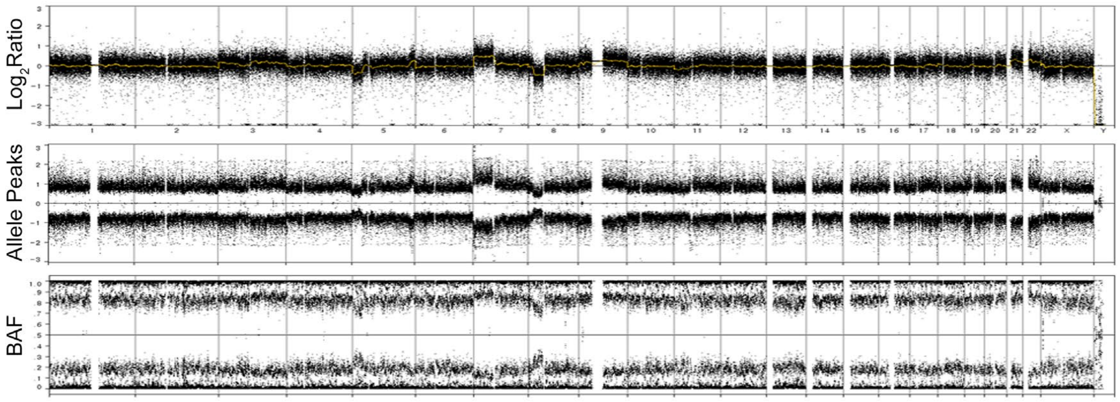

GC19 (Type1: mono-spermic CHM)

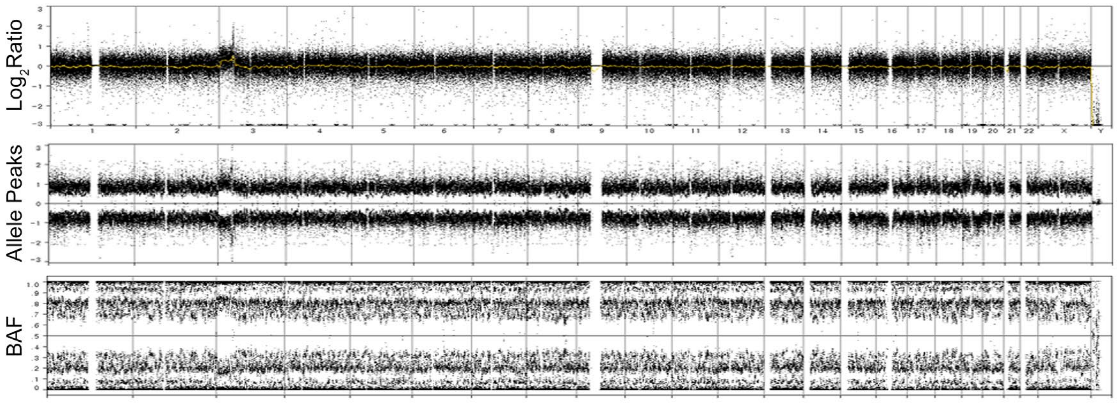

GC20 (Type1: mono-spermic CHM)

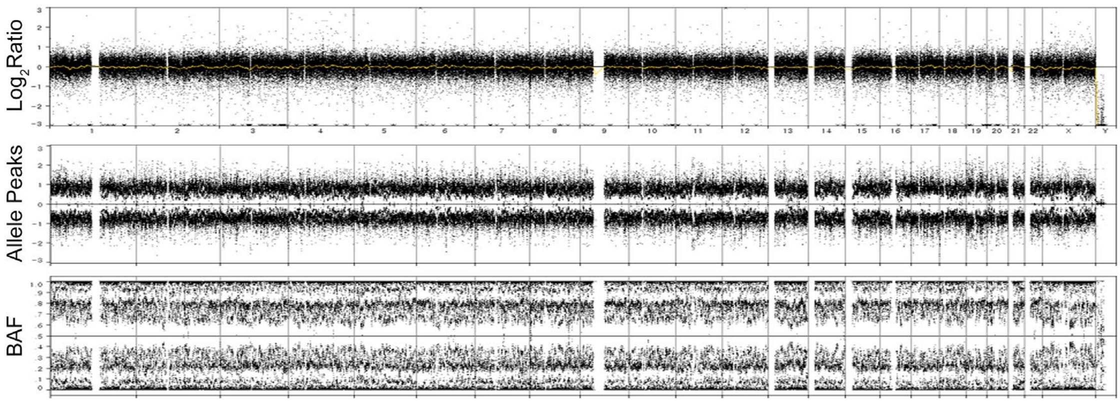

GC21 (Type1: mono-spermic CHM)

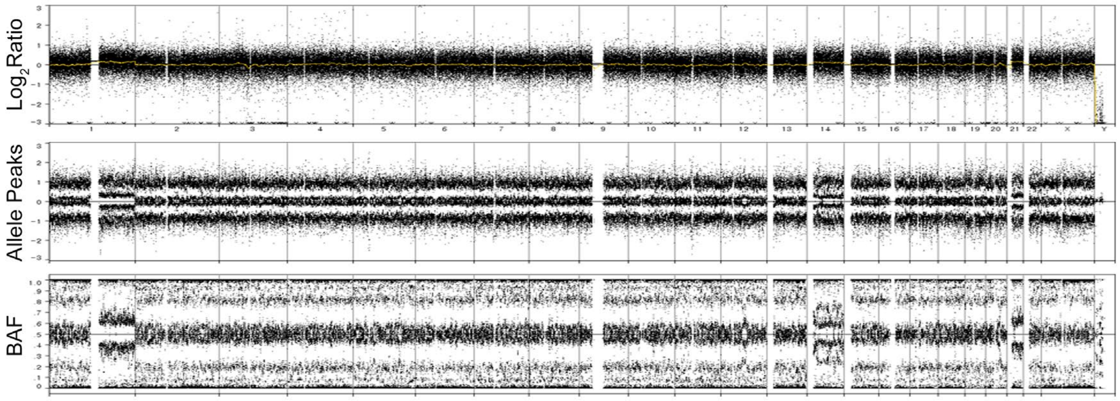

GC22 (Type2: di-spermic CHM)

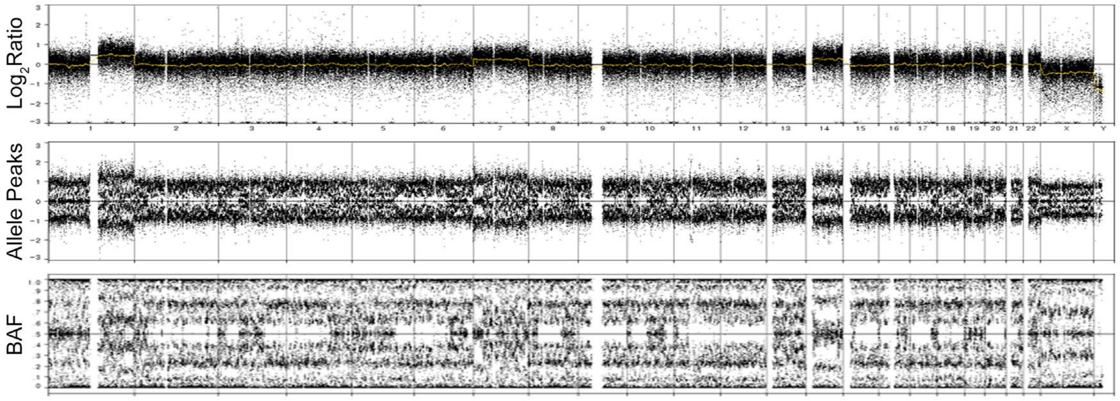

GC23 (Type1: mono-spermic CHM)

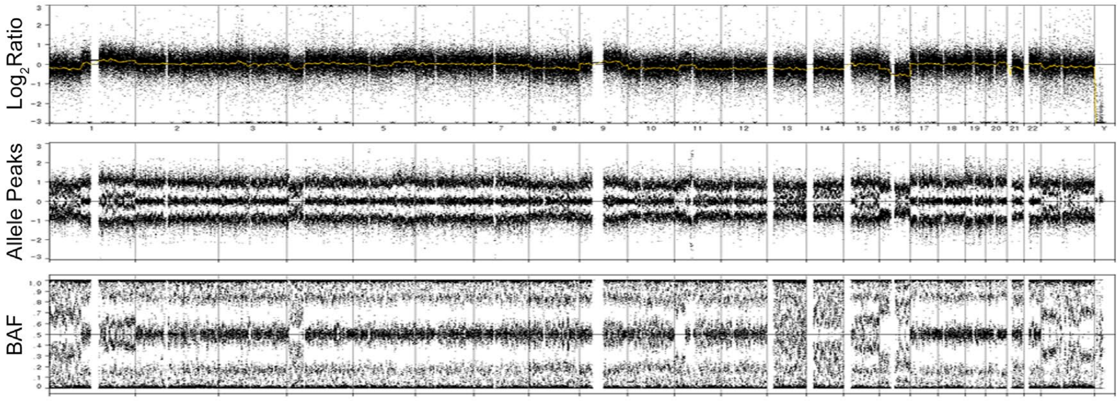

GC24 (Type1: mono-spermic CHM)

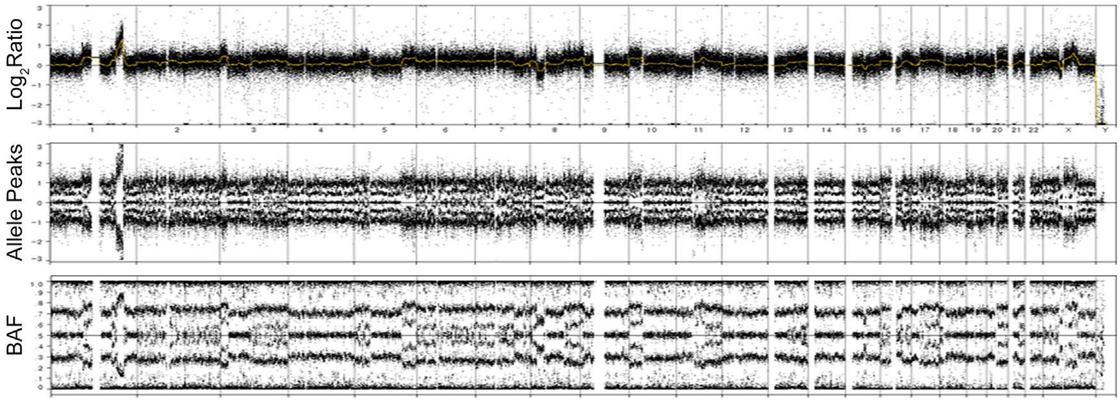

GC25 (Type2: di-spermic CHM)

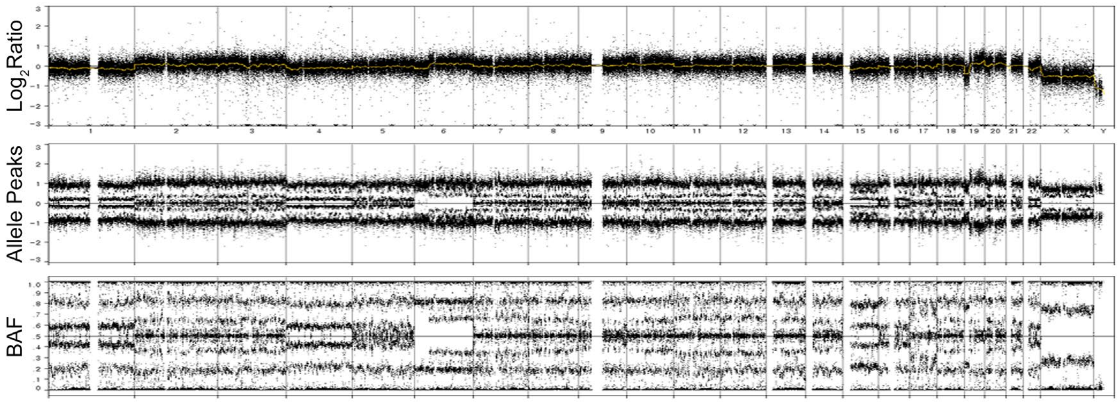

GC26 (Type2: di-spermic CHM)

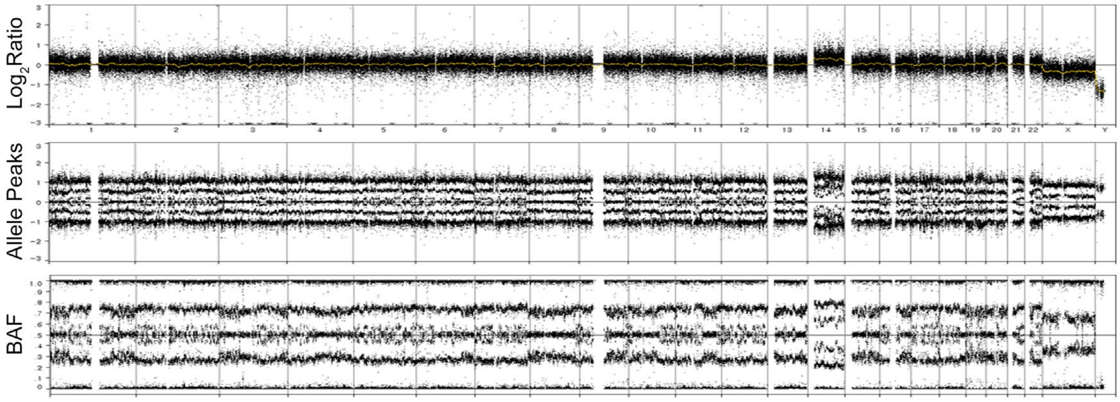

GC27 (Type1: mono-spermic CHM)

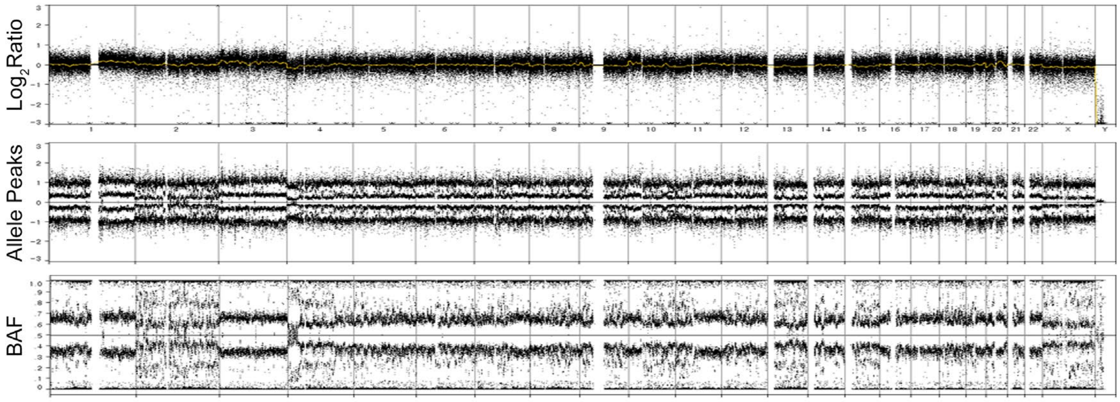

GC28 (Type2: di-spermic CHM)

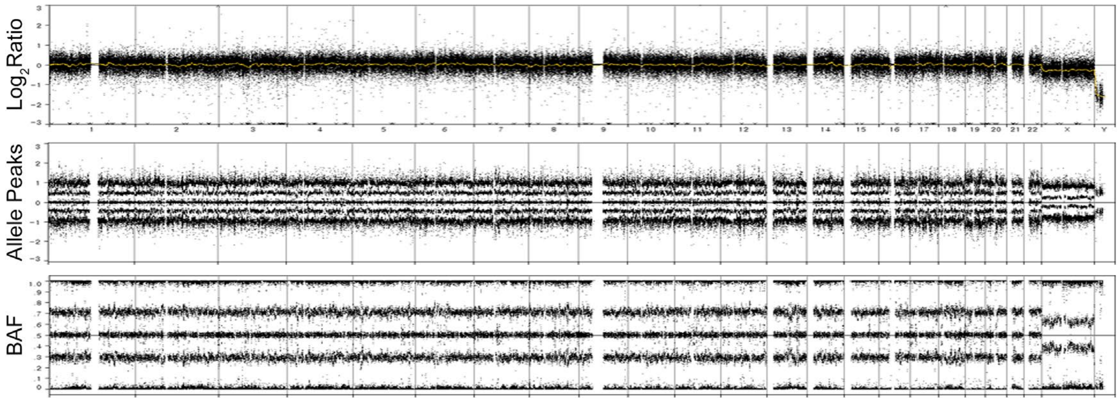

GC29 (Type1: mono-spermic CHM)

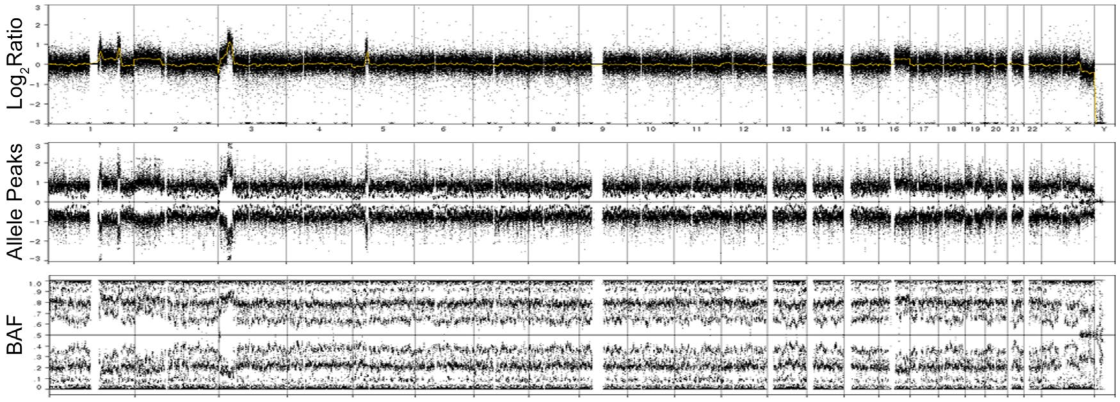

Supplementary Fig. S1. CN-LOH patterns of 29 GCs. B-allele patterns are analyzed by an SNP microarray. For each GC, Log_2Ratio (upper), allele peak (middle), and B-allele frequency (lower) are shown.

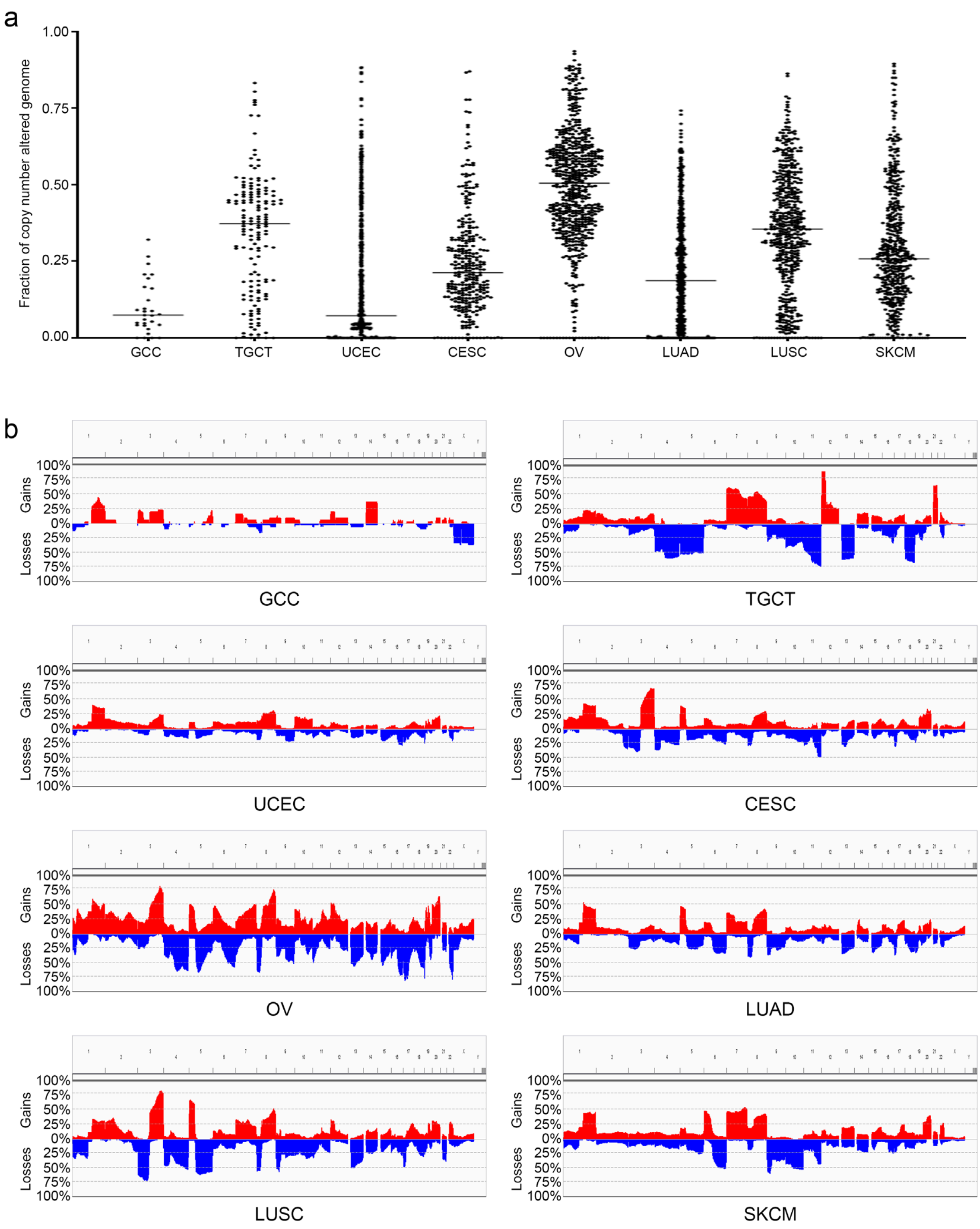

Supplementary Fig. S2. Fraction of the genome altered (FGA). (a) Comparison of FGA rates between GC and other cancer genomes. (b) Frequencies (y-axis) of copy number gains and losses across the whole genomes of GC genomes. Red denotes copy number gains and blue denotes copy number losses. FGA rates and CNA frequencies are from the Cancer Genome Atlas (TCGA) consortium. (TGCT, Testicular Germ Cell Tumors; UCEC, Uterine Corpus Endometrial Carcinoma; CESC, Cervical squamous cell carcinoma and endocervical adenocarcinoma; OV, Ovarian serous cystadenocarcinoma; LUAD, Lung adenocarcinoma; LUSC, Lung squamous cell carcinoma; SKCM, Skin Cutaneous Melanoma

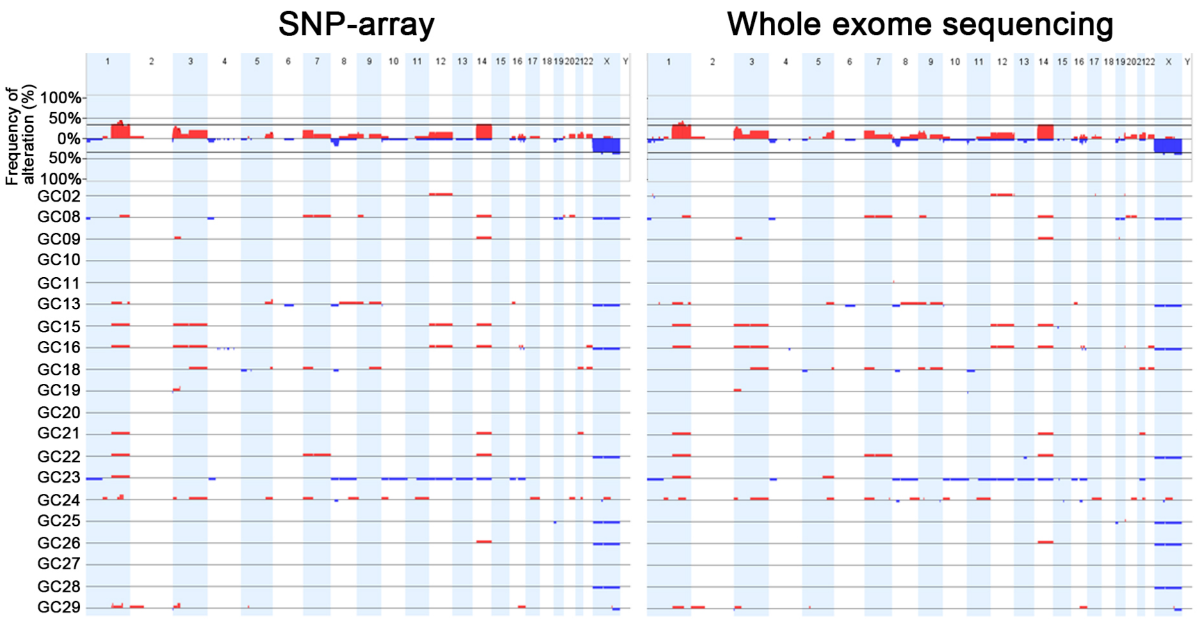

	GC02	GC08	GC09	GC10	GC11	GC13	GC15	GC16	GC18	GC19	GC20	GC21	GC22	GC23	GC24	GC25	GC26	GC27	GC28	GC29
Concordance (%)	85.2%	93.8%	95.3%	N/A	N/A	95.3%	97.2%	96.5%	79.8%	85.7%	N/A	99.1%	96.1%	90.1%	91.9%	95.1%	98.4%	N/A	99.9%	97.4%

Supplementary Fig. S3. Comparison of CNA calls from SNP array and WES. Copy number gains and losses identified from SNP-assay (left) and WES (right) are shown. Red denotes copy number gains and blue denotes copy number losses. The concordance level was estimated by calculating the correlation for overlapped lengths of alterations between SNP array and WES. The concordance between the two platforms reached to 93.6% (range, 79.8-99.9%).

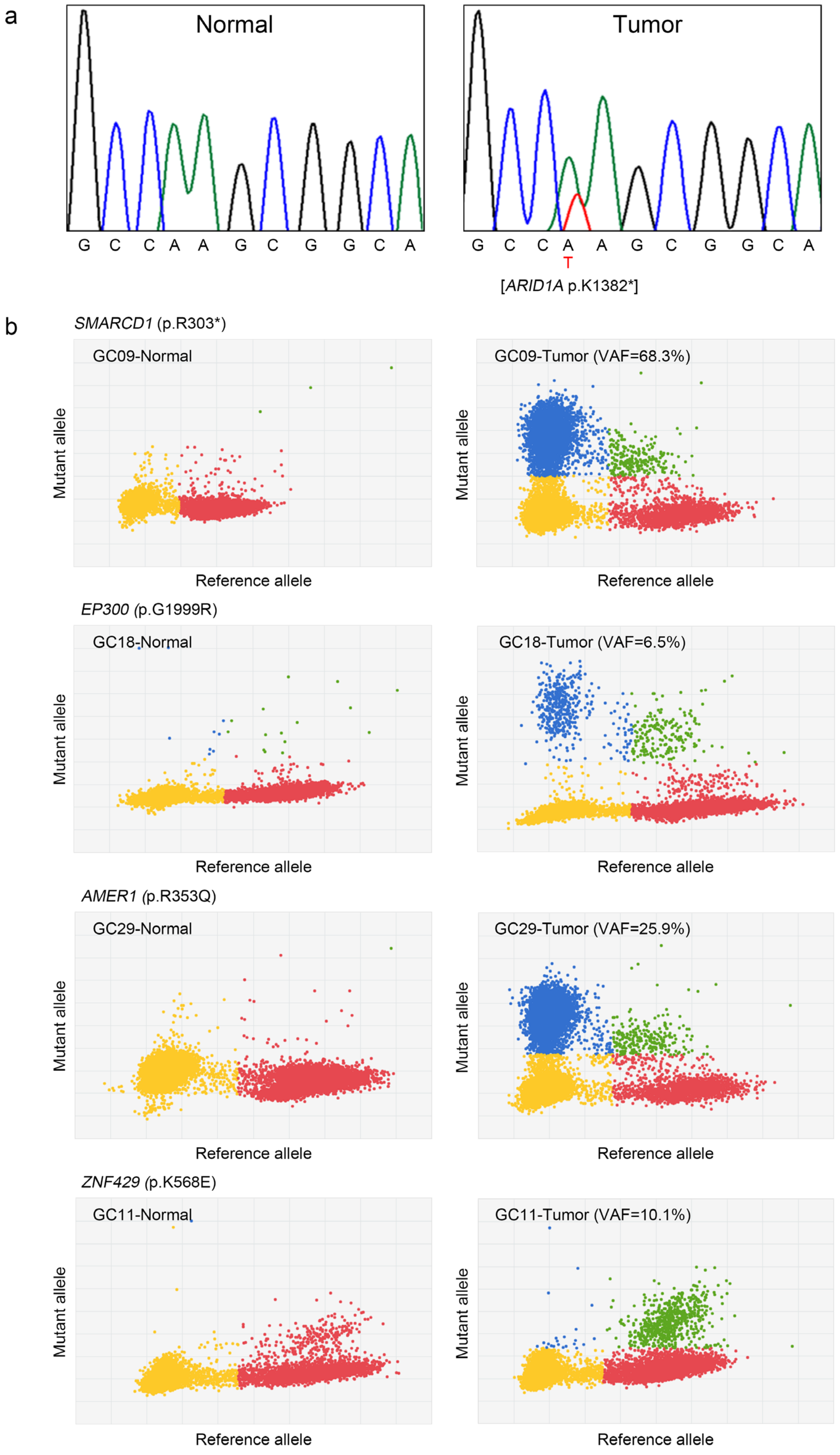

Supplementary Fig. S4. Validation of the mutations. (a) Sanger sequencing of *ARID1A* gene in tumor with matched normal are shown. *ARID1A* p.K1382* mutation was validated as somatic. (b) Digital PCR of 4 genes (*SMARCD1*, *EP300*, *AMER1*, and *ZNF429*) in tumor with matched normal are shown. Red and blue dot represent the reference allele and mutant allele, respectively. All mutations were validated as somatic.

Supplementary Table S1. Expected B-allele frequencies for various levels of normal cell contamination

Type	GC purity	BAF1	BAF2	BAF3	BAF4	BAF5	BAF6	BAF7	BAF8	BAF9
Diploid mono-spermic CHM	100	1	1	1	0	0	0			
Diploid mono-spermic CHM	95	1	0.975	0.95	0.05	0.025	0			
Diploid mono-spermic CHM	90	1	0.95	0.9	0.1	0.05	0			
Diploid mono-spermic CHM	85	1	0.925	0.85	0.15	0.075	0			
Diploid mono-spermic CHM	80	1	0.9	0.8	0.2	0.1	0			
Diploid mono-spermic CHM	75	1	0.875	0.75	0.25	0.125	0			
Diploid mono-spermic CHM	70	1	0.85	0.7	0.3	0.15	0			
Diploid mono-spermic CHM	65	1	0.825	0.65	0.35	0.175	0			
Diploid mono-spermic CHM	60	1	0.8	0.6	0.4	0.2	0			
Diploid mono-spermic CHM	55	1	0.775	0.55	0.45	0.225	0			
Diploid mono-spermic CHM	50	1	0.75	0.5	0.5	0.25	0			
Diploid mono-spermic CHM	45	1	0.725	0.45	0.55	0.275	0			
Diploid mono-spermic CHM	40	1	0.7	0.4	0.6	0.3	0			
Diploid mono-spermic CHM	35	1	0.675	0.35	0.65	0.325	0			
Diploid mono-spermic CHM	30	1	0.65	0.3	0.7	0.35	0			
Diploid mono-spermic CHM	25	1	0.625	0.25	0.75	0.375	0			
Diploid mono-spermic CHM	20	1	0.6	0.2	0.8	0.4	0			
Diploid mono-spermic CHM	15	1	0.575	0.15	0.85	0.425	0			
Diploid mono-spermic CHM	10	1	0.55	0.1	0.9	0.45	0			
Diploid mono-spermic CHM	5	1	0.525	0.05	0.95	0.475	0			
Diploid mono-spermic CHM	0	1	0.5	0	1	0.5	0			
Diploid di-spermic CHM	100	1	1	1	0.5	0.5	0.5	0	0	0
Diploid di-spermic CHM	95	1	0.975	0.95	0.525	0.5	0.475	0.05	0.025	0
Diploid di-spermic CHM	90	1	0.95	0.9	0.55	0.5	0.45	0.1	0.05	0
Diploid di-spermic CHM	85	1	0.925	0.85	0.575	0.5	0.425	0.15	0.075	0
Diploid di-spermic CHM	80	1	0.9	0.8	0.6	0.5	0.4	0.2	0.1	0
Diploid di-spermic CHM	75	1	0.875	0.75	0.625	0.5	0.375	0.25	0.125	0
Diploid di-spermic CHM	70	1	0.85	0.7	0.65	0.5	0.35	0.3	0.15	0
Diploid di-spermic CHM	65	1	0.825	0.65	0.675	0.5	0.325	0.35	0.175	0

Type	GC purity	BAF1	BAF2	BAF3	BAF4	BAF5	BAF6	BAF7	BAF8	BAF9
Diploid di-spermic CHM	60	1	0.8	0.6	0.7	0.5	0.3	0.4	0.2	0
Diploid di-spermic CHM	55	1	0.775	0.55	0.725	0.5	0.275	0.45	0.225	0
Diploid di-spermic CHM	50	1	0.75	0.5	0.75	0.5	0.25	0.5	0.25	0
Diploid di-spermic CHM	45	1	0.725	0.45	0.775	0.5	0.225	0.55	0.275	0
Diploid di-spermic CHM	40	1	0.7	0.4	0.8	0.5	0.2	0.6	0.3	0
Diploid di-spermic CHM	35	1	0.675	0.35	0.825	0.5	0.175	0.65	0.325	0
Diploid di-spermic CHM	30	1	0.65	0.3	0.85	0.5	0.15	0.7	0.35	0
Diploid di-spermic CHM	25	1	0.625	0.25	0.875	0.5	0.125	0.75	0.375	0
Diploid di-spermic CHM	20	1	0.6	0.2	0.9	0.5	0.1	0.8	0.4	0
Diploid di-spermic CHM	15	1	0.575	0.15	0.925	0.5	0.075	0.85	0.425	0
Diploid di-spermic CHM	10	1	0.55	0.1	0.95	0.5	0.05	0.9	0.45	0
Diploid di-spermic CHM	5	1	0.525	0.05	0.975	0.5	0.025	0.95	0.475	0
Diploid di-spermic CHM	0	1	0.5	0	1	0.5	0	1	0.5	0

Supplementary Table S2. STR marker analysis for 24 gestational choriocarcinomas and their matched normal tissues

Sample ID	Tissue	D8S1179	D21S11	D7S820	CSF1PO	D3S1358	TH01	D13S317	D16S539	D2S1338	D19S433	vWA	TPOX	D18S51	Amelogenin	D5S818	FGA
GC01	N	10/12	29/32.2	NA	NA	16/16	7/9.3	8/11	11/12	NA	13/15.2	14/19	8/11	12/14	X	10/15	24/25
	T	10/12/13	NA	NA	NA	16/18	NA	NA	NA	NA	13/13	17/17	NA	NA	X	11/11	NA
GC04	N	11/14	NA	NA	NA	15/16	6/7	NA	NA	NA	14/14	14/14	NA	NA	X	10/11	NA
	T	11/14/15	NA	NA	NA	14/15/16	6/7/9	NA	NA	NA	14/14.2	14/15	NA	NA	X	10/11	NA
GC05	N	10/11	NA	NA	NA	16/16	NA	NA	NA	NA	13/15.2	NA	NA	NA	X	NA	NA
	T	10/16	NA	NA	NA	16/17	NA	NA	NA	NA	13/15.2	NA	NA	NA	X	NA	NA
GC07	N	NA	NA	NA	NA	NA	NA	NA	NA	NA	13.2/16.2	12/15	NA	NA	X	NA	NA
	T	NA	NA	NA	NA	NA	NA	NA	NA	NA	13/13.2	NA	NA	NA	X	NA	NA
GC08	N	NA	NA	NA	NA	16/16	NA	NA	NA	NA	13/13	14/14	NA	NA	X	NA	NA
	T	NA	NA	NA	NA	14/16/17	NA	NA	NA	NA	13/14	NA	NA	NA	X	NA	NA
GC09	N	12/12	28/29	NA	NA	15/16	6/9	8/11	9/10	NA	14/14	14/18	8/8	NA	X	10/11	23/23
	T	12/12	28/29/30	NA	NA	15/16	6/9	8/11	NA	NA	14/15	14/17/18	8/11	NA	X	10/11	21/23
GC10	N	10/15	29.2/33	11/12	10/12	15/16	7/9	11/12	10/12	19/23	13/14	14/18	8/8	13/20	X	11/13	20/21
	T	10/11/15	29.2/30/33	NA	12/12	15/16/17	7/9	11/12	10/12/13	NA	13/14	14/17/18	8/11	13/20	X	11/13	19/20/21
GC11	N	13/15	30/30	NA	NA	16/16	9/9	8/14	9/10	NA	13/14	14/17	8/9	NA	X	10/10	18/24
	T	12/13/15	30/33.2	NA	NA	16/16	9/9	8/11/14	9/10/13	NA	13/14/14.2	14/16/17	8/9	14/14	X	10/13	18/19/24
GC12	N	10/13	31/31	10/12	9/12	15/15	8/9	11/13	9/9	22/23	13/14	17/19	8/11	18/20	X	11/12	21/24
	T	10/13	30/31	10/11/12	9/12	15/16	7/8/9	11/12/13	9/9	18/22/23/24	13/14/14.2/15.2	16/17/19	8/11	8/14/18/20	XY	10/11/12	19/21/24
GC13	N	13/14	29/29.2	NA	NA	16/16	6/9	8/13	11/11	NA	14/14	16/17	8/11	15/15	X	10/12	21/22
	T	13/14	29/29	NA	12/12	16/16	6/9/10	8/9/13	10/11	NA	13/14/16.2	14/16/17	8/8	13/15/22	X	10/12	20/21/22/24
GC14	N	12/15	30/30	11/12	10/12	15/16	6/6	12/13	10/11	20/23	14.2/15.2	16/18	11/11	15/16	X	9/10	23/23
	T	12/15	30/30	8/11/12	10/12	14/15/16	6/9	8/12/13	9/10/11	20/23	14/14.2/15.2	15/16/18	11/11	13/15/16	X	9/10/13	23/25
GC15	N	12/15	30/32.2	11/11	9/12	16/16	7/8	10/11	11/12	NA	13/14.2	14/16	8/11	13/15	X	10/10	21/21
	HM	15/15	30/30	12/12	10/10	16/16	9/9	13/13	9/9	19/19	13/14/14.2	17/17	8/8	12/12	X	12/12	25/25
	IM	15/15	30/30	NA	NA	16/16	9/9	13/13	9/9	NA	13/14/14.2	17/17	8/8	NA	X	12/12	NA
	T	12/15	30/30	11/12	9/10/12	16/16	7/8/9	10/11/13	9/11/12	NA	13/14/14.2	14/16/17	8/11	12/13/15	X	10/12	21/25
GC16	N	10/15	30/32.2	11/11	10/12	16/17	7/9	10/11	9/9	22/24	14.2/15.2	16/19	8/11	14/15	X	11/11	20/23
	T	10/14/15	30/32.2/33.2	9/11	12/12	15/16/17	6/7/9	8/10/11	9/11	18/19/22/24	13/14.2/15.2	14/16/19	8/10/11	13/14/15	X	11/12	23/23

Sample ID	Tissue	D8S1179	D21S11	D7S820	CSF1PO	D3S1358	TH01	D13S317	D16S539	D2S1338	D19S433	vWA	TPOX	D18S51	Amelogenin	D5S818	FGA
GC17	N	11/13	NA	NA	NA	15/17	NA	NA	NA	NA	13/15.2	17/18	NA	NA	X	11/11	NA
	T	11/13	NA	NA	NA	15/16/17	NA	NA	NA	NA	12.2/13/15.2	14/17	NA	NA	X	11/11	NA
GC18	N	13/15	29/30	8/12	NA	15/15	7/7	10/12	9/11	18/19	13/15.2	18/20	8/8	17/22	X	7/11	22/24
	T	11/11	32.2/32.2	11/11	NA	15/15	9/9	8/8	13/13	NA	13/13	14/14	8/8	18/18	X	11/11	19/19
GC19	N	14/17	30/31.2	NA	NA	14/15	7/9	8/12	NA	NA	14/14.2	14/16	8/8	NA	X	10/11	19/25.2
	T	13/13	NA	NA	NA	15/17	9/9	NA	NA	NA	13/13	14/14	NA	NA	X	11/11	NA
GC20	N	NA	28/29	NA	NA	14/17	5/8	NA	NA	NA	12/13	15/18	7/10	NA	X	10/13	NA
	T	NA	30/30	NA	NA	17/17	9/9	NA	NA	NA	13/14	17/17	9/9	NA	X	13/13	NA
GC21	N	13/14	NA	NA	NA	15/16	7/9	10/10	NA	NA	12/14.2	17/17	NA	NA	X	10/12	NA
	T	13/14/15	NA	NA	NA	15/16	7/7	9/9	NA	NA	12/14.2/15.2	17/17	NA	NA	X	10/10	NA
GC23	N	15/16	30/31.2	11/12	12/12	15/18	7/9	10/11	10/12	NA	13/14.2	14/16	8/8	13/15	X	11/11	20/24
	T	15/16	30/31/31.2	NA	12/12	15/18	7/10	10/12	10/10	NA	13/14/14.2	14/16/17	8/8	NA	X	10/11	20/24/25
GC24	N	10/12	30/30	NA	NA	16/17	7/9	9/10	9/9	NA	13/13	17/18	8/8	15/15	X	13/13	19/22
	T	10/12	29/30	NA	NA	16/17/19	7/9	9/10	11/11	NA	13/15.2	17/18	8/11	15/15	X	13/13	19/22
GC25	N	12/13	29/30	8/11	10/12	15/16	9/9	10/11	9/9	19/21	13/14	14/16	8/11	13/14	X	11/12	20/25.2
	T	10/12/13	29/29.2/30	8/11	12/12	15/16/17	7/9	9/10/11	9/10	19/21/24	11.2/13/14/15.2	14/16/18	8/11	13/14/16	XY	11/12	20/25.2/26
GC27	N	10/16	30/34.2	11/12	12/12	15/16	9/9	8/9	9/11	19/23	13/15.2	15/17	11/11	14/16	X	12/12	21/23
	T	10/11/16	30/31.2/34.2	11/12	11/12	15/16	9/9	8/9	9/11/12	18/19/23	13/15.2	15/17/18	8/11	13/14/16	X	9/12	21/23
GC28	N	13/14	30/31	NA	NA	16/18	7/7	9/12	9/9	NA	13/14	14/16	NA	NA	X	11/12	21/23
	T	13/14/15	30/31	8/10/11	7/10/12	16/18	7/9	9/12	9/9	23/24	13/14/15.2	14/16/18	8/11	13/15	XY	11/12	20/21/23
GC29	N	10/16	29/30	12/12	10/10	15/16	9/9	12/13	9/13	19/24	13/15.2	14/16	8/8	15/16	X	10/12	22/23
	T	10/13/16	29/29	10/12	10/13	14/15/16	7/9	10/12/13	12/12	18/19/24	13/14/15.2	14/16/20	8/11	15/16/20	X	10/12	22/23/26

N, Normal; T, Tumor; HM, hydatidiform mole; IM, invasive mole

Reds indicate informative allele

For the five cases (GC02, 03, 06, 22, and 26), STR was not analyzed because DNA could not be amplified from the tissues.

Supplementary Table S3. Copy number alterations identified across 29 gestational choriocarcinomas

Sample_ID	Position	Event	Length	Cytoband	Cancer-related Genes
GC01	chr1:190,441,508-228,782,886	Gain	38,341,379	q31.1 - q42.13	MDM4, ELK4, SLC45A3, H3F3A
GC01	chr3:93,517,443-198,022,430	Gain	104,504,988	q11.1 - q29	TFG, CBLB, GATA2, RPN1, FOXL2, WWTR1, GMPS, MLF1, EVI1, PIK3CA, SOX2, ETV5, EIF4A2, BCL6, LPP, TFRC
GC01	chr4:0-42,369,641	Loss	42,369,642	p16.3 - p13	FGFR3, WHSC1, SLC34A2, PHOX2B
GC01	chr4:43,034,384-49,092,454	Gain	6,058,071	p13 - p11	
GC01	chr5:153,489,326-170,359,661	Loss	16,870,336	q33.2 - q35.1	ITK, EBF1, RANBP17
GC01	chr5:170,359,661-180,915,260	Gain	10,555,600	q35.1 - q35.3	RANBP17, TLX3, NPM1, NSD1
GC01	chr7:67,742,846-88,630,361	Gain	20,887,516	q11.22 - q21.13	ELN, HIP1
GC01	chr8:0-33,108,738	Loss	33,108,739	p23.3 - p12	PCM1, WRN
GC01	chr8:33,108,738-37,643,563	Gain	4,534,826	p12 - p11.23	
GC01	chr8:42,328,029-43,776,756	Gain	1,448,728	p11.21 - p11.1	HOOK3
GC01	chr8:46,896,972-146,364,022	Gain	99,467,051	q11.1 - q24.3	TCEA1, PLAG1, CHCHD7, NCOA2, HEY1, COX6C, EXT1, MYC, NDRG1, RECQL4
GC01	chr10:0-39,146,676	Gain	39,146,677	p15.3 - p11.1	GATA3, MLLT10, KIF5B
GC01	chr11:0-46,309,144	Loss	46,309,145	p15.5 - p11.2	HRAS, CARS, NUP98, LMO1, FANCF, WT1, LMO2, EXT2, CREB3L1
GC01	chr11:54,795,357-69,480,387	Loss	14,685,031	q11 - q13.3	MEN1, CCND1
GC01	chr11:76,084,081-80,265,067	Loss	4,180,987	q13.5 - q14.1	
GC01	chr12:1-35,800,000	Gain	35,800,000	p13.33 - p11	KDM5A, CCND2, ZNF384, ETV6, KRAS
GC01	chr13:19,084,823-115,169,878	Loss	96,085,056	q11 - q34	CDX2, FLT3, BRCA2, LHFP, LCP1, RB1, ERCC5
GC01	chr19:0-24,544,320	Loss	24,544,321	p13.3 - p11	FSTL3, STK11, TCF3, GNA11, SH3GL1, MLLT1, DNM2, SMARCA4, LYL1, BRD4, TPM4, JAK3, ELL
GC01	chr22:16,054,713-23,793,102	Gain	7,738,390	q11.1 - q11.23	CLTCL1, BCR
GC01	chr22:23,793,102-51,304,566	Loss	27,511,465	q11.23 - q13.33	SMARCB1, MN1, CHEK2, EWSR1, NF2, MYH9, PDGFB, MKL1, EP300
GC01	chrX:1,796,873-57,407,161	Loss	55,610,289	p22.33 - p11.21	ZRSR2, BCOR, KDM6A, SSX1, SSX4, WAS, GATA1, TFE3, SSX2, KDM5C
GC01	chrX:63,414,952-155,270,560	Loss	91,855,609	q11.2 - q28	MSN, MED12, NONO, ATRX, SEPT6, ELF4, GPC3, PHF6, MTCP1
GC02	chr12:0-35,800,000	Gain	35,800,001	p13.33 - p11	KDM5A, CCND2, ZNF384, ETV6, KRAS
GC02	chr12:37,902,988-133,851,895	Gain	95,948,908	q11 - q24.33	ARID2, MLL2, ATF1, HOXC13, HOXC11, NACA, DDIT3, CDK4, LRIG3, WIF1, HMGA2, MDM2, BTG1, ALDH2, PTPN11, BCL7A
GC03	chr1:144,009,053-249,250,621	Gain	105,241,569	q21.1 - q44	PDE4DIP, BCL9, ARNT, TPM3, MUC1, PRCC, NTRK1, SDHC, FCGR2B, PBX1, ABL2, TPR, MDM4, ELK4, SLC45A3, H3F3A, FH
GC03	chr3:93,517,443-196,561,903	Gain	103,044,461	q11.1 - q29	TFG, CBLB, GATA2, RPN1, FOXL2, WWTR1, GMPS, MLF1, EVI1, PIK3CA, SOX2, ETV5, EIF4A2, BCL6, LPP, TFRC
GC03	chr14:20,034,753-107,349,540	Gain	87,314,788	q11.2 - q32.33	CCNB1IP1, TRA@, NKX2-1, NIN, KTN1, GPHN, TSHR, TRIP11, GOLGA5, DICER1, TCL6, TCL1A, BCL11B, AKT1, IGH@
GC04	chr12:0-35,800,000	Gain	35,800,001	p13.33 - p11	KDM5A, CCND2, ZNF384, ETV6, KRAS
GC04	chr14:20,219,083-106,534,342	Gain	86,315,260	q11.2 - q32.33	CCNB1IP1, TRA@, NKX2-1, NIN, KTN1, GPHN, TSHR, TRIP11, GOLGA5, DICER1, TCL6, TCL1A, BCL11B, AKT1, IGH@

Sample_ID	Position	Event	Length	Cytoband	Cancer-related Genes
GC04	chr16:14,908,682-18,530,801	Gain	3,622,120	p13.11 - p12.3	MYH11
GC04	chr16:87,129,132-90,071,315	Gain	2,942,184	q24.2 - q24.3	CBFA2T3, FANCA
GC05	chr1:162,457,567-233,286,777	Gain	70,829,211	q23.3 - q42.2	PBX1, ABL2, TPR, MDM4, ELK4, SLC45A3, H3F3A
GC05	chr5:147,158,012-175,763,320	Gain	28,605,309	q32 - q35.2	PDGFRB, CD74, ITK, EBF1, RANBP17, TLX3, NPM1
GC05	chr14:21,170,186-106,778,612	Gain	85,608,427	q11.2 - q32.33	TRA@, NKX2-1, NIN, KTN1, GPHN, TSHR, TRIP11, GOLGA5, DICER1, TCL6, TCL1A, BCL11B, AKT1, IGH@
GC06	chr1:4,686,477-30,646,011	Loss	25,959,535	p36.32 - p35.2	RPL22, CAMTA1, SDHB, PAX7, MDS2, ARID1A
GC06	chr3:279,427-45,351,600	Gain	45,072,174	p26.3 - p21.31	SRGAP3, FANCD2, VHL, PPARG, RAF1, XPC, MLH1, MYD88, CTNNB1
GC06	chr5:163,741,452-180,915,260	Gain	17,173,809	q34 - q35.3	RANBP17, TLX3, NPM1, NSD1
GC06	chr7:99,939,975-159,138,663	Loss	59,198,689	q22.1 - q36.3	MET, SMO, CREB3L2, KIAA1549, BRAF, EZH2, MLL3
GC06	chr8:103,816,189-146,364,022	Loss	42,547,834	q22.3 - q24.3	EXT1, MYC, NDRG1, RECQL4
GC06	chr15:40,891,212-48,953,446	Loss	8,062,235	q15.1 - q21.1	
GC06	chr18:46,534,567-78,077,248	Loss	31,542,682	q21.1 - q23	MALT1, BCL2
GC07	chr1:144,009,053-245,480,493	Gain	101,471,441	q21.1 - q44	PDE4DIP, BCL9, ARNT, TPM3, MUC1, PRCC, NTRK1, SDHC, FCGR2B, PBX1, ABL2, TPR, MDM4, ELK4, SLC45A3, H3F3A, FH
GC07	chr6:65,085,738-91,164,955	Loss	26,079,218	q12 - q15	
GC07	chr11:55,480,738-135,006,516	Gain	79,525,779	q11 - q25	MEN1, CCND1, NUMA1, PICALM, MAML2, BIRC3, ATM, DDX10, POU2AF1, SDHD, PAFAH1B2, PCSK7, MLL, DDX6, CBL, ARHGEF12, FLI1
GC07	chr12:1-35,800,000	Gain	35,800,000	p13.33 - p11	KDM5A, CCND2, ZNF384, ETV6, KRAS
GC07	chr17:76,187,310-79,969,402	Gain	3,782,093	q25.3	CANT1, ASPSCR1
GC07	chr22:24,024,141-44,864,360	Loss	20,840,220	q11.23 - q13.31	SMARCB1, MN1, CHEK2, EWSR1, NF2, MYH9, PDGFB, MKL1, EP300
GC08	chr1:0-24,821,659	Loss	24,821,660	p36.33 - p36.11	TNFRSF14, PRDM16, RPL22, CAMTA1, SDHB, PAX7, MDS2
GC08	chr1:190,845,791-248,864,690	Gain	58,018,900	q31.2 - q44	MDM4, ELK4, SLC45A3, H3F3A, FH
GC08	chr4:0-38,580,974	Loss	38,580,975	p16.3 - p14	FGFR3, WHSC1, SLC34A2
GC08	chr7:0-58,025,423	Gain	58,025,424	p22.3 - p11.1	CARD11, PMS2, ETV1, HNRNPA2B1, HOXA9, HOXA11, HOXA13, JAZF1, IKZF1, EGFR
GC08	chr7:61,064,518-159,138,663	Gain	98,074,146	q11.1 - q36.3	SBDS, ELN, HIP1, AKAP9, CDK6, MET, SMO, CREB3L2, KIAA1549, BRAF, EZH2, MLL3
GC08	chr9:6,623,327-39,184,065	Gain	32,560,739	p24.1 - p13.1	NFIB, MLLT3, FANCG, PAX5
GC08	chr14:20,460,487-106,534,342	Gain	86,073,856	q11.2 - q32.33	CCNB1IP1, TRA@, NKX2-1, NIN, KTN1, GPHN, TSHR, TRIP11, GOLGA5, DICER1, TCL6, TCL1A, BCL11B, AKT1, IGH@
GC08	chr19:0-24,544,320	Loss	24,544,321	p13.3 - p11	FSTL3, STK11, TCF3, GNA11, SH3GL1, MLLT1, DNM2, SMARCA4, LYL1, BRD4, TPM4, JAK3, ELL
GC08	chr19:27,754,573-54,207,333	Loss	26,452,761	q11 - q13.42	CCNE1, CEBPA, AKT2, CD79A, CIC, BCL3, CBLC, ERCC2, KLK2, PPP2R1A, ZNF331
GC08	chr19:54,207,333-56,157,458	Gain	1,950,126	q13.42	TFPT
GC08	chr20:0-6,286,597	Gain	6,286,598	p13 - p12.3	
GC08	chr20:29,519,156-63,025,520	Gain	33,506,365	q11.21 - q13.33	ASXL1, MAFB, TOP1, SDC4, GNAS, SS18L1
GC08	chrX:0-58,470,802	Loss	58,470,803	p22.33 - p11.1	CRLF2, P2RY8, ZRSR2, BCOR, KDM6A, SSX1, SSX4, WAS, GATA1, TFE3, SSX2, KDM5C
GC08	chrX:61,732,394-155,270,560	Loss	93,538,167	q11.1 - q28	MSN, MED12, NONO, ATRX, SEPT6, ELF4, GPC3, PHF6, MTCP1

Sample_ID	Position	Event	Length	Cytoband	Cancer-related Genes
GC09	chr3:10,387,620-47,624,641	Gain	37,237,022	p25.3 - p21.31	PPARG, RAF1, XPC, MLH1, MYD88, CTNNB1, SETD2
GC09	chr14:20,219,083-106,534,342	Gain	86,315,260	q11.2 - q32.33	CCNB1IP1, TRA@, NKX2-1, NIN, KTN1, GPHN, TSHR, TRIP11, GOLGA5, DICER1, TCL6, TCL1A, BCL11B, AKT1, IGH@
GC12	chrX:0-58,470,802	Loss	58,470,803	p22.33 - p11.1	CRLF2, P2RY8, ZRSR2, BCOR, KDM6A, SSX1, SSX4, WAS, GATA1, TFE3, SSX2, KDM5C
GC12	chrX:61,732,394-155,270,560	Loss	93,538,167	q11.1 - q28	MSN, MED12, NONO, ATRX, SEPT6, ELF4, GPC3, PHF6, MTCP1
GC13	chr1:144,009,053-204,583,550	Gain	60,574,498	q21.1 - q32.1	PDE4DIP, BCL9, ARNT, TPM3, MUC1, PRCC, NTRK1, SDHC, FCGR2B, PBX1, ABL2, TPR, MDM4
GC13	chr1:235,124,954-249,250,621	Gain	14,125,668	q42.3 - q44	FH
GC13	chr5:135,621,515-171,386,090	Gain	35,764,576	q31.1 - q35.1	PDGFRB, CD74, ITK, EBF1, RANBP17, TLX3, NPM1
GC13	chr5:171,386,091-180,915,260	Gain	9,529,170	q35.1 - q35.3	NSD1
GC13	chr6:64,755,126-119,662,161	Loss	54,907,036	q12 - q22.31	PRDM1, ROS1, GOPC
GC13	chr8:0-43,767,534	Loss	43,767,535	p23.3 - p11.1	PCM1, WRN, WHSC1L1, FGFR1, HOOK3
GC13	chr8:46,896,972-146,364,022	Gain	99,467,051	q11.1 - q24.3	TCEA1, PLAG1, CHCHD7, NCOA2, HEY1, COX6C, EXT1, MYC, NDRG1, RECQL4
GC13	chr9:0-39,184,065	Gain	39,184,066	p24.3 - p13.1	JAK2, CD274, NFIB, MLLT3, FANCG, PAX5
GC13	chr9:70,984,372-140,777,171	Gain	69,792,800	q21.11 - q34.3	GNAQ, SYK, OMD, FANCC, XPA, NR4A3, TAL2, SET, FNBP1, ABL1, NUP214, TSC1, RALGDS, BRD3, NOTCH1
GC13	chr10:2,463,645-5,193,680	Loss	2,730,036	p15.3 - p15.1	
GC13	chr16:12,141,686-31,952,510	Gain	19,810,825	p13.13 - p11.2	ERCC4, MYH11, PALB2, IL21R, FUS
GC13	chrX:0-58,470,802	Loss	58,470,803	p22.33 - p11.1	CRLF2, P2RY8, ZRSR2, BCOR, KDM6A, SSX1, SSX4, WAS, GATA1, TFE3, SSX2, KDM5C
GC13	chrX:61,732,394-155,270,560	Loss	93,538,167	q11.1 - q28	MSN, MED12, NONO, ATRX, SEPT6, ELF4, GPC3, PHF6, MTCP1
GC14	chr2:1-84,233,458	Gain	84,233,458	p25.3 - p11.2	MYCN, C2orf44, NCOA1, DNMT3A, ALK, EML4, MSH2, MSH6, FBXO11, BCL11A, REL, XPO1
GC14	chr3:155,313,592-194,366,678	Gain	39,053,087	q25.31 - q29	GMPS, MLF1, EVI1, PIK3CA, SOX2, ETV5, EIF4A2, BCL6, LPP
GC14	chr5:97,232,938-124,653,590	Loss	27,420,653	q15 - q23.2	APC
GC14	chr9:71,258,907-141,213,431	Gain	69,954,525	q21.11 - q34.3	GNAQ, SYK, OMD, FANCC, XPA, NR4A3, TAL2, SET, FNBP1, ABL1, NUP214, TSC1, RALGDS, BRD3, NOTCH1
GC14	chr18:27,074,172-78,077,248	Loss	51,003,077	q12.1 - q23	MALT1, BCL2
GC15	chr1:144,009,053-249,250,621	Gain	105,241,569	q21.1 - q44	PDE4DIP, BCL9, ARNT, TPM3, MUC1, PRCC, NTRK1, SDHC, FCGR2B, PBX1, ABL2, TPR, MDM4, ELK4, SLC45A3, H3F3A, FH
GC15	chr3:3,136,534-91,000,000	Gain	87,863,467	p26.2 - q11.1	SRGAP3, FANCD2, VHL, PPARG, RAF1, XPC, MLH1, MYD88, CTNNB1, SETD2, BAP1, PBRM1, FHIT, MITF, FOXP1
GC15	chr3:93,847,479-198,022,430	Gain	104,174,952	q11.1 - q29	TFG, CBLB, GATA2, RPN1, FOXL2, WWTR1, GMPS, MLF1, EVI1, PIK3CA, SOX2, ETV5, EIF4A2, BCL6, LPP, TFRC
GC15	chr12:0-35,800,000	Gain	35,800,001	p13.33 - p11	KDM5A, CCND2, ZNF384, ETV6, KRAS
GC15	chr12:37,902,988-133,851,895	Gain	95,948,908	q11 - q24.33	ARID2, MLL2, ATF1, HOXC13, HOXC11, NACA, DDIT3, CDK4, LRIG3, WIF1, HMGA2, MDM2, BTG1, ALDH2, PTPN11, BCL7A
GC15	chr14:21,171,763-106,393,378	Gain	85,221,616	q11.2 - q32.33	TRA@, NKX2-1, NIN, KTN1, GPHN, TSHR, TRIP11, GOLGA5, DICER1, TCL6, TCL1A, BCL11B, AKT1, IGH@
GC16	chr1:144,009,053-249,250,621	Gain	105,241,569	q21.1 - q44	PDE4DIP, BCL9, ARNT, TPM3, MUC1, PRCC, NTRK1, SDHC, FCGR2B, PBX1, ABL2, TPR, MDM4, ELK4, SLC45A3, H3F3A, FH

Sample_ID	Position	Event	Length	Cytoband	Cancer-related Genes
GC16	chr3:2,043,558-89,472,762	Gain	87,429,205	p26.3 - p11.1	SRGAP3, FANCD2, VHL, PPARG, RAF1, XPC, MLH1, MYD88, CTNNB1, SETD2, BAP1, PBRM1, FHIT, MITF, FOXP1
GC16	chr3:93,517,443-198,022,430	Gain	104,504,988	q11.1 - q29	TFG, CBLB, GATA2, RPN1, FOXL2, WWTR1, GMPS, MLF1, EVI1, PIK3CA, SOX2, ETV5, EIF4A2, BCL6, LPP, TFRC
GC16	chr4:56,919,886-62,663,226	Loss	5,743,341	q12 - q13.1	
GC16	chr4:69,529,506-71,108,120	Loss	1,578,615	q13.2 - q13.3	
GC16	chr4:94,557,803-98,406,108	Loss	3,848,306	q22.2 - q22.3	
GC16	chr4:113,874,485-115,324,738	Loss	1,450,254	q25 - q26	
GC16	chr4:116,253,141-123,218,150	Loss	6,965,010	q26 - q27	
GC16	chr4:149,514,023-151,581,380	Loss	2,067,358	q31.23 - q31.3	
GC16	chr12:862,034-34,524,481	Gain	33,662,448	p13.33 - p11.1	CCND2, ZNF384, ETV6, KRAS
GC16	chr12:38,426,187-133,851,895	Gain	95,425,709	q12 - q24.33	ARID2, MLL2, ATF1, HOXC13, HOXC11, NACA, DDIT3, CDK4, LRIG3, WIF1, HMGA2, MDM2, BTG1, ALDH2, PTPN11, BCL7A
GC16	chr14:20,219,083-107,349,540	Gain	87,130,458	q11.2 - q32.33	CCNB1IP1, TRA@, NKX2-1, NIN, KTN1, GPHN, TSHR, TRIP11, GOLGA5, DICER1, TCL6, TCL1A, BCL11B, AKT1, IGH@
GC16	chr16:52,763,033-59,859,416	Gain	7,096,384	q12.2 - q21	HERPUD1
GC16	chr16:60,643,493-63,659,740	Loss	3,016,248	q21	
GC16	chr16:65,266,889-75,278,871	Gain	10,011,983	q21 - q23.1	CBFB, CDH1
GC16	chr16:76,088,021-83,062,850	Loss	6,974,830	q23.1 - q23.3	MAF
GC16	chr22:16,054,713-51,304,566	Gain	35,249,854	q11.1 - q13.33	CLTCL1, BCR, SMARCB1, MN1, CHEK2, EWSR1, NF2, MYH9, PDGFB, MKL1, EP300
GC16	chrX:1-58,641,718	Loss	58,641,718	p22.33 - p11.1	CRLF2, P2RY8, ZRSR2, BCOR, KDM6A, SSX1, SSX4, WAS, GATA1, TFE3, SSX2, KDM5C
GC16	chrX:61,742,360-155,270,560	Loss	93,528,201	q11.1 - q28	MSN, MED12, NONO, ATRX, SEPT6, ELF4, GPC3, PHF6, MTCP1
GC17	chr5:0-12,354,270	Loss	12,354,271	p15.33 - p15.2	
GC17	chr5:104,442,367-180,915,260	Gain	76,472,894	q21.2 - q35.3	APC, PDGFRB, CD74, ITK, EBF1, RANBP17, TLX3, NPM1, NSD1
GC18	chr3:93,517,443-198,022,430	Gain	104,504,988	q11.1 - q29	TFG, CBLB, GATA2, RPN1, FOXL2, WWTR1, GMPS, MLF1, EVI1, PIK3CA, SOX2, ETV5, EIF4A2, BCL6, LPP, TFRC
GC18	chr5:1-32,958,618	Loss	32,958,618	p15.33 - p13.3	
GC18	chr5:56,592,464-57,754,808	Loss	1,162,345	q11.2	
GC18	chr5:165,173,423-180,698,312	Gain	15,524,890	q34 - q35.3	RANBP17, TLX3, NPM1, NSD1
GC18	chr7:1-58,025,423	Gain	58,025,423	p22.3 - p11.1	CARD11, PMS2, ETV1, HNRNPA2B1, HOXA9, HOXA11, HOXA13, JAZF1, IKZF1, EGFR
GC18	chr8:14,999,817-43,752,358	Loss	28,752,542	p22 - p11.1	PCM1, WRN, WHSC1L1, FGFR1, HOOK3
GC18	chr9:70,984,372-141,213,431	Gain	70,229,060	q21.11 - q34.3	GNAQ, SYK, OMD, FANCC, XPA, NR4A3, TAL2, SET, FNBP1, ABL1, NUP214, TSC1, RALGDS, BRD3, NOTCH1
GC18	chr21:14,344,537-48,129,895	Gain	33,785,359	q11.2 - q22.3	OLIG2, RUNX1, ERG, TMPRSS2, U2AF1
GC18	chr22:16,054,713-51,304,566	Gain	35,249,854	q11.1 - q13.33	CLTCL1, BCR, SMARCB1, MN1, CHEK2, EWSR1, NF2, MYH9, PDGFB, MKL1, EP300
GC19	chr3:1-1,691,090	Loss	1,691,090	p26.3	
GC19	chr3:1,696,212-39,129,742	Gain	37,433,531	p26.3 - p22.2	SRGAP3, FANCD2, VHL, PPARG, RAF1, XPC, MLH1, MYD88

Sample_ID	Position	Event	Length	Cytoband	Cancer-related Genes
GC19	chr3:40,342,844-42,894,677	Gain	2,551,834	p22.1	CTNNB1
GC21	chr1:144,009,053-249,250,621	Gain	105,241,569	q21.1 - q44	PDE4DIP, BCL9, ARNT, TPM3, MUC1, PRCC, NTRK1, SDHC, FCGR2B, PBX1, ABL2, TPR, MDM4, ELK4, SLC45A3, H3F3A, FH
GC21	chr14:20,219,083-107,349,540	Gain	87,130,458	q11.2 - q32.33	CCNB1IP1, TRA@, NKX2-1, NIN, KTN1, GPHN, TSHR, TRIP11, GOLGA5, DICER1, TCL6, TCL1A, BCL11B, AKT1, IGH@
GC21	chr21:14,344,537-48,129,895	Gain	33,785,359	q11.2 - q22.3	OLIG2, RUNX1, ERG, TMPRSS2, U2AF1
GC22	chr1:144,009,053-249,250,621	Gain	105,241,569	q21.1 - q44	PDE4DIP, BCL9, ARNT, TPM3, MUC1, PRCC, NTRK1, SDHC, FCGR2B, PBX1, ABL2, TPR, MDM4, ELK4, SLC45A3, H3F3A, FH
GC22	chr7:1-58,025,423	Gain	58,025,423	p22.3 - p11.1	CARD11, PMS2, ETV1, HNRNPA2B1, HOXA9, HOXA11, HOXA13, JAZF1, IKZF1, EGFR
GC22	chr7:61,064,518-159,138,663	Gain	98,074,146	q11.1 - q36.3	SBDS, ELN, HIP1, AKAP9, CDK6, MET, SMO, CREB3L2, KIAA1549, BRAF, EZH2, MLL3
GC22	chr14:20,219,083-107,349,540	Gain	87,130,458	q11.2 - q32.33	CCNB1IP1, TRA@, NKX2-1, NIN, KTN1, GPHN, TSHR, TRIP11, GOLGA5, DICER1, TCL6, TCL1A, BCL11B, AKT1, IGH@
GC22	chrX:1-58,641,718	Loss	58,641,718	p22.33 - p11.1	CRLF2, P2RY8, ZRSR2, BCOR, KDM6A, SSX1, SSX4, WAS, GATA1, TFE3, SSX2, KDM5C
GC22	chrX:61,742,360-155,270,560	Loss	93,528,201	q11.1 - q28	MSN, MED12, NONO, ATRX, SEPT6, ELF4, GPC3, PHF6, MTCP1
GC23	chr1:1-93,439,821	Loss	93,439,821	p36.33 - p22.1	TNFRSF14, PRDM16, RPL22, CAMTA1, SDHB, PAX7, MDS2, ARID1A, LCK, SFPQ, THRAP3, MYCL1, MPL, MUTYH, TAL1, CDKN2C, EPS15, JUN, JAK1, FUBP1, BCL10
GC23	chr1:144,009,053-249,250,621	Gain	105,241,569	q21.1 - q44	PDE4DIP, BCL9, ARNT, TPM3, MUC1, PRCC, NTRK1, SDHC, FCGR2B, PBX1, ABL2, TPR, MDM4, ELK4, SLC45A3, H3F3A, FH
GC23	chr4:6,736,751-47,055,106	Loss	40,318,356	p16.1 - p12	SLC34A2, PHOX2B
GC23	chr8:1-43,767,534	Loss	43,767,534	p23.3 - p11.1	PCM1, WRN, WHSC1L1, FGFR1, HOOK3
GC23	chr8:46,896,972-146,364,022	Loss	99,467,051	q11.1 - q24.3	TCEA1, PLAG1, CHCHD7, NCOA2, HEY1, COX6C, EXT1, MYC, NDRG1, RECQL4
GC23	chr10:1-39,146,676	Loss	39,146,676	p15.3 - p11.1	GATA3, MLLT10, KIF5B
GC23	chr10:42,413,322-135,534,747	Loss	93,121,426	q11.21 - q26.3	RET, NCOA4, CCDC6, PRF1, BMPR1A, FAM22A, PTEN, TLX1, NFKB2, SUFU, VTI1A, TCF7L2, FGFR2, DUX4
GC23	chr11:192,764-13,090,955	Loss	12,898,192	p15.5 - p15.2	HRAS, CARS, NUP98, LMO1
GC23	chr11:63,267,937-134,938,847	Loss	71,670,911	q12.3 - q25	MEN1, CCND1, NUMA1, PICALM, MAML2, BIRC3, ATM, DDX10, POU2AF1, SDHD, PAFAH1B2, PCSK7, MLL, DDX6, CBL, ARHGEF12, FLI1
GC23	chr12:1-35,800,000	Loss	35,800,000	p13.33 - q11	KDM5A, CCND2, ZNF384, ETV6, KRAS
GC23	chr12:37,902,988-133,851,895	Loss	95,948,908	q11 - q24.33	ARID2, MLL2, ATF1, HOXC13, HOXC11, NACA, DDIT3, CDK4, LRIG3, WIF1, HMGA2, MDM2, BTG1, ALDH2, PTPN11, BCL7A
GC23	chr13:19,084,823-115,169,878	Loss	96,085,056	q11 - q34	CDX2, FLT3, BRCA2, LHFP, LCP1, RB1, ERCC5
GC23	chr14:20,219,083-107,349,540	Loss	87,130,458	q11.2 - q32.33	CCNB1IP1, TRA@, NKX2-1, NIN, KTN1, GPHN, TSHR, TRIP11, GOLGA5, DICER1, TCL6, TCL1A, BCL11B, AKT1, IGH@
GC23	chr16:1-29,146,666	Loss	29,146,666	p13.3 - p11.2	TSC2, CREBBP, CIITA, SOCS1, TNFRSF17, ERCC4, MYH11, PALB2, IL21R
GC23	chr16:29,146,668-35,271,725	Loss	6,125,058	p11.2 - p11.1	FUS
GC23	chr16:46,461,309-90,354,753	Loss	43,893,445	q11.2 - q24.3	CYLD, HERPUD1, CDH11, CBFB, CDH1, MAF, CBFA2T3, FANCA
GC24	chr1:94,455,420-121,350,934	Gain	94,455,419	p22.1 - p11.2	RBM15, TRIM33, NRAS, FAM46C, NOTCH2
GC24	chr1:177,425,070-191,362,050	Gain	13,936,981	q25.2 - q31.2	ABL2, TPR
GC24	chr1:191,377,664-204,549,564	Gain	13,171,901	q31.2 - q32.1	MDM4

Sample_ID	Position	Event	Length	Cytoband	Cancer-related Genes
GC24	chr1:204,551,407-213,164,918	Gain	8,613,512	q32.1 - q32.3	MDM4, ELK4, SLC45A3
GC24	chr3:2,431,546-23,590,068	Gain	21,158,523	p26.3 - p24.3	SRGAP3, FANCD2, VHL, PPARG, RAF1, XPC
GC24	chr3:93,517,443-198,022,430	Gain	104,504,988	q11.1 - q29	TFG, CBLB, GATA2, RPN1, FOXL2, WWTR1, GMPS, MLF1, EVI1, PIK3CA, SOX2, ETV5, EIF4A2, BCL6, LPP, TFRC
GC24	chr5:139,517,197-180,915,260	Gain	41,398,064	q31.3 - q35.3	PDGFRB, CD74, ITK, EBF1, RANBP17, TLX3, NPM1, NSD1
GC24	chr7:1-58,025,423	Gain	58,025,423	p22.3 - p11.1	CARD11, PMS2, ETV1, HNRNPA2B1, HOXA9, HOXA11, HOXA13, JAZF1, IKZF1, EGFR
GC24	chr8:20,418,893-41,531,478	Loss	21,112,586	p21.3 - p11.21	WRN, WHSC1L1, FGFR1
GC24	chr8:98,381,596-146,364,022	Gain	47,982,427	q22.1 - q24.3	COX6C, EXT1, MYC, NDRG1, RECQL4
GC24	chr9:1-12,288,920	Gain	12,288,920	p24.3 - p23	JAK2, CD274
GC24	chr10:1-39,146,676	Gain	39,146,676	p15.3 - p11.1	GATA3, MLLT10, KIF5B
GC24	chr11:54,795,357-135,006,516	Gain	80,211,160	q11 - q25	MEN1, CCND1, NUMA1, PICALM, MAML2, BIRC3, ATM, DDX10, POU2AF1, SDHD, PAFAH1B2, PCSK7, MLL, DDX6, CBL, ARHGEF12, FLI1
GC24	chr17:25,326,941-81,195,210	Gain	55,868,270	q11.1 - q25.3	NF1, SUZ12, TAF15, MLLT6, LASP1, CDK12, ERBB2, RARA, BRCA1, ETV4, COL1A1, HLF, MSI2, CLTC, BRIP1, CD79B, DDX5, PRKAR1A, SRSF2, CANT1, ASPSCR1
GC24	chr20:29,519,156-63,025,520	Gain	33,506,365	q11.21 - q13.33	ASXL1, MAFB, TOP1, SDC4, GNAS, SS18L1
GC24	chr21:30,588,803-44,056,281	Gain	13,467,479	q21.3 - q22.3	OLIG2, RUNX1, ERG, TMPRSS2
GC24	chrX:49,371,123-57,543,583	Loss	8,172,461	p11.23 - p11.21	SSX2, KDM5C
GC24	chrX:61,732,394-102,914,338	Gain	41,181,945	q11.1 - q22.2	MSN, MED12, NONO, ATRX
GC25	chr19:247,232-15,363,052	Loss	15,115,821	p13.3 - p13.12	FSTL3, STK11, TCF3, GNA11, SH3GL1, MLLT1, DNM2, SMARCA4, LYL1, BRD4
GC25	chrX:2,686,900-58,470,802	Loss	55,783,903	p22.33 - p11.1	ZRSR2, BCOR, KDM6A, SSX1, SSX4, WAS, GATA1, TFE3, SSX2, KDM5C
GC25	chrX:61,732,394-155,270,560	Loss	93,538,167	q11.1 - q28	MSN, MED12, NONO, ATRX, SEPT6, ELF4, GPC3, PHF6, MTCP1
GC26	chr14:20,219,083-107,349,540	Gain	87,130,458	q11.2 - q32.33	CCNB1IP1, TRA@, NKX2-1, NIN, KTN1, GPHN, TSHR, TRIP11, GOLGA5, DICER1, TCL6, TCL1A, BCL11B, AKT1, IGH@
GC26	chrX:2,726,248-58,470,802	Loss	55,744,555	p22.33 - p11.1	ZRSR2, BCOR, KDM6A, SSX1, SSX4, WAS, GATA1, TFE3, SSX2, KDM5C
GC26	chrX:61,732,394-155,270,560	Loss	93,538,167	q11.1 - q28	MSN, MED12, NONO, ATRX, SEPT6, ELF4, GPC3, PHF6, MTCP1
GC28	chrX:1-58,470,802	Loss	58,470,802	p22.33 - p11.1	CRLF2, P2RY8, ZRSR2, BCOR, KDM6A, SSX1, SSX4, WAS, GATA1, TFE3, SSX2, KDM5C
GC28	chrX:61,732,394-155,270,560	Loss	93,538,167	q11.1 - q28	MSN, MED12, NONO, ATRX, SEPT6, ELF4, GPC3, PHF6, MTCP1
GC29	chr1:145,677,085-147,032,779	Gain	1,355,695	q21.1 - q21.2	BCL9
GC29	chr1:149,742,045-154,793,762	Gain	5,051,718	q21.2 - q21.3	ARNT, TPM3
GC29	chr1:154,793,763-202,410,428	Gain	47,616,666	q21.3 - q32.1	MUC1, PRCC, NTRK1, SDHC, FCGR2B, PBX1, ABL2, TPR
GC29	chr1:202,410,429-210,008,673	Gain	7,598,245	q32.1 - q32.2	MDM4, ELK4, SLC45A3
GC29	chr2:1-79,948,921	Gain	79,948,921	p25.3 - p12	MYCN, C2orf44, NCOA1, DNMT3A, ALK, EML4, MSH2, MSH6, FBXO11, BCL11A, REL, XPO1
GC29	chr3:1-4,451,994	Loss	4,451,994	p26.3 - p26.1	
GC29	chr3:4,451,995-27,721,197	Gain	23,269,203	p26.1 - p24.1	SRGAP3, FANCD2, VHL, PPARG, RAF1, XPC
GC29	chr3:27,721,198-43,715,466	Gain	15,994,269	p24.1 - p21.33	MLH1, MYD88, CTNNB1
GC29	chr5:39,637,565-45,365,071	Gain	5,727,507	p13.1 - p12	

Sample_ID	Position	Event	Length	Cytoband	Cancer-related Genes
GC29	chr16:46,461,309-90,354,753	Gain	43,893,445	q11.2 - q24.3	CYLD, HERPUD1, CDH11, CBFB, CDH1, MAF, CBFA2T3, FANCA
GC29	chrX:108,593,683-112,259,924	Gain	3,666,242	q22.3 - q23	
GC29	chrX:112,259,925-155,270,560	Loss	43,010,636	q23 - q28	SEPT6, ELF4, GPC3, PHF6, MTCP1

Supplementary Table S4. Recurrent regions of copy number alterations

Region	Event	Cytoband	Cancer-related Genes	Frequency	Cases
chr1:144009053-249250621	Gain	q21.1-q44	PDE4DIP, BCL9, ARNT, TPM3, MUC1, PRCC, NTRK1, SDHC, FCGR2B, PBX1, ABL2, TPR, MDM4, ELK4, SLC45A3, H3F3A, FH	13 (44.8%)	GC01, GC03, GC05, GC07, GC08, GC13, GC15, GC16, GC21, GC22, GC23, GC24, GC29
chr3:4451995-39129742	Gain	p26.1 - p22.2	SRGAP3, FANCD2, VHL, PPARG, RAF1, XPC, MLH1, MYD88	7 (24.1%)	GC06, GC09, GC15, GC16, GC19, GC24, GC29
chr3:93847479-196561903	Gain	q11.1-q29	TFG, CBLB, GATA2, RPN1, FOXL2, WWTR1, GMPS, MLF1, EVI1, PIK3CA, SOX2, ETV5, EIF4A2, BCL6, LPP, TFRC	7 (24.1%)	GC01, GC03, GC14, GC15, GC16, GC18, GC24
chr5:165173423-180698312	Gain	q34 - q35.3	RANBP17, TLX3, NPM1, NSD1	7 (24.1%)	GC01, GC05, GC06, GC13, GC17, GC18, GC24
chr12:862034-34524481	Gain	p13.33-p11.1	CCND2, ZNF384, ETV6, KRAS	6 (20.7%)	GC01, GC02, GC04, GC07, GC15, GC16
chr14:20219083-106778612	Gain	q11.2-q32.33	CCNB1IP1, TRA@, NKX2-1, NIN, KTN1, GPHN, TSHR, TRIP11, GOLGA5, DICER1, TCL6, TCL1A, BCL11B, AKT1, IGH@	10 (34.5%)	GC03, GC04, GC05, GC08, GC09, GC15, GC16, GC21, GC22, GC26
chrX:1796873-58470802	Loss	p22.33-p11.1	ZRSR2, BCOR, KDM6A, SSX1, SSX4, WAS, GATA1, TFE3, SSX2, KDM5C	9 (31.0%)	GC01, GC08, GC13, GC16, GC22, GC24, GC25, GC26, GC28
chrX:61742360-155270560	Loss	q11.1-q28	MSN, MED12, NONO, ATRX, SEPT6, ELF4, GPC3, PHF6, MTCP1	9 (31.0%)	GC01, GC08, GC13, GC16, GC22, GC25, GC26, GC28, GC29

Supplementary Table S5. The description of whole-exome sequencing data for 20 choriocarcinomas as well as HM/IM tissues

Sample ID	Tissue	Sequencing reads	Mapped reads (%)	Mapped reads in exon (%)	Coverage (mean)*	Percent of bases (>= 20 reads)*
GC02	Normal	97,020,393	90,668,277 / 93.5%	42,922,636 / 44.2%	60.15	87.48%
	Tumor	77,730,618	67,565,972 / 86.9%	42,148,019 / 54.2%	61.08	93.85%
GC08	Normal	100,496,402	100,063,462 / 99.6%	61,245,661 / 60.9%	102.26	97.04%
	Tumor	86,919,499	86,466,988 / 99.5%	60,713,564 / 69.9%	104.83	97.14%
GC09	Normal	65,043,173	64,877,800 / 99.8%	34,304,020 / 52.7%	54.54	67.82%
	Tumor	66,708,680	66,523,980 / 99.7%	31,522,140 / 47.3%	48.93	63.62%
GC10	Normal	110,477,371	110,088,208 / 99.7%	70,490,118 / 63.8%	119.82	97.68%
	Tumor	114,261,092	113,922,988 / 99.7%	65,654,749 / 57.5%	110.11	97.59%
GC11	Normal	83,696,616	83,492,382 / 99.8%	47,604,294 / 56.9%	76.53	73.86%
	Tumor	74,885,274	74,690,699 / 99.7%	39,429,250 / 52.7%	62.98	70.18%
GC13	Normal	102,791,494	102,444,133 / 99.7%	72,962,788 / 71.0%	124.31	96.42%
	Tumor	75,093,073	74,707,769 / 99.5%	56,198,985 / 74.8%	95.73	96.20%
GC15**	Normal	96,193,502	95,692,025 / 99.5%	71,339,060 / 74.2%	123.30	97.84%
	HM	152,295,457	151,702,727 / 99.6%	88,916,456 / 58.4%	152.47	98.61%
	IM	101,058,858	100,376,828 / 99.3%	57,972,299 / 57.4%	98.50	97.99%
	Tumor	72,875,110	72,157,056 / 99.0%	56,666,660 / 77.8%	97.35	96.58%
GC16	Normal	105,011,214	104,674,102 / 99.7%	76,917,624 / 73.3%	133.94	98.44%
	Tumor	95,541,954	95,253,607 / 99.7%	70,167,186 / 73.4%	120.69	97.02%
GC18	Normal	83,784,628	83,717,421 / 99.9%	53,463,750 / 63.8%	86.01	93.01%
	Tumor	99,091,548	99,038,466 / 99.9%	63,162,022 / 63.7%	104.52	95.19%
GC19	Tumor	77,157,014	77,041,008 / 99.9%	44,011,940 / 57.0%	66.85	91.60%
GC20	Tumor	94,623,491	94,549,357 / 99.9%	61,645,827 / 65.2%	103.04	96.60%
GC21	Tumor	77,779,434	77,573,013 / 99.7%	49,880,840 / 64.1%	77.27	95.00%
GC22	Tumor	111,716,469	111,636,105 / 99.9%	81,234,052 / 72.7%	139.29	98.05%
GC23	Tumor	101,285,293	101,146,743 / 99.9%	70,243,259 / 69.4%	118.65	96.86%
GC24	Normal	103,004,807	102,842,529 / 99.8%	71,519,365 / 69.4%	122.23	96.64%
	Tumor	104,186,550	104,026,684 / 99.9%	74,071,669 / 71.1%	126.60	97.35%
GC25	Normal	117,942,215	117,875,680 / 99.9%	86,013,680 / 72.9%	147.43	98.65%
	Tumor	113,317,419	113,204,901 / 99.9%	83,100,231 / 73.3%	143.32	98.68%
GC26	Tumor	119,425,467	119,299,106 / 99.9%	83,682,019 / 70.1%	143.11	98.84%
GC27	Normal	107,574,039	107,503,594 / 99.9%	80,277,623 / 74.6%	138.44	98.30%
	Tumor	111,606,225	111,487,130 / 99.9%	81,822,116 / 73.3%	140.43	98.43%
GC28	Tumor	113,107,909	113,016,444 / 99.9%	83,392,729 / 73.7%	143.20	98.56%
GC29	Normal	120,390,614	120,251,943 / 99.9%	87,164,589 / 72.4%	149.87	98.84%
	Tumor	122,217,082	122,160,128 / 99.9%	89,561,417 / 73.3%	154.07	98.61%

*The mean coverage and the % of bases (>= 20 reads) were calculated onto the targeted regions (Agilent SureSelect 50Mb exon).

**tissues of hydatidiform mole, invasive mole and choriocarcinoma were obtained as disease progressed

Supplementary Table S6. Somatic mutations detected in 20 choriocarcinomas as well as HM/IM tissues

Sample_ID	Chr	Position	Ref	Alt	Gene	ExonicFunction	AAChange	Altered read#	Total read#	Tumor VAF	COSMIC Census	COSMIC Mutation	Clin Var
GC02	1	983658	C	A	AGRN	Missense	p.H1340N	4	34	11.8%	.	.	.
GC02	1	4772643	A	T	AJAP1	Missense	p.E238V	7	30	23.3%	.	.	.
GC02	1	6530384	G	A	PLEKHG5	Missense	p.T571I	6	52	11.5%	.	.	.
GC02	1	20517226	G	A	UBXN10	Missense	p.V58M	4	31	12.9%	.	.	.
GC02	1	27278472	C	T	KDF1	Missense	p.V134M	4	25	16.0%	.	.	.
GC02	1	27690760	C	A	MAP3K6	Missense	p.E202D	4	26	15.4%	.	.	.
GC02	1	27721071	G	T	GPR3	Missense	p.A257S	4	30	13.3%	.	.	.
GC02	1	39879565	G	C	KIAA0754	Missense	p.A1210P	6	25	24.0%	.	.	.
GC02	1	39895547	G	A	MACF1	Missense	p.R3584H	4	28	14.3%	.	O	.
GC02	1	42050219	G	C	HIVEP3	Missense	p.P84A	4	32	12.5%	.	.	.
GC02	1	43213435	C	T	P3H1	Missense	p.G625R	6	24	25.0%	.	.	.
GC02	1	45125852	G	C	TMEM53	Missense	p.H59Q	4	36	11.1%	.	.	.
GC02	1	45797911	G	A	MUTYH	Missense	p.A144V	4	30	13.3%	O	.	.
GC02	1	55089026	C	T	FAM151A	Missense	p.V15M	4	20	20.0%	.	.	.
GC02	1	60520914	G	T	C1orf87	Missense	p.L102I	5	64	7.8%	.	.	.
GC02	1	64644053	C	G	ROR1	Missense	p.P777A	4	35	11.4%	.	.	.
GC02	1	75072293	A	T	ERICH3	Nonsense	p.L494X	6	32	18.8%	.	.	.
GC02	1	75072294	A	T	ERICH3	Missense	p.L494I	5	32	15.6%	.	.	.
GC02	1	110593606	G	T	STRIP1	Missense	p.W567L	7	32	21.9%	.	.	.
GC02	1	119428119	G	C	TBX15	Missense	p.P349A	4	29	13.8%	.	.	.
GC02	1	145474739	C	T	ANKRD34A	Missense	p.R471C	4	25	16.0%	.	.	.
GC02	1	149885095	C	T	SV2A	Missense	p.G100S	5	37	13.5%	.	.	.
GC02	1	150444227	G	A	RPRD2	Missense	p.D909N	7	53	13.2%	.	.	.
GC02	1	153723641	C	T	INTS3	Missense	p.H219Y	8	63	12.7%	.	.	.
GC02	1	155448139	G	A	ASH1L	Nonsense	p.R1508X	8	49	16.3%	.	.	.
GC02	1	156228807	G	T	SMG5	Missense	p.Q765K	7	46	15.2%	.	.	.
GC02	1	162040073	G	C	NOS1AP	Splicing	c.105+1G>C	3	21	14.3%	.	.	.
GC02	1	167096828	C	G	DUSP27	Missense	p.S820R	6	39	15.4%	.	.	.
GC02	1	205273049	C	A	NUAK2	Missense	p.L516F	4	39	10.3%	.	.	.

GC02	1	208390667	G	A	PLXNA2	Missense	p.R201W	4	23	17.4%	.	.	.
GC02	1	214171265	C	A	PROX1	Missense	p.P463T	6	54	11.1%	.	.	.
GC02	1	237889589	G	A	RYR2	Missense	p.G3569D	7	40	17.5%	.	.	.
GC02	1	240656310	G	A	GREM2	Missense	p.R156W	6	58	10.3%	.	.	.
GC02	1	241850796	G	A	WDR64	Missense	p.M281I	4	26	15.4%	.	.	.
GC02	2	25523022	G	A	DNMT3A	Missense	p.R55C	4	31	12.9%	O	O	.
GC02	2	33745023	C	T	RASGRP3	Nonsense	p.R60X	5	38	13.2%	.	O	.
GC02	2	48809586	G	A	STON1	Missense	p.S605N	5	43	11.6%	.	.	.
GC02	2	61118940	C	T	REL	Nonsense	p.R45X	5	43	11.6%	.	.	.
GC02	2	68385615	G	A	PNO1	Missense	p.G104E	5	30	16.7%	.	.	.
GC02	2	70096996	G	T	GMCL1	Missense	p.R455I	5	44	11.4%	.	.	.
GC02	2	85628344	C	T	CAPG	Missense	p.A154T	8	41	19.5%	.	O	.
GC02	2	88913309	C	T	EIF2AK3	Missense	p.G124D	6	36	16.7%	.	.	.
GC02	2	119732007	G	A	MARCO	Missense	p.G187R	6	31	19.4%	.	.	.
GC02	2	128528489	G	A	WDR33	Nonsense	p.R23X	5	33	15.2%	.	O	.
GC02	2	178095601	G	A	NFE2L2	Missense	p.S547F	5	42	11.9%	O	O	.
GC02	2	179444687	G	A	TTN	Missense	p.R13378C	9	36	25.0%	.	O	.
GC02	2	211541772	C	A	CPS1	Missense	p.P988H	4	30	13.3%	.	.	.
GC02	2	215279053	G	T	VWC2L	Missense	p.D46Y	9	55	16.4%	.	.	.
GC02	2	215279077	G	T	VWC2L	Missense	p.V54F	8	53	15.1%	.	.	.
GC02	2	219612393	G	T	TTLL4	Missense	p.D775Y	5	46	10.9%	.	.	.
GC02	2	219689007	C	T	PRKAG3	Missense	p.V431I	4	34	11.8%	.	.	.
GC02	2	233323719	G	C	ALPI	Missense	p.A484P	5	24	20.8%	.	.	.
GC02	3	14485276	C	T	SLC6A6	Missense	p.S45F	5	35	14.3%	.	.	.
GC02	3	14952404	G	A	FGD5	Missense	p.G1125R	10	45	22.2%	.	.	.
GC02	3	29476298	A	G	RBMS3	Missense	p.N46S	4	33	12.1%	.	O	.
GC02	3	43344921	G	A	SNRK	Missense	p.V76I	5	59	8.5%	.	.	.
GC02	3	44284670	A	T	TOPAZ1	Missense	p.L224F	4	27	14.8%	.	.	.
GC02	3	47719760	A	T	SMARCC1	Missense	p.L500Q	5	30	16.7%	.	.	.
GC02	3	49688156	C	T	BSN	Missense	p.P544S	4	29	13.8%	.	.	.
GC02	3	51746975	G	A	GRM2	Missense	p.G313S	10	35	28.6%	.	.	.

GC02	3	56763312	C	T	ARHGEF3	Missense	p.E529K	9	41	22.0%	.	.	.
GC02	3	99569350	T	C	FILIP1L	Missense	p.I150M	5	77	6.5%	.	.	.
GC02	3	114070254	C	T	ZBTB20	Missense	p.S224N	4	34	11.8%	.	.	.
GC02	3	119469918	G	A	MAATS1	Missense	p.E634K	4	23	17.4%	.	O	.
GC02	3	122288348	C	T	DTX3L	Missense	p.A471V	4	28	14.3%	.	.	.
GC02	3	123332960	C	T	MYLK	Missense	p.E152K	5	56	8.9%	.	.	.
GC02	3	129286376	G	C	PLXND1	Missense	p.L1349V	5	38	13.2%	.	.	.
GC02	3	190282077	G	A	IL1RAP	Splicing	.	4	28	14.3%	.	.	.
GC02	4	3318016	C	T	RGS12	Missense	p.A40V	6	31	19.4%	.	.	.
GC02	4	15638261	G	A	FBXL5	Missense	p.L207F	11	37	29.7%	.	.	.
GC02	4	22390543	C	A	ADGRA3	Missense	p.L917F	10	34	29.4%	.	.	.
GC02	4	38016578	G	A	TBC1D1	Missense	p.R289Q	4	34	11.8%	.	O	.
GC02	4	40245262	G	A	RHOH	Missense	p.A86T	4	36	11.1%	.	.	.
GC02	4	74279143	C	A	ALB	Missense	p.L284I	5	42	11.9%	.	.	.
GC02	4	76489361	A	G	C4orf26	Missense	p.K50R	17	52	32.7%	.	.	.
GC02	4	96762085	G	A	PDHA2	Missense	p.E262K	6	47	12.8%	.	.	.
GC02	4	115544529	C	T	UGT8	Missense	p.P165S	9	44	20.5%	.	.	.
GC02	4	123193320	C	T	KIAA1109	Missense	p.R2736W	6	25	24.0%	.	O	.
GC02	4	141888986	C	T	RNF150	Missense	p.G176R	5	49	10.2%	.	.	.
GC02	4	152096158	G	A	SH3D19	Missense	p.P120S	4	34	11.8%	.	.	.
GC02	4	152499022	G	A	FAM160A1	Missense	p.D176N	6	56	10.7%	.	.	.
GC02	4	154216897	G	A	TRIM2	Missense	p.E380K	4	42	9.5%	.	.	.
GC02	4	165878544	G	A	FAM218A	Missense	p.V124I	4	37	10.8%	.	.	.
GC02	5	163257	G	A	PLEKHG4B	Missense	p.E1024K	4	27	14.8%	.	.	.
GC02	5	3599798	G	A	IRX1	Missense	p.E246K	5	49	10.2%	.	.	.
GC02	5	35065910	C	T	PRLR	Missense	p.E283K	6	41	14.6%	.	.	.
GC02	5	38944627	G	A	RICTOR	Missense	p.L1289F	9	39	23.1%	.	.	.
GC02	5	43039746	C	T	ANXA2R	Missense	p.D135N	4	29	13.8%	.	.	.
GC02	5	56231527	G	T	MIER3	Missense	p.T168K	5	59	8.5%	.	.	.
GC02	5	78610120	G	A	JMY	Missense	p.R702Q	4	28	14.3%	.	.	.
GC02	5	127595224	C	T	FBN2	Missense	p.E2888K	4	35	11.4%	.	.	.

GC02	5	139917093	G	T	ANKHD1	Missense	p.A2383S	5	51	9.8%	.	.	.
GC02	5	141359788	C	T	RNF14	Missense	p.P186L	4	34	11.8%	.	O	.
GC02	5	145509680	C	G	LARS	Missense	p.M797I	4	31	12.9%	.	.	.
GC02	5	145838700	C	T	TCERG1	Missense	p.A231V	5	29	17.2%	.	.	.
GC02	5	149759266	G	A	TCOF1	Missense	p.E867K	6	31	19.4%	.	.	.
GC02	5	153432582	C	T	MFAP3	Missense	p.S133F	6	40	15.0%	.	.	.
GC02	5	154183768	G	A	LARP1	Missense	p.R739Q	5	37	13.5%	.	O	.
GC02	5	159842174	C	T	SLU7	Missense	p.R43Q	5	42	11.9%	.	O	.
GC02	5	172096932	C	T	NEURL1B	Missense	p.A59V	5	39	12.8%	.	.	.
GC02	5	172661851	G	A	NKX2-5	Missense	p.P79L	7	48	14.6%	.	.	.
GC02	5	175717647	A	T	SIMC1	Missense	p.T374S	5	36	13.9%	.	.	.
GC02	5	175717648	C	T	SIMC1	Missense	p.T374I	5	36	13.9%	.	.	.
GC02	5	176002158	G	A	CDHR2	Missense	p.S190N	4	28	14.3%	.	.	.
GC02	5	176520412	C	T	FGFR4	Missense	p.P368L	5	32	15.6%	O	.	.
GC02	5	178035447	G	A	CLK4	Missense	p.S355F	5	37	13.5%	.	.	.
GC02	6	7374154	G	T	CAGE1	Missense	p.Q164K	6	54	11.1%	.	.	.
GC02	6	25921528	G	T	SLC17A2	Missense	p.A118E	6	29	20.7%	.	.	.
GC02	6	28542734	G	C	ZBED9	Missense	p.S583C	3	22	13.6%	.	.	.
GC02	6	29797471	G	A	HLA-G	Splicing	c.895+1G>A	9	28	32.1%	.	.	.
GC02	6	30993554	G	A	MUC22	Missense	p.D116N	6	47	12.8%	.	.	.
GC02	6	30994949	G	A	MUC22	Missense	p.A581T	7	37	18.9%	.	.	.
GC02	6	30996519	A	G	MUC22	Missense	p.E1104G	11	51	21.6%	.	.	.
GC02	6	31323135	A	T	HLA-B	Missense	p.V285E	5	27	18.5%	.	.	.
GC02	6	31599622	C	T	PRRC2A	Nonsense	p.R1058X	4	25	16.0%	.	.	.
GC02	6	31608011	C	T	BAG6	Missense	p.A861T	4	36	11.1%	.	.	.
GC02	6	31868781	A	T	ZBTB12	Missense	p.V101D	4	26	15.4%	.	.	.
GC02	6	36106150	C	G	MAPK13	Missense	p.Q231E	4	31	12.9%	.	.	.
GC02	6	36891195	G	A	C6orf89	Missense	p.G348E	6	34	17.6%	.	O	.
GC02	6	42797170	C	A	BICRAL	Missense	p.Q367K	5	51	9.8%	.	.	.
GC02	6	76623817	G	T	MYO6	Missense	p.Q1136H	4	32	12.5%	.	.	.
GC02	6	84798904	G	A	MRAP2	Missense	p.G22S	5	42	11.9%	.	.	.

GC02	6	87969954	G	A	ZNF292	Missense	p.E2203K	5	28	17.9%	.	.	.
GC02	6	109175624	C	G	ARMC2	Missense	p.Q52E	17	42	40.5%	.	.	.
GC02	6	110746129	C	T	SLC22A16	Missense	p.G561R	4	29	13.8%	.	.	.
GC02	6	111894145	C	T	TRAF3IP2	Missense	p.V415M	4	37	10.8%	.	.	.
GC02	6	116757139	C	A	DSE	Missense	p.T503K	6	40	15.0%	.	.	.
GC02	6	143092937	G	A	HIVEP2	Missense	p.S980F	7	26	26.9%	.	O	.
GC02	7	27148210	G	A	HOXA3	Missense	p.P219L	4	35	11.4%	.	.	.
GC02	7	50097730	G	T	ZPBP	Missense	p.N113K	4	38	10.5%	.	.	.
GC02	7	75877528	C	A	SRRM3	Missense	p.Q86K	4	29	13.8%	.	.	.
GC02	7	86469030	G	A	GRM3	Missense	p.D734N	5	43	11.6%	.	.	.
GC02	7	91871403	C	T	KRIT1	Missense	p.R16H	5	48	10.4%	.	O	.
GC02	7	99129260	C	G	ZKSCAN5	Missense	p.N563K	7	44	15.9%	.	.	.
GC02	7	100680014	C	T	MUC17	Missense	p.P1773S	6	37	16.2%	.	.	.
GC02	7	102108756	A	G	LRWD1	Missense	p.K132R	4	32	12.5%	.	.	.
GC02	7	114655939	G	A	MDFIC	Missense	p.D231N	5	52	9.6%	.	.	.
GC02	7	128359042	C	T	FAM71F1	Nonsense	p.Q198X	4	30	13.3%	.	.	.
GC02	7	135123066	G	A	CNOT4	Missense	p.P5L	5	52	9.6%	.	.	.
GC02	7	143043672	G	A	CLCN1	Missense	p.G762D	4	32	12.5%	.	.	.
GC02	7	143054391	C	T	FAM131B	Missense	p.E104K	4	27	14.8%	.	O	.
GC02	7	154862865	G	A	HTR5A	Missense	p.D86N	7	35	20.0%	.	O	.
GC02	8	9437776	C	A	TNKS	Missense	p.H261N	7	58	12.1%	.	.	.
GC02	8	22874864	A	T	RHOBTB2	Missense	p.D696V	5	37	13.5%	.	.	.
GC02	8	23428923	C	A	SLC25A37	Missense	p.T40N	13	42	31.0%	.	.	.
GC02	8	30695362	T	C	TEX15	Missense	p.E2813G	5	40	12.5%	.	.	.
GC02	8	67577801	G	A	VCPIP1	Missense	p.R465C	6	74	8.1%	.	.	.
GC02	8	69243488	C	T	C8orf34	Missense	p.R81W	12	47	25.5%	.	.	.
GC02	8	77767637	C	T	ZFHX4	Missense	p.T2827I	7	53	13.2%	.	.	.
GC02	8	126369496	G	A	NSMCE2	Missense	p.E152K	13	43	30.2%	.	.	.
GC02	8	142367428	G	A	GPR20	Missense	p.A199V	7	32	21.9%	.	.	.
GC02	8	145584080	T	C	SLC52A2	Missense	p.Y310H	9	24	37.5%	.	.	.
GC02	8	145652322	G	A	VPS28	Missense	p.H3Y	6	51	11.8%	.	.	.

GC02	9	35546690	G	A	RUSC2	Missense	p.D58N	8	36	22.2%	.	.	.
GC02	9	72003309	G	T	FAM189A2	Missense	p.L364F	7	26	26.9%	.	.	.
GC02	9	77614728	C	T	CARNMT1	Missense	p.E138K	4	25	16.0%	.	.	.
GC02	9	104170273	G	A	ZNF189	Missense	p.E61K	4	33	12.1%	.	.	.
GC02	9	109688390	G	A	ZNF462	Missense	p.E733K	4	30	13.3%	.	O	.
GC02	9	112900315	C	A	AKAP2	Missense	p.Q689K	4	39	10.3%	.	.	.
GC02	9	125642953	G	A	RC3H2	Missense	p.R294C	4	24	16.7%	.	O	.
GC02	9	130580504	G	C	ENG	Missense	p.D527E	5	44	11.4%	.	.	.
GC02	9	134501387	C	T	RAPGEF1	Missense	p.V542M	5	46	10.9%	.	.	.
GC02	9	135939896	C	T	CEL	Missense	p.P61S	5	27	18.5%	.	.	.
GC02	9	139370464	C	T	SEC16A	Missense	p.G535D	4	34	11.8%	.	.	.
GC02	10	7786826	A	T	ITIH2	Missense	p.L827F	6	58	10.3%	.	.	.
GC02	10	50740980	G	T	ERCC6	Missense	p.Q11K	6	38	15.8%	.	.	.
GC02	10	71158559	G	A	HK1	Missense	p.G830R	4	29	13.8%	.	.	.
GC02	10	97817850	G	A	CCNJ	Missense	p.S335N	10	33	30.3%	.	.	.
GC02	10	101841346	G	A	CPN1	Missense	p.L13F	4	29	13.8%	.	.	.
GC02	10	103870446	G	A	LDB1	Missense	p.T122I	6	46	13.0%	.	.	.
GC02	10	104173701	G	T	PSD	Missense	p.P460T	5	29	17.2%	.	.	.
GC02	10	115947720	C	T	TDRD1	Missense	p.P44S	4	30	13.3%	.	O	.
GC02	10	123848059	G	T	TACC2	Missense	p.M1842I	10	21	47.6%	.	.	.
GC02	11	551723	C	T	LRRC56	Missense	p.S290F	4	24	16.7%	.	.	.
GC02	11	3845558	C	T	PGAP2	Missense	p.H74Y	6	37	16.2%	.	.	.
GC02	11	8252001	C	A	LMO1	Missense	p.G25C	37	56	66.1%	.	O	.
GC02	11	9009783	G	T	NRIP3	Missense	p.S74Y	6	38	15.8%	.	.	.
GC02	11	17660018	C	A	OTOG	Missense	p.P2618T	15	48	31.3%	.	.	.
GC02	11	47364455	C	A	MYBPC3	Missense	p.E461D	4	32	12.5%	.	.	.
GC02	11	62393298	G	A	GANAB	Missense	p.H828Y	7	56	12.5%	.	.	.
GC02	11	62415694	C	T	INTS5	Missense	p.E620K	6	46	13.0%	.	.	.
GC02	11	63764624	C	T	OTUB1	Missense	p.R176W	10	52	19.2%	.	.	.
GC02	11	64135643	A	T	RPS6KA4	Missense	p.N371Y	5	50	10.0%	.	.	.
GC02	11	64564322	C	T	MAP4K2	Missense	p.R476Q	5	56	8.9%	.	.	.

GC02	11	64669514	C	T	ATG2A	Missense	p.E1347K	6	41	14.6%	.	.	.
GC02	11	66043651	G	A	RAB1B	Missense	p.R183Q	7	39	17.9%	.	.	.
GC02	11	71820956	C	T	ANAPC15	Missense	p.E113K	4	30	13.3%	.	O	.
GC02	11	73715028	G	A	UCP3	Missense	p.S223F	4	26	15.4%	.	.	.
GC02	11	74979993	C	T	ARRB1	Missense	p.V337M	4	30	13.3%	.	.	.
GC02	11	107969257	G	A	CUL5	Splicing	c.2148+1G>A	7	26	26.9%	.	.	.
GC02	11	117301592	C	T	DSCAML1	Missense	p.M1904I	14	52	26.9%	.	.	.
GC02	11	134037982	G	A	NCAPD3	Missense	p.S1161F	6	34	17.6%	.	.	.
GC02	12	275030	C	T	IQSEC3	Missense	p.T679M	4	32	12.5%	.	O	.
GC02	12	995094	T	A	WNK1	Missense	p.S1461R	9	46	19.6%	.	.	.
GC02	12	6707152	G	T	CHD4	Missense	p.N593K	6	69	8.7%	O	.	.
GC02	12	6760499	C	T	ING4	Missense	p.R210Q	8	63	12.7%	.	.	.
GC02	12	48369230	G	T	COL2A1	Missense	p.D1183E	5	30	16.7%	O	.	.
GC02	12	49176779	T	C	ADCY6	Missense	p.M147V	5	32	15.6%	.	.	.
GC02	12	49177175	G	A	ADCY6	Missense	p.R15W	4	36	11.1%	.	.	.
GC02	12	49434040	C	T	KMT2D	Missense	p.E2505K	6	27	22.2%	O	.	.
GC02	12	49447916	G	A	KMT2D	Missense	p.S173F	4	32	12.5%	O	.	.
GC02	12	51318960	T	A	METTL7A	Missense	p.Y47N	5	55	9.1%	.	.	.
GC02	12	53662808	G	A	ESPL1	Missense	p.E28K	7	50	14.0%	.	.	.
GC02	12	57114854	G	A	NACA	Missense	p.P154S	7	68	10.3%	.	.	.
GC02	12	57559729	G	A	LRP1	Splicing	c.2671+1G>A	15	45	33.3%	.	.	.
GC02	12	57567601	A	G	LRP1	Missense	p.N1129D	5	39	12.8%	.	.	.
GC02	12	57604663	G	A	LRP1	Splicing	c.12916+1G>A	5	35	14.3%	.	.	.
GC02	12	69135707	G	A	NUP107	Missense	p.E844K	9	88	10.2%	.	.	.
GC02	12	76462716	C	T	NAP1L1	Missense	p.E26K	6	59	10.2%	.	.	.
GC02	12	99117517	C	A	APAF1	Missense	p.S1048Y	5	33	15.2%	.	.	.
GC02	12	109675181	C	A	ACACB	Missense	p.T1553K	5	35	14.3%	.	O	.
GC02	12	113645935	G	A	IQCD	Nonsense	p.Q13X	5	53	9.4%	.	.	.
GC02	12	113645943	G	A	IQCD	Missense	p.P10L	5	52	9.6%	.	.	.
GC02	12	132401607	G	A	ULK1	Missense	p.A728T	4	28	14.3%	.	.	.
GC02	13	24798203	T	A	SPATA13	Missense	p.V379D	6	33	18.2%	.	.	.

GC02	13	25744209	C	T	AMER2	Missense	p.V517I	4	37	10.8%	.	O	.
GC02	13	27847195	C	T	RASL11A	Missense	p.S56F	4	35	11.4%	.	.	.
GC02	13	42876714	C	T	AKAP11	Nonsense	p.R1278X	4	34	11.8%	.	O	.
GC02	13	77632484	C	T	MYCBP2	Missense	p.V4400I	4	25	16.0%	.	.	.
GC02	13	113897300	G	A	CUL4A	Missense	p.V352I	5	38	13.2%	.	.	.
GC02	14	23528595	C	T	ACIN1	Missense	p.R536Q	6	80	7.5%	.	.	.
GC02	14	39514456	G	A	SEC23A	Missense	p.R604C	6	47	12.8%	.	O	.
GC02	14	52906048	G	A	TXNDC16	Missense	p.L685F	6	38	15.8%	.	.	.
GC02	14	59112996	C	T	DACT1	Missense	p.A515V	4	25	16.0%	.	.	.
GC02	14	61186931	C	T	SIX4	Missense	p.G366R	5	37	13.5%	.	.	.
GC02	14	64953975	A	T	ZBTB25	Missense	p.V325E	5	33	15.2%	.	.	.
GC02	14	69256894	C	T	ZFP36L1	Missense	p.E125K	4	35	11.4%	.	.	.
GC02	14	105419969	C	T	AHNAK2	Missense	p.E507K	8	105	7.6%	.	.	.
GC02	15	34078091	C	T	RYR3	Missense	p.S3166F	4	36	11.1%	.	.	.
GC02	15	45556096	C	T	SLC28A2	Missense	p.S155F	6	55	10.9%	.	.	.
GC02	15	52258189	C	T	LEO1	Missense	p.E191K	6	73	8.2%	.	.	.
GC02	15	53081252	C	T	ONECUT1	Missense	p.G277D	5	26	19.2%	.	.	.
GC02	15	56996459	G	T	ZNF280D	Nonsense	p.S12X	6	35	17.1%	.	.	.
GC02	15	63926049	G	A	HERC1	Nonsense	p.Q4231X	5	40	12.5%	.	.	.
GC02	15	65684693	G	T	IGDCC4	Missense	p.A634E	7	22	31.8%	.	.	.
GC02	15	65917612	G	T	SLC24A1	Missense	p.K398N	7	42	16.7%	.	.	.
GC02	15	65992945	C	A	DENND4A	Nonsense	p.E923X	8	27	29.6%	.	.	.
GC02	15	89862218	G	T	POLG	Missense	p.P1073T	6	38	15.8%	.	.	.
GC02	16	844196	G	A	CHTF18	Missense	p.V649I	7	35	20.0%	.	.	.
GC02	16	1818749	G	A	MAPK8IP3	Missense	p.S1306N	4	29	13.8%	.	.	.
GC02	16	2282879	G	A	E4F1	Missense	p.V285M	4	33	12.1%	.	.	.
GC02	16	2580848	C	A	CEMP1	Missense	p.R76M	5	21	23.8%	.	.	.
GC02	16	2580857	T	A	CEMP1	Missense	p.Q73L	5	20	25.0%	.	.	.
GC02	16	4625415	C	T	C16orf96	Missense	p.L312F	11	36	30.6%	.	.	.
GC02	16	4751112	G	T	ANKS3	Missense	p.S208R	8	43	18.6%	.	.	.
GC02	16	4933882	C	T	PPL	Missense	p.E1592K	9	67	13.4%	.	O	.

GC02	16	11137915	C	T	CLEC16A	Nonsense	p.R583X	4	35	11.4%	.	.	.
GC02	16	24372999	C	T	CACNG3	Missense	p.H255Y	9	52	17.3%	.	O	.
GC02	16	31123275	C	T	BCKDK	Nonsense	p.Q311X	6	31	19.4%	.	.	.
GC02	16	53276764	C	T	CHD9	Nonsense	p.R964X	4	22	18.2%	.	O	.
GC02	16	58554908	C	T	CNOT1	Missense	p.G2357E	5	48	10.4%	.	.	.
GC02	16	66600423	C	T	CMTM1	Missense	p.P3S	6	42	14.3%	.	.	.
GC02	16	67679508	G	A	CARMIL2	Missense	p.E36K	5	23	21.7%	.	.	.
GC02	16	67763670	G	A	RANBP10	Nonsense	p.R237X	4	25	16.0%	.	.	.
GC02	16	70509363	A	G	FUK	Missense	p.T830A	4	22	18.2%	.	.	.
GC02	16	75203654	G	A	ZFP1	Missense	p.V183I	4	27	14.8%	.	.	.
GC02	16	85839405	T	A	COX4I1	Missense	p.V103D	4	29	13.8%	.	.	.
GC02	16	90020713	T	A	DEF8	Missense	p.F18Y	4	23	17.4%	.	.	.
GC02	16	90020714	C	A	DEF8	Missense	p.F18L	4	22	18.2%	.	.	.
GC02	17	986834	C	T	ABR	Missense	p.D152N	6	46	13.0%	.	.	.
GC02	17	1548907	C	A	SCARF1	Missense	p.V29F	4	39	10.3%	.	.	.
GC02	17	1946217	C	T	OVCA2	Missense	p.S168L	5	26	19.2%	.	.	.
GC02	17	4088145	G	A	ANKFY1	Missense	p.P598L	5	48	10.4%	.	.	.
GC02	17	7227556	C	T	NEURL4	Missense	p.D645N	5	35	14.3%	.	.	.
GC02	17	7797802	G	A	CHD3	Missense	p.C441Y	4	37	10.8%	.	.	.
GC02	17	8170911	G	T	PFAS	Missense	p.G1104W	5	34	14.7%	.	.	.
GC02	17	10298527	C	T	MYH8	Missense	p.E1629K	4	32	12.5%	.	.	.
GC02	17	11572434	C	A	DNAH9	Missense	p.P929T	4	35	11.4%	.	.	.
GC02	17	15878073	C	T	ADORA2B	Missense	p.T139I	11	40	27.5%	.	.	.
GC02	17	18047267	G	A	MYO15A	Missense	p.D2044N	5	29	17.2%	.	.	.
GC02	17	21319163	G	A	KCNJ12	Missense	p.C170Y	5	57	8.8%	.	O	.
GC02	17	33806913	G	T	SLFN12L	Missense	p.P106T	4	38	10.5%	.	.	.
GC02	17	37565937	G	A	MED1	Missense	p.A846V	4	27	14.8%	.	.	.
GC02	17	38859917	G	A	KRT24	Missense	p.S10F	7	31	22.6%	.	.	.
GC02	17	38926279	C	T	KRT26	Missense	p.M259I	7	54	13.0%	.	.	.
GC02	17	42476843	G	A	GPATCH8	Missense	p.R790C	5	45	11.1%	.	O	.
GC02	17	43474297	C	T	ARHGAP27	Missense	p.A646T	5	44	11.4%	.	.	.

GC02	17	44828942	G	A	NSF	Missense	p.G706E	5	52	9.6%	.	.	.
GC02	17	48941131	C	T	TOB1	Missense	p.R83H	5	64	7.8%	.	.	.
GC02	17	49280091	C	T	MBTD1	Missense	p.R345Q	9	57	15.8%	.	.	.
GC02	17	53342861	C	T	HLF	Nonsense	p.R6X	6	47	12.8%	.	.	.
GC02	17	54990820	T	G	TRIM25	Missense	p.H177P	4	23	17.4%	.	.	.
GC02	17	54990821	G	A	TRIM25	Missense	p.H177Y	4	23	17.4%	.	.	.
GC02	17	57126541	G	T	TRIM37	Missense	p.H476N	7	31	22.6%	.	.	.
GC02	17	59560552	C	T	TBX4	Missense	p.P438L	5	48	10.4%	.	O	.
GC02	17	60023929	C	T	MED13	Nonsense	p.W2142X	10	25	40.0%	.	.	.
GC02	17	74739517	C	T	MFSD11	Nonsense	p.Q159X	6	42	14.3%	.	.	.
GC02	17	74944751	C	T	MGAT5B	Missense	p.S746L	5	31	16.1%	.	.	.
GC02	17	77092793	G	A	RBFOX3	Missense	p.S293F	5	52	9.6%	.	.	.
GC02	17	79495663	T	G	FSCN2	Missense	p.S36A	4	27	14.8%	.	.	.
GC02	17	80202680	G	A	CSNK1D	Missense	p.L409F	5	34	14.7%	.	.	.
GC02	18	2931295	G	A	LPIN2	Missense	p.S472F	7	62	11.3%	.	.	.
GC02	18	29054222	C	T	DSG3	Missense	p.S747L	7	43	16.3%	.	.	.
GC02	18	47803498	C	T	MBD1	Missense	p.D63N	5	48	10.4%	.	O	.
GC02	18	76754173	G	A	SALL3	Missense	p.V728M	9	35	25.7%	.	.	.
GC02	19	2290774	C	A	LINGO3	Missense	p.E334D	5	26	19.2%	.	.	.
GC02	19	4453478	G	A	UBXN6	Missense	p.P44S	4	27	14.8%	.	.	.
GC02	19	8503386	G	C	MARCH2	Missense	p.A163P	5	28	17.9%	.	.	.
GC02	19	9065134	C	T	MUC16	Missense	p.D7438N	7	40	17.5%	.	.	.
GC02	19	9270919	G	A	ZNF317	Missense	p.G168R	17	50	34.0%	.	.	.
GC02	19	10265344	C	T	DNMT1	Missense	p.G568R	4	25	16.0%	.	.	.
GC02	19	10335224	C	G	S1PR2	Missense	p.V120L	6	31	19.4%	.	.	.
GC02	19	10445300	C	T	ICAM3	Missense	p.E270K	5	31	16.1%	.	.	.
GC02	19	10794379	G	A	ILF3	Missense	p.R644Q	4	21	19.0%	.	O	.
GC02	19	11322779	C	T	DOCK6	Missense	p.V1514M	4	23	17.4%	.	.	.
GC02	19	12126861	C	T	ZNF433	Missense	p.R274K	9	42	21.4%	.	.	.
GC02	19	17411666	C	T	ABHD8	Missense	p.G254S	4	20	20.0%	.	O	.
GC02	19	17534395	G	A	MVB12A	Missense	p.G168S	5	43	11.6%	.	.	.

GC02	19	31768374	G	T	TSHZ3	Missense	p.D775E	4	39	10.3%	.	.	.
GC02	19	36498145	A	-	SYNE4	Frameshift	p.L102fs	24	51	47.1%	.	.	.
GC02	19	37005528	C	G	ZNF260	Missense	p.E205Q	5	38	13.2%	.	.	.
GC02	19	37005533	T	A	ZNF260	Missense	p.Q203L	5	35	14.3%	.	.	.
GC02	19	44242323	C	T	SMG9	Missense	p.R287H	5	49	10.2%	.	O	.
GC02	19	45296847	-	C	CBLC	Frameshift	p.D372fs	19	62	30.6%	.	.	.
GC02	19	45561033	G	A	CLASRP	Missense	p.G102S	5	43	11.6%	.	O	.
GC02	19	47181862	G	A	PRKD2	Missense	p.S553L	8	42	19.0%	.	.	.
GC02	19	49573850	G	A	KCNA7	Nonsense	p.R281X	5	45	11.1%	.	O	.
GC02	19	51015474	G	A	ASPDH	Missense	p.H138Y	4	26	15.4%	.	.	.
GC02	19	55112196	C	T	LILRA1	Missense	p.L262F	4	30	13.3%	.	.	.
GC02	19	55451561	T	C	NLRP7	Missense	p.Y209C	20	38	52.6%	.	.	.
GC02	19	55489175	C	A	NLRP2	Missense	p.F127L	5	38	13.2%	.	.	.
GC02	19	57066004	T	A	ZFP28	Missense	p.F617Y	9	45	20.0%	.	.	.
GC02	19	58102080	G	A	ZIK1	Missense	p.G198R	5	39	12.8%	.	.	.
GC02	20	2443285	G	A	SNRPB	Nonsense	p.R228X	4	30	13.3%	.	O	.
GC02	20	31385029	G	A	DNMT3B	Missense	p.D376N	7	48	14.6%	.	.	.
GC02	20	34090887	G	A	CEP250	Missense	p.E932K	7	47	14.9%	.	.	.
GC02	20	44444167	C	T	UBE2C	Missense	p.L90F	4	29	13.8%	.	.	.
GC02	20	45130384	G	A	ZNF334	Missense	p.L532F	4	39	10.3%	.	.	.
GC02	20	46265024	C	T	NCOA3	Missense	p.R632W	4	32	12.5%	.	O	.
GC02	21	34924764	C	T	SON	Missense	p.S1076F	5	49	10.2%	.	.	.
GC02	22	20130519	G	T	ZDHHC8	Missense	p.G456W	5	24	20.8%	.	.	.
GC02	22	31091117	C	T	OSBP2	Missense	p.S74L	5	31	16.1%	.	.	.
GC02	22	31979896	G	T	SFI1	Missense	p.Q373H	5	44	11.4%	.	O	.
GC02	22	36538048	C	T	APOL3	Missense	p.E137K	8	27	29.6%	.	.	.
GC02	22	41545852	C	T	EP300	Missense	p.P823S	6	49	12.2%	O	.	.
GC02	22	46655363	G	T	PKDREJ	Missense	p.T1286K	7	46	15.2%	.	.	.
GC02	22	51065611	G	A	ARSA	Missense	p.P150S	5	29	17.2%	.	.	.
GC02	X	38178202	C	T	RPGR	Missense	p.G117R	5	32	15.6%	.	.	.
GC02	X	48382202	C	A	EBP	Missense	p.H15N	5	39	12.8%	.	.	.

GC02	X	48681568	T	C	HDAC6	Missense	p.L920P	9	45	20.0%	.	.	.
GC02	X	50350729	-	TCC	SHROOM4	Nonframeshift	p.E1138delins GK	27	80	33.8%	.	.	.
GC02	X	75650496	G	A	MAGEE1	Missense	p.D725N	6	36	16.7%	.	.	.
GC02	X	106844553	A	T	FRMPD3	Missense	p.Q1128L	8	31	25.8%	.	.	.
GC02	X	107393387	G	T	ATG4A	Nonsense	p.E212X	4	29	13.8%	.	.	.
GC02	X	107553977	C	T	COL4A6	Splicing	c.144+1G>A;NM_033641	7	34	20.6%	.	.	.
GC02	X	152826228	G	A	ATP2B3	Missense	p.M978I	6	38	15.8%	.	.	.
GC08	1	9778767	G	T	PIK3CD	Missense	p.G317W	5	60	8.3%	.	.	.
GC08	1	11026465	C	A	C1orf127	Missense	p.G108C	5	62	8.1%	.	.	.
GC08	1	11562093	G	T	DISP3	Missense	p.R348S	4	26	15.4%	.	.	.
GC08	1	13910561	G	C	PDPN	Missense	p.L87F	25	48	52.1%	.	.	.
GC08	1	20470031	G	T	PLA2G2F	Missense	p.G88C	4	30	13.3%	.	.	.
GC08	1	20964372	C	A	PINK1	Missense	p.P142Q	5	65	7.7%	.	.	.
GC08	1	22202814	G	T	HSPG2	Missense	p.P972T	5	48	10.4%	.	.	.
GC08	1	26688171	G	T	ZNF683	Missense	p.L496M	5	49	10.2%	.	.	.
GC08	1	26772827	G	T	DHDDS	Missense	p.G115V	6	55	10.9%	.	.	.
GC08	1	27745508	C	A	WASF2	Missense	p.R71M	6	85	7.1%	.	.	.
GC08	1	32950863	G	T	ZBTB8B	Missense	p.L444F	8	119	6.7%	.	.	.
GC08	1	36028910	G	T	NCDN	Missense	p.W481L	7	66	10.6%	.	O	.
GC08	1	38489217	G	T	UTP11	Missense	p.D227Y	61	84	72.6%	.	.	.
GC08	1	39782191	C	A	MACF1	Nonsense	p.S1198X	4	28	14.3%	.	.	.
GC08	1	43201620	T	C	CLDN19	Missense	p.H157R	26	35	74.3%	.	.	.
GC08	1	43828786	C	A	CDC20	Missense	p.Q496K	8	128	6.3%	.	.	.
GC08	1	44056994	G	T	PTPRF	Missense	p.W434L	5	44	11.4%	.	.	.
GC08	1	44423171	G	T	IPO13	Missense	p.R497L	6	95	6.3%	.	.	.
GC08	1	44877783	G	T	RNF220	Missense	p.R5L	6	87	6.9%	.	.	.
GC08	1	45140026	G	T	TMEM53	Missense	p.P13Q	6	96	6.3%	.	.	.
GC08	1	45266582	C	A	PLK3	Missense	p.P94Q	7	99	7.1%	.	.	.
GC08	1	45671754	G	T	ZSWIM5	Missense	p.P90Q	7	97	7.2%	.	.	.
GC08	1	45808275	C	A	TOE1	Missense	p.R238S	6	95	6.3%	.	.	.
GC08	1	52293539	C	A	NRDC	Missense	p.W290L	8	118	6.8%	.	.	.

GC08	1	76228447	-	A	ACADM	Frameshift	p.X233delinsX	29	50	58.0%	.	.	.
GC08	1	91403345	G	T	ZNF644	Missense	p.R1129S	7	126	5.6%	.	.	.
GC08	1	112999474	G	T	CTTNBP2N L	Missense	p.G454W	7	112	6.3%	.	.	.
GC08	1	113255063	C	A	PPM1J	Missense	p.R249L	6	35	17.1%	.	.	.
GC08	1	117122286	-	TCC	IGSF3	Nonframeshift	p.D1021delins GN	80	150	53.3%	.	.	.
GC08	1	117699505	C	A	VTCN1	Missense	p.G46W	8	129	6.2%	.	.	.
GC08	1	144923740	G	T	PDE4DIP	Missense	p.H403N	8	141	5.7%	.	.	.
GC08	1	145578353	C	A	PIAS3	Missense	p.L106M	9	98	9.2%	.	.	.
GC08	1	146672844	G	T	FMO5	Missense	p.P358H	7	77	9.1%	.	.	.
GC08	1	152382747	G	T	CRNN	Missense	p.H271N	7	92	7.6%	.	.	.
GC08	1	154544300	G	T	CHRNB2	Missense	p.W334L	8	114	7.0%	.	.	.
GC08	1	155176073	C	A	THBS3	Missense	p.Q68H	6	56	10.7%	.	.	.
GC08	1	156883052	C	A	PEAR1	Missense	p.P830Q	8	115	7.0%	.	.	.
GC08	1	161833069	G	T	ATF6	Missense	p.R562S	6	79	7.6%	.	.	.
GC08	1	162560301	G	T	UAP1	Missense	p.R453L	8	118	6.8%	.	.	.
GC08	1	166039512	C	A	FAM78B	Missense	p.R251L	5	63	7.9%	.	.	.
GC08	1	169565319	C	A	SELP	Missense	p.G649W	7	73	9.6%	.	.	.
GC08	1	197480862	G	T	DENND1B	Missense	p.P604H	6	64	9.4%	.	.	.
GC08	1	203274766	C	A	BTG2	Missense	p.P11Q	9	187	4.8%	.	.	.
GC08	1	221057646	C	A	HLX	Missense	p.P356Q	7	113	6.2%	.	.	.
GC08	1	224621444	G	T	WDR26	Missense	p.Q122K	6	87	6.9%	.	.	.
GC08	1	227149101	G	T	COQ8A	Missense	p.L5F	5	45	11.1%	.	.	.
GC08	1	228596323	G	T	TRIM17	Missense	p.P338H	8	142	5.6%	.	.	.
GC08	1	230829049	G	T	COG2	Missense	p.L708F	9	142	6.3%	.	.	.
GC08	1	231673019	G	T	TSNAX	Missense	p.R61L	7	141	5.0%	.	.	.
GC08	1	231830319	G	T	DISC1	Missense	p.R272L	7	131	5.3%	.	.	.
GC08	1	233136134	G	T	PCNX2	Missense	p.L1749M	7	103	6.8%	.	O	.
GC08	1	234743082	G	T	IRF2BP2	Missense	p.P506Q	8	129	6.2%	.	.	.
GC08	1	235866199	C	A	LYST	Missense	p.G3408W	7	117	6.0%	.	.	.
GC08	1	235940434	C	A	LYST	Nonsense	p.E1797X	6	96	6.3%	.	.	.
GC08	1	237850771	G	T	RYR2	Nonsense	p.G3012X	12	186	6.5%	.	.	.

GC08	1	240975323	C	A	RGS7	Missense	p.R273M	9	173	5.2%	.	.	.
GC08	1	248129487	G	T	OR2AK2	Missense	p.R285L	7	99	7.1%	.	O	.
GC08	2	3660908	G	T	COLEC11	Missense	p.R17L	6	86	7.0%	.	.	.
GC08	2	24261159	C	A	WDCP	Missense	p.L402F	6	71	8.5%	.	.	.
GC08	2	25466832	G	T	DNMT3A	Missense	p.P472Q	6	87	6.9%	O	.	.
GC08	2	27455446	C	A	CAD	Missense	p.H863N	8	117	6.8%	.	.	.
GC08	2	27720118	G	A	GCKR	Missense	p.G23E	17	50	34.0%	.	.	.
GC08	2	30748613	C	A	LCLAT1	Missense	p.L53I	6	94	6.4%	.	.	.
GC08	2	32740096	G	T	BIRC6	Missense	p.M3536I	6	83	7.2%	.	.	.
GC08	2	63712085	G	T	WDPCP	Missense	p.P97Q	9	135	6.7%	.	.	.
GC08	2	74590244	G	T	DCTN1	Missense	p.H997N	6	76	7.9%	.	.	.
GC08	2	80831267	C	T	CTNNA2	Missense	p.S432F	11	39	28.2%	.	O	.
GC08	2	85554542	G	T	TGOLN2	Missense	p.Q105K	9	153	5.9%	.	.	.
GC08	2	88472748	G	T	THNSL2	Missense	p.G27W	6	91	6.6%	.	.	.
GC08	2	96947577	G	T	SNRNP200	Missense	p.Q1667K	6	81	7.4%	.	.	.
GC08	2	97031640	G	T	NCAPH	Missense	p.L439F	5	40	12.5%	.	.	.
GC08	2	98277086	C	A	ACTR1B	Missense	p.R46L	7	111	6.3%	.	O	.
GC08	2	99858901	G	T	LYG2	Missense	p.P189T	8	115	7.0%	.	.	.
GC08	2	102804349	G	T	IL1RL2	Missense	p.G8W	7	123	5.7%	.	.	.
GC08	2	113322024	G	T	POLR1B	Missense	p.W354L	6	89	6.7%	.	.	.
GC08	2	131415073	A	G	POTEJ	Missense	p.S914G	5	45	11.1%	.	.	.
GC08	2	167760293	C	A	XIRP2	Missense	p.L101M	6	93	6.5%	.	O	.
GC08	2	168114370	G	T	XIRP2	Missense	p.L216F	5	46	10.9%	.	O	.
GC08	2	179451522	G	T	TTN	Missense	p.P12304H	5	45	11.1%	.	.	.
GC08	2	180008390	G	T	SESTD1	Missense	p.R260S	6	92	6.5%	.	.	.
GC08	2	190320125	G	T	WDR75	Missense	p.L151F	7	103	6.8%	.	O	.
GC08	2	202153458	C	A	ALS2CR12	Missense	p.G397W	7	86	8.1%	.	.	.
GC08	2	202339452	G	T	STRADB	Missense	p.W133L	5	44	11.4%	.	.	.
GC08	2	207012341	C	A	NDUFS1	Missense	p.E44D	6	81	7.4%	.	.	.
GC08	2	220078385	C	A	ABCB6	Missense	p.G482W	7	97	7.2%	.	.	.
GC08	2	220089360	G	T	ATG9A	Missense	p.R245S	7	112	6.3%	.	.	.

GC08	2	220361591	-	A	LOC100996693	Frameshift	p.V63fs	17	49	34.7%	.	.	.
GC08	2	233387890	C	T	PRSS56	Missense	p.A276V	39	118	33.1%	.	.	.
GC08	2	236761415	-	CAGG	AGAP1	Frameshift	p.P379fs	117	203	57.6%	.	.	.
GC08	2	241706392	C	A	KIF1A	Missense	p.G533W	5	39	12.8%	.	.	.
GC08	3	9825845	G	T	TADA3	Missense	p.Q325K	8	125	6.4%	.	.	.
GC08	3	12857922	G	T	CAND2	Missense	p.M404I	7	99	7.1%	.	.	.
GC08	3	25679800	C	A	TOP2B	Nonsense	p.E128X	5	23	21.7%	.	.	.
GC08	3	37032815	C	A	EPM2AIP1	Missense	p.R585L	8	135	5.9%	.	.	.
GC08	3	43074218	C	A	FAM198A	Missense	p.Q155K	6	69	8.7%	.	.	.
GC08	3	50212622	G	A	SEMA3F	Splicing	c.549+1G>A	15	52	28.8%	.	.	.
GC08	3	51399392	G	T	DOCK3	Missense	p.M1703I	6	75	8.0%	.	.	.
GC08	3	52817016	G	T	ITIH1	Missense	p.G37V	6	82	7.3%	.	.	.
GC08	3	57107807	C	A	SPATA12	Missense	p.L29I	29	94	30.9%	.	.	.
GC08	3	58109006	G	T	FLNB	Missense	p.G1105W	6	94	6.4%	.	.	.
GC08	3	87322579	C	A	POU1F1	Missense	p.L70F	7	63	11.1%	.	.	.
GC08	3	100595376	G	T	ABI3BP	Missense	p.P249Q	6	73	8.2%	.	.	.
GC08	3	101447722	G	T	CEP97	Missense	p.D130Y	6	87	6.9%	.	.	.
GC08	3	113125779	C	A	CFAP44	Missense	p.G334W	6	65	9.2%	.	.	.
GC08	3	122835077	G	T	PDIA5	Splicing	c.542-1G>T	6	95	6.3%	.	.	.
GC08	3	135720690	T	G	PPP2R3A	Missense	p.I117S	5	109	4.6%	.	.	.
GC08	3	136162223	C	A	STAG1	Missense	p.L484F	8	129	6.2%	.	.	.
GC08	3	140281941	A	T	CLSTN2	Missense	p.D793V	23	78	29.5%	.	.	.
GC08	3	142261573	C	A	ATR	Missense	p.L1128F	6	68	8.8%	O	.	.
GC08	3	142542399	C	A	PCOLCE2	Missense	p.E308D	8	138	5.8%	.	.	.
GC08	3	148545680	G	T	CPB1	Missense	p.G24C	7	90	7.8%	.	.	.
GC08	3	151161167	C	A	IGSF10	Missense	p.W1856C	6	81	7.4%	.	O	.
GC08	3	161221488	G	T	OTOL1	Missense	p.G398W	6	65	9.2%	.	.	.
GC08	3	183479340	G	T	YEATS2	Missense	p.G568W	6	78	7.7%	.	.	.
GC08	3	183755920	G	T	HTR3D	Missense	p.G85W	8	139	5.8%	.	.	.
GC08	3	183823671	C	A	HTR3E	Missense	p.P306Q	6	93	6.5%	.	.	.
GC08	3	186793468	G	T	ST6GAL1	Missense	p.M366I	8	111	7.2%	.	.	.

GC08	4	6956063	C	G	TBC1D14	Missense	p.D12E	28	66	42.4%	.	.	.
GC08	4	7698965	G	A	SORCS2	Missense	p.M544I	16	25	64.0%	.	.	.
GC08	4	71024289	G	T	PRR27	Missense	p.R107M	8	99	8.1%	.	O	.
GC08	4	75248372	G	T	EREG	Missense	p.G97C	6	91	6.6%	.	.	.
GC08	4	84383717	G	T	ABRAXAS1	Missense	p.Q270K	6	91	6.6%	.	.	.
GC08	4	88036338	G	T	AFF1	Missense	p.G416W	6	91	6.6%	.	.	.
GC08	4	106359189	C	A	PPA2	Missense	p.W149L	6	76	7.9%	.	O	.
GC08	4	115544322	G	T	UGT8	Missense	p.G96W	6	61	9.8%	.	.	.
GC08	4	134072051	A	C	PCDH10	Missense	p.Q252H	30	84	35.7%	.	.	.
GC08	4	152638129	G	T	GATB	Missense	p.P180H	6	74	8.1%	.	.	.
GC08	4	158091725	G	T	GLRB	Nonsense	p.G447X	7	79	8.9%	.	O	.
GC08	4	169835107	G	T	PALLD	Missense	p.M380I	5	39	12.8%	.	.	.
GC08	4	177071084	G	T	WDR17	Missense	p.W675L	6	77	7.8%	.	.	.
GC08	4	177098226	G	T	WDR17	Missense	p.R1156L	6	83	7.2%	.	.	.
GC08	4	184366693	C	A	CDKN2AIP	Missense	p.P93H	6	50	12.0%	.	.	.
GC08	4	186381254	G	T	CCDC110	Missense	p.Q126K	6	83	7.2%	.	.	.
GC08	4	189012771	G	T	TRIML2	Missense	p.P357H	6	98	6.1%	.	.	.
GC08	5	7873625	G	T	MTRR	Missense	p.R90L	5	51	9.8%	.	.	.
GC08	5	10261857	G	T	CCT5	Missense	p.M338I	6	75	8.0%	.	.	.
GC08	5	13901663	C	A	DNAH5	Missense	p.G584C	6	49	12.2%	.	.	.
GC08	5	14609050	G	T	FAM105A	Missense	p.R274M	6	83	7.2%	.	.	.
GC08	5	54577331	C	A	DHX29	Nonsense	p.G609X	6	94	6.4%	.	.	.
GC08	5	54635891	C	A	SKIV2L2	Missense	p.P190Q	7	80	8.8%	.	.	.
GC08	5	78329110	G	T	DMGDH	Missense	p.Q439K	8	88	9.1%	.	.	.
GC08	5	121409809	G	T	LOX	Missense	p.Q82K	7	65	10.8%	.	.	.
GC08	5	131630314	G	T	SLC22A4	Missense	p.R2L	7	99	7.1%	.	.	.
GC08	5	133502930	C	A	SKP1	Missense	p.L34F	6	65	9.2%	.	O	.
GC08	5	134022502	T	G	SEC24A	Missense	p.V505G	5	113	4.4%	.	.	.
GC08	5	137427126	C	A	WNT8A	Missense	p.P353Q	6	67	9.0%	.	.	.
GC08	5	137781196	C	A	REEP2	Missense	p.P202Q	6	84	7.1%	.	.	.
GC08	5	140175955	C	A	PCDHA2	Missense	p.P469Q	5	52	9.6%	.	.	.

GC08	5	140740508	G	T	PCDHGB2	Missense	p.R269L	7	105	6.7%	.	.	.
GC08	5	140773467	G	T	PCDHGA8	Missense	p.G363W	7	107	6.5%	.	.	.
GC08	5	141337115	C	A	PCDH12	Missense	p.W101L	6	92	6.5%	.	.	.
GC08	5	145894953	G	T	GPR151	Missense	p.Q242K	6	64	9.4%	.	.	.
GC08	5	175793529	G	T	ARL10	Missense	p.W110C	6	86	7.0%	.	.	.
GC08	5	180477283	T	C	BTNL9	Missense	p.V217A	30	104	28.8%	.	.	.
GC08	6	2838140	G	T	SERPINB1	Missense	p.Q134K	8	114	7.0%	.	.	.
GC08	6	7405210	C	A	RIOK1	Missense	p.P247Q	7	103	6.8%	.	.	.
GC08	6	10398804	C	T	TFAP2A	Missense	p.G381D	24	59	40.7%	.	.	.
GC08	6	12123585	C	A	HIVEP1	Missense	p.P1186H	6	96	6.3%	.	.	.
GC08	6	16295348	-	ACTC	GMPR	Frameshift	p.K323fs	17	48	35.4%	.	.	.
GC08	6	26123855	C	A	HIST1H2BC	Missense	p.R93M	6	52	11.5%	.	.	.
GC08	6	31124578	G	T	CCHCR1	Missense	p.Q107K	5	46	10.9%	.	.	.
GC08	6	32013994	C	A	TNXB	Missense	p.G3520W	9	88	10.2%	.	.	.
GC08	6	34827117	C	A	UHRF1BP1	Missense	p.P995Q	6	86	7.0%	.	.	.
GC08	6	39159162	G	T	KCNK5	Missense	p.P335Q	7	82	8.5%	.	.	.
GC08	6	43475575	G	T	LRRC73	Missense	p.Q216K	7	100	7.0%	.	.	.
GC08	6	44274233	G	T	AARS2	Missense	p.Q396K	6	79	7.6%	.	.	.
GC08	6	75841726	G	T	COL12A1	Missense	p.P792H	6	95	6.3%	.	.	.
GC08	6	82882115	T	G	IBTK	Missense	p.K1289N	29	92	31.5%	.	.	.
GC08	6	108041970	C	A	SCML4	Missense	p.G62W	5	48	10.4%	.	.	.
GC08	6	111697606	G	T	REV3L	Missense	p.P651Q	6	70	8.6%	.	.	.
GC08	6	117859860	G	T	DCBLD1	Nonsense	p.G280X	7	99	7.1%	.	.	.
GC08	6	133836480	G	T	EYA4	Missense	p.R454M	7	92	7.6%	.	.	.
GC08	6	137245251	C	A	SLC35D3	Missense	p.P223Q	5	52	9.6%	.	.	.
GC08	6	142397029	C	A	NMBR	Missense	p.R162L	6	87	6.9%	.	.	.
GC08	6	144207187	T	C	ZC2HC1B	Missense	p.C37R	16	61	26.2%	.	.	.
GC08	6	152469432	C	A	SYNE1	Missense	p.D397Y	5	50	10.0%	.	.	.
GC08	6	158924256	G	T	TULP4	Missense	p.M1187I	7	88	8.0%	.	.	.
GC08	6	158994546	G	T	TMEM181	Missense	p.G172W	7	97	7.2%	.	.	.
GC08	6	160174499	G	T	WTAP	Missense	p.G154W	6	78	7.7%	.	.	.

GC08	6	166912082	C	A	RPS6KA2	Missense	p.G123W	6	73	8.2%	.	.	.
GC08	6	167754337	G	T	TTLL2	Missense	p.G317C	8	113	7.1%	.	.	.
GC08	7	2472956	C	A	CHST12	Missense	p.R228S	7	127	5.5%	.	.	.
GC08	7	24663333	G	T	MPP6	Missense	p.G16V	8	128	6.3%	.	.	.
GC08	7	30951637	C	A	AQP1	Missense	p.P38Q	7	131	5.3%	.	.	.
GC08	7	43351633	G	T	HECW1	Missense	p.W100L	9	115	7.8%	.	O	.
GC08	7	43495899	G	T	HECW1	Missense	p.W801L	9	150	6.0%	.	.	.
GC08	7	47944758	G	T	PKD1L1	Missense	p.Q563K	5	37	13.5%	.	.	.
GC08	7	73112161	G	T	BUD23	Splicing	c.843-1G>T	8	157	5.1%	.	.	.
GC08	7	93125241	C	T	CALCR	Missense	p.M1I	28	34	82.4%	.	.	.
GC08	7	97820122	C	A	LMTK2	Missense	p.Q361K	6	95	6.3%	.	.	.
GC08	7	97822533	C	A	LMTK2	Missense	p.P919Q	10	186	5.4%	.	O	.
GC08	7	98589778	C	A	TRRAP	Missense	p.Q3234K	7	115	6.1%	O	.	.
GC08	7	99377639	C	A	CYP3A4	Missense	p.L47F	6	51	11.8%	.	.	.
GC08	7	99527014	C	A	GJC3	Missense	p.W77L	6	50	12.0%	.	.	.
GC08	7	100410542	G	T	EPHB4	Missense	p.L649M	9	154	5.8%	.	O	.
GC08	7	121653244	C	A	PTPRZ1	Missense	p.H1382N	7	102	6.9%	.	.	.
GC08	7	127894626	G	T	LEP	Missense	p.R105L	7	119	5.9%	.	.	.
GC08	7	127961398	G	T	RBM28	Missense	p.P354Q	6	82	7.3%	.	.	.
GC08	7	127970892	G	T	RBM28	Missense	p.P229Q	7	115	6.1%	.	.	.
GC08	7	129910620	C	A	CPA2	Missense	p.L126M	5	49	10.2%	.	.	.
GC08	7	130027737	G	T	CPA1	Missense	p.R382L	8	79	10.1%	.	.	.
GC08	7	134001427	C	A	SLC35B4	Missense	p.R26L	5	46	10.9%	.	.	.
GC08	7	143092447	G	T	EPHA1	Missense	p.P683Q	7	132	5.3%	.	.	.
GC08	7	150558197	G	T	AOC1	Missense	p.R719L	8	121	6.6%	.	.	.
GC08	8	7308706	C	A	SPAG11B	Missense	p.R24L	7	124	5.6%	.	.	.
GC08	8	10555322	C	A	C8orf74	Missense	p.P152Q	6	82	7.3%	.	.	.
GC08	8	15095113	A	T	SGCZ	Missense	p.L7Q	14	46	30.4%	.	.	.
GC08	8	17218747	G	T	MTMR7	Missense	p.P116H	6	86	7.0%	.	.	.
GC08	8	21957343	G	T	FAM160B2	Missense	p.G427V	6	88	6.8%	.	.	.
GC08	8	25266387	G	T	DOCK5	Missense	p.G1779C	5	64	7.8%	.	.	.

GC08	8	30567347	C	A	GSR	Missense	p.G136C	8	117	6.8%	.	.	.
GC08	8	67039506	G	T	TRIM55	Missense	p.M1I	6	67	9.0%	.	.	.
GC08	8	74922360	G	T	LY96	Missense	p.K79N	5	47	10.6%	.	.	.
GC08	8	77896143	G	T	PEX2	Missense	p.P91H	6	77	7.8%	.	.	.
GC08	8	86385929	G	T	CA2	Missense	p.K80N	5	40	12.5%	.	.	.
GC08	8	91643855	C	A	TMEM64	Missense	p.L292F	6	81	7.4%	.	.	.
GC08	8	99961221	C	A	OSR2	Missense	p.P14Q	7	74	9.5%	.	.	.
GC08	8	100829871	G	T	VPS13B	Missense	p.R2759L	6	77	7.8%	.	.	.
GC08	8	113657397	G	T	CSMD3	Missense	p.P980Q	5	60	8.3%	.	O	.
GC08	8	121587359	C	A	SNTB1	Missense	p.S368I	20	62	32.3%	.	.	.
GC08	8	122626809	C	A	HAS2	Missense	p.R400L	6	94	6.4%	.	.	.
GC08	8	124989745	G	T	FER1L6	Missense	p.R320M	6	65	9.2%	.	.	.
GC08	8	130761712	G	T	GSDMC	Missense	p.Q425K	5	34	14.7%	.	.	.
GC08	8	144378363	G	T	ZNF696	Missense	p.R173L	7	147	4.8%	.	.	.
GC08	8	144804257	C	G	MAPK15	Missense	p.L491V	3	31	9.7%	.	.	.
GC08	8	145112365	G	T	OPLAH	Missense	p.R470S	5	56	8.9%	.	.	.
GC08	8	145726241	G	T	PPP1R16A	Missense	p.E308D	6	71	8.5%	.	.	.
GC08	9	5069161	A	C	JAK2	Missense	p.D340A	44	69	63.8%	O	.	.
GC08	9	12775728	C	A	LURAP1L	Missense	p.P5Q	8	112	7.1%	.	.	.
GC08	9	18826446	C	A	ADAMTSL1	Missense	p.Q1367K	6	79	7.6%	.	.	.
GC08	9	22008820	G	T	CDKN2B	Missense	p.R45S	5	47	10.6%	.	.	.
GC08	9	27524388	G	T	IFNK	Missense	p.M18I	6	80	7.5%	.	.	.
GC08	9	34611064	C	A	RPP25L	Missense	p.R77L	7	147	4.8%	.	.	.
GC08	9	35095357	C	A	PIGO	Missense	p.R69L	7	146	4.8%	.	.	.
GC08	9	35705973	G	T	TLN1	Missense	p.Q1833K	9	140	6.4%	.	.	.
GC08	9	37512596	C	A	FBXO10	Missense	p.R940L	6	95	6.3%	.	.	.
GC08	9	37746114	C	A	FRMPD1	Missense	p.P1362H	7	108	6.5%	.	.	.
GC08	9	71869274	G	T	TJP2	Missense	p.R1039L	7	100	7.0%	.	O	.
GC08	9	87475975	G	T	NTRK2	Missense	p.G473W	7	84	8.3%	.	.	.
GC08	9	96061441	G	T	WNK2	Missense	p.G2005W	6	80	7.5%	.	.	.
GC08	9	99522169	C	A	ZNF510	Missense	p.G253W	6	98	6.1%	.	.	.

GC08	9	109690506	C	A	ZNF462	Missense	p.P1438Q	7	120	5.8%	.	.	.
GC08	9	114128803	C	A	KIAA0368	Missense	p.G1918W	9	124	7.3%	.	.	.
GC08	9	117241006	C	A	WHRN	Missense	p.G222W	4	25	16.0%	.	.	.
GC08	9	123783894	G	T	C5	Missense	p.Q405K	8	89	9.0%	.	.	.
GC08	9	124989215	G	T	LHX6	Missense	p.P55Q	5	46	10.9%	.	.	.
GC08	9	130224587	C	A	LRSAM1	Missense	p.L155I	7	83	8.4%	.	.	.
GC08	9	130477839	G	T	PTRH1	Missense	p.P27Q	7	104	6.7%	.	.	.
GC08	9	136199094	C	A	SURF6	Missense	p.G233W	6	89	6.7%	.	.	.
GC08	9	139333256	G	T	INPP5E	Missense	p.L206M	6	68	8.8%	.	.	.
GC08	10	1405851	G	T	ADARB2	Missense	p.P150Q	7	122	5.7%	.	.	.
GC08	10	5789029	G	T	FAM208B	Missense	p.L1134F	8	145	5.5%	.	.	.
GC08	10	44876298	G	T	CXCL12	Missense	p.P31Q	6	65	9.2%	.	.	.
GC08	10	50574251	-	G	DRGX	Frameshift	p.P234fs	26	53	49.1%	.	.	.
GC08	10	54528151	G	T	MBL2	Missense	p.P165T	7	83	8.4%	.	.	.
GC08	10	64960315	C	G	JMJD1C	Missense	p.E1514Q	49	130	37.7%	.	.	.
GC08	10	70516073	C	A	CCAR1	Missense	p.H542N	6	95	6.3%	.	.	.
GC08	10	71851565	C	A	H2AFY2	Missense	p.P111H	7	110	6.4%	.	.	.
GC08	10	91098703	G	T	IFIT3	Missense	p.W97C	7	89	7.9%	.	.	.
GC08	10	91469819	C	T	KIF20B	Nonsense	p.Q163X	23	91	25.3%	.	.	.
GC08	10	101163279	C	A	GOT1	Missense	p.Q302H	6	57	10.5%	.	.	.
GC08	10	103827549	G	T	HPS6	Missense	p.R773L	5	48	10.4%	.	.	.
GC08	10	104262402	C	A	ACTR1A	Missense	p.M1I	7	96	7.3%	.	.	.
GC08	10	104629929	G	T	AS3MT	Missense	p.R44L	6	97	6.2%	.	.	.
GC08	10	123844783	G	T	TACC2	Missense	p.W923L	8	78	10.3%	.	.	.
GC08	10	124758072	G	T	IKZF5	Missense	p.H24N	7	118	5.9%	.	.	.
GC08	10	128925942	C	A	DOCK1	Missense	p.Q921K	7	89	7.9%	.	.	.
GC08	10	134158134	C	A	LRRC27	Missense	p.P51H	6	98	6.1%	.	.	.
GC08	11	199406	G	T	ODF3	Missense	p.R191L	6	66	9.1%	.	.	.
GC08	11	1030231	C	A	MUC6	Missense	p.G333W	5	51	9.8%	.	.	.
GC08	11	1256560	G	T	MUC5B	Missense	p.G933W	8	108	7.4%	.	.	.
GC08	11	1262378	G	T	MUC5B	Missense	p.R1423L	5	73	6.8%	.	.	.

GC08	11	1272811	G	T	MUC5B	Missense	p.G4901W	6	86	7.0%	.	.	.
GC08	11	2320866	-	G	C11orf21	Frameshift	p.A73fs	23	63	36.5%	.	.	.
GC08	11	4388696	G	T	OR52B4	Missense	p.P277Q	7	115	6.1%	.	.	.
GC08	11	4929326	C	A	OR51A7	Missense	p.H243N	7	98	7.1%	.	O	.
GC08	11	5152998	G	T	OR52A5	Missense	p.P292H	6	67	9.0%	.	.	.
GC08	11	6662746	-	CAG	DCHS1	Nonframeshift	p.L33delinsLW	28	54	51.9%	.	.	.
GC08	11	10050066	G	C	SBF2	Missense	p.I184M	37	124	29.8%	.	.	.
GC08	11	47266379	G	T	ACP2	Missense	p.Q164K	7	69	10.1%	.	.	.
GC08	11	48152172	G	T	PTPRJ	Missense	p.G507C	6	63	9.5%	.	.	.
GC08	11	60696300	C	A	TMEM132A	Missense	p.P245Q	6	78	7.7%	.	.	.
GC08	11	60906277	G	T	VPS37C	Missense	p.L9M	7	108	6.5%	.	.	.
GC08	11	63533560	G	T	C11orf95	Missense	p.P119H	6	57	10.5%	.	.	.
GC08	11	64593950	G	T	CDC42BPG	Missense	p.L1531I	5	46	10.9%	.	.	.
GC08	11	65350191	G	A	EHBP1L1	Missense	p.G683E	27	83	32.5%	.	.	.
GC08	11	65729294	G	T	SART1	Missense	p.G15W	5	69	7.2%	.	.	.
GC08	11	66114905	C	A	B4GAT1	Missense	p.G38W	6	66	9.1%	.	.	.
GC08	11	73020376	-	CTC	ARHGEF17	Nonframeshift	p.C231delinsCL	33	88	37.5%	.	.	.
GC08	11	76924995	G	T	MYO7A	Missense	p.G2177W	6	99	6.1%	.	.	.
GC08	11	100999521	C	A	PGR	Missense	p.R94M	6	84	7.1%	.	.	.
GC08	11	108009695	C	A	ACAT1	Missense	p.P169Q	6	76	7.9%	.	.	.
GC08	11	108383419	G	T	EXPH5	Missense	p.H751N	8	136	5.9%	.	.	.
GC08	11	110485337	G	T	ARHGAP20	Missense	p.P170Q	8	128	6.3%	.	.	.
GC08	11	120979995	G	T	TECTA	Missense	p.G92W	7	98	7.1%	.	.	.
GC08	11	121476151	A	G	SORL1	Missense	p.S1607G	50	70	71.4%	.	.	.
GC08	11	128856468	C	A	ARHGAP32	Missense	p.R102L	5	58	8.6%	.	.	.
GC08	11	129739934	C	A	NFRKB	Missense	p.G1021W	6	77	7.8%	.	.	.
GC08	12	667726	G	T	B4GALNT3	Missense	p.G887V	6	87	6.9%	.	.	.
GC08	12	1940370	G	T	LRTM2	Missense	p.G113W	6	49	12.2%	.	.	.
GC08	12	6935873	G	T	GPR162	Missense	p.R140L	6	86	7.0%	.	.	.
GC08	12	7310247	G	T	CLSTN3	Missense	p.W897L	6	77	7.8%	.	.	.
GC08	12	14822658	C	A	GUCY2C	Missense	p.R427L	6	57	10.5%	.	.	.

GC08	12	14959571	C	A	SMCO3	Missense	p.R15L	6	80	7.5%	.	.	.
GC08	12	48538907	C	A	PFKM	Missense	p.R696S	6	66	9.1%	.	.	.
GC08	12	49391004	G	T	DDN	Missense	p.P552Q	6	74	8.1%	.	.	.
GC08	12	49959900	G	T	MCRS1	Missense	p.Q50K	5	46	10.9%	.	.	.
GC08	12	52451212	G	T	NR4A1	Missense	p.G480W	6	95	6.3%	.	O	.
GC08	12	53776164	G	T	SP1	Missense	p.G97W	7	94	7.4%	.	.	.
GC08	12	53880364	C	A	MAP3K12	Missense	p.W163L	5	36	13.9%	.	.	.
GC08	12	54576298	G	T	SMUG1	Missense	p.P30Q	8	91	8.8%	.	.	.
GC08	12	65564752	G	T	LEMD3	Missense	p.R459L	6	92	6.5%	.	.	.
GC08	12	72179410	C	A	RAB21	Missense	p.P212H	7	81	8.6%	.	.	.
GC08	12	96674618	G	T	CDK17	Missense	p.P501Q	7	137	5.1%	.	.	.
GC08	12	97311429	A	G	NEDD1	Missense	p.T95A	20	27	74.1%	.	.	.
GC08	12	102069145	C	A	MYBPC1	Missense	p.P911Q	5	46	10.9%	.	.	.
GC08	12	105280191	C	A	SLC41A2	Missense	p.G303C	6	85	7.1%	.	.	.
GC08	12	109935723	C	A	UBE3B	Missense	p.P272T	6	89	6.7%	.	.	.
GC08	12	112654709	C	A	HECTD4	Missense	p.W2284L	8	109	7.3%	.	.	.
GC08	12	113633582	C	A	IQCD	Missense	p.R281L	5	63	7.9%	.	O	.
GC08	12	121854110	C	A	RNF34	Missense	p.P52Q	6	84	7.1%	.	.	.
GC08	12	123805026	G	T	SBNO1	Missense	p.L874M	7	105	6.7%	.	.	.
GC08	12	124220151	C	A	ATP6V0A2	Missense	p.R269S	6	95	6.3%	.	O	.
GC08	12	129283878	G	T	SLC15A4	Nonsense	p.S500X	6	96	6.3%	.	.	.
GC08	13	20717069	G	T	GJA3	Missense	p.P120H	7	87	8.0%	.	.	.
GC08	13	20797126	C	A	GJB6	Missense	p.W165L	7	90	7.8%	.	.	.
GC08	13	26788818	G	T	RNF6	Missense	p.L401I	6	81	7.4%	.	.	.
GC08	13	29599716	C	A	MTUS2	Missense	p.P304Q	7	81	8.6%	.	.	.
GC08	13	32821530	C	A	FRY	Missense	p.P2300H	6	79	7.6%	.	.	.
GC08	13	32839663	C	A	FRY	Missense	p.A2619E	29	77	37.7%	.	.	.
GC08	13	46425646	G	T	SIAH3	Missense	p.P40H	6	47	12.8%	.	O	.
GC08	13	50115880	G	T	RCBTB1	Missense	p.P419Q	8	111	7.2%	.	.	.
GC08	13	52333880	G	T	WDFY2	Missense	p.W393L	6	56	10.7%	.	.	.
GC08	13	52345959	G	T	DHRS12	Missense	p.P186Q	6	59	10.2%	.	.	.

GC08	13	53035752	G	T	CKAP2	Missense	p.R265L	6	80	7.5%	.	.	.
GC08	13	99550442	C	A	DOCK9	Missense	p.W527L	6	86	7.0%	.	.	.
GC08	13	111099144	G	T	COL4A2	Splicing	c.1012-1G>T	7	80	8.8%	.	.	.
GC08	14	20502502	C	A	OR4K13	Missense	p.R139L	6	94	6.4%	.	O	.
GC08	14	23524543	C	A	CDH24	Missense	p.R74L	7	123	5.7%	.	.	.
GC08	14	23870120	G	T	MYH6	Missense	p.P403H	8	139	5.8%	.	.	.
GC08	14	31592140	G	T	HECTD1	Missense	p.Q1699K	9	137	6.6%	.	.	.
GC08	14	31790754	-	G	HEATR5A	Frameshift	p.P1307fs	126	177	71.2%	.	.	.
GC08	14	36004558	C	A	INSM2	Missense	p.P367Q	8	92	8.7%	.	.	.
GC08	14	37132327	G	T	PAX9	Missense	p.R77L	7	129	5.4%	.	.	.
GC08	14	67671667	C	A	FAM71D	Missense	p.P258Q	8	109	7.3%	.	.	.
GC08	14	75276436	G	T	YLPM1	Missense	p.M1625I	10	170	5.9%	.	.	.
GC08	14	75538351	G	T	ZC2HC1C	Missense	p.G359C	7	145	4.8%	.	.	.
GC08	14	81329115	G	T	CEP128	Missense	p.L250M	6	58	10.3%	.	.	.
GC08	14	91779528	G	T	CCDC88C	Missense	p.R878S	9	185	4.9%	.	.	.
GC08	14	92465687	G	T	TRIP11	Missense	p.R1596S	6	94	6.4%	.	.	.
GC08	14	94088213	C	A	UNC79	Missense	p.P1368H	9	132	6.8%	.	.	.
GC08	14	94545746	G	T	DDX24	Missense	p.Q115K	9	129	7.0%	.	.	.
GC08	14	102486291	G	T	DYNC1H1	Missense	p.W2802L	11	176	6.3%	.	.	.
GC08	14	102792974	A	G	ZNF839	Missense	p.K198R	15	41	36.6%	.	.	.
GC08	14	103173694	G	T	RCOR1	Missense	p.G169W	7	122	5.7%	.	.	.
GC08	14	104026526	G	T	BAG5	Missense	p.P326T	8	162	4.9%	.	.	.
GC08	14	104618785	C	A	KIF26A	Missense	p.P241Q	7	95	7.4%	.	.	.
GC08	15	22933864	C	G	CYFIP1	Missense	p.H139Q	5	35	14.3%	.	.	.
GC08	15	31619903	C	A	KLF13	Missense	p.P163Q	7	82	8.5%	.	.	.
GC08	15	42058546	T	C	MGA	Missense	p.Y2547H	41	99	41.4%	.	.	.
GC08	15	42145527	G	T	SPTBN5	Missense	p.R3367S	7	111	6.3%	.	.	.
GC08	15	42174582	G	T	SPTBN5	Missense	p.P711Q	6	72	8.3%	.	.	.
GC08	15	55611584	C	A	PIGB	Missense	p.Q46K	6	73	8.2%	.	.	.
GC08	15	56721376	C	-	MNS1	Frameshift	p.E471fs	30	65	46.2%	.	.	.
GC08	15	58467167	G	A	AQP9	Missense	p.A143T	25	79	31.6%	.	.	.

GC08	15	91795652	G	T	SV2B	Missense	p.R78L	6	65	9.2%	.	.	.
GC08	15	93545480	A	G	CHD2	Missense	p.Q1404R	41	111	36.9%	.	.	.
GC08	15	94910946	G	T	MCTP2	Missense	p.G60W	5	49	10.2%	.	.	.
GC08	15	98995080	G	T	FAM169B	Nonsense	p.S115X	7	85	8.2%	.	.	.
GC08	16	613515	G	T	PRR35	Missense	p.W74L	6	58	10.3%	.	.	.
GC08	16	716089	C	A	WDR90	Missense	p.P1525Q	6	89	6.7%	.	.	.
GC08	16	2287921	C	T	DNASE1L2	Missense	p.R249C	24	76	31.6%	.	.	.
GC08	16	3019757	G	T	PAQR4	Missense	p.G28W	6	83	7.2%	.	.	.
GC08	16	3642821	G	T	SLX4	Missense	p.Q736K	6	79	7.6%	.	.	.
GC08	16	8993615	C	A	USP7	Splicing	c.2136-1G>T;NM_001286457	7	58	12.1%	.	.	.
GC08	16	9934634	C	A	GRIN2A	Missense	p.M507I	6	86	7.0%	O	.	.
GC08	16	11815514	C	A	TXNDC11	Missense	p.G24W	5	67	7.5%	.	.	.
GC08	16	14312752	C	A	MKL2	Missense	p.P197Q	7	103	6.8%	.	.	.
GC08	16	28848089	G	T	ATXN2L	Missense	p.G1053W	6	72	8.3%	.	.	.
GC08	16	28935847	G	T	RABEP2	Missense	p.L51M	8	127	6.3%	.	.	.
GC08	16	28949129	G	T	CD19	Missense	p.R490L	6	73	8.2%	.	O	.
GC08	16	29819127	C	A	MAZ	Missense	p.H341N	7	88	8.0%	.	.	.
GC08	16	30977225	G	T	SETD1A	Missense	p.G675W	8	110	7.3%	.	.	.
GC08	16	31473965	G	T	ARMC5	Missense	p.R366L	7	72	9.7%	.	.	.
GC08	16	53348771	G	T	CHD9	Missense	p.G2467W	6	56	10.7%	.	.	.
GC08	16	72094244	G	T	HP	Missense	p.G167W	4	28	14.3%	.	.	.
GC08	16	72153752	G	T	PMFBP1	Missense	p.P1002Q	6	73	8.2%	.	.	.
GC08	16	75498402	G	T	TMEM170A	Missense	p.L33M	6	82	7.3%	.	.	.
GC08	16	75669634	G	T	KARS	Missense	p.R247S	6	56	10.7%	.	.	.
GC08	16	77325290	G	T	ADAMTS18	Missense	p.P920Q	7	95	7.4%	.	O	.
GC08	16	81979806	G	T	PLCG2	Missense	p.G1170W	7	99	7.1%	.	O	.
GC08	16	84203606	C	A	DNAAF1	Missense	p.P155Q	7	96	7.3%	.	.	.
GC08	16	84520599	C	A	TLDC1	Missense	p.W199L	6	69	8.7%	.	.	.
GC08	17	1382964	C	A	MYO1C	Missense	p.L314F	6	70	8.6%	.	.	.
GC08	17	2236379	G	T	TSR1	Missense	p.P394H	7	122	5.7%	.	.	.
GC08	17	2270585	C	A	SGSM2	Missense	p.L437I	6	58	10.3%	.	.	.

GC08	17	3665261	G	T	ITGAE	Missense	p.P88H	5	40	12.5%	.	.	.
GC08	17	4013094	C	A	ZZEF1	Missense	p.R377M	9	161	5.6%	.	.	.
GC08	17	4192613	G	T	UBE2G1	Missense	p.P113H	7	119	5.9%	.	.	.
GC08	17	7577552	C	A	TP53	Missense	p.M111I	5	44	11.4%	O	.	.
GC08	17	8157565	G	T	PFAS	Missense	p.R75L	6	78	7.7%	.	.	.
GC08	17	8161140	G	T	PFAS	Missense	p.W364L	6	45	13.3%	.	.	.
GC08	17	11572852	G	T	DNAH9	Missense	p.G1032W	6	86	7.0%	.	.	.
GC08	17	17881054	C	A	DRC3	Missense	p.L48M	4	27	14.8%	.	.	.
GC08	17	18154277	C	A	FLII	Missense	p.G496W	5	37	13.5%	.	O	.
GC08	17	21318928	C	T	KCNJ12	Missense	p.L92F	10	160	6.3%	.	O	.
GC08	17	26657504	G	T	IFT20	Missense	p.Q60K	6	91	6.6%	.	.	.
GC08	17	35310470	C	A	AATF	Missense	p.Q190K	7	87	8.0%	.	.	.
GC08	17	36104758	C	A	HNF1B	Missense	p.G40W	8	85	9.4%	.	.	.
GC08	17	37665959	G	T	CDK12	Missense	p.G871W	6	87	6.9%	O	.	.
GC08	17	39459036	G	T	KRTAP29-1	Missense	p.P23Q	8	109	7.3%	.	.	.
GC08	17	39890606	C	A	HAP1	Missense	p.R94L	7	107	6.5%	.	.	.
GC08	17	40762174	C	A	TUBG1	Missense	p.P33H	8	115	7.0%	.	.	.
GC08	17	41346446	C	A	NBR1	Missense	p.P514Q	6	65	9.2%	.	.	.
GC08	17	42390802	G	T	RUNDC3A	Missense	p.R130L	7	124	5.6%	.	.	.
GC08	17	45752067	C	A	KPNB1	Missense	p.L466M	7	79	8.9%	.	.	.
GC08	17	45820458	C	A	TBX21	Missense	p.P223Q	6	95	6.3%	.	.	.
GC08	17	58711230	G	T	PPM1D	Missense	p.G240W	9	106	8.5%	O	.	.
GC08	17	59067503	G	C	BCAS3	Missense	p.E465Q	40	111	36.0%	.	.	.
GC08	17	60526038	G	T	METTL2A	Missense	p.R362L	8	94	8.5%	.	O	.
GC08	17	61492944	C	A	TANC2	Missense	p.P1275H	6	73	8.2%	.	.	.
GC08	17	62290172	G	T	TEX2	Missense	p.P469Q	7	121	5.8%	.	.	.
GC08	17	71380041	G	T	SDK2	Missense	p.P1560Q	7	86	8.1%	.	.	.
GC08	17	72287236	C	A	DNAI2	Missense	p.H230N	7	123	5.7%	.	.	.
GC08	17	72916207	G	T	USH1G	Missense	p.R139S	11	159	6.9%	.	.	.
GC08	17	73919579	G	T	FBF1	Missense	p.P371H	7	70	10.0%	.	.	.
GC08	17	74003588	G	T	EVPL	Missense	p.Q1922K	8	119	6.7%	.	.	.

GC08	17	78305952	G	T	RNF213	Missense	p.G1222W	6	54	11.1%	.	.	.
GC08	17	79220048	G	T	SLC38A10	Missense	p.Q890K	7	86	8.1%	.	.	.
GC08	18	18619508	G	A	ROCK1	Nonsense	p.R326X	39	132	29.5%	.	.	.
GC08	18	19423091	G	T	MIB1	Splicing	c.1963-1G>T	6	88	6.8%	.	.	.
GC08	18	48513195	C	A	ELAC1	Missense	p.L278M	10	114	8.8%	.	.	.
GC08	18	59157854	G	T	CDH20	Missense	p.W23L	8	87	9.2%	.	.	.
GC08	18	61652399	C	A	SERPINB8	Missense	p.Q34K	6	88	6.8%	.	.	.
GC08	18	77171178	G	T	NFATC1	Missense	p.L301F	7	81	8.6%	.	.	.
GC08	19	1043419	G	T	ABCA7	Missense	p.G293W	5	63	7.9%	.	.	.
GC08	19	9062458	G	T	MUC16	Missense	p.H8330N	7	76	9.2%	.	.	.
GC08	19	9069073	C	G	MUC16	Missense	p.A6125P	4	73	5.5%	.	.	.
GC08	19	9086846	G	T	MUC16	Missense	p.P1657T	5	49	10.2%	.	.	.
GC08	19	11024599	C	A	CARM1	Missense	p.P239Q	5	39	12.8%	.	.	.
GC08	19	11258698	G	T	SPC24	Missense	p.P134H	7	72	9.7%	.	.	.
GC08	19	12430478	G	T	ZNF563	Missense	p.H121N	6	75	8.0%	.	.	.
GC08	19	12460694	T	C	ZNF442	Missense	p.K569E	5	38	13.2%	.	.	.
GC08	19	16060286	G	T	OR10H4	Missense	p.G157W	6	80	7.5%	.	.	.
GC08	19	33602687	G	T	GPATCH1	Missense	p.R548L	4	22	18.2%	.	O	.
GC08	19	38126079	G	T	ZFP30	Missense	p.Q455K	6	76	7.9%	.	.	.
GC08	19	38126616	CA	-	ZFP30	Frameshift	p.C275fs	67	107	62.6%	.	.	.
GC08	19	41928968	G	T	BCKDHA	Missense	p.W354L	6	86	7.0%	.	.	.
GC08	19	48229209	C	A	EHD2	Missense	p.R215S	6	100	6.0%	.	.	.
GC08	19	56052822	C	A	SBK3	Missense	p.R157L	6	64	9.4%	.	.	.
GC08	19	56056865	C	A	SBK3	Missense	p.E15D	6	81	7.4%	.	.	.
GC08	19	56244113	G	T	NLRP9	Missense	p.Q362K	7	99	7.1%	.	.	.
GC08	19	57910020	C	A	ZNF548	Missense	p.P122Q	8	93	8.6%	.	.	.
GC08	19	58059301	C	A	ZNF550	Missense	p.R104L	6	78	7.7%	.	.	.
GC08	19	58500050	G	T	ZNF606	Missense	p.Q73K	6	76	7.9%	.	.	.
GC08	20	398416	G	T	RBCK1	Missense	p.W59L	7	129	5.4%	.	.	.
GC08	20	2776413	G	T	CPXM1	Missense	p.R518S	9	121	7.4%	.	O	.
GC08	20	3102750	G	T	UBOX5	Missense	p.H179N	6	78	7.7%	.	.	.

GC08	20	3687732	G	T	SIGLEC1	Missense	p.P15Q	6	69	8.7%	.	.	.
GC08	20	3729882	G	T	HSPA12B	Missense	p.R199L	6	80	7.5%	.	.	.
GC08	20	10030572	C	A	ANKEF1	Missense	p.P452Q	6	95	6.3%	.	.	.
GC08	20	30354436	C	A	TPX2	Missense	p.L103M	7	105	6.7%	.	.	.
GC08	20	32212767	G	T	CBFA2T2	Missense	p.R297L	7	128	5.5%	.	.	.
GC08	20	33867379	G	T	EIF6	Missense	p.L221I	6	93	6.5%	.	.	.
GC08	20	34025372	G	T	GDF5	Missense	p.Q113K	6	68	8.8%	.	.	.
GC08	20	34220447	G	T	CPNE1	Missense	p.L101I	8	120	6.7%	.	.	.
GC08	20	44643075	G	T	MMP9	Missense	p.G656V	6	98	6.1%	.	.	.
GC08	20	46294624	G	T	SULF2	Missense	p.H627N	8	82	9.8%	.	.	.
GC08	20	49575017	C	A	DPM1	Missense	p.R15L	8	126	6.3%	.	.	.
GC08	20	50769424	C	A	ZFP64	Missense	p.R382L	7	124	5.6%	.	.	.
GC08	20	57572737	G	T	CTSZ	Missense	p.L187I	6	62	9.7%	.	.	.
GC08	20	61834156	G	T	YTHDF1	Missense	p.P379Q	7	116	6.0%	.	.	.
GC08	20	61907521	G	T	ARFGAP1	Missense	p.G47W	6	93	6.5%	.	.	.
GC08	20	61981274	C	T	CHRNA4	Missense	p.D497N	66	83	79.5%	.	.	.
GC08	20	62196189	C	A	HELZ2	Missense	p.R760L	7	129	5.4%	.	.	.
GC08	20	62221576	G	T	GMEB2	Missense	p.L487M	6	87	6.9%	.	.	.
GC08	21	34132200	C	A	PAXBP1	Nonsense	p.G361X	5	44	11.4%	.	.	.
GC08	21	35166768	C	A	ITSN1	Missense	p.Q650K	7	68	10.3%	.	.	.
GC08	21	43983963	C	A	SLC37A1	Missense	p.L371M	7	78	9.0%	.	.	.
GC08	21	45750761	C	A	C21orf2	Missense	p.R195L	6	44	13.6%	.	.	.
GC08	22	17280789	C	A	XKR3	Missense	p.R154L	7	134	5.2%	.	.	.
GC08	22	18387503	G	T	MICAL3	Missense	p.R123S	5	59	8.5%	.	.	.
GC08	22	19496157	C	A	CDC45	Missense	p.P341H	6	91	6.6%	.	.	.
GC08	22	20040061	C	A	TANGO2	Missense	p.H14N	5	37	13.5%	.	.	.
GC08	22	20760138	G	T	ZNF74	Missense	p.R201L	5	72	6.9%	.	.	.
GC08	22	24718104	G	T	SPECC1L	Missense	p.G386W	6	87	6.9%	.	.	.
GC08	22	26692953	G	T	SEZ6L	Missense	p.G357W	5	50	10.0%	.	.	.
GC08	22	26864527	G	T	HPS4	Missense	p.P215H	5	32	15.6%	.	.	.
GC08	22	29454803	G	T	C22orf31	Missense	p.P267H	7	111	6.3%	.	.	.

GC08	22	29704337	C	A	GAS2L1	Missense	p.P81Q	6	68	8.8%	.	.	.
GC08	22	30198131	G	T	ASCC2	Missense	p.Q398K	7	64	10.9%	.	.	.
GC08	22	32110034	G	T	PRR14L	Missense	p.P1264Q	6	69	8.7%	.	.	.
GC08	22	36556838	C	A	APOL3	Missense	p.Q34H	6	88	6.8%	.	.	.
GC08	22	36680293	G	T	MYH9	Missense	p.R1871S	7	113	6.2%	.	.	.
GC08	22	37209713	C	A	PVALB	Missense	p.G94V	7	114	6.1%	.	.	.
GC08	22	38134698	C	A	TRIOBP	Missense	p.P1719H	6	85	7.1%	.	.	.
GC08	22	38483156	-	TCATGGGTG	BAIAP2L2	Nonsense	p.N412delinsHPX	16	37	43.2%	.	.	.
GC08	22	38509610	C	A	PLA2G6	Missense	p.G642W	6	69	8.7%	.	.	.
GC08	22	41513719	C	A	EP300	Missense	p.P208Q	6	88	6.8%	O	.	.
GC08	22	42609430	G	T	TCF20	Missense	p.Q628K	8	101	7.9%	.	.	.
GC08	22	50515289	C	A	MLC1	Missense	p.R193L	5	57	8.8%	.	.	.
GC08	X	7268021	G	T	STS	Missense	p.G491W	6	90	6.7%	.	.	.
GC08	X	9859097	C	A	SHROOM2	Missense	p.P133Q	5	50	10.0%	.	.	.
GC08	X	34149012	G	T	FAM47A	Missense	p.P462T	5	44	11.4%	.	.	.
GC08	X	48418231	G	T	TBC1D25	Missense	p.R328L	6	81	7.4%	.	.	.
GC08	X	49074227	G	T	CACNA1F	Missense	p.P1026T	5	45	11.1%	.	.	.
GC08	X	70643007	C	A	TAF1	Missense	p.P1518H	6	51	11.8%	.	.	.
GC08	X	86773036	C	A	KLHL4	Missense	p.P47Q	4	27	14.8%	.	.	.
GC08	X	100667516	C	G	HNRNPH2	Nonsense	p.Y180X	4	59	6.8%	.	.	.
GC08	X	114426618	C	A	RBMXL3	Missense	p.H872N	6	90	6.7%	.	.	.
GC08	X	147891443	G	T	AFF2	Missense	p.R32L	6	89	6.7%	.	.	.
GC09	1	8424198	G	A	RERE	Missense	p.P553L	9	21	42.9%	.	.	.
GC09	1	19518811	A	G	UBR4	Missense	p.L422P	40	61	65.6%	.	.	.
GC09	2	169727976	C	T	SPC25	Missense	p.V214I	70	106	66.0%	.	.	.
GC09	2	201477450	T	C	AOX1	Missense	p.I461T	27	37	73.0%	.	.	.
GC09	3	20042894	T	-	PP2D1	Nonsense	p.M240X	99	152	65.1%	.	.	.
GC09	4	265975	G	T	ZNF732	Missense	p.T223K	6	53	11.3%	.	O	.
GC09	4	266059	G	T	ZNF732	Missense	p.T195K	7	62	11.3%	.	O	.
GC09	4	435773	C	T	ZNF721	Missense	p.R828K	9	198	4.5%	.	O	.
GC09	4	69973946	A	G	UGT2B7	Missense	p.M406V	76	106	71.7%	.	.	.

GC09	4	110932390	-	C	EGF	Frameshift	p.P1094fs	22	50	44.0%	.	.	.
GC09	5	96430508	T	G	LIX1	Missense	p.T265P	10	21	47.6%	.	.	.
GC09	5	178359606	C	T	ZFP2	Missense	p.T431I	5	56	8.9%	.	.	.
GC09	6	138539184	-	G	PBOV1	Frameshift	p.L117fs	134	197	68.0%	.	.	.
GC09	7	63808939	G	A	ZNF736	Missense	p.G233E	7	110	6.4%	.	O	.
GC09	8	22972239	C	A	TNFRSF10C	Missense	p.S79Y	19	30	63.3%	.	.	.
GC09	8	142161937	-	GTTA	DENND3	Splicing	c.834+1->GTTA	21	43	48.8%	.	.	.
GC09	9	103082617	G	A	TEX10	Missense	p.T714I	22	33	66.7%	.	.	.
GC09	10	27702257	-	C	PTCHD3	Frameshift	p.G308fs	24	48	50.0%	.	.	.
GC09	10	64574003	G	T	EGR2	Missense	p.A132D	4	23	17.4%	.	.	.
GC09	11	10215455	A	C	SBF2	Missense	p.I45M	31	45	68.9%	.	.	.
GC09	11	49221893	C	T	FOLH1	Missense	p.E109K	52	82	63.4%	.	.	.
GC09	12	50484057	C	T	SMARCD1	Nonsense	p.R303X	33	47	70.2%	O	O	.
GC09	12	51873930	G	A	SLC4A8	Nonsense	p.W723X	6	43	14.0%	.	.	.
GC09	12	53431384	A	C	EIF4B	Missense	p.T461P	4	49	8.2%	.	.	.
GC09	12	75900660	T	C	KRR1	Missense	p.M99V	22	30	73.3%	.	.	.
GC09	12	82796855	A	-	METTL25	Frameshift	p.S322fs	29	47	61.7%	.	.	.
GC09	12	109316526	C	T	SVOP	Missense	p.E313K	28	41	68.3%	.	.	.
GC09	14	24470691	-	A	DHRS4L2	Frameshift	p.L68fs	21	52	40.4%	.	.	.
GC09	15	56387083	G	T	RFX7	Missense	p.P948H	5	47	10.6%	.	O	.
GC09	15	59377880	G	A	RNF111	Missense	p.V825M	31	44	70.5%	.	.	.
GC09	15	99511805	-	C	PGPEP1L	Frameshift	p.V165fs	27	40	67.5%	.	.	.
GC09	16	22546267	G	A	NPIPB5	Missense	p.V655I	161	444	36.3%	.	.	.
GC09	16	23632686	A	G	PALB2	Missense	p.I1037T	25	35	71.4%	O	.	O
GC09	16	68941449	G	T	TANGO6	Missense	p.A591S	25	33	75.8%	.	.	.
GC09	17	20320806	C	T	FAM106B	Missense	p.T15I	71	109	65.1%	.	.	.
GC09	17	60813602	C	T	MARCH10	Missense	p.E543K	32	53	60.4%	.	O	.
GC09	19	9361802	C	G	OR7E24	Nonsense	p.S28X	6	110	5.5%	.	O	.
GC09	19	9361877	A	G	OR7E24	Missense	p.Y53C	8	193	4.1%	.	O	.
GC09	19	9677096	G	C	ZNF121	Missense	p.N231K	5	57	8.8%	.	.	.
GC09	19	23543328	G	C	ZNF91	Missense	p.T786R	10	248	4.0%	.	O	.

GC09	19	24115619	A	G	ZNF726	Missense	p.Y234C	6	81	7.4%	.	.	.
GC09	19	50285249	A	C	AP2A1	Missense	p.K61T	42	130	32.3%	.	.	.
GC09	19	52919678	C	G	ZNF528	Missense	p.Q525E	4	32	12.5%	.	O	.
GC09	19	53410359	T	C	ZNF888	Missense	p.R406G	5	62	8.1%	.	.	.
GC09	19	53884886	A	G	ZNF525	Missense	p.K352E	6	135	4.4%	.	.	.
GC09	20	47775475	-	C	STAU1	Splicing	c.21+1->G;NM_001322931	26	54	48.1%	.	.	.
GC09	X	47272203	G	A	ZNF157	Missense	p.R244K	6	47	12.8%	.	.	.
GC09	X	62570146	A	G	SPIN4	Missense	p.Y185H	27	47	57.4%	.	.	.
GC10	1	64672515	A	T	UBE2U	Missense	p.I73L	4	45	8.9%	.	.	.
GC10	1	120483281	T	C	NOTCH2	Missense	p.N1027S	7	70	10.0%	O	.	.
GC10	1	226074687	A	G	LEFTY1	Missense	p.W281R	4	26	15.4%	.	.	.
GC10	1	241519052	G	C	RGS7	Missense	p.Q9E	5	84	6.0%	.	.	.
GC10	2	107429627	C	G	ST6GAL2	Missense	p.D458H	4	45	8.9%	.	O	.
GC10	3	113252027	C	A	SIDT1	Missense	p.S53R	10	77	13.0%	.	.	.
GC10	4	6086694	T	C	JAKMIP1	Splicing	c.835-2A>G;NM_144720	5	41	12.2%	.	.	.
GC10	4	57181760	A	G	KIAA1211	Missense	p.T698A	6	66	9.1%	.	.	.
GC10	4	71698904	C	T	GRSF1	Missense	p.E39K	9	90	10.0%	.	.	.
GC10	4	75041107	G	C	MTHFD2L	Missense	p.Q146H	13	67	19.4%	.	.	.
GC10	4	185689561	C	G	ACSL1	Missense	p.G312A	6	134	4.5%	.	.	.
GC10	5	137520357	G	T	KIF20A	Missense	p.G559C	6	76	7.9%	.	.	.
GC10	5	137903154	C	G	HSPA9	Missense	p.E236Q	4	32	12.5%	.	.	.
GC10	6	16307048	C	A	ATXN1	Nonsense	p.G654X	5	60	8.3%	.	.	.
GC10	6	17652073	T	C	NUP153	Missense	p.R494G	5	89	5.6%	.	O	.
GC10	6	152599342	A	T	SYNE1	Missense	p.L6081Q	20	91	22.0%	.	.	.
GC10	7	29111976	C	T	CPVL	Missense	p.E225K	5	78	6.4%	.	.	.
GC10	7	66410024	C	T	TMEM248	Missense	p.T74I	19	147	12.9%	.	.	.
GC10	7	82579457	C	G	PCLO	Missense	p.D3483H	23	111	20.7%	.	.	.
GC10	7	121942381	G	C	FEZF1	Missense	p.H366Q	5	92	5.4%	.	.	.
GC10	7	148311267	G	A	C7orf33	Missense	p.R113K	14	101	13.9%	.	.	.
GC10	9	135251340	A	G	TTF1	Missense	p.S379P	5	69	7.2%	.	O	.

GC10	10	50667088	G	C	ERCC6	Missense	p.L1419V	15	89	16.9%	.	.	.
GC10	10	134036246	C	A	STK32C	Nonsense	p.E397X	4	47	8.5%	.	.	.
GC10	11	46701812	A	T	ARHGAP1	Missense	p.F281I	5	86	5.8%	.	.	.
GC10	11	123848187	G	T	OR10S1	Missense	p.P71H	14	68	20.6%	.	.	.
GC10	12	45833519	G	A	ANO6	Missense	p.R863H	5	62	8.1%	.	O	.
GC10	12	49718038	A	C	TROAP	Missense	p.H121P	4	41	9.8%	.	.	.
GC10	12	112148171	T	C	ACAD10	Splicing	c.783+2T>C	10	115	8.7%	.	.	.
GC10	13	39263795	A	G	FREM2	Missense	p.T772A	6	90	6.7%	.	.	.
GC10	14	24662279	A	G	TM9SF1	Missense	p.V181A	16	109	14.7%	.	.	.
GC10	14	50101632	C	A	DNAAF2	Missense	p.R79L	5	54	9.3%	.	.	.
GC10	14	99641571	G	C	BCL11B	Missense	p.D462E	5	81	6.2%	.	.	.
GC10	15	42138944	A	T	PLA2G4B	Missense	p.I620F	5	73	6.8%	.	.	.
GC10	15	66386766	C	G	MEGF11	Missense	p.G123A	5	66	7.6%	.	.	.
GC10	16	67976346	T	C	LCAT	Missense	p.K223R	4	45	8.9%	.	.	.
GC10	16	85952122	C	G	IRF8	Missense	p.P234R	6	45	13.3%	.	.	.
GC10	17	47376113	T	G	ZNF652	Missense	p.T495P	6	92	6.5%	.	.	.
GC10	17	78188083	C	T	SGSH	Missense	p.G184E	5	56	8.9%	.	.	.
GC10	17	80399015	G	C	HEXDC	Missense	p.Q375H	12	103	11.7%	.	.	.
GC10	18	53298559	T	C	TCF4	Missense	p.T86A	5	74	6.8%	.	O	.
GC10	18	56402537	G	C	MALT1	Missense	p.V516L	11	58	19.0%	.	.	.
GC10	19	10463753	G	C	TYK2	Missense	p.Q1017E	12	57	21.1%	.	.	.
GC10	19	17373717	G	C	USHBP1	Missense	p.H32D	6	44	13.6%	.	O	.
GC10	19	46299164	C	T	RSPH6A	Missense	p.G706D	5	67	7.5%	.	.	.
GC10	20	36151075	A	T	NNAT	Missense	p.R27W	7	66	10.6%	.	O	.
GC10	20	45174048	A	C	OCSTAMP	Missense	p.V322G	5	58	8.6%	.	O	.
GC10	20	56227315	C	G	PMEPA1	Missense	p.G192R	6	32	18.8%	.	.	.
GC10	X	15534275	T	G	BMX	Missense	p.S122R	11	68	16.2%	.	.	.
GC10	X	24191735	T	C	ZFX	Missense	p.F24L	6	98	6.1%	.	O	.
GC10	X	53595763	C	A	HUWE1	Missense	p.S2199I	5	83	6.0%	.	.	.
GC10	X	140995132	T	C	MAGEC1	Missense	p.S648P	8	130	6.2%	.	O	.
GC10	X	140995157	T	C	MAGEC1	Missense	p.V656A	6	114	5.3%	.	.	.

GC11	1	43021962	G	C	CCDC30	Missense	p.Q187H	47	58	81.0%	.	.	.
GC11	1	152732285	G	T	KPRP	Missense	p.C74F	4	45	8.9%	.	.	.
GC11	2	133541398	G	T	NCKAP5	Missense	p.P996T	5	59	8.5%	.	.	.
GC11	2	135107399	C	T	MGAT5	Missense	p.S379L	9	82	11.0%	.	O	.
GC11	2	153533079	C	T	PRPF40A	Missense	p.V291I	40	49	81.6%	.	.	.
GC11	2	231077703	A	G	SP110	Missense	p.I119T	24	33	72.7%	.	.	.
GC11	2	236761415	-	CAGG	AGAP1	Frameshift	p.P379fs	19	30	63.3%	.	.	.
GC11	3	195505819	A	T	MUC4	Missense	p.V4211D	15	289	5.2%	.	O	.
GC11	4	83788032	G	C	SEC31A	Missense	p.Q371E	95	129	73.6%	.	.	.
GC11	4	110932390	-	C	EGF	Frameshift	p.P1094fs	51	70	72.9%	.	.	.
GC11	5	34925070	C	T	BRIX1	Missense	p.S261L	21	33	63.6%	.	.	.
GC11	5	136315055	C	A	SPOCK1	Missense	p.L365F	9	55	16.4%	.	.	.
GC11	5	140182967	G	T	PCDHA3	Nonsense	p.E729X	21	26	80.8%	.	.	.
GC11	6	137815225	C	T	OLIG3	Missense	p.R28H	7	51	13.7%	.	O	.
GC11	6	160174524	G	C	WTAP	Missense	p.R162P	7	53	13.2%	.	O	.
GC11	7	12391269	-	A	VWDE	Frameshift	p.N1157fs	36	55	65.5%	.	.	.
GC11	7	36375822	T	G	KIAA0895	Missense	p.K25Q	119	163	73.0%	.	.	.
GC11	7	63726817	C	A	ZNF679	Missense	p.T269K	9	168	5.4%	.	O	.
GC11	7	64169017	-	GAA	ZNF107	Nonframeshift	p.E816delinsGK	72	100	72.0%	.	.	.
GC11	7	64292444	A	G	ZNF138	Missense	p.E128G	6	114	5.3%	.	O	.
GC11	8	105361542	T	G	DCSTAMP	Missense	p.F254L	32	42	76.2%	.	.	.
GC11	8	145722779	C	T	PPP1R16A	Missense	p.L68F	20	23	87.0%	.	.	.
GC11	10	27702257	-	C	PTCHD3	Frameshift	p.G308fs	33	53	62.3%	.	.	.
GC11	11	89819889	-	T	UBTFL1	Frameshift	p.W258fs	26	74	35.1%	.	.	.
GC11	12	102131630	C	G	SYCP3	Missense	p.E28D	77	101	76.2%	.	.	.
GC11	14	78023457	C	A	SPTLC2	Missense	p.A295S	10	49	20.4%	.	.	.
GC11	14	92959835	C	G	SLC24A4	Missense	p.L578V	20	29	69.0%	.	.	.
GC11	14	105419446	T	G	AHNAK2	Missense	p.K681T	22	154	14.3%	.	O	.
GC11	16	5134779	C	A	ALG1	Missense	p.A320E	5	43	11.6%	.	O	.
GC11	16	15808810	G	C	MYH11	Missense	p.N1914K	38	55	69.1%	.	.	.
GC11	17	8018944	C	-	ALOXE3	Frameshift	p.A271fs	27	52	51.9%	.	.	.

GC11	18	28993550	C	A	DSG4	Missense	p.Q1058K	29	42	69.0%	.	.	.
GC11	18	29793436	C	T	MEP1B	Missense	p.T498I	82	115	71.3%	.	.	.
GC11	18	48443769	T	G	ME2	Missense	p.V174G	75	97	77.3%	.	.	.
GC11	19	1802063	T	C	ATP8B3	Splicing	c.905-2A>G	5	43	11.6%	.	O	.
GC11	19	12384502	C	A	ZNF44	Missense	p.V190L	88	115	76.5%	.	.	.
GC11	19	21720557	A	G	ZNF429	Missense	p.K568E	8	174	4.6%	O	O	.
GC11	19	43762496	-	AC	PSG9	Frameshift	p.T181fs	188	278	67.6%	.	.	.
GC11	19	54080575	G	C	ZNF331	Missense	p.R254P	6	99	6.1%	.	.	.
GC11	19	55525689	A	C	GP6	Missense	p.C542G	6	103	5.8%	.	O	.
GC11	X	50659211	-	TCT	BMP15	Nonframeshift	p.S261delinsS S	14	28	50.0%	.	.	.
GC11	X	107978565	C	T	IRS4	Missense	p.R337K	10	100	10.0%	.	.	.
GC13	1	949767	C	A	ISG15	Missense	p.P136Q	6	74	8.1%	.	.	.
GC13	1	985333	G	T	AGRN	Missense	p.G1599W	6	35	17.1%	.	.	.
GC13	1	1231610	C	A	ACAP3	Missense	p.W463L	6	66	9.1%	.	.	.
GC13	1	1275137	C	A	DVL1	Missense	p.R322L	6	77	7.8%	.	.	.
GC13	1	1322772	G	T	CCNL2	Missense	p.R246S	10	112	8.9%	.	.	.
GC13	1	1421506	G	T	ATAD3B	Missense	p.R281L	6	100	6.0%	.	.	.
GC13	1	2005703	C	A	PRKCZ	Missense	p.P4Q	6	41	14.6%	.	.	.
GC13	1	2938864	C	A	ACTRT2	Missense	p.P205H	6	64	9.4%	.	.	.
GC13	1	3342257	G	T	PRDM16	Missense	p.G1018W	8	79	10.1%	.	.	.
GC13	1	3669101	G	T	CCDC27	Missense	p.R19L	6	100	6.0%	.	.	.
GC13	1	3765202	G	T	CEP104	Missense	p.P86H	9	128	7.0%	.	.	.
GC13	1	5965352	C	A	NPHP4	Missense	p.R140I	8	108	7.4%	.	O	.
GC13	1	6158595	G	T	KCNAB2	Missense	p.L288F	8	81	9.9%	.	.	.
GC13	1	6194861	C	A	CHD5	Missense	p.G977W	7	66	10.6%	.	.	.
GC13	1	6202604	G	T	CHD5	Missense	p.P702Q	6	57	10.5%	.	.	.
GC13	1	6534102	G	T	PLEKHG5	Missense	p.Q188K	6	46	13.0%	.	.	.
GC13	1	6648370	G	T	ZBTB48	Missense	p.G517W	8	91	8.8%	.	.	.
GC13	1	6661916	G	T	KLHL21	Missense	p.P321Q	8	70	11.4%	.	.	.
GC13	1	7807783	G	T	CAMTA1	Missense	p.R587L	6	93	6.5%	.	.	.
GC13	1	7844944	C	A	PER3	Missense	p.R3S	7	66	10.6%	.	.	.

GC13	1	7869983	G	T	PER3	Missense	p.G425W	6	83	7.2%	.	.	.
GC13	1	7890118	G	T	PER3	Missense	p.M1037I	8	129	6.2%	.	.	.
GC13	1	7895878	C	A	PER3	Missense	p.Q1091K	6	56	10.7%	.	.	.
GC13	1	8075639	G	T	ERRFI1	Missense	p.P14Q	7	96	7.3%	.	.	.
GC13	1	8384718	C	A	SLC45A1	Missense	p.P144Q	5	58	8.6%	.	.	.
GC13	1	8418675	C	A	RERE	Missense	p.R753L	5	65	7.7%	.	.	.
GC13	1	9083080	C	A	SLC2A7	Missense	p.G70W	5	40	12.5%	.	.	.
GC13	1	9305394	G	T	H6PD	Missense	p.R145L	8	74	10.8%	.	.	.
GC13	1	9305499	G	T	H6PD	Missense	p.R180L	7	111	6.3%	.	.	.
GC13	1	9776093	C	A	PIK3CD	Missense	p.P186Q	5	57	8.8%	.	.	.
GC13	1	9777093	G	T	PIK3CD	Missense	p.R286L	6	47	12.8%	.	.	.
GC13	1	9780906	C	A	PIK3CD	Missense	p.P514Q	6	51	11.8%	.	.	.
GC13	1	10363351	C	A	KIF1B	Missense	p.P703Q	8	110	7.3%	.	.	.
GC13	1	10527366	G	T	DFFA	Missense	p.Q108K	8	97	8.2%	.	.	.
GC13	1	11090247	G	T	MASP2	Missense	p.P428Q	7	83	8.4%	.	.	.
GC13	1	11118912	C	A	SRM	Missense	p.R109L	5	65	7.7%	.	.	.
GC13	1	11158174	C	A	EXOSC10	Missense	p.G51W	7	78	9.0%	.	.	.
GC13	1	11182094	C	A	MTOR	Missense	p.R2251L	5	47	10.6%	O	.	.
GC13	1	11561609	G	T	DISP3	Missense	p.R187L	7	71	9.9%	.	.	.
GC13	1	11589653	G	T	DISP3	Missense	p.G947W	6	71	8.5%	.	.	.
GC13	1	11594490	G	T	DISP3	Missense	p.W1143L	6	81	7.4%	.	.	.
GC13	1	11718815	G	T	FBXO44	Missense	p.R129L	6	58	10.3%	.	.	.
GC13	1	11850892	G	T	MTHFR	Missense	p.R647S	7	74	9.5%	.	.	.
GC13	1	11852352	G	T	MTHFR	Missense	p.H580N	6	60	10.0%	.	.	.
GC13	1	11862990	G	T	MTHFR	Missense	p.L103M	7	93	7.5%	.	.	.
GC13	1	11866389	C	A	CLCN6	Missense	p.R24S	5	49	10.2%	.	.	.
GC13	1	12010512	C	A	PLOD1	Missense	p.P134Q	5	27	18.5%	.	.	.
GC13	1	12056305	G	T	MFN2	Missense	p.R135L	7	60	11.7%	.	.	.
GC13	1	12062051	C	A	MFN2	Missense	p.Q351K	7	96	7.3%	.	.	.
GC13	1	12198419	C	A	TNFRSF8	Missense	p.P378Q	4	32	12.5%	.	.	.
GC13	1	12266906	G	T	TNFRSF1B	Missense	p.M405I	9	88	10.2%	.	.	.

GC13	1	12318083	C	A	VPS13D	Missense	p.R345S	8	108	7.4%	.	.	.
GC13	1	12351084	G	T	VPS13D	Missense	p.G1919W	7	79	8.9%	.	.	.
GC13	1	12704733	G	T	AADACL4	Missense	p.L56F	8	85	9.4%	.	.	.
GC13	1	12711256	G	T	AADACL4	Missense	p.G95W	8	99	8.1%	.	.	.
GC13	1	14107969	G	T	PRDM2	Missense	p.G1026W	6	94	6.4%	.	.	.
GC13	1	14109055	G	T	PRDM2	Missense	p.G1388W	7	84	8.3%	.	.	.
GC13	1	15545982	G	T	TMEM51	Nonsense	p.E169X	13	58	22.4%	.	O	.
GC13	1	15890794	G	T	DNAJC16	Missense	p.W201L	9	129	7.0%	.	.	.
GC13	1	15894650	C	A	DNAJC16	Missense	p.P464Q	7	82	8.5%	.	.	.
GC13	1	15900208	G	T	AGMAT	Missense	p.L333M	9	91	9.9%	.	.	.
GC13	1	15988158	G	T	RSC1A1	Missense	p.G599W	8	106	7.5%	.	.	.
GC13	1	16202969	G	T	SPEN	Missense	p.R226L	6	79	7.6%	O	O	.
GC13	1	16254768	G	T	SPEN	Missense	p.R678L	6	93	6.5%	O	.	.
GC13	1	16262020	G	T	SPEN	Missense	p.Q3095H	7	90	7.8%	O	.	.
GC13	1	16264425	G	T	SPEN	Missense	p.G3543V	6	57	10.5%	O	.	.
GC13	1	16332485	G	T	C1orf64	Missense	p.G52W	4	36	11.1%	.	O	.
GC13	1	16474999	G	T	EPHA2	Missense	p.H179N	5	41	12.2%	.	.	.
GC13	1	16736388	G	T	SPATA21	Missense	p.H99N	5	31	16.1%	.	.	.
GC13	1	16775632	C	A	NECAP2	Missense	p.P142Q	6	64	9.4%	.	.	.
GC13	1	17559339	G	T	PADI1	Missense	p.R396L	6	75	8.0%	.	.	.
GC13	1	17570722	G	T	PADI1	Missense	p.W659L	6	59	10.2%	.	.	.
GC13	1	17949630	G	T	ARHGEF10L	Missense	p.W165L	11	74	14.9%	.	.	.
GC13	1	17949650	G	T	ARHGEF10L	Missense	p.G172W	6	73	8.2%	.	.	.
GC13	1	18691735	G	T	IGSF21	Missense	p.G187W	8	88	9.1%	.	.	.
GC13	1	19204044	C	A	ALDH4A1	Missense	p.G275W	5	49	10.2%	.	.	.
GC13	1	19499407	G	T	UBR4	Missense	p.L1158I	6	69	8.7%	.	.	.
GC13	1	19585207	G	T	MRTO4	Missense	p.K201N	8	139	5.8%	.	.	.
GC13	1	20964376	G	T	PINK1	Missense	p.L143F	8	100	8.0%	.	.	.
GC13	1	21016783	C	A	KIF17	Nonsense	p.E427X	4	24	16.7%	.	.	.
GC13	1	21106355	C	A	HP1BP3	Missense	p.R49L	6	71	8.5%	.	.	.
GC13	1	21267986	G	T	EIF4G3	Missense	p.P498Q	8	131	6.1%	.	.	.

GC13	1	21268499	G	T	EIF4G3	Missense	p.P327Q	8	115	7.0%	.	.	.
GC13	1	21582501	G	T	ECE1	Missense	p.P308Q	8	57	14.0%	.	.	.
GC13	1	22055084	G	T	USP48	Missense	p.Q477K	8	112	7.1%	.	.	.
GC13	1	22817940	G	T	ZBTB40	Missense	p.G249C	6	66	9.1%	.	.	.
GC13	1	22817970	C	A	ZBTB40	Missense	p.Q259K	9	78	11.5%	.	.	.
GC13	1	22832675	C	A	ZBTB40	Missense	p.P322Q	5	23	21.7%	.	.	.
GC13	1	22852708	C	A	ZBTB40	Missense	p.P1068Q	6	54	11.1%	.	.	.
GC13	1	23381583	G	T	KDM1A	Missense	p.R251L	6	94	6.4%	.	O	.
GC13	1	23419187	G	T	LUZP1	Missense	p.P523Q	8	87	9.2%	.	.	.
GC13	1	23420349	G	T	LUZP1	Missense	p.Q136K	8	121	6.6%	.	O	.
GC13	1	23520443	C	A	HTR1D	Missense	p.L90F	9	91	9.9%	.	.	.
GC13	1	23667386	G	T	HNRNPR	Missense	p.P39Q	11	103	10.7%	.	.	.
GC13	1	23765314	G	T	ASAP3	Missense	p.P334Q	6	72	8.3%	.	.	.
GC13	1	23767920	C	A	ASAP3	Missense	p.R256L	6	50	12.0%	.	.	.
GC13	1	23847529	C	A	E2F2	Missense	p.G205W	8	42	19.0%	.	.	.
GC13	1	24449887	C	A	IL22RA1	Nonsense	p.G233X	6	73	8.2%	.	.	.
GC13	1	24663258	G	T	GRHL3	Missense	p.G139W	7	87	8.0%	.	.	.
GC13	1	24921944	G	T	NCMAP	Missense	p.G8V	8	111	7.2%	.	.	.
GC13	1	24995886	G	T	SRRM1	Missense	p.R671L	6	93	6.5%	.	.	.
GC13	1	26349634	C	A	EXTL1	Missense	p.P166Q	6	59	10.2%	.	.	.
GC13	1	26515107	G	T	CNKSR1	Missense	p.G544W	9	70	12.9%	.	.	.
GC13	1	26596026	C	A	CEP85	Missense	p.Q473K	7	80	8.8%	.	.	.
GC13	1	26603760	G	T	CEP85	Missense	p.M187I	8	118	6.8%	.	O	.
GC13	1	27101628	G	T	ARID1A	Missense	p.R1637L	7	56	12.5%	O	.	.
GC13	1	27105827	C	A	ARID1A	Missense	p.P1813Q	6	75	8.0%	O	.	.
GC13	1	27124218	G	T	PIGV	Missense	p.M455I	7	81	8.6%	.	.	.
GC13	1	27427084	G	T	SLC9A1	Missense	p.P721Q	8	89	9.0%	.	.	.
GC13	1	27440591	C	A	SLC9A1	Missense	p.R180L	6	59	10.2%	.	.	.
GC13	1	27875811	C	A	AHDC1	Missense	p.R939L	6	59	10.2%	.	O	.
GC13	1	28176730	C	A	PPP1R8	Missense	p.Q53K	8	118	6.8%	.	.	.
GC13	1	28279783	C	A	SMPDL3B	Missense	p.P144Q	8	90	8.9%	.	.	.

GC13	1	28562906	G	T	ATPIF1	Missense	p.R41L	7	88	8.0%	.	.	.
GC13	1	28764930	C	A	PHACTR4	Missense	p.P12Q	8	108	7.4%	.	.	.
GC13	1	28819603	G	T	PHACTR4	Missense	p.R708L	7	102	6.9%	.	.	.
GC13	1	29069364	G	T	YTHDF2	Missense	p.L144F	7	40	17.5%	.	.	.
GC13	1	29314012	G	T	EPB41	Missense	p.E21D	8	106	7.5%	.	.	.
GC13	1	29319974	C	A	EPB41	Missense	p.Q201K	8	103	7.8%	.	.	.
GC13	1	29424445	C	A	EPB41	Missense	p.Q682K	5	46	10.9%	.	.	.
GC13	1	29522477	G	T	MECR	Missense	p.Q238K	7	97	7.2%	.	.	.
GC13	1	29644395	G	T	PTPRU	Missense	p.G1214W	9	70	12.9%	.	.	.
GC13	1	29647284	G	T	PTPRU	Missense	p.G1256W	7	67	10.4%	.	.	.
GC13	1	31478731	G	T	PUM1	Missense	p.P230Q	5	62	8.1%	.	.	.
GC13	1	31532239	C	A	PUM1	Missense	p.G59W	8	89	9.0%	.	.	.
GC13	1	31840268	C	A	FABP3	Missense	p.R118L	6	96	6.3%	.	.	.
GC13	1	31902328	G	T	SERINC2	Missense	p.W323L	7	82	8.5%	.	.	.
GC13	1	32121096	G	T	COL16A1	Missense	p.P1370Q	8	122	6.6%	.	.	.
GC13	1	32164193	C	A	COL16A1	Missense	p.R94L	6	46	13.0%	.	.	.
GC13	1	32265619	G	T	SPOCD1	Missense	p.Q69K	6	45	13.3%	.	.	.
GC13	1	32279753	C	A	SPOCD1	Missense	p.L394F	6	56	10.7%	.	.	.
GC13	1	32280507	G	T	SPOCD1	Missense	p.P143Q	8	103	7.8%	.	.	.
GC13	1	32502519	G	T	KHDRBS1	Missense	p.M221I	8	99	8.1%	.	.	.
GC13	1	32841883	G	T	BSDC1	Missense	p.P284H	6	58	10.3%	.	.	.
GC13	1	33293618	G	T	S100PBP	Missense	p.G286W	8	144	5.6%	.	.	.
GC13	1	33321575	G	T	S100PBP	Missense	p.W388L	10	97	10.3%	.	.	.
GC13	1	33404071	C	A	RNF19B	Missense	p.G557C	7	66	10.6%	.	.	.
GC13	1	33799692	G	T	PHC2	Missense	p.P51Q	6	74	8.1%	.	.	.
GC13	1	34002651	C	A	CSMD2	Missense	p.G3140W	6	69	8.7%	.	.	.
GC13	1	34015828	G	T	CSMD2	Missense	p.R2812S	7	64	10.9%	.	.	.
GC13	1	34070968	G	T	CSMD2	Missense	p.P2149Q	5	33	15.2%	.	.	.
GC13	1	34082506	C	A	CSMD2	Missense	p.A2006S	8	101	7.9%	.	.	.
GC13	1	34174743	C	A	CSMD2	Missense	p.K1174N	6	54	11.1%	.	.	.
GC13	1	34383773	G	A	CSMD2	Missense	p.A281V	43	70	61.4%	.	.	.

GC13	1	34667732	G	T	C1orf94	Missense	p.G440W	11	96	11.5%	.	.	.
GC13	1	35227597	G	T	GJB4	Missense	p.G248W	6	45	13.3%	.	.	.
GC13	1	35351777	C	A	DLGAP3	Missense	p.R499L	6	58	10.3%	.	.	.
GC13	1	35474441	G	T	ZMYM6	Missense	p.P526Q	9	90	10.0%	.	.	.
GC13	1	35579681	G	T	ZMYM1	Missense	p.L675F	7	80	8.8%	.	O	.
GC13	1	35579776	G	T	ZMYM1	Missense	p.G707V	9	123	7.3%	.	.	.
GC13	1	35656427	G	T	SFPQ	Missense	p.P396H	9	136	6.6%	.	.	.
GC13	1	35865111	G	T	ZMYM4	Missense	p.Q830H	9	115	7.8%	.	.	.
GC13	1	35885214	G	T	ZMYM4	Missense	p.R1204M	7	99	7.1%	.	.	.
GC13	1	36204815	G	T	CLSPN	Missense	p.Q1054K	7	88	8.0%	.	.	.
GC13	1	36297657	G	T	AGO4	Splicing	c.1117-1G>T	7	93	7.5%	.	.	.
GC13	1	36316561	G	T	AGO4	Missense	p.C795F	5	69	7.2%	.	.	.
GC13	1	36552552	G	T	TEKT2	Missense	p.W218L	9	90	10.0%	.	.	.
GC13	1	36755053	C	A	THRAP3	Missense	p.P478H	7	98	7.1%	.	.	.
GC13	1	36755107	C	A	THRAP3	Missense	p.P496Q	9	87	10.3%	.	.	.
GC13	1	36759520	G	T	THRAP3	Missense	p.R700L	7	123	5.7%	.	.	.
GC13	1	36775183	G	T	SH3D21	Missense	p.R143L	7	70	10.0%	.	.	.
GC13	1	36824418	G	T	STK40	Missense	p.R45S	7	91	7.7%	.	.	.
GC13	1	36859630	C	A	LSM10	Missense	p.R34L	5	48	10.4%	.	.	.
GC13	1	37948878	G	T	ZC3H12A	Missense	p.R489L	5	52	9.6%	.	.	.
GC13	1	38005896	G	T	SNIP1	Missense	p.P263Q	7	72	9.7%	.	.	.
GC13	1	38047913	G	T	GNL2	Missense	p.Q91K	7	88	8.0%	.	.	.
GC13	1	38158377	G	T	CDCA8	Missense	p.K5N	8	105	7.6%	.	.	.
GC13	1	38287812	G	T	MTF1	Missense	p.P583Q	5	41	12.2%	.	.	.
GC13	1	39823368	C	A	MACF1	Missense	p.L1854M	7	76	9.2%	.	.	.
GC13	1	39893786	C	A	MACF1	Missense	p.R3482S	9	123	7.3%	.	.	.
GC13	1	39893796	G	T	MACF1	Missense	p.R3485L	8	128	6.3%	.	.	.
GC13	1	39909224	G	T	MACF1	Missense	p.R4411M	9	63	14.3%	.	.	.
GC13	1	39920689	G	T	MACF1	Nonsense	p.E4940X	6	60	10.0%	.	O	.
GC13	1	39924757	G	T	MACF1	Splicing	c.15020-1G>T	8	103	7.8%	.	.	.
GC13	1	40029582	G	T	PABPC4	Missense	p.P489Q	7	104	6.7%	.	.	.

GC13	1	40149707	G	T	HPCAL4	Missense	p.R94S	7	83	8.4%	.	.	.
GC13	1	40310205	G	T	TRIT1	Missense	p.Q290K	8	121	6.6%	.	.	.
GC13	1	40882755	G	T	SMAP2	Missense	p.W354L	7	89	7.9%	.	.	.
GC13	1	41012273	G	T	ZNF684	Missense	p.R93L	9	134	6.7%	.	.	.
GC13	1	41012334	G	T	ZNF684	Missense	p.M113I	7	89	7.9%	.	.	.
GC13	1	41285571	G	T	KCNQ4	Missense	p.G287C	7	81	8.6%	.	.	.
GC13	1	41608592	C	A	SCMH1	Missense	p.G67W	7	92	7.6%	.	.	.
GC13	1	42046136	G	T	HIVEP3	Missense	p.Q1445K	8	133	6.0%	.	.	.
GC13	1	42046294	G	T	HIVEP3	Missense	p.P1392Q	7	79	8.9%	.	.	.
GC13	1	42664829	G	T	FOXJ3	Missense	p.Q201K	8	115	7.0%	.	.	.
GC13	1	42744159	C	A	FOXJ3	Missense	p.G77W	9	95	9.5%	.	.	.
GC13	1	42744307	C	A	FOXJ3	Missense	p.M27I	8	86	9.3%	.	.	.
GC13	1	43110363	G	T	CCDC30	Splicing	c.1776-1G>T	7	96	7.3%	.	.	.
GC13	1	43205566	G	T	CLDN19	Missense	p.Q57K	8	92	8.7%	.	.	.
GC13	1	43296576	C	A	ERMAP	Missense	p.Q75K	7	71	9.9%	.	.	.
GC13	1	43630448	G	T	EBNA1BP2	Missense	p.Q246K	8	103	7.8%	.	.	.
GC13	1	43663223	G	T	CFAP57	Splicing	c.1123-1G>T;NM_001195831	6	49	12.2%	.	.	.
GC13	1	43782967	G	T	TIE1	Missense	p.W791L	6	68	8.8%	.	.	.
GC13	1	43825238	C	A	CDC20	Missense	p.Q87K	8	119	6.7%	.	.	.
GC13	1	43887790	C	A	SZT2	Missense	p.H555N	6	56	10.7%	.	.	.
GC13	1	43911579	G	T	SZT2	Missense	p.R2890L	7	87	8.0%	.	.	.
GC13	1	43917962	G	T	HYI	Missense	p.Q114K	7	79	8.9%	.	.	.
GC13	1	44057558	C	G	PTPRF	Missense	p.P536R	30	49	61.2%	.	.	.
GC13	1	44360102	G	T	ST3GAL3	Missense	p.W70L	8	75	10.7%	.	.	.
GC13	1	44442904	G	T	ATP6V0B	Missense	p.G203W	10	102	9.8%	.	.	.
GC13	1	44443073	G	T	ATP6V0B	Missense	p.W259L	8	123	6.5%	.	.	.
GC13	1	44482727	G	T	SLC6A9	Missense	p.P27T	5	41	12.2%	.	.	.
GC13	1	44679448	G	T	DMAP1	Missense	p.R8L	6	69	8.7%	.	.	.
GC13	1	44878316	G	T	RNF220	Missense	p.G183W	7	89	7.9%	.	.	.
GC13	1	45125854	G	T	TMEM53	Missense	p.H59N	9	89	10.1%	.	.	.
GC13	1	45220447	G	T	KIF2C	Missense	p.W189L	7	61	11.5%	.	.	.

GC13	1	45274520	G	T	BTBD19	Missense	p.G10W	8	91	8.8%	.	.	.
GC13	1	45293814	C	A	PTCH2	Missense	p.G587W	6	58	10.3%	.	.	.
GC13	1	45473946	C	A	HECTD3	Missense	p.W429L	7	79	8.9%	.	.	.
GC13	1	45474319	C	A	HECTD3	Missense	p.R376L	6	74	8.1%	.	.	.
GC13	1	45484247	C	A	ZSWIM5	Missense	p.R1146L	10	106	9.4%	.	O	.
GC13	1	45811000	C	A	TESK2	Missense	p.G327W	7	79	8.9%	.	.	.
GC13	1	46032280	C	A	AKR1A1	Missense	p.R42S	7	82	8.5%	.	.	.
GC13	1	46073239	C	A	NASP	Missense	p.P155Q	7	78	9.0%	.	.	.
GC13	1	46159257	G	T	TMEM69	Missense	p.G142W	7	91	7.7%	.	.	.
GC13	1	46160154	C	A	IPP	Missense	p.W562L	8	100	8.0%	.	.	.
GC13	1	46468494	G	T	MAST2	Missense	p.W229L	7	65	10.8%	.	.	.
GC13	1	46498076	G	T	MAST2	Missense	p.W1138C	7	76	9.2%	.	.	.
GC13	1	46532602	G	T	PIK3R3	Missense	p.P3Q	8	118	6.8%	.	.	.
GC13	1	46660274	C	A	POMGNT1	Missense	p.W212C	8	99	8.1%	.	.	.
GC13	1	46660312	C	A	POMGNT1	Missense	p.G200W	6	86	7.0%	.	.	.
GC13	1	46745864	C	A	LRRC41	Nonsense	p.E674X	7	85	8.2%	.	.	.
GC13	1	47149014	G	T	EFCAB14	Missense	p.Q424K	9	126	7.1%	.	.	.
GC13	1	47560310	G	T	CYP4Z1	Missense	p.W282L	9	91	9.9%	.	.	.
GC13	1	47603206	G	T	CYP4A22	Missense	p.G17W	7	89	7.9%	.	.	.
GC13	1	47606504	C	A	CYP4A22	Missense	p.P83Q	13	105	12.4%	.	.	.
GC13	1	47606561	C	A	CYP4A22	Missense	p.P102H	6	58	10.3%	.	.	.
GC13	1	47611537	G	T	CYP4A22	Splicing	c.1223-1G>T	6	63	9.5%	.	.	.
GC13	1	47746728	G	T	STIL	Missense	p.Q468K	7	82	8.5%	.	.	.
GC13	1	47748124	G	T	STIL	Missense	p.Q381K	6	64	9.4%	.	.	.
GC13	1	47761449	G	T	STIL	Missense	p.P258Q	8	77	10.4%	.	.	.
GC13	1	47767312	G	T	STIL	Missense	p.P125Q	7	78	9.0%	.	.	.
GC13	1	48764454	C	A	SPATA6	Missense	p.M452I	7	91	7.7%	.	.	.
GC13	1	48918712	G	T	SPATA6	Missense	p.P48Q	7	81	8.6%	.	.	.
GC13	1	48918793	G	T	SPATA6	Missense	p.P21Q	7	99	7.1%	.	.	.
GC13	1	50610818	G	T	ELAVL4	Missense	p.G67W	9	99	9.1%	.	.	.
GC13	1	51753850	C	A	TTC39A	Missense	p.M148I	8	110	7.3%	.	.	.

GC13	1	52277718	G	T	NRDC	Missense	p.P576Q	7	88	8.0%	.	.	.
GC13	1	52703908	G	T	ZFYVE9	Missense	p.Q273H	7	74	9.5%	.	.	.
GC13	1	52704023	C	A	ZFYVE9	Missense	p.H312N	6	64	9.4%	.	.	.
GC13	1	52863406	G	T	ORC1	Missense	p.P118Q	7	67	10.4%	.	.	.
GC13	1	53237196	G	T	ZYG11B	Missense	p.R234L	6	82	7.3%	.	.	.
GC13	1	53535799	C	A	PODN	Missense	p.P139Q	7	82	8.5%	.	.	.
GC13	1	53675798	G	T	CPT2	Missense	p.R151L	8	128	6.3%	.	.	.
GC13	1	53676038	G	T	CPT2	Missense	p.R231L	6	91	6.6%	.	.	.
GC13	1	54266395	G	T	NDC1	Missense	p.P358Q	7	91	7.7%	.	.	.
GC13	1	54360015	G	T	DIO1	Missense	p.M44I	7	100	7.0%	.	.	.
GC13	1	54644917	C	A	CYB5RL	Missense	p.G217C	7	94	7.4%	.	.	.
GC13	1	55199330	G	T	TTC4	Missense	p.G309C	6	50	12.0%	.	.	.
GC13	1	55224039	C	A	PARS2	Nonsense	p.G266X	7	53	13.2%	.	.	.
GC13	1	55307473	G	T	LEXM	Splicing	c.1185-1G>T	8	79	10.1%	.	.	.
GC13	1	55451890	C	A	TMEM61	Missense	p.Q46K	7	62	11.3%	.	.	.
GC13	1	57221597	C	A	C1orf168	Missense	p.G368W	6	66	9.1%	.	.	.
GC13	1	59131270	C	A	MYSM1	Missense	p.G689W	7	69	10.1%	.	.	.
GC13	1	60463392	G	T	C1orf87	Missense	p.Q457K	8	89	9.0%	.	.	.
GC13	1	60520952	G	T	C1orf87	Missense	p.P89Q	8	120	6.7%	.	.	.
GC13	1	61554253	C	A	NFIA	Missense	p.P154T	7	76	9.2%	.	.	.
GC13	1	61920986	G	T	NFIA	Missense	p.G478W	6	72	8.3%	.	.	.
GC13	1	62261139	G	T	PATJ	Missense	p.G390V	8	121	6.6%	.	.	.
GC13	1	62673033	C	A	L1TD1	Missense	p.L245M	8	120	6.7%	.	.	.
GC13	1	62673253	C	A	L1TD1	Missense	p.P318Q	12	127	9.4%	.	.	.
GC13	1	62910645	C	A	USP1	Missense	p.P265Q	9	86	10.5%	.	.	.
GC13	1	63010677	C	A	DOCK7	Missense	p.G934C	7	77	9.1%	.	.	.
GC13	1	63068016	G	T	ANGPTL3	Missense	p.W299L	10	85	11.8%	.	.	.
GC13	1	63300516	C	A	ATG4C	Missense	p.P361H	11	136	8.1%	.	.	.
GC13	1	63789990	G	T	FOXD3	Missense	p.G421W	4	28	14.3%	.	.	.
GC13	1	63872018	C	A	ALG6	Missense	p.P126H	7	90	7.8%	.	.	.
GC13	1	64515446	G	T	ROR1	Missense	p.G83W	7	84	8.3%	.	.	.

GC13	1	64605944	C	A	ROR1	Missense	p.L255M	7	97	7.2%	.	.	.
GC13	1	64643970	G	T	ROR1	Missense	p.W749L	10	84	11.9%	.	.	.
GC13	1	64644099	C	A	ROR1	Missense	p.P792Q	7	96	7.3%	.	.	.
GC13	1	65119521	G	T	CACHD1	Nonsense	p.E245X	20	73	27.4%	.	.	.
GC13	1	65141145	C	A	CACHD1	Missense	p.P634Q	9	92	9.8%	.	.	.
GC13	1	65854990	G	T	DNAJC6	Missense	p.Q415H	7	96	7.3%	.	.	.
GC13	1	66070852	G	T	LEPR	Missense	p.W512L	8	121	6.6%	.	.	.
GC13	1	66074505	G	T	LEPR	Missense	p.W558L	10	120	8.3%	.	.	.
GC13	1	66458696	C	A	PDE4B	Missense	p.P36Q	8	146	5.5%	.	.	.
GC13	1	66723334	G	T	PDE4B	Missense	p.G146C	7	142	4.9%	.	.	.
GC13	1	66821229	G	T	PDE4B	Missense	p.R84L	7	117	6.0%	.	.	.
GC13	1	67185008	G	T	SGIP1	Missense	p.M357I	6	71	8.5%	.	.	.
GC13	1	67207080	G	T	SGIP1	Missense	p.G612W	13	147	8.8%	.	O	.
GC13	1	67220338	G	T	TCTEX1D1	Splicing	.	8	134	6.0%	.	.	.
GC13	1	67263901	G	T	INSL5	Missense	p.P68Q	7	71	9.9%	.	.	.
GC13	1	67303458	C	A	WDR78	Missense	p.G506W	10	152	6.6%	.	.	.
GC13	1	67359033	C	A	WDR78	Missense	p.G137C	9	108	8.3%	.	.	.
GC13	1	67391848	C	A	MIER1	Missense	p.Q31K	7	56	12.5%	.	.	.
GC13	1	67450408	C	A	MIER1	Missense	p.P455Q	9	116	7.8%	.	.	.
GC13	1	67558794	G	T	C1orf141	Missense	p.P366H	9	162	5.6%	.	.	.
GC13	1	67635086	G	T	IL23R	Missense	p.M44I	9	106	8.5%	.	.	.
GC13	1	67787323	C	A	IL12RB2	Missense	p.H39N	7	82	8.5%	.	.	.
GC13	1	67891921	C	A	SERBP1	Missense	p.G121W	6	45	13.3%	.	.	.
GC13	1	68906550	G	T	RPE65	Missense	p.P210Q	6	61	9.8%	.	.	.
GC13	1	70446106	G	T	LRRC7	Missense	p.M214I	9	116	7.8%	.	.	.
GC13	1	70502298	G	T	LRRC7	Missense	p.W722L	11	129	8.5%	.	.	.
GC13	1	70504368	C	A	LRRC7	Missense	p.P916Q	8	142	5.6%	.	.	.
GC13	1	70518718	C	A	LRRC7	Missense	p.Q1336K	8	125	6.4%	.	.	.
GC13	1	70518742	G	T	LRRC7	Nonsense	p.G1344X	8	123	6.5%	.	.	.
GC13	1	70698050	C	A	SRSF11	Missense	p.P146Q	7	51	13.7%	.	.	.
GC13	1	70761826	C	A	ANKRD13C	Missense	p.R347L	8	144	5.6%	.	.	.

GC13	1	70890001	C	A	CTH	Missense	p.Q132K	10	137	7.3%	.	.	.
GC13	1	71513119	C	A	PTGER3	Nonsense	p.G48X	7	96	7.3%	.	.	.
GC13	1	74648235	G	T	LRRIQ3	Missense	p.P187Q	6	68	8.8%	.	.	.
GC13	1	75036821	G	T	ERICH3	Missense	p.P1525T	7	60	11.7%	.	.	.
GC13	1	75055332	G	T	ERICH3	Missense	p.P720H	11	91	12.1%	.	.	.
GC13	1	75172644	G	T	CRYZ	Missense	p.P119Q	7	95	7.4%	.	.	.
GC13	1	75609535	G	T	LHX8	Missense	p.G196W	7	52	13.5%	.	.	.
GC13	1	75614305	C	A	LHX8	Missense	p.P240T	8	111	7.2%	.	.	.
GC13	1	78245375	G	T	MIGA1	Missense	p.W12L	6	46	13.0%	.	.	.
GC13	1	78272712	G	T	MIGA1	Missense	p.W188L	7	84	8.3%	.	.	.
GC13	1	78407868	G	T	NEXN	Missense	p.R481M	7	100	7.0%	.	.	.
GC13	1	79383588	C	A	ADGRL4	Missense	p.G537C	7	77	9.1%	.	.	.
GC13	1	82408866	C	A	ADGRL2	Missense	p.P204Q	8	97	8.2%	.	.	.
GC13	1	82409331	C	A	ADGRL2	Missense	p.P359H	8	95	8.4%	.	.	.
GC13	1	82417789	C	A	ADGRL2	Missense	p.P582H	6	62	9.7%	.	.	.
GC13	1	85029067	C	A	CTBS	Missense	p.R277L	4	38	10.5%	.	O	.
GC13	1	85331695	C	A	LPAR3	Missense	p.G37W	7	100	7.0%	.	.	.
GC13	1	85403446	G	T	MCOLN2	Missense	p.H443N	6	61	9.8%	.	.	.
GC13	1	85624672	G	T	SYDE2	Missense	p.P1116T	7	100	7.0%	.	.	.
GC13	1	85648757	G	T	SYDE2	Missense	p.P523Q	8	113	7.1%	.	.	.
GC13	1	86047796	G	T	CYR61	Missense	p.G155W	7	93	7.5%	.	.	.
GC13	1	86948018	C	A	CLCA1	Missense	p.R230S	6	55	10.9%	.	.	.
GC13	1	87025996	C	A	CLCA4	Missense	p.P135T	6	59	10.2%	.	.	.
GC13	1	87029421	C	A	CLCA4	Missense	p.R176S	7	102	6.9%	.	.	.
GC13	1	87805885	G	T	LMO4	Missense	p.K163N	8	138	5.8%	.	.	.
GC13	1	90049706	G	T	LRRC8B	Missense	p.M499I	7	71	9.9%	.	.	.
GC13	1	90179169	G	T	LRRC8C	Missense	p.R347L	9	104	8.7%	.	.	.
GC13	1	90400092	C	A	LRRC8D	Missense	p.L489I	7	91	7.7%	.	.	.
GC13	1	90400737	C	A	LRRC8D	Missense	p.H704N	10	123	8.1%	.	.	.
GC13	1	90400908	G	T	LRRC8D	Missense	p.G761W	8	123	6.5%	.	.	.
GC13	1	90463703	G	T	ZNF326	Missense	p.Q17H	13	127	10.2%	.	.	.

GC13	1	91182647	C	A	BARHL2	Missense	p.G36C	5	42	11.9%	.	.	.
GC13	1	91403345	G	T	ZNF644	Missense	p.R1129S	9	148	6.1%	.	.	.
GC13	1	91403925	G	T	ZNF644	Missense	p.R996S	8	114	7.0%	.	.	.
GC13	1	91851285	C	A	HFM1	Missense	p.G201W	7	97	7.2%	.	.	.
GC13	1	92595311	C	A	BTBD8	Missense	p.Q244K	7	79	8.9%	.	.	.
GC13	1	92645973	G	T	KIAA1107	Missense	p.K398N	7	92	7.6%	.	.	.
GC13	1	92647268	C	A	KIAA1107	Missense	p.P830H	8	108	7.4%	.	.	.
GC13	1	93070928	C	A	EVI5	Missense	p.R653L	6	87	6.9%	.	.	.
GC13	1	94000394	G	T	FNBP1L	Missense	p.G307W	6	69	8.7%	.	O	.
GC13	1	94341846	G	T	DNTTIP2	Missense	p.L549I	7	57	12.3%	.	.	.
GC13	1	94342680	G	T	DNTTIP2	Missense	p.H271N	9	101	8.9%	.	.	.
GC13	1	94343139	G	T	DNTTIP2	Missense	p.P118T	8	102	7.8%	.	.	.
GC13	1	94526286	G	T	ABCA4	Missense	p.P656H	11	118	9.3%	.	.	.
GC13	1	95365028	C	A	CNN3	Missense	p.G137W	6	73	8.2%	.	.	.
GC13	1	99422288	C	A	PLPPR5	Nonsense	p.G83X	6	72	8.3%	.	.	.
GC13	1	99772460	G	T	PLPPR4	Missense	p.R671L	6	78	7.7%	.	.	.
GC13	1	100154868	G	T	PALMD	Missense	p.W351L	9	115	7.8%	.	.	.
GC13	1	100318259	G	T	AGL	Missense	p.G12W	6	62	9.7%	.	.	.
GC13	1	100357208	C	A	AGL	Missense	p.P999Q	10	103	9.7%	.	.	.
GC13	1	100546179	G	T	MFSD14A	Missense	p.L410F	8	108	7.4%	.	.	.
GC13	1	100598765	C	A	TRMT13	Missense	p.P14Q	6	51	11.8%	.	.	.
GC13	1	100964693	G	T	CDC14A	Missense	p.G251W	6	98	6.1%	.	.	.
GC13	1	101186049	G	T	VCAM1	Nonsense	p.E28X	6	72	8.3%	.	O	.
GC13	1	101188666	C	A	VCAM1	Missense	p.P144Q	7	61	11.5%	.	.	.
GC13	1	104085994	C	A	RNPC3	Missense	p.H324N	7	70	10.0%	.	.	.
GC13	1	108319910	G	T	VAV3	Missense	p.P130Q	7	92	7.6%	.	.	.
GC13	1	108679283	G	T	SLC25A24	Missense	p.Q476K	7	94	7.4%	.	.	.
GC13	1	108700211	G	T	SLC25A24	Missense	p.P181Q	9	109	8.3%	.	.	.
GC13	1	109200161	C	A	HENMT1	Missense	p.R21M	8	79	10.1%	.	.	.
GC13	1	109235362	G	T	PRPF38B	Missense	p.W50L	7	77	9.1%	.	.	.
GC13	1	109342890	C	A	STXBP3	Missense	p.Q500K	7	95	7.4%	.	.	.

GC13	1	109538443	C	A	WDR47	Missense	p.G492C	8	104	7.7%	.	.	.
GC13	1	109554139	G	T	WDR47	Missense	p.P184T	10	141	7.1%	.	.	.
GC13	1	109637154	C	A	TMEM167B	Missense	p.Q107K	7	78	9.0%	.	.	.
GC13	1	109811586	C	A	CELSR2	Missense	p.P2196Q	6	64	9.4%	.	.	.
GC13	1	110018245	G	T	SYPL2	Missense	p.G58W	5	41	12.2%	.	O	.
GC13	1	110561016	G	T	AHCYL1	Missense	p.R335L	10	129	7.8%	.	.	.
GC13	1	110596437	G	T	STRIP1	Missense	p.R711L	10	97	10.3%	.	.	.
GC13	1	110883502	G	T	RBM15	Missense	p.W492L	8	102	7.8%	.	.	.
GC13	1	110883721	G	T	RBM15	Missense	p.R565L	6	94	6.4%	.	.	.
GC13	1	111060292	C	A	KCNA10	Missense	p.R373L	6	73	8.2%	.	.	.
GC13	1	111061150	C	A	KCNA10	Missense	p.R87L	7	60	11.7%	.	.	.
GC13	1	111146320	G	T	KCNA2	Missense	p.P362Q	7	74	9.5%	.	.	.
GC13	1	111146336	G	T	KCNA2	Missense	p.Q357K	7	83	8.4%	.	.	.
GC13	1	111146810	C	A	KCNA2	Missense	p.G199W	7	95	7.4%	.	.	.
GC13	1	111957846	G	T	OVGP1	Missense	p.P426Q	6	55	10.9%	.	.	.
GC13	1	111964027	C	A	OVGP1	Missense	p.M258I	7	55	12.7%	.	.	.
GC13	1	112305308	G	T	DDX20	Missense	p.G372W	9	115	7.8%	.	.	.
GC13	1	112999193	G	T	CTTNBP2NL	Missense	p.R360M	8	91	8.8%	.	.	.
GC13	1	113062955	G	T	WNT2B	Missense	p.G242C	6	94	6.4%	.	.	.
GC13	1	113196269	G	T	CAPZA1	Nonsense	p.G69X	7	77	9.1%	.	O	.
GC13	1	113201665	G	T	CAPZA1	Missense	p.G150W	6	67	9.0%	.	.	.
GC13	1	113238857	C	A	MOV10	Missense	p.L623I	7	69	10.1%	.	.	.
GC13	1	113252885	G	T	PPM1J	Missense	p.P473H	5	37	13.5%	.	.	.
GC13	1	113253160	C	A	PPM1J	Missense	p.W431L	10	103	9.7%	.	O	.
GC13	1	113460166	C	A	SLC16A1	Missense	p.G288W	7	85	8.2%	.	.	.
GC13	1	113633939	G	T	LRIG2	Splicing	c.240-1G>T	8	118	6.8%	.	.	.
GC13	1	114226173	C	A	MAGI3	Missense	p.P1328Q	8	97	8.2%	.	.	.
GC13	1	114246785	C	A	PHTF1	Missense	p.R317L	6	73	8.2%	.	.	.
GC13	1	114267420	G	T	PHTF1	Missense	p.P195H	7	91	7.7%	.	.	.
GC13	1	114506008	G	T	HIPK1	Splicing	c.2104-1G>T;NM_198269	8	99	8.1%	.	.	.

GC13	1	114511107	G	T	HIPK1	Missense	p.G476W	7	88	8.0%	.	.	.
GC13	1	114516057	G	T	HIPK1	Missense	p.G792W	6	79	7.6%	.	.	.
GC13	1	114524246	G	T	OLFML3	Missense	p.R298L	7	83	8.4%	.	O	.
GC13	1	114680497	C	A	SYT6	Missense	p.G146W	7	93	7.5%	.	.	.
GC13	1	114970419	C	A	TRIM33	Missense	p.W418L	9	109	8.3%	.	.	.
GC13	1	115419387	C	A	SYCP1	Missense	p.L253I	8	139	5.8%	.	.	.
GC13	1	115828855	C	A	NGF	Missense	p.G188W	7	93	7.5%	.	.	.
GC13	1	115828878	C	A	NGF	Missense	p.R180L	8	91	8.8%	.	.	.
GC13	1	116206619	G	T	VANGL1	Missense	p.R179L	7	109	6.4%	.	.	.
GC13	1	116941624	C	A	ATP1A1	Missense	p.P789Q	8	83	9.6%	O	.	.
GC13	1	116944248	G	T	ATP1A1	Missense	p.M974I	8	114	7.0%	O	.	.
GC13	1	117552691	G	T	CD101	Missense	p.R88L	6	99	6.1%	.	.	.
GC13	1	117554216	G	T	CD101	Missense	p.G157C	8	63	12.7%	.	.	.
GC13	1	117620571	G	T	TTF2	Missense	p.W536L	6	49	12.2%	.	.	.
GC13	1	117660785	G	T	TRIM45	Missense	p.R365S	5	61	8.2%	.	.	.
GC13	1	117661184	G	T	TRIM45	Missense	p.H232N	7	53	13.2%	.	.	.
GC13	1	117663760	C	A	TRIM45	Missense	p.G22W	7	66	10.6%	.	.	.
GC13	1	117948207	G	T	MAN1A2	Missense	p.G199W	7	92	7.6%	.	.	.
GC13	1	118462956	G	T	GDAP2	Missense	p.Q9K	11	102	10.8%	.	.	.
GC13	1	118492452	C	A	WDR3	Missense	p.Q535K	12	88	13.6%	.	.	.
GC13	1	118509377	C	A	SPAG17	Splicing	c.6388-1G>T	7	88	8.0%	.	.	.
GC13	1	118628545	C	A	SPAG17	Missense	p.D588Y	7	89	7.9%	.	.	.
GC13	1	118644425	G	T	SPAG17	Missense	p.P191Q	5	36	13.9%	.	.	.
GC13	1	118658004	C	A	SPAG17	Missense	p.G126W	7	92	7.6%	.	.	.
GC13	1	118693959	C	A	SPAG17	Missense	p.G41W	6	56	10.7%	.	.	.
GC13	1	120438355	G	T	ADAM30	Missense	p.P202Q	7	80	8.8%	.	.	.
GC13	1	120438932	G	T	ADAM30	Missense	p.Q10K	6	62	9.7%	.	.	.
GC13	1	120458118	C	A	NOTCH2	Missense	p.Q2409H	7	70	10.0%	O	.	.
GC13	1	120459193	C	A	NOTCH2	Missense	p.R2051L	5	71	7.0%	O	.	.
GC13	1	120529663	G	T	NOTCH2	Missense	p.P265H	8	72	11.1%	O	.	.
GC13	1	144930862	C	A	PDE4DIP	Missense	p.G283W	12	267	4.5%	.	.	.

GC13	1	144931287	C	A	PDE4DIP	Missense	p.W141L	10	188	5.3%	.	.	.
GC13	1	144931582	G	T	PDE4DIP	Missense	p.R43S	13	317	4.1%	.	.	.
GC13	1	145439925	G	T	TXNIP	Missense	p.M102I	9	144	6.3%	.	.	.
GC13	1	145440783	C	A	TXNIP	Missense	p.P273Q	9	171	5.3%	.	.	.
GC13	1	145474665	G	T	ANKRD34A	Missense	p.R446L	9	129	7.0%	.	.	.
GC13	1	145474698	G	T	ANKRD34A	Missense	p.G457V	9	131	6.9%	.	.	.
GC13	1	145522530	C	A	PEX11B	Missense	p.L117I	11	169	6.5%	.	.	.
GC13	1	145532762	C	A	ITGA10	Missense	p.H218N	9	124	7.3%	.	O	.
GC13	1	145539049	G	T	ITGA10	Missense	p.G854W	8	78	10.3%	.	.	.
GC13	1	145601837	C	A	POLR3C	Missense	p.G245W	7	86	8.1%	.	.	.
GC13	1	146672961	C	A	FMO5	Missense	p.R319M	9	177	5.1%	.	.	.
GC13	1	146684011	C	A	FMO5	Missense	p.G194W	8	136	5.9%	.	.	.
GC13	1	146724357	G	T	CHD1L	Missense	p.W5L	7	84	8.3%	.	.	.
GC13	1	146740536	G	T	CHD1L	Missense	p.G158W	7	100	7.0%	.	.	.
GC13	1	146742656	G	T	CHD1L	Missense	p.M179I	10	163	6.1%	.	.	.
GC13	1	147091826	C	A	BCL9	Missense	p.P622Q	9	146	6.2%	.	.	.
GC13	1	147091892	C	A	BCL9	Missense	p.P644H	8	138	5.8%	.	.	.
GC13	1	147095719	G	T	BCL9	Missense	p.M1080I	9	140	6.4%	.	.	.
GC13	1	147142088	C	A	ACP6	Missense	p.R28L	6	64	9.4%	.	.	.
GC13	1	147231114	C	A	GJA5	Missense	p.W78L	9	145	6.2%	.	.	.
GC13	1	149859172	C	A	HIST2H2AB	Missense	p.G99W	9	168	5.4%	.	.	.
GC13	1	149885251	G	T	SV2A	Missense	p.R48S	9	146	6.2%	.	.	.
GC13	1	149895734	C	A	SF3B4	Missense	p.M362I	10	88	11.4%	.	.	.
GC13	1	149905522	G	T	MTMR11	Missense	p.P209T	9	142	6.3%	.	.	.
GC13	1	149905572	G	T	MTMR11	Missense	p.P192H	8	118	6.8%	.	.	.
GC13	1	149916403	G	T	OTUD7B	Missense	p.Q629K	12	177	6.8%	.	.	.
GC13	1	150131668	C	A	PLEKHO1	Missense	p.H222N	7	91	7.7%	.	.	.
GC13	1	150255784	G	T	CIART	Missense	p.R36M	9	137	6.6%	.	.	.
GC13	1	150413464	C	A	RPRD2	Missense	p.P134Q	11	183	6.0%	.	.	.
GC13	1	150443146	G	T	RPRD2	Missense	p.K548N	10	168	6.0%	.	.	.
GC13	1	150443741	G	T	RPRD2	Missense	p.G747W	9	138	6.5%	.	.	.

GC13	1	150444182	G	T	RPRD2	Missense	p.G894W	12	134	9.0%	.	.	.
GC13	1	150444281	G	T	RPRD2	Missense	p.G927W	9	166	5.4%	.	.	.
GC13	1	150444536	G	T	RPRD2	Missense	p.G1012C	10	165	6.1%	.	.	.
GC13	1	150445028	G	T	RPRD2	Missense	p.G1176W	12	143	8.4%	.	.	.
GC13	1	150471140	G	T	TARS2	Missense	p.Q337H	9	143	6.3%	.	.	.
GC13	1	150484919	G	T	ECM1	Missense	p.R267L	8	128	6.3%	.	.	.
GC13	1	150485214	G	T	ECM1	Missense	p.G315W	10	114	8.8%	.	.	.
GC13	1	150529805	G	T	ADAMTSL4	Missense	p.G681W	11	156	7.1%	.	.	.
GC13	1	150530600	G	T	ADAMTSL4	Missense	p.W786L	9	103	8.7%	.	.	.
GC13	1	150667217	G	T	GOLPH3L	Missense	p.P33Q	9	183	4.9%	.	.	.
GC13	1	150689646	G	T	HORMAD1	Missense	p.P49Q	10	116	8.6%	.	.	.
GC13	1	150727593	G	T	CTSS	Missense	p.L95M	9	142	6.3%	.	.	.
GC13	1	150772163	G	T	CTSK	Missense	p.P214Q	10	156	6.4%	.	.	.
GC13	1	150779209	G	T	CTSK	Missense	p.H25N	8	83	9.6%	.	.	.
GC13	1	150789839	G	T	ARNT	Missense	p.Q511K	9	96	9.4%	.	.	.
GC13	1	150790412	C	A	ARNT	Nonsense	p.G482X	11	153	7.2%	.	.	.
GC13	1	150923406	G	T	SETDB1	Missense	p.G685W	13	145	9.0%	.	.	.
GC13	1	150941535	C	A	CERS2	Missense	p.W11L	11	140	7.9%	.	.	.
GC13	1	150960358	G	T	ANXA9	Missense	p.R208L	5	56	8.9%	.	.	.
GC13	1	150975051	C	A	MINDY1	Missense	p.G63W	7	100	7.0%	.	.	.
GC13	1	151001275	G	T	PRUNE1	Missense	p.R62M	6	56	10.7%	.	.	.
GC13	1	151010135	C	A	BNIPL	Missense	p.P45H	8	70	11.4%	.	.	.
GC13	1	151021099	G	T	C1orf56	Missense	p.R259L	10	131	7.6%	.	.	.
GC13	1	151060757	C	A	GABPB2	Missense	p.P31Q	9	149	6.0%	.	.	.
GC13	1	151110816	G	T	SEMA6C	Missense	p.Q175K	7	95	7.4%	.	.	.
GC13	1	151146038	G	T	TMOD4	Missense	p.L112M	8	104	7.7%	.	.	.
GC13	1	151204807	G	T	PIP5K1A	Missense	p.R139L	9	102	8.8%	.	.	.
GC13	1	151234744	C	A	PSMD4	Missense	p.P45H	9	144	6.3%	.	.	.
GC13	1	151267010	C	A	PI4KB	Missense	p.G343W	13	121	10.7%	.	.	.
GC13	1	151288738	G	T	PI4KB	Missense	p.P74T	9	129	7.0%	.	.	.
GC13	1	151314747	G	T	RFX5	Missense	p.P589Q	8	121	6.6%	.	.	.

GC13	1	151314919	G	T	RFX5	Missense	p.Q532K	14	135	10.4%	.	O	.
GC13	1	151342362	C	A	SELENBP1	Missense	p.W5L	8	125	6.4%	.	.	.
GC13	1	151378471	G	T	POGZ	Missense	p.Q919K	12	211	5.7%	.	.	.
GC13	1	151378908	C	A	POGZ	Missense	p.R773L	8	49	16.3%	.	.	.
GC13	1	151402188	C	A	POGZ	Splicing	c.301-1G>T;NM_207171	8	126	6.3%	.	.	.
GC13	1	151495934	C	A	CGN	Missense	p.Q389K	7	84	8.3%	.	.	.
GC13	1	151512886	C	A	TUFT1	Missense	p.P15Q	7	78	9.0%	.	.	.
GC13	1	151553512	C	A	TUFT1	Missense	p.P343Q	7	89	7.9%	.	.	.
GC13	1	151630828	G	T	SNX27	Missense	p.G221W	7	93	7.5%	.	.	.
GC13	1	151747630	C	A	TDRKH	Missense	p.G483W	10	170	5.9%	.	.	.
GC13	1	151751181	G	T	TDRKH	Missense	p.P288Q	9	92	9.8%	.	.	.
GC13	1	151754043	C	A	TDRKH	Missense	p.G49W	6	78	7.7%	.	.	.
GC13	1	151801911	G	T	RORC	Missense	p.H22N	11	147	7.5%	.	.	.
GC13	1	151823532	G	T	THEM5	Missense	p.P154Q	6	71	8.5%	.	.	.
GC13	1	152057468	C	A	TCHHL1	Missense	p.R897M	11	158	7.0%	.	.	.
GC13	1	152058230	G	T	TCHHL1	Missense	p.P643Q	8	125	6.4%	.	.	.
GC13	1	152058888	C	A	TCHHL1	Missense	p.G424W	11	181	6.1%	.	.	.
GC13	1	152081750	G	T	TCHH	Missense	p.Q1315K	12	235	5.1%	.	.	.
GC13	1	152081812	G	T	TCHH	Missense	p.P1294Q	17	230	7.4%	.	.	.
GC13	1	152081924	G	T	TCHH	Missense	p.Q1257K	12	178	6.7%	.	.	.
GC13	1	152085338	G	T	TCHH	Missense	p.Q119K	10	181	5.5%	.	.	.
GC13	1	152127249	G	T	RPTN	Missense	p.H776N	12	229	5.2%	.	.	.
GC13	1	152127629	C	A	RPTN	Missense	p.W649L	11	158	7.0%	.	.	.
GC13	1	152128479	G	T	RPTN	Missense	p.H366N	9	172	5.2%	.	.	.
GC13	1	152192031	C	A	HRNR	Missense	p.G692W	7	80	8.8%	.	.	.
GC13	1	152323388	C	A	FLG2	Missense	p.G2292W	11	138	8.0%	.	.	.
GC13	1	152325271	C	A	FLG2	Missense	p.G1664V	10	129	7.8%	.	.	.
GC13	1	152326373	C	A	FLG2	Nonsense	p.G1297X	12	163	7.4%	.	.	.
GC13	1	152382293	G	T	CRNN	Missense	p.P422Q	10	137	7.3%	.	.	.
GC13	1	152383038	G	T	CRNN	Missense	p.Q174K	8	128	6.3%	.	.	.
GC13	1	152733152	G	T	KPRP	Missense	p.W363L	9	101	8.9%	.	.	.

GC13	1	152883190	C	A	IVL	Missense	p.P306Q	8	74	10.8%	.	.	.
GC13	1	153271655	G	T	PGLYRP3	Missense	p.Q261K	10	104	9.6%	.	.	.
GC13	1	153277453	G	T	PGLYRP3	Missense	p.L116M	7	56	12.5%	.	O	.
GC13	1	153516260	G	T	S100A4	Missense	p.P94Q	9	117	7.7%	.	.	.
GC13	1	153631634	G	T	SNAPIN	Missense	p.R55L	10	150	6.7%	.	.	.
GC13	1	153659675	G	T	NPR1	Splicing	c.1936-1G>T	9	116	7.8%	.	.	.
GC13	1	153742689	G	T	INTS3	Missense	p.W802L	9	65	13.8%	.	.	.
GC13	1	153785881	G	T	GATAD2B	Missense	p.Q422K	10	157	6.4%	.	.	.
GC13	1	153905433	C	A	DENND4B	Missense	p.W1176L	9	137	6.6%	.	.	.
GC13	1	153923907	C	A	CRTC2	Missense	p.L411F	7	98	7.1%	.	.	.
GC13	1	153925060	C	A	CRTC2	Missense	p.W230L	11	138	8.0%	.	.	.
GC13	1	153932669	G	T	SLC39A1	Missense	p.Q294K	8	110	7.3%	.	.	.
GC13	1	153946401	C	A	CREB3L4	Missense	p.Q350K	9	114	7.9%	.	.	.
GC13	1	153991434	G	T	NUP210L	Missense	p.P1543Q	8	110	7.3%	.	.	.
GC13	1	154018643	G	T	NUP210L	Missense	p.Q1200K	9	142	6.3%	.	.	.
GC13	1	154018659	C	A	NUP210L	Missense	p.M1194I	12	155	7.7%	.	.	.
GC13	1	154042818	G	T	NUP210L	Missense	p.H829N	12	152	7.9%	.	.	.
GC13	1	154101791	G	T	NUP210L	Missense	p.P347Q	8	107	7.5%	.	.	.
GC13	1	154163700	G	T	TPM3	Missense	p.Q69K	11	178	6.2%	.	.	.
GC13	1	154184967	C	A	C1orf43	Missense	p.L106F	10	167	6.0%	.	.	.
GC13	1	154224035	G	T	UBAP2L	Missense	p.G524W	10	149	6.7%	.	.	.
GC13	1	154233391	G	T	UBAP2L	Missense	p.G868W	8	121	6.6%	.	.	.
GC13	1	154243356	G	T	UBAP2L	Splicing	c.3202-1G>T	10	166	6.0%	.	.	.
GC13	1	154246065	C	A	HAX1	Missense	p.H55N	9	131	6.9%	.	.	.
GC13	1	154300278	G	T	ATP8B2	Missense	p.M1I	9	167	5.4%	.	.	.
GC13	1	154313401	G	T	ATP8B2	Missense	p.R402L	7	94	7.4%	.	.	.
GC13	1	154315704	G	T	ATP8B2	Missense	p.M556I	9	102	8.8%	.	.	.
GC13	1	154316964	G	T	ATP8B2	Missense	p.R743L	8	125	6.4%	.	.	.
GC13	1	154318848	G	T	ATP8B2	Missense	p.G1007W	8	121	6.6%	.	.	.
GC13	1	154318870	G	T	ATP8B2	Missense	p.R1014L	11	125	8.8%	.	.	.
GC13	1	154318878	G	T	ATP8B2	Missense	p.G1017C	9	134	6.7%	.	.	.

GC13	1	154407598	G	T	IL6R	Missense	p.R258L	8	123	6.5%	.	.	.
GC13	1	154479737	C	A	TDRD10	Missense	p.P8H	9	135	6.7%	.	.	.
GC13	1	154523414	C	A	UBE2Q1	Missense	p.G413C	7	130	5.4%	.	.	.
GC13	1	154543907	G	T	CHRNB2	Missense	p.W203L	7	89	7.9%	.	.	.
GC13	1	154557338	C	A	ADAR	Missense	p.G914W	9	111	8.1%	.	O	.
GC13	1	154561939	C	A	ADAR	Splicing	c.1784-1G>T;NM_015841	9	112	8.0%	.	.	.
GC13	1	154570359	C	A	ADAR	Missense	p.G332W	7	82	8.5%	.	.	.
GC13	1	154574310	G	T	ADAR	Missense	p.P270T	10	165	6.1%	.	.	.
GC13	1	154687451	C	A	KCNN3	Missense	p.R577L	9	145	6.2%	.	.	.
GC13	1	154744557	G	T	KCNN3	Missense	p.R448S	9	167	5.4%	.	.	.
GC13	1	154744587	C	A	KCNN3	Missense	p.G438W	8	138	5.8%	.	.	.
GC13	1	154918757	G	T	PBXIP1	Missense	p.H310N	10	138	7.2%	.	.	.
GC13	1	154920155	G	T	PBXIP1	Missense	p.P79Q	9	77	11.7%	.	.	.
GC13	1	154936351	C	A	SHC1	Missense	p.M561I	9	140	6.4%	.	.	.
GC13	1	154938171	C	A	SHC1	Missense	p.G492W	8	89	9.0%	.	.	.
GC13	1	154938871	C	A	SHC1	Missense	p.R369L	6	84	7.1%	.	.	.
GC13	1	154960580	G	T	FLAD1	Missense	p.Q25H	10	159	6.3%	.	.	.
GC13	1	154989046	G	T	ZBTB7B	Missense	p.W502L	10	105	9.5%	.	.	.
GC13	1	155030568	G	T	ADAM15	Missense	p.R563L	8	126	6.3%	.	.	.
GC13	1	155057629	C	A	EFNA3	Missense	p.P64Q	9	143	6.3%	.	.	.
GC13	1	155109344	G	T	SLC50A1	Missense	p.G21W	10	102	9.8%	.	.	.
GC13	1	155110545	G	T	SLC50A1	Missense	p.G70W	11	143	7.7%	.	.	.
GC13	1	155112458	G	T	DPM3	Missense	p.R117S	6	90	6.7%	.	.	.
GC13	1	155148381	G	T	TRIM46	Missense	p.G92W	10	123	8.1%	.	.	.
GC13	1	155160639	C	A	MUC1	Missense	p.M85I	8	129	6.2%	.	.	.
GC13	1	155160752	C	A	MUC1	Missense	p.G66W	9	122	7.4%	.	.	.
GC13	1	155168335	G	T	THBS3	Missense	p.Q527K	9	116	7.8%	.	.	.
GC13	1	155172731	G	T	THBS3	Missense	p.H157N	7	100	7.0%	.	.	.
GC13	1	155205565	C	A	GBA	Missense	p.W345L	8	111	7.2%	.	.	.
GC13	1	155292262	C	A	RUSC1	Missense	p.P233Q	12	128	9.4%	.	.	.
GC13	1	155313185	C	A	ASH1L	Missense	p.R2738L	9	126	7.1%	.	.	.

GC13	1	155451799	C	A	ASH1L	Missense	p.G288W	12	212	5.7%	.	.	.
GC13	1	155582305	G	T	MSTO1	Missense	p.G304W	14	213	6.6%	.	.	.
GC13	1	155629519	G	T	YY1AP1	Missense	p.P717T	10	195	5.1%	.	.	.
GC13	1	155695202	G	T	DAP3	Missense	p.M66I	8	131	6.1%	.	.	.
GC13	1	155697408	G	T	DAP3	Missense	p.W127L	14	207	6.8%	.	.	.
GC13	1	155697423	G	T	DAP3	Missense	p.R132L	13	211	6.2%	.	.	.
GC13	1	155723043	G	T	GON4L	Missense	p.Q1932K	7	93	7.5%	.	.	.
GC13	1	155734981	G	T	GON4L	Missense	p.P1428Q	12	176	6.8%	.	.	.
GC13	1	155735183	G	T	GON4L	Missense	p.P1361T	14	160	8.8%	.	.	.
GC13	1	155747487	G	T	GON4L	Missense	p.Q673K	8	106	7.5%	.	.	.
GC13	1	155838347	G	T	SYT11	Missense	p.R209L	7	132	5.3%	.	.	.
GC13	1	155880264	G	T	RIT1	Missense	p.P11Q	11	169	6.5%	.	.	.
GC13	1	155880533	G	T	RIT1	Missense	p.P24Q	9	144	6.3%	.	.	.
GC13	1	155934779	C	A	ARHGEF2	Nonsense	p.E242X	9	170	5.3%	.	.	.
GC13	1	155979422	C	A	SSR2	Missense	p.G154V	5	36	13.9%	.	.	.
GC13	1	156040047	G	T	RAB25	Missense	p.G204V	11	143	7.7%	.	.	.
GC13	1	156046892	C	A	MEX3A	Nonsense	p.G346X	9	148	6.1%	.	.	.
GC13	1	156084974	C	A	LMNA	Missense	p.R89S	9	129	7.0%	.	O	.
GC13	1	156130236	G	T	SEMA4A	Missense	p.G59W	9	72	12.5%	.	.	.
GC13	1	156216034	C	A	PAQR6	Missense	p.W20L	8	90	8.9%	.	.	.
GC13	1	156264306	C	A	GLMP	Missense	p.L57F	7	100	7.0%	.	.	.
GC13	1	156280395	C	A	CCT3	Missense	p.W458L	8	121	6.6%	.	.	.
GC13	1	156304553	G	T	CCT3	Missense	p.P53Q	13	193	6.7%	.	.	.
GC13	1	156316721	C	A	TSACC	Missense	p.P109Q	9	162	5.6%	.	.	.
GC13	1	156376958	C	A	C1orf61	Missense	p.G113W	7	94	7.4%	.	.	.
GC13	1	156377662	C	A	C1orf61	Missense	p.D76Y	6	74	8.1%	.	.	.
GC13	1	156437843	G	T	MEF2D	Missense	p.P492Q	8	131	6.1%	.	.	.
GC13	1	156438758	C	A	MEF2D	Missense	p.G347V	8	73	11.0%	.	.	.
GC13	1	156450646	C	A	MEF2D	Missense	p.G126W	6	92	6.5%	.	.	.
GC13	1	156498840	C	A	IQGAP3	Missense	p.R1480L	5	53	9.4%	.	O	.
GC13	1	156508701	G	T	IQGAP3	Missense	p.Q1061K	9	138	6.5%	.	.	.

GC13	1	156508784	G	T	IQGAP3	Missense	p.P1033Q	11	160	6.9%	.	O	.
GC13	1	156509770	G	T	IQGAP3	Missense	p.H918N	8	92	8.7%	.	.	.
GC13	1	156533567	C	A	IQGAP3	Missense	p.G163W	9	113	8.0%	.	.	.
GC13	1	156536300	G	T	IQGAP3	Missense	p.P55Q	9	138	6.5%	.	.	.
GC13	1	156536301	G	T	IQGAP3	Missense	p.P55T	10	136	7.4%	.	.	.
GC13	1	156551679	C	A	TTC24	Missense	p.H175N	11	133	8.3%	.	.	.
GC13	1	156565124	G	T	GPATCH4	Missense	p.P337T	10	189	5.3%	.	.	.
GC13	1	156566185	G	T	GPATCH4	Missense	p.P145Q	10	135	7.4%	.	.	.
GC13	1	156568808	C	A	GPATCH4	Missense	p.G16W	7	82	8.5%	.	.	.
GC13	1	156639623	G	T	NES	Missense	p.Q1453K	10	132	7.6%	.	.	.
GC13	1	156640954	G	T	NES	Missense	p.P1009Q	10	106	9.4%	.	.	.
GC13	1	156642166	G	T	NES	Missense	p.P605H	9	138	6.5%	.	.	.
GC13	1	156693178	C	A	ISG20L2	Missense	p.W342L	9	88	10.2%	.	.	.
GC13	1	156698932	G	T	RRNAD1	Missense	p.E12D	9	139	6.5%	.	.	.
GC13	1	156702813	C	A	RRNAD1	Missense	p.Q156K	7	98	7.1%	.	.	.
GC13	1	156721151	G	T	HDGF	Missense	p.P24Q	7	84	8.3%	.	.	.
GC13	1	156811536	C	A	INSRR	Missense	p.G1150W	9	131	6.9%	.	.	.
GC13	1	156812801	G	T	INSRR	Missense	p.H1041N	10	164	6.1%	.	.	.
GC13	1	156874616	G	T	PEAR1	Nonsense	p.E60X	7	87	8.0%	.	.	.
GC13	1	156880487	G	T	PEAR1	Missense	p.G635W	12	167	7.2%	.	.	.
GC13	1	156880532	C	A	PEAR1	Missense	p.Q650K	9	143	6.3%	.	.	.
GC13	1	156916729	C	A	ARHGEF11	Missense	p.R816L	6	90	6.7%	.	.	.
GC13	1	157103927	G	T	ETV3	Missense	p.P126Q	10	188	5.3%	.	.	.
GC13	1	157516853	C	A	FCRL5	Missense	p.G63W	12	113	10.6%	.	.	.
GC13	1	157557753	G	T	FCRL4	Missense	p.P155Q	9	122	7.4%	.	.	.
GC13	1	157804517	G	T	CD5L	Missense	p.P133Q	7	64	10.9%	.	O	.
GC13	1	158047910	G	T	KIRREL	Missense	p.R111L	7	136	5.1%	.	.	.
GC13	1	158227286	G	T	CD1A	Missense	p.W309L	8	106	7.5%	.	.	.
GC13	1	158261152	G	T	CD1C	Missense	p.R97L	6	60	10.0%	.	.	.
GC13	1	158298030	G	T	CD1B	Missense	p.P333Q	10	168	6.0%	.	.	.
GC13	1	158326569	G	T	CD1E	Missense	p.M149I	10	206	4.9%	.	.	.

GC13	1	158450072	G	T	OR10R2	Missense	p.L135F	12	184	6.5%	.	.	.
GC13	1	158517290	C	A	OR6Y1	Missense	p.M202I	8	116	6.9%	.	.	.
GC13	1	158549504	C	A	OR10X1	Missense	p.M62I	7	94	7.4%	.	.	.
GC13	1	158614045	G	T	SPTA1	Missense	p.Q1446K	9	133	6.8%	.	.	.
GC13	1	158669562	C	A	OR6K2	Missense	p.R294M	9	165	5.5%	.	.	.
GC13	1	158735716	C	A	OR6N1	Missense	p.G253W	11	125	8.8%	.	.	.
GC13	1	158746668	C	A	OR6N2	Missense	p.G253V	8	116	6.9%	.	O	.
GC13	1	158747105	C	A	OR6N2	Missense	p.L107F	8	112	7.1%	.	O	.
GC13	1	158984473	G	T	IFI16	Missense	p.M1I	9	155	5.8%	.	.	.
GC13	1	158988045	G	T	IFI16	Missense	p.Q136H	10	156	6.4%	.	.	.
GC13	1	159023470	G	T	IFI16	Missense	p.G689W	9	98	9.2%	.	.	.
GC13	1	159163258	G	T	CADM3	Missense	p.R143L	9	156	5.8%	.	O	.
GC13	1	159163296	G	T	CADM3	Missense	p.G156W	10	157	6.4%	.	.	.
GC13	1	159174769	G	T	ACKR1	Missense	p.R7M	8	133	6.0%	.	.	.
GC13	1	159283534	C	A	OR10J3	Missense	p.G306W	10	171	5.8%	.	.	.
GC13	1	159409817	C	A	OR10J1	Missense	p.P90Q	8	143	5.6%	.	.	.
GC13	1	159779401	G	T	FCRL6	Missense	p.G272W	8	112	7.1%	.	O	.
GC13	1	159802711	G	T	SLAMF8	Missense	p.R29M	11	142	7.7%	.	.	.
GC13	1	159860291	C	A	CFAP45	Missense	p.R84L	9	118	7.6%	.	.	.
GC13	1	159897275	G	T	IGSF9	Missense	p.H1134N	9	115	7.8%	.	.	.
GC13	1	159898677	G	T	IGSF9	Missense	p.P834Q	7	68	10.3%	.	.	.
GC13	1	159900050	G	T	IGSF9	Missense	p.Q665K	8	102	7.8%	.	.	.
GC13	1	160000778	C	A	PIGM	Missense	p.W251L	8	120	6.7%	.	.	.
GC13	1	160011506	C	A	KCNJ10	Missense	p.G273C	9	144	6.3%	.	.	.
GC13	1	160109502	G	T	ATP1A2	Missense	p.M971I	8	108	7.4%	.	.	.
GC13	1	160136824	G	T	ATP1A4	Missense	p.R438L	7	127	5.5%	.	.	.
GC13	1	160160663	C	A	CASQ1	Missense	p.P41H	10	91	11.0%	.	.	.
GC13	1	160160683	C	A	CASQ1	Missense	p.R48S	8	101	7.9%	.	.	.
GC13	1	160201121	G	T	DCAF8	Missense	p.Q346K	8	110	7.3%	.	.	.
GC13	1	160201124	G	T	DCAF8	Missense	p.H345N	8	111	7.2%	.	.	.
GC13	1	160252275	G	T	PEX19	Missense	p.Q122K	11	163	6.7%	.	.	.

GC13	1	160261151	C	A	COPA	Missense	p.G1141W	6	44	13.6%	.	.	.
GC13	1	160263020	C	A	COPA	Missense	p.W929L	10	175	5.7%	.	.	.
GC13	1	160269047	C	A	COPA	Missense	p.G568W	4	26	15.4%	.	.	.
GC13	1	160535491	C	A	CD84	Missense	p.G31W	8	140	5.7%	.	.	.
GC13	1	160718161	G	T	SLAMF7	Missense	p.R78M	9	146	6.2%	.	.	.
GC13	1	160719791	G	T	SLAMF7	Missense	p.W79L	7	95	7.4%	.	.	.
GC13	1	160719883	G	T	SLAMF7	Nonsense	p.E110X	10	133	7.5%	.	.	.
GC13	1	160769764	C	A	LY9	Missense	p.L116M	9	156	5.8%	.	.	.
GC13	1	160793480	C	A	LY9	Missense	p.P485Q	8	130	6.2%	.	.	.
GC13	1	160811258	G	T	CD244	Missense	p.R138S	9	163	5.5%	.	.	.
GC13	1	161018937	G	T	ARHGAP30	Missense	p.P448Q	12	120	10.0%	.	.	.
GC13	1	161019272	G	T	ARHGAP30	Missense	p.P366H	9	115	7.8%	.	.	.
GC13	1	161043533	G	T	NECTIN4	Missense	p.H404N	8	99	8.1%	.	.	.
GC13	1	161071912	G	T	PFDN2	Missense	p.R72S	10	161	6.2%	.	.	.
GC13	1	161090018	G	T	NIT1	Missense	p.W223L	12	122	9.8%	.	.	.
GC13	1	161092178	C	A	DEDD	Missense	p.R239L	9	143	6.3%	.	.	.
GC13	1	161092301	C	A	DEDD	Missense	p.R198L	14	169	8.3%	.	.	.
GC13	1	161092925	C	A	DEDD	Missense	p.G174W	8	94	8.5%	.	.	.
GC13	1	161093629	C	A	DEDD	Missense	p.V145L	6	36	16.7%	.	O	.
GC13	1	161130639	G	T	USP21	Missense	p.R99L	8	136	5.9%	.	.	.
GC13	1	161134933	G	T	USP21	Missense	p.R565L	12	144	8.3%	.	.	.
GC13	1	161141836	C	A	B4GALT3	Missense	p.G318W	8	138	5.8%	.	.	.
GC13	1	161141857	G	T	B4GALT3	Missense	p.Q311K	8	136	5.9%	.	.	.
GC13	1	161197770	G	T	TOMM40L	Missense	p.G94W	8	127	6.3%	.	.	.
GC13	1	161199655	C	A	NR1I3	Missense	p.G259W	6	74	8.1%	.	.	.
GC13	1	161206345	C	A	NR1I3	Missense	p.R4M	6	61	9.8%	.	.	.
GC13	1	161335316	C	A	CFAP126	Missense	p.K116N	9	150	6.0%	.	.	.
GC13	1	161692790	G	T	FCRLB	Missense	p.G15W	14	151	9.3%	.	.	.
GC13	1	161722932	C	A	DUSP12	Missense	p.H248N	9	137	6.6%	.	.	.
GC13	1	161993126	G	T	OLFML2B	Missense	p.P32Q	8	147	5.4%	.	.	.
GC13	1	162344396	C	A	SPATA46	Missense	p.L76F	9	125	7.2%	.	.	.

GC13	1	162536127	G	T	UAP1	Missense	p.W90L	8	103	7.8%	.	.	.
GC13	1	162724599	G	T	DDR2	Missense	p.R124L	9	108	8.3%	O	O	.
GC13	1	162740194	G	T	DDR2	Missense	p.G466W	10	160	6.3%	O	.	.
GC13	1	162748419	G	T	DDR2	Missense	p.W778L	9	152	5.9%	O	.	.
GC13	1	163298695	C	A	NUF2	Missense	p.P112Q	8	138	5.8%	.	.	.
GC13	1	164532494	C	A	PBX1	Missense	p.H71N	10	192	5.2%	.	.	.
GC13	1	165183012	G	T	LMX1A	Missense	p.H179N	9	123	7.3%	.	.	.
GC13	1	165370562	C	A	RXRG	Missense	p.G321W	9	81	11.1%	.	O	.
GC13	1	165721356	C	A	TMCO1	Missense	p.M119I	9	125	7.2%	.	.	.
GC13	1	165797149	G	T	UCK2	Missense	p.G27W	6	41	14.6%	.	.	.
GC13	1	166818283	G	T	POGK	Missense	p.R38L	8	142	5.6%	.	.	.
GC13	1	166818696	C	A	POGK	Missense	p.Q176K	8	116	6.9%	.	.	.
GC13	1	166826949	C	A	TADA1	Missense	p.R288M	10	174	5.7%	.	.	.
GC13	1	167095998	C	A	DUSP27	Missense	p.L544M	8	104	7.7%	.	.	.
GC13	1	167096157	C	A	DUSP27	Missense	p.Q597K	7	98	7.1%	.	.	.
GC13	1	167096331	G	T	DUSP27	Missense	p.G655W	6	87	6.9%	.	.	.
GC13	1	167097723	C	A	DUSP27	Missense	p.Q1119K	10	174	5.7%	.	.	.
GC13	1	167381381	G	T	POU2F1	Missense	p.G518W	8	146	5.5%	.	.	.
GC13	1	167384916	G	T	POU2F1	Missense	p.G661W	9	149	6.0%	.	.	.
GC13	1	167805547	C	A	ADCY10	Missense	p.M950I	7	81	8.6%	.	.	.
GC13	1	167962517	C	A	DCAF6	Missense	p.H217N	9	107	8.4%	.	.	.
GC13	1	168065808	C	A	GPR161	Missense	p.R268L	9	149	6.0%	.	.	.
GC13	1	168066133	C	A	GPR161	Missense	p.G160W	11	129	8.5%	.	.	.
GC13	1	168153186	G	T	TIPRL	Nonsense	p.G51X	9	147	6.1%	.	.	.
GC13	1	168206008	G	T	SFT2D2	Missense	p.W138L	10	196	5.1%	.	O	.
GC13	1	168260510	G	T	TBX19	Missense	p.G106W	8	132	6.1%	.	.	.
GC13	1	168282085	C	A	TBX19	Missense	p.Q398K	8	119	6.7%	.	.	.
GC13	1	169349783	G	T	BLZF1	Missense	p.G245W	8	169	4.7%	.	.	.
GC13	1	169390726	C	A	CCDC181	Missense	p.G315W	10	151	6.6%	.	.	.
GC13	1	169390732	C	A	CCDC181	Missense	p.G313W	11	162	6.8%	.	.	.
GC13	1	169391521	G	T	CCDC181	Missense	p.Q50K	11	187	5.9%	.	.	.

GC13	1	169511383	C	A	F5	Missense	p.W982L	9	163	5.5%	.	.	.
GC13	1	169512271	G	T	F5	Missense	p.P686Q	12	163	7.4%	.	.	.
GC13	1	169772384	G	T	C1orf112	Missense	p.L140F	8	141	5.7%	.	.	.
GC13	1	169831884	C	A	SCYL3	Missense	p.R337L	8	105	7.6%	.	.	.
GC13	1	169947269	C	A	KIFAP3	Missense	p.M574I	10	170	5.9%	.	.	.
GC13	1	169985693	G	T	KIFAP3	Missense	p.L321I	9	145	6.2%	.	.	.
GC13	1	170115259	G	T	METTL11B	Missense	p.R4L	7	117	6.0%	.	.	.
GC13	1	170136723	G	T	METTL11B	Missense	p.R226L	8	147	5.4%	.	.	.
GC13	1	171073057	G	T	FMO3	Missense	p.Q88H	12	138	8.7%	.	.	.
GC13	1	171083265	G	T	FMO3	Missense	p.G253W	10	155	6.5%	.	.	.
GC13	1	171254526	G	T	FMO1	Missense	p.W485L	10	148	6.8%	.	.	.
GC13	1	171310603	G	T	FMO4	Missense	p.M434I	8	130	6.2%	.	.	.
GC13	1	171310677	G	T	FMO4	Missense	p.W459L	12	133	9.0%	.	.	.
GC13	1	171310854	G	T	FMO4	Missense	p.W518L	7	95	7.4%	.	.	.
GC13	1	171486802	C	A	PRRC2C	Missense	p.P198H	8	108	7.4%	.	.	.
GC13	1	171505323	G	T	PRRC2C	Missense	p.M731I	10	154	6.5%	.	.	.
GC13	1	171509981	C	A	PRRC2C	Missense	p.Q1124K	10	142	7.0%	.	.	.
GC13	1	171510161	G	T	PRRC2C	Nonsense	p.G1184X	19	148	12.8%	.	.	.
GC13	1	171511098	G	T	PRRC2C	Missense	p.W1496L	11	175	6.3%	.	.	.
GC13	1	171526463	G	T	PRRC2C	Missense	p.G1736W	9	169	5.3%	.	.	.
GC13	1	171560763	G	T	PRRC2C	Missense	p.R2744L	9	170	5.3%	.	.	.
GC13	1	171605098	C	A	MYOC	Missense	p.M494I	9	153	5.9%	.	.	.
GC13	1	171756912	G	T	METTL13	Missense	p.R228L	8	121	6.6%	.	.	.
GC13	1	171761303	C	A	METTL13	Missense	p.Q385K	7	96	7.3%	.	.	.
GC13	1	172002372	G	T	DNM3	Missense	p.M272I	8	108	7.4%	.	.	.
GC13	1	172007513	G	T	DNM3	Nonsense	p.G302X	9	125	7.2%	.	.	.
GC13	1	172062835	G	T	DNM3	Splicing	c.1546-1G>T;NM_001350204	10	160	6.3%	.	.	.
GC13	1	172410915	C	A	PIGC	Missense	p.W283L	11	179	6.1%	.	.	.
GC13	1	172501669	G	T	SUCO	Missense	p.W19C	11	145	7.6%	.	.	.
GC13	1	172526894	C	A	SUCO	Missense	p.P194Q	9	153	5.9%	.	.	.
GC13	1	173446558	G	T	PRDX6	Missense	p.G8W	9	175	5.1%	.	.	.

GC13	1	173474413	G	T	SLC9C2	Missense	p.Q1095K	9	160	5.6%	.	.	.
GC13	1	173556887	C	A	SLC9C2	Missense	p.W147L	12	139	8.6%	.	.	.
GC13	1	173567082	C	A	SLC9C2	Missense	p.L106F	10	133	7.5%	.	.	.
GC13	1	173593928	G	T	ANKRD45	Missense	p.P243Q	7	78	9.0%	.	.	.
GC13	1	173628329	C	A	ANKRD45	Missense	p.G77W	16	228	7.0%	.	.	.
GC13	1	173703216	C	A	KLHL20	Missense	p.Q130K	12	146	8.2%	.	.	.
GC13	1	173725160	G	T	KLHL20	Missense	p.G317V	11	152	7.2%	.	.	.
GC13	1	173754374	C	A	KLHL20	Missense	p.H607N	11	138	8.0%	.	.	.
GC13	1	173822581	G	T	DARS2	Missense	p.W480L	8	101	7.9%	.	.	.
GC13	1	173883999	C	A	SERPINC1	Missense	p.G34W	6	83	7.2%	.	.	.
GC13	1	174418121	G	T	GPR52	Missense	p.R291L	8	142	5.6%	.	.	.
GC13	1	174671361	G	T	RABGAP1L	Missense	p.M712I	9	175	5.1%	.	.	.
GC13	1	174973969	G	T	CACYBP	Nonsense	p.G36X	10	171	5.8%	.	.	.
GC13	1	174976265	G	T	CACYBP	Missense	p.G79W	10	100	10.0%	.	.	.
GC13	1	175049409	G	T	TNN	Missense	p.G299W	9	97	9.3%	.	.	.
GC13	1	175087766	C	A	TNN	Missense	p.P819Q	7	128	5.5%	.	.	.
GC13	1	175087924	C	A	TNN	Missense	p.Q872K	6	71	8.5%	.	.	.
GC13	1	175362968	C	A	TNR	Missense	p.W102L	8	92	8.7%	.	O	.
GC13	1	175372638	G	T	TNR	Missense	p.P205Q	8	130	6.2%	.	.	.
GC13	1	176526363	C	A	PAPPA2	Missense	p.P302Q	6	69	8.7%	.	.	.
GC13	1	176709227	G	T	PAPPA2	Missense	p.R1349L	13	180	7.2%	.	.	.
GC13	1	176833511	C	A	ASTN1	Missense	p.W1265L	10	106	9.4%	.	.	.
GC13	1	177250179	C	A	BRINP2	Missense	p.Q623K	11	95	11.6%	.	.	.
GC13	1	177905471	G	T	SEC16B	Missense	p.Q845K	8	114	7.0%	.	.	.
GC13	1	178423758	G	T	RASAL2	Missense	p.W581L	9	136	6.6%	.	.	.
GC13	1	178423772	C	A	RASAL2	Missense	p.Q586K	9	128	7.0%	.	.	.
GC13	1	178427099	G	T	RASAL2	Missense	p.R750L	9	143	6.3%	.	.	.
GC13	1	178490397	G	T	TEX35	Missense	p.R203L	9	159	5.7%	.	.	.
GC13	1	178871281	G	T	RALGPS2	Missense	p.W496L	10	175	5.7%	.	.	.
GC13	1	179033082	G	T	FAM20B	Missense	p.G199W	11	122	9.0%	.	.	.
GC13	1	179041053	G	T	FAM20B	Missense	p.R335L	5	44	11.4%	.	.	.

GC13	1	179041229	G	T	FAM20B	Missense	p.G394W	12	161	7.5%	.	.	.
GC13	1	179078453	C	A	ABL2	Missense	p.G629V	8	107	7.5%	.	.	.
GC13	1	179079573	C	A	ABL2	Missense	p.G536W	10	124	8.1%	.	.	.
GC13	1	179600010	C	A	TDRD5	Missense	p.Q361K	8	114	7.0%	.	.	.
GC13	1	179603691	C	A	TDRD5	Missense	p.P409H	9	116	7.8%	.	.	.
GC13	1	179604857	C	A	TDRD5	Missense	p.P7Q	7	143	4.9%	.	.	.
GC13	1	179604922	G	T	TDRD5	Missense	p.G29W	10	169	5.9%	.	.	.
GC13	1	179609564	G	T	TDRD5	Missense	p.W150L	10	101	9.9%	.	.	.
GC13	1	179638376	G	T	TDRD5	Missense	p.L400F	12	140	8.6%	.	.	.
GC13	1	179815290	C	A	TOR1AIP2	Missense	p.L443F	8	118	6.8%	.	.	.
GC13	1	179886826	C	A	TOR1AIP1	Missense	p.H403N	10	193	5.2%	.	.	.
GC13	1	179887180	G	T	TOR1AIP1	Nonsense	p.G521X	9	173	5.2%	.	.	.
GC13	1	179955351	C	A	CEP350	Missense	p.P12Q	10	134	7.5%	.	.	.
GC13	1	179961295	C	A	CEP350	Missense	p.L112M	9	102	8.8%	.	.	.
GC13	1	180003012	G	T	CEP350	Missense	p.K1247N	10	151	6.6%	.	.	.
GC13	1	180010837	G	T	CEP350	Missense	p.R1421L	7	103	6.8%	.	.	.
GC13	1	180044288	G	T	CEP350	Missense	p.W1900L	7	91	7.7%	.	.	.
GC13	1	180062687	C	A	CEP350	Missense	p.P2483T	10	153	6.5%	.	.	.
GC13	1	180155259	C	A	QSOX1	Missense	p.P320Q	6	80	7.5%	.	.	.
GC13	1	180843035	G	T	XPR1	Missense	p.G524W	8	134	6.0%	.	.	.
GC13	1	180885687	G	T	KIAA1614	Missense	p.G150W	7	92	7.6%	.	.	.
GC13	1	180904879	C	A	KIAA1614	Missense	p.L612M	9	128	7.0%	.	O	.
GC13	1	181058858	G	T	IER5	Missense	p.G274C	6	95	6.3%	.	.	.
GC13	1	181480552	G	T	CACNA1E	Missense	p.G140W	8	102	7.8%	.	.	.
GC13	1	181686273	G	T	CACNA1E	Missense	p.G454W	12	167	7.2%	.	.	.
GC13	1	181745289	G	T	CACNA1E	Missense	p.R1712L	11	163	6.7%	.	.	.
GC13	1	182026236	C	A	ZNF648	Nonsense	p.E304X	5	64	7.8%	.	.	.
GC13	1	182026796	G	T	ZNF648	Missense	p.P117Q	6	94	6.4%	.	.	.
GC13	1	182369470	C	A	TEDDM1	Missense	p.G51W	8	140	5.7%	.	.	.
GC13	1	182443400	G	T	RGSL1	Missense	p.R385L	12	133	9.0%	.	.	.
GC13	1	182545464	G	T	RNASEL	Missense	p.Q656K	8	126	6.3%	.	.	.

GC13	1	182554636	G	T	RNASEL	Missense	p.L436I	11	122	9.0%	.	.	.
GC13	1	182554734	C	A	RNASEL	Missense	p.R403L	7	98	7.1%	.	.	.
GC13	1	182617421	G	T	RGS8	Missense	p.R89S	8	158	5.1%	.	.	.
GC13	1	182775509	G	T	NPL	Missense	p.G6W	6	70	8.6%	.	.	.
GC13	1	182823291	C	A	DHX9	Missense	p.Q202K	9	104	8.7%	.	.	.
GC13	1	182845591	G	T	DHX9	Missense	p.R680L	9	166	5.4%	.	.	.
GC13	1	182847212	G	T	DHX9	Missense	p.W752L	11	133	8.3%	.	.	.
GC13	1	183087263	G	T	LAMC1	Missense	p.G658W	10	147	6.8%	.	.	.
GC13	1	183111885	C	A	LAMC1	Missense	p.P1597Q	8	134	6.0%	.	.	.
GC13	1	183184707	C	A	LAMC2	Missense	p.Q130K	5	43	11.6%	.	.	.
GC13	1	183196665	G	T	LAMC2	Missense	p.G434V	10	159	6.3%	.	.	.
GC13	1	183197625	G	T	LAMC2	Missense	p.G529W	8	85	9.4%	.	.	.
GC13	1	183208512	G	T	LAMC2	Missense	p.Q961H	9	165	5.5%	.	.	.
GC13	1	183209472	G	T	LAMC2	Missense	p.G1092W	9	141	6.4%	.	.	.
GC13	1	183495791	C	A	SMG7	Missense	p.Q83K	9	161	5.6%	.	.	.
GC13	1	183506323	C	A	SMG7	Missense	p.H361N	8	146	5.5%	.	.	.
GC13	1	183513497	C	A	SMG7	Missense	p.Q576K	9	157	5.7%	.	.	.
GC13	1	183514411	G	T	SMG7	Missense	p.L736F	12	205	5.9%	.	.	.
GC13	1	183515342	G	T	SMG7	Missense	p.W829L	8	101	7.9%	.	.	.
GC13	1	183518938	G	T	SMG7	Missense	p.G963W	8	89	9.0%	.	.	.
GC13	1	183525314	G	T	NCF2	Missense	p.P426H	13	195	6.7%	.	.	.
GC13	1	183617652	C	A	APOBEC4	Missense	p.G89W	11	166	6.6%	.	O	.
GC13	1	183617784	G	T	APOBEC4	Missense	p.L45I	10	174	5.7%	.	.	.
GC13	1	183835196	G	T	RGL1	Missense	p.M136I	9	159	5.7%	.	.	.
GC13	1	183854007	G	T	RGL1	Missense	p.G294W	8	133	6.0%	.	.	.
GC13	1	183891389	C	A	RGL1	Missense	p.Q651K	11	167	6.6%	.	.	.
GC13	1	183908043	C	A	COLGALT2	Missense	p.W458L	9	76	11.8%	.	.	.
GC13	1	183944256	C	A	COLGALT2	Missense	p.R156M	8	98	8.2%	.	.	.
GC13	1	184675810	G	T	EDEM3	Missense	p.Q724K	8	100	8.0%	.	.	.
GC13	1	184680971	C	A	EDEM3	Missense	p.W526L	8	133	6.0%	.	.	.
GC13	1	184688642	G	T	EDEM3	Missense	p.H338N	11	178	6.2%	.	.	.

GC13	1	184702008	G	T	EDEM3	Missense	p.P192Q	9	140	6.4%	.	.	.
GC13	1	184706750	C	A	EDEM3	Missense	p.L100F	9	117	7.7%	.	.	.
GC13	1	184764133	G	T	FAM129A	Missense	p.P922Q	9	169	5.3%	.	.	.
GC13	1	184764196	G	T	FAM129A	Missense	p.P901Q	8	155	5.2%	.	O	.
GC13	1	184764970	G	T	FAM129A	Missense	p.P643H	7	84	8.3%	.	.	.
GC13	1	184777252	G	T	FAM129A	Missense	p.H431N	9	145	6.2%	.	.	.
GC13	1	185089333	C	A	TRMT1L	Missense	p.G518C	8	126	6.3%	.	.	.
GC13	1	185113056	G	T	TRMT1L	Missense	p.P98H	8	137	5.8%	.	.	.
GC13	1	185135725	C	A	SWT1	Missense	p.P36T	11	234	4.7%	.	O	.
GC13	1	185276710	G	T	IVNS1ABP	Missense	p.R148S	8	156	5.1%	.	.	.
GC13	1	185951521	G	T	HMCN1	Missense	p.M930I	8	142	5.6%	.	.	.
GC13	1	185953337	G	T	HMCN1	Missense	p.G943W	8	121	6.6%	.	.	.
GC13	1	186034447	G	T	HMCN1	Missense	p.G2531C	6	57	10.5%	.	.	.
GC13	1	186050375	C	A	HMCN1	Missense	p.P2879H	8	134	6.0%	.	.	.
GC13	1	186056373	G	T	HMCN1	Missense	p.R3024L	8	97	8.2%	.	.	.
GC13	1	186086161	C	A	HMCN1	Missense	p.P3866H	9	147	6.1%	.	.	.
GC13	1	186088957	G	T	HMCN1	Missense	p.G4013W	10	170	5.9%	.	.	.
GC13	1	186113398	C	A	HMCN1	Missense	p.P4673Q	7	96	7.3%	.	.	.
GC13	1	186143672	C	A	HMCN1	Missense	p.H5281N	9	142	6.3%	.	.	.
GC13	1	186151404	G	T	HMCN1	Nonsense	p.G5467X	8	104	7.7%	.	.	.
GC13	1	186277117	G	T	PRG4	Missense	p.G622W	8	75	10.7%	.	.	.
GC13	1	186280178	G	T	PRG4	Missense	p.W1037L	9	161	5.6%	.	.	.
GC13	1	186280232	G	T	PRG4	Missense	p.W1055L	9	155	5.8%	.	.	.
GC13	1	186413572	G	T	PDC	Missense	p.R42S	11	199	5.5%	.	.	.
GC13	1	186648553	G	T	PTGS2	Missense	p.H24N	10	96	10.4%	.	.	.
GC13	1	186839585	C	A	PLA2G4A	Missense	p.H18N	7	72	9.7%	.	.	.
GC13	1	186915905	G	T	PLA2G4A	Missense	p.M330I	6	70	8.6%	.	.	.
GC13	1	186925356	G	T	PLA2G4A	Missense	p.G427W	8	127	6.3%	.	.	.
GC13	1	190067854	C	A	BRINP3	Missense	p.R430L	8	145	5.5%	.	.	.
GC13	1	190250775	C	A	BRINP3	Missense	p.L114F	8	144	5.6%	.	.	.
GC13	1	193045635	G	T	TROVE2	Missense	p.W269L	9	129	7.0%	.	.	.

GC13	1	193094281	G	T	CDC73	Missense	p.L57F	10	178	5.6%	O	.	.
GC13	1	193150092	G	T	B3GALT2	Missense	p.L201I	10	183	5.5%	.	.	.
GC13	1	193150445	C	A	B3GALT2	Missense	p.W83L	8	129	6.2%	.	.	.
GC13	1	196309472	C	A	KCNT2	Missense	p.M544I	9	135	6.7%	.	.	.
GC13	1	196438188	C	A	KCNT2	Missense	p.W132L	11	135	8.1%	.	.	.
GC13	1	196451471	C	A	KCNT2	Missense	p.W105L	10	189	5.3%	.	.	.
GC13	1	196642210	C	A	CFH	Missense	p.P54H	8	130	6.2%	.	O	.
GC13	1	196709784	G	T	CFH	Missense	p.G940C	8	136	5.9%	.	.	.
GC13	1	196918656	C	A	CFHR2	Missense	p.Q44K	6	79	7.6%	.	O	.
GC13	1	196963350	C	A	CFHR5	Missense	p.Q191K	10	107	9.3%	.	.	.
GC13	1	197030176	C	A	F13B	Nonsense	p.G161X	7	89	7.9%	.	.	.
GC13	1	197071093	G	T	ASPM	Missense	p.L2430I	9	173	5.2%	.	.	.
GC13	1	197071451	C	A	ASPM	Missense	p.Q2310H	10	165	6.1%	.	.	.
GC13	1	197072737	G	T	ASPM	Missense	p.Q1882K	9	125	7.2%	.	.	.
GC13	1	197072740	G	T	ASPM	Missense	p.L1881I	9	127	7.1%	.	.	.
GC13	1	197094292	C	A	ASPM	Missense	p.W989L	9	164	5.5%	.	.	.
GC13	1	197097686	G	T	ASPM	Missense	p.P957Q	11	121	9.1%	.	.	.
GC13	1	197112930	C	A	ASPM	Missense	p.W151L	11	167	6.6%	.	.	.
GC13	1	197398195	G	T	CRB1	Missense	p.W854L	8	127	6.3%	.	.	.
GC13	1	197479835	G	T	DENND1B	Missense	p.Q695K	10	117	8.5%	.	.	.
GC13	1	197552341	G	T	DENND1B	Missense	p.R364S	6	76	7.9%	.	.	.
GC13	1	197704751	C	A	DENND1B	Missense	p.R6L	7	89	7.9%	.	.	.
GC13	1	197898246	G	T	LHX9	Nonsense	p.G351X	8	148	5.4%	.	.	.
GC13	1	198248120	G	T	NEK7	Missense	p.G173V	9	132	6.8%	.	.	.
GC13	1	198663263	C	A	PTPRC	Missense	p.H73N	5	25	20.0%	O	.	.
GC13	1	200017691	G	T	NR5A2	Missense	p.M213I	8	145	5.5%	.	.	.
GC13	1	200377380	G	T	ZNF281	Missense	p.P485Q	9	140	6.4%	.	.	.
GC13	1	200583527	C	A	KIF14	Missense	p.M458I	10	186	5.4%	.	.	.
GC13	1	200610371	C	A	DDX59	Missense	p.W537L	9	130	6.9%	.	.	.
GC13	1	200613569	C	A	DDX59	Missense	p.W444L	11	175	6.3%	.	.	.
GC13	1	200708958	G	T	CAMSAP2	Missense	p.M1I	7	72	9.7%	.	.	.

GC13	1	200797720	G	T	CAMSAP2	Missense	p.W222L	11	202	5.4%	.	.	.
GC13	1	200819205	C	A	CAMSAP2	Missense	p.P1087Q	10	193	5.2%	.	.	.
GC13	1	200843115	G	T	GPR25	Missense	p.R317L	6	75	8.0%	.	.	.
GC13	1	200965419	G	T	KIF21B	Missense	p.Q728K	6	66	9.1%	.	.	.
GC13	1	201054653	C	A	CACNA1S	Missense	p.R354L	7	104	6.7%	.	.	.
GC13	1	201060822	C	A	CACNA1S	Missense	p.G214W	8	111	7.2%	.	.	.
GC13	1	201175628	C	A	IGFN1	Missense	p.P536Q	6	77	7.8%	.	.	.
GC13	1	201184772	C	A	IGFN1	Missense	p.P3034H	8	49	16.3%	.	.	.
GC13	1	201190722	C	A	IGFN1	Missense	p.P3350Q	7	93	7.5%	.	.	.
GC13	1	201292267	C	A	PKP1	Missense	p.L544M	7	94	7.4%	.	.	.
GC13	1	201332429	G	T	TNNT2	Missense	p.Q184K	6	53	11.3%	.	.	.
GC13	1	201355755	G	T	LAD1	Missense	p.P245Q	9	172	5.2%	.	.	.
GC13	1	201751388	G	T	NAV1	Missense	p.R192L	9	129	7.0%	.	.	.
GC13	1	201752676	G	T	NAV1	Missense	p.G443W	9	137	6.6%	.	.	.
GC13	1	201934595	G	T	TIMM17A	Missense	p.M118I	7	93	7.5%	.	.	.
GC13	1	202123415	G	T	PTPN7	Missense	p.Q208K	7	92	7.6%	.	.	.
GC13	1	202287935	G	T	LGR6	Missense	p.R696L	9	139	6.5%	.	.	.
GC13	1	202711608	G	T	KDM5B	Missense	p.Q789K	8	110	7.3%	.	.	.
GC13	1	202715291	G	T	KDM5B	Missense	p.P681H	9	172	5.2%	.	.	.
GC13	1	202743804	C	A	KDM5B	Missense	p.Q114H	11	179	6.1%	.	.	.
GC13	1	202880267	C	A	KLHL12	Missense	p.R249L	7	87	8.0%	.	.	.
GC13	1	202931785	C	A	CYB5R1	Missense	p.R263L	6	92	6.5%	.	.	.
GC13	1	202990076	G	T	TMEM183A	Missense	p.V312F	6	63	9.5%	.	.	.
GC13	1	203025609	C	A	PPFIA4	Missense	p.P738Q	6	88	6.8%	.	.	.
GC13	1	203144514	G	T	MYBPH	Missense	p.P94Q	8	95	8.4%	.	.	.
GC13	1	203186066	G	T	CHIT1	Missense	p.P432Q	7	117	6.0%	.	.	.
GC13	1	203191406	C	A	CHIT1	Missense	p.W199L	12	121	9.9%	.	.	.
GC13	1	203274744	G	T	BTG2	Missense	p.G4W	12	149	8.1%	.	.	.
GC13	1	203274766	C	A	BTG2	Missense	p.P11Q	10	135	7.4%	.	.	.
GC13	1	203452341	C	A	PRELP	Missense	p.P10Q	9	110	8.2%	.	.	.
GC13	1	203468932	C	A	OPTC	Missense	p.R229S	5	50	10.0%	.	.	.

GC13	1	203667384	G	T	ATP2B4	Missense	p.W98L	12	110	10.9%	.	.	.
GC13	1	203691632	G	T	ATP2B4	Missense	p.G947W	8	87	9.2%	.	.	.
GC13	1	203702524	G	T	ATP2B4	Missense	p.M1161I	9	149	6.0%	.	.	.
GC13	1	203768420	G	T	ZBED6	Missense	p.M590I	18	200	9.0%	.	.	.
GC13	1	204219717	C	A	PLEKHA6	Missense	p.R517L	7	94	7.4%	.	.	.
GC13	1	204236666	C	A	PLEKHA6	Missense	p.G73W	6	83	7.2%	.	.	.
GC13	1	204378967	G	T	PPP1R15B	Missense	p.Q525K	10	166	6.0%	.	.	.
GC13	1	204379512	G	T	PPP1R15B	Missense	p.P343Q	10	144	6.9%	.	.	.
GC13	1	204379947	C	A	PPP1R15B	Missense	p.R198L	14	177	7.9%	.	.	.
GC13	1	204380364	C	A	PPP1R15B	Missense	p.R59L	8	133	6.0%	.	.	.
GC13	1	204380404	C	A	PPP1R15B	Missense	p.G46W	7	107	6.5%	.	.	.
GC13	1	204403680	C	A	PIK3C2B	Missense	p.L1191F	10	130	7.7%	.	.	.
GC13	1	204415174	C	A	PIK3C2B	Missense	p.W863L	8	75	10.7%	.	.	.
GC13	1	204429777	C	A	PIK3C2B	Missense	p.L441F	6	71	8.5%	.	.	.
GC13	1	204433668	G	T	PIK3C2B	Missense	p.P367T	7	79	8.9%	.	.	.
GC13	1	204438434	C	A	PIK3C2B	Missense	p.W166L	7	91	7.7%	.	.	.
GC13	1	204511978	G	T	MDM4	Missense	p.W95L	7	84	8.3%	.	.	.
GC13	1	204513706	G	T	MDM4	Missense	p.W141L	8	119	6.7%	.	.	.
GC13	1	204518567	G	T	MDM4	Missense	p.M84I	11	211	5.2%	.	.	.
GC13	1	205085003	C	A	RBBP5	Missense	p.G10W	9	132	6.8%	.	.	.
GC13	1	205350818	G	T	LEMD1	Missense	p.L131M	8	110	7.3%	.	.	.
GC13	1	205350874	G	T	LEMD1	Missense	p.Q65K	10	128	7.8%	.	.	.
GC13	1	205628660	G	T	SLC45A3	Missense	p.P455Q	8	70	11.4%	.	.	.
GC13	1	205768949	C	A	SLC41A1	Nonsense	p.G164X	6	47	12.8%	.	O	.
GC13	1	205902109	C	A	SLC26A9	Missense	p.G77C	6	74	8.1%	.	.	.
GC13	1	206331029	G	T	CTSE	Missense	p.W223L	5	35	14.3%	.	.	.
GC13	1	206331104	G	T	CTSE	Missense	p.W248L	6	69	8.7%	.	.	.
GC13	1	206822155	G	T	DYRK3	Missense	p.G538W	8	96	8.3%	.	.	.
GC13	1	206822190	G	T	DYRK3	Missense	p.Q549H	8	103	7.8%	.	.	.
GC13	1	207314480	G	T	C4BPA	Missense	p.G435W	9	119	7.6%	.	.	.
GC13	1	207532923	G	T	CD55	Missense	p.G372W	9	104	8.7%	.	.	.

GC13	1	207646958	G	T	CR2	Missense	p.G683W	5	43	11.6%	.	.	.
GC13	1	207782952	G	T	CR1	Missense	p.G1622W	5	62	8.1%	.	.	.
GC13	1	208215699	C	A	PLXNA2	Missense	p.G1344W	5	47	10.6%	.	.	.
GC13	1	209956294	G	T	C1orf74	Missense	p.P229Q	8	105	7.6%	.	.	.
GC13	1	210001421	G	T	DIEXF	Missense	p.G5W	7	107	6.5%	.	.	.
GC13	1	210012276	G	T	DIEXF	Missense	p.R362L	8	122	6.6%	.	.	.
GC13	1	210412837	G	T	SERTAD4	Splicing	c.176-1G>T	8	107	7.5%	.	.	.
GC13	1	211093033	G	T	KCNH1	Missense	p.P444T	7	85	8.2%	.	.	.
GC13	1	211487015	C	A	RCOR3	Missense	p.R465S	6	96	6.3%	.	.	.
GC13	1	211545937	C	A	TRAF5	Missense	p.R523S	8	105	7.6%	.	.	.
GC13	1	211751753	G	T	SLC30A1	Missense	p.Q68K	9	108	8.3%	.	.	.
GC13	1	212190359	C	A	INTS7	Missense	p.L77F	7	90	7.8%	.	.	.
GC13	1	212273843	G	T	DTL	Missense	p.W233L	12	104	11.5%	.	.	.
GC13	1	212274379	C	A	DTL	Missense	p.Q412K	7	72	9.7%	.	.	.
GC13	1	212798698	G	T	FAM71A	Missense	p.R160L	7	112	6.3%	.	.	.
GC13	1	212799459	C	A	FAM71A	Missense	p.Q414K	8	73	11.0%	.	.	.
GC13	1	213445958	G	T	RPS6KC1	Missense	p.W596L	6	60	10.0%	.	.	.
GC13	1	214209032	G	T	PROX1	Missense	p.R690L	8	102	7.8%	.	.	.
GC13	1	214794166	G	T	CENPF	Missense	p.G248W	7	82	8.5%	.	.	.
GC13	1	214819395	G	T	CENPF	Missense	p.R2161M	8	114	7.0%	.	.	.
GC13	1	215751056	G	T	KCTD3	Missense	p.G101W	6	81	7.4%	.	.	.
GC13	1	215987179	G	T	USH2A	Missense	p.P3213Q	6	100	6.0%	.	O	.
GC13	1	216061964	G	T	USH2A	Missense	p.P2676Q	6	86	7.0%	.	O	.
GC13	1	217793383	G	T	GPATCH2	Missense	p.P172Q	9	114	7.9%	.	.	.
GC13	1	217947722	G	T	SPATA17	Missense	p.W189L	7	78	9.0%	.	.	.
GC13	1	220142265	C	A	EPRS	Missense	p.M1474I	6	68	8.8%	.	.	.
GC13	1	220195710	G	T	EPRS	Missense	p.P365Q	7	76	9.2%	.	.	.
GC13	1	220298632	G	T	IARS2	Missense	p.W565L	7	86	8.1%	.	.	.
GC13	1	220326613	G	T	RAB3GAP2	Missense	p.H1261N	9	93	9.7%	.	.	.
GC13	1	220406190	C	A	RAB3GAP2	Missense	p.W44L	12	142	8.5%	.	O	.
GC13	1	220789326	C	A	MARK1	Missense	p.R159S	5	69	7.2%	.	.	.

GC13	1	220826612	C	A	MARK1	Missense	p.H614N	7	67	10.4%	.	.	.
GC13	1	221053212	G	T	HLX	Missense	p.G5W	5	38	13.2%	.	.	.
GC13	1	221876002	C	A	DUSP10	Missense	p.G401W	7	46	15.2%	.	.	.
GC13	1	221879739	C	A	DUSP10	Missense	p.R294L	4	25	16.0%	.	O	.
GC13	1	222717003	G	T	HHIPL2	Missense	p.R284S	7	90	7.8%	.	.	.
GC13	1	222750961	G	T	TAF1A	Missense	p.L30I	7	95	7.4%	.	.	.
GC13	1	222794515	G	T	MIA3	Missense	p.G50C	6	91	6.6%	.	O	.
GC13	1	222802144	C	A	MIA3	Missense	p.L528I	7	84	8.3%	.	.	.
GC13	1	222818982	G	T	MIA3	Missense	p.L66F	9	129	7.0%	.	.	.
GC13	1	222825627	C	A	MIA3	Missense	p.H225N	8	104	7.7%	.	.	.
GC13	1	222898805	C	A	BROX	Missense	p.P131Q	8	119	6.7%	.	.	.
GC13	1	223285242	G	T	TLR5	Missense	p.Q378K	7	93	7.5%	.	.	.
GC13	1	223285572	C	A	TLR5	Missense	p.G268W	8	93	8.6%	.	.	.
GC13	1	223949939	C	A	CAPN2	Missense	p.Q462K	7	74	9.5%	.	.	.
GC13	1	224377473	C	A	DEGS1	Missense	p.H93N	8	84	9.5%	.	.	.
GC13	1	224559012	G	T	CNIH4	Missense	p.M30I	5	30	16.7%	.	.	.
GC13	1	225328632	G	A	DNAH14	Missense	p.A1456T	52	86	60.5%	.	.	.
GC13	1	225418839	G	T	DNAH14	Missense	p.K2146N	8	92	8.7%	.	O	.
GC13	1	225428378	G	T	DNAH14	Missense	p.W2179L	7	91	7.7%	.	.	.
GC13	1	225528340	C	A	DNAH14	Missense	p.L3446I	8	119	6.7%	.	.	.
GC13	1	225541556	G	T	DNAH14	Missense	p.W3769L	9	141	6.4%	.	.	.
GC13	1	226342353	G	T	ACBD3	Missense	p.P362Q	8	125	6.4%	.	.	.
GC13	1	226829674	C	A	ITPKB	Missense	p.R800L	8	68	11.8%	.	.	.
GC13	1	227077792	C	A	PSEN2	Missense	p.Q282K	7	63	11.1%	.	.	.
GC13	1	227172280	G	T	COQ8A	Missense	p.R477M	7	84	8.3%	.	.	.
GC13	1	227174333	G	T	COQ8A	Missense	p.M613I	7	88	8.0%	.	.	.
GC13	1	227216679	G	T	CDC42BPA	Missense	p.R1255S	8	91	8.8%	.	.	.
GC13	1	227219133	C	A	CDC42BPA	Missense	p.W1098L	10	117	8.5%	.	.	.
GC13	1	227441769	C	A	CDC42BPA	Missense	p.G89V	8	146	5.5%	.	.	.
GC13	1	228112991	G	T	WNT9A	Missense	p.R109S	4	34	11.8%	.	.	.
GC13	1	228238363	G	T	WNT3A	Missense	p.R107M	6	47	12.8%	.	.	.

GC13	1	228463634	C	A	OBSCN	Missense	p.R2043S	6	49	12.2%	.	.	.
GC13	1	228470847	C	A	OBSCN	Missense	p.R2867S	6	76	7.9%	.	.	.
GC13	1	229568543	G	T	ACTA1	Missense	p.P72T	5	40	12.5%	.	.	.
GC13	1	229634054	C	A	NUP133	Splicing	c.649-1G>T	7	84	8.3%	.	.	.
GC13	1	229738250	C	A	TAF5L	Missense	p.G222C	6	91	6.6%	.	.	.
GC13	1	229770862	C	A	URB2	Missense	p.Q168K	7	71	9.9%	.	.	.
GC13	1	229772644	C	A	URB2	Missense	p.L762M	7	68	10.3%	.	.	.
GC13	1	229773781	C	A	URB2	Missense	p.Q1141K	8	84	9.5%	.	.	.
GC13	1	230381819	G	T	GALNT2	Missense	p.R209L	7	75	9.3%	.	.	.
GC13	1	231472795	G	T	EXOC8	Missense	p.L233I	10	99	10.1%	.	.	.
GC13	1	231830165	G	T	DISC1	Missense	p.G221W	7	109	6.4%	.	.	.
GC13	1	232650880	G	T	SIPA1L2	Missense	p.P69Q	12	115	10.4%	.	.	.
GC13	1	233150457	G	T	PCNX2	Missense	p.L1636M	7	71	9.9%	.	.	.
GC13	1	233394002	C	A	PCNX2	Missense	p.G536W	8	110	7.3%	.	.	.
GC13	1	234536949	G	T	TARBP1	Missense	p.P1350Q	9	98	9.2%	.	.	.
GC13	1	235345367	G	T	ARID4B	Missense	p.P870Q	7	133	5.3%	.	.	.
GC13	1	235345371	G	T	ARID4B	Missense	p.P869T	12	133	9.0%	.	.	.
GC13	1	235596291	G	T	TBCE	Missense	p.G231C	7	102	6.9%	.	.	.
GC13	1	235613543	G	T	B3GALNT2	Missense	p.P494H	15	280	5.4%	.	.	.
GC13	1	235617571	G	T	B3GALNT2	Missense	p.P403Q	6	97	6.2%	.	.	.
GC13	1	235747096	G	T	GNG4	Missense	p.Q15K	10	105	9.5%	.	.	.
GC13	1	235875398	G	T	LYST	Missense	p.P3295Q	11	191	5.8%	.	.	.
GC13	1	235884021	G	T	LYST	Missense	p.P3167Q	11	162	6.8%	.	.	.
GC13	1	235892928	G	T	LYST	Missense	p.P3025Q	11	148	7.4%	.	.	.
GC13	1	235896912	G	T	LYST	Missense	p.Q2898K	12	195	6.2%	.	.	.
GC13	1	235896930	G	T	LYST	Missense	p.Q2892K	11	196	5.6%	.	.	.
GC13	1	235922581	G	T	LYST	Missense	p.P2191Q	9	196	4.6%	.	.	.
GC13	1	235929499	C	A	LYST	Nonsense	p.G2001X	8	131	6.1%	.	.	.
GC13	1	235945287	G	T	LYST	Missense	p.Q1655K	12	196	6.1%	.	O	.
GC13	1	235955113	G	T	LYST	Missense	p.L1477M	14	193	7.3%	.	.	.
GC13	1	235955296	C	A	LYST	Missense	p.G1416W	10	195	5.1%	.	.	.

GC13	1	235964239	G	T	LYST	Missense	p.H1291N	10	170	5.9%	.	.	.
GC13	1	235966217	G	T	LYST	Missense	p.Q1235K	11	201	5.5%	.	.	.
GC13	1	235969561	G	T	LYST	Missense	p.Q959K	11	211	5.2%	.	.	.
GC13	1	235972487	G	T	LYST	Missense	p.P544H	9	195	4.6%	.	.	.
GC13	1	236141229	C	A	NID1	Missense	p.G1228W	13	161	8.1%	.	.	.
GC13	1	236145070	G	T	NID1	Missense	p.P1023Q	9	179	5.0%	.	.	.
GC13	1	236148709	C	A	NID1	Missense	p.G1009C	8	119	6.7%	.	.	.
GC13	1	236157038	G	T	NID1	Missense	p.Q888K	8	73	11.0%	.	.	.
GC13	1	236187466	C	A	NID1	Missense	p.G678W	9	147	6.1%	.	.	.
GC13	1	236212087	G	T	NID1	Missense	p.P143Q	8	96	8.3%	.	.	.
GC13	1	236212232	C	A	NID1	Missense	p.G95W	11	127	8.7%	.	.	.
GC13	1	236368549	G	T	GPR137B	Missense	p.G364C	8	84	9.5%	.	.	.
GC13	1	236385277	G	T	ERO1B	Missense	p.R386S	10	219	4.6%	.	.	.
GC13	1	236557772	G	T	EDARADD	Missense	p.G10W	8	105	7.6%	.	.	.
GC13	1	236758924	C	A	HEATR1	Missense	p.G336W	10	196	5.1%	.	.	.
GC13	1	236758965	G	T	HEATR1	Missense	p.P322H	10	195	5.1%	.	.	.
GC13	1	236761240	G	T	HEATR1	Missense	p.H181N	10	176	5.7%	.	.	.
GC13	1	236987440	G	T	MTR	Missense	p.L262F	8	143	5.6%	.	.	.
GC13	1	236990149	G	T	MTR	Missense	p.L316F	9	176	5.1%	.	.	.
GC13	1	236992488	G	T	MTR	Splicing	c.996-1G>T;NM_001291940	11	159	6.9%	.	.	.
GC13	1	237433859	G	T	RYR2	Missense	p.L37F	9	134	6.7%	.	.	.
GC13	1	237604660	G	T	RYR2	Missense	p.M349I	11	129	8.5%	.	.	.
GC13	1	237666608	G	T	RYR2	Missense	p.G806W	7	83	8.4%	.	.	.
GC13	1	237713991	G	T	RYR2	Missense	p.A1072S	11	176	6.3%	.	.	.
GC13	1	237753102	C	A	RYR2	Missense	p.P1203H	11	162	6.8%	.	.	.
GC13	1	237755119	G	T	RYR2	Missense	p.R1414L	15	233	6.4%	.	.	.
GC13	1	237765399	G	T	RYR2	Missense	p.L1557F	7	82	8.5%	.	.	.
GC13	1	237791307	C	A	RYR2	Missense	p.L2123I	8	144	5.6%	.	.	.
GC13	1	237794726	G	T	RYR2	Splicing	c.6441-1G>T	14	211	6.6%	.	.	.
GC13	1	237850804	G	T	RYR2	Missense	p.G3023C	16	271	5.9%	.	.	.
GC13	1	237870447	G	T	RYR2	Missense	p.R3260L	9	170	5.3%	.	.	.

GC13	1	237947487	C	A	RYR2	Missense	p.Q4159K	10	161	6.2%	.	.	.
GC13	1	237947955	G	T	RYR2	Missense	p.G4315W	10	189	5.3%	.	.	.
GC13	1	237969529	G	T	RYR2	Missense	p.M4748I	12	230	5.2%	.	.	.
GC13	1	240071103	G	T	CHRM3	Missense	p.G118W	9	167	5.4%	.	.	.
GC13	1	240255689	C	A	FMN2	Missense	p.R94S	7	98	7.1%	.	O	.
GC13	1	240351536	C	A	FMN2	Missense	p.Q654K	12	177	6.8%	.	.	.
GC13	1	240370573	G	T	FMN2	Missense	p.G821W	11	164	6.7%	.	.	.
GC13	1	240975278	G	T	RGS7	Missense	p.P288Q	12	217	5.5%	.	.	.
GC13	1	241099990	C	A	RGS7	Missense	p.L81F	7	83	8.4%	.	O	.
GC13	1	241676959	G	T	FH	Missense	p.Q108K	12	202	5.9%	O	.	.
GC13	1	241753361	G	T	KMO	Missense	p.M382I	9	190	4.7%	.	.	.
GC13	1	241797120	G	T	CHML	Missense	p.P650Q	10	178	5.6%	.	.	.
GC13	1	241803403	C	A	OPN3	Missense	p.G52W	8	68	11.8%	.	.	.
GC13	1	241850789	G	T	WDR64	Missense	p.W279L	10	180	5.6%	.	.	.
GC13	1	242024002	G	T	EXO1	Missense	p.G314W	10	171	5.8%	.	.	.
GC13	1	242052808	C	A	EXO1	Missense	p.P816Q	8	92	8.7%	.	.	.
GC13	1	242162254	C	A	MAP1LC3C	Missense	p.L19F	14	211	6.6%	.	.	.
GC13	1	242162288	G	T	MAP1LC3C	Missense	p.P8Q	9	170	5.3%	.	.	.
GC13	1	243456504	G	T	SDCCAG8	Missense	p.G220C	10	184	5.4%	.	.	.
GC13	1	243468063	C	A	SDCCAG8	Missense	p.Q144K	17	180	9.4%	.	.	.
GC13	1	243471433	C	A	SDCCAG8	Missense	p.Q197K	10	128	7.8%	.	.	.
GC13	1	243809209	G	T	AKT3	Missense	p.H139N	7	93	7.5%	.	.	.
GC13	1	243809262	G	T	AKT3	Missense	p.P121Q	9	158	5.7%	.	.	.
GC13	1	244217203	C	A	ZBTB18	Missense	p.Q34K	11	182	6.0%	.	.	.
GC13	1	244724118	G	T	CATSPERE	Missense	p.R242M	11	207	5.3%	.	.	.
GC13	1	244868867	G	T	DESI2	Missense	p.G88W	9	117	7.7%	.	.	.
GC13	1	245019445	G	T	HNRNPU	Missense	p.P624Q	11	152	7.2%	.	.	.
GC13	1	245019848	G	T	HNRNPU	Missense	p.P589Q	10	162	6.2%	.	.	.
GC13	1	245021470	G	T	HNRNPU	Missense	p.P427Q	10	203	4.9%	.	.	.
GC13	1	245022577	C	A	HNRNPU	Missense	p.G354C	10	162	6.2%	.	.	.
GC13	1	245023723	G	T	HNRNPU	Missense	p.L292I	13	184	7.1%	.	.	.

GC13	1	245530209	G	T	KIF26B	Missense	p.R180L	8	110	7.3%	.	.	.
GC13	1	245847561	G	T	KIF26B	Missense	p.R762L	8	176	4.5%	.	O	.
GC13	1	245847623	G	T	KIF26B	Missense	p.G783W	9	187	4.8%	.	.	.
GC13	1	245851614	G	T	KIF26B	Missense	p.G1777W	8	65	12.3%	.	.	.
GC13	1	246498751	C	A	SMYD3	Missense	p.R85L	12	175	6.9%	.	.	.
GC13	1	246719993	G	T	TFB2M	Missense	p.P196Q	11	190	5.8%	.	.	.
GC13	1	246797310	G	T	CNST	Missense	p.W234L	13	217	6.0%	.	.	.
GC13	1	246810897	G	T	CNST	Missense	p.R465L	11	208	5.3%	.	.	.
GC13	1	246922356	G	T	SCCPDH	Missense	p.R239L	9	195	4.6%	.	.	.
GC13	1	246927624	C	A	SCCPDH	Missense	p.P356Q	8	142	5.6%	.	.	.
GC13	1	247021041	G	T	AHCTF1	Missense	p.P1403Q	6	86	7.0%	.	.	.
GC13	1	247050538	C	A	AHCTF1	Missense	p.W816L	7	67	10.4%	.	.	.
GC13	1	247050568	C	A	AHCTF1	Missense	p.W806L	8	98	8.2%	.	.	.
GC13	1	247265046	C	A	ZNF669	Missense	p.G60W	12	166	7.2%	.	.	.
GC13	1	247320122	C	A	ZNF124	Missense	p.G268W	11	152	7.2%	.	.	.
GC13	1	247320437	G	T	ZNF124	Missense	p.R163S	9	114	7.9%	.	.	.
GC13	1	247587493	C	A	NLRP3	Missense	p.Q250K	10	136	7.4%	.	.	.
GC13	1	247592890	G	T	NLRP3	Missense	p.L718F	11	149	7.4%	.	.	.
GC13	1	247615248	G	T	OR2B11	Missense	p.P13T	10	150	6.7%	.	.	.
GC13	1	247654619	G	T	OR2W5	Missense	p.G64W	11	182	6.0%	.	O	.
GC13	1	247655127	G	T	OR2W5	Missense	p.R233M	7	85	8.2%	.	.	.
GC13	1	247655330	C	A	OR2W5	Missense	p.H301N	11	135	8.1%	.	O	.
GC13	1	247695009	G	T	OR2C3	Missense	p.H269N	9	144	6.3%	.	O	.
GC13	1	247695054	C	A	OR2C3	Missense	p.G254W	9	163	5.5%	.	.	.
GC13	1	247751790	G	T	OR2G2	Missense	p.L43F	10	170	5.9%	.	.	.
GC13	1	247769170	G	T	OR2G3	Missense	p.G95C	9	177	5.1%	.	.	.
GC13	1	247769698	G	T	OR2G3	Missense	p.G271W	8	134	6.0%	.	.	.
GC13	1	248005078	C	A	OR11L1	Missense	p.G41W	8	113	7.1%	.	.	.
GC13	1	248129487	G	T	OR2AK2	Missense	p.R285L	11	160	6.9%	.	O	.
GC13	1	248262719	G	T	OR2L13	Missense	p.L14F	10	178	5.6%	.	O	.
GC13	1	248402516	G	T	OR2M4	Missense	p.G96C	10	166	6.0%	.	.	.

GC13	1	248402616	C	A	OR2M4	Missense	p.P129H	10	165	6.1%	.	.	.
GC13	1	248402922	G	T	OR2M4	Missense	p.G231V	8	117	6.8%	.	.	.
GC13	1	248525493	G	T	OR2T4	Missense	p.R204L	8	147	5.4%	.	.	.
GC13	1	248550994	G	T	OR2T6	Missense	p.G29C	11	159	6.9%	.	.	.
GC13	1	248569411	G	T	OR2T1	Missense	p.W39L	10	188	5.3%	.	.	.
GC13	1	248616447	G	T	OR2T2	Missense	p.G117C	9	138	6.5%	.	.	.
GC13	1	248636661	G	T	OR2T3	Missense	p.G4W	12	279	4.3%	.	.	.
GC13	1	248814115	G	T	OR2T27	Missense	p.P24H	8	125	6.4%	.	.	.
GC13	1	248845118	C	A	OR14I1	Missense	p.R163L	9	171	5.3%	.	.	.
GC13	1	249148190	C	A	ZNF692	Missense	p.G322W	9	123	7.3%	.	.	.
GC13	1	249151606	C	A	ZNF692	Missense	p.R106L	6	97	6.2%	.	.	.
GC13	1	249211535	G	T	PGBD2	Missense	p.R251L	10	213	4.7%	.	.	.
GC13	1	249212521	G	T	PGBD2	Missense	p.G329C	10	164	6.1%	.	.	.
GC13	2	1320064	C	A	SNTG2	Missense	p.Q469K	7	98	7.1%	.	.	.
GC13	2	1457465	G	T	TPO	Splicing	c.483-1G>T;NM_000547	14	76	18.4%	.	O	.
GC13	2	1499939	C	A	TPO	Missense	p.L556M	7	63	11.1%	.	.	.
GC13	2	3392346	G	T	TRAPPC12	Nonsense	p.G318X	6	51	11.8%	.	.	.
GC13	2	3687919	C	A	COLEC11	Missense	p.P35Q	6	43	14.0%	.	.	.
GC13	2	3744978	G	T	ALLC	Missense	p.W261L	8	83	9.6%	.	.	.
GC13	2	8871289	C	A	KIDINS220	Missense	p.R1570L	6	95	6.3%	.	.	.
GC13	2	9554367	C	A	ITGB1BP1	Missense	p.G31W	6	58	10.3%	.	.	.
GC13	2	9581996	C	A	CPSF3	Missense	p.P292Q	8	97	8.2%	.	.	.
GC13	2	10053360	G	T	TAF1B	Missense	p.W163L	10	127	7.9%	.	.	.
GC13	2	10187915	G	T	KLF11	Missense	p.G134W	6	77	7.8%	.	.	.
GC13	2	10267076	G	T	RRM2	Missense	p.G215V	5	34	14.7%	.	.	.
GC13	2	10743250	C	A	NOL10	Missense	p.R370L	7	88	8.0%	.	O	.
GC13	2	11053091	C	A	KCNF1	Missense	p.P180Q	6	56	10.7%	.	.	.
GC13	2	11389836	C	A	ROCK2	Missense	p.W52L	8	132	6.1%	.	.	.
GC13	2	11728968	G	T	GREB1	Missense	p.R419L	6	80	7.5%	.	.	.
GC13	2	11922459	G	T	LPIN1	Missense	p.G328W	9	110	8.2%	.	.	.
GC13	2	15417014	G	T	NBAS	Missense	p.H1784N	8	91	8.8%	.	.	.

GC13	2	15615837	G	T	NBAS	Missense	p.H439N	6	63	9.5%	.	.	.
GC13	2	15739840	G	T	DDX1	Missense	p.A103S	8	109	7.3%	.	.	.
GC13	2	15763586	C	A	DDX1	Missense	p.L492M	8	101	7.9%	.	O	.
GC13	2	17698134	G	T	RAD51AP2	Missense	p.H517N	7	70	10.0%	.	.	.
GC13	2	17888613	G	T	SMC6	Missense	p.Q627K	7	90	7.8%	.	.	.
GC13	2	17913080	C	A	SMC6	Missense	p.G137C	8	118	6.8%	.	.	.
GC13	2	17962910	G	T	GEN1	Missense	p.G811W	10	112	8.9%	.	.	.
GC13	2	20113336	G	T	WDR35	Missense	p.H1166N	8	129	6.2%	.	.	.
GC13	2	21230282	C	A	APOB	Missense	p.W3153L	10	110	9.1%	.	.	.
GC13	2	21231408	C	A	APOB	Missense	p.G2778W	9	93	9.7%	.	.	.
GC13	2	21233348	C	A	APOB	Missense	p.W2131L	8	96	8.3%	.	.	.
GC13	2	21238259	C	A	APOB	Missense	p.R1164M	8	91	8.8%	.	.	.
GC13	2	21250700	C	A	APOB	Missense	p.E689D	7	85	8.2%	.	.	.
GC13	2	21251283	G	T	APOB	Missense	p.P582Q	7	99	7.1%	.	.	.
GC13	2	21362063	C	A	TDRD15	Missense	p.P575Q	7	99	7.1%	.	.	.
GC13	2	23977087	G	T	ATAD2B	Missense	p.R1428S	6	83	7.2%	.	O	.
GC13	2	24110838	G	T	ATAD2B	Missense	p.L146I	7	95	7.4%	.	.	.
GC13	2	24261355	G	T	WDCP	Missense	p.P337Q	8	100	8.0%	.	.	.
GC13	2	24345367	C	A	PFN4	Missense	p.L13F	7	82	8.5%	.	O	.
GC13	2	24471745	C	A	ITSN2	Missense	p.G1003W	6	63	9.5%	.	.	.
GC13	2	24531566	C	A	ITSN2	Missense	p.W238L	6	64	9.4%	.	.	.
GC13	2	24964832	G	T	NCOA1	Missense	p.Q1161H	7	91	7.7%	.	.	.
GC13	2	25062824	G	T	ADCY3	Missense	p.L425M	6	60	10.0%	.	.	.
GC13	2	25352918	G	T	EFR3B	Missense	p.R202L	6	48	12.5%	.	.	.
GC13	2	25523052	C	A	DNMT3A	Missense	p.A45S	10	24	41.7%	O	.	.
GC13	2	26204081	G	T	KIF3C	Missense	p.H236N	6	60	10.0%	.	.	.
GC13	2	26410172	G	T	GAREM2	Missense	p.W480C	7	63	11.1%	.	.	.
GC13	2	26507751	G	T	HADHB	Missense	p.G369C	7	81	8.6%	.	.	.
GC13	2	26681020	C	A	OTOF	Missense	p.R1194L	7	88	8.0%	.	.	.
GC13	2	27121620	G	T	DPYSL5	Missense	p.G85W	7	96	7.3%	.	.	.
GC13	2	27278623	G	T	AGBL5	Missense	p.G328W	5	48	10.4%	.	.	.

GC13	2	27278872	G	T	AGBL5	Missense	p.G411W	7	97	7.2%	.	.	.
GC13	2	27292484	G	T	AGBL5	Missense	p.G800V	7	82	8.5%	.	.	.
GC13	2	27324221	G	T	CGREF1	Missense	p.P293Q	8	81	9.9%	.	.	.
GC13	2	27324939	G	T	CGREF1	Missense	p.P77Q	6	62	9.7%	.	.	.
GC13	2	27447688	C	A	CAD	Missense	p.Q444K	7	99	7.1%	.	.	.
GC13	2	27455912	G	T	CAD	Missense	p.M902I	6	61	9.8%	.	.	.
GC13	2	27460945	C	A	CAD	Missense	p.H1521N	7	76	9.2%	.	.	.
GC13	2	27592406	C	A	EIF2B4	Missense	p.R50M	8	125	6.4%	.	.	.
GC13	2	27658034	G	T	NRBP1	Missense	p.G169W	7	53	13.2%	.	.	.
GC13	2	27663313	G	T	NRBP1	Missense	p.M328I	7	95	7.4%	.	.	.
GC13	2	27668677	G	T	IFT172	Missense	p.H1617N	5	43	11.6%	.	.	.
GC13	2	27679490	G	T	IFT172	Missense	p.H1087N	7	99	7.1%	.	.	.
GC13	2	27700142	C	A	IFT172	Missense	p.G423W	10	78	12.8%	.	.	.
GC13	2	27720474	G	T	GCKR	Missense	p.G88W	6	90	6.7%	.	.	.
GC13	2	27799552	C	A	C2orf16	Missense	p.P38Q	9	110	8.2%	.	.	.
GC13	2	27802257	C	A	C2orf16	Missense	p.Q940K	7	98	7.1%	.	.	.
GC13	2	27803571	G	T	C2orf16	Missense	p.G1378W	8	111	7.2%	.	.	.
GC13	2	27804076	G	T	C2orf16	Missense	p.R1546M	10	105	9.5%	.	.	.
GC13	2	27911567	C	T	SLC4A1AP	Missense	p.A735V	8	49	16.3%	.	.	.
GC13	2	28268595	G	T	BABAM2	Missense	p.G167C	6	70	8.6%	.	.	.
GC13	2	28626981	G	T	FOSL2	Missense	p.R37L	8	77	10.4%	.	.	.
GC13	2	28821528	G	T	PLB1	Missense	p.R781L	8	95	8.4%	.	.	.
GC13	2	29039115	G	T	SPDYA	Missense	p.D79Y	6	47	12.8%	.	.	.
GC13	2	29087948	G	T	TRMT61B	Missense	p.P246Q	8	92	8.7%	.	.	.
GC13	2	29092449	G	T	TRMT61B	Missense	p.P232Q	8	128	6.3%	.	.	.
GC13	2	29117686	G	T	WDR43	Missense	p.W41L	7	66	10.6%	.	.	.
GC13	2	29124862	C	A	WDR43	Missense	p.Q79K	8	113	7.1%	.	.	.
GC13	2	29245126	C	A	TOGARAM 2	Missense	p.P433Q	5	42	11.9%	.	.	.
GC13	2	29344306	G	T	CLIP4	Nonsense	p.G18X	7	87	8.0%	.	.	.
GC13	2	29368225	C	A	CLIP4	Missense	p.P338H	8	79	10.1%	.	.	.
GC13	2	30975988	C	A	CAPN13	Nonsense	p.G340X	7	64	10.9%	.	.	.

GC13	2	31403861	C	A	CAPN14	Missense	p.G551W	6	52	11.5%	.	.	.
GC13	2	31483447	C	A	EHD3	Missense	p.H192N	8	107	7.5%	.	.	.
GC13	2	31560551	G	T	XDH	Missense	p.P1303T	7	58	12.1%	.	.	.
GC13	2	32370016	G	T	SPAST	Nonsense	p.G511X	7	74	9.5%	.	.	.
GC13	2	32409406	G	T	SLC30A6	Missense	p.G21W	11	115	9.6%	.	.	.
GC13	2	32445491	G	T	SLC30A6	Missense	p.K168N	9	86	10.5%	.	.	.
GC13	2	32664684	C	A	BIRC6	Missense	p.P1247Q	7	80	8.8%	.	.	.
GC13	2	32664689	C	A	BIRC6	Missense	p.L1249I	7	81	8.6%	.	.	.
GC13	2	32667398	G	T	BIRC6	Missense	p.K1371N	8	120	6.7%	.	O	.
GC13	2	32668585	C	A	BIRC6	Missense	p.P1422Q	8	129	6.2%	.	.	.
GC13	2	32689674	C	A	BIRC6	Missense	p.P1680Q	6	80	7.5%	.	.	.
GC13	2	32695261	G	T	BIRC6	Missense	p.G2125C	7	100	7.0%	.	.	.
GC13	2	32713670	C	A	BIRC6	Missense	p.Q2662K	6	61	9.8%	.	.	.
GC13	2	32819062	C	A	BIRC6	Missense	p.P4479Q	9	94	9.6%	.	.	.
GC13	2	32836609	G	T	BIRC6	Missense	p.R4785L	5	59	8.5%	.	.	.
GC13	2	33810040	G	T	FAM98A	Missense	p.H259N	7	79	8.9%	.	.	.
GC13	2	37259898	G	T	HEATR5B	Missense	p.H1079N	10	144	6.9%	.	.	.
GC13	2	37292958	C	A	HEATR5B	Missense	p.G425W	5	58	8.6%	.	.	.
GC13	2	37443562	C	A	CEBPZ	Splicing	c.2209-1G>T	7	61	11.5%	.	.	.
GC13	2	37596927	G	T	QPCT	Nonsense	p.E275X	7	96	7.3%	.	.	.
GC13	2	37873086	C	A	CDC42EP3	Missense	p.K215N	8	54	14.8%	.	.	.
GC13	2	38178956	G	T	RMDN2	Missense	p.G200W	13	131	9.9%	.	.	.
GC13	2	38179072	G	T	RMDN2	Missense	p.R238S	9	107	8.4%	.	.	.
GC13	2	38812796	G	T	HNRNPLL	Missense	p.P179Q	6	74	8.1%	.	.	.
GC13	2	39033777	G	T	DHX57	Missense	p.P1145Q	8	121	6.6%	.	.	.
GC13	2	39148564	G	T	ARHGEF33	Missense	p.Q25H	8	89	9.0%	.	.	.
GC13	2	39414838	G	T	CDKL4	Missense	p.P222Q	10	96	10.4%	.	.	.
GC13	2	39440603	C	A	CDKL4	Nonsense	p.G101X	8	100	8.0%	.	.	.
GC13	2	42284773	G	T	PKDCC	Missense	p.W476L	6	67	9.0%	.	.	.
GC13	2	42543154	G	T	EML4	Missense	p.G616W	9	92	9.8%	.	.	.
GC13	2	42924968	C	A	MTA3	Missense	p.P277Q	8	98	8.2%	.	.	.

GC13	2	43921631	G	T	PLEKHH2	Missense	p.W133L	8	134	6.0%	.	.	.
GC13	2	43986080	G	T	PLEKHH2	Missense	p.R1328L	7	84	8.3%	.	.	.
GC13	2	44145245	G	T	LRPPRC	Missense	p.L1023M	7	84	8.3%	.	.	.
GC13	2	44203333	G	T	LRPPRC	Missense	p.P229Q	7	90	7.8%	.	.	.
GC13	2	44428361	C	A	PPM1B	Missense	p.P8H	9	94	9.6%	.	.	.
GC13	2	44457754	C	A	PPM1B	Missense	p.P159Q	8	117	6.8%	.	.	.
GC13	2	44502778	C	A	SLC3A1	Missense	p.P35Q	7	86	8.1%	.	.	.
GC13	2	45829088	G	T	SRBD1	Missense	p.P72Q	7	81	8.6%	.	.	.
GC13	2	46607774	G	T	EPAS1	Missense	p.G655W	5	64	7.8%	O	.	.
GC13	2	46607797	G	T	EPAS1	Missense	p.L662F	10	64	15.6%	O	.	.
GC13	2	46739373	G	T	ATP6V1E2	Missense	p.Q160K	7	93	7.5%	.	.	.
GC13	2	46739706	G	T	ATP6V1E2	Missense	p.Q49K	8	100	8.0%	.	.	.
GC13	2	47357356	G	T	STPG4	Missense	p.P148Q	7	81	8.6%	.	.	.
GC13	2	48026782	C	A	MSH6	Missense	p.R424S	7	101	6.9%	O	.	.
GC13	2	48027005	G	T	MSH6	Missense	p.W498L	7	95	7.4%	O	.	.
GC13	2	48033497	G	T	MSH6	Missense	p.M1137I	9	139	6.5%	O	.	.
GC13	2	48807915	G	T	STON1	Missense	p.R48M	8	107	7.5%	.	.	.
GC13	2	49190825	C	A	FSHR	Missense	p.G353W	8	72	11.1%	.	.	.
GC13	2	50149216	G	T	NRXN1	Missense	p.H99N	7	92	7.6%	.	.	.
GC13	2	54001289	G	T	CHAC2	Missense	p.W61L	8	93	8.6%	.	.	.
GC13	2	54850707	G	T	SPTBN1	Missense	p.G373W	7	83	8.4%	.	.	.
GC13	2	54877004	G	T	SPTBN1	Missense	p.G1806W	8	101	7.9%	.	.	.
GC13	2	55449510	G	T	CLHC1	Missense	p.P13Q	13	137	9.5%	.	.	.
GC13	2	55471206	G	T	MTIF2	Missense	p.L424I	7	49	14.3%	.	.	.
GC13	2	55530217	G	T	CCDC88A	Missense	p.R1486S	7	113	6.2%	.	.	.
GC13	2	55561865	G	T	CCDC88A	Missense	p.Q698K	12	132	9.1%	.	.	.
GC13	2	55562234	G	T	CCDC88A	Missense	p.Q575K	9	161	5.6%	.	.	.
GC13	2	55777075	G	T	PPP4R3B	Missense	p.P756H	8	124	6.5%	.	.	.
GC13	2	55874588	C	A	PNPT1	Missense	p.G499V	8	109	7.3%	.	.	.
GC13	2	55906885	G	T	PNPT1	Missense	p.P204Q	7	96	7.3%	.	.	.
GC13	2	55910954	G	T	PNPT1	Missense	p.P140Q	7	80	8.8%	.	.	.

GC13	2	55920882	C	A	PNPT1	Missense	p.R26L	8	102	7.8%	.	.	.
GC13	2	56103821	C	A	EFEMP1	Missense	p.G273C	10	118	8.5%	.	.	.
GC13	2	58362295	C	A	VRK2	Missense	p.Q155K	7	77	9.1%	.	.	.
GC13	2	58459232	G	T	FANCL	Missense	p.L38I	7	79	8.9%	.	.	.
GC13	2	60687964	C	A	BCL11A	Missense	p.G695W	7	90	7.8%	.	.	.
GC13	2	61145562	G	T	REL	Missense	p.W225L	8	105	7.6%	.	.	.
GC13	2	61389744	G	T	C2orf74	Missense	p.C39F	7	63	11.1%	.	.	.
GC13	2	61441635	C	A	USP34	Missense	p.G2748C	7	95	7.4%	.	.	.
GC13	2	61507391	C	A	USP34	Missense	p.K1748N	8	118	6.8%	.	.	.
GC13	2	61571112	G	T	USP34	Missense	p.Q780K	7	95	7.4%	.	.	.
GC13	2	61726876	G	T	XPO1	Missense	p.Q188K	7	72	9.7%	O	.	.
GC13	2	62449840	G	T	B3GNT2	Missense	p.R162L	6	88	6.8%	.	.	.
GC13	2	64189454	G	T	VPS54	Missense	p.Q238K	8	114	7.0%	.	.	.
GC13	2	64778652	C	A	AFTPH	Missense	p.P15Q	8	83	9.6%	.	.	.
GC13	2	65659102	G	T	SPRED2	Missense	p.P7Q	6	65	9.2%	.	.	.
GC13	2	67631648	C	A	ETAA1	Missense	p.Q612K	8	102	7.8%	.	.	.
GC13	2	67631997	C	A	ETAA1	Missense	p.P728Q	7	91	7.7%	.	.	.
GC13	2	68358480	G	T	WDR92	Missense	p.Q322K	7	85	8.2%	.	.	.
GC13	2	68384542	G	T	WDR92	Missense	p.H12N	9	88	10.2%	.	.	.
GC13	2	68385127	C	A	PNO1	Missense	p.R21S	7	110	6.4%	.	.	.
GC13	2	68691393	G	T	FBXO48	Missense	p.P139Q	10	138	7.2%	.	.	.
GC13	2	68873149	G	T	PROKR1	Missense	p.G66W	9	119	7.6%	.	.	.
GC13	2	69049605	C	A	ARHGAP25	Missense	p.P438H	8	93	8.6%	.	.	.
GC13	2	69049838	C	A	ARHGAP25	Missense	p.Q516K	9	71	12.7%	.	.	.
GC13	2	69732743	G	T	AAK1	Missense	p.Q743K	7	92	7.6%	.	.	.
GC13	2	70443340	C	A	TIA1	Missense	p.R179L	7	65	10.8%	.	.	.
GC13	2	71043612	G	T	CLEC4F	Missense	p.Q301K	7	84	8.3%	.	.	.
GC13	2	71429688	G	T	PAIP2B	Missense	p.P11Q	8	80	10.0%	.	.	.
GC13	2	71654475	C	A	ZNF638	Missense	p.Q1826K	8	120	6.7%	.	.	.
GC13	2	71742849	A	G	DYSF	Missense	p.I255V	28	54	51.9%	.	.	.
GC13	2	72740307	C	A	EXOC6B	Missense	p.W261L	7	90	7.8%	.	.	.

GC13	2	73479835	G	T	CCT7	Missense	p.W289L	5	48	10.4%	.	.	.
GC13	2	73519072	G	T	EGR4	Missense	p.P428H	5	34	14.7%	.	.	.
GC13	2	73677912	G	T	ALMS1	Missense	p.G1419W	8	94	8.5%	.	.	.
GC13	2	73829348	C	A	ALMS1	Missense	p.R4050S	7	104	6.7%	.	.	.
GC13	2	73957839	C	A	TPRKB	Missense	p.G97C	7	69	10.1%	.	.	.
GC13	2	74041250	G	T	C2orf78	Missense	p.M248I	6	68	8.8%	.	.	.
GC13	2	74688563	C	A	MOGS	Missense	p.G785C	8	80	10.0%	.	.	.
GC13	2	74690429	C	A	MOGS	Missense	p.G222W	8	105	7.6%	.	.	.
GC13	2	74719848	C	A	TTC31	Missense	p.R413S	6	88	6.8%	.	.	.
GC13	2	74729877	G	T	LBX2	Missense	p.P37Q	6	97	6.2%	.	.	.
GC13	2	74747153	C	A	DQX1	Missense	p.G502W	6	31	19.4%	.	.	.
GC13	2	74756541	C	A	AUP1	Missense	p.G46W	6	72	8.3%	.	.	.
GC13	2	74760063	G	T	HTRA2	Missense	p.R346L	7	100	7.0%	.	.	.
GC13	2	74783779	G	T	DOK1	Missense	p.E114D	8	94	8.5%	.	.	.
GC13	2	74783937	G	T	DOK1	Missense	p.R167L	6	58	10.3%	.	.	.
GC13	2	74901799	C	A	SEMA4F	Missense	p.Q178K	6	54	11.1%	.	.	.
GC13	2	74902365	G	T	SEMA4F	Missense	p.R254L	6	84	7.1%	.	.	.
GC13	2	74907146	G	T	SEMA4F	Missense	p.G553V	7	82	8.5%	.	O	.
GC13	2	75882230	G	T	MRPL19	Missense	p.W233L	7	79	8.9%	.	.	.
GC13	2	76975988	C	A	LRRTM4	Missense	p.G537W	6	59	10.2%	.	.	.
GC13	2	84668397	C	A	SUCLG1	Missense	p.G169W	8	111	7.2%	.	.	.
GC13	2	84774668	G	A	DNAH6	Missense	p.R373K	13	86	15.1%	.	.	.
GC13	2	85361493	G	T	TCF7L1	Missense	p.G121W	5	43	11.6%	.	.	.
GC13	2	85850772	G	T	USP39	Missense	p.R43L	8	115	7.0%	.	.	.
GC13	2	85922535	G	T	GNLY	Nonsense	p.E49X	8	62	12.9%	.	.	.
GC13	2	86733065	C	A	CHMP3	Missense	p.L111F	7	79	8.9%	.	.	.
GC13	2	88327892	G	T	KRCC1	Missense	p.P64Q	7	84	8.3%	.	.	.
GC13	2	88327950	C	A	KRCC1	Missense	p.G45W	7	85	8.2%	.	.	.
GC13	2	88390543	G	T	SMYD1	Missense	p.G181C	6	89	6.7%	.	.	.
GC13	2	88828648	G	T	TEX37	Missense	p.G67W	7	97	7.2%	.	.	.
GC13	2	95774082	G	T	MRPS5	Missense	p.Q80K	10	110	9.1%	.	.	.

GC13	2	95944793	C	A	PROM2	Missense	p.P392Q	4	25	16.0%	.	.	.
GC13	2	96781411	C	A	ADRA2B	Missense	p.G160W	5	46	10.9%	.	.	.
GC13	2	96933652	C	A	CIAO1	Missense	p.P160Q	11	99	11.1%	.	.	.
GC13	2	96953215	G	T	SNRNP200	Missense	p.P1149H	7	86	8.1%	.	.	.
GC13	2	96961357	C	A	SNRNP200	Missense	p.G571W	5	24	20.8%	.	.	.
GC13	2	97354992	G	T	FER1L5	Missense	p.G802W	6	47	12.8%	.	.	.
GC13	2	97475129	G	T	CNNM4	Missense	p.G735W	7	107	6.5%	.	.	.
GC13	2	97494732	G	T	CNNM3	Splicing	c.1777-1G>T	7	68	10.3%	.	.	.
GC13	2	97498333	G	T	CNNM3	Missense	p.G654W	7	88	8.0%	.	.	.
GC13	2	98418891	C	A	TMEM131	Missense	p.R884M	7	93	7.5%	.	.	.
GC13	2	99169319	C	A	INPP4A	Missense	p.Q412K	8	91	8.8%	.	.	.
GC13	2	99182138	G	T	INPP4A	Missense	p.G680W	6	35	17.1%	.	.	.
GC13	2	99256418	G	T	MGAT4A	Missense	p.P264Q	7	80	8.8%	.	.	.
GC13	2	99681512	G	T	TSGA10	Missense	p.R323S	6	88	6.8%	.	.	.
GC13	2	99725868	G	T	TSGA10	Missense	p.P12Q	7	86	8.1%	.	.	.
GC13	2	99779159	C	A	LIPT1	Missense	p.L247I	8	122	6.6%	.	.	.
GC13	2	99995805	G	T	EIF5B	Missense	p.G659C	6	93	6.5%	.	.	.
GC13	2	100006771	G	T	EIF5B	Missense	p.M831I	9	125	7.2%	.	.	.
GC13	2	100022452	C	A	REV1	Missense	p.G910W	8	69	11.6%	.	.	.
GC13	2	101624582	G	T	TBC1D8	Missense	p.Q1042K	8	73	11.0%	.	.	.
GC13	2	102805626	G	T	IL1RL2	Missense	p.G50V	9	110	8.2%	.	.	.
GC13	2	102979140	C	A	IL18R1	Missense	p.P7H	8	107	7.5%	.	.	.
GC13	2	102984324	G	T	IL18R1	Missense	p.G33V	7	93	7.5%	.	.	.
GC13	2	103324760	C	A	SLC9A2	Missense	p.H751N	7	74	9.5%	.	.	.
GC13	2	103324866	C	A	SLC9A2	Missense	p.P786Q	6	79	7.6%	.	.	.
GC13	2	105915096	C	A	TGFBRAP1	Missense	p.G252V	6	44	13.6%	.	.	.
GC13	2	106471565	C	A	NCK2	Missense	p.Q16K	7	85	8.2%	.	.	.
GC13	2	107460085	C	A	ST6GAL2	Missense	p.G117W	7	81	8.6%	.	O	.
GC13	2	108609582	G	T	SLC5A7	Missense	p.L44F	6	67	9.0%	.	.	.
GC13	2	109088132	C	A	GCC2	Missense	p.L783I	9	139	6.5%	.	.	.
GC13	2	109106464	G	T	GCC2	Missense	p.R1408L	8	69	11.6%	.	.	.

GC13	2	109380479	C	A	RANBP2	Missense	p.H1162N	8	94	8.5%	.	.	.
GC13	2	109380846	G	T	RANBP2	Missense	p.R1284M	8	107	7.5%	.	.	.
GC13	2	111875347	G	T	ACOXL	Missense	p.R566L	6	48	12.5%	.	.	.
GC13	2	112686756	G	T	MERTK	Missense	p.G41W	7	91	7.7%	.	.	.
GC13	2	112767558	C	A	MERTK	Missense	p.P665Q	8	101	7.9%	.	.	.
GC13	2	112940391	G	T	FBLN7	Missense	p.G186W	6	65	9.2%	.	.	.
GC13	2	113089658	G	T	ZC3H6	Missense	p.G1055C	7	106	6.6%	.	.	.
GC13	2	113260643	G	T	TTL	Missense	p.G254W	10	110	9.1%	.	.	.
GC13	2	113315637	G	T	POLR1B	Missense	p.G226W	8	91	8.8%	.	.	.
GC13	2	113332992	C	A	POLR1B	Missense	p.Q821K	8	83	9.6%	.	.	.
GC13	2	113590325	C	A	IL1B	Missense	p.R127L	6	84	7.1%	.	.	.
GC13	2	113742582	G	T	IL36G	Missense	p.G121W	8	86	9.3%	.	.	.
GC13	2	116599896	C	A	DPP10	Missense	p.P535Q	8	112	7.1%	.	.	.
GC13	2	120409612	C	A	CFAP221	Missense	p.P794H	7	98	7.1%	.	.	.
GC13	2	122520593	G	T	TSN	Missense	p.R129L	13	153	8.5%	.	.	.
GC13	2	128015197	G	T	ERCC3	Missense	p.P775Q	7	88	8.0%	O	.	.
GC13	2	128050323	G	T	ERCC3	Missense	p.H112N	7	61	11.5%	O	.	.
GC13	2	128281350	G	T	IWS1	Missense	p.P18T	9	104	8.7%	.	.	.
GC13	2	128331542	G	T	MYO7B	Missense	p.G214W	14	122	11.5%	.	.	.
GC13	2	128393337	G	T	MYO7B	Missense	p.R1928L	4	30	13.3%	.	.	.
GC13	2	128408898	C	A	GPR17	Missense	p.L197M	7	58	12.1%	.	.	.
GC13	2	128938606	C	A	UGGT1	Missense	p.P1348Q	7	92	7.6%	.	.	.
GC13	2	130910947	G	T	SMPD4	Missense	p.P594H	7	80	8.8%	.	.	.
GC13	2	130948170	G	T	MZT2B	Missense	p.W100L	6	57	10.5%	.	.	.
GC13	2	131103802	C	A	IMP4	Missense	p.R155S	9	72	12.5%	.	.	.
GC13	2	131487065	G	T	GPR148	Missense	p.W114L	9	77	11.7%	.	.	.
GC13	2	131801136	C	A	ARHGEF4	Missense	p.Q527K	10	111	9.0%	.	.	.
GC13	2	131897777	C	A	PLEKHB2	Missense	p.R111S	4	39	10.3%	.	.	.
GC13	2	133403797	C	A	LYPD1	Missense	p.G31W	6	61	9.8%	.	.	.
GC13	2	135180460	G	T	MGAT5	Missense	p.E588D	7	95	7.4%	.	.	.
GC13	2	135712201	C	A	CCNT2	Missense	p.Q726K	7	92	7.6%	.	.	.

GC13	2	135743674	C	A	MAP3K19	Missense	p.W810L	7	69	10.1%	.	.	.
GC13	2	136409575	G	T	R3HDM1	Missense	p.Q504H	8	111	7.2%	.	O	.
GC13	2	136529993	C	A	UBXN4	Missense	p.R276S	7	102	6.9%	.	.	.
GC13	2	136536536	G	T	UBXN4	Missense	p.G358C	5	67	7.5%	.	.	.
GC13	2	136567266	C	A	LCT	Missense	p.W884L	10	72	13.9%	.	.	.
GC13	2	136570243	G	T	LCT	Missense	p.P664H	6	51	11.8%	.	.	.
GC13	2	136615575	C	A	MCM6	Splicing	c.1363-1G>T	7	83	8.4%	.	.	.
GC13	2	136623824	G	T	MCM6	Missense	p.R269S	7	92	7.6%	.	.	.
GC13	2	136626359	G	T	MCM6	Missense	p.P146Q	7	88	8.0%	.	.	.
GC13	2	136670067	G	T	DARS	Missense	p.P307T	7	96	7.3%	.	.	.
GC13	2	136873185	C	A	CXCR4	Missense	p.G109W	6	72	8.3%	O	.	.
GC13	2	141294213	G	T	LRP1B	Missense	p.Q2527K	7	74	9.5%	O	O	.
GC13	2	141474367	C	A	LRP1B	Missense	p.W1926L	6	76	7.9%	O	.	.
GC13	2	141709503	G	T	LRP1B	Missense	p.P965Q	7	82	8.5%	O	.	.
GC13	2	145147311	G	T	ZEB2	Missense	p.P1094T	9	149	6.0%	.	.	.
GC13	2	145161533	G	T	ZEB2	Missense	p.R229S	6	84	7.1%	.	.	.
GC13	2	145187504	G	T	ZEB2	Missense	p.P55T	8	82	9.8%	.	.	.
GC13	2	148657445	C	A	ACVR2A	Missense	p.P61H	7	93	7.5%	O	.	.
GC13	2	149225934	G	T	MBD5	Missense	p.R141L	8	112	7.1%	.	.	.
GC13	2	149226389	G	T	MBD5	Nonsense	p.G293X	7	81	8.6%	.	.	.
GC13	2	149227883	G	T	MBD5	Missense	p.G791W	6	91	6.6%	.	.	.
GC13	2	149240816	G	T	MBD5	Missense	p.G886W	9	100	9.0%	.	.	.
GC13	2	149402655	G	T	EPC2	Missense	p.K23N	6	57	10.5%	.	.	.
GC13	2	149528612	G	T	EPC2	Splicing	c.1377-1G>T	10	82	12.2%	.	.	.
GC13	2	152107917	G	T	RBM43	Missense	p.Q193K	9	91	9.9%	.	.	.
GC13	2	152292094	G	T	RIF1	Missense	p.G399C	9	102	8.8%	.	.	.
GC13	2	152300086	G	T	RIF1	Missense	p.G617C	8	133	6.0%	.	.	.
GC13	2	152421576	C	A	NEB	Missense	p.M4438I	7	74	9.5%	.	.	.
GC13	2	152466471	G	T	NEB	Missense	p.P3806Q	7	94	7.4%	.	.	.
GC13	2	152521103	C	A	NEB	Missense	p.W1788L	8	99	8.1%	.	.	.
GC13	2	152551124	C	A	NEB	Missense	p.W565L	7	88	8.0%	.	.	.

GC13	2	152579981	C	A	NEB	Missense	p.W211L	7	98	7.1%	.	.	.
GC13	2	152663408	G	T	ARL5A	Missense	p.Q103K	6	41	14.6%	.	.	.
GC13	2	152992067	G	T	STAM2	Missense	p.P252Q	7	99	7.1%	.	.	.
GC13	2	153378473	C	A	FMNL2	Missense	p.P45H	8	112	7.1%	.	.	.
GC13	2	153529534	C	A	PRPF40A	Missense	p.W388L	7	98	7.1%	.	.	.
GC13	2	155566132	G	T	KCNJ3	Missense	p.E240D	5	43	11.6%	.	O	.
GC13	2	157184993	G	T	NR4A2	Missense	p.P306Q	6	65	9.2%	.	.	.
GC13	2	157186056	C	A	NR4A2	Missense	p.G215W	5	47	10.6%	.	.	.
GC13	2	157352608	G	T	GPD2	Missense	p.R52M	8	102	7.8%	.	.	.
GC13	2	158115030	G	T	GALNT5	Missense	p.G146W	6	93	6.5%	.	.	.
GC13	2	159530398	G	T	PKP4	Missense	p.G1005W	8	52	15.4%	.	.	.
GC13	2	160035527	G	T	TANC1	Missense	p.W587L	7	87	8.0%	.	.	.
GC13	2	160087278	C	A	TANC1	Missense	p.Q1580K	8	98	8.2%	.	.	.
GC13	2	160242988	G	T	BAZ2B	Missense	p.P1080Q	10	124	8.1%	.	.	.
GC13	2	160303398	C	A	BAZ2B	Missense	p.M134I	8	105	7.6%	.	.	.
GC13	2	160604839	G	T	MARCH7	Missense	p.Q290H	10	109	9.2%	.	.	.
GC13	2	160605387	C	A	MARCH7	Missense	p.P473Q	6	56	10.7%	.	.	.
GC13	2	160639873	G	T	CD302	Missense	p.H59N	7	96	7.3%	.	.	.
GC13	2	160755424	G	T	LY75	Missense	p.Q81K	6	69	8.7%	.	.	.
GC13	2	160804049	G	T	PLA2R1	Missense	p.P1244Q	8	96	8.3%	.	.	.
GC13	2	162081221	C	A	TANK	Missense	p.H162N	8	134	6.0%	.	.	.
GC13	2	162273186	G	T	TBR1	Missense	p.G89W	7	101	6.9%	.	.	.
GC13	2	162735792	C	A	SLC4A10	Missense	p.P337Q	6	73	8.2%	.	.	.
GC13	2	162738900	G	T	SLC4A10	Missense	p.K350N	7	90	7.8%	.	.	.
GC13	2	163123885	C	A	IFIH1	Missense	p.W968L	8	109	7.3%	.	O	.
GC13	2	163280022	C	A	KCNH7	Missense	p.G653W	7	74	9.5%	.	.	.
GC13	2	165551938	C	A	COBLL1	Missense	p.R655M	8	117	6.8%	.	.	.
GC13	2	166152389	G	T	SCN2A	Missense	p.R19M	8	124	6.5%	.	.	.
GC13	2	166536123	C	A	CSRNP3	Missense	p.Q540K	8	114	7.0%	.	.	.
GC13	2	167060534	G	T	SCN9A	Missense	p.L1558I	8	78	10.3%	.	.	.
GC13	2	167262559	G	T	SCN7A	Missense	p.P1527H	8	97	8.2%	.	.	.

GC13	2	167263061	C	A	SCN7A	Nonsense	p.G1360X	7	98	7.1%	.	.	.
GC13	2	167300111	C	A	SCN7A	Missense	p.G568C	7	92	7.6%	.	.	.
GC13	2	167322382	C	A	SCN7A	Missense	p.M260I	6	78	7.7%	.	O	.
GC13	2	168100510	G	T	XIRP2	Missense	p.G648C	7	71	9.9%	.	.	.
GC13	2	168100862	C	A	XIRP2	Missense	p.P765Q	8	76	10.5%	.	.	.
GC13	2	168104245	C	A	XIRP2	Missense	p.Q1893K	9	98	9.2%	.	.	.
GC13	2	168115091	C	A	XIRP2	Missense	p.Q457K	8	93	8.6%	.	O	.
GC13	2	169547575	G	T	CERS6	Missense	p.W166L	7	94	7.4%	.	.	.
GC13	2	169783803	G	T	ABCB11	Missense	p.Q1161K	8	103	7.8%	.	.	.
GC13	2	169938374	C	A	DHRS9	Missense	p.Q95K	8	38	21.1%	.	.	.
GC13	2	170042197	G	T	LRP2	Missense	p.L3221I	7	92	7.6%	.	.	.
GC13	2	170063626	T	C	LRP2	Missense	p.R2202G	65	96	67.7%	.	O	.
GC13	2	170135979	G	T	LRP2	Missense	p.R490S	7	81	8.6%	.	.	.
GC13	2	170136041	G	T	LRP2	Missense	p.P469Q	6	74	8.1%	.	.	.
GC13	2	170462563	G	T	PPIG	Missense	p.G51W	7	78	9.0%	.	.	.
GC13	2	170531237	G	T	CCDC173	Missense	p.Q171K	9	140	6.4%	.	.	.
GC13	2	170804155	C	A	UBR3	Missense	p.P1005Q	8	95	8.4%	.	.	.
GC13	2	170863699	C	A	UBR3	Missense	p.P1410Q	5	36	13.9%	.	.	.
GC13	2	170917667	C	A	UBR3	Missense	p.H1622N	8	113	7.1%	.	.	.
GC13	2	171242776	G	T	MYO3B	Missense	p.L456F	9	98	9.2%	.	.	.
GC13	2	171702519	G	T	GAD1	Missense	p.R316S	6	56	10.7%	.	.	.
GC13	2	171709260	G	T	GAD1	Missense	p.M407I	6	87	6.9%	.	O	.
GC13	2	171850340	C	A	TLK1	Missense	p.G655W	9	84	10.7%	.	.	.
GC13	2	172569288	G	T	DYNC1I2	Missense	p.W110L	9	124	7.3%	.	.	.
GC13	2	172641848	G	T	SLC25A12	Missense	p.P658Q	7	111	6.3%	.	O	.
GC13	2	172967029	C	A	DLX2	Missense	p.G80W	5	38	13.2%	.	.	.
GC13	2	174055838	G	T	MAP3K20	Missense	p.W172L	7	96	7.3%	.	.	.
GC13	2	175268891	C	A	SCRN3	Missense	p.P194Q	8	89	9.0%	.	.	.
GC13	2	175436843	C	A	WIPF1	Missense	p.L230F	6	52	11.5%	.	.	.
GC13	2	175446125	C	A	WIPF1	Missense	p.G32W	7	82	8.5%	.	.	.
GC13	2	175939524	G	T	ATF2	Missense	p.P386Q	6	97	6.2%	.	O	.

GC13	2	175957847	C	A	ATF2	Missense	p.W318L	8	149	5.4%	.	.	.
GC13	2	176866915	G	T	LNPK	Missense	p.P35Q	5	27	18.5%	.	.	.
GC13	2	176988086	G	T	HOXD9	Missense	p.R197L	6	57	10.5%	.	.	.
GC13	2	176996161	C	A	HOXD8	Missense	p.H231N	8	86	9.3%	.	.	.
GC13	2	178483401	G	T	TTC30A	Missense	p.P10H	7	96	7.3%	.	.	.
GC13	2	178981098	G	T	RBM45	Missense	p.R137L	6	99	6.1%	.	O	.
GC13	2	179197389	G	T	OSBPL6	Missense	p.G111W	9	106	8.5%	.	.	.
GC13	2	179238645	C	A	OSBPL6	Missense	p.P444H	8	126	6.3%	.	.	.
GC13	2	179393565	C	A	TTN	Missense	p.W26573L	9	120	7.5%	.	.	.
GC13	2	179396181	G	T	TTN	Missense	p.P25989H	9	101	8.9%	.	.	.
GC13	2	179398528	C	A	TTN	Missense	p.G25207C	10	74	13.5%	.	.	.
GC13	2	179400375	G	T	TTN	Missense	p.P24591H	10	97	10.3%	.	.	.
GC13	2	179412874	C	A	TTN	Missense	p.W22095L	8	116	6.9%	.	.	.
GC13	2	179423191	G	T	TTN	Missense	p.H19934N	8	123	6.5%	.	.	.
GC13	2	179425444	C	A	TTN	Missense	p.W19407L	8	81	9.9%	.	.	.
GC13	2	179440846	C	A	TTN	Missense	p.R14273L	7	82	8.5%	.	O	.
GC13	2	179445100	G	T	TTN	Missense	p.L13271M	8	117	6.8%	.	O	.
GC13	2	179457285	G	T	TTN	Missense	p.P10751Q	9	135	6.7%	.	.	.
GC13	2	179472337	G	T	TTN	Missense	p.P8628Q	7	84	8.3%	.	.	.
GC13	2	179474966	G	T	TTN	Missense	p.P8031Q	8	88	9.1%	.	.	.
GC13	2	179476647	G	T	TTN	Missense	p.R7732S	7	103	6.8%	.	O	.
GC13	2	179513995	G	T	TTN	Missense	p.P13346Q	9	130	6.9%	.	.	.
GC13	2	179542503	G	T	TTN	Missense	p.P10135H	8	142	5.6%	.	.	.
GC13	2	179560627	C	A	TTN	Missense	p.R9147M	8	137	5.8%	.	.	.
GC13	2	179574485	G	T	TTN	Missense	p.Q8277K	10	84	11.9%	.	.	.
GC13	2	179588160	G	T	TTN	Missense	p.R5979S	7	114	6.1%	.	O	.
GC13	2	179596101	C	A	TTN	Missense	p.G4554W	9	123	7.3%	.	.	.
GC13	2	179597025	C	A	TTN	Missense	p.K4313N	8	96	8.3%	.	O	.
GC13	2	179632584	C	A	TTN	Missense	p.G3079W	8	107	7.5%	.	.	.
GC13	2	179639648	C	A	TTN	Missense	p.G2218C	9	86	10.5%	.	O	.
GC13	2	179640141	C	A	TTN	Missense	p.M2104I	8	81	9.9%	.	O	.

GC13	2	179641178	G	T	TTN	Missense	p.Q1759K	8	135	5.9%	.	O	.
GC13	2	179642195	G	T	TTN	Missense	p.Q1487K	7	61	11.5%	.	.	.
GC13	2	179647731	C	A	TTN	Nonsense	p.G922X	6	64	9.4%	.	.	.
GC13	2	179702413	G	T	CCDC141	Missense	p.P1178Q	8	79	10.1%	.	.	.
GC13	2	179720207	G	T	CCDC141	Missense	p.P976Q	6	51	11.8%	.	.	.
GC13	2	179730535	G	T	CCDC141	Missense	p.R895S	5	40	12.5%	.	.	.
GC13	2	180810329	C	A	CWC22	Missense	p.G752W	7	128	5.5%	.	.	.
GC13	2	182780990	G	T	SSFA2	Missense	p.G722W	8	94	8.5%	.	.	.
GC13	2	182786786	C	A	SSFA2	Missense	p.Q933K	8	96	8.3%	.	.	.
GC13	2	182852624	C	A	PPP1R1C	Missense	p.P47Q	8	101	7.9%	.	.	.
GC13	2	183623598	C	A	DNAJC10	Missense	p.L614M	8	95	8.4%	.	.	.
GC13	2	183790468	G	T	NCKAP1	Missense	p.H1117N	8	137	5.8%	.	.	.
GC13	2	183853815	C	A	NCKAP1	Missense	p.R297L	7	123	5.7%	.	O	.
GC13	2	185463763	G	T	ZNF804A	Missense	p.R26L	7	121	5.8%	.	.	.
GC13	2	185801879	C	A	ZNF804A	Missense	p.H586N	9	98	9.2%	.	.	.
GC13	2	186654024	C	A	FSIP2	Missense	p.Q721K	9	143	6.3%	.	.	.
GC13	2	186654835	C	A	FSIP2	Missense	p.P991H	8	105	7.6%	.	.	.
GC13	2	186655060	G	T	FSIP2	Missense	p.W1066L	8	128	6.3%	.	.	.
GC13	2	186659737	C	A	FSIP2	Missense	p.P2625Q	7	94	7.4%	.	.	.
GC13	2	186662074	G	T	FSIP2	Missense	p.R3404L	8	100	8.0%	.	.	.
GC13	2	186673682	C	A	FSIP2	Missense	p.P6550H	7	84	8.3%	.	.	.
GC13	2	187693097	C	A	ZSWIM2	Missense	p.G506W	7	60	11.7%	.	.	.
GC13	2	187698693	G	T	ZSWIM2	Missense	p.H270N	6	45	13.3%	.	O	.
GC13	2	188245226	G	T	CALCRL	Missense	p.Q126K	7	95	7.4%	.	.	.
GC13	2	189864602	C	A	COL3A1	Missense	p.P755Q	6	72	8.3%	.	.	O
GC13	2	189901433	G	T	COL5A2	Missense	p.P1341Q	7	86	8.1%	.	.	.
GC13	2	190606103	G	T	ANKAR	Missense	p.G1246C	8	97	8.2%	.	.	.
GC13	2	190611225	C	A	ANKAR	Missense	p.H1393N	7	100	7.0%	.	.	.
GC13	2	190619017	C	A	OSGEPL1	Missense	p.G222W	10	121	8.3%	.	.	.
GC13	2	190619040	G	T	OSGEPL1	Missense	p.P214Q	8	109	7.3%	.	.	.
GC13	2	190626165	G	T	OSGEPL1	Missense	p.Q68K	9	84	10.7%	.	.	.

GC13	2	190640388	G	T	ORMDL1	Missense	p.Q77K	6	79	7.6%	.	.	.
GC13	2	190924810	G	T	MSTN	Missense	p.P242Q	10	89	11.2%	.	.	.
GC13	2	191233878	G	T	INPP1	Missense	p.Q172H	6	57	10.5%	.	.	.
GC13	2	196578156	G	T	SLC39A10	Splicing	c.1576-1G>T	7	87	8.0%	.	.	.
GC13	2	196682469	G	T	DNAH7	Missense	p.L3126I	8	120	6.7%	.	.	.
GC13	2	196737054	G	T	DNAH7	Missense	p.R2185S	8	122	6.6%	.	.	.
GC13	2	196756435	G	T	DNAH7	Missense	p.H1664N	8	76	10.5%	.	.	.
GC13	2	196825140	C	A	DNAH7	Missense	p.W912L	9	132	6.8%	.	.	.
GC13	2	196825314	C	A	DNAH7	Missense	p.W854L	8	100	8.0%	.	.	.
GC13	2	197092916	G	T	HECW2	Missense	p.P920Q	6	62	9.7%	.	.	.
GC13	2	197105182	C	A	HECW2	Missense	p.G896V	8	109	7.3%	.	.	.
GC13	2	197585378	C	A	CCDC150	Missense	p.Q749K	5	48	10.4%	.	.	.
GC13	2	197650245	G	T	GTF3C3	Missense	p.Q321K	7	90	7.8%	.	.	.
GC13	2	197791327	G	T	PGAP1	Nonsense	p.S5X	6	84	7.1%	.	.	.
GC13	2	197863136	C	A	ANKRD44	Missense	p.L891F	7	58	12.1%	.	.	.
GC13	2	197866508	C	A	ANKRD44	Missense	p.G802C	6	94	6.4%	.	.	.
GC13	2	198260836	C	A	SF3B1	Missense	p.M1161I	7	88	8.0%	O	.	.
GC13	2	198264832	G	T	SF3B1	Missense	p.P987H	7	85	8.2%	O	.	.
GC13	2	198285179	G	T	SF3B1	Missense	p.P130T	9	143	6.3%	O	.	.
GC13	2	198285223	C	A	SF3B1	Missense	p.R115L	9	146	6.2%	O	O	.
GC13	2	198646547	G	T	BOLL	Missense	p.P16T	7	91	7.7%	.	.	.
GC13	2	198949130	C	A	PLCL1	Missense	p.R297S	7	123	5.7%	.	.	.
GC13	2	200776184	G	T	C2orf69	Missense	p.R8L	5	61	8.2%	.	.	.
GC13	2	200790200	C	A	C2orf69	Missense	p.P250Q	7	80	8.8%	.	.	.
GC13	2	201354980	C	A	KCTD18	Missense	p.R375L	5	61	8.2%	.	O	.
GC13	2	201438338	C	A	SGO2	Missense	p.P1090Q	9	106	8.5%	.	.	.
GC13	2	201438614	C	A	SGO2	Missense	p.P1182Q	8	69	11.6%	.	.	.
GC13	2	201523958	C	A	AOX1	Missense	p.P1081H	6	60	10.0%	.	.	.
GC13	2	201756848	C	A	NIF3L1	Missense	p.P61Q	7	93	7.5%	.	.	.
GC13	2	202000744	C	A	CFLAR	Missense	p.P50Q	9	122	7.4%	.	.	.
GC13	2	202060670	C	A	CASP10	Missense	p.P228Q	5	53	9.4%	.	.	.

GC13	2	202251103	C	A	TRAK2	Missense	p.G601W	7	93	7.5%	.	.	.
GC13	2	202257715	G	T	TRAK2	Missense	p.Q344K	7	81	8.6%	.	.	.
GC13	2	202260123	C	A	TRAK2	Missense	p.G272W	7	100	7.0%	.	.	.
GC13	2	202358017	G	T	C2CD6	Missense	p.P1016Q	11	66	16.7%	.	.	.
GC13	2	202359451	G	T	C2CD6	Missense	p.P538H	9	126	7.1%	.	.	.
GC13	2	202606500	C	A	ALS2	Missense	p.G750C	8	95	8.4%	.	.	.
GC13	2	202617957	G	T	ALS2	Missense	p.P550Q	7	98	7.1%	.	.	.
GC13	2	202626154	G	T	ALS2	Missense	p.P188Q	8	123	6.5%	.	.	.
GC13	2	202737010	C	A	CDK15	Missense	p.P340Q	8	101	7.9%	.	.	.
GC13	2	203162141	C	A	NOP58	Missense	p.R371S	7	89	7.9%	.	.	.
GC13	2	203972634	C	A	NBEAL1	Missense	p.R529S	7	89	7.9%	.	.	.
GC13	2	203974916	C	A	NBEAL1	Missense	p.H636N	8	98	8.2%	.	.	.
GC13	2	203977768	G	T	NBEAL1	Missense	p.G716W	8	97	8.2%	.	.	.
GC13	2	203977877	G	T	NBEAL1	Missense	p.W752L	9	97	9.3%	.	.	.
GC13	2	204045162	G	T	NBEAL1	Missense	p.M2145I	9	143	6.3%	.	.	.
GC13	2	204261618	G	T	ABI2	Missense	p.G315W	9	101	8.9%	.	.	.
GC13	2	204737480	C	A	CTLA4	Missense	p.P206Q	10	84	11.9%	.	.	.
GC13	2	206305303	G	T	PARD3B	Missense	p.R915L	8	83	9.6%	.	.	.
GC13	2	206641182	G	T	NRP2	Missense	p.G885W	7	96	7.3%	.	.	.
GC13	2	206869148	C	A	INO80D	Missense	p.G1010C	7	69	10.1%	.	.	.
GC13	2	206921629	G	T	INO80D	Missense	p.P86Q	6	73	8.2%	.	.	.
GC13	2	207012523	G	T	NDUFS1	Missense	p.P14Q	9	82	11.0%	.	.	.
GC13	2	207171149	G	T	ZDBF2	Missense	p.G633W	7	87	8.0%	.	.	.
GC13	2	207172538	C	A	ZDBF2	Missense	p.Q1096K	9	105	8.6%	.	.	.
GC13	2	207395659	C	A	ADAM23	Missense	p.P187Q	7	65	10.8%	.	O	.
GC13	2	207436511	G	T	ADAM23	Missense	p.G543W	8	112	7.1%	.	O	.
GC13	2	207527785	G	T	DYTN	Missense	p.P492H	10	120	8.3%	.	.	.
GC13	2	207625629	C	A	MDH1B	Missense	p.W44L	10	129	7.8%	.	.	.
GC13	2	207825627	G	T	CPO	Missense	p.G179W	8	108	7.4%	.	.	.
GC13	2	207833909	G	T	CPO	Missense	p.G292W	8	89	9.0%	.	.	.
GC13	2	208432247	C	A	CREB1	Missense	p.Q108K	7	62	11.3%	.	.	.

GC13	2	209218841	G	T	PIKFYVE	Missense	p.G2022W	8	85	9.4%	.	.	.
GC13	2	210559173	C	A	MAP2	Missense	p.P760H	6	55	10.9%	.	.	.
GC13	2	210569206	G	T	MAP2	Missense	p.Q183H	7	100	7.0%	.	.	.
GC13	2	210570307	G	T	MAP2	Nonsense	p.G174X	5	65	7.7%	.	.	.
GC13	2	210791693	G	T	UNC80	Missense	p.R1864L	7	95	7.4%	.	.	.
GC13	2	210837016	C	A	UNC80	Missense	p.P2717Q	7	80	8.8%	.	.	.
GC13	2	210882204	G	T	RPE	Missense	p.W94L	5	35	14.3%	.	.	.
GC13	2	210889861	C	A	KANSL1L	Missense	p.W802L	7	73	9.6%	.	O	.
GC13	2	211018238	G	T	KANSL1L	Missense	p.L357I	8	116	6.9%	.	.	.
GC13	2	211057537	G	T	ACADL	Missense	p.P397Q	8	96	8.3%	.	.	.
GC13	2	211444487	G	T	CPS1	Missense	p.R174L	6	75	8.0%	.	.	.
GC13	2	211447353	G	T	CPS1	Missense	p.G181W	8	106	7.5%	.	.	.
GC13	2	211503934	G	T	CPS1	Missense	p.G513C	6	83	7.2%	.	.	.
GC13	2	212289014	C	A	ERBB4	Missense	p.W911L	5	42	11.9%	O	.	.
GC13	2	212576809	C	A	ERBB4	Missense	p.G364W	10	92	10.9%	O	O	.
GC13	2	214182010	C	A	SPAG16	Missense	p.Q156K	9	118	7.6%	.	.	.
GC13	2	215440482	G	T	VWC2L	Missense	p.G203W	7	87	8.0%	.	O	.
GC13	2	215646045	G	T	BARD1	Missense	p.P166T	10	137	7.3%	.	.	.
GC13	2	215839577	G	T	ABCA12	Missense	p.P1480Q	6	98	6.1%	.	.	.
GC13	2	215846958	G	T	ABCA12	Missense	p.P1193Q	7	73	9.6%	.	.	.
GC13	2	215854178	C	A	ABCA12	Missense	p.W917L	8	108	7.4%	.	.	.
GC13	2	215875109	C	A	ABCA12	Missense	p.L488F	8	79	10.1%	.	.	.
GC13	2	216251556	C	A	FN1	Missense	p.G1399W	9	79	11.4%	.	.	.
GC13	2	216271096	C	A	FN1	Missense	p.G951W	7	67	10.4%	.	.	.
GC13	2	217057441	G	T	XRCC5	Missense	p.W675L	8	66	12.1%	.	.	.
GC13	2	217347593	C	A	SMARCAL1	Missense	p.Q920K	12	93	12.9%	.	.	.
GC13	2	218937199	C	A	RUFY4	Missense	p.H86N	5	39	12.8%	.	.	.
GC13	2	219028992	C	A	CXCR1	Nonsense	p.G315X	7	97	7.2%	.	.	.
GC13	2	219029493	G	T	CXCR1	Missense	p.Q148K	8	114	7.0%	.	.	.
GC13	2	219127769	G	T	GPBAR1	Missense	p.G108W	8	81	9.9%	.	.	.
GC13	2	219130369	G	T	AAMP	Missense	p.Q306K	7	94	7.4%	.	.	.

GC13	2	219135313	C	A	PNKD	Missense	p.R19S	5	41	12.2%	.	.	.
GC13	2	219232171	C	A	CATIP	Missense	p.P295Q	7	60	11.7%	.	.	.
GC13	2	219232590	C	A	CATIP	Missense	p.P367Q	5	36	13.9%	.	.	.
GC13	2	219301928	C	A	VIL1	Missense	p.H685N	6	64	9.4%	.	.	.
GC13	2	219513996	C	A	ZNF142	Missense	p.R212L	7	66	10.6%	.	.	.
GC13	2	219603276	C	A	TTLL4	Missense	p.H293N	7	109	6.4%	.	.	.
GC13	2	219609945	G	T	TTLL4	Missense	p.R592L	6	58	10.3%	.	.	.
GC13	2	219677361	G	T	CYP27A1	Missense	p.G245W	7	104	6.7%	.	O	.
GC13	2	219738451	G	T	WNT6	Missense	p.G328W	4	36	11.1%	.	.	.
GC13	2	219894193	G	T	CFAP65	Missense	p.H463N	16	34	47.1%	.	.	.
GC13	2	220088309	G	T	ATG9A	Missense	p.Q533K	6	68	8.8%	.	.	.
GC13	2	220100449	C	A	ANKZF1	Missense	p.P398Q	6	57	10.5%	.	.	.
GC13	2	220104133	C	A	GLB1L	Missense	p.G225C	7	90	7.8%	.	.	.
GC13	2	220104522	C	A	GLB1L	Missense	p.R162L	8	69	11.6%	.	.	.
GC13	2	220345419	G	T	SPEG	Missense	p.C1790F	6	63	9.5%	.	.	.
GC13	2	220370760	G	T	GMPPA	Missense	p.R320L	4	29	13.8%	.	.	.
GC13	2	220401787	G	T	ASIC4	Splicing	c.1611-1G>T	8	91	8.8%	.	.	.
GC13	2	220413913	G	T	TMEM198	Missense	p.R261L	5	68	7.4%	.	.	.
GC13	2	220419364	C	A	OBSL1	Missense	p.G1570W	5	32	15.6%	.	.	.
GC13	2	220421217	C	A	OBSL1	Missense	p.R1432L	6	79	7.6%	.	O	.
GC13	2	222365814	C	A	EPHA4	Missense	p.W250L	7	63	11.1%	.	.	.
GC13	2	223806274	C	A	ACSL3	Missense	p.P689T	7	99	7.1%	.	.	.
GC13	2	224824625	C	A	MRPL44	Missense	p.P185Q	8	86	9.3%	.	.	.
GC13	2	225244673	C	A	FAM124B	Missense	p.G329C	7	92	7.6%	.	.	.
GC13	2	225642926	G	T	DOCK10	Missense	p.Q1905K	8	101	7.9%	.	.	.
GC13	2	225727406	G	T	DOCK10	Missense	p.R548S	7	125	5.6%	.	O	.
GC13	2	226273759	C	A	NYAP2	Missense	p.L55I	7	85	8.2%	.	.	.
GC13	2	228159223	G	T	COL4A3	Missense	p.G1119C	7	96	7.3%	.	.	.
GC13	2	228211968	G	T	MFF	Missense	p.R156L	5	74	6.8%	.	.	.
GC13	2	228552200	C	A	SLC19A3	Missense	p.K468N	7	90	7.8%	.	.	.
GC13	2	230282824	C	A	DNER	Nonsense	p.G537X	7	98	7.1%	.	.	.

GC13	2	230667087	C	A	TRIP12	Missense	p.M684I	7	78	9.0%	.	.	.
GC13	2	230723686	G	T	TRIP12	Missense	p.R235S	8	98	8.2%	.	.	.
GC13	2	230923992	G	T	SLC16A14	Missense	p.P26Q	7	81	8.6%	.	.	.
GC13	2	231072711	G	T	SP110	Missense	p.P298Q	9	83	10.8%	.	.	.
GC13	2	231314950	C	A	SP100	Missense	p.P242Q	6	86	7.0%	.	.	.
GC13	2	231339116	G	T	SP100	Missense	p.M518I	7	49	14.3%	.	.	.
GC13	2	231943451	C	A	PSMD1	Missense	p.Q384K	7	97	7.2%	.	.	.
GC13	2	231973752	C	A	HTR2B	Missense	p.G309W	8	127	6.3%	.	.	.
GC13	2	233709103	G	T	GIGYF2	Missense	p.G1036W	10	110	9.1%	.	.	.
GC13	2	234186289	C	A	ATG16L1	Missense	p.R181S	7	93	7.5%	.	.	.
GC13	2	234186305	G	T	ATG16L1	Missense	p.W186L	8	106	7.5%	.	.	.
GC13	2	234198521	G	T	ATG16L1	Missense	p.G246W	6	48	12.5%	.	.	.
GC13	2	234346931	G	T	DGKD	Missense	p.G287W	7	57	12.3%	.	.	.
GC13	2	234377078	G	T	DGKD	Missense	p.W1101L	6	52	11.5%	.	.	.
GC13	2	234402142	C	A	USP40	Missense	p.W960L	7	77	9.1%	.	.	.
GC13	2	234460079	C	A	USP40	Missense	p.K272N	8	135	5.9%	.	.	.
GC13	2	234669351	C	A	UGT1A1	Missense	p.L140M	7	90	7.8%	.	.	.
GC13	2	234722399	C	A	MROH2A	Missense	p.P915Q	6	63	9.5%	.	.	.
GC13	2	234726726	C	A	MROH2A	Missense	p.R1017S	6	89	6.7%	.	.	.
GC13	2	234750330	G	T	HJURP	Missense	p.Q281K	7	84	8.3%	.	.	.
GC13	2	234750782	C	A	HJURP	Missense	p.W130L	7	73	9.6%	.	.	.
GC13	2	235949681	G	T	SH3BP4	Missense	p.G90C	6	65	9.2%	.	.	.
GC13	2	235950899	G	T	SH3BP4	Missense	p.G496W	10	108	9.3%	.	.	.
GC13	2	235951350	C	A	SH3BP4	Missense	p.P646Q	7	95	7.4%	.	.	.
GC13	2	236659019	C	A	AGAP1	Missense	p.P187Q	6	90	6.7%	.	.	.
GC13	2	236708026	G	T	AGAP1	Missense	p.G273W	5	24	20.8%	.	.	.
GC13	2	236949461	C	A	AGAP1	Missense	p.H570N	6	58	10.3%	.	.	.
GC13	2	238253428	C	A	COL6A3	Missense	p.E1804D	9	94	9.6%	.	.	.
GC13	2	238455270	G	T	MLPH	Missense	p.G401W	7	86	8.1%	.	.	.
GC13	2	238738005	G	T	RBM44	Missense	p.G917W	7	95	7.4%	.	.	.
GC13	2	239096824	G	T	ILKAP	Missense	p.H125N	5	48	10.4%	.	.	.

GC13	2	239102939	G	T	ILKAP	Missense	p.P52Q	7	90	7.8%	.	.	.
GC13	2	240048300	C	A	HDAC4	Missense	p.R457L	5	33	15.2%	.	.	.
GC13	2	241398459	G	T	GPC1	Missense	p.R60L	6	78	7.7%	.	O	.
GC13	2	241451336	G	T	ANKMY1	Missense	p.P513Q	4	25	16.0%	.	.	.
GC13	2	242003028	C	A	SNED1	Missense	p.P799Q	5	65	7.7%	.	.	.
GC13	2	242065674	G	A	PASK	Nonsense	p.Q886X	23	38	60.5%	.	.	.
GC13	2	242076549	C	A	PASK	Missense	p.R336L	4	36	11.1%	.	.	.
GC13	2	242179082	G	T	HDLBP	Missense	p.L816I	6	64	9.4%	.	.	.
GC13	2	242274601	C	A	SEPT2	Missense	p.P24Q	6	68	8.8%	.	.	.
GC13	2	242403313	G	T	FARP2	Splicing	c.1812-1G>T;NM_001282984	7	88	8.0%	.	.	.
GC13	3	382476	G	T	CHL1	Splicing	c.386-1G>T;NM_001253388	5	34	14.7%	.	.	.
GC13	3	391189	G	T	CHL1	Missense	p.L332F	6	66	9.1%	.	.	.
GC13	3	3078937	C	A	CNTN4	Missense	p.R344S	6	83	7.2%	.	.	.
GC13	3	3137065	C	A	IL5RA	Missense	p.W258L	6	56	10.7%	.	.	.
GC13	3	3139673	C	A	IL5RA	Missense	p.W197L	7	77	9.1%	.	.	.
GC13	3	3182228	G	T	TRNT1	Missense	p.R126L	7	86	8.1%	.	.	.
GC13	3	4711396	C	A	ITPR1	Missense	p.Q634K	7	84	8.3%	.	.	.
GC13	3	5025010	G	T	BHLHE40	Missense	p.R291L	9	112	8.0%	.	.	.
GC13	3	5255095	G	T	EDEM1	Missense	p.R591L	7	101	6.9%	.	.	.
GC13	3	9146464	C	A	SRGAP3	Missense	p.R108L	8	91	8.8%	.	.	.
GC13	3	9483873	C	A	SETD5	Missense	p.R341S	6	85	7.1%	.	.	.
GC13	3	9483919	C	A	SETD5	Missense	p.P356Q	7	86	8.1%	.	.	.
GC13	3	9506282	G	T	SETD5	Nonsense	p.G884X	8	104	7.7%	.	.	.
GC13	3	9512311	G	T	SETD5	Nonsense	p.G965X	8	108	7.4%	.	.	.
GC13	3	9594227	C	A	LHFPL4	Missense	p.W46L	7	68	10.3%	.	O	.
GC13	3	9780825	C	A	BRPF1	Missense	p.L248I	7	87	8.0%	.	.	.
GC13	3	9787313	C	A	BRPF1	Missense	p.P941Q	7	43	16.3%	.	.	.
GC13	3	10130160	G	T	FANCD2	Missense	p.R1165L	6	58	10.3%	O	.	.
GC13	3	10291167	G	T	TATDN2	Missense	p.G95W	7	81	8.6%	.	.	.
GC13	3	10391843	C	A	ATP2B2	Missense	p.R741L	6	50	12.0%	.	.	.

GC13	3	11301948	C	A	HRH1	Missense	p.R409S	6	68	8.8%	.	.	.
GC13	3	11350462	G	T	ATG7	Missense	p.W113L	8	105	7.6%	.	.	.
GC13	3	11399970	C	A	ATG7	Missense	p.R416S	6	77	7.8%	.	.	.
GC13	3	11468362	G	T	ATG7	Missense	p.G654C	8	118	6.8%	.	.	.
GC13	3	12434213	G	T	PPARG	Missense	p.R166L	7	109	6.4%	.	.	.
GC13	3	12531483	G	T	TSEN2	Missense	p.G62W	8	102	7.8%	.	.	.
GC13	3	12785426	C	A	TMEM40	Nonsense	p.G108X	7	56	12.5%	.	.	.
GC13	3	13370363	C	A	NUP210	Missense	p.L1398F	8	44	18.2%	.	.	.
GC13	3	13916596	C	A	WNT7A	Missense	p.R49L	7	76	9.2%	.	.	.
GC13	3	14485345	C	A	SLC6A6	Missense	p.P68Q	6	65	9.2%	.	.	.
GC13	3	14489201	G	T	SLC6A6	Missense	p.W159L	7	78	9.0%	.	.	.
GC13	3	15094093	G	T	MRPS25	Missense	p.P126Q	7	75	9.3%	.	.	.
GC13	3	15283018	C	A	CAPN7	Missense	p.Q571K	9	89	10.1%	.	.	.
GC13	3	15288300	G	T	CAPN7	Missense	p.R691L	7	52	13.5%	.	.	.
GC13	3	17255863	C	A	TBC1D5	Splicing	c.1655-1G>T;NM_001349073	5	36	13.9%	.	.	.
GC13	3	19959783	G	T	EFHB	Missense	p.P373Q	8	110	7.3%	.	.	.
GC13	3	20153276	C	A	KAT2B	Missense	p.P347Q	7	92	7.6%	.	.	.
GC13	3	20202643	G	T	SGO1	Missense	p.P201Q	8	108	7.4%	.	.	.
GC13	3	20212681	C	A	SGO1	Missense	p.K173N	9	91	9.9%	.	.	.
GC13	3	21465520	C	A	ZNF385D	Missense	p.G297W	8	76	10.5%	.	.	.
GC13	3	23934624	C	A	NKIRAS1	Missense	p.G181W	8	119	6.7%	.	.	.
GC13	3	23952393	C	A	NKIRAS1	Missense	p.M1I	10	127	7.9%	.	.	.
GC13	3	24006526	G	T	NR1D2	Missense	p.W327L	7	87	8.0%	.	.	.
GC13	3	25215988	G	T	RARB	Nonsense	p.G34X	7	74	9.5%	.	.	.
GC13	3	27490255	G	T	SLC4A7	Missense	p.P55Q	7	88	8.0%	.	.	.
GC13	3	29323175	G	T	RBMS3	Missense	p.M1I	7	54	13.0%	.	.	.
GC13	3	29925698	G	T	RBMS3	Nonsense	p.G263X	9	105	8.6%	.	.	.
GC13	3	30045349	C	A	RBMS3	Missense	p.P419Q	9	158	5.7%	.	.	.
GC13	3	31661227	G	T	STT3B	Missense	p.W411L	6	86	7.0%	.	.	.
GC13	3	32754733	C	A	CNOT10	Missense	p.Q149K	10	141	7.1%	.	.	.
GC13	3	32932471	C	A	TRIM71	Missense	p.P592Q	7	62	11.3%	.	.	.

GC13	3	33576725	C	A	CLASP2	Missense	p.D1051Y	8	124	6.5%	.	.	.
GC13	3	36422175	G	T	STAC	Missense	p.G14W	5	48	10.4%	.	.	.
GC13	3	36872940	G	T	TRANK1	Missense	p.Q2668K	11	88	12.5%	.	.	.
GC13	3	36873119	G	T	TRANK1	Missense	p.P2608H	7	60	11.7%	.	.	.
GC13	3	37032956	G	T	EPM2AIP1	Missense	p.P538Q	9	118	7.6%	.	.	.
GC13	3	37366033	C	A	GOLGA4	Missense	p.Q886K	8	116	6.9%	.	.	.
GC13	3	37368556	C	A	GOLGA4	Missense	p.Q1727K	9	103	8.7%	.	.	.
GC13	3	37458965	C	A	C3orf35	Missense	p.L70M	8	88	9.1%	.	.	.
GC13	3	37826486	C	A	ITGA9	Missense	p.Q936K	6	59	10.2%	.	.	.
GC13	3	38017318	G	T	CTDSPL	Missense	p.R202L	5	74	6.8%	.	.	.
GC13	3	38052770	C	A	PLCD1	Missense	p.R263L	7	90	7.8%	.	.	.
GC13	3	38105372	G	T	DLEC1	Missense	p.G379W	8	129	6.2%	.	.	.
GC13	3	38125651	G	T	DLEC1	Missense	p.M392I	7	80	8.8%	.	.	.
GC13	3	38139036	G	T	DLEC1	Missense	p.G825W	6	59	10.2%	.	.	.
GC13	3	38158048	G	T	DLEC1	Missense	p.G1324W	5	57	8.8%	.	O	.
GC13	3	38169284	G	T	ACAA1	Missense	p.Q174K	8	123	6.5%	.	.	.
GC13	3	38173488	G	T	ACAA1	Missense	p.P111Q	8	97	8.2%	.	.	.
GC13	3	38287644	G	T	OXSR1	Missense	p.G397W	8	115	7.0%	.	.	.
GC13	3	38307512	G	T	SLC22A13	Missense	p.W54L	9	102	8.8%	.	.	.
GC13	3	38317813	G	T	SLC22A13	Missense	p.G425W	6	49	12.2%	.	.	.
GC13	3	38417660	C	A	XYLB	Missense	p.R216S	6	83	7.2%	.	.	.
GC13	3	38739453	G	T	SCN10A	Missense	p.P1655Q	6	86	7.0%	.	.	.
GC13	3	38889128	C	A	SCN11A	Missense	p.R1478L	6	83	7.2%	.	.	.
GC13	3	38949549	G	T	SCN11A	Missense	p.P455Q	7	86	8.1%	.	.	.
GC13	3	39225964	C	A	XIRP1	Missense	p.R341L	7	60	11.7%	.	.	.
GC13	3	39374322	C	A	CCR8	Missense	p.P167Q	7	75	9.3%	.	.	.
GC13	3	39453212	C	A	RPSA	Missense	p.R196S	4	27	14.8%	.	.	.
GC13	3	40529685	G	T	ZNF619	Missense	p.G518W	10	141	7.1%	.	.	.
GC13	3	40557458	C	A	ZNF620	Missense	p.Q11K	8	88	9.1%	.	O	.
GC13	3	41977390	G	T	ULK4	Missense	p.P94Q	8	115	7.0%	.	.	.
GC13	3	42201876	G	T	TRAK1	Missense	p.G8V	7	94	7.4%	.	.	.

GC13	3	42201929	C	A	TRAK1	Missense	p.H26N	8	128	6.3%	.	O	.
GC13	3	44284882	C	A	TOPAZ1	Missense	p.P295Q	7	94	7.4%	.	.	.
GC13	3	44286509	G	T	TOPAZ1	Missense	p.R837S	8	95	8.4%	.	.	.
GC13	3	44369766	C	A	TOPAZ1	Missense	p.P1567Q	12	96	12.5%	.	.	.
GC13	3	44489668	G	T	ZNF445	Missense	p.H499N	7	92	7.6%	.	.	.
GC13	3	44489746	G	T	ZNF445	Missense	p.Q473K	8	85	9.4%	.	.	.
GC13	3	44683629	C	A	ZNF197	Missense	p.P123Q	9	118	7.6%	.	.	.
GC13	3	44700873	G	T	ZNF35	Missense	p.G340W	7	87	8.0%	.	.	.
GC13	3	44762695	G	T	ZNF502	Missense	p.W129L	7	96	7.3%	.	.	.
GC13	3	44762958	G	T	ZNF502	Missense	p.G217W	7	56	12.5%	.	.	.
GC13	3	44844420	C	A	KIF15	Missense	p.Q542K	7	68	10.3%	.	.	.
GC13	3	45132961	C	A	CDCP1	Missense	p.R566L	8	52	15.4%	.	.	.
GC13	3	45557736	C	A	LARS2	Missense	p.P671H	5	39	12.8%	.	.	.
GC13	3	45988910	G	T	CXCR6	Missense	p.G313W	9	105	8.6%	.	.	.
GC13	3	46010045	G	T	FYCO1	Missense	p.Q261K	6	68	8.8%	.	.	.
GC13	3	46399343	G	T	CCR2	Missense	p.G109W	8	124	6.5%	.	O	.
GC13	3	46571446	C	A	LRRC2	Missense	p.W241L	10	107	9.3%	.	.	.
GC13	3	46935460	C	A	PTH1R	Missense	p.Q47K	7	78	9.0%	.	.	.
GC13	3	47047500	G	T	NBEAL2	Missense	p.R2289L	6	75	8.0%	.	.	.
GC13	3	47049038	C	A	NBEAL2	Missense	p.P2453Q	6	54	11.1%	.	.	.
GC13	3	47125481	G	T	SETD2	Missense	p.P1886Q	7	91	7.7%	O	O	.
GC13	3	47144841	G	T	SETD2	Missense	p.Q1594K	9	141	6.4%	O	.	.
GC13	3	47165273	G	T	SETD2	Missense	p.H241N	10	115	8.7%	O	.	.
GC13	3	47316878	G	T	KIF9	Missense	p.Q66K	10	112	8.9%	.	.	.
GC13	3	47453061	C	A	PTPN23	Missense	p.P1132Q	7	59	11.9%	.	.	.
GC13	3	47663795	G	T	SMARCC1	Missense	p.L895M	9	152	5.9%	.	.	.
GC13	3	47957697	C	A	MAP4	Missense	p.M540I	7	89	7.9%	.	.	.
GC13	3	48228255	G	T	CDC25A	Missense	p.P62Q	6	79	7.6%	.	.	.
GC13	3	48611922	G	T	COL7A1	Missense	p.P2152Q	7	81	8.6%	.	.	.
GC13	3	48615799	C	A	COL7A1	Splicing	c.5488-1G>T	6	42	14.3%	.	.	.
GC13	3	48667505	C	A	SLC26A6	Missense	p.R364L	4	28	14.3%	.	.	.

GC13	3	48683302	C	A	CELSR3	Splicing	c.7464-1G>T	6	46	13.0%	.	.	.
GC13	3	48698996	C	A	CELSR3	Missense	p.G358W	6	72	8.3%	.	.	.
GC13	3	48699856	C	A	CELSR3	Missense	p.R71L	6	69	8.7%	.	.	.
GC13	3	48716593	G	T	NCKIPSD	Missense	p.Q532K	6	38	15.8%	.	.	.
GC13	3	48727117	G	T	IP6K2	Missense	p.R212S	8	108	7.4%	.	.	.
GC13	3	48730498	G	T	IP6K2	Missense	p.P106Q	7	91	7.7%	.	.	.
GC13	3	49049745	C	A	WDR6	Missense	p.R234S	7	92	7.6%	.	.	.
GC13	3	49141865	C	A	QARS	Missense	p.G53W	6	73	8.2%	.	.	.
GC13	3	49153983	G	T	USP19	Missense	p.P279Q	6	96	6.3%	.	.	.
GC13	3	49159740	G	T	LAMB2	Missense	p.P1546Q	9	102	8.8%	.	.	.
GC13	3	49160591	G	T	LAMB2	Missense	p.L1400M	7	89	7.9%	.	.	.
GC13	3	49167401	G	T	LAMB2	Missense	p.H426N	6	38	15.8%	.	.	.
GC13	3	49169068	C	A	LAMB2	Missense	p.G183V	5	31	16.1%	.	.	.
GC13	3	49400050	G	T	RHOA	Missense	p.Q56K	6	67	9.0%	O	.	.
GC13	3	49569020	G	T	DAG1	Missense	p.R359M	7	80	8.8%	.	.	.
GC13	3	49700683	G	T	BSN	Missense	p.G3698W	7	99	7.1%	.	.	.
GC13	3	49700900	G	T	BSN	Missense	p.G3770V	8	73	11.0%	.	.	.
GC13	3	49714417	C	A	APEH	Missense	p.P320Q	7	77	9.1%	.	.	.
GC13	3	49714428	C	A	APEH	Missense	p.P324T	7	71	9.9%	.	.	.
GC13	3	49846985	G	T	UBA7	Missense	p.P693Q	7	87	8.0%	.	.	.
GC13	3	49848720	G	T	UBA7	Missense	p.Q370K	11	123	8.9%	.	.	.
GC13	3	50127870	G	T	RBM5	Missense	p.M1I	7	61	11.5%	.	.	.
GC13	3	50197147	G	T	SEMA3F	Missense	p.R31L	8	108	7.4%	.	.	.
GC13	3	50289931	G	T	GNAI2	Missense	p.M7I	6	53	11.3%	.	.	.
GC13	3	50383008	C	A	ZMYND10	Missense	p.M1I	7	80	8.8%	.	.	.
GC13	3	50385243	G	T	NPRL2	Missense	p.P341H	6	43	14.0%	.	.	.
GC13	3	51297624	G	T	DOCK3	Missense	p.R741L	9	92	9.8%	.	.	.
GC13	3	51387822	G	T	DOCK3	Missense	p.R1369L	9	57	15.8%	.	.	.
GC13	3	51418775	G	T	DOCK3	Missense	p.G1960W	7	86	8.1%	.	.	.
GC13	3	52181037	C	A	POC1A	Missense	p.R177L	5	68	7.4%	.	.	.
GC13	3	52236570	C	A	ALAS1	Missense	p.Q83K	9	110	8.2%	.	.	.

GC13	3	52255675	C	A	TLR9	Missense	p.W886L	8	117	6.8%	.	.	.
GC13	3	52403901	C	A	DNAH1	Missense	p.R2002S	5	36	13.9%	.	.	.
GC13	3	52432066	C	A	DNAH1	Missense	p.Q3993K	8	77	10.4%	.	.	.
GC13	3	52439208	C	A	BAP1	Missense	p.G345V	7	49	14.3%	O	O	.
GC13	3	52728916	G	T	GLT8D1	Missense	p.P354Q	8	85	9.4%	.	.	.
GC13	3	52728940	C	A	GLT8D1	Missense	p.W346L	7	85	8.2%	.	.	.
GC13	3	52731737	C	A	GLT8D1	Missense	p.R110L	7	78	9.0%	.	.	.
GC13	3	52786204	G	T	NEK4	Missense	p.P282H	9	152	5.9%	.	.	.
GC13	3	53139766	G	T	RFT1	Missense	p.Q294K	8	69	11.6%	.	.	.
GC13	3	53757928	G	T	CACNA1D	Missense	p.G668W	9	81	11.1%	O	.	.
GC13	3	53810016	G	T	CACNA1D	Missense	p.G1421W	8	69	11.6%	O	.	.
GC13	3	53835367	G	T	CACNA1D	Missense	p.G1760W	6	50	12.0%	O	.	.
GC13	3	53916122	G	T	ACTR8	Missense	p.Q3K	8	74	10.8%	.	.	.
GC13	3	54958827	C	A	LRTM1	Missense	p.L65F	7	88	8.0%	.	.	.
GC13	3	55768836	C	A	ERC2	Missense	p.R890L	9	111	8.1%	.	.	.
GC13	3	56468705	G	T	ERC2	Missense	p.H111N	9	79	11.4%	.	.	.
GC13	3	56657824	G	T	FAM208A	Missense	p.Q1511K	7	83	8.4%	.	.	.
GC13	3	56672752	C	A	FAM208A	Missense	p.G540C	6	48	12.5%	.	.	.
GC13	3	56763462	C	A	ARHGEF3	Missense	p.G479W	6	86	7.0%	.	.	.
GC13	3	56787576	G	T	ARHGEF3	Missense	p.Q138K	7	87	8.0%	.	.	.
GC13	3	57131876	G	T	IL17RD	Missense	p.Q619K	8	61	13.1%	.	.	.
GC13	3	57144315	C	A	IL17RD	Missense	p.R112L	6	96	6.3%	.	.	.
GC13	3	57269609	G	T	APPL1	Missense	p.G24C	7	93	7.5%	.	.	.
GC13	3	57274556	C	A	APPL1	Missense	p.Q117K	8	114	7.0%	.	.	.
GC13	3	57542141	G	T	PDE12	Missense	p.R12L	5	49	10.2%	.	.	.
GC13	3	57614564	G	T	DENND6A	Missense	p.L557I	8	121	6.6%	.	.	.
GC13	3	58253001	G	T	ABHD6	Missense	p.G69W	7	88	8.0%	.	.	.
GC13	3	58817504	G	T	C3orf67	Missense	p.H640N	6	53	11.3%	.	.	.
GC13	3	61734645	G	T	PTPRG	Missense	p.W60L	7	92	7.6%	.	.	.
GC13	3	62631400	G	T	CADPS	Missense	p.P441Q	5	38	13.2%	.	.	.
GC13	3	63981968	C	A	ATXN7	Missense	p.H679N	7	93	7.5%	.	.	.

GC13	3	64132745	G	T	PRICKLE2	Missense	p.P474Q	11	108	10.2%	.	O	.
GC13	3	64133228	C	A	PRICKLE2	Missense	p.G313V	8	80	10.0%	.	.	.
GC13	3	65342451	G	T	MAGI1	Missense	p.R1331S	6	60	10.0%	.	.	.
GC13	3	66465387	G	T	LRIG1	Missense	p.Q202K	9	65	13.8%	.	.	.
GC13	3	67411097	G	T	SUCLG2	Missense	p.H427N	9	110	8.2%	.	.	.
GC13	3	69168536	C	A	LMOD3	Missense	p.G324C	8	118	6.8%	.	.	.
GC13	3	69230749	G	T	FRMD4B	Missense	p.Q718K	7	99	7.1%	.	.	.
GC13	3	69230779	C	A	FRMD4B	Missense	p.D708Y	5	71	7.0%	.	.	.
GC13	3	71247460	G	T	FOXP1	Missense	p.H25N	6	58	10.3%	.	.	.
GC13	3	72799482	G	T	SHQ1	Missense	p.R563S	7	89	7.9%	.	.	.
GC13	3	75786443	C	A	ZNF717	Missense	p.Q777H	5	39	12.8%	.	.	.
GC13	3	77666843	G	T	ROBO2	Missense	p.R1158L	7	91	7.7%	.	O	.
GC13	3	78700930	G	T	ROBO1	Missense	p.R886S	7	92	7.6%	.	.	.
GC13	3	78767000	G	T	ROBO1	Missense	p.R192S	9	98	9.2%	.	.	.
GC13	3	81586210	G	T	GBE1	Missense	p.P552Q	7	64	10.9%	.	.	.
GC13	3	81630391	C	A	GBE1	Missense	p.M457I	8	109	7.3%	.	.	.
GC13	3	85851302	G	T	CADM2	Missense	p.W56L	7	88	8.0%	.	.	.
GC13	3	87294894	G	T	CHMP2B	Missense	p.G12C	7	94	7.4%	.	.	.
GC13	3	88205567	C	A	C3orf38	Missense	p.R258S	8	86	9.3%	.	.	.
GC13	3	89456433	G	T	EPHA3	Missense	p.G537C	6	75	8.0%	.	.	.
GC13	3	96706285	C	A	EPHA6	Missense	p.R188S	9	85	10.6%	.	.	.
GC13	3	97356800	G	T	EPHA6	Missense	p.M278I	8	90	8.9%	.	O	.
GC13	3	97664663	C	A	RIOX2	Missense	p.M406I	7	92	7.6%	.	.	.
GC13	3	97806531	G	T	OR5AC2	Missense	p.R172M	9	126	7.1%	.	.	.
GC13	3	98506920	G	T	ST3GAL6	Missense	p.G14W	7	63	11.1%	.	.	.
GC13	3	100498994	G	T	ABI3BP	Missense	p.H697N	6	48	12.5%	.	.	.
GC13	3	100547249	C	A	ABI3BP	Missense	p.G750W	7	95	7.4%	.	.	.
GC13	3	100964717	G	T	IMPG2	Missense	p.P491Q	7	132	5.3%	.	.	.
GC13	3	101058943	G	T	SENP7	Missense	p.L621I	7	47	14.9%	.	.	.
GC13	3	101066842	C	A	SENP7	Missense	p.G407W	9	114	7.9%	.	.	.
GC13	3	101231956	C	A	SENP7	Missense	p.G7W	9	128	7.0%	.	.	.

GC13	3	101284301	C	A	TRMT10C	Missense	p.Q226K	6	61	9.8%	.	.	.
GC13	3	101371448	G	T	ZBTB11	Missense	p.H846N	10	116	8.6%	.	.	.
GC13	3	101371658	G	T	ZBTB11	Missense	p.H812N	9	137	6.6%	.	.	.
GC13	3	101384440	G	T	ZBTB11	Missense	p.Q331K	9	123	7.3%	.	.	.
GC13	3	101477094	G	T	CEP97	Missense	p.L489F	8	117	6.8%	.	.	.
GC13	3	101520554	C	A	NXPE3	Missense	p.P190H	7	67	10.4%	.	.	.
GC13	3	101520628	C	A	NXPE3	Missense	p.R215S	6	76	7.9%	.	.	.
GC13	3	101525898	G	T	NXPE3	Missense	p.G284C	7	100	7.0%	.	.	.
GC13	3	101571825	C	A	NFKBIZ	Missense	p.Q186K	8	96	8.3%	.	.	.
GC13	3	101573447	G	T	NFKBIZ	Missense	p.G455W	9	104	8.7%	.	.	.
GC13	3	101576185	G	T	NFKBIZ	Missense	p.R662L	8	127	6.3%	.	.	.
GC13	3	105404164	C	A	CBLB	Missense	p.R685L	7	85	8.2%	O	.	.
GC13	3	107097177	G	T	CCDC54	Missense	p.W248L	9	128	7.0%	.	.	.
GC13	3	107941109	G	T	IFT57	Missense	p.R21S	6	83	7.2%	.	.	.
GC13	3	108072358	C	A	HHLA2	Missense	p.P50H	7	100	7.0%	.	.	.
GC13	3	108724147	G	T	MORC1	Missense	p.Q595K	7	98	7.1%	.	.	.
GC13	3	111296387	G	T	CD96	Missense	p.K194N	8	118	6.8%	.	.	.
GC13	3	111651204	C	A	PHLDB2	Missense	p.P697H	7	46	15.2%	.	.	.
GC13	3	111842476	C	A	GCSAM	Missense	p.L106F	9	106	8.5%	.	.	.
GC13	3	112357653	C	A	CCDC80	Missense	p.R367L	5	73	6.8%	.	.	.
GC13	3	112647849	C	A	CD200R1	Missense	p.G172W	7	82	8.5%	.	.	.
GC13	3	112710030	G	T	GTPBP8	Missense	p.G62W	8	85	9.4%	.	.	.
GC13	3	113003252	G	T	BOC	Missense	p.L909F	8	82	9.8%	.	.	.
GC13	3	113027010	G	T	CFAP44	Missense	p.P1541Q	10	105	9.5%	.	.	.
GC13	3	113304082	G	T	SIDT1	Missense	p.L260F	7	100	7.0%	.	.	.
GC13	3	113375029	C	A	USF3	Missense	p.G1834W	7	59	11.9%	.	.	.
GC13	3	113376407	C	A	USF3	Missense	p.M1374I	8	71	11.3%	.	.	.
GC13	3	113377849	G	T	USF3	Missense	p.Q894K	7	99	7.1%	.	.	.
GC13	3	113503144	G	T	ATP6V1A	Missense	p.M95I	6	56	10.7%	.	.	.
GC13	3	113677317	G	T	ZDHHC23	Missense	p.R383L	6	62	9.7%	.	.	.
GC13	3	113801601	C	A	QTRT2	Missense	p.Q220K	7	92	7.6%	.	.	.

GC13	3	113804687	G	T	QTRT2	Missense	p.R289L	8	105	7.6%	.	.	.
GC13	3	113850179	C	A	DRD3	Missense	p.L264F	9	119	7.6%	.	.	.
GC13	3	113955819	G	T	ZNF80	Missense	p.Q35K	6	71	8.5%	.	.	.
GC13	3	114058108	C	A	ZBTB20	Missense	p.R657L	7	90	7.8%	.	.	.
GC13	3	114058183	C	A	ZBTB20	Missense	p.R632M	7	89	7.9%	.	.	.
GC13	3	119133179	G	T	ARHGAP31	Missense	p.K801N	7	84	8.3%	.	.	.
GC13	3	119217717	C	A	TIMMDC1	Missense	p.P46Q	9	122	7.4%	.	.	.
GC13	3	119217756	G	T	TIMMDC1	Missense	p.R59L	7	108	6.5%	.	.	.
GC13	3	119582331	C	A	GSK3B	Missense	p.R344L	7	49	14.3%	.	.	.
GC13	3	119886946	C	A	GPR156	Missense	p.G456W	7	71	9.9%	.	.	.
GC13	3	119892227	G	T	GPR156	Missense	p.Q338K	8	66	12.1%	.	.	.
GC13	3	119904156	C	A	GPR156	Missense	p.W225L	8	65	12.3%	.	.	.
GC13	3	120050205	C	A	LRRC58	Missense	p.G320W	6	63	9.5%	.	.	.
GC13	3	120067649	G	T	LRRC58	Missense	p.L148M	7	89	7.9%	.	.	.
GC13	3	120357340	C	A	HGD	Missense	p.G323V	8	89	9.0%	.	.	.
GC13	3	120417368	C	A	RABL3	Missense	p.G146W	9	142	6.3%	.	.	.
GC13	3	121168220	C	A	POLQ	Missense	p.M2402I	7	97	7.2%	.	.	.
GC13	3	121190908	C	A	POLQ	Missense	p.R2216L	6	75	8.0%	.	.	.
GC13	3	121207145	G	T	POLQ	Missense	p.H1545N	7	73	9.6%	.	.	.
GC13	3	121342158	C	A	FBXO40	Missense	p.H628N	7	88	8.0%	.	.	.
GC13	3	121395775	G	T	GOLGB1	Missense	p.Q2968K	8	89	9.0%	.	.	.
GC13	3	121410941	G	T	GOLGB1	Missense	p.Q2344K	9	137	6.6%	.	.	.
GC13	3	121415118	C	A	GOLGB1	Nonsense	p.G1338X	8	119	6.7%	.	.	.
GC13	3	121415796	G	T	GOLGB1	Missense	p.R1112S	8	132	6.1%	.	.	.
GC13	3	121416272	G	T	GOLGB1	Missense	p.P953Q	9	116	7.8%	.	.	.
GC13	3	121417341	G	T	GOLGB1	Missense	p.L597I	9	111	8.1%	.	.	.
GC13	3	121641148	G	T	SLC15A2	Missense	p.W229L	8	112	7.1%	.	.	.
GC13	3	121641942	C	A	SLC15A2	Missense	p.P277Q	9	128	7.0%	.	.	.
GC13	3	122145936	C	A	KPNA1	Missense	p.G505W	6	92	6.5%	.	.	.
GC13	3	122259526	C	A	PARP9	Missense	p.G555W	7	96	7.3%	.	.	.
GC13	3	122283302	C	A	DTX3L	Missense	p.P10Q	5	34	14.7%	.	.	.

GC13	3	122288772	G	T	DTX3L	Missense	p.E612D	7	94	7.4%	.	.	.
GC13	3	122354741	G	T	PARP15	Missense	p.G308W	7	88	8.0%	.	.	.
GC13	3	123419472	G	T	MYLK	Missense	p.P772H	7	66	10.6%	.	.	.
GC13	3	123650046	G	T	CCDC14	Missense	p.Q409K	10	85	11.8%	.	.	.
GC13	3	124215192	G	T	KALRN	Missense	p.G1571W	7	84	8.3%	.	.	.
GC13	3	124540235	C	A	ITGB5	Missense	p.L289F	6	72	8.3%	.	.	.
GC13	3	124729328	G	T	HEG1	Missense	p.H1010N	7	63	11.1%	.	.	.
GC13	3	124732577	C	A	HEG1	Missense	p.G616W	10	111	9.0%	.	.	.
GC13	3	124738188	C	A	HEG1	Missense	p.L502F	8	102	7.8%	.	.	.
GC13	3	125166671	C	A	SNX4	Splicing	c.1306-1G>T	6	62	9.7%	.	.	.
GC13	3	125872304	C	A	ALDH1L1	Missense	p.G180W	9	69	13.0%	.	.	.
GC13	3	126226595	C	A	UROC1	Missense	p.G198W	7	55	12.7%	.	.	.
GC13	3	126291272	G	T	TXNRD3NB	Missense	p.H39N	7	61	11.5%	.	.	.
GC13	3	126723739	G	T	PLXNA1	Missense	p.R544L	7	74	9.5%	.	.	.
GC13	3	126751247	G	T	PLXNA1	Missense	p.W1750L	7	50	14.0%	.	.	.
GC13	3	126915669	G	T	C3orf56	Missense	p.L47F	8	76	10.5%	.	.	.
GC13	3	126915967	G	T	C3orf56	Missense	p.G147W	7	68	10.3%	.	.	.
GC13	3	127831808	G	T	RUVBL1	Missense	p.P95Q	6	67	9.0%	.	.	.
GC13	3	128181581	C	A	DNAJB8	Missense	p.G170W	7	64	10.9%	.	.	.
GC13	3	128181787	C	A	DNAJB8	Missense	p.R101L	6	68	8.8%	.	.	.
GC13	3	128350835	G	T	RPN1	Missense	p.Q267K	6	34	17.6%	.	.	.
GC13	3	128356947	C	A	RPN1	Missense	p.G110W	9	93	9.7%	.	.	.
GC13	3	128525403	G	T	RAB7A	Missense	p.L123F	7	74	9.5%	.	.	.
GC13	3	128616476	G	T	ACAD9	Missense	p.G186W	8	87	9.2%	.	.	.
GC13	3	128889324	C	A	CNBP	Missense	p.R171L	8	123	6.5%	.	.	.
GC13	3	128890334	C	A	CNBP	Missense	p.G58C	9	135	6.7%	.	.	.
GC13	3	128971732	G	T	COPG1	Missense	p.R86L	6	85	7.1%	.	.	.
GC13	3	129225266	G	T	IFT122	Missense	p.G778W	6	37	16.2%	.	.	.
GC13	3	129389618	G	T	TMCC1	Missense	p.Q183K	8	88	9.1%	.	O	.
GC13	3	130110390	C	A	COL6A5	Missense	p.H929N	8	72	11.1%	.	.	.
GC13	3	130113939	C	A	COL6A5	Missense	p.Q1067K	8	121	6.6%	.	.	.

GC13	3	130114038	G	T	COL6A5	Missense	p.G1100C	7	85	8.2%	.	.	.
GC13	3	130150734	C	A	COL6A5	Missense	p.L1892M	8	103	7.8%	.	.	.
GC13	3	130159229	G	T	COL6A5	Missense	p.W2016L	7	100	7.0%	.	O	.
GC13	3	130279248	C	A	COL6A6	Missense	p.H14N	9	94	9.6%	.	.	.
GC13	3	130427186	C	A	PIK3R4	Missense	p.G828W	6	64	9.4%	.	.	.
GC13	3	130447376	G	T	PIK3R4	Missense	p.H580N	12	124	9.7%	.	O	.
GC13	3	130452792	C	A	PIK3R4	Missense	p.L350F	8	129	6.2%	.	.	.
GC13	3	130463714	G	T	PIK3R4	Missense	p.R117S	7	98	7.1%	.	.	.
GC13	3	130718433	G	T	ATP2C1	Missense	p.M848I	7	96	7.3%	.	.	.
GC13	3	130887743	C	A	NEK11	Missense	p.Q311K	6	63	9.5%	.	.	.
GC13	3	132244605	G	T	DNAJC13	Missense	p.R2071L	8	107	7.5%	.	.	.
GC13	3	132249841	G	T	DNAJC13	Splicing	c.6526-1G>T	7	95	7.4%	.	.	.
GC13	3	132350248	G	T	ACAD11	Missense	p.L260M	8	117	6.8%	.	.	.
GC13	3	132410103	G	T	NPHP3	Missense	p.L835M	7	74	9.5%	.	.	.
GC13	3	132427060	G	T	NPHP3	Missense	p.P387H	7	98	7.1%	.	.	.
GC13	3	133099900	C	A	TMEM108	Missense	p.H449N	6	57	10.5%	.	.	.
GC13	3	133337215	C	A	TOPBP1	Missense	p.W1145L	8	102	7.8%	.	O	.
GC13	3	134225950	G	T	CEP63	Splicing	c.45-1G>T;NM_001042400	7	78	9.0%	.	.	.
GC13	3	135722273	G	T	PPP2R3A	Missense	p.G645C	9	131	6.9%	.	.	.
GC13	3	135870750	C	A	MSL2	Missense	p.G251W	7	91	7.7%	.	.	.
GC13	3	136046565	G	T	PCCB	Missense	p.M463I	8	67	11.9%	.	.	.
GC13	3	136062752	C	A	STAG1	Missense	p.R1123L	8	49	16.3%	.	.	.
GC13	3	136664878	C	A	NCK1	Missense	p.P163Q	9	96	9.4%	.	.	.
GC13	3	136667134	G	T	NCK1	Missense	p.G261W	8	115	7.0%	.	.	.
GC13	3	137882234	C	A	DBR1	Missense	p.G300W	7	91	7.7%	.	.	.
GC13	3	137893555	G	T	DBR1	Missense	p.P28Q	5	69	7.2%	.	.	.
GC13	3	138219241	C	A	CEP70	Missense	p.D361Y	7	99	7.1%	.	.	.
GC13	3	138374353	C	A	PIK3CB	Missense	p.G543W	8	90	8.9%	.	.	.
GC13	3	138417810	G	T	PIK3CB	Missense	p.P82Q	8	110	7.3%	.	.	.
GC13	3	138452236	C	A	PIK3CB	Missense	p.K339N	7	60	11.7%	.	O	.
GC13	3	138474793	G	T	PIK3CB	Missense	p.P67Q	8	112	7.1%	.	.	.

GC13	3	138724390	C	A	PRR23A	Missense	p.G241W	8	82	9.8%	.	.	.
GC13	3	138762972	G	T	PRR23C	Missense	p.P164Q	7	70	10.0%	.	.	.
GC13	3	139065880	G	T	MRPS22	Missense	p.L111F	7	92	7.6%	.	.	.
GC13	3	139297771	C	A	NMNAT3	Missense	p.R42L	7	74	9.5%	.	.	.
GC13	3	140281957	G	T	CLSTN2	Missense	p.K798N	7	88	8.0%	.	.	.
GC13	3	140409919	G	T	TRIM42	Missense	p.R657L	6	94	6.4%	.	.	.
GC13	3	141011431	G	T	PXYLP1	Missense	p.G276V	7	88	8.0%	.	.	.
GC13	3	141164583	G	T	ZBTB38	Missense	p.R1118L	8	93	8.6%	.	.	.
GC13	3	141499215	G	T	GRK7	Splicing	c.613-1G>T	9	125	7.2%	.	.	.
GC13	3	141499243	G	T	GRK7	Missense	p.G214W	8	115	7.0%	.	.	.
GC13	3	141535708	G	T	GRK7	Missense	p.R493L	7	109	6.4%	.	.	.
GC13	3	141634818	G	T	ATP1B3	Nonsense	p.G180X	6	87	6.9%	.	.	.
GC13	3	142074288	C	A	XRN1	Missense	p.Q1241H	7	98	7.1%	.	O	.
GC13	3	142389896	G	T	PLS1	Missense	p.R99M	7	95	7.4%	.	.	.
GC13	3	142422740	G	T	PLS1	Missense	p.G468W	6	60	10.0%	.	.	.
GC13	3	142742851	C	A	U2SURP	Missense	p.P422Q	7	96	7.3%	.	.	.
GC13	3	142985613	C	A	SLC9A9	Missense	p.E623D	7	74	9.5%	.	.	.
GC13	3	143271292	C	A	SLC9A9	Missense	p.G334V	9	120	7.5%	.	.	.
GC13	3	145795701	C	A	PLOD2	Missense	p.R504M	8	104	7.7%	.	.	.
GC13	3	145806407	G	T	PLOD2	Missense	p.P324Q	6	75	8.0%	.	.	.
GC13	3	147131328	G	T	ZIC1	Missense	p.W445L	7	67	10.4%	.	O	.
GC13	3	148727102	G	T	GYG1	Missense	p.W174L	7	76	9.2%	.	.	.
GC13	3	148876529	C	A	HPS3	Missense	p.R425S	7	123	5.7%	.	.	.
GC13	3	148895690	C	A	CP	Missense	p.M985I	8	125	6.4%	.	.	.
GC13	3	150281322	G	T	EIF2A	Missense	p.G80W	7	80	8.8%	.	.	.
GC13	3	150377806	C	A	ERICH6	Missense	p.W476L	9	119	7.6%	.	.	.
GC13	3	151017886	C	A	GPR87	Missense	p.M1I	9	107	8.4%	.	.	.
GC13	3	151154548	C	A	IGSF10	Missense	p.G628W	9	127	7.1%	.	.	.
GC13	3	151155201	G	T	IGSF10	Missense	p.P410Q	8	103	7.8%	.	.	.
GC13	3	151161361	G	T	IGSF10	Missense	p.Q1792K	7	91	7.7%	.	.	.
GC13	3	151163562	C	A	IGSF10	Missense	p.G1403W	7	97	7.2%	.	.	.

GC13	3	151598771	G	T	SUCNR1	Missense	p.W147L	10	120	8.3%	.	.	.
GC13	3	152165493	G	T	MBNL1	Missense	p.G230C	6	75	8.0%	.	.	.
GC13	3	154055553	G	T	GPR149	Missense	p.Q711K	8	131	6.1%	.	.	.
GC13	3	156422552	C	A	TIPARP	Missense	p.Q536K	7	97	7.2%	.	.	.
GC13	3	157131880	C	A	VEPH1	Splicing	c.697-1G>T;NM_001167912	7	60	11.7%	.	.	.
GC13	3	157188165	C	A	VEPH1	Missense	p.G98W	8	97	8.2%	.	.	.
GC13	3	158371149	G	T	GFM1	Missense	p.L297F	8	102	7.8%	.	.	.
GC13	3	158384175	C	A	GFM1	Missense	p.P534Q	8	114	7.0%	.	.	.
GC13	3	158545119	C	A	MFSD1	Missense	p.H473N	10	130	7.7%	.	.	.
GC13	3	159998558	G	T	IFT80	Missense	p.L384I	9	118	7.6%	.	.	.
GC13	3	160099343	C	A	IFT80	Missense	p.L69F	8	88	9.1%	.	.	.
GC13	3	160143934	C	A	SMC4	Missense	p.P851T	10	139	7.2%	.	.	.
GC13	3	160155765	G	T	TRIM59	Missense	p.H403N	7	76	9.2%	.	.	.
GC13	3	160245761	C	A	KPNA4	Missense	p.W179L	6	36	16.7%	.	.	.
GC13	3	164714370	C	A	SI	Nonsense	p.G1549X	8	91	8.8%	.	.	.
GC13	3	164714385	G	T	SI	Missense	p.R1544S	6	90	6.7%	.	.	.
GC13	3	164725701	G	T	SI	Missense	p.P1422Q	7	79	8.9%	.	.	.
GC13	3	164741478	C	A	SI	Missense	p.M993I	8	110	7.3%	.	.	.
GC13	3	164906038	G	T	SLITRK3	Missense	p.R861S	5	45	11.1%	.	.	.
GC13	3	167159932	G	T	SERPINI2	Missense	p.Q395K	8	80	10.0%	.	.	.
GC13	3	167159957	C	A	SERPINI2	Missense	p.M386I	6	57	10.5%	.	.	.
GC13	3	167745580	G	T	GOLIM4	Missense	p.P492Q	7	69	10.1%	.	.	.
GC13	3	168834269	G	T	MECOM	Missense	p.P276Q	6	89	6.7%	.	.	.
GC13	3	169487214	G	T	ACTRT3	Missense	p.P32Q	8	78	10.3%	.	.	.
GC13	3	169801860	G	T	GPR160	Missense	p.G34W	9	146	6.2%	.	.	.
GC13	3	170723871	C	A	SLC2A2	Missense	p.L93F	8	60	13.3%	.	.	.
GC13	3	170884959	G	T	TNIK	Missense	p.Q292K	7	86	8.1%	.	.	.
GC13	3	171404475	C	A	PLD1	Missense	p.D623Y	7	79	8.9%	.	.	.
GC13	3	171969217	G	T	FNDC3B	Missense	p.G226W	8	93	8.6%	.	.	.
GC13	3	172224569	G	T	TNFSF10	Missense	p.Q187K	9	109	8.3%	.	.	.
GC13	3	172502593	G	T	ECT2	Missense	p.D549Y	7	78	9.0%	.	O	.

GC13	3	172520718	C	A	ECT2	Missense	p.P625H	8	119	6.7%	.	.	.
GC13	3	178928274	C	A	PIK3CA	Missense	p.P487Q	7	88	8.0%	O	.	.
GC13	3	179082937	G	T	MFN1	Missense	p.R226L	5	47	10.6%	.	.	.
GC13	3	179096231	G	T	MFN1	Nonsense	p.E478X	7	74	9.5%	.	.	.
GC13	3	179096579	G	T	MFN1	Nonsense	p.G547X	8	85	9.4%	.	.	.
GC13	3	179137202	C	A	GNB4	Missense	p.W63L	8	97	8.2%	.	.	.
GC13	3	179418868	C	A	USP13	Missense	p.P143Q	9	82	11.0%	.	.	.
GC13	3	179499558	G	T	USP13	Missense	p.M815I	7	97	7.2%	.	.	.
GC13	3	180320114	G	T	TTC14	Missense	p.R22L	7	79	8.9%	.	.	.
GC13	3	180323625	G	T	TTC14	Missense	p.C310F	9	122	7.4%	.	.	.
GC13	3	180327692	G	T	TTC14	Missense	p.G559W	7	89	7.9%	.	.	.
GC13	3	182756896	G	T	MCCC1	Missense	p.P315H	8	110	7.3%	.	.	.
GC13	3	182775103	G	T	MCCC1	Missense	p.P173Q	6	64	9.4%	.	.	.
GC13	3	183013256	G	T	MCF2L2	Missense	p.Q503K	7	99	7.1%	.	.	.
GC13	3	183368439	C	A	KLHL24	Missense	p.H99N	7	93	7.5%	.	.	.
GC13	3	183368676	C	A	KLHL24	Missense	p.H178N	8	130	6.2%	.	.	.
GC13	3	183470041	G	T	YEATS2	Nonsense	p.G384X	9	135	6.7%	.	.	.
GC13	3	183753010	G	T	HTR3D	Missense	p.M37I	7	96	7.3%	.	.	.
GC13	3	183755920	G	T	HTR3D	Missense	p.G85W	7	88	8.0%	.	.	.
GC13	3	183772556	G	T	HTR3C	Missense	p.G39W	7	66	10.6%	.	.	.
GC13	3	183906716	G	T	ABCF3	Splicing	c.900-1G>T;NM_001351299	9	122	7.4%	.	.	.
GC13	3	183956444	G	T	VWA5B2	Missense	p.R444L	6	66	9.1%	.	.	.
GC13	3	183963529	G	T	ALG3	Missense	p.Q42K	7	75	9.3%	.	.	.
GC13	3	183976238	G	T	ECE2	Missense	p.G215W	5	64	7.8%	.	.	.
GC13	3	184034500	G	T	EIF4G1	Splicing	c.148-1G>T	7	87	8.0%	.	.	.
GC13	3	184046468	C	A	EIF4G1	Missense	p.H1140N	7	92	7.6%	.	.	.
GC13	3	184090776	G	T	THPO	Missense	p.P196Q	7	73	9.6%	.	.	.
GC13	3	184299327	C	A	EPHB3	Missense	p.L972M	6	50	12.0%	.	O	.
GC13	3	184552500	G	T	VPS8	Missense	p.K139N	12	148	8.1%	.	.	.
GC13	3	185146472	G	T	MAP3K13	Missense	p.G35W	7	88	8.0%	O	.	.
GC13	3	185369898	G	T	IGF2BP2	Missense	p.P376Q	4	26	15.4%	.	.	.

GC13	3	185637247	G	T	TRA2B	Missense	p.Q154K	7	78	9.0%	.	.	.
GC13	3	185978244	C	A	DGKG	Missense	p.G431W	6	56	10.7%	.	.	.
GC13	3	186281932	C	A	TBCCD1	Missense	p.G63W	8	115	7.0%	.	.	.
GC13	3	186369047	G	T	FETUB	Missense	p.W181L	12	121	9.9%	.	.	.
GC13	3	186395610	G	T	HRG	Missense	p.G506W	7	94	7.4%	.	.	.
GC13	3	186459945	G	T	KNG1	Missense	p.W587L	8	126	6.3%	.	.	.
GC13	3	187088718	C	A	RTP4	Missense	p.Q100K	7	64	10.9%	.	.	.
GC13	3	188327297	G	T	LPP	Missense	p.G260W	7	75	9.3%	.	.	.
GC13	3	188592226	C	A	LPP	Missense	p.R453S	6	95	6.3%	.	.	.
GC13	3	189526115	G	T	TP63	Missense	p.G33C	6	97	6.2%	O	.	.
GC13	3	190026083	C	A	CLDN1	Missense	p.G207W	5	65	7.7%	.	.	.
GC13	3	190366206	G	T	IL1RAP	Missense	p.Q475H	8	109	7.3%	.	.	.
GC13	3	190374215	G	T	IL1RAP	Missense	p.R628L	8	89	9.0%	.	.	.
GC13	3	192992873	C	A	ATP13A5	Missense	p.L1205F	9	98	9.2%	.	.	.
GC13	3	193061824	G	T	ATP13A5	Missense	p.R279S	6	98	6.1%	.	.	.
GC13	3	193120518	G	T	ATP13A4	Missense	p.H1172N	7	79	8.9%	.	O	.
GC13	3	193343977	C	A	OPA1	Missense	p.Q205K	7	98	7.1%	.	.	.
GC13	3	193355814	C	A	OPA1	Missense	p.P279Q	11	78	14.1%	.	.	.
GC13	3	194062860	G	T	CPN2	Missense	p.P191Q	7	76	9.2%	.	.	.
GC13	3	194331739	C	A	TMEM44	Missense	p.W308L	8	78	10.3%	.	.	.
GC13	3	194371650	G	T	LSG1	Missense	p.P460Q	7	93	7.5%	.	.	.
GC13	3	195498599	C	A	MUC4	Missense	p.R102L	5	48	10.4%	.	.	.
GC13	3	195594690	C	A	TNK2	Missense	p.G844W	6	56	10.7%	.	.	.
GC13	3	195610110	C	A	TNK2	Missense	p.R239L	6	64	9.4%	.	.	.
GC13	3	195936290	G	T	ZDHHC19	Missense	p.P122Q	6	69	8.7%	.	.	.
GC13	3	196612512	G	T	SENP5	Missense	p.G154C	7	116	6.0%	.	.	.
GC13	3	196612777	G	T	SENP5	Missense	p.R242L	6	79	7.6%	.	.	.
GC13	3	196626812	G	T	SENP5	Missense	p.R546M	8	102	7.8%	.	.	.
GC13	3	196675185	G	T	PIGZ	Missense	p.H195N	8	88	9.1%	.	.	.
GC13	3	196796090	G	T	DLG1	Missense	p.L578I	6	90	6.7%	.	.	.
GC13	3	196817861	C	A	DLG1	Missense	p.R445L	7	133	5.3%	.	.	.

GC13	3	196867089	G	T	DLG1	Missense	p.P129Q	11	123	8.9%	.	.	.
GC13	3	197751634	C	A	LMLN	Missense	p.P544Q	8	82	9.8%	.	.	.
GC13	4	289304	G	T	ZNF732	Missense	p.P49Q	6	63	9.5%	.	.	.
GC13	4	435591	C	A	ZNF721	Nonsense	p.G889X	6	53	11.3%	.	.	.
GC13	4	499658	C	A	PIGG	Missense	p.P171Q	9	85	10.6%	.	.	.
GC13	4	780389	G	T	CPLX1	Missense	p.P102Q	8	66	12.1%	.	.	.
GC13	4	955800	C	A	DGKQ	Missense	p.R762L	6	67	9.0%	.	.	.
GC13	4	1720072	G	T	TMEM129	Missense	p.R163S	7	81	8.6%	.	O	.
GC13	4	1902784	G	T	NSD2	Missense	p.G135W	8	111	7.2%	.	.	.
GC13	4	1944073	G	T	NSD2	Missense	p.W630L	10	77	13.0%	.	.	.
GC13	4	2082701	G	T	POLN	Missense	p.H791N	6	52	11.5%	.	.	.
GC13	4	2200366	G	T	POLN	Missense	p.P265T	7	88	8.0%	.	.	.
GC13	4	2240484	C	A	HAUS3	Missense	p.R399L	7	118	5.9%	.	.	.
GC13	4	2673929	G	T	FAM193A	Nonsense	p.G430X	6	82	7.3%	.	O	.
GC13	4	2834081	G	T	SH3BP2	Missense	p.R477L	5	46	10.9%	.	.	.
GC13	4	2955371	C	A	NOP14	Missense	p.R205M	8	107	7.5%	.	.	.
GC13	4	2958457	C	A	NOP14	Nonsense	p.E138X	8	128	6.3%	.	.	.
GC13	4	3441212	C	A	RGS12	Missense	p.P734Q	7	53	13.2%	.	.	.
GC13	4	3449227	G	T	HGFAC	Missense	p.R455M	5	37	13.5%	.	.	.
GC13	4	3768887	C	A	ADRA2C	Missense	p.P185Q	6	69	8.7%	.	.	.
GC13	4	4304716	C	A	ZBTB49	Missense	p.Q385K	7	71	9.9%	.	.	.
GC13	4	4322465	C	A	ZBTB49	Missense	p.R574S	7	58	12.1%	.	.	.
GC13	4	6864116	G	T	KIAA0232	Missense	p.L669F	8	119	6.7%	.	.	.
GC13	4	7435784	G	T	PSAPL1	Missense	p.L275M	7	92	7.6%	.	.	.
GC13	4	8031431	C	A	ABLIM2	Missense	p.G142W	6	58	10.3%	.	.	.
GC13	4	8218738	G	T	SH3TC1	Missense	p.R152L	6	75	8.0%	.	.	.
GC13	4	8443012	C	A	TRMT44	Missense	p.R155S	6	86	7.0%	.	.	.
GC13	4	8443051	G	T	TRMT44	Missense	p.G168W	7	113	6.2%	.	.	.
GC13	4	10446227	G	T	ZNF518B	Missense	p.Q576K	8	97	8.2%	.	.	.
GC13	4	10447001	G	T	ZNF518B	Missense	p.P318T	8	125	6.4%	.	.	.
GC13	4	13602999	G	T	BOD1L1	Missense	p.P1842Q	9	128	7.0%	.	.	.

GC13	4	13603393	G	T	BOD1L1	Missense	p.Q1711K	8	98	8.2%	.	O	.
GC13	4	13606453	G	T	BOD1L1	Missense	p.Q691K	7	97	7.2%	.	.	.
GC13	4	15443876	C	A	C1QTNF7	Missense	p.P115Q	7	66	10.6%	.	.	.
GC13	4	15444341	G	T	C1QTNF7	Missense	p.W270L	7	90	7.8%	.	.	.
GC13	4	15818157	G	T	CD38	Missense	p.W86L	11	87	12.6%	.	.	.
GC13	4	15938117	G	T	FGFBP1	Missense	p.Q47K	8	107	7.5%	.	.	.
GC13	4	16165088	G	T	TAPT1	Missense	p.P516Q	9	111	8.1%	.	.	.
GC13	4	17493949	C	A	QDPR	Missense	p.G120C	5	44	11.4%	.	.	.
GC13	4	20525492	G	T	SLIT2	Missense	p.G418W	8	107	7.5%	.	.	.
GC13	4	22390543	C	A	ADGRA3	Missense	p.L917F	9	78	11.5%	.	.	.
GC13	4	23815532	C	A	PPARGC1A	Missense	p.W398L	7	90	7.8%	.	.	.
GC13	4	24838911	G	T	CCDC149	Missense	p.H201N	6	82	7.3%	.	.	.
GC13	4	25278666	G	T	PI4K2B	Missense	p.G435W	7	97	7.2%	.	.	.
GC13	4	25366083	G	T	ZCCHC4	Missense	p.R406M	8	133	6.0%	.	.	.
GC13	4	25384940	C	A	ANAPC4	Missense	p.P98H	7	100	7.0%	.	.	.
GC13	4	25669537	G	T	SLC34A2	Missense	p.G186W	8	86	9.3%	.	.	.
GC13	4	25849023	G	T	SEL1L3	Missense	p.P174Q	8	104	7.7%	.	.	.
GC13	4	26483618	C	A	CCKAR	Missense	p.R310M	8	84	9.5%	.	.	.
GC13	4	36069611	C	A	ARAP2	Missense	p.R1678M	8	98	8.2%	.	.	.
GC13	4	36286033	G	T	DTHD1	Missense	p.R111M	9	102	8.8%	.	.	.
GC13	4	37445291	C	A	NWD2	Missense	p.H561N	8	116	6.9%	.	.	.
GC13	4	37446575	G	T	NWD2	Missense	p.G989C	7	97	7.2%	.	.	.
GC13	4	37903841	G	T	TBC1D1	Missense	p.W42L	7	88	8.0%	.	.	.
GC13	4	39408898	G	T	KLB	Missense	p.W110L	9	115	7.8%	.	.	.
GC13	4	39512141	C	A	UGDH	Missense	p.E98D	9	122	7.4%	.	.	.
GC13	4	39780014	G	T	UBE2K	Missense	p.W137L	13	160	8.1%	.	.	.
GC13	4	40104384	G	T	N4BP2	Missense	p.G307W	7	95	7.4%	.	.	.
GC13	4	40115083	G	T	N4BP2	Missense	p.R540L	6	100	6.0%	.	O	.
GC13	4	40123792	G	T	N4BP2	Missense	p.G1354V	7	96	7.3%	.	.	.
GC13	4	42077792	C	A	SLC30A9	Missense	p.Q513K	6	76	7.9%	.	.	.
GC13	4	42145595	G	T	BEND4	Missense	p.H302N	7	90	7.8%	.	.	.

GC13	4	42526810	G	T	ATP8A1	Missense	p.L578I	7	89	7.9%	.	.	.
GC13	4	44691887	G	T	GUF1	Missense	p.M86I	9	92	9.8%	.	.	.
GC13	4	46125835	C	A	GABRG1	Missense	p.L32F	7	61	11.5%	.	.	.
GC13	4	47746494	G	T	CORIN	Missense	p.Q175K	8	99	8.1%	.	.	.
GC13	4	48384828	G	T	SLAIN2	Missense	p.R369L	7	111	6.3%	.	.	.
GC13	4	48559026	C	A	FRYL	Missense	p.G1412V	7	88	8.0%	.	.	.
GC13	4	52862314	C	A	LRRC66	Missense	p.G292W	6	67	9.0%	.	.	.
GC13	4	54966593	G	T	GSX2	Missense	p.G28W	7	92	7.6%	.	.	.
GC13	4	54968000	G	T	GSX2	Missense	p.G276W	6	80	7.5%	.	.	.
GC13	4	55948737	G	T	KDR	Missense	p.P1243Q	8	121	6.6%	O	O	.
GC13	4	55961751	G	T	KDR	Missense	p.P937H	8	105	7.6%	O	.	.
GC13	4	56236131	G	T	SRD5A3	Missense	p.W277L	8	94	8.5%	.	.	.
GC13	4	56726632	G	T	EXOC1	Missense	p.K60N	9	97	9.3%	.	.	.
GC13	4	56768573	C	A	EXOC1	Missense	p.Q786K	7	84	8.3%	.	.	.
GC13	4	57204783	G	T	AASDH	Missense	p.H543N	7	91	7.7%	.	.	.
GC13	4	57215867	C	A	AASDH	Missense	p.G199W	8	124	6.5%	.	.	.
GC13	4	57357730	C	A	SRP72	Missense	p.Q485K	38	106	35.8%	.	.	.
GC13	4	57366738	C	A	SRP72	Missense	p.P511Q	6	44	13.6%	.	.	.
GC13	4	57777229	C	A	REST	Missense	p.P142H	9	84	10.7%	.	.	.
GC13	4	57797408	G	T	REST	Missense	p.R795M	8	71	11.3%	.	.	.
GC13	4	62936262	G	T	ADGRL3	Missense	p.R1349L	6	83	7.2%	.	.	.
GC13	4	66242747	G	T	EPHA5	Missense	p.L610M	6	46	13.0%	.	.	.
GC13	4	68488608	C	A	UBA6	Missense	p.Q988H	9	152	5.9%	.	.	.
GC13	4	69811088	G	T	UGT2A3	Missense	p.P267Q	6	85	7.1%	.	.	.
GC13	4	70504880	G	T	UGT2A1	Missense	p.P160Q	8	96	8.3%	.	.	.
GC13	4	70512813	C	A	UGT2A1	Missense	p.G184W	7	77	9.1%	.	.	.
GC13	4	70719951	C	A	SULT1E1	Missense	p.W118L	9	109	8.3%	.	.	.
GC13	4	71066251	G	T	ODAM	Missense	p.W154L	7	79	8.9%	.	.	.
GC13	4	71201117	G	T	CABS1	Missense	p.G121W	7	100	7.0%	.	.	.
GC13	4	71232526	G	T	SMR3A	Missense	p.G74W	12	86	14.0%	.	.	.
GC13	4	71232565	G	T	SMR3A	Missense	p.G87W	10	97	10.3%	.	.	.

GC13	4	71255533	G	T	SMR3B	Missense	p.G70W	8	103	7.8%	.	O	.
GC13	4	72205121	G	T	SLC4A4	Missense	p.L52F	7	67	10.4%	.	.	.
GC13	4	72420898	G	T	SLC4A4	Missense	p.M868I	9	99	9.1%	.	.	.
GC13	4	73012769	G	T	NPFFR2	Missense	p.W270L	9	118	7.6%	.	.	.
GC13	4	73178137	G	T	ADAMTS3	Missense	p.Q598K	7	94	7.4%	.	.	.
GC13	4	74304021	C	A	AFP	Missense	p.Q90K	5	49	10.2%	.	.	.
GC13	4	74320988	G	T	AFP	Missense	p.L607F	7	56	12.5%	.	.	.
GC13	4	74347572	G	T	AFM	Missense	p.R27L	11	61	18.0%	.	.	.
GC13	4	74719768	G	T	PF4V1	Missense	p.G82W	9	122	7.4%	.	.	.
GC13	4	74847655	C	A	PF4	Missense	p.G6W	7	58	12.1%	.	.	.
GC13	4	74863805	G	T	CXCL5	Missense	p.L84M	9	85	10.6%	.	.	.
GC13	4	76517356	G	T	CDKL2	Missense	p.P505H	10	117	8.5%	.	.	.
GC13	4	76797508	G	T	PPEF2	Missense	p.R418S	11	53	20.8%	.	.	.
GC13	4	76926008	G	T	CXCL9	Missense	p.P77Q	9	145	6.2%	.	.	.
GC13	4	76956399	G	T	CXCL11	Missense	p.P53Q	9	105	8.6%	.	.	.
GC13	4	77230526	G	T	STBD1	Missense	p.M150I	6	77	7.8%	.	.	.
GC13	4	77662924	C	A	SHROOM3	Missense	p.Q1200K	5	33	15.2%	.	.	.
GC13	4	77940328	C	A	SEPT11	Missense	p.P233Q	8	69	11.6%	.	.	.
GC13	4	77952071	C	A	SEPT11	Missense	p.Q408K	8	80	10.0%	.	.	.
GC13	4	78804530	C	A	MRPL1	Missense	p.P93Q	7	127	5.5%	.	.	.
GC13	4	78830495	G	T	MRPL1	Missense	p.R249M	9	98	9.2%	.	.	.
GC13	4	79166386	G	T	FRAS1	Splicing	c.217-1G>T	7	82	8.5%	.	.	.
GC13	4	79343032	G	T	FRAS1	Missense	p.R1519L	6	74	8.1%	.	.	.
GC13	4	79351453	G	T	FRAS1	Missense	p.Q1617H	7	88	8.0%	.	.	.
GC13	4	79387483	G	T	FRAS1	Missense	p.R2384L	9	88	10.2%	.	O	.
GC13	4	79475641	G	T	ANXA3	Missense	p.W5L	7	98	7.1%	.	.	.
GC13	4	79754809	G	T	BMP2K	Missense	p.W124L	7	88	8.0%	.	.	.
GC13	4	79831947	C	A	BMP2K	Missense	p.P749H	7	100	7.0%	.	.	.
GC13	4	81121266	G	T	PRDM8	Missense	p.W11L	5	45	11.1%	.	.	.
GC13	4	82126161	G	T	PRKG2	Missense	p.P14Q	7	68	10.3%	.	.	.
GC13	4	83349220	G	T	HNRNPDL	Missense	p.P242Q	6	59	10.2%	.	.	.

GC13	4	83719593	G	T	SCD5	Missense	p.P33Q	6	83	7.2%	.	.	.
GC13	4	83763500	C	A	SEC31A	Missense	p.G882W	7	97	7.2%	.	.	.
GC13	4	83838523	G	T	THAP9	Missense	p.L386F	8	121	6.6%	.	.	.
GC13	4	83905960	G	T	LIN54	Missense	p.P13Q	7	90	7.8%	.	.	.
GC13	4	84193241	C	A	COQ2	Missense	p.L259F	7	86	8.1%	.	.	.
GC13	4	84380902	C	A	MRPS18C	Missense	p.Q54K	7	87	8.0%	.	.	.
GC13	4	84518066	G	T	GPAT3	Nonsense	p.G304X	7	96	7.3%	.	.	.
GC13	4	85612860	G	T	WDFY3	Missense	p.P3043Q	8	99	8.1%	.	.	.
GC13	4	85717840	G	T	WDFY3	Missense	p.H1001N	8	79	10.1%	.	.	.
GC13	4	87019720	C	A	MAPK10	Missense	p.M215I	8	92	8.7%	.	.	.
GC13	4	87692457	C	A	PTPN13	Missense	p.P1455Q	8	89	9.0%	O	.	.
GC13	4	87809417	C	A	C4orf36	Missense	p.W26L	6	60	10.0%	.	.	.
GC13	4	88235052	C	A	HSD17B13	Missense	p.L170F	7	82	8.5%	.	.	.
GC13	4	88415495	G	T	SPARCL1	Missense	p.Q28K	8	110	7.3%	.	.	.
GC13	4	88732768	G	T	IBSP	Missense	p.Q220H	7	75	9.3%	.	.	.
GC13	4	89022440	G	T	ABCG2	Missense	p.Q437K	8	93	8.6%	.	.	.
GC13	4	89363482	C	A	HERC6	Missense	p.P944Q	10	128	7.8%	.	.	.
GC13	4	90857328	C	A	MMRN1	Missense	p.Q833K	7	89	7.9%	.	.	.
GC13	4	91230606	C	A	CCSER1	Missense	p.P391T	7	69	10.1%	.	.	.
GC13	4	94751092	G	T	ATOH1	Missense	p.G339W	6	73	8.2%	.	.	.
GC13	4	95174148	G	T	SMARCAD 1	Missense	p.W424L	8	99	8.1%	.	.	.
GC13	4	96073902	C	A	BMPR1B	Missense	p.P454Q	6	66	9.1%	.	.	.
GC13	4	96222888	C	A	UNC5C	Missense	p.R120L	6	87	6.9%	.	.	.
GC13	4	99027144	G	T	STPG2	Missense	p.P191Q	9	110	8.2%	.	.	.
GC13	4	99579371	C	A	TSPAN5	Missense	p.G3W	6	83	7.2%	.	.	.
GC13	4	100052684	G	T	ADH4	Missense	p.L272I	7	90	7.8%	.	O	.
GC13	4	100460421	G	T	C4orf17	Missense	p.G244W	9	99	9.1%	.	.	.
GC13	4	100479253	G	T	TRMT10A	Missense	p.L101I	12	124	9.7%	.	.	.
GC13	4	100485343	C	A	MTTP	Missense	p.R31S	8	108	7.4%	.	.	.
GC13	4	100774444	G	T	DAPP1	Missense	p.R143L	7	64	10.9%	.	.	.
GC13	4	104577450	C	A	TACR3	Missense	p.M263I	6	57	10.5%	.	.	.

GC13	4	106196570	G	T	TET2	Nonsense	p.G1635X	8	120	6.7%	O	.	.
GC13	4	106196822	G	T	TET2	Missense	p.G1719W	8	82	9.8%	O	.	.
GC13	4	106621021	G	T	INTS12	Missense	p.R48S	6	85	7.1%	.	.	.
GC13	4	109790307	C	A	COL25A1	Splicing	c.1009-1G>T;NM_032518	9	97	9.3%	.	.	.
GC13	4	110384689	G	T	SEC24B	Nonsense	p.G256X	8	114	7.0%	.	.	.
GC13	4	110384800	G	T	SEC24B	Missense	p.V293F	7	95	7.4%	.	.	.
GC13	4	110884392	G	T	EGF	Missense	p.W417L	9	97	9.3%	.	.	.
GC13	4	110932390	-	C	EGF	Frameshift	p.P1094fs	55	99	55.6%	.	.	.
GC13	4	111553612	C	G	PITX2	Missense	p.C24S	16	49	32.7%	.	.	.
GC13	4	113352267	C	A	ALPK1	Missense	p.L444I	7	80	8.8%	.	.	.
GC13	4	113475002	G	T	ZGRF1	Missense	p.L1721M	7	99	7.1%	.	.	.
GC13	4	113508681	C	A	ZGRF1	Missense	p.G1120W	9	131	6.9%	.	.	.
GC13	4	113538834	C	A	ZGRF1	Missense	p.L788F	8	81	9.9%	.	.	.
GC13	4	113539050	C	A	ZGRF1	Missense	p.L716F	7	98	7.1%	.	.	.
GC13	4	114275565	G	T	ANK2	Missense	p.G1931W	9	110	8.2%	.	.	.
GC13	4	114275746	G	T	ANK2	Missense	p.R1991L	7	108	6.5%	.	O	.
GC13	4	115751038	G	T	NDST4	Missense	p.Q803K	7	91	7.7%	.	.	.
GC13	4	115997544	C	A	NDST4	Missense	p.G217W	8	94	8.5%	.	.	.
GC13	4	118005773	C	A	TRAM1L1	Missense	p.L259F	7	89	7.9%	.	.	.
GC13	4	119256631	G	T	PRSS12	Missense	p.H273N	9	84	10.7%	.	.	.
GC13	4	119273413	G	T	PRSS12	Missense	p.R155S	6	69	8.7%	.	O	.
GC13	4	119659495	C	A	SEC24D	Missense	p.R807L	5	48	10.4%	.	O	.
GC13	4	120182988	G	T	USP53	Missense	p.W314L	8	105	7.6%	.	O	.
GC13	4	120221534	G	T	C4orf3	Missense	p.L53I	9	109	8.3%	.	O	.
GC13	4	121957874	G	T	NDNF	Missense	p.L418I	8	107	7.5%	.	.	.
GC13	4	122749615	G	T	BBS7	Missense	p.P611Q	9	120	7.5%	.	.	.
GC13	4	122749774	C	A	BBS7	Nonsense	p.E596X	6	100	6.0%	.	.	.
GC13	4	123151254	G	T	KIAA1109	Missense	p.G1071W	6	70	8.6%	.	.	.
GC13	4	123178446	G	T	KIAA1109	Missense	p.G2139C	7	89	7.9%	.	.	.
GC13	4	123192489	C	A	KIAA1109	Missense	p.P2604T	8	128	6.3%	.	.	.
GC13	4	123246414	C	A	KIAA1109	Missense	p.P3645Q	8	91	8.8%	.	.	.

GC13	4	123264750	G	T	KIAA1109	Nonsense	p.G4180X	8	93	8.6%	.	.	.
GC13	4	123274221	C	A	KIAA1109	Missense	p.P4671Q	7	76	9.2%	.	.	.
GC13	4	123663618	G	T	BBS12	Missense	p.G191W	9	114	7.9%	.	.	.
GC13	4	125631653	C	A	ANKRD50	Missense	p.W5L	8	97	8.2%	.	.	.
GC13	4	126238017	G	T	FAT4	Nonsense	p.G151X	5	64	7.8%	O	.	.
GC13	4	126240479	G	T	FAT4	Missense	p.M971I	8	88	9.1%	O	.	.
GC13	4	126242023	G	T	FAT4	Missense	p.R1486L	10	112	8.9%	O	.	.
GC13	4	126336141	G	T	FAT4	Missense	p.R2008L	7	117	6.0%	O	.	.
GC13	4	126369987	G	T	FAT4	Missense	p.G2608W	7	86	8.1%	O	.	.
GC13	4	126373182	C	A	FAT4	Missense	p.Q3673K	7	98	7.1%	O	.	.
GC13	4	128842737	G	T	MFSD8	Missense	p.P431Q	5	50	10.0%	.	.	.
GC13	4	139964494	C	A	NOCT	Missense	p.Q153K	6	80	7.5%	.	O	.
GC13	4	140272719	C	A	NAA15	Missense	p.P323Q	8	106	7.5%	.	.	.
GC13	4	141074336	G	T	MAML3	Missense	p.P49Q	4	25	16.0%	.	.	.
GC13	4	141300791	C	A	SCOC	Missense	p.Q108K	8	77	10.4%	.	.	.
GC13	4	141313418	G	T	CLGN	Missense	p.L536M	10	145	6.9%	.	.	.
GC13	4	141598221	G	T	TBC1D9	Missense	p.R296S	6	74	8.1%	.	.	.
GC13	4	142143568	C	A	ZNF330	Missense	p.R15S	6	85	7.1%	.	.	.
GC13	4	144618692	C	A	FREM3	Missense	p.W1046L	9	95	9.5%	.	.	.
GC13	4	145916543	C	G	ANAPC10	Missense	p.M180I	53	82	64.6%	.	.	.
GC13	4	146058907	G	T	OTUD4	Missense	p.P942H	9	100	9.0%	.	O	.
GC13	4	146576486	G	T	MMAA	Missense	p.R386L	7	66	10.6%	.	.	.
GC13	4	148441012	G	T	EDNRA	Missense	p.G144W	6	64	9.4%	.	O	.
GC13	4	148545055	G	T	TMEM184C	Missense	p.W65L	9	138	6.5%	.	.	.
GC13	4	149035287	G	T	NR3C2	Missense	p.Q806K	5	26	19.2%	.	.	.
GC13	4	151170732	C	A	DCLK2	Missense	p.Q657K	10	85	11.8%	.	.	.
GC13	4	153831276	G	T	ARFIP1	Missense	p.G311W	8	113	7.1%	.	.	.
GC13	4	154191623	G	T	TRIM2	Missense	p.R29L	6	86	7.0%	.	.	.
GC13	4	154524467	G	T	TMEM131L	Missense	p.L884F	8	73	11.0%	.	.	.
GC13	4	154557691	C	A	TMEM131L	Missense	p.P1599Q	8	96	8.3%	.	.	.
GC13	4	154624536	G	T	TLR2	Missense	p.M159I	9	99	9.1%	.	.	.

GC13	4	154626383	G	T	TLR2	Missense	p.W775L	7	76	9.2%	.	.	.
GC13	4	154649378	G	T	RNF175	Missense	p.L128I	6	31	19.4%	.	.	.
GC13	4	155219152	G	T	DCHS2	Missense	p.P1650H	8	72	11.1%	.	.	.
GC13	4	155506759	C	A	FGA	Nonsense	p.G608X	6	99	6.1%	.	.	.
GC13	4	158057795	C	A	GLRB	Missense	p.Q158K	7	96	7.3%	.	.	.
GC13	4	159624602	G	T	ETFDH	Missense	p.G321C	7	110	6.4%	.	.	.
GC13	4	159631914	C	A	PPID	Missense	p.L327F	9	107	8.4%	.	.	.
GC13	4	159753264	G	T	FNIP2	Missense	p.M199I	7	75	9.3%	.	.	.
GC13	4	160162401	C	A	RAPGEF2	Missense	p.H20N	6	73	8.2%	.	.	.
GC13	4	160263060	G	T	RAPGEF2	Missense	p.R799M	8	105	7.6%	.	.	.
GC13	4	160272246	G	T	RAPGEF2	Missense	p.G1140W	7	64	10.9%	.	.	.
GC13	4	164393396	C	A	TKTL2	Missense	p.K497N	8	79	10.1%	.	.	.
GC13	4	164394430	G	T	TKTL2	Missense	p.L153I	7	79	8.9%	.	.	.
GC13	4	165878407	G	T	FAM218A	Missense	p.W78L	7	57	12.3%	.	.	.
GC13	4	169294930	G	T	DDX60L	Missense	p.Q1555K	7	71	9.9%	.	.	.
GC13	4	169337911	C	A	DDX60L	Missense	p.W883L	9	87	10.3%	.	.	.
GC13	4	169847515	G	T	PALLD	Missense	p.L666F	8	104	7.7%	.	.	.
GC13	4	169931135	G	T	CBR4	Missense	p.L36M	5	33	15.2%	.	.	.
GC13	4	170038692	G	T	SH3RF1	Missense	p.R587S	5	65	7.7%	.	.	.
GC13	4	170038914	G	T	SH3RF1	Missense	p.Q513K	11	108	10.2%	.	.	.
GC13	4	170459000	C	A	NEK1	Missense	p.R473L	8	131	6.1%	.	.	.
GC13	4	170506613	G	T	NEK1	Missense	p.R232S	6	91	6.6%	.	.	.
GC13	4	170912791	G	T	MFAP3L	Missense	p.P220Q	9	116	7.8%	.	.	.
GC13	4	174238823	G	T	GALNT7	Missense	p.M549I	9	126	7.1%	.	.	.
GC13	4	175898489	G	T	ADAM29	Missense	p.G605W	6	73	8.2%	.	.	.
GC13	4	175898921	C	A	ADAM29	Missense	p.Q749K	10	143	7.0%	.	.	.
GC13	4	177056350	G	T	WDR17	Missense	p.W397L	8	101	7.9%	.	.	.
GC13	4	177071084	G	T	WDR17	Missense	p.W675L	8	95	8.4%	.	.	.
GC13	4	177089923	C	A	WDR17	Missense	p.L1031I	7	95	7.4%	.	.	.
GC13	4	183601473	C	A	TENM3	Missense	p.P537Q	7	87	8.0%	.	.	.
GC13	4	183676267	C	A	TENM3	Missense	p.Q1583K	8	126	6.3%	.	.	.

GC13	4	183714691	G	T	TENM3	Missense	p.G2289V	8	132	6.1%	.	.	.
GC13	4	184192291	G	T	WWC2	Missense	p.W820L	9	96	9.4%	.	.	.
GC13	4	184930551	G	T	STOX2	Missense	p.R187M	8	99	8.1%	.	.	.
GC13	4	184931371	G	T	STOX2	Missense	p.R460S	7	92	7.6%	.	O	.
GC13	4	184938247	G	T	STOX2	Missense	p.R864L	8	100	8.0%	.	.	.
GC13	4	185074857	C	A	ENPP6	Missense	p.G91W	6	77	7.8%	.	.	.
GC13	4	186112150	C	A	CFAP97	Missense	p.K67N	8	101	7.9%	.	.	.
GC13	4	186283146	G	T	SNX25	Missense	p.R743L	7	112	6.3%	.	.	.
GC13	4	186361806	C	A	C4orf47	Missense	p.P90Q	8	97	8.2%	.	.	.
GC13	4	186545384	G	T	SORBS2	Missense	p.P300Q	7	66	10.6%	.	.	.
GC13	4	186559234	G	T	SORBS2	Missense	p.P302Q	6	75	8.0%	.	.	.
GC13	4	187003647	G	T	TLR3	Missense	p.L269F	8	106	7.5%	.	.	.
GC13	4	187171503	C	A	KLKB1	Missense	p.P23Q	11	113	9.7%	.	.	.
GC13	4	187525668	G	T	FAT1	Missense	p.H3471N	7	75	9.3%	O	.	.
GC13	4	187554978	C	A	FAT1	Splicing	c.4184-1G>T	7	91	7.7%	O	O	.
GC13	4	187630191	G	T	FAT1	Missense	p.P264Q	7	95	7.4%	O	.	.
GC13	5	143353	G	T	PLEKHG4B	Missense	p.G557W	6	89	6.7%	.	.	.
GC13	5	171339	G	T	PLEKHG4B	Missense	p.R1277L	7	78	9.0%	.	.	.
GC13	5	205527	C	A	CCDC127	Missense	p.W223L	7	98	7.1%	.	.	.
GC13	5	354006	G	T	AHRR	Missense	p.R79L	5	33	15.2%	.	.	.
GC13	5	488463	C	A	SLC9A3	Missense	p.G215W	7	82	8.5%	.	.	.
GC13	5	730552	A	G	ZDHHC11B	Missense	p.L352P	12	59	20.3%	.	.	.
GC13	5	886774	G	T	BRD9	Missense	p.P203T	8	105	7.6%	.	.	.
GC13	5	1074734	G	T	SLC12A7	Missense	p.R674S	5	49	10.2%	.	.	.
GC13	5	1244382	G	T	SLC6A18	Missense	p.G464W	7	78	9.0%	.	.	.
GC13	5	1409198	C	A	SLC6A3	Missense	p.G481C	5	46	10.9%	.	.	.
GC13	5	3599703	C	A	IRX1	Missense	p.P214Q	6	71	8.5%	.	O	.
GC13	5	6600301	C	A	NSUN2	Missense	p.R646L	6	100	6.0%	.	.	.
GC13	5	7831983	C	A	C5orf49	Missense	p.G142W	7	68	10.3%	.	.	.
GC13	5	7895949	G	T	MTRR	Missense	p.G554W	8	93	8.6%	.	.	.
GC13	5	9063191	C	A	SEMA5A	Missense	p.G776W	5	28	17.9%	.	.	.

GC13	5	9237992	C	A	SEMA5A	Missense	p.W94L	6	74	8.1%	.	.	.
GC13	5	14389408	G	T	TRIO	Missense	p.W1320L	8	104	7.7%	.	.	.
GC13	5	14508164	G	T	TRIO	Missense	p.W2976L	7	91	7.7%	.	.	.
GC13	5	14713753	C	A	ANKH	Missense	p.G389W	4	22	18.2%	.	.	.
GC13	5	16463848	C	A	ZNF622	Missense	p.W210L	7	89	7.9%	.	.	.
GC13	5	16783577	C	A	MYO10	Missense	p.G157C	7	98	7.1%	.	.	.
GC13	5	31468049	G	T	DROSHA	Missense	p.P751Q	6	78	7.7%	O	.	.
GC13	5	31532543	C	A	C5orf22	Missense	p.P15H	6	60	10.0%	.	.	.
GC13	5	31532552	C	A	C5orf22	Missense	p.P18Q	7	63	11.1%	.	.	.
GC13	5	32057996	G	T	PDZD2	Nonsense	p.G663X	9	104	8.7%	.	.	.
GC13	5	33683184	G	T	ADAMTS12	Missense	p.P285Q	6	59	10.2%	.	.	.
GC13	5	34005962	C	A	AMACR	Missense	p.R97L	6	62	9.7%	.	.	.
GC13	5	35033587	G	T	AGXT2	Missense	p.P218H	8	90	8.9%	.	.	.
GC13	5	35876322	G	T	IL7R	Missense	p.G372W	7	88	8.0%	O	.	.
GC13	5	35965642	G	T	UGT3A1	Missense	p.P230Q	8	106	7.5%	.	.	.
GC13	5	36257117	G	T	RANBP3L	Missense	p.Q253K	7	98	7.1%	.	.	.
GC13	5	36629623	G	T	SLC1A3	Missense	p.G85W	9	119	7.6%	.	.	.
GC13	5	36679893	G	T	SLC1A3	Missense	p.R230L	6	93	6.5%	.	.	.
GC13	5	37002852	G	T	NIPBL	Missense	p.M1251I	11	103	10.7%	.	.	.
GC13	5	37221471	G	T	C5orf42	Missense	p.L901M	8	133	6.0%	.	.	.
GC13	5	37227792	C	A	C5orf42	Nonsense	p.G417X	7	90	7.8%	.	.	.
GC13	5	37370986	G	T	NUP155	Missense	p.R32S	6	97	6.2%	.	.	.
GC13	5	38338867	G	T	EGFLAM	Missense	p.R92L	5	45	11.1%	.	.	.
GC13	5	38919075	G	T	OSMR	Missense	p.R499M	9	128	7.0%	.	.	.
GC13	5	38932594	C	A	OSMR	Missense	p.P775H	8	91	8.8%	.	.	.
GC13	5	38942984	G	T	RICTOR	Missense	p.P1345Q	6	96	6.3%	.	.	.
GC13	5	38943015	G	T	RICTOR	Missense	p.H1335N	8	102	7.8%	.	.	.
GC13	5	38953194	C	A	RICTOR	Splicing	c.1936-1G>T;NM_001285439	7	84	8.3%	.	.	.
GC13	5	38953603	C	A	RICTOR	Missense	p.W917L	9	158	5.7%	.	.	.
GC13	5	38958840	G	T	RICTOR	Missense	p.Q758K	8	119	6.7%	.	.	.
GC13	5	38975670	C	A	RICTOR	Missense	p.M286I	9	125	7.2%	.	.	.

GC13	5	39383003	C	A	DAB2	Missense	p.R332L	6	99	6.1%	.	.	.
GC13	5	40728550	G	T	TTC33	Missense	p.P111Q	10	70	14.3%	.	.	.
GC13	5	40853190	C	A	CARD6	Missense	p.Q586K	9	93	9.7%	.	.	.
GC13	5	40976891	G	T	C7	Missense	p.W705L	7	84	8.3%	.	.	.
GC13	5	41009459	C	A	MROH2B	Missense	p.G1115W	9	98	9.2%	.	.	.
GC13	5	41012818	G	T	MROH2B	Missense	p.P1001Q	7	88	8.0%	.	.	.
GC13	5	41042290	C	A	MROH2B	Missense	p.L619F	7	83	8.4%	.	.	.
GC13	5	41161926	G	T	C6	Missense	p.L443M	9	105	8.6%	.	.	.
GC13	5	41862770	G	T	OXCT1	Missense	p.P54H	8	108	7.4%	.	.	.
GC13	5	43161910	C	A	ZNF131	Missense	p.H32N	7	87	8.0%	.	.	.
GC13	5	43529976	G	T	PAIP1	Missense	p.Q420K	7	94	7.4%	.	.	.
GC13	5	43543189	C	A	PAIP1	Missense	p.M217I	7	56	12.5%	.	.	.
GC13	5	43644712	G	T	NNT	Splicing	c.1099-1G>T;NM_001331026	7	69	10.1%	.	.	.
GC13	5	44813237	G	T	MRPS30	Missense	p.G295C	7	127	5.5%	.	.	.
GC13	5	52097647	G	T	PELO	Missense	p.E377D	10	114	8.8%	.	.	.
GC13	5	52177804	C	A	ITGA1	Missense	p.Q242K	6	56	10.7%	.	.	.
GC13	5	52243291	G	T	ITGA1	Missense	p.K1165N	8	98	8.2%	.	.	.
GC13	5	52368464	G	T	ITGA2	Missense	p.G790C	7	90	7.8%	.	.	.
GC13	5	52374699	C	A	ITGA2	Missense	p.L975M	8	122	6.6%	.	.	.
GC13	5	53751981	C	A	HSPB3	Missense	p.P121Q	10	152	6.6%	.	.	.
GC13	5	54320635	G	T	GZMK	Missense	p.R71L	6	85	7.1%	.	.	.
GC13	5	54423175	C	A	CDC20B	Missense	p.W300L	8	115	7.0%	.	.	.
GC13	5	54456057	G	T	GPX8	Missense	p.G13W	6	95	6.3%	.	.	.
GC13	5	54557291	G	T	DHX29	Missense	p.L1238I	9	111	8.1%	.	.	.
GC13	5	54579263	C	A	DHX29	Missense	p.R578L	8	136	5.9%	.	.	.
GC13	5	54646775	G	T	SKIV2L2	Missense	p.R450M	7	79	8.9%	.	.	.
GC13	5	54649025	G	T	SKIV2L2	Missense	p.M487I	7	85	8.2%	.	.	.
GC13	5	55238524	G	T	IL6ST	Missense	p.Q604K	5	46	10.9%	.	.	.
GC13	5	55479377	C	A	ANKRD55	Missense	p.R48L	6	75	8.0%	.	.	.
GC13	5	56170922	C	A	MAP3K1	Missense	p.L584I	7	97	7.2%	O	.	.
GC13	5	56777915	C	A	ACTBL2	Missense	p.R207L	7	91	7.7%	.	.	.

GC13	5	58270801	C	A	PDE4D	Missense	p.W483L	7	66	10.6%	.	.	.
GC13	5	58270873	C	A	PDE4D	Missense	p.W459L	6	68	8.8%	.	.	.
GC13	5	60241189	C	A	NDUFAF2	Missense	p.P36Q	6	59	10.2%	.	.	.
GC13	5	61778942	G	T	IPO11	Missense	p.L321F	7	79	8.9%	.	.	.
GC13	5	63496731	G	T	RNF180	Missense	p.G33C	7	84	8.3%	.	.	.
GC13	5	64181305	G	T	CWC27	Missense	p.R325L	10	85	11.8%	.	.	.
GC13	5	64868018	G	T	PPWD1	Missense	p.G136W	9	97	9.3%	.	.	.
GC13	5	64892886	G	T	TRIM23	Missense	p.P434Q	7	83	8.4%	.	.	.
GC13	5	65016588	C	A	SGTB	Missense	p.R16L	7	113	6.2%	.	.	.
GC13	5	65339999	C	A	ERBIN	Missense	p.P488Q	12	70	17.1%	.	.	.
GC13	5	66282291	G	T	MAST4	Missense	p.G232W	9	102	8.8%	.	.	.
GC13	5	66300888	G	T	MAST4	Missense	p.G23W	9	77	11.7%	.	.	.
GC13	5	66414523	G	T	MAST4	Missense	p.L341F	11	98	11.2%	.	.	.
GC13	5	66459085	G	T	MAST4	Missense	p.G1099W	7	81	8.6%	.	.	.
GC13	5	66459103	G	T	MAST4	Nonsense	p.G1105X	8	80	10.0%	.	.	.
GC13	5	68805231	C	A	OCLN	Missense	p.P105H	7	87	8.0%	.	.	.
GC13	5	70307105	G	T	NAIP	Missense	p.P222H	8	76	10.5%	.	.	.
GC13	5	70806137	C	A	BDP1	Missense	p.P1073Q	7	90	7.8%	.	.	.
GC13	5	70806382	C	A	BDP1	Missense	p.P1155T	8	126	6.3%	.	.	.
GC13	5	70806625	C	A	BDP1	Missense	p.Q1236K	8	130	6.2%	.	.	.
GC13	5	70952584	G	T	MCCC2	Missense	p.G530V	7	97	7.2%	.	.	.
GC13	5	71482492	G	T	MAP1B	Missense	p.G15W	5	42	11.9%	.	.	.
GC13	5	71489696	G	T	MAP1B	Missense	p.G46W	6	92	6.5%	.	.	.
GC13	5	71490665	C	A	MAP1B	Missense	p.Q369K	8	103	7.8%	.	.	.
GC13	5	71491452	C	A	MAP1B	Missense	p.P631Q	7	94	7.4%	.	.	.
GC13	5	71654043	G	T	PTCD2	Missense	p.R210M	7	79	8.9%	.	.	.
GC13	5	71740025	C	A	ZNF366	Missense	p.R598L	6	87	6.9%	.	.	.
GC13	5	72192304	G	T	TNPO1	Missense	p.L721F	6	39	15.4%	.	.	.
GC13	5	72427429	G	T	TMEM171	Missense	p.G282W	7	88	8.0%	.	.	.
GC13	5	73932027	G	T	ENC1	Missense	p.P22Q	7	96	7.3%	.	.	.
GC13	5	74021569	C	A	GFM2	Nonsense	p.G595X	7	89	7.9%	.	.	.

GC13	5	74021808	G	T	GFM2	Missense	p.Q577K	6	56	10.7%	.	.	.
GC13	5	74137491	G	T	FAM169A	Missense	p.P4H	8	117	6.8%	.	.	.
GC13	5	74675277	G	T	COL4A3BP	Missense	p.P564Q	7	99	7.1%	.	.	.
GC13	5	76717679	G	T	PDE8B	Missense	p.G665W	8	76	10.5%	.	.	.
GC13	5	76717749	C	A	PDE8B	Missense	p.P688Q	6	59	10.2%	.	.	.
GC13	5	78076386	C	A	ARSB	Missense	p.R479L	6	86	7.0%	.	.	.
GC13	5	78938808	G	T	PAPD4	Missense	p.A276S	7	89	7.9%	.	.	.
GC13	5	79026231	C	A	CMYA5	Missense	p.P548Q	10	134	7.5%	.	.	.
GC13	5	79363910	C	A	THBS4	Missense	p.Q346K	10	121	8.3%	.	.	.
GC13	5	79368163	G	T	THBS4	Missense	p.G504C	6	70	8.6%	.	.	.
GC13	5	79442021	C	A	SERINC5	Missense	p.R377L	5	40	12.5%	.	.	.
GC13	5	79473137	C	A	SERINC5	Missense	p.A153S	12	94	12.8%	.	.	.
GC13	5	79855589	C	A	ANKRD34B	Missense	p.G84W	10	101	9.9%	.	.	.
GC13	5	79855715	G	T	ANKRD34B	Missense	p.R42S	6	78	7.7%	.	.	.
GC13	5	81548396	G	T	ATG10	Missense	p.G157W	10	153	6.5%	.	.	.
GC13	5	82400809	G	T	XRCC4	Missense	p.W24L	11	96	11.5%	.	.	.
GC13	5	82816264	G	T	VCAN	Missense	p.M713I	10	95	10.5%	.	.	.
GC13	5	82816900	G	T	VCAN	Missense	p.L925F	8	101	7.9%	.	.	.
GC13	5	82816969	G	T	VCAN	Missense	p.E948D	7	80	8.8%	.	.	.
GC13	5	82832910	C	A	VCAN	Missense	p.P376Q	8	85	9.4%	.	.	.
GC13	5	82833059	G	T	VCAN	Missense	p.G426W	8	105	7.6%	.	.	.
GC13	5	82849273	G	T	VCAN	Missense	p.R454L	7	94	7.4%	.	.	.
GC13	5	82937367	G	T	HAPLN1	Missense	p.P338Q	9	100	9.0%	.	.	.
GC13	5	83362383	G	T	EDIL3	Missense	p.Q222K	7	92	7.6%	.	O	.
GC13	5	89979762	G	T	ADGRV1	Missense	p.M2008I	7	94	7.4%	.	.	.
GC13	5	89999608	C	A	ADGRV1	Missense	p.P2761H	8	129	6.2%	.	.	.
GC13	5	90012413	G	T	ADGRV1	Missense	p.R3105L	9	96	9.4%	.	.	.
GC13	5	90106476	G	T	ADGRV1	Missense	p.M5133I	6	71	8.5%	.	.	.
GC13	5	92923881	C	A	NR2F1	Missense	p.P241Q	6	86	7.0%	.	.	.
GC13	5	92929459	G	T	NR2F1	Missense	p.G395C	7	92	7.6%	.	.	.
GC13	5	92929504	G	T	NR2F1	Missense	p.G410W	9	91	9.9%	.	.	.

GC13	5	93489760	A	C	KIAA0825	Missense	p.I1260M	11	44	25.0%	.	.	.
GC13	5	94877029	G	T	TTC37	Missense	p.Q128K	8	117	6.8%	.	.	.
GC13	5	95072689	C	A	RHOBTB3	Missense	p.H109N	7	74	9.5%	.	.	.
GC13	5	95103799	G	T	RHOBTB3	Missense	p.W404L	8	141	5.7%	.	.	.
GC13	5	95119593	C	A	RHOBTB3	Missense	p.Q517K	9	113	8.0%	.	.	.
GC13	5	96101812	C	A	CAST	Missense	p.P561Q	7	82	8.5%	.	.	.
GC13	5	96121660	G	T	ERAP1	Missense	p.P592Q	8	102	7.8%	.	.	.
GC13	5	96460270	G	T	LIX1	Missense	p.P49Q	6	75	8.0%	.	.	.
GC13	5	101726750	C	A	SLCO6A1	Splicing	c.1815-1G>T;NM_173488	8	121	6.6%	.	.	.
GC13	5	102309859	G	T	PAM	Missense	p.R401M	9	135	6.7%	.	O	.
GC13	5	102474159	C	A	PPIP5K2	Missense	p.P158Q	7	100	7.0%	.	.	.
GC13	5	108294950	G	T	FER	Missense	p.G151W	10	111	9.0%	.	.	.
GC13	5	108380380	G	T	FER	Splicing	c.1189-1G>T;NM_001308031	8	95	8.4%	.	.	.
GC13	5	108714713	C	A	PJA2	Nonsense	p.G159X	8	107	7.5%	.	.	.
GC13	5	109124749	G	T	MAN2A1	Missense	p.M625I	9	113	8.0%	.	.	.
GC13	5	109961033	G	T	TMEM232	Missense	p.R235S	7	113	6.2%	.	.	.
GC13	5	110462495	C	A	WDR36	Missense	p.Q924K	9	116	7.8%	.	.	.
GC13	5	111481676	G	T	EPB41L4A	Missense	p.P651H	7	94	7.4%	.	.	.
GC13	5	111541164	G	T	EPB41L4A	Missense	p.Q406K	9	83	10.8%	.	.	.
GC13	5	111595639	G	T	EPB41L4A	Missense	p.P227Q	6	84	7.1%	.	.	.
GC13	5	112176494	G	T	APC	Missense	p.G1717W	8	94	8.5%	O	.	.
GC13	5	112176774	C	A	APC	Missense	p.P1810Q	8	128	6.3%	O	.	.
GC13	5	112770223	C	A	TSSK1B	Missense	p.R105L	7	60	11.7%	.	.	.
GC13	5	114607026	G	T	CCDC112	Missense	p.Q406K	8	126	6.3%	.	.	.
GC13	5	115361784	G	T	LVRN	Missense	p.R981M	8	111	7.2%	.	.	.
GC13	5	115814333	C	A	SEMA6A	Missense	p.K444N	8	109	7.3%	.	.	.
GC13	5	118440942	G	T	DMXL1	Missense	p.W118L	7	76	9.2%	.	.	.
GC13	5	118469591	C	A	DMXL1	Missense	p.H658N	8	130	6.2%	.	.	.
GC13	5	118484736	C	A	DMXL1	Missense	p.Q1072K	9	122	7.4%	.	.	.
GC13	5	118485342	G	T	DMXL1	Missense	p.G1274W	6	70	8.6%	.	.	.

GC13	5	118865649	G	T	HSD17B4	Missense	p.G592C	7	112	6.3%	.	.	.
GC13	5	118872153	C	A	HSD17B4	Missense	p.Q659K	6	80	7.5%	.	.	.
GC13	5	121188017	G	T	FTMT	Missense	p.R120L	8	94	8.5%	.	.	.
GC13	5	121362742	G	T	SRFBP1	Missense	p.W404L	10	123	8.1%	.	.	.
GC13	5	121413188	C	A	LOX	Missense	p.G165C	6	81	7.4%	.	.	.
GC13	5	121767743	C	A	SNCAIP	Missense	p.P55Q	7	90	7.8%	.	.	.
GC13	5	123983692	C	A	ZNF608	Missense	p.M795I	9	89	10.1%	.	.	.
GC13	5	125918651	C	A	ALDH7A1	Missense	p.G137W	8	131	6.1%	.	.	.
GC13	5	126887531	C	A	PRRC1	Missense	p.R421S	7	71	9.9%	.	.	.
GC13	5	126993451	C	A	CTXN3	Missense	p.L80I	8	53	15.1%	.	.	.
GC13	5	127448549	C	A	SLC12A2	Missense	p.P267Q	10	97	10.3%	.	.	.
GC13	5	127488407	G	T	SLC12A2	Missense	p.W758L	7	71	9.9%	.	.	.
GC13	5	127623023	C	A	FBN2	Missense	p.R2286M	8	111	7.2%	.	.	.
GC13	5	127671228	G	T	FBN2	Missense	p.Q1256K	9	130	6.9%	.	.	.
GC13	5	127782275	G	T	FBN2	Missense	p.P284Q	7	96	7.3%	.	O	.
GC13	5	129030536	C	A	ADAMTS19	Missense	p.P981Q	6	74	8.1%	.	.	.
GC13	5	130522775	G	T	LYRM7	Missense	p.G73C	8	104	7.7%	.	.	.
GC13	5	130766596	G	T	RAPGEF6	Missense	p.P1474Q	7	91	7.7%	.	.	.
GC13	5	130840411	G	T	RAPGEF6	Missense	p.H388N	8	113	7.1%	.	.	.
GC13	5	131066698	C	A	FNIP1	Missense	p.G85W	7	92	7.6%	.	.	.
GC13	5	131539514	G	T	P4HA2	Missense	p.P393H	8	92	8.7%	.	.	.
GC13	5	132052632	G	T	KIF3A	Missense	p.H221N	9	99	9.1%	.	.	.
GC13	5	132159936	C	A	SHROOM1	Missense	p.G473W	5	37	13.5%	.	.	.
GC13	5	132232832	G	T	AFF4	Missense	p.P497Q	7	90	7.8%	.	.	.
GC13	5	133478765	C	A	TCF7	Missense	p.P219T	7	74	9.5%	.	.	.
GC13	5	134223540	C	A	TXNDC15	Missense	p.Q19K	7	57	12.3%	.	.	.
GC13	5	134678979	G	T	H2AFY	Missense	p.M87I	8	92	8.7%	.	.	.
GC13	5	134679061	C	A	H2AFY	Missense	p.Q115K	8	135	5.9%	.	.	.
GC13	5	135229710	G	T	IL9	Missense	p.P105Q	5	25	20.0%	.	.	.
GC13	5	137089614	G	T	HNRNPA0	Missense	p.R48S	9	161	5.6%	.	.	.
GC13	5	137222941	G	T	MYOT	Missense	p.R271L	11	214	5.1%	.	.	.

GC13	5	137222979	G	T	MYOT	Missense	p.G284W	14	212	6.6%	.	.	.
GC13	5	137235246	G	T	PKD2L2	Missense	p.W189L	11	204	5.4%	.	.	.
GC13	5	137244511	C	A	PKD2L2	Missense	p.R380S	9	172	5.2%	.	.	.
GC13	5	137257394	G	T	PKD2L2	Missense	p.L444F	11	210	5.2%	.	.	.
GC13	5	137276030	G	T	FAM13B	Missense	p.Q850K	11	256	4.3%	.	.	.
GC13	5	137295904	C	A	FAM13B	Splicing	c.891-1G>T;NM_001101800	10	200	5.0%	.	.	.
GC13	5	137419934	G	T	WNT8A	Missense	p.G40C	13	223	5.8%	.	.	.
GC13	5	137488277	G	T	BRD8	Missense	p.P917Q	11	165	6.7%	.	.	.
GC13	5	137520800	C	A	KIF20A	Missense	p.Q614K	13	202	6.4%	.	.	.
GC13	5	137522962	G	T	KIF20A	Missense	p.G845W	13	165	7.9%	.	.	.
GC13	5	137622886	G	T	CDC25C	Missense	p.P260Q	10	202	5.0%	.	.	.
GC13	5	137681021	G	T	FAM53C	Missense	p.W215L	10	179	5.6%	.	.	.
GC13	5	137713411	G	T	KDM3B	Missense	p.M159I	9	172	5.2%	.	.	.
GC13	5	137721820	C	A	KDM3B	Missense	p.P297H	12	162	7.4%	.	.	.
GC13	5	137727481	G	T	KDM3B	Missense	p.M720I	10	164	6.1%	.	.	.
GC13	5	137728982	C	T	KDM3B	Missense	p.R918C	12	116	10.3%	.	.	.
GC13	5	137759878	C	A	KDM3B	Missense	p.Q1363K	8	128	6.3%	.	.	.
GC13	5	137766019	C	A	KDM3B	Missense	p.R1659S	7	147	4.8%	.	.	.
GC13	5	137848611	G	T	ETF1	Missense	p.R178S	11	214	5.1%	.	.	.
GC13	5	137878526	G	T	ETF1	Missense	p.R28S	5	70	7.1%	.	.	.
GC13	5	138643245	G	T	MATR3	Missense	p.Q47H	9	144	6.3%	.	.	.
GC13	5	138643965	G	T	MATR3	Missense	p.K287N	9	143	6.3%	.	.	.
GC13	5	138700356	C	A	PAIP2	Missense	p.P81Q	8	125	6.4%	.	.	.
GC13	5	138707847	C	A	SLC23A1	Missense	p.G549W	5	43	11.6%	.	.	.
GC13	5	138728921	G	T	PROB1	Missense	p.P617Q	6	80	7.5%	.	.	.
GC13	5	138729153	C	A	PROB1	Missense	p.G540W	8	105	7.6%	.	.	.
GC13	5	139060650	C	A	CXXC5	Missense	p.P181Q	6	66	9.1%	.	O	.
GC13	5	139192907	G	T	PSD2	Missense	p.G129W	4	25	16.0%	.	.	.
GC13	5	139231361	G	T	NRG2	Missense	p.Q468K	7	85	8.2%	.	.	.
GC13	5	139266962	G	T	NRG2	Missense	p.R279S	8	116	6.9%	.	.	.
GC13	5	139722298	G	T	HBEGF	Missense	p.P107Q	11	117	9.4%	.	.	.

GC13	5	139742076	C	A	SLC4A9	Missense	p.H256N	5	36	13.9%	.	.	.
GC13	5	139745472	G	T	SLC4A9	Missense	p.Q574H	8	115	7.0%	.	.	.
GC13	5	139751046	G	T	SLC4A9	Missense	p.R773M	8	121	6.6%	.	.	.
GC13	5	139876525	C	A	ANKHD1	Missense	p.P889Q	10	99	10.1%	.	.	.
GC13	5	139907769	G	T	ANKHD1	Missense	p.R1746S	9	82	11.0%	.	.	.
GC13	5	139908520	G	T	ANKHD1	Missense	p.G1997W	10	165	6.1%	.	.	.
GC13	5	139918960	G	T	ANKHD1	Missense	p.W2529L	12	150	8.0%	.	.	.
GC13	5	139921791	G	T	ANKHD1-EIF4EBP3	Missense	p.G2535V	12	143	8.4%	.	.	.
GC13	5	140033599	C	A	IK	Missense	p.H161N	9	106	8.5%	.	.	.
GC13	5	140039391	G	T	IK	Missense	p.G416W	5	41	12.2%	.	.	.
GC13	5	140052858	G	T	DND1	Missense	p.P47Q	6	64	9.4%	.	.	.
GC13	5	140056672	G	T	HARS	Missense	p.Q171K	8	112	7.1%	.	.	.
GC13	5	140073622	C	A	HARS2	Missense	p.P71T	8	113	7.1%	.	.	.
GC13	5	140182644	C	A	PCDHA3	Missense	p.P621Q	7	86	8.1%	.	.	.
GC13	5	140202075	C	A	PCDHA5	Missense	p.P239T	10	154	6.5%	.	.	.
GC13	5	140203639	G	T	PCDHA5	Missense	p.G760V	7	95	7.4%	.	.	.
GC13	5	140207690	C	A	PCDHA6	Missense	p.P5Q	6	71	8.5%	.	.	.
GC13	5	140208823	G	T	PCDHA6	Missense	p.G383W	8	91	8.8%	.	.	.
GC13	5	140223071	G	T	PCDHA8	Missense	p.R722L	7	68	10.3%	.	.	.
GC13	5	140237479	G	T	PCDHA10	Missense	p.G616C	6	66	9.1%	.	.	.
GC13	5	140249077	C	A	PCDHA11	Missense	p.P130Q	7	91	7.7%	.	.	.
GC13	5	140249153	G	T	PCDHA11	Missense	p.E155D	9	118	7.6%	.	.	.
GC13	5	140250547	C	A	PCDHA11	Missense	p.P620Q	7	91	7.7%	.	.	.
GC13	5	140255696	G	T	PCDHA12	Missense	p.M213I	10	140	7.1%	.	.	.
GC13	5	140307114	C	A	PCDHAC1	Missense	p.R213S	6	81	7.4%	.	.	.
GC13	5	140308489	G	T	PCDHAC1	Missense	p.W671L	10	114	8.8%	.	.	.
GC13	5	140348194	G	T	PCDHAC2	Missense	p.G615W	8	120	6.7%	.	.	.
GC13	5	140432829	G	C	PCDHB1	Missense	p.A592P	11	109	10.1%	.	.	.
GC13	5	140432895	G	T	PCDHB1	Missense	p.G614W	8	119	6.7%	.	.	.
GC13	5	140475317	C	A	PCDHB2	Missense	p.Q315K	10	102	9.8%	.	.	.
GC13	5	140475470	C	A	PCDHB2	Missense	p.L366I	9	122	7.4%	.	.	.

GC13	5	140530382	C	A	PCDHB6	Missense	p.R182S	7	113	6.2%	.	.	.
GC13	5	140530410	C	A	PCDHB6	Missense	p.P191Q	7	100	7.0%	.	.	.
GC13	5	140530658	G	T	PCDHB6	Missense	p.G274W	11	156	7.1%	.	.	.
GC13	5	140552862	G	T	PCDHB7	Missense	p.G149V	9	125	7.2%	.	.	.
GC13	5	140558810	G	T	PCDHB8	Missense	p.G399W	10	172	5.8%	.	.	.
GC13	5	140562339	C	A	PCDHB16	Missense	p.Q69K	7	68	10.3%	.	.	.
GC13	5	140562739	G	T	PCDHB16	Missense	p.R202L	8	111	7.2%	.	.	.
GC13	5	140563269	G	T	PCDHB16	Missense	p.G379W	8	119	6.7%	.	O	.
GC13	5	140579718	G	T	PCDHB11	Missense	p.R124M	11	174	6.3%	.	.	.
GC13	5	140580194	C	A	PCDHB11	Missense	p.H283N	14	166	8.4%	.	O	.
GC13	5	140580625	G	T	PCDHB11	Missense	p.L426F	7	83	8.4%	.	.	.
GC13	5	140589326	C	A	PCDHB12	Missense	p.H283N	9	147	6.1%	.	O	.
GC13	5	140589636	C	A	PCDHB12	Missense	p.P386Q	11	125	8.8%	.	.	.
GC13	5	140625591	G	T	PCDHB15	Missense	p.G149W	9	131	6.9%	.	.	.
GC13	5	140682747	G	T	SLC25A2	Missense	p.P229Q	10	98	10.2%	.	.	.
GC13	5	140698953	C	A	TAF7	Missense	p.R220L	9	161	5.6%	.	.	.
GC13	5	140719059	C	A	PCDHGA2	Missense	p.P174Q	10	129	7.8%	.	.	.
GC13	5	140725212	G	T	PCDHGA3	Missense	p.G538W	8	144	5.6%	.	.	.
GC13	5	140732027	G	T	PCDHGB1	Missense	p.G734W	8	105	7.6%	.	.	.
GC13	5	140735003	C	A	PCDHGA4	Missense	p.P110Q	6	65	9.2%	.	.	.
GC13	5	140741915	G	T	PCDHGB2	Missense	p.G738V	9	126	7.1%	.	O	.
GC13	5	140744720	G	T	PCDHGA5	Missense	p.G275W	11	126	8.7%	.	.	.
GC13	5	140745186	C	A	PCDHGA5	Missense	p.P430Q	8	115	7.0%	.	.	.
GC13	5	140752342	C	A	PCDHGB3	Missense	p.P794Q	8	109	7.3%	.	.	.
GC13	5	140754885	G	T	PCDHGA6	Missense	p.R412L	9	114	7.9%	.	O	.
GC13	5	140763553	G	T	PCDHGA7	Missense	p.G363W	8	100	8.0%	.	.	.
GC13	5	140763701	G	T	PCDHGA7	Missense	p.R412L	6	90	6.7%	.	.	.
GC13	5	140763745	G	T	PCDHGA7	Missense	p.G427C	8	98	8.2%	.	.	.
GC13	5	140764085	C	A	PCDHGA7	Missense	p.P540Q	6	95	6.3%	.	.	.
GC13	5	140769843	C	A	PCDHGB4	Missense	p.H798N	12	158	7.6%	.	.	.
GC13	5	140778449	G	T	PCDHGB5	Missense	p.R252L	9	141	6.4%	.	.	.

GC13	5	140788374	G	T	PCDHGB6	Missense	p.R202L	7	118	5.9%	.	.	.
GC13	5	140788884	G	T	PCDHGB6	Missense	p.R372L	9	156	5.8%	.	.	.
GC13	5	140811292	G	T	PCDHGA12	Missense	p.M322I	10	122	8.2%	.	.	.
GC13	5	140811755	C	A	PCDHGA12	Missense	p.H477N	9	140	6.4%	.	.	.
GC13	5	140856933	G	T	PCDHGC3	Missense	p.R417L	11	141	7.8%	.	.	.
GC13	5	140857734	G	T	PCDHGC3	Missense	p.R684L	7	104	6.7%	.	.	.
GC13	5	140865563	G	T	PCDHGC4	Missense	p.G275C	8	91	8.8%	.	.	.
GC13	5	140865819	C	A	PCDHGC4	Missense	p.P360H	8	109	7.3%	.	.	.
GC13	5	140866031	G	T	PCDHGC4	Missense	p.G431W	8	120	6.7%	.	.	.
GC13	5	140870257	G	T	PCDHGC5	Missense	p.G484W	9	98	9.2%	.	.	.
GC13	5	140913911	G	T	DIAPH1	Missense	p.Q849K	8	117	6.8%	.	.	.
GC13	5	140953478	C	A	DIAPH1	Missense	p.G638W	6	41	14.6%	.	.	.
GC13	5	140954548	G	T	DIAPH1	Missense	p.Q534K	5	42	11.9%	.	.	.
GC13	5	141020000	G	T	RELL2	Missense	p.G301W	9	92	9.8%	.	.	.
GC13	5	141024191	G	T	FCHSD1	Missense	p.L531I	8	94	8.5%	.	.	.
GC13	5	141035827	C	A	ARAP3	Missense	p.R1269L	9	107	8.4%	.	.	.
GC13	5	141059944	C	A	ARAP3	Missense	p.R37L	6	98	6.1%	.	.	.
GC13	5	141303520	C	A	KIAA0141	Missense	p.P5Q	6	53	11.3%	.	.	.
GC13	5	141331146	G	T	PCDH12	Missense	p.Q964K	8	111	7.2%	.	.	.
GC13	5	141511803	G	T	NDFIP1	Missense	p.G60W	8	114	7.0%	.	O	.
GC13	5	141515314	G	T	NDFIP1	Missense	p.R101L	5	59	8.5%	.	.	.
GC13	5	143545089	C	A	YIPF5	Missense	p.G10W	6	70	8.6%	.	.	.
GC13	5	145393454	G	T	SH3RF2	Missense	p.G297W	8	116	6.9%	.	.	.
GC13	5	145502084	C	A	LARS	Missense	p.G1009W	9	132	6.8%	.	.	.
GC13	5	145524042	G	T	LARS	Missense	p.L504M	10	194	5.2%	.	.	.
GC13	5	145537049	G	T	LARS	Missense	p.Q282K	10	177	5.6%	.	.	.
GC13	5	145537303	G	T	LARS	Missense	p.P230Q	8	131	6.1%	.	.	.
GC13	5	145539016	G	T	LARS	Missense	p.P196Q	8	112	7.1%	.	.	.
GC13	5	145613126	G	T	RBM27	Missense	p.G322W	12	132	9.1%	.	.	.
GC13	5	145859424	C	A	TCERG1	Missense	p.P569Q	11	117	9.4%	.	.	.
GC13	5	145894467	G	T	GPR151	Missense	p.P404T	9	117	7.7%	.	.	.

GC13	5	146257613	G	T	PPP2R2B	Missense	p.R74S	7	106	6.6%	.	.	.
GC13	5	146619200	G	T	STK32A	Missense	p.M1I	8	123	6.5%	.	.	.
GC13	5	146722568	G	T	STK32A	Splicing	c.435-1G>T;NM_001112724	13	177	7.3%	.	.	.
GC13	5	146780395	C	A	DPYSL3	Missense	p.G438W	7	98	7.1%	.	.	.
GC13	5	147003213	G	T	JAKMIP2	Missense	p.Q658K	8	113	7.1%	.	.	.
GC13	5	147513385	C	A	SPINK5	Missense	p.R1041S	6	100	6.0%	.	.	.
GC13	5	147795533	G	T	FBXO38	Missense	p.R436L	7	133	5.3%	.	.	.
GC13	5	147806820	C	A	FBXO38	Missense	p.H655N	10	150	6.7%	.	.	.
GC13	5	148207332	G	T	ADRB2	Missense	p.W313L	8	138	5.8%	.	.	.
GC13	5	148207539	C	A	ADRB2	Missense	p.P382Q	12	126	9.5%	.	.	.
GC13	5	148424184	C	A	SH3TC2	Missense	p.L99F	7	86	8.1%	.	.	.
GC13	5	148618838	C	A	ABLIM3	Missense	p.Q358K	8	105	7.6%	.	.	.
GC13	5	148997775	G	T	ARHGEF37	Missense	p.R232L	9	132	6.8%	.	.	.
GC13	5	149212325	G	T	PPARGC1B	Missense	p.R191L	7	114	6.1%	.	.	.
GC13	5	149212497	G	T	PPARGC1B	Missense	p.M248I	7	84	8.3%	.	.	.
GC13	5	149357813	G	T	SLC26A2	Missense	p.G200W	10	93	10.8%	.	.	.
GC13	5	149360255	G	T	SLC26A2	Missense	p.G367W	9	129	7.0%	.	.	.
GC13	5	149361236	C	A	SLC26A2	Missense	p.L694I	8	135	5.9%	.	O	.
GC13	5	149429957	C	A	HMGXB3	Missense	p.P1115Q	9	66	13.6%	.	.	.
GC13	5	149500479	C	A	PDGFRB	Missense	p.R853L	8	110	7.3%	.	.	.
GC13	5	149602628	G	T	CAMK2A	Missense	p.R453S	5	69	7.2%	.	.	.
GC13	5	149631371	G	T	CAMK2A	Missense	p.P212Q	4	32	12.5%	.	.	.
GC13	5	149631598	G	T	CAMK2A	Missense	p.P182Q	8	90	8.9%	.	.	.
GC13	5	149677066	C	A	ARSI	Missense	p.R474L	6	97	6.2%	.	.	.
GC13	5	149754913	G	T	TCOF1	Missense	p.L423F	9	119	7.6%	.	.	.
GC13	5	149754914	G	T	TCOF1	Missense	p.G424W	12	117	10.3%	.	O	.
GC13	5	149755296	G	T	TCOF1	Missense	p.G496W	10	113	8.8%	.	.	.
GC13	5	149758537	G	T	TCOF1	Missense	p.G727W	9	92	9.8%	.	.	.
GC13	5	149758816	C	A	TCOF1	Missense	p.Q758K	8	116	6.9%	.	.	.
GC13	5	149776294	C	A	TCOF1	Missense	p.Q1334K	8	103	7.8%	.	.	.
GC13	5	149901271	G	T	NDST1	Missense	p.R152L	7	102	6.9%	.	.	.

GC13	5	150029416	G	T	SYNPO	Missense	p.G527W	7	111	6.3%	.	.	.
GC13	5	150029792	G	T	SYNPO	Missense	p.R652M	8	126	6.3%	.	.	.
GC13	5	150052001	G	T	MYOZ3	Missense	p.R175L	7	120	5.8%	.	.	.
GC13	5	150228096	G	T	IRGM	Missense	p.M137I	8	120	6.7%	.	.	.
GC13	5	150275419	C	A	ZNF300	Missense	p.G425V	8	108	7.4%	.	.	.
GC13	5	150509045	G	T	ANXA6	Missense	p.R249S	8	73	11.0%	.	.	.
GC13	5	150512686	C	A	ANXA6	Missense	p.L171F	8	116	6.9%	.	O	.
GC13	5	150923471	G	T	FAT2	Missense	p.P2406H	8	82	9.8%	.	.	.
GC13	5	150925004	C	A	FAT2	Missense	p.R1895L	10	128	7.8%	.	.	.
GC13	5	150925354	C	A	FAT2	Missense	p.M1778I	10	171	5.8%	.	.	.
GC13	5	150931165	C	A	FAT2	Missense	p.G1387W	9	131	6.9%	.	.	.
GC13	5	150932746	T	C	FAT2	Missense	p.N1383S	12	69	17.4%	.	.	.
GC13	5	150945471	G	T	FAT2	Missense	p.L1008I	9	87	10.3%	.	.	.
GC13	5	150947892	C	A	FAT2	Missense	p.G201C	7	126	5.6%	.	.	.
GC13	5	151202326	G	T	GLRA1	Missense	p.R337S	10	147	6.8%	.	O	.
GC13	5	151304076	C	A	GLRA1	Missense	p.W12L	8	104	7.7%	.	.	.
GC13	5	153377365	C	A	FAM114A2	Missense	p.G373W	9	168	5.4%	.	.	.
GC13	5	153406753	G	T	FAM114A2	Missense	p.Q188K	13	158	8.2%	.	.	.
GC13	5	154173283	G	T	LARP1	Missense	p.G213W	10	116	8.6%	.	.	.
GC13	5	154275813	G	T	GEMIN5	Missense	p.H1145N	7	77	9.1%	.	.	.
GC13	5	154311712	C	A	GEMIN5	Missense	p.W203L	8	95	8.4%	.	.	.
GC13	5	154316631	C	A	GEMIN5	Missense	p.W94L	8	127	6.3%	.	.	.
GC13	5	154393528	G	T	KIF4B	Missense	p.G37W	7	99	7.1%	.	.	.
GC13	5	154394824	C	A	KIF4B	Missense	p.L469M	8	112	7.1%	.	.	.
GC13	5	154395782	G	T	KIF4B	Missense	p.R788L	7	105	6.7%	.	.	.
GC13	5	154395791	C	A	KIF4B	Missense	p.P791Q	8	105	7.6%	.	.	.
GC13	5	156074522	G	T	SGCD	Missense	p.R183M	8	94	8.5%	.	.	.
GC13	5	156347515	C	A	TIMD4	Splicing	c.1013-1G>T	8	111	7.2%	.	.	.
GC13	5	156376662	C	A	TIMD4	Nonsense	p.E254X	7	68	10.3%	.	.	.
GC13	5	156482389	G	T	HAVCR1	Missense	p.H68N	8	129	6.2%	.	.	.
GC13	5	156533749	G	T	HAVCR2	Missense	p.L95M	9	130	6.9%	.	.	.

GC13	5	156589916	G	T	FAM71B	Missense	p.R454S	8	136	5.9%	.	.	.
GC13	5	156593133	G	T	FAM71B	Missense	p.S16Y	20	125	16.0%	.	.	.
GC13	5	156672790	G	T	ITK	Missense	p.G502W	9	111	8.1%	.	.	.
GC13	5	156760340	G	T	CYFIP2	Missense	p.L731F	7	99	7.1%	.	.	.
GC13	5	157053499	C	A	SOX30	Missense	p.G704V	9	161	5.6%	.	.	.
GC13	5	158139214	C	A	EBF1	Missense	p.L468F	7	82	8.5%	.	.	.
GC13	5	158140045	C	A	EBF1	Missense	p.M403I	7	85	8.2%	.	.	.
GC13	5	158753720	C	A	IL12B	Missense	p.W24L	6	61	9.8%	.	.	.
GC13	5	159343963	G	T	ADRA1B	Missense	p.W17C	9	83	10.8%	.	.	.
GC13	5	159344072	G	A	ADRA1B	Missense	p.A54T	20	85	23.5%	.	.	.
GC13	5	159520540	C	A	PWWP2A	Missense	p.G373W	9	160	5.6%	.	.	.
GC13	5	159776711	G	T	C1QTNF2	Missense	p.R153S	6	87	6.9%	.	.	.
GC13	5	159820940	G	T	ZBED8	Missense	p.Q520K	8	134	6.0%	.	.	.
GC13	5	159821120	C	A	ZBED8	Nonsense	p.G460X	9	116	7.8%	.	.	.
GC13	5	159849368	C	A	PTTG1	Missense	p.R17S	9	145	6.2%	.	.	.
GC13	5	160114906	C	A	ATP10B	Missense	p.W59L	9	93	9.7%	.	O	.
GC13	5	160758089	C	A	GABRB2	Missense	p.R293L	11	93	11.8%	.	.	.
GC13	5	161119029	G	T	GABRA6	Missense	p.M303I	8	135	5.9%	.	.	.
GC13	5	161292794	G	T	GABRA1	Missense	p.M85I	8	105	7.6%	.	.	.
GC13	5	162883987	C	A	NUDCD2	Missense	p.W63L	10	170	5.9%	.	.	.
GC13	5	167489211	G	T	TENM2	Missense	p.G254W	8	64	12.5%	.	.	.
GC13	5	167673980	G	T	TENM2	Missense	p.L1773F	8	109	7.3%	.	.	.
GC13	5	167674632	G	T	TENM2	Missense	p.G1991W	6	87	6.9%	.	.	.
GC13	5	167868727	G	T	WWC1	Missense	p.G774V	8	84	9.5%	.	.	.
GC13	5	167920966	C	A	RARS	Missense	p.P146Q	11	152	7.2%	.	.	.
GC13	5	167927719	C	A	RARS	Missense	p.R316S	8	95	8.4%	.	.	.
GC13	5	167984596	C	A	PANK3	Missense	p.G365W	8	132	6.1%	.	.	.
GC13	5	169230068	G	T	DOCK2	Missense	p.R854L	10	117	8.5%	.	.	.
GC13	5	169310388	G	T	FAM196B	Missense	p.P172Q	9	127	7.1%	.	.	.
GC13	5	169310518	G	T	FAM196B	Missense	p.Q129K	10	119	8.4%	.	.	.
GC13	5	169477281	G	T	DOCK2	Missense	p.G1365W	9	112	8.0%	.	O	.

GC13	5	169483741	G	T	DOCK2	Missense	p.R1450M	8	103	7.8%	.	.	.
GC13	5	169494643	G	T	DOCK2	Missense	p.G1533W	7	69	10.1%	.	.	.
GC13	5	169679440	G	T	LCP2	Missense	p.Q441K	8	115	7.0%	.	.	.
GC13	5	170236695	G	T	GABRP	Missense	p.G319V	8	83	9.6%	.	.	.
GC13	5	171305129	C	A	FBXW11	Missense	p.W231L	9	97	9.3%	.	.	.
GC13	5	171517362	G	T	STK10	Missense	p.P520Q	7	144	4.9%	.	.	.
GC13	5	171766050	G	T	SH3PXD2B	Missense	p.Q687K	11	201	5.5%	.	.	.
GC13	5	171766059	C	A	SH3PXD2B	Nonsense	p.E684X	14	201	7.0%	.	.	.
GC13	5	171766541	G	T	SH3PXD2B	Missense	p.P523H	12	185	6.5%	.	.	.
GC13	5	171780985	G	T	SH3PXD2B	Missense	p.P231Q	13	181	7.2%	.	.	.
GC13	5	171800808	C	A	SH3PXD2B	Missense	p.G138V	8	149	5.4%	.	.	.
GC13	5	172196045	C	A	DUSP1	Missense	p.R275M	11	178	6.2%	.	.	.
GC13	5	172196626	G	T	DUSP1	Missense	p.H229N	12	275	4.4%	.	.	.
GC13	5	172395594	C	A	RPL26L1	Missense	p.P101T	13	255	5.1%	.	.	.
GC13	5	172517702	G	T	CREBRF	Missense	p.G174C	18	289	6.2%	.	.	.
GC13	5	172517913	C	A	CREBRF	Missense	p.P244Q	13	275	4.7%	.	.	.
GC13	5	172571561	C	A	BNIP1	Missense	p.Q5K	11	153	7.2%	.	.	.
GC13	5	172590827	G	T	BNIP1	Missense	p.R163L	13	250	5.2%	.	.	.
GC13	5	172660468	C	A	NKX2-5	Splicing	c.335-1G>T	9	99	9.1%	.	.	.
GC13	5	172750280	G	T	STC2	Missense	p.Q150K	10	194	5.2%	.	.	.
GC13	5	173316905	G	T	CPEB4	Missense	p.G57W	11	187	5.9%	.	.	.
GC13	5	173317415	G	T	CPEB4	Missense	p.G227W	14	290	4.8%	.	.	.
GC13	5	173317673	C	A	CPEB4	Missense	p.Q313K	11	172	6.4%	.	.	.
GC13	5	173380175	C	A	CPEB4	Missense	p.P596Q	10	179	5.6%	.	.	.
GC13	5	173380237	C	A	CPEB4	Missense	p.R617S	10	195	5.1%	.	.	.
GC13	5	173382970	C	A	CPEB4	Missense	p.R649S	10	225	4.4%	.	.	.
GC13	5	174869336	G	T	DRD1	Missense	p.P256Q	12	223	5.4%	.	.	.
GC13	5	174943645	G	T	SFXN1	Nonsense	p.G209X	13	256	5.1%	.	.	.
GC13	5	174948932	G	T	SFXN1	Missense	p.M219I	12	219	5.5%	.	.	.
GC13	5	175110956	G	T	HRH2	Missense	p.M240I	12	182	6.6%	.	.	.
GC13	5	175305773	G	T	CPLX2	Missense	p.G11W	10	150	6.7%	.	.	.

GC13	5	175764090	G	T	SIMC1	Missense	p.W227L	13	286	4.5%	.	.	.
GC13	5	175782708	C	A	KIAA1191	Missense	p.G6W	17	162	10.5%	.	O	.
GC13	5	175812277	C	A	NOP16	Missense	p.G90W	13	192	6.8%	.	.	.
GC13	5	175815340	G	T	NOP16	Missense	p.H33N	11	195	5.6%	.	.	.
GC13	5	175816383	G	T	HIGD2A	Missense	p.R69L	6	78	7.7%	.	.	.
GC13	5	175819780	G	T	CLTB	Missense	p.R211S	10	202	5.0%	.	.	.
GC13	5	175825009	G	T	CLTB	Missense	p.Q92K	10	168	6.0%	.	.	.
GC13	5	175843297	G	T	CLTB	Missense	p.P23Q	8	184	4.3%	.	.	.
GC13	5	175843318	G	T	CLTB	Missense	p.P16Q	9	180	5.0%	.	.	.
GC13	5	175933775	C	A	FAF2	Missense	p.H388N	12	280	4.3%	.	.	.
GC13	5	175956042	C	A	RNF44	Missense	p.R429M	10	150	6.7%	.	.	.
GC13	5	176016406	G	T	CDHR2	Missense	p.L1028F	13	146	8.9%	.	.	.
GC13	5	176017089	C	A	CDHR2	Missense	p.Q1073K	11	216	5.1%	.	.	.
GC13	5	176024908	G	T	GPRIN1	Missense	p.P643H	11	200	5.5%	.	.	.
GC13	5	176025370	G	T	GPRIN1	Missense	p.P489H	12	231	5.2%	.	.	.
GC13	5	176026075	G	T	GPRIN1	Missense	p.P254Q	20	220	9.1%	.	.	.
GC13	5	176053464	C	A	SNCB	Missense	p.G59W	8	132	6.1%	.	.	.
GC13	5	176079789	G	T	TSPAN17	Missense	p.G111W	11	193	5.7%	.	.	.
GC13	5	176301348	G	T	UNC5A	Missense	p.G387W	9	175	5.1%	.	.	.
GC13	5	176301540	G	T	UNC5A	Missense	p.G451W	10	168	6.0%	.	.	.
GC13	5	176308306	G	T	HK3	Missense	p.P875Q	12	231	5.2%	.	.	.
GC13	5	176314052	G	T	HK3	Missense	p.P603Q	10	144	6.9%	.	.	.
GC13	5	176315759	G	T	HK3	Missense	p.L341M	10	158	6.3%	.	.	.
GC13	5	176317848	G	T	HK3	Missense	p.P170H	10	160	6.3%	.	O	.
GC13	5	176318067	G	T	HK3	Missense	p.Q129K	9	131	6.9%	.	.	.
GC13	5	176318401	G	T	HK3	Missense	p.P83T	10	195	5.1%	.	.	.
GC13	5	176338346	C	A	UIMC1	Missense	p.G548W	12	203	5.9%	.	.	.
GC13	5	176385149	G	T	UIMC1	Missense	p.Q403K	17	195	8.7%	.	.	.
GC13	5	176471427	C	A	ZNF346	Missense	p.P105H	9	148	6.1%	.	.	.
GC13	5	176524573	G	T	FGFR4	Missense	p.G729W	13	248	5.2%	O	.	.
GC13	5	176618961	C	A	NSD1	Missense	p.P335Q	13	206	6.3%	.	.	.

GC13	5	176618991	C	A	NSD1	Missense	p.P345Q	9	200	4.5%	.	.	.
GC13	5	176638425	G	T	NSD1	Missense	p.G1009C	12	287	4.2%	.	.	.
GC13	5	176638533	C	A	NSD1	Missense	p.R1045S	13	314	4.1%	.	.	.
GC13	5	176638893	C	A	NSD1	Missense	p.Q1165K	11	221	5.0%	.	.	.
GC13	5	176665247	C	A	NSD1	Missense	p.R1311S	13	283	4.6%	.	.	.
GC13	5	176687055	G	T	NSD1	Missense	p.G1678W	15	245	6.1%	.	.	.
GC13	5	176707683	C	A	NSD1	Missense	p.R1914S	10	224	4.5%	.	.	.
GC13	5	176707798	G	T	NSD1	Missense	p.R1952L	9	152	5.9%	.	.	.
GC13	5	176720839	G	T	NSD1	Missense	p.W2157L	15	253	5.9%	.	.	.
GC13	5	176720871	G	T	NSD1	Missense	p.G2168W	15	258	5.8%	.	.	.
GC13	5	176721918	G	T	NSD1	Missense	p.G2517W	11	201	5.5%	.	.	.
GC13	5	176722158	G	T	NSD1	Missense	p.G2597W	8	142	5.6%	.	.	.
GC13	5	176722371	G	T	NSD1	Missense	p.G2668W	11	158	7.0%	.	.	.
GC13	5	176734179	C	A	MXD3	Missense	p.G175W	15	204	7.4%	.	.	.
GC13	5	176764459	C	A	LMAN2	Missense	p.G194W	10	198	5.1%	.	.	.
GC13	5	176799038	G	T	RGS14	Missense	p.G555W	11	214	5.1%	.	.	.
GC13	5	176815188	C	A	SLC34A1	Missense	p.Q280K	8	123	6.5%	.	.	.
GC13	5	176816653	G	T	SLC34A1	Missense	p.G317V	12	199	6.0%	.	.	.
GC13	5	176832079	G	T	F12	Missense	p.H169N	12	239	5.0%	.	.	.
GC13	5	176863257	C	A	GRK6	Missense	p.P414Q	7	122	5.7%	.	.	.
GC13	5	176884486	C	A	DBN1	Missense	p.W633L	11	221	5.0%	.	.	.
GC13	5	176885600	C	A	DBN1	Missense	p.R412L	8	115	7.0%	.	.	.
GC13	5	176887674	G	T	DBN1	Missense	p.H268N	11	204	5.4%	.	.	.
GC13	5	176895144	G	T	DBN1	Missense	p.Q74K	11	189	5.8%	.	.	.
GC13	5	176915130	G	T	PDLIM7	Missense	p.P330Q	11	177	6.2%	.	.	.
GC13	5	176931453	C	A	DOK3	Missense	p.R285L	6	89	6.7%	.	.	.
GC13	5	176936603	G	T	DOK3	Missense	p.P36Q	13	121	10.7%	.	.	.
GC13	5	176941963	G	T	DDX41	Missense	p.P125H	11	195	5.6%	.	.	.
GC13	5	176951689	C	A	FAM193B	Missense	p.W598L	9	199	4.5%	.	.	.
GC13	5	176951698	C	A	FAM193B	Missense	p.R595L	12	189	6.3%	.	.	.
GC13	5	176952142	C	A	FAM193B	Missense	p.R447L	12	266	4.5%	.	.	.

GC13	5	176952191	G	T	FAM193B	Missense	p.Q431K	11	235	4.7%	.	.	.
GC13	5	176966081	C	A	FAM193B	Missense	p.W93L	12	225	5.3%	.	.	.
GC13	5	177031184	G	T	B4GALT7	Missense	p.G19W	8	122	6.6%	.	.	.
GC13	5	177421138	C	A	PROP1	Missense	p.R104L	9	187	4.8%	.	.	.
GC13	5	177421184	C	A	PROP1	Missense	p.G89W	12	167	7.2%	.	.	.
GC13	5	177546769	C	A	N4BP3	Missense	p.P62H	13	226	5.8%	.	.	.
GC13	5	177547221	G	T	N4BP3	Missense	p.G125W	7	131	5.3%	.	.	.
GC13	5	177574758	G	T	RMND5B	Missense	p.W318L	11	248	4.4%	.	.	.
GC13	5	177577941	G	T	NHP2	Missense	p.P95Q	8	120	6.7%	.	.	.
GC13	5	177649522	C	A	PHYKPL	Missense	p.R213L	8	138	5.8%	.	.	.
GC13	5	177649863	G	T	PHYKPL	Missense	p.Q190K	7	88	8.0%	.	.	.
GC13	5	177658469	C	A	PHYKPL	Missense	p.G39W	15	282	5.3%	.	.	.
GC13	5	177658477	C	A	PHYKPL	Missense	p.R36L	11	275	4.0%	.	.	.
GC13	5	177673292	G	T	COL23A1	Missense	p.P459Q	7	141	5.0%	.	.	.
GC13	5	178030852	G	T	CLK4	Missense	p.P433Q	10	244	4.1%	.	.	.
GC13	5	178050284	G	T	CLK4	Missense	p.P45Q	15	279	5.4%	.	O	.
GC13	5	178139129	C	A	ZNF354A	Missense	p.G542W	14	205	6.8%	.	.	.
GC13	5	178139531	G	T	ZNF354A	Missense	p.H408N	11	145	7.6%	.	.	.
GC13	5	178139633	C	A	ZNF354A	Missense	p.G374W	12	138	8.7%	.	.	.
GC13	5	178140322	G	T	ZNF354A	Missense	p.P144Q	13	312	4.2%	.	.	.
GC13	5	178140434	G	T	ZNF354A	Missense	p.H107N	20	383	5.2%	.	.	.
GC13	5	178310801	C	A	ZNF354B	Missense	p.H450N	9	175	5.1%	.	.	.
GC13	5	178358420	C	A	ZFP2	Missense	p.H36N	12	238	5.0%	.	.	.
GC13	5	178358690	G	T	ZFP2	Missense	p.G126W	8	110	7.3%	.	.	.
GC13	5	178373399	C	A	ZNF454	Missense	p.Q25K	8	146	5.5%	.	.	.
GC13	5	178373930	C	A	ZNF454	Missense	p.Q65K	10	202	5.0%	.	.	.
GC13	5	178391659	G	T	ZNF454	Missense	p.W85L	11	219	5.0%	.	.	.
GC13	5	178392690	C	A	ZNF454	Missense	p.Q429K	9	162	5.6%	.	.	.
GC13	5	178392909	G	T	ZNF454	Missense	p.G502W	13	232	5.6%	.	.	.
GC13	5	178413651	G	T	GRM6	Missense	p.P535H	10	137	7.3%	.	.	.
GC13	5	178416296	G	T	GRM6	Missense	p.Q375K	8	96	8.3%	.	.	.

GC13	5	178417593	C	A	GRM6	Nonsense	p.G338X	8	101	7.9%	.	.	.
GC13	5	178455065	C	A	ZNF879	Missense	p.Q65K	15	188	8.0%	.	.	.
GC13	5	178459499	C	A	ZNF879	Missense	p.R184S	10	182	5.5%	.	.	.
GC13	5	178459577	G	T	ZNF879	Missense	p.G210W	13	172	7.6%	.	.	.
GC13	5	178460528	C	A	ZNF879	Missense	p.L527I	9	153	5.9%	.	.	.
GC13	5	178504161	C	A	ZNF354C	Missense	p.R82S	6	90	6.7%	.	.	.
GC13	5	178506517	G	T	ZNF354C	Missense	p.G362W	10	181	5.5%	.	.	.
GC13	5	178506601	G	T	ZNF354C	Missense	p.G390W	17	239	7.1%	.	.	.
GC13	5	178506628	C	A	ZNF354C	Missense	p.L399I	14	257	5.4%	.	.	.
GC13	5	178506937	G	T	ZNF354C	Missense	p.G502W	10	155	6.5%	.	.	.
GC13	5	178564935	C	A	ADAMTS2	Missense	p.G596W	6	99	6.1%	.	.	.
GC13	5	178987145	C	A	RUFY1	Missense	p.P36T	10	193	5.2%	.	.	.
GC13	5	179020560	C	A	RUFY1	Missense	p.H335N	11	236	4.7%	.	.	.
GC13	5	179192402	G	T	MAML1	Missense	p.G131W	10	180	5.6%	.	.	.
GC13	5	179193587	G	T	MAML1	Missense	p.G526W	14	221	6.3%	.	.	.
GC13	5	179200973	G	T	MAML1	Missense	p.G716W	11	181	6.1%	.	.	.
GC13	5	179201046	G	T	MAML1	Missense	p.R740L	9	200	4.5%	.	.	.
GC13	5	179201219	G	T	MAML1	Missense	p.G798W	9	160	5.6%	.	.	.
GC13	5	179201279	C	A	MAML1	Missense	p.P818T	10	161	6.2%	.	.	.
GC13	5	179222626	C	A	LTC4S	Missense	p.R34S	7	114	6.1%	.	.	.
GC13	5	179250993	C	A	SQSTM1	Missense	p.P146Q	12	225	5.3%	.	.	.
GC13	5	179251062	C	A	SQSTM1	Missense	p.P169H	12	217	5.5%	.	.	.
GC13	5	179263543	G	T	SQSTM1	Nonsense	p.G425X	11	202	5.4%	.	.	.
GC13	5	179275060	C	A	MRNIP	Missense	p.G45C	11	137	8.0%	.	.	.
GC13	5	179674866	G	T	MAPK9	Missense	p.P226Q	10	127	7.9%	.	.	.
GC13	5	179688785	G	C	MAPK9	Missense	p.Q117E	28	300	9.3%	.	.	.
GC13	5	179696387	C	A	MAPK9	Missense	p.G49W	14	335	4.2%	.	.	.
GC13	5	179707470	G	T	MAPK9	Missense	p.P31Q	14	235	6.0%	.	.	.
GC13	5	179739451	G	T	GFPT2	Missense	p.R509S	16	282	5.7%	.	.	.
GC13	5	179745889	C	A	GFPT2	Missense	p.G288W	9	161	5.6%	.	.	.
GC13	5	179763563	C	A	GFPT2	Missense	p.G44W	12	299	4.0%	.	.	.

GC13	5	179956364	C	A	CNOT6	Missense	p.H30N	9	168	5.4%	.	.	.
GC13	5	179976961	G	T	CNOT6	Missense	p.W48L	10	227	4.4%	.	O	.
GC13	5	179996163	C	A	CNOT6	Missense	p.H361N	10	202	5.0%	.	.	.
GC13	5	179996279	G	T	CNOT6	Missense	p.L399F	13	234	5.6%	.	.	.
GC13	5	179998328	G	T	CNOT6	Missense	p.G453W	11	221	5.0%	.	.	.
GC13	5	180036973	G	T	FLT4	Missense	p.R1247S	7	139	5.0%	.	.	.
GC13	5	180048755	C	A	FLT4	Missense	p.G603W	9	186	4.8%	.	.	.
GC13	5	180218891	G	T	MGAT1	Missense	p.R361S	8	130	6.2%	.	.	.
GC13	5	180219178	G	T	MGAT1	Missense	p.P265H	13	149	8.7%	.	.	.
GC13	5	180219194	G	T	MGAT1	Missense	p.R260S	10	147	6.8%	.	.	.
GC13	5	180219542	C	A	MGAT1	Missense	p.G144W	8	91	8.8%	.	.	.
GC13	5	180275824	G	T	ZFP62	Missense	p.L891M	13	173	7.5%	.	.	.
GC13	5	180275854	G	T	ZFP62	Missense	p.Q881K	11	173	6.4%	.	.	.
GC13	5	180276124	C	A	ZFP62	Missense	p.G791W	8	133	6.0%	.	.	.
GC13	5	180276292	C	A	ZFP62	Missense	p.G735W	9	191	4.7%	.	.	.
GC13	5	180276408	G	T	ZFP62	Missense	p.P696H	10	139	7.2%	.	.	.
GC13	5	180276741	C	A	ZFP62	Missense	p.G585V	8	124	6.5%	.	.	.
GC13	5	180278056	C	A	ZFP62	Missense	p.G147W	13	215	6.0%	.	.	.
GC13	5	180278185	G	T	ZFP62	Missense	p.Q104K	13	251	5.2%	.	.	.
GC13	5	180278391	G	T	ZFP62	Missense	p.P35H	16	268	6.0%	.	.	.
GC13	5	180335891	C	A	BTNL8	Missense	p.Q119K	10	150	6.7%	.	.	.
GC13	5	180374632	G	T	BTNL8	Missense	p.R265L	9	172	5.2%	.	.	.
GC13	5	180430855	G	T	BTNL3	Splicing	c.788-1G>T	11	199	5.5%	.	.	.
GC13	5	180552181	C	A	OR2V1	Missense	p.G42W	9	159	5.7%	.	.	.
GC13	5	180552257	C	A	OR2V1	Missense	p.L16F	12	160	7.5%	.	.	.
GC13	5	180582066	G	T	OR2V2	Missense	p.G42W	9	137	6.6%	.	.	.
GC13	5	180582399	G	T	OR2V2	Missense	p.G153W	9	173	5.2%	.	.	.
GC13	5	180582574	C	A	OR2V2	Missense	p.P211Q	14	228	6.1%	.	.	.
GC13	5	180582829	G	T	OR2V2	Missense	p.R296M	12	187	6.4%	.	.	.
GC13	5	180582840	G	T	OR2V2	Missense	p.G300W	10	177	5.6%	.	.	.
GC13	5	180651637	C	A	TRIM41	Missense	p.P213Q	9	138	6.5%	.	.	.

GC13	5	180661522	G	T	TRIM41	Missense	p.R547L	7	130	5.4%	.	.	.
GC13	5	180687165	C	A	TRIM52	Missense	p.R217L	9	176	5.1%	.	O	.
GC13	6	549196	C	A	EXOC2	Missense	p.Q739H	8	121	6.6%	.	.	.
GC13	6	1611027	G	T	FOXC1	Missense	p.R116L	7	89	7.9%	.	.	.
GC13	6	3456186	C	A	SLC22A23	Missense	p.W203L	6	50	12.0%	.	.	.
GC13	6	4070155	C	A	FAM217A	Splicing	c.303-1G>T	5	22	22.7%	.	.	.
GC13	6	5004214	C	A	RPP40	Missense	p.R8L	5	38	13.2%	.	.	.
GC13	6	5999261	G	T	NRN1	Missense	p.P126Q	6	88	6.8%	.	.	.
GC13	6	7229596	G	T	RREB1	Missense	p.G422C	7	81	8.6%	.	.	.
GC13	6	7229879	G	T	RREB1	Missense	p.R516L	6	67	9.0%	.	.	.
GC13	6	7370267	G	T	CAGE1	Missense	p.P457Q	6	82	7.3%	.	.	.
GC13	6	7580675	C	A	DSP	Missense	p.Q1418K	7	89	7.9%	.	.	.
GC13	6	7583112	C	A	DSP	Missense	p.R1274S	8	106	7.5%	.	.	.
GC13	6	7585651	C	A	DSP	Missense	p.P2120Q	6	71	8.5%	.	.	.
GC13	6	8097500	C	A	EEF1E1	Missense	p.K96N	8	93	8.6%	.	.	.
GC13	6	10586809	C	A	GCNT2	Missense	p.P196H	8	104	7.7%	.	.	.
GC13	6	10730857	G	T	TMEM14C	Missense	p.M99I	8	114	7.0%	.	.	.
GC13	6	10791969	G	T	MAK	Missense	p.H419N	10	74	13.5%	.	.	.
GC13	6	10809043	C	A	MAK	Missense	p.W164L	7	87	8.0%	.	.	.
GC13	6	10956416	G	T	SYCP2L	Missense	p.G702C	8	101	7.9%	.	.	.
GC13	6	11104828	C	A	ERVFRD-1	Missense	p.W239L	8	94	8.5%	.	.	.
GC13	6	11190616	G	T	NEDD9	Missense	p.H347N	8	88	9.1%	.	.	.
GC13	6	11575711	C	A	TMEM170B	Missense	p.P106T	7	75	9.3%	.	.	.
GC13	6	11723667	C	A	ADTRP	Missense	p.L191F	8	85	9.4%	.	.	.
GC13	6	13325353	G	T	TBC1D7	Missense	p.R56S	6	92	6.5%	.	.	.
GC13	6	13657505	C	A	RANBP9	Missense	p.W247L	7	95	7.4%	.	.	.
GC13	6	13977981	C	A	RNF182	Missense	p.L211I	8	109	7.3%	.	.	.
GC13	6	15374367	C	A	JARID2	Missense	p.P22Q	7	98	7.1%	.	.	.
GC13	6	15496700	C	A	JARID2	Missense	p.P243Q	7	99	7.1%	.	.	.
GC13	6	17421883	G	T	CAP2	Missense	p.G33W	6	72	8.3%	.	.	.
GC13	6	17675258	C	A	NUP153	Missense	p.G244W	6	48	12.5%	.	.	.

GC13	6	17764603	C	A	KIF13A	Missense	p.R1671M	7	91	7.7%	.	.	.
GC13	6	17765020	C	A	KIF13A	Missense	p.R1532L	6	89	6.7%	.	.	.
GC13	6	18256037	C	A	DEK	Missense	p.L132F	8	128	6.3%	.	.	.
GC13	6	20152931	C	A	MBOAT1	Missense	p.G57C	7	83	8.4%	.	.	.
GC13	6	20490633	C	A	E2F3	Missense	p.P326Q	6	63	9.5%	.	.	.
GC13	6	20739845	G	T	CDKAL1	Missense	p.G156V	6	56	10.7%	.	.	.
GC13	6	21065383	C	A	CDKAL1	Missense	p.P387Q	9	144	6.3%	.	.	.
GC13	6	24134700	C	A	NRSN1	Missense	p.Q49K	7	81	8.6%	.	.	.
GC13	6	24454338	C	A	GPLD1	Missense	p.G414W	5	68	7.4%	.	.	.
GC13	6	24515410	G	T	ALDH5A1	Missense	p.G248W	8	81	9.9%	.	.	.
GC13	6	24515422	G	T	ALDH5A1	Missense	p.G252C	9	87	10.3%	.	.	.
GC13	6	24828503	G	T	RIPOR2	Missense	p.Q843K	8	85	9.4%	.	.	.
GC13	6	25540182	C	A	CARMIL1	Missense	p.P735Q	6	47	12.8%	.	.	.
GC13	6	25983475	C	A	TRIM38	Missense	p.Q320K	9	103	8.7%	.	.	.
GC13	6	26091323	C	A	HFE	Missense	p.H111N	7	83	8.4%	.	.	.
GC13	6	26200099	G	T	HIST1H2BF	Missense	p.G105W	5	40	12.5%	.	O	.
GC13	6	26373308	G	T	BTN3A2	Missense	p.R300L	9	124	7.3%	.	.	.
GC13	6	26392708	C	A	BTN2A2	Missense	p.P152Q	9	98	9.2%	.	.	.
GC13	6	27860921	C	A	HIST1H2AM	Nonsense	p.G3X	7	81	8.6%	.	.	.
GC13	6	28056838	G	T	ZNF165	Missense	p.G350W	9	102	8.8%	.	.	.
GC13	6	28213340	G	T	ZKSCAN4	Missense	p.H243N	7	83	8.4%	.	.	.
GC13	6	28227259	G	T	NKAPL	Missense	p.W37L	6	58	10.3%	.	.	.
GC13	6	28268887	C	A	PGBD1	Missense	p.P419Q	9	149	6.0%	.	.	.
GC13	6	28402261	G	T	ZSCAN23	Missense	p.P384Q	7	92	7.6%	.	.	.
GC13	6	28554247	G	T	ZBED9	Missense	p.P83Q	9	103	8.7%	.	.	.
GC13	6	29141861	G	T	OR2J2	Missense	p.W150L	9	109	8.3%	.	.	.
GC13	6	29141909	G	T	OR2J2	Missense	p.W166L	9	132	6.8%	.	.	.
GC13	6	29323856	C	A	OR5V1	Missense	p.L39F	8	93	8.6%	.	.	.
GC13	6	29342338	G	T	OR12D3	Missense	p.H243N	7	89	7.9%	.	.	.
GC13	6	29364515	G	T	OR12D2	Missense	p.L13F	8	92	8.7%	.	.	.
GC13	6	29408444	G	T	OR10C1	Missense	p.G218W	7	68	10.3%	.	.	.

GC13	6	30040978	G	T	RNF39	Missense	p.P213H	8	81	9.9%	.	.	.
GC13	6	30126358	G	T	TRIM10	Missense	p.L192M	7	88	8.0%	.	.	.
GC13	6	30136254	C	A	TRIM15	Missense	p.Q219K	5	46	10.9%	.	.	.
GC13	6	30309849	G	T	TRIM39	Missense	p.R427L	6	73	8.2%	.	.	.
GC13	6	30557489	C	A	ABCF1	Missense	p.H648N	8	96	8.3%	.	.	.
GC13	6	30573708	G	T	PPP1R10	Missense	p.P282Q	8	75	10.7%	.	O	.
GC13	6	30623089	G	T	DHX16	Missense	p.Q836K	8	100	8.0%	.	.	.
GC13	6	30671460	G	T	MDC1	Missense	p.Q1834K	12	134	9.0%	.	.	.
GC13	6	30675748	G	T	MDC1	Missense	p.Q870K	11	163	6.7%	.	.	.
GC13	6	30680130	G	T	MDC1	Missense	p.P530Q	7	78	9.0%	.	.	.
GC13	6	30682940	G	T	MDC1	Missense	p.Q5K	7	76	9.2%	.	.	.
GC13	6	30954118	G	T	MUC21	Missense	p.G56W	8	102	7.8%	.	.	.
GC13	6	30995708	G	T	MUC22	Missense	p.G834W	8	120	6.7%	.	.	.
GC13	6	31118326	C	A	CCHCR1	Missense	p.R337L	5	30	16.7%	.	.	.
GC13	6	31122281	G	T	CCHCR1	Missense	p.Q229K	8	121	6.6%	.	.	.
GC13	6	31496879	G	T	MCCD1	Missense	p.G30W	6	77	7.8%	.	.	.
GC13	6	31516094	G	T	NFKBIL1	Missense	p.R71L	5	31	16.1%	.	.	.
GC13	6	31591538	G	T	PRRC2A	Missense	p.G48W	5	37	13.5%	.	.	.
GC13	6	31594860	G	T	PRRC2A	Missense	p.W392L	8	77	10.4%	.	.	.
GC13	6	31599959	G	T	PRRC2A	Missense	p.R1170L	4	37	10.8%	.	.	.
GC13	6	31602919	G	T	PRRC2A	Missense	p.R1724L	6	82	7.3%	.	.	.
GC13	6	31608571	G	T	BAG6	Missense	p.Q942K	8	97	8.2%	.	.	.
GC13	6	31636396	C	A	CSNK2B	Missense	p.R86S	5	51	9.8%	.	.	.
GC13	6	31637620	G	T	CSNK2B	Missense	p.G186C	7	72	9.7%	.	.	.
GC13	6	31708975	G	T	MSH5	Missense	p.L61F	9	91	9.9%	.	O	.
GC13	6	31750141	C	T	VARS	Missense	p.A691T	29	73	39.7%	.	.	.
GC13	6	31752010	G	T	VARS	Missense	p.P551H	7	61	11.5%	.	.	.
GC13	6	31777930	G	T	HSPA1L	Missense	p.P607H	7	72	9.7%	.	.	.
GC13	6	31856252	C	A	EHMT2	Missense	p.R489L	7	70	10.0%	.	.	.
GC13	6	31933589	G	T	SKIV2L	Missense	p.M667I	7	83	8.4%	.	.	.
GC13	6	32006908	G	T	CYP21A2	Missense	p.L80F	7	97	7.2%	.	.	.

GC13	6	32036638	C	A	TNXB	Missense	p.G1955W	9	82	11.0%	.	.	.
GC13	6	32039842	G	T	TNXB	Missense	p.R1639S	5	47	10.6%	.	.	.
GC13	6	32039958	G	T	TNXB	Missense	p.P1600H	7	91	7.7%	.	.	.
GC13	6	32050052	C	A	TNXB	Missense	p.W1166L	5	34	14.7%	.	.	.
GC13	6	32089040	C	A	ATF6B	Missense	p.Q185H	7	93	7.5%	.	.	.
GC13	6	32147501	C	A	RNF5	Missense	p.P78Q	7	97	7.2%	.	.	.
GC13	6	32163368	C	A	NOTCH4	Missense	p.W1953L	8	96	8.3%	.	.	.
GC13	6	32261486	G	T	C6orf10	Missense	p.Q322K	10	119	8.4%	.	.	.
GC13	6	32809482	C	A	PSMB8	Missense	p.G186W	6	71	8.5%	.	.	.
GC13	6	32945907	G	T	BRD2	Missense	p.R528L	10	109	9.2%	.	.	.
GC13	6	33148036	G	T	COL11A2	Missense	p.P346Q	7	92	7.6%	.	.	.
GC13	6	33162571	C	A	RXRB	Missense	p.R311L	6	82	7.3%	.	.	.
GC13	6	33286959	C	A	DAXX	Missense	p.G585W	6	71	8.5%	O	.	.
GC13	6	33288701	G	T	DAXX	Missense	p.P209H	6	56	10.7%	O	.	.
GC13	6	33384501	C	A	CUTA	Missense	p.G133W	7	89	7.9%	.	.	.
GC13	6	33384718	G	T	CUTA	Missense	p.P108Q	8	120	6.7%	.	.	.
GC13	6	33384906	C	A	CUTA	Missense	p.G88W	7	72	9.7%	.	.	.
GC13	6	33652436	G	T	ITPR3	Missense	p.R1703L	6	64	9.4%	.	.	.
GC13	6	33653458	C	A	ITPR3	Missense	p.R1841S	7	58	12.1%	.	.	.
GC13	6	34802613	G	T	UHRF1BP1	Missense	p.R215M	6	49	12.2%	.	.	.
GC13	6	34826205	G	T	UHRF1BP1	Missense	p.W691L	7	83	8.4%	.	.	.
GC13	6	34846476	C	A	TAF11	Missense	p.G144W	10	112	8.9%	.	.	.
GC13	6	35050477	C	A	ANKS1A	Missense	p.R907S	6	72	8.3%	.	.	.
GC13	6	35260366	G	T	ZNF76	Missense	p.G323W	7	56	12.5%	.	.	.
GC13	6	35588033	C	A	FKBP5	Missense	p.W90L	7	89	7.9%	.	.	.
GC13	6	35837321	C	A	SRPK1	Missense	p.R450L	6	61	9.8%	.	.	.
GC13	6	35837423	G	T	SRPK1	Missense	p.P416H	8	110	7.3%	.	.	.
GC13	6	35949898	C	A	SLC26A8	Missense	p.S237I	7	95	7.4%	.	.	.
GC13	6	36075368	C	A	MAPK14	Missense	p.P300H	8	115	7.0%	.	.	.
GC13	6	36261941	G	T	PNPLA1	Missense	p.W74L	5	34	14.7%	.	.	.
GC13	6	36336833	C	A	ETV7	Missense	p.W76L	10	64	15.6%	.	.	.

GC13	6	36690045	C	A	RAB44	Missense	p.Q712K	8	97	8.2%	.	.	.
GC13	6	36882382	C	A	C6orf89	Missense	p.P210H	7	88	8.0%	.	.	.
GC13	6	36930777	G	T	PI16	Missense	p.R220L	6	87	6.9%	.	.	.
GC13	6	36930808	G	T	PI16	Missense	p.M230I	8	98	8.2%	.	.	.
GC13	6	36930824	C	A	PI16	Missense	p.L236I	7	95	7.4%	.	.	.
GC13	6	38843548	C	A	DNAH8	Missense	p.P2601Q	8	75	10.7%	.	.	.
GC13	6	39159217	C	A	KCNK5	Missense	p.G317W	7	87	8.0%	.	.	.
GC13	6	39284659	G	T	KCNK16	Missense	p.P187Q	8	35	22.9%	.	.	.
GC13	6	41617368	G	T	MDFI	Missense	p.G30W	8	84	9.5%	.	.	.
GC13	6	41766516	C	A	USP49	Missense	p.G608W	7	116	6.0%	.	.	.
GC13	6	41884535	G	T	MED20	Missense	p.L53I	9	109	8.3%	.	.	.
GC13	6	42237220	C	A	TRERF1	Missense	p.G37W	6	60	10.0%	.	.	.
GC13	6	42620317	G	T	UBR2	Missense	p.M901I	8	113	7.1%	.	.	.
GC13	6	42631075	G	T	UBR2	Nonsense	p.G1206X	7	145	4.8%	.	O	.
GC13	6	42797031	G	T	BICRAL	Missense	p.M320I	8	116	6.9%	.	.	.
GC13	6	42797064	G	T	BICRAL	Missense	p.L331F	8	130	6.2%	.	.	.
GC13	6	42986651	C	A	KLHDC3	Missense	p.R291S	6	87	6.9%	.	.	.
GC13	6	43017805	G	T	CUL7	Missense	p.L573M	7	60	11.7%	.	.	.
GC13	6	43018031	G	T	CUL7	Missense	p.Q531K	8	117	6.8%	.	.	.
GC13	6	43019445	G	T	CUL7	Missense	p.L297M	7	85	8.2%	.	.	.
GC13	6	43022140	G	T	MRPL2	Missense	p.R264S	7	117	6.0%	.	.	.
GC13	6	43024157	C	A	MRPL2	Missense	p.G98W	6	80	7.5%	.	.	.
GC13	6	43096946	G	T	PTK7	Missense	p.R112L	5	46	10.9%	.	.	.
GC13	6	43109427	G	T	PTK7	Missense	p.W417L	7	96	7.3%	.	.	.
GC13	6	43222851	G	T	TTBK1	Missense	p.R214L	5	64	7.8%	.	.	.
GC13	6	43322613	C	A	ZNF318	Missense	p.R820M	7	99	7.1%	.	.	.
GC13	6	43324997	G	T	ZNF318	Missense	p.P352H	7	98	7.1%	.	.	.
GC13	6	43412948	G	T	ABCC10	Missense	p.G948C	7	86	8.1%	.	.	.
GC13	6	43745284	C	A	VEGFA	Missense	p.P246H	8	110	7.3%	.	.	.
GC13	6	44147777	G	T	CAPN11	Missense	p.R506L	7	85	8.2%	.	.	.
GC13	6	44223064	C	A	SLC35B2	Missense	p.M93I	10	64	15.6%	.	.	.

GC13	6	45405777	G	T	RUNX2	Missense	p.R211L	8	105	7.6%	.	.	.
GC13	6	46682266	C	A	PLA2G7	Missense	p.W134L	7	75	9.3%	.	.	.
GC13	6	46856123	C	A	ADGRF5	Missense	p.G93W	10	142	7.0%	.	.	.
GC13	6	46977213	G	T	ADGRF1	Missense	p.P653H	9	118	7.6%	.	.	.
GC13	6	46977874	C	A	ADGRF1	Missense	p.G433W	9	86	10.5%	.	.	.
GC13	6	46977987	C	A	ADGRF1	Missense	p.R395L	6	68	8.8%	.	.	.
GC13	6	47847521	C	A	PTCHD4	Missense	p.M353I	7	66	10.6%	.	.	.
GC13	6	49580226	G	T	RHAG	Missense	p.L277I	6	47	12.8%	.	.	.
GC13	6	49806191	G	T	CRISP1	Missense	p.P194Q	7	78	9.0%	.	.	.
GC13	6	50696972	C	A	TFAP2D	Missense	p.P277Q	7	68	10.3%	.	.	.
GC13	6	50810857	G	T	TFAP2B	Missense	p.G379W	6	53	11.3%	.	.	.
GC13	6	51750732	C	A	PKHD1	Missense	p.W2383L	7	77	9.1%	.	.	.
GC13	6	52053932	G	T	IL17A	Missense	p.G104W	8	67	11.9%	.	.	.
GC13	6	52303306	C	A	EFHC1	Missense	p.H164N	9	105	8.6%	.	O	.
GC13	6	53379239	G	T	GCLC	Missense	p.P168Q	7	98	7.1%	.	O	.
GC13	6	53519754	G	T	KLHL31	Missense	p.P106Q	6	72	8.3%	.	.	.
GC13	6	53764656	C	A	LRRC1	Missense	p.Q252K	8	119	6.7%	.	.	.
GC13	6	53989503	C	A	MLIP	Missense	p.P151Q	6	77	7.8%	.	.	.
GC13	6	54214596	G	T	TINAG	Missense	p.G328W	7	88	8.0%	.	.	.
GC13	6	54735124	G	T	FAM83B	Missense	p.W27L	8	128	6.3%	.	.	.
GC13	6	54806699	C	A	FAM83B	Missense	p.P977Q	7	78	9.0%	.	.	.
GC13	6	55739487	C	A	BMP5	Missense	p.L59F	9	107	8.4%	.	.	.
GC13	6	56031747	G	T	COL21A1	Missense	p.P412Q	10	127	7.9%	.	.	.
GC13	6	56032960	G	T	COL21A1	Missense	p.Q388K	7	68	10.3%	.	.	.
GC13	6	56330981	G	T	DST	Missense	p.Q4905K	8	82	9.8%	.	.	.
GC13	6	56373468	A	T	DST	Missense	p.D3743E	29	88	33.0%	.	.	.
GC13	6	56374520	G	T	DST	Missense	p.P3688Q	9	136	6.6%	.	.	.
GC13	6	56380390	C	A	DST	Missense	p.R3522L	6	76	7.9%	.	.	.
GC13	6	56420257	G	T	DST	Missense	p.Q2385K	10	137	7.3%	.	.	.
GC13	6	56422151	G	T	DST	Missense	p.P2246Q	6	61	9.8%	.	.	.
GC13	6	56436959	G	T	DST	Missense	p.L1931I	7	90	7.8%	.	.	.

GC13	6	56483809	G	T	DST	Missense	p.Q1675K	7	98	7.1%	.	.	.
GC13	6	57012325	C	A	ZNF451	Missense	p.P481Q	8	123	6.5%	.	.	.
GC13	6	57018796	G	T	ZNF451	Missense	p.E959D	7	93	7.5%	.	.	.
GC13	6	64394203	C	A	PHF3	Missense	p.H194N	8	80	10.0%	.	.	.
GC13	6	64421833	G	T	PHF3	Missense	p.W1450L	10	110	9.1%	.	.	.
GC13	6	64776312	C	A	EYS	Missense	p.G2215V	6	58	10.3%	.	.	.
GC13	6	65301424	G	T	EYS	Missense	p.R1446S	7	56	12.5%	.	.	.
GC13	6	74119047	C	A	DDX43	Missense	p.P419Q	7	81	8.6%	.	.	.
GC13	6	74228541	G	T	EEF1A1	Missense	p.R218S	4	37	10.8%	.	.	.
GC13	6	74446127	C	A	CD109	Missense	p.Q100K	6	61	9.8%	.	.	.
GC13	6	74481186	C	A	CD109	Missense	p.P493Q	7	61	11.5%	.	.	.
GC13	6	74528157	C	A	CD109	Missense	p.L1243I	6	60	10.0%	.	.	.
GC13	6	79680532	G	T	PHIP	Missense	p.P988H	6	52	11.5%	.	.	.
GC13	6	82933278	G	T	IBTK	Missense	p.L335I	6	37	16.2%	.	.	.
GC13	6	82935322	G	T	IBTK	Missense	p.Q233K	6	46	13.0%	.	.	.
GC13	6	84936096	G	T	CEP162	Missense	p.Q6K	6	52	11.5%	.	O	.
GC13	6	89600304	G	T	RNGTT	Missense	p.P209H	7	64	10.9%	.	.	.
GC13	6	89793517	C	A	PNRC1	Missense	p.H196N	7	89	7.9%	.	.	.
GC13	6	90572393	G	T	CASP8AP2	Missense	p.W322L	7	74	9.5%	.	.	.
GC13	6	90573976	C	A	CASP8AP2	Missense	p.L850M	7	67	10.4%	.	.	.
GC13	6	91257045	G	T	MAP3K7	Missense	p.P381Q	5	28	17.9%	.	.	.
GC13	6	93979281	C	A	EPHA7	Missense	p.R516L	6	69	8.7%	.	O	.
GC13	6	96054027	G	T	MANEA	Missense	p.G379W	6	58	10.3%	.	.	.
GC13	6	101090562	G	T	ASCC3	Missense	p.Q1266K	7	68	10.3%	.	.	.
GC13	6	106967066	G	T	CRYBG1	Missense	p.L253F	6	57	10.5%	.	.	.
GC13	6	109830502	G	T	AK9	Missense	p.P1454Q	6	72	8.3%	.	.	.
GC13	6	109935623	G	T	AK9	Missense	p.P487Q	8	70	11.4%	.	.	.
GC13	6	110942420	C	A	CDK19	Missense	p.G378W	5	26	19.2%	.	.	.
GC13	6	111701228	G	T	REV3L	Missense	p.Q471K	8	71	11.3%	.	.	.
GC13	6	112397658	G	T	TUBE1	Missense	p.P168Q	6	55	10.9%	.	.	.
GC13	6	116938147	G	T	RSPH4A	Missense	p.G121W	7	66	10.6%	.	.	.

GC13	6	117241534	G	T	RFX6	Missense	p.R415M	6	53	11.3%	.	.	.
GC13	6	117631307	G	T	ROS1	Missense	p.P2124Q	6	49	12.2%	.	.	.
GC13	6	117858355	C	A	DCBLD1	Missense	p.L243M	7	43	16.3%	.	.	.
GC13	6	121452793	G	T	TBC1D32	Missense	p.P960H	8	113	7.1%	.	.	.
GC13	6	122753120	G	T	HSF2	Missense	p.G438W	8	67	11.9%	.	.	.
GC13	6	122779801	G	T	SERINC1	Missense	p.P22Q	8	62	12.9%	.	.	.
GC13	6	123824926	G	T	TRDN	Missense	p.P244Q	7	81	8.6%	.	.	.
GC13	6	125404009	G	T	RNF217	Splicing	c.795-1G>T	7	92	7.6%	.	O	.
GC13	6	126210969	C	A	NCOA7	Missense	p.P475Q	7	83	8.4%	.	.	.
GC13	6	126329617	G	T	TRMT11	Missense	p.L55F	8	101	7.9%	.	.	.
GC13	6	126661474	C	A	CENPW	Missense	p.R19S	6	70	8.6%	.	.	.
GC13	6	127607898	C	A	RNF146	Missense	p.P47Q	8	130	6.2%	.	.	.
GC13	6	127768523	G	T	KIAA0408	Missense	p.P314H	7	86	8.1%	.	.	.
GC13	6	127771389	G	T	KIAA0408	Missense	p.P82T	7	95	7.4%	.	.	.
GC13	6	128134452	G	T	THEMIS	Missense	p.P445Q	10	104	9.6%	.	.	.
GC13	6	128134580	C	A	THEMIS	Missense	p.E402D	10	139	7.2%	.	.	.
GC13	6	128135049	G	T	THEMIS	Missense	p.P246H	8	103	7.8%	.	.	.
GC13	6	129475769	C	A	LAMA2	Missense	p.Q383K	6	60	10.0%	.	.	.
GC13	6	129799953	C	A	LAMA2	Missense	p.L2519M	5	40	12.5%	.	.	.
GC13	6	129899695	G	T	ARHGAP18	Missense	p.P652Q	10	124	8.1%	.	.	.
GC13	6	131190962	G	T	EPB41L2	Missense	p.P311Q	6	67	9.0%	.	.	.
GC13	6	131211457	C	A	EPB41L2	Missense	p.R144L	11	70	15.7%	.	.	.
GC13	6	131490304	G	T	AKAP7	Missense	p.Q160H	9	84	10.7%	.	.	.
GC13	6	131946011	G	T	MED23	Missense	p.P93Q	7	76	9.2%	.	.	.
GC13	6	131996328	G	T	ENPP3	Nonsense	p.G291X	8	105	7.6%	.	.	.
GC13	6	132171244	C	A	ENPP1	Missense	p.P143Q	8	118	6.8%	.	.	.
GC13	6	132645128	G	T	MOXD1	Missense	p.P352H	6	64	9.4%	.	.	.
GC13	6	132892053	G	T	TAAR6	Missense	p.W198L	7	95	7.4%	.	.	.
GC13	6	133077170	C	A	VNN2	Missense	p.G117C	5	45	11.1%	.	.	.
GC13	6	133078962	C	A	VNN2	Missense	p.G21C	8	120	6.7%	.	.	.
GC13	6	134638570	G	T	SGK1	Missense	p.P10Q	7	83	8.4%	.	.	.

GC13	6	135308833	G	T	HBS1L	Missense	p.R352S	8	89	9.0%	.	.	.
GC13	6	135511377	C	A	MYB	Missense	p.P140Q	8	110	7.3%	.	.	.
GC13	6	135516981	G	T	MYB	Missense	p.L313F	7	68	10.3%	.	.	.
GC13	6	135611629	G	T	AHI1	Missense	p.Q1174K	7	100	7.0%	.	O	.
GC13	6	135749775	G	T	AHI1	Missense	p.P872Q	9	104	8.7%	.	.	.
GC13	6	135776957	C	A	AHI1	Missense	p.W420L	7	75	9.3%	.	.	.
GC13	6	135778748	C	A	AHI1	Missense	p.L345F	8	102	7.8%	.	.	.
GC13	6	136593192	G	T	BCLAF1	Missense	p.L660M	6	46	13.0%	.	.	.
GC13	6	137528101	C	A	IFNGR1	Missense	p.G67C	7	78	9.0%	.	.	.
GC13	6	138192467	G	T	TNFAIP3	Missense	p.G35W	8	106	7.5%	O	.	.
GC13	6	138539301	G	T	PBOV1	Missense	p.H78N	8	142	5.6%	.	O	.
GC13	6	138576606	G	T	ARFGEF3	Missense	p.L268F	7	92	7.6%	.	.	.
GC13	6	138615172	G	T	ARFGEF3	Missense	p.L1137F	9	112	8.0%	.	.	.
GC13	6	138724824	G	T	HEBP2	Missense	p.G42W	5	46	10.9%	.	.	.
GC13	6	138750915	C	A	NHSL1	Missense	p.G1344W	6	60	10.0%	.	.	.
GC13	6	138753902	G	T	NHSL1	Missense	p.P531Q	8	96	8.3%	.	.	.
GC13	6	138754175	C	A	NHSL1	Missense	p.R440L	5	68	7.4%	.	.	.
GC13	6	139222150	G	T	ECT2L	Missense	p.R827L	7	105	6.7%	O	.	.
GC13	6	139234081	C	A	REPS1	Missense	p.G507C	5	30	16.7%	.	.	.
GC13	6	142759426	G	T	ADGRG6	Missense	p.G1139C	8	98	8.2%	.	.	.
GC13	6	142759487	G	T	ADGRG6	Missense	p.R1159M	7	91	7.7%	.	.	.
GC13	6	143074888	C	A	HIVEP2	Missense	p.G2233W	8	70	11.4%	.	.	.
GC13	6	143094876	C	A	HIVEP2	Missense	p.G334W	7	84	8.3%	.	.	.
GC13	6	144165687	G	T	LTV1	Missense	p.R23L	7	118	5.9%	.	.	.
GC13	6	144178506	C	A	LTV1	Missense	p.P155Q	8	124	6.5%	.	.	.
GC13	6	144750831	C	A	UTRN	Missense	p.P270Q	8	102	7.8%	.	.	.
GC13	6	144809855	G	T	UTRN	Missense	p.R1340L	6	67	9.0%	.	.	.
GC13	6	144875995	G	T	UTRN	Missense	p.R2367L	6	86	7.0%	.	O	.
GC13	6	144898369	G	T	UTRN	Missense	p.R2475L	6	93	6.5%	.	.	.
GC13	6	145119111	C	A	UTRN	Missense	p.P3077Q	8	69	11.6%	.	.	.
GC13	6	145149943	G	A	UTRN	Missense	p.R3206K	10	73	13.7%	.	.	.

GC13	6	146125798	G	T	FBXO30	Missense	p.R582S	6	86	7.0%	.	.	.
GC13	6	149699613	G	T	TAB2	Missense	p.G188C	8	117	6.8%	.	O	.
GC13	6	149699955	G	T	TAB2	Missense	p.G302C	9	150	6.0%	.	.	.
GC13	6	149918648	G	T	KATNA1	Missense	p.P306Q	6	99	6.1%	.	.	.
GC13	6	149925816	G	T	KATNA1	Missense	p.Q202K	8	111	7.2%	.	.	.
GC13	6	149982989	G	T	LATS1	Missense	p.P810Q	8	130	6.2%	.	.	.
GC13	6	150005051	C	A	LATS1	Missense	p.G112W	8	75	10.7%	.	.	.
GC13	6	150005422	C	A	LATS1	Missense	p.W268L	9	94	9.6%	.	.	.
GC13	6	150052868	C	A	NUP43	Missense	p.W265L	9	92	9.8%	.	.	.
GC13	6	150063603	G	T	NUP43	Missense	p.P142Q	8	82	9.8%	.	.	.
GC13	6	150114787	G	T	PCMT1	Missense	p.G157W	11	116	9.5%	.	.	.
GC13	6	151670719	C	A	AKAP12	Missense	p.P300Q	6	69	8.7%	.	.	.
GC13	6	151671987	G	T	AKAP12	Missense	p.G723W	7	89	7.9%	.	.	.
GC13	6	151672057	C	A	AKAP12	Missense	p.P746Q	6	79	7.6%	.	.	.
GC13	6	151687459	C	A	ZBTB2	Missense	p.G248W	9	103	8.7%	.	.	.
GC13	6	152489272	G	T	SYNE1	Missense	p.P23Q	7	91	7.7%	.	.	.
GC13	6	152552651	G	T	SYNE1	Missense	p.Q6901K	9	152	5.9%	.	.	.
GC13	6	152557954	G	T	SYNE1	Missense	p.Q6662K	8	107	7.5%	.	.	.
GC13	6	152651944	C	A	SYNE1	Missense	p.G4555W	9	127	7.1%	.	.	.
GC13	6	152673439	C	A	SYNE1	Missense	p.R3753L	6	84	7.1%	.	.	.
GC13	6	152737622	G	T	SYNE1	Missense	p.Q1991K	8	131	6.1%	.	O	.
GC13	6	152823782	C	A	SYNE1	Missense	p.G299W	9	127	7.1%	.	.	.
GC13	6	152832707	G	T	SYNE1	Missense	p.P110Q	8	95	8.4%	.	.	.
GC13	6	154412561	G	T	OPRM1	Missense	p.R273I	10	70	14.3%	.	.	.
GC13	6	155153278	G	T	SCAF8	Missense	p.L855F	7	84	8.3%	.	.	.
GC13	6	155450655	G	T	TIAM2	Missense	p.G100W	7	86	8.1%	.	.	.
GC13	6	155577685	G	T	TIAM2	Missense	p.K437N	7	71	9.9%	.	.	.
GC13	6	157405824	C	A	ARID1B	Missense	p.P676H	9	121	7.4%	O	.	.
GC13	6	157522622	G	T	ARID1B	Missense	p.V1619F	7	88	8.0%	O	.	.
GC13	6	158923610	G	T	TULP4	Missense	p.R972L	5	64	7.8%	.	.	.
GC13	6	159398662	G	T	RSPH3	Missense	p.Q435K	10	118	8.5%	.	O	.

GC13	6	159403615	G	T	RSPH3	Missense	p.L246M	8	101	7.9%	.	.	.
GC13	6	159407416	C	A	RSPH3	Missense	p.R223L	8	78	10.3%	.	.	.
GC13	6	160103640	C	A	SOD2	Missense	p.W146L	7	81	8.6%	.	.	.
GC13	6	160218528	C	A	MRPL18	Missense	p.P150Q	7	97	7.2%	.	.	.
GC13	6	160234646	C	A	PNLDC1	Missense	p.P318Q	6	74	8.1%	.	.	.
GC13	6	160328762	G	T	MAS1	Missense	p.G259W	8	92	8.7%	.	.	.
GC13	6	160671735	C	A	SLC22A2	Splicing	c.519-1G>T	6	56	10.7%	.	.	.
GC13	6	161470220	G	T	MAP3K4	Missense	p.G306W	8	115	7.0%	.	.	.
GC13	6	161587282	C	A	AGPAT4	Missense	p.G116W	7	89	7.9%	.	.	.
GC13	6	163984742	G	T	QKI	Missense	p.G301C	7	63	11.1%	O	.	.
GC13	6	166862328	C	A	RPS6KA2	Missense	p.G308W	7	87	8.0%	.	.	.
GC13	6	167753665	G	T	TTLL2	Nonsense	p.E93X	7	62	11.3%	.	O	.
GC13	6	168303056	C	A	AFDN	Missense	p.P531Q	7	107	6.5%	.	.	.
GC13	6	168709400	G	T	DACT2	Missense	p.P176Q	8	90	8.9%	.	.	.
GC13	6	170626633	G	T	FAM120B	Missense	p.W64L	10	86	11.6%	.	.	.
GC13	6	170891354	C	A	PDCD2	Splicing	c.659-1G>T	9	87	10.3%	.	.	.
GC13	7	1131387	G	T	GPER1	Missense	p.R8L	8	76	10.5%	.	.	.
GC13	7	1514401	C	A	INTS1	Missense	p.G1859V	6	40	15.0%	.	.	.
GC13	7	1519094	G	T	INTS1	Missense	p.P1434Q	5	50	10.0%	.	.	.
GC13	7	1536877	C	A	INTS1	Missense	p.R500L	7	57	12.3%	.	.	.
GC13	7	2473266	C	A	CHST12	Missense	p.P331Q	8	98	8.2%	.	.	.
GC13	7	2565128	G	T	LFNG	Missense	p.R221L	5	68	7.4%	.	.	.
GC13	7	2752065	G	T	AMZ1	Missense	p.G294W	4	32	12.5%	.	.	.
GC13	7	2978462	C	A	CARD11	Missense	p.G290W	6	61	9.8%	O	.	.
GC13	7	4245577	G	T	SDK1	Missense	p.W209L	6	43	14.0%	.	.	.
GC13	7	4308336	C	A	SDK1	Missense	p.P665Q	7	101	6.9%	.	.	.
GC13	7	4855861	C	A	RADIL	Missense	p.R655M	5	39	12.8%	.	.	.
GC13	7	5541208	C	A	FBXL18	Missense	p.R231L	7	68	10.3%	.	.	.
GC13	7	5752451	G	T	RNF216	Missense	p.P626Q	6	85	7.1%	.	.	.
GC13	7	6049025	G	T	AIMP2	Missense	p.G11W	8	89	9.0%	.	.	.
GC13	7	6187456	G	T	USP42	Missense	p.R440L	8	89	9.0%	.	.	.

GC13	7	6730559	G	T	ZNF12	Missense	p.H672N	8	107	7.5%	.	.	.
GC13	7	6730994	C	A	ZNF12	Missense	p.G527W	9	96	9.4%	.	.	.
GC13	7	7480438	C	A	COL28A1	Missense	p.G569W	15	79	19.0%	.	.	.
GC13	7	7571163	G	T	COL28A1	Missense	p.P166Q	8	76	10.5%	.	.	.
GC13	7	7613022	G	T	MIOS	Missense	p.G306W	8	104	7.7%	.	.	.
GC13	7	8126023	G	T	GLCCI1	Missense	p.R500L	8	117	6.8%	.	.	.
GC13	7	12373182	G	T	VWDE	Missense	p.P1441Q	7	99	7.1%	.	.	.
GC13	7	16640475	C	A	ANKMY2	Missense	p.G413W	7	128	5.5%	.	.	.
GC13	7	16714070	G	T	BZW2	Missense	p.R31M	7	91	7.7%	.	.	.
GC13	7	18066489	C	A	PRPS1L1	Missense	p.G306V	8	104	7.7%	.	.	.
GC13	7	18633652	G	T	HDAC9	Nonsense	p.E186X	7	89	7.9%	.	.	.
GC13	7	20449572	G	T	ITGB8	Missense	p.M754I	8	86	9.3%	.	.	.
GC13	7	20782512	G	T	ABCB5	Missense	p.G568W	8	99	8.1%	.	.	.
GC13	7	21609714	C	A	DNAH11	Missense	p.L408I	8	111	7.2%	.	.	.
GC13	7	21640485	G	T	DNAH11	Missense	p.M1064I	8	117	6.8%	.	.	.
GC13	7	21751457	C	A	DNAH11	Missense	p.P2321Q	6	63	9.5%	.	.	.
GC13	7	22162031	C	A	RAPGEF5	Missense	p.R729L	6	67	9.0%	.	.	.
GC13	7	23004085	G	T	FAM126A	Missense	p.P231Q	9	148	6.1%	.	.	.
GC13	7	23205423	C	A	KLHL7	Missense	p.P348Q	7	88	8.0%	.	.	.
GC13	7	23313721	G	T	GPNMB	Missense	p.G533W	8	111	7.2%	.	.	.
GC13	7	23313785	C	A	GPNMB	Missense	p.P554Q	9	123	7.3%	.	.	.
GC13	7	23352073	G	T	IGF2BP3	Missense	p.Q550K	6	88	6.8%	.	.	.
GC13	7	24839806	C	A	OSBPL3	Missense	p.W820L	7	81	8.6%	.	.	.
GC13	7	24874242	C	A	OSBPL3	Missense	p.G470W	6	93	6.5%	.	.	.
GC13	7	24874289	G	T	OSBPL3	Missense	p.P454Q	7	88	8.0%	.	.	.
GC13	7	27140854	G	T	HOXA2	Missense	p.Q208K	8	139	5.8%	.	.	.
GC13	7	27211549	C	A	HOXA10	Missense	p.R401L	7	67	10.4%	.	O	.
GC13	7	27211670	G	T	HOXA10	Missense	p.L361I	7	94	7.4%	.	O	.
GC13	7	27689129	G	T	HIBADH	Missense	p.P72H	9	118	7.6%	.	.	.
GC13	7	29976301	C	A	SCRN1	Missense	p.R191L	6	71	8.5%	.	.	.
GC13	7	30118327	C	A	PLEKHA8	Missense	p.P495H	11	87	12.6%	.	.	.

GC13	7	30651803	C	A	GARS	Missense	p.Q271K	7	99	7.1%	.	.	.
GC13	7	30668206	C	A	GARS	Missense	p.P523H	7	83	8.4%	.	.	.
GC13	7	30825481	G	T	MINDY4	Missense	p.W179L	8	104	7.7%	.	.	.
GC13	7	31008468	C	A	GHRHR	Missense	p.P26Q	7	93	7.5%	.	.	.
GC13	7	31682380	C	A	CCDC129	Missense	p.Q466K	9	118	7.6%	.	.	.
GC13	7	31682574	G	T	CCDC129	Missense	p.R530S	7	75	9.3%	.	.	.
GC13	7	32535414	G	T	AVL9	Missense	p.Q31H	6	46	13.0%	.	.	.
GC13	7	32598853	G	T	AVL9	Missense	p.W331L	6	62	9.7%	.	.	.
GC13	7	34192788	C	A	BMPER	Missense	p.P654Q	7	92	7.6%	.	.	.
GC13	7	35674029	C	A	HERPUD2	Nonsense	p.G318X	6	91	6.6%	.	.	.
GC13	7	35678035	G	T	HERPUD2	Missense	p.P181Q	7	93	7.5%	.	.	.
GC13	7	36656039	C	A	AOAH	Missense	p.G233W	8	72	11.1%	.	.	.
GC13	7	37780690	G	T	GPR141	Missense	p.G232V	7	76	9.2%	.	.	.
GC13	7	37953877	C	A	SFRP4	Missense	p.R177L	9	104	8.7%	O	.	.
GC13	7	38259228	G	T	STARD3NL	Missense	p.G206C	7	79	8.9%	.	.	.
GC13	7	38433643	G	T	AMPH	Missense	p.Q482K	6	75	8.0%	.	.	.
GC13	7	38835138	G	T	VPS41	Missense	p.P190Q	7	63	11.1%	.	.	.
GC13	7	38857466	G	T	VPS41	Missense	p.P109Q	8	82	9.8%	.	.	.
GC13	7	39504010	G	T	POU6F2	Missense	p.G565W	6	82	7.3%	.	O	.
GC13	7	40132616	G	T	CDK13	Missense	p.L1156F	9	131	6.9%	.	.	.
GC13	7	40314253	C	A	SUGCT	Missense	p.Q247K	5	48	10.4%	.	.	.
GC13	7	40535939	C	A	SUGCT	Missense	p.P307Q	9	111	8.1%	.	.	.
GC13	7	41739747	G	T	INHBA	Missense	p.Q76K	14	106	13.2%	.	.	.
GC13	7	42003966	G	T	GLI3	Missense	p.L1569I	8	84	9.5%	.	.	.
GC13	7	42079662	C	A	GLI3	Missense	p.G335C	7	90	7.8%	.	.	.
GC13	7	43485122	C	A	HECW1	Missense	p.P784Q	5	58	8.6%	.	.	.
GC13	7	43830858	G	T	BLVRA	Missense	p.G49W	4	37	10.8%	.	.	.
GC13	7	43846808	C	A	BLVRA	Missense	p.Q289K	7	99	7.1%	.	.	.
GC13	7	43918589	C	A	URGCP	Missense	p.W149L	8	75	10.7%	.	.	.
GC13	7	44113467	G	T	POLM	Missense	p.P330Q	6	60	10.0%	.	.	.
GC13	7	44147471	G	T	AEBP1	Missense	p.R268L	6	60	10.0%	.	.	.

GC13	7	44151479	G	T	AEBP1	Missense	p.G623W	5	39	12.8%	.	.	.
GC13	7	44579257	G	T	NPC1L1	Missense	p.Q247K	8	65	12.3%	.	.	.
GC13	7	44579353	G	T	NPC1L1	Missense	p.P215T	7	88	8.0%	.	.	.
GC13	7	44579367	C	A	NPC1L1	Missense	p.G210V	6	85	7.1%	.	.	.
GC13	7	44579368	C	G	NPC1L1	Missense	p.G210R	6	83	7.2%	.	.	.
GC13	7	44612540	C	A	DDX56	Missense	p.R111L	6	88	6.8%	.	.	.
GC13	7	44798952	G	T	ZMIZ2	Missense	p.G296W	7	73	9.6%	.	.	.
GC13	7	45006389	C	A	MYO1G	Missense	p.G611W	6	67	9.0%	.	.	.
GC13	7	45010529	G	T	MYO1G	Missense	p.L326M	7	53	13.2%	.	.	.
GC13	7	45016187	G	T	MYO1G	Missense	p.P125Q	9	89	10.1%	.	O	.
GC13	7	47463706	G	T	TNS3	Missense	p.Q156K	7	81	8.6%	.	.	.
GC13	7	47851605	C	A	PKD1L1	Missense	p.W2464L	7	76	9.2%	.	.	.
GC13	7	47853023	G	T	C7orf69	Splicing	.	10	135	7.4%	.	.	.
GC13	7	47894600	C	A	PKD1L1	Missense	p.R1580L	8	86	9.3%	.	.	.
GC13	7	47955165	C	A	PKD1L1	Missense	p.L364F	8	114	7.0%	.	.	.
GC13	7	47988001	G	T	PKD1L1	Missense	p.Q13K	8	92	8.7%	.	.	.
GC13	7	48141449	G	T	UPP1	Missense	p.R64L	4	38	10.5%	.	.	.
GC13	7	48273651	G	T	ABCA13	Missense	p.W267L	7	85	8.2%	.	.	.
GC13	7	48313212	G	T	ABCA13	Nonsense	p.G1317X	10	131	7.6%	.	.	.
GC13	7	48313527	G	T	ABCA13	Missense	p.G1422W	9	120	7.5%	.	.	.
GC13	7	48315099	G	T	ABCA13	Missense	p.G1946C	7	73	9.6%	.	.	.
GC13	7	48318920	G	T	ABCA13	Missense	p.W2710L	7	74	9.5%	.	.	.
GC13	7	48336863	G	T	ABCA13	Missense	p.G3052W	7	72	9.7%	.	.	.
GC13	7	48431598	G	T	ABCA13	Missense	p.R3912M	7	80	8.8%	.	.	.
GC13	7	48619912	G	T	ABCA13	Missense	p.W4816L	6	62	9.7%	.	.	.
GC13	7	49842376	C	A	VWC2	Missense	p.Q256K	8	70	11.4%	.	.	.
GC13	7	50180922	G	T	C7orf72	Missense	p.L341F	11	109	10.1%	.	.	.
GC13	7	50444356	C	A	IKZF1	Missense	p.H96N	7	75	9.3%	.	.	.
GC13	7	50514832	G	T	FIGNL1	Missense	p.Q52K	6	60	10.0%	.	.	.
GC13	7	50531130	C	A	DDC	Splicing	c.1243-1G>T;NM_001242888	8	79	10.1%	.	.	.
GC13	7	51096980	G	T	COBL	Missense	p.H605N	9	72	12.5%	.	.	.

GC13	7	51097058	G	T	COBL	Missense	p.L579M	7	74	9.5%	.	.	.
GC13	7	51132856	C	A	COBL	Missense	p.G463W	10	115	8.7%	.	.	.
GC13	7	54825199	G	T	SEC61G	Missense	p.P28H	8	119	6.7%	.	.	.
GC13	7	55214320	G	T	EGFR	Missense	p.R149L	6	80	7.5%	O	.	.
GC13	7	55238124	G	T	EGFR	Nonsense	p.G669X	8	92	8.7%	O	.	.
GC13	7	56007325	G	T	ZNF713	Missense	p.G320W	7	56	12.5%	.	.	.
GC13	7	56156627	C	A	PHKG1	Missense	p.R3L	7	71	9.9%	.	O	.
GC13	7	63726465	C	A	ZNF679	Missense	p.Q152K	6	50	12.0%	.	.	.
GC13	7	64452985	C	A	ERV3-1	Missense	p.M140I	7	67	10.4%	.	.	.
GC13	7	65817500	G	T	TPST1	Missense	p.K351N	9	111	8.1%	.	.	.
GC13	7	66410077	G	T	TMEM248	Missense	p.G92W	7	80	8.8%	.	.	.
GC13	7	66520836	G	T	TYW1	Missense	p.G373W	7	62	11.3%	.	.	.
GC13	7	71135054	C	A	GALNT17	Missense	p.P455Q	7	74	9.5%	.	O	.
GC13	7	72744273	C	A	FKBP6	Missense	p.P99Q	5	30	16.7%	.	.	.
GC13	7	72856717	G	T	BAZ1B	Missense	p.R1421S	7	115	6.1%	.	.	.
GC13	7	72856884	C	A	BAZ1B	Splicing	c.4095-1G>T	7	82	8.5%	.	.	.
GC13	7	72884799	G	T	BAZ1B	Missense	p.Q870K	8	77	10.4%	.	.	.
GC13	7	72957879	C	A	BCL7B	Missense	p.Q88H	7	79	8.9%	.	.	.
GC13	7	73973201	G	T	GTF2IRD1	Missense	p.L738F	5	44	11.4%	.	.	.
GC13	7	74005205	G	T	GTF2IRD1	Missense	p.R849L	5	40	12.5%	.	.	.
GC13	7	74016741	C	A	GTF2IRD1	Missense	p.P971Q	6	80	7.5%	.	O	.
GC13	7	75959381	C	A	YWHAG	Missense	p.R86L	8	131	6.1%	.	.	.
GC13	7	75959391	G	T	YWHAG	Missense	p.R83S	8	133	6.0%	.	.	.
GC13	7	76069913	C	A	ZP3	Missense	p.R349S	9	68	13.2%	.	.	.
GC13	7	76825730	G	T	FGL2	Missense	p.Q396K	8	100	8.0%	.	.	.
GC13	7	77325887	G	T	RSBN1L	Missense	p.R34L	9	111	8.1%	.	O	.
GC13	7	77378756	C	A	RSBN1L	Missense	p.P240Q	6	39	15.4%	.	.	.
GC13	7	77531175	G	T	PHTF2	Missense	p.W90L	10	134	7.5%	.	.	.
GC13	7	77569371	G	T	PHTF2	Nonsense	p.G460X	8	109	7.3%	.	.	.
GC13	7	77579038	G	T	PHTF2	Missense	p.W630L	8	82	9.8%	.	.	.
GC13	7	77579045	G	T	PHTF2	Missense	p.L632F	7	86	8.1%	.	.	.

GC13	7	77649131	C	A	MAGI2	Missense	p.R1276L	6	38	15.8%	.	.	.
GC13	7	77885581	C	A	MAGI2	Missense	p.G576C	6	70	8.6%	.	.	.
GC13	7	79840317	G	T	GNAI1	Missense	p.R156L	5	47	10.6%	.	.	.
GC13	7	80141240	C	A	GNAT3	Missense	p.M1I	7	97	7.2%	.	.	.
GC13	7	81372695	C	A	HGF	Missense	p.W280L	8	83	9.6%	.	.	.
GC13	7	82508736	G	T	PCLO	Missense	p.P4524Q	8	121	6.6%	.	.	.
GC13	7	82579741	G	T	PCLO	Missense	p.P3388H	7	100	7.0%	.	.	.
GC13	7	82581601	G	T	PCLO	Missense	p.Q2890K	7	96	7.3%	.	.	.
GC13	7	82595355	G	T	PCLO	Missense	p.P1250Q	9	121	7.4%	.	.	.
GC13	7	82785087	C	A	PCLO	Missense	p.R290S	7	78	9.0%	.	.	.
GC13	7	86394877	C	A	GRM3	Missense	p.P139Q	9	112	8.0%	.	O	.
GC13	7	86539317	C	A	KIAA1324L	Missense	p.G484W	7	59	11.9%	.	.	.
GC13	7	86823246	C	A	DMTF1	Missense	p.P531H	9	109	8.3%	.	.	.
GC13	7	86990752	C	A	CROT	Missense	p.P96Q	8	101	7.9%	.	.	.
GC13	7	87031429	C	A	ABCB4	Missense	p.G1228W	6	79	7.6%	.	.	.
GC13	7	87160783	G	T	ABCB1	Missense	p.Q838K	10	111	9.0%	.	.	.
GC13	7	87763647	G	T	ADAM22	Missense	p.G360W	7	81	8.6%	.	O	.
GC13	7	91700270	C	A	AKAP9	Missense	p.L2187I	7	98	7.1%	.	.	.
GC13	7	91707085	G	T	AKAP9	Missense	p.G2281W	8	103	7.8%	.	.	.
GC13	7	91708576	C	A	AKAP9	Missense	p.L2377I	10	93	10.8%	.	.	.
GC13	7	91708996	C	A	AKAP9	Missense	p.Q2517K	8	128	6.3%	.	.	.
GC13	7	91709125	C	A	AKAP9	Missense	p.Q2560K	8	147	5.4%	.	.	.
GC13	7	91981883	G	T	ANKIB1	Nonsense	p.G442X	8	130	6.2%	.	.	.
GC13	7	92028099	C	A	ANKIB1	Missense	p.Q1036K	10	83	12.0%	.	.	.
GC13	7	92085764	G	T	GATAD1	Missense	p.R233L	7	80	8.8%	.	.	.
GC13	7	92130903	G	T	PEX1	Missense	p.P777H	7	85	8.2%	.	.	.
GC13	7	92730894	G	T	SAMD9	Missense	p.P1506Q	11	129	8.5%	.	.	.
GC13	7	92735268	C	A	SAMD9	Missense	p.W48L	8	119	6.7%	.	.	.
GC13	7	92760769	G	T	SAMD9L	Missense	p.L1506I	9	135	6.7%	.	.	.
GC13	7	92763111	C	A	SAMD9L	Missense	p.W725L	7	94	7.4%	.	.	.
GC13	7	92764431	C	A	SAMD9L	Missense	p.R285L	7	99	7.1%	.	.	.

GC13	7	93073070	C	A	CALCR	Splicing	c.751-1G>T;NM_001742	7	96	7.3%	.	.	.
GC13	7	93555451	G	T	GNG11	Nonsense	p.G49X	6	68	8.8%	.	.	.
GC13	7	94041975	C	A	COL1A2	Missense	p.P495H	5	42	11.9%	.	.	.
GC13	7	94059575	G	T	COL1A2	Missense	p.W1324L	7	82	8.5%	.	.	.
GC13	7	94248262	G	T	SGCE	Missense	p.P116Q	5	41	12.2%	.	.	.
GC13	7	94740686	C	A	PPP1R9A	Missense	p.P504Q	11	90	12.2%	.	.	.
GC13	7	94898625	C	A	PPP1R9A	Missense	p.P937Q	6	78	7.7%	.	.	.
GC13	7	95166932	C	A	ASB4	Missense	p.P381Q	7	103	6.8%	.	.	.
GC13	7	95167056	G	T	ASB4	Missense	p.E422D	8	111	7.2%	.	.	.
GC13	7	96650142	C	A	DLX5	Missense	p.W259L	6	56	10.7%	.	.	.
GC13	7	97820060	G	T	LMTK2	Missense	p.W340L	7	93	7.5%	.	.	.
GC13	7	98030115	C	A	BAIAP2L1	Missense	p.R17L	5	49	10.2%	.	.	.
GC13	7	98495461	C	A	TRRAP	Missense	p.P202Q	6	80	7.5%	O	.	.
GC13	7	98564677	G	T	TRRAP	Missense	p.R2372L	6	66	9.1%	O	O	.
GC13	7	98574577	C	A	TRRAP	Missense	p.L2730I	9	104	8.7%	O	.	.
GC13	7	98574595	C	A	TRRAP	Missense	p.L2736M	8	109	7.3%	O	.	.
GC13	7	98990357	G	T	ARPC1B	Missense	p.G283W	5	52	9.6%	.	.	.
GC13	7	99097695	G	T	ZNF394	Missense	p.Q8K	7	92	7.6%	.	.	.
GC13	7	99145166	C	A	FAM200A	Nonsense	p.G289X	8	117	6.8%	.	.	.
GC13	7	99202041	G	T	TMEM225B	Missense	p.K10N	7	62	11.3%	.	.	.
GC13	7	99204389	G	T	TMEM225B	Missense	p.R76L	9	67	13.4%	.	.	.
GC13	7	99217398	C	A	ZSCAN25	Missense	p.Q57K	7	59	11.9%	.	O	.
GC13	7	99436325	G	T	CYP3A43	Missense	p.W64L	5	30	16.7%	.	.	.
GC13	7	99473726	G	T	OR2AE1	Missense	p.Q311K	8	104	7.7%	.	.	.
GC13	7	99473966	C	A	OR2AE1	Missense	p.G231W	7	80	8.8%	.	.	.
GC13	7	99631774	C	A	ZKSCAN1	Missense	p.P336Q	8	110	7.3%	.	.	.
GC13	7	99689362	C	A	COPS6	Missense	p.L312I	10	93	10.8%	.	.	.
GC13	7	99797239	C	A	STAG3	Missense	p.P492Q	5	50	10.0%	.	.	.
GC13	7	99821311	C	A	GATS	Missense	p.R141L	5	49	10.2%	.	.	.
GC13	7	99971778	G	T	PILRA	Missense	p.W59L	7	84	8.3%	.	.	.
GC13	7	99999555	C	A	ZCWPW1	Missense	p.M527I	7	90	7.8%	.	.	.

GC13	7	100007080	G	T	ZCWPW1	Missense	p.P281Q	6	59	10.2%	.	.	.
GC13	7	100028570	G	T	MEPCE	Missense	p.R310L	7	54	13.0%	.	.	.
GC13	7	100054529	C	A	C7orf61	Missense	p.W156L	7	88	8.0%	.	.	.
GC13	7	100088653	G	T	NYAP1	Nonsense	p.G735X	6	69	8.7%	.	.	.
GC13	7	100174580	C	A	LRCH4	Missense	p.Q471H	6	47	12.8%	.	.	.
GC13	7	100246201	G	T	ACTL6B	Missense	p.P216Q	5	39	12.8%	.	.	.
GC13	7	100304860	G	T	POP7	Missense	p.R136M	7	90	7.8%	.	.	.
GC13	7	100420133	G	T	EPHB4	Missense	p.L190M	7	79	8.9%	.	.	.
GC13	7	100463446	G	T	SLC12A9	Missense	p.R655M	8	100	8.0%	.	.	.
GC13	7	100491685	C	A	ACHE	Missense	p.G57W	5	48	10.4%	.	O	.
GC13	7	100551902	G	T	MUC3A	Missense	p.R828M	13	204	6.4%	.	.	.
GC13	7	100608335	G	T	MUC3A	Missense	p.K1300N	13	208	6.3%	.	.	.
GC13	7	100634002	G	T	MUC12	Missense	p.G53V	7	92	7.6%	.	.	.
GC13	7	100657199	C	A	MUC12	Missense	p.P5215Q	5	34	14.7%	.	.	.
GC13	7	100684481	T	A	MUC17	Missense	p.S3262T	5	74	6.8%	.	.	.
GC13	7	100686368	G	T	MUC17	Missense	p.G3891W	8	122	6.6%	.	.	.
GC13	7	100731113	G	T	TRIM56	Missense	p.G174W	6	39	15.4%	.	.	.
GC13	7	100732008	G	T	TRIM56	Missense	p.R472L	6	83	7.2%	.	O	.
GC13	7	100859482	G	T	PLOD3	Missense	p.P155H	9	94	9.6%	.	.	.
GC13	7	101842124	C	A	CUX1	Missense	p.P657Q	6	95	6.3%	O	.	.
GC13	7	102194965	A	G	SPDYE2	Missense	p.K107R	5	26	19.2%	.	O	.
GC13	7	102760211	G	T	NAPEPLD	Missense	p.Q252K	8	90	8.9%	.	.	.
GC13	7	102769037	C	A	NAPEPLD	Missense	p.G63W	8	112	7.1%	.	.	.
GC13	7	103126779	C	A	RELN	Missense	p.W3283L	7	74	9.5%	.	.	.
GC13	7	103179618	G	T	RELN	Missense	p.R2363S	5	50	10.0%	.	O	.
GC13	7	103198439	G	T	RELN	Missense	p.Q1863K	7	89	7.9%	.	.	.
GC13	7	103270544	G	T	RELN	Missense	p.Q849K	7	96	7.3%	.	.	.
GC13	7	103338316	G	T	RELN	Missense	p.P376Q	8	126	6.3%	.	.	.
GC13	7	103835605	G	T	ORC5	Missense	p.P180H	7	88	8.0%	.	.	.
GC13	7	103969484	G	T	LHFPL3	Missense	p.R86L	6	65	9.2%	.	.	.
GC13	7	104752749	G	T	KMT2E	Nonsense	p.G1516X	7	95	7.4%	.	.	.

GC13	7	105099707	C	A	PUS7	Splicing	c.1776-1G>T;NM_019042	7	69	10.1%	.	.	.
GC13	7	105909739	C	A	NAMPT	Missense	p.W156L	8	102	7.8%	.	.	.
GC13	7	106822908	C	A	HBP1	Missense	p.P97Q	8	79	10.1%	.	.	.
GC13	7	107217791	C	A	DUS4L	Missense	p.P247Q	8	93	8.6%	.	.	.
GC13	7	107341549	G	T	SLC26A4	Nonsense	p.G571X	9	103	8.7%	.	.	.
GC13	7	107399381	G	T	CBLL1	Missense	p.G411C	6	76	7.9%	.	.	.
GC13	7	107558397	C	A	DLD	Missense	p.P323Q	7	98	7.1%	.	.	.
GC13	7	107577655	G	T	LAMB1	Missense	p.Q1277K	9	121	7.4%	.	.	.
GC13	7	107602108	C	A	LAMB1	Missense	p.W624L	7	82	8.5%	.	.	.
GC13	7	107710315	G	T	LAMB4	Missense	p.Q714K	6	67	9.0%	.	.	.
GC13	7	107815800	C	A	NRCAM	Missense	p.G1052C	8	109	7.3%	.	.	.
GC13	7	107875075	C	A	NRCAM	Missense	p.R61L	7	91	7.7%	.	.	.
GC13	7	108205303	C	A	THAP5	Missense	p.G12W	6	62	9.7%	.	.	.
GC13	7	111428737	G	T	DOCK4	Missense	p.R1128S	6	55	10.9%	.	.	.
GC13	7	111503530	G	T	DOCK4	Missense	p.L791M	7	82	8.5%	.	.	.
GC13	7	112412992	C	A	TMEM168	Nonsense	p.G464X	7	90	7.8%	.	.	.
GC13	7	113519487	C	A	PPP1R3A	Missense	p.G554W	7	89	7.9%	.	.	.
GC13	7	114302213	C	A	FOXP2	Missense	p.Q580K	10	106	9.4%	.	.	.
GC13	7	115891899	C	A	TES	Missense	p.P263Q	7	93	7.5%	.	.	.
GC13	7	116166611	G	T	CAV1	Missense	p.Q21H	8	108	7.4%	.	.	.
GC13	7	116397725	G	T	MET	Missense	p.G237C	6	91	6.6%	O	.	.
GC13	7	117417740	G	T	CTTNBP2	Missense	p.P868Q	7	95	7.4%	.	.	.
GC13	7	117431784	C	A	CTTNBP2	Missense	p.R489L	6	88	6.8%	.	.	.
GC13	7	117450985	G	T	CTTNBP2	Missense	p.P83Q	6	87	6.9%	.	.	.
GC13	7	117865017	C	A	ANKRD7	Missense	p.L45I	8	91	8.8%	.	.	.
GC13	7	120385936	G	T	KCND2	Nonsense	p.G524X	8	104	7.7%	.	.	.
GC13	7	120629901	G	T	CPED1	Missense	p.G76C	7	95	7.4%	.	.	.
GC13	7	120979139	C	A	WNT16	Missense	p.H270N	7	96	7.3%	.	.	.
GC13	7	121636558	C	A	PTPRZ1	Missense	p.Q351K	7	91	7.7%	.	.	.
GC13	7	121638060	G	T	PTPRZ1	Nonsense	p.E414X	7	97	7.2%	.	.	.
GC13	7	121652938	C	A	PTPRZ1	Missense	p.H1280N	9	120	7.5%	.	O	.

GC13	7	121726085	C	A	AASS	Missense	p.R722L	9	86	10.5%	.	.	.
GC13	7	122153356	G	T	CADPS2	Missense	p.L497I	9	129	7.0%	.	.	.
GC13	7	122342248	C	A	RNF148	Missense	p.W186L	6	58	10.3%	.	.	.
GC13	7	122634907	C	A	TAS2R16	Missense	p.W261L	7	87	8.0%	.	.	.
GC13	7	122769437	C	A	SLC13A1	Missense	p.R344M	7	98	7.1%	.	.	.
GC13	7	123143318	G	T	IQUB	Missense	p.P211Q	7	103	6.8%	.	.	.
GC13	7	123267247	C	A	ASB15	Missense	p.L261I	6	45	13.3%	.	.	.
GC13	7	123335903	C	A	WASL	Missense	p.G216C	8	101	7.9%	.	.	.
GC13	7	123672250	C	A	TMEM229A	Missense	p.G270W	6	79	7.6%	.	.	.
GC13	7	124386859	C	A	GPR37	Missense	p.G521V	7	94	7.4%	.	.	.
GC13	7	124404387	G	T	GPR37	Missense	p.P215Q	6	75	8.0%	.	.	.
GC13	7	127978355	G	T	RBM28	Missense	p.L164I	7	78	9.0%	.	.	.
GC13	7	128323134	G	T	FAM71F2	Missense	p.W284L	7	96	7.3%	.	.	.
GC13	7	128478827	C	A	FLNC	Missense	p.R461S	4	35	11.4%	.	.	.
GC13	7	128488043	C	A	FLNC	Missense	p.H1501N	8	61	13.1%	.	.	.
GC13	7	128582239	G	T	IRF5	Missense	p.W35L	5	35	14.3%	.	.	.
GC13	7	128846062	G	T	SMO	Missense	p.W331L	9	82	11.0%	O	.	.
GC13	7	129350268	G	T	NRF1	Missense	p.G113W	7	76	9.2%	.	.	.
GC13	7	129765736	G	T	KLHDC10	Missense	p.W299L	9	160	5.6%	.	.	.
GC13	7	129815380	C	A	TMEM209	Missense	p.W397L	10	113	8.8%	.	.	.
GC13	7	129929571	G	T	CPA2	Missense	p.R415L	13	74	17.6%	.	O	.
GC13	7	129950728	G	T	CPA4	Missense	p.G266W	7	72	9.7%	.	.	.
GC13	7	130008323	G	T	CPA5	Missense	p.G371W	6	67	9.0%	.	.	.
GC13	7	130023540	G	T	CPA1	Missense	p.G201W	6	82	7.3%	.	.	.
GC13	7	130027737	G	T	CPA1	Missense	p.R382L	5	57	8.8%	.	.	.
GC13	7	131865475	G	T	PLXNA4	Missense	p.P1170Q	7	66	10.6%	.	.	.
GC13	7	131887416	G	T	PLXNA4	Missense	p.R859S	4	38	10.5%	.	.	.
GC13	7	132481321	G	T	CHCHD3	Missense	p.P181Q	7	91	7.7%	.	.	.
GC13	7	133314885	C	A	EXOC4	Missense	p.P502Q	8	85	9.4%	.	.	.
GC13	7	134346320	C	A	BPGM	Missense	p.R21S	8	90	8.9%	.	.	.
GC13	7	134618277	C	A	CALD1	Missense	p.H253N	11	89	12.4%	.	.	.

GC13	7	134719064	C	A	AGBL3	Missense	p.P241Q	7	101	6.9%	.	.	.
GC13	7	134719162	G	T	AGBL3	Missense	p.G274W	7	90	7.8%	.	.	.
GC13	7	134722300	C	A	AGBL3	Missense	p.P490Q	8	108	7.4%	.	.	.
GC13	7	134851461	G	T	C7orf49	Missense	p.Q113K	7	86	8.1%	.	.	.
GC13	7	134930077	C	A	STRA8	Missense	p.P148T	7	96	7.3%	.	.	.
GC13	7	135277896	G	T	NUP205	Nonsense	p.G238X	8	85	9.4%	.	.	.
GC13	7	135289184	G	T	NUP205	Missense	p.L575F	11	136	8.1%	.	.	.
GC13	7	135301970	G	T	NUP205	Missense	p.R864L	7	94	7.4%	.	.	.
GC13	7	135304358	C	A	NUP205	Missense	p.P1026Q	8	100	8.0%	.	.	.
GC13	7	135304540	G	T	NUP205	Missense	p.G1054C	8	122	6.6%	.	.	.
GC13	7	135323356	C	A	NUP205	Missense	p.P1415T	8	120	6.7%	.	.	.
GC13	7	136699730	G	T	CHRM2	Missense	p.G40W	9	82	11.0%	.	.	.
GC13	7	137569768	G	T	CREB3L2	Missense	p.L352M	7	83	8.4%	.	.	.
GC13	7	138391434	C	A	ATP6V0A4	Missense	p.G820W	5	46	10.9%	.	.	.
GC13	7	138424296	C	A	ATP6V0A4	Missense	p.G521W	8	108	7.4%	.	.	.
GC13	7	138522806	G	A	KIAA1549	Missense	p.H1900Y	34	53	64.2%	.	.	.
GC13	7	139305208	G	A	HIPK2	Missense	p.P574L	7	61	11.5%	.	.	.
GC13	7	140125700	G	T	RAB19	Missense	p.W135L	9	92	9.8%	.	.	.
GC13	7	140125861	G	T	RAB19	Missense	p.G189W	10	94	10.6%	.	O	.
GC13	7	140266903	G	T	DENND2A	Missense	p.Q588K	5	49	10.2%	.	.	.
GC13	7	140301833	G	T	DENND2A	Missense	p.P122Q	8	81	9.9%	.	.	.
GC13	7	140373701	G	T	ADCK2	Missense	p.G191W	7	98	7.1%	.	.	.
GC13	7	141170468	G	T	TMEM178B	Missense	p.W256L	6	68	8.8%	.	.	.
GC13	7	141464610	G	T	TAS2R3	Missense	p.G218W	8	66	12.1%	.	.	.
GC13	7	141618792	G	T	OR9A4	Missense	p.M39I	9	100	9.0%	.	.	.
GC13	7	141726959	G	T	MGAM	Missense	p.G343W	9	90	10.0%	.	.	.
GC13	7	141859115	C	A	MGAM2	Missense	p.P731H	6	60	10.0%	.	.	.
GC13	7	141898414	G	T	MGAM2	Splicing	c.4924-1G>T	9	147	6.1%	.	.	.
GC13	7	141920039	G	T	MGAM2	Missense	p.G1910C	8	121	6.6%	.	.	.
GC13	7	141920414	G	T	MGAM2	Missense	p.G2035C	7	98	7.1%	.	.	.
GC13	7	141955391	G	T	PRSS58	Missense	p.P48Q	5	47	10.6%	.	.	.

GC13	7	142562117	G	T	EPHB6	Missense	p.G187W	7	68	10.3%	.	.	.
GC13	7	142611823	C	A	TRPV5	Missense	p.L502F	8	112	7.1%	.	.	.
GC13	7	142626120	G	T	TRPV5	Missense	p.L195M	8	108	7.4%	.	.	.
GC13	7	142630554	C	A	TRPV5	Missense	p.M1I	7	85	8.2%	.	O	.
GC13	7	142724103	C	A	OR9A2	Missense	p.M39I	7	90	7.8%	.	O	.
GC13	7	142881237	G	T	TAS2R39	Missense	p.M242I	7	95	7.4%	.	.	.
GC13	7	142919852	G	T	TAS2R40	Missense	p.M227I	10	102	9.8%	.	.	.
GC13	7	143002055	G	T	CASP2	Missense	p.R417L	8	97	8.2%	.	.	.
GC13	7	143092269	G	T	EPHA1	Missense	p.P697Q	6	90	6.7%	.	.	.
GC13	7	143141078	G	T	TAS2R60	Missense	p.W178L	7	86	8.1%	.	O	.
GC13	7	143175128	C	A	TAS2R41	Missense	p.R55S	8	50	16.0%	.	.	.
GC13	7	143632348	G	T	OR2F2	Missense	p.W8L	6	61	9.8%	.	.	.
GC13	7	143747907	G	T	OR2A5	Missense	p.W138L	12	110	10.9%	.	.	.
GC13	7	146536985	G	T	CNTNAP2	Missense	p.G131W	7	86	8.1%	.	.	.
GC13	7	148495675	G	T	CUL1	Missense	p.R681M	7	77	9.1%	.	.	.
GC13	7	148514459	G	T	EZH2	Missense	p.P378Q	7	87	8.0%	O	.	.
GC13	7	148544378	C	A	EZH2	Missense	p.G5W	9	113	8.0%	O	O	.
GC13	7	148712105	G	T	PDIA4	Missense	p.Q169K	8	98	8.2%	.	O	.
GC13	7	148802439	C	A	ZNF425	Missense	p.R175L	7	85	8.2%	.	O	.
GC13	7	148815317	G	T	ZNF425	Missense	p.L48M	7	80	8.8%	.	.	.
GC13	7	148875927	G	T	ZNF398	Missense	p.E321D	7	80	8.8%	.	.	.
GC13	7	148876217	G	T	ZNF398	Missense	p.R418L	7	115	6.1%	.	.	.
GC13	7	148907778	C	A	ZNF282	Missense	p.P312T	8	85	9.4%	.	.	.
GC13	7	149461600	C	A	ZNF467	Missense	p.G144W	6	63	9.5%	.	.	.
GC13	7	149481219	C	A	SSPO	Missense	p.R901S	4	23	17.4%	.	.	.
GC13	7	149489697	G	T	SSPO	Missense	p.W1918L	6	56	10.7%	.	.	.
GC13	7	149559142	G	T	ZNF862	Missense	p.G965W	8	62	12.9%	.	.	.
GC13	7	149561279	G	T	ZNF862	Missense	p.R1139M	5	31	16.1%	.	.	.
GC13	7	149576654	C	A	ATP6V0E2	Missense	p.P177Q	9	97	9.3%	.	.	.
GC13	7	150034402	G	T	LRRC61	Missense	p.R151L	6	65	9.2%	.	.	.
GC13	7	150167961	G	T	GIMAP8	Missense	p.Q227H	7	100	7.0%	.	.	.

GC13	7	150438000	G	T	GIMAP5	Nonsense	p.G10X	6	49	12.2%	.	.	.
GC13	7	150553842	G	T	AOC1	Missense	p.R95L	6	61	9.8%	.	.	.
GC13	7	150553850	C	A	AOC1	Missense	p.R98S	5	60	8.3%	.	.	.
GC13	7	150648118	C	A	KCNH2	Missense	p.R339L	6	68	8.8%	.	.	.
GC13	7	150649841	C	A	KCNH2	Missense	p.W70L	6	64	9.4%	.	.	.
GC13	7	150695524	G	T	NOS3	Missense	p.R221L	9	94	9.6%	.	.	.
GC13	7	150711202	G	T	NOS3	Missense	p.R1186L	6	94	6.4%	.	.	.
GC13	7	150772563	G	T	SLC4A2	Missense	p.R1081L	8	88	9.1%	.	.	.
GC13	7	150846067	G	T	GBX1	Missense	p.P234Q	6	71	8.5%	.	.	.
GC13	7	151188101	C	A	RHEB	Splicing	c.53-1G>T	9	83	10.8%	.	.	.
GC13	7	151680091	C	A	GALNTL5	Missense	p.P130Q	9	124	7.3%	.	.	.
GC13	7	151818036	C	A	GALNT11	Missense	p.Q479K	6	54	11.1%	.	.	.
GC13	7	151842284	G	T	KMT2C	Missense	p.R4710S	9	114	7.9%	O	.	.
GC13	7	151849915	G	T	KMT2C	Missense	p.P4134Q	10	104	9.6%	O	.	.
GC13	7	151849943	C	A	KMT2C	Missense	p.G4125C	7	100	7.0%	O	.	.
GC13	7	151860469	C	A	KMT2C	Missense	p.R3398L	10	146	6.8%	O	.	.
GC13	7	151874538	G	T	KMT2C	Missense	p.P2667Q	6	82	7.3%	O	.	.
GC13	7	152520596	C	A	ACTR3B	Missense	p.P197Q	9	70	12.9%	.	.	.
GC13	7	152551624	G	T	ACTR3B	Nonsense	p.G257X	6	64	9.4%	.	.	.
GC13	7	154739638	C	A	PAXIP1	Missense	p.R966L	8	85	9.4%	.	.	.
GC13	7	154863106	G	T	HTR5A	Missense	p.W166L	6	47	12.8%	.	.	.
GC13	7	155090287	C	A	INSIG1	Missense	p.L98I	7	50	14.0%	.	.	.
GC13	7	155099995	C	A	INSIG1	Missense	p.P155Q	8	117	6.8%	.	O	.
GC13	7	155757393	G	T	LOC389602	Missense	p.G232W	6	59	10.2%	.	.	.
GC13	7	156556426	C	A	LMBR1	Missense	p.G70W	8	106	7.5%	.	.	.
GC13	7	156743197	C	A	NOM1	Missense	p.Q256K	7	71	9.9%	.	.	.
GC13	7	157929352	G	T	PTPRN2	Missense	p.Q352K	7	85	8.2%	.	O	.
GC13	7	158935221	G	T	VIPR2	Missense	p.P23Q	8	93	8.6%	.	.	.
GC13	8	1893710	G	T	ARHGEF10	Missense	p.G1058W	6	52	11.5%	.	.	.
GC13	8	2041882	C	A	MYOM2	Missense	p.Q697K	6	60	10.0%	.	.	.
GC13	8	6614739	G	T	AGPAT5	Missense	p.G309W	6	48	12.5%	.	.	.

GC13	8	8234483	G	T	PRAG1	Missense	p.P479Q	4	38	10.5%	.	.	.
GC13	8	17157535	G	T	MTMR7	Missense	p.Q607K	6	66	9.1%	.	O	.
GC13	8	17500197	C	A	PDGFRL	Missense	p.Q339K	6	67	9.0%	.	.	.
GC13	8	17722172	C	A	FGL1	Missense	p.G290W	5	43	11.6%	.	.	.
GC13	8	18729292	C	A	PSD3	Missense	p.W361L	7	71	9.9%	.	.	.
GC13	8	20007225	G	T	SLC18A1	Missense	p.L338I	7	65	10.8%	.	.	.
GC13	8	21767131	C	A	DOK2	Missense	p.L156F	6	52	11.5%	.	.	.
GC13	8	21931255	G	T	DMTN	Missense	p.L208F	6	83	7.2%	.	.	.
GC13	8	22138703	G	T	PIWIL2	Missense	p.R90L	6	77	7.8%	.	.	.
GC13	8	22147822	C	A	PIWIL2	Missense	p.H382N	6	35	17.1%	.	.	.
GC13	8	22481859	G	T	BIN3	Missense	p.P119Q	6	49	12.2%	.	.	.
GC13	8	22972206	C	A	TNFRSF10C	Missense	p.P68Q	6	67	9.0%	.	.	.
GC13	8	23538777	G	T	NKX3-1	Missense	p.P221Q	6	49	12.2%	.	.	.
GC13	8	24771769	C	A	NEFM	Missense	p.R155S	5	36	13.9%	.	.	.
GC13	8	25199254	G	T	DOCK5	Splicing	c.2440-1G>T	6	46	13.0%	.	.	.
GC13	8	25290100	G	T	KCTD9	Missense	p.L325M	5	35	14.3%	.	.	.
GC13	8	25319722	C	A	CDCA2	Missense	p.Q114K	5	39	12.8%	.	.	.
GC13	8	25340976	C	A	CDCA2	Missense	p.P350Q	7	83	8.4%	.	.	.
GC13	8	27101228	C	A	STMN4	Missense	p.A5S	5	34	14.7%	.	.	.
GC13	8	27287612	G	T	PTK2B	Missense	p.R185L	9	66	13.6%	.	.	.
GC13	8	28360581	G	T	FZD3	Missense	p.M17I	6	56	10.7%	.	.	.
GC13	8	28574160	C	A	EXTL3	Missense	p.P195Q	6	79	7.6%	.	.	.
GC13	8	30469830	G	T	GTF2E2	Missense	p.Q179K	8	48	16.7%	.	.	.
GC13	8	30706382	G	T	TEX15	Missense	p.P434Q	6	61	9.8%	.	.	.
GC13	8	33310844	G	T	FUT10	Missense	p.P89Q	6	65	9.2%	.	.	.
GC13	8	37697686	G	T	ADGRA2	Missense	p.M853I	5	37	13.5%	.	.	.
GC13	8	37993256	C	A	ASH2L	Missense	p.P425Q	7	63	11.1%	.	.	.
GC13	8	38003931	G	T	STAR	Missense	p.P114Q	8	50	16.0%	.	.	.
GC13	8	38139102	C	A	NSD3	Splicing	c.3502-1G>T	7	72	9.7%	.	.	.
GC13	8	38146132	G	T	NSD3	Missense	p.P1125Q	5	73	6.8%	.	.	.
GC13	8	38272337	C	A	FGFR1	Missense	p.R557L	7	62	11.3%	.	.	.

GC13	8	38852962	G	T	TM2D2	Missense	p.R21S	6	96	6.3%	.	.	.
GC13	8	41800501	C	A	KAT6A	Missense	p.R749L	7	72	9.7%	.	.	.
GC13	8	41834634	G	T	KAT6A	Missense	p.P419T	6	57	10.5%	.	.	.
GC13	8	41906248	G	T	KAT6A	Missense	p.P83H	8	109	7.3%	.	.	.
GC13	8	42196153	G	T	POLB	Missense	p.R4L	6	42	14.3%	.	.	.
GC13	8	42257212	C	A	VDAC3	Missense	p.P106Q	6	86	7.0%	.	.	.
GC13	8	42586932	C	A	CHRNB3	Missense	p.P161Q	6	78	7.7%	.	.	.
GC13	8	48309186	G	T	SPIDR	Missense	p.R189I	7	98	7.1%	.	.	.
GC13	8	52320898	C	A	PXDNL	Missense	p.G1096W	8	82	9.8%	.	.	.
GC13	8	52361591	G	T	PXDNL	Missense	p.P446Q	8	120	6.7%	.	.	.
GC13	8	53073992	C	A	ST18	Missense	p.G513C	7	144	4.9%	.	.	.
GC13	8	53084594	G	T	ST18	Missense	p.P276H	11	139	7.9%	.	.	.
GC13	8	53568791	G	T	RB1CC1	Missense	p.Q1200K	11	198	5.6%	.	.	.
GC13	8	53574224	G	T	RB1CC1	Missense	p.P410H	9	177	5.1%	.	.	.
GC13	8	53586756	C	A	RB1CC1	Missense	p.L217F	9	137	6.6%	.	O	.
GC13	8	53852781	G	T	NPBWR1	Missense	p.G105V	7	99	7.1%	.	.	.
GC13	8	54147549	C	A	OPRK1	Missense	p.G127V	8	99	8.1%	.	.	.
GC13	8	54978314	G	T	LYPLA1	Missense	p.P54Q	6	73	8.2%	.	.	.
GC13	8	55533848	C	A	RP1	Missense	p.H108N	6	60	10.0%	.	.	.
GC13	8	55539314	C	A	RP1	Missense	p.H958N	9	122	7.4%	.	.	.
GC13	8	55539409	G	T	RP1	Missense	p.E989D	10	134	7.5%	.	O	.
GC13	8	55540301	G	T	RP1	Missense	p.G1287C	11	122	9.0%	.	O	.
GC13	8	56436653	G	T	XKR4	Missense	p.W607L	10	109	9.2%	.	.	.
GC13	8	56699137	C	A	TGS1	Missense	p.P134H	11	136	8.1%	.	.	.
GC13	8	57080783	G	T	PLAG1	Missense	p.Q16K	8	105	7.6%	.	.	.
GC13	8	57228812	G	T	SDR16C5	Missense	p.P32Q	13	149	8.7%	.	.	.
GC13	8	59329417	G	T	UBXN2B	Missense	p.L31F	6	63	9.5%	.	.	.
GC13	8	59404300	C	A	CYP7A1	Missense	p.G417W	7	120	5.8%	.	.	.
GC13	8	59409524	C	A	CYP7A1	Missense	p.G183W	10	130	7.7%	.	.	.
GC13	8	59509959	C	A	NSMAF	Missense	p.M624I	7	98	7.1%	.	.	.
GC13	8	59515801	C	A	NSMAF	Missense	p.W369L	9	153	5.9%	.	.	.

GC13	8	61693905	C	A	CHD7	Missense	p.P671Q	6	83	7.2%	.	.	.
GC13	8	61748793	C	A	CHD7	Missense	p.Q1314K	5	49	10.2%	.	.	.
GC13	8	61757539	G	T	CHD7	Missense	p.G1656V	7	94	7.4%	.	.	.
GC13	8	61766050	C	A	CHD7	Missense	p.Q2256K	5	30	16.7%	.	.	.
GC13	8	61778158	C	A	CHD7	Missense	p.P838Q	8	137	5.8%	.	.	.
GC13	8	62366809	G	T	CLVS1	Missense	p.R247L	9	142	6.3%	.	.	.
GC13	8	63976766	C	A	TTPA	Missense	p.R221L	10	144	6.9%	.	.	.
GC13	8	65509456	C	A	CYP7B1	Missense	p.G422C	6	84	7.1%	.	.	.
GC13	8	66639448	G	T	PDE7A	Missense	p.P262H	7	91	7.7%	.	.	.
GC13	8	66691981	G	T	PDE7A	Missense	p.P86Q	9	108	8.3%	.	.	.
GC13	8	67086806	G	T	TRIM55	Missense	p.W235L	7	78	9.0%	.	.	.
GC13	8	67479011	C	A	MYBL1	Missense	p.G620W	9	159	5.7%	.	.	.
GC13	8	67479193	C	A	MYBL1	Missense	p.R588L	9	137	6.6%	.	.	.
GC13	8	67488408	G	T	MYBL1	Missense	p.P435Q	7	95	7.4%	.	.	.
GC13	8	67514708	G	T	MYBL1	Missense	p.P24Q	11	130	8.5%	.	.	.
GC13	8	67547031	C	A	VCPIP1	Missense	p.R1125L	9	124	7.3%	.	.	.
GC13	8	67547289	G	T	VCPIP1	Missense	p.P1039Q	8	148	5.4%	.	.	.
GC13	8	67547496	G	T	VCPIP1	Missense	p.P970H	10	142	7.0%	.	.	.
GC13	8	67577416	G	T	VCPIP1	Missense	p.P593H	10	155	6.5%	.	.	.
GC13	8	67590012	G	T	C8orf44	Missense	p.M23I	9	79	11.4%	.	.	.
GC13	8	67755747	C	A	SGK3	Missense	p.L347I	7	84	8.3%	.	.	.
GC13	8	67796019	G	T	MCMDC2	Missense	p.R288M	7	98	7.1%	.	.	.
GC13	8	67796105	G	T	MCMDC2	Missense	p.G317W	8	136	5.9%	.	.	.
GC13	8	67831375	G	T	MCMDC2	Missense	p.G673W	9	164	5.5%	.	.	.
GC13	8	67969619	G	T	COPS5	Missense	p.P190Q	8	101	7.9%	.	.	.
GC13	8	68130081	G	T	ARFGEF1	Missense	p.L1517I	7	100	7.0%	.	.	.
GC13	8	68137195	G	T	ARFGEF1	Missense	p.P1384Q	10	126	7.9%	.	.	.
GC13	8	68140459	C	A	ARFGEF1	Missense	p.V1141L	8	143	5.6%	.	.	.
GC13	8	68189554	G	T	ARFGEF1	Missense	p.P389H	8	106	7.5%	.	.	.
GC13	8	69005856	G	T	PREX2	Missense	p.W756L	9	133	6.8%	O	.	.
GC13	8	69017372	G	T	PREX2	Splicing	c.2716-1G>T	8	118	6.8%	O	.	.

GC13	8	69017422	G	T	PREX2	Missense	p.W922L	11	109	10.1%	O	O	.
GC13	8	69129903	G	T	PREX2	Nonsense	p.G1553X	7	104	6.7%	O	.	.
GC13	8	70536204	G	T	SULF1	Missense	p.R541L	9	135	6.7%	.	.	.
GC13	8	70744127	C	A	SLCO5A1	Missense	p.W261L	8	102	7.8%	.	.	.
GC13	8	71068744	G	T	NCOA2	Missense	p.P465Q	7	86	8.1%	.	.	.
GC13	8	71068882	G	T	NCOA2	Missense	p.P419Q	9	102	8.8%	.	.	.
GC13	8	73993380	G	T	SBSPON	Missense	p.R95S	6	83	7.2%	.	.	.
GC13	8	74162517	C	A	C8orf89	Missense	p.W108L	10	149	6.7%	.	.	.
GC13	8	74209431	G	T	RDH10	Missense	p.G98W	10	124	8.1%	.	.	.
GC13	8	76468241	G	T	HNF4G	Missense	p.G214W	7	77	9.1%	.	.	.
GC13	8	77617521	C	A	ZFHX4	Missense	p.Q400K	9	71	12.7%	.	.	.
GC13	8	77617690	C	A	ZFHX4	Missense	p.P456Q	8	131	6.1%	.	.	.
GC13	8	77618133	G	T	ZFHX4	Nonsense	p.G604X	7	95	7.4%	.	.	.
GC13	8	77618386	G	T	ZFHX4	Missense	p.R688L	6	78	7.7%	.	.	.
GC13	8	77765114	G	T	ZFHX4	Missense	p.R1986M	11	136	8.1%	.	.	.
GC13	8	77768291	G	T	ZFHX4	Missense	p.R3045L	8	104	7.7%	.	.	.
GC13	8	77775359	G	T	ZFHX4	Missense	p.G3137W	10	140	7.1%	.	.	.
GC13	8	77776322	G	T	ZFHX4	Missense	p.G3458W	7	88	8.0%	.	.	.
GC13	8	80677844	C	A	HEY1	Missense	p.R75L	7	116	6.0%	.	.	.
GC13	8	81411915	C	A	ZBTB10	Missense	p.R387S	10	191	5.2%	.	.	.
GC13	8	81412350	C	A	ZBTB10	Missense	p.Q532K	11	143	7.7%	.	.	.
GC13	8	81905338	C	A	PAG1	Missense	p.R42M	7	88	8.0%	.	.	.
GC13	8	81905409	C	A	PAG1	Missense	p.W18C	7	68	10.3%	.	.	.
GC13	8	82752075	C	A	SNX16	Missense	p.M49I	10	160	6.3%	.	.	.
GC13	8	85717036	G	T	RALYL	Missense	p.G49C	9	131	6.9%	.	.	.
GC13	8	86118455	G	T	E2F5	Missense	p.G184C	8	83	9.6%	.	.	.
GC13	8	86193495	G	T	CA13	Missense	p.G236C	8	122	6.6%	.	.	.
GC13	8	86193532	C	A	CA13	Missense	p.P248Q	10	110	9.1%	.	.	.
GC13	8	86352093	G	T	CA3	Missense	p.G63W	7	82	8.5%	.	.	.
GC13	8	86389348	G	T	CA2	Splicing	c.508-1G>T	8	106	7.5%	.	.	.
GC13	8	87226817	G	T	SLC7A13	Missense	p.P413Q	9	133	6.8%	.	O	.

GC13	8	87241981	G	T	SLC7A13	Missense	p.L176I	9	143	6.3%	.	.	.
GC13	8	87500794	G	T	RMDN1	Missense	p.Q108K	10	182	5.5%	.	.	.
GC13	8	88886009	C	A	DCAF4L2	Missense	p.W64L	9	134	6.7%	.	.	.
GC13	8	89128786	C	A	MMP16	Missense	p.G345W	8	103	7.8%	.	O	.
GC13	8	89179946	G	T	MMP16	Missense	p.H221N	8	95	8.4%	.	.	.
GC13	8	91992594	G	T	C8orf88	Missense	p.P24T	8	114	7.0%	.	.	.
GC13	8	92115038	C	A	LRRC69	Missense	p.P50H	10	128	7.8%	.	.	.
GC13	8	92382061	G	T	SLC26A7	Nonsense	p.E258X	9	137	6.6%	.	.	.
GC13	8	93004034	C	A	RUNX1T1	Missense	p.R238L	7	75	9.3%	.	O	.
GC13	8	94809423	G	T	TMEM67	Missense	p.W641L	9	124	7.3%	.	.	.
GC13	8	95507134	C	A	VIRMA	Missense	p.W1532L	9	163	5.5%	.	.	.
GC13	8	95538676	G	T	VIRMA	Missense	p.P599Q	9	165	5.5%	.	.	.
GC13	8	95565623	G	T	VIRMA	Missense	p.P18Q	6	96	6.3%	.	.	.
GC13	8	95750607	G	T	DPY19L4	Missense	p.R88L	6	94	6.4%	.	.	.
GC13	8	95862268	C	A	INTS8	Missense	p.P486T	9	152	5.9%	.	.	.
GC13	8	95879423	G	T	INTS8	Missense	p.R784L	8	123	6.5%	.	.	.
GC13	8	96166551	G	T	PLEKHF2	Missense	p.E93D	9	143	6.3%	.	.	.
GC13	8	97258134	G	T	MTERF3	Missense	p.P284Q	9	145	6.2%	.	.	.
GC13	8	97269255	G	T	MTERF3	Missense	p.P141Q	8	129	6.2%	.	.	.
GC13	8	97270752	C	A	MTERF3	Missense	p.W56L	8	139	5.8%	.	.	.
GC13	8	97342478	G	T	PTDSS1	Missense	p.W258L	8	91	8.8%	.	.	.
GC13	8	98289327	G	T	TSPYL5	Missense	p.P249Q	7	101	6.9%	.	.	.
GC13	8	98712030	G	T	MTDH	Missense	p.W366L	11	142	7.7%	.	.	.
GC13	8	98787981	G	T	LAPTM4B	Missense	p.R6L	5	34	14.7%	.	.	.
GC13	8	99148966	G	T	POP1	Missense	p.S423I	9	148	6.1%	.	.	.
GC13	8	99440530	G	T	KCNS2	Missense	p.W108L	8	140	5.7%	.	.	.
GC13	8	100182363	G	T	VPS13B	Missense	p.G769W	9	88	10.2%	.	.	.
GC13	8	100443770	C	A	VPS13B	Missense	p.R1030S	9	136	6.6%	.	.	.
GC13	8	100454698	G	T	VPS13B	Missense	p.G1094C	11	169	6.5%	.	O	.
GC13	8	100494016	G	T	VPS13B	Missense	p.G1286W	7	92	7.6%	.	.	.
GC13	8	100654376	C	A	VPS13B	Missense	p.P1878Q	8	148	5.4%	.	.	.

GC13	8	100673675	C	A	VPS13B	Missense	p.P2026H	8	110	7.3%	.	.	.
GC13	8	100732647	G	T	VPS13B	Missense	p.E2269D	8	97	8.2%	.	.	.
GC13	8	100791128	G	T	VPS13B	Missense	p.G2575W	8	122	6.6%	.	O	.
GC13	8	100844859	G	T	VPS13B	Missense	p.R3223L	7	98	7.1%	.	.	.
GC13	8	101059724	G	T	RGS22	Missense	p.P416Q	14	186	7.5%	.	.	.
GC13	8	101146462	C	A	FBXO43	Missense	p.W602L	9	134	6.7%	.	.	.
GC13	8	101153319	C	A	FBXO43	Missense	p.R388L	8	134	6.0%	.	.	.
GC13	8	101153886	C	A	FBXO43	Missense	p.R199M	8	140	5.7%	.	.	.
GC13	8	101154037	C	A	FBXO43	Missense	p.G149W	10	157	6.4%	.	.	.
GC13	8	101237442	G	T	SPAG1	Missense	p.R577L	8	132	6.1%	.	.	.
GC13	8	101299730	C	A	RNF19A	Nonsense	p.G225X	9	121	7.4%	.	.	.
GC13	8	101299847	G	T	RNF19A	Missense	p.H186N	10	174	5.7%	.	O	.
GC13	8	101300144	C	A	RNF19A	Missense	p.G87W	8	148	5.4%	.	.	.
GC13	8	101300195	G	T	RNF19A	Missense	p.L70M	9	139	6.5%	.	.	.
GC13	8	102644462	G	T	GRHL2	Missense	p.G436W	8	97	8.2%	.	.	.
GC13	8	102649140	G	A	GRHL2	Missense	p.D485N	46	152	30.3%	.	.	.
GC13	8	103288022	G	T	UBR5	Missense	p.H2182N	10	141	7.1%	O	.	.
GC13	8	103323645	G	T	UBR5	Missense	p.P833H	8	128	6.3%	O	.	.
GC13	8	103338776	G	T	UBR5	Missense	p.Q533K	8	128	6.3%	O	.	.
GC13	8	103338796	G	T	UBR5	Missense	p.P526Q	8	148	5.4%	O	.	.
GC13	8	103663811	G	T	KLF10	Missense	p.P239Q	8	113	7.1%	.	.	.
GC13	8	104064958	G	T	ATP6V1C1	Splicing	c.382-1G>T	7	77	9.1%	.	O	.
GC13	8	104394761	C	A	CTHRC1	Missense	p.P208Q	9	105	8.6%	.	.	.
GC13	8	104419907	G	T	SLC25A32	Missense	p.P87Q	9	130	6.9%	.	.	.
GC13	8	104427012	C	A	SLC25A32	Missense	p.V52L	8	123	6.5%	.	.	.
GC13	8	104709362	G	T	RIMS2	Missense	p.M75I	10	179	5.6%	.	.	.
GC13	8	104933987	C	A	RIMS2	Missense	p.P579Q	10	166	6.0%	.	.	.
GC13	8	104943572	G	T	RIMS2	Missense	p.G615W	7	72	9.7%	.	.	.
GC13	8	104948877	G	T	RIMS2	Missense	p.R664L	9	162	5.6%	.	.	.
GC13	8	105010469	G	T	RIMS2	Missense	p.R826M	9	163	5.5%	.	.	.
GC13	8	105361408	C	A	DCSTAMP	Missense	p.L210M	7	91	7.7%	.	.	.

GC13	8	105361583	C	A	DCSTAMP	Missense	p.P268Q	6	99	6.1%	.	.	.
GC13	8	105361622	C	A	DCSTAMP	Missense	p.P281Q	9	100	9.0%	.	.	.
GC13	8	105510241	G	T	LRP12	Missense	p.P161Q	9	138	6.5%	.	.	.
GC13	8	106815662	G	T	ZFPM2	Missense	p.G1118W	8	103	7.8%	.	.	.
GC13	8	107718839	G	T	OXR1	Missense	p.G357C	9	132	6.8%	.	.	.
GC13	8	108334249	T	C	ANGPT1	Missense	p.Y28C	33	125	26.4%	.	.	.
GC13	8	108509546	G	T	ANGPT1	Missense	p.Q81K	9	144	6.3%	.	.	.
GC13	8	108972943	G	T	RSPO2	Missense	p.P66Q	8	118	6.8%	.	.	.
GC13	8	109796844	C	T	TMEM74	Missense	p.D162N	51	110	46.4%	.	O	.
GC13	8	110453569	G	T	PKHD1L1	Missense	p.G1389W	9	139	6.5%	.	O	.
GC13	8	110455225	G	T	PKHD1L1	Missense	p.G1482W	8	124	6.5%	.	.	.
GC13	8	110455927	G	T	PKHD1L1	Missense	p.L1529F	8	123	6.5%	.	.	.
GC13	8	110466971	C	A	PKHD1L1	Missense	p.P2255Q	9	138	6.5%	.	.	.
GC13	8	110510886	G	T	PKHD1L1	Missense	p.G3572W	7	95	7.4%	.	.	.
GC13	8	110510895	G	T	PKHD1L1	Missense	p.G3575W	7	95	7.4%	.	.	.
GC13	8	110588168	C	A	SYBU	Missense	p.R190L	8	121	6.6%	.	.	.
GC13	8	110598392	C	A	SYBU	Splicing	c.428-1G>T;NM_017786	5	36	13.9%	.	.	.
GC13	8	113293561	G	T	CSMD3	Missense	p.P2948Q	9	103	8.7%	.	.	.
GC13	8	113347672	C	A	CSMD3	Missense	p.G2247C	7	119	5.9%	.	O	.
GC13	8	113649178	C	A	CSMD3	Missense	p.G1091W	7	113	6.2%	.	.	.
GC13	8	113649190	C	A	CSMD3	Missense	p.G1087C	8	111	7.2%	.	.	.
GC13	8	116599467	G	T	TRPS1	Missense	p.Q812K	7	66	10.6%	.	O	.
GC13	8	119391778	C	A	SAMD12	Missense	p.G162W	10	141	7.1%	.	.	.
GC13	8	120255698	G	T	MAL2	Missense	p.L167F	9	147	6.1%	.	.	.
GC13	8	120768323	G	T	TAF2	Missense	p.P934Q	8	143	5.6%	.	.	.
GC13	8	120772961	C	A	TAF2	Missense	p.R859L	9	125	7.2%	.	.	.
GC13	8	120795715	G	T	TAF2	Missense	p.P673H	11	121	9.1%	.	.	.
GC13	8	120803649	C	A	TAF2	Missense	p.W443L	8	116	6.9%	.	.	.
GC13	8	120807789	G	T	TAF2	Missense	p.R392S	6	97	6.2%	.	.	.
GC13	8	121457757	C	A	MTBP	Missense	p.P16Q	5	38	13.2%	.	.	.
GC13	8	121522211	G	T	MTBP	Missense	p.G641W	8	134	6.0%	.	.	.

GC13	8	123965341	C	A	ZHX2	Missense	p.Q531K	9	115	7.8%	.	.	.
GC13	8	124096473	G	T	TBC1D31	Missense	p.G33W	8	121	6.6%	.	O	.
GC13	8	124138427	C	A	TBC1D31	Missense	p.Q463K	9	181	5.0%	.	O	.
GC13	8	124238727	G	T	ZHX1-C8orf76	Missense	p.L289I	9	174	5.2%	.	.	.
GC13	8	124371917	G	T	ATAD2	Missense	p.P389Q	9	160	5.6%	.	.	.
GC13	8	124664296	C	A	KLHL38	Nonsense	p.G291X	7	101	6.9%	.	.	.
GC13	8	125088524	G	T	FER1L6	Missense	p.R1393L	9	101	8.9%	.	.	.
GC13	8	125463973	C	A	TRMT12	Missense	p.H269N	8	138	5.8%	.	.	.
GC13	8	125498594	C	A	RNF139	Missense	p.P235Q	8	128	6.3%	.	.	.
GC13	8	125989114	C	A	ZNF572	Missense	p.H202N	7	89	7.9%	.	.	.
GC13	8	126021483	C	A	SQLE	Missense	p.Q336K	10	169	5.9%	.	.	.
GC13	8	126448627	G	T	TRIB1	Missense	p.G179W	8	139	5.8%	.	.	.
GC13	8	127569243	G	T	FAM84B	Missense	p.P131Q	6	84	7.1%	.	.	.
GC13	8	130772791	C	A	GSDMC	Nonsense	p.E241X	8	107	7.5%	.	.	.
GC13	8	131072847	G	T	ASAP1	Missense	p.P1057Q	7	82	8.5%	.	.	.
GC13	8	131104390	C	A	ASAP1	Splicing	c.2381-1G>T	6	41	14.6%	.	.	.
GC13	8	131124346	C	A	ASAP1	Missense	p.G799W	5	45	11.1%	.	O	.
GC13	8	131792689	C	A	ADCY8	Missense	p.G1235C	7	126	5.6%	.	.	.
GC13	8	132968118	G	T	EFR3A	Missense	p.G212W	10	179	5.6%	.	.	.
GC13	8	132991167	C	A	EFR3A	Missense	p.P431Q	8	123	6.5%	.	.	.
GC13	8	132996389	G	T	EFR3A	Missense	p.G491W	9	157	5.7%	.	.	.
GC13	8	133015532	G	T	EFR3A	Missense	p.R751L	8	151	5.3%	.	O	.
GC13	8	133023077	G	T	EFR3A	Missense	p.G765W	8	117	6.8%	.	.	.
GC13	8	133078159	C	A	HHLA1	Missense	p.R509M	10	132	7.6%	.	.	.
GC13	8	133187773	G	T	KCNQ3	Missense	p.P167Q	8	125	6.4%	.	.	.
GC13	8	133726452	C	A	TMEM71	Missense	p.M191I	11	136	8.1%	.	.	.
GC13	8	133816118	G	T	PHF20L1	Missense	p.G162W	10	160	6.3%	.	.	.
GC13	8	133827032	G	T	PHF20L1	Missense	p.G335W	8	126	6.3%	.	.	.
GC13	8	133900351	G	T	TG	Missense	p.G767W	8	127	6.3%	.	.	.
GC13	8	133900606	G	T	TG	Missense	p.G852W	9	101	8.9%	.	.	.
GC13	8	133900625	G	T	TG	Missense	p.R858M	9	95	9.5%	.	.	.

GC13	8	133923746	C	A	TG	Missense	p.P1376Q	9	138	6.5%	.	.	.
GC13	8	133931647	C	A	TG	Missense	p.Q1469K	8	114	7.0%	.	.	.
GC13	8	133975249	G	T	TG	Missense	p.M1826I	8	102	7.8%	.	.	.
GC13	8	133978845	G	T	TG	Missense	p.Q1863H	8	150	5.3%	.	O	.
GC13	8	134239843	C	A	WISP1	Missense	p.R87S	7	104	6.7%	.	O	.
GC13	8	134239928	C	A	WISP1	Missense	p.P115H	9	98	9.2%	.	.	.
GC13	8	135612857	C	A	ZFAT	Missense	p.R704L	9	118	7.6%	.	.	.
GC13	8	135613894	C	A	ZFAT	Missense	p.G628C	6	76	7.9%	.	.	.
GC13	8	139163466	C	A	FAM135B	Missense	p.L1084F	10	89	11.2%	.	.	.
GC13	8	139163732	G	T	FAM135B	Missense	p.Q996K	8	118	6.8%	.	.	.
GC13	8	139164175	G	T	FAM135B	Missense	p.P848H	8	91	8.8%	.	.	.
GC13	8	139164439	C	A	FAM135B	Missense	p.R760L	9	94	9.6%	.	.	.
GC13	8	139165313	G	T	FAM135B	Missense	p.Q469K	12	126	9.5%	.	O	.
GC13	8	140922503	G	T	TRAPPC9	Missense	p.P942Q	7	81	8.6%	.	.	.
GC13	8	142221681	C	A	SLC45A4	Missense	p.G753C	8	122	6.6%	.	.	.
GC13	8	142264300	G	T	SLC45A4	Missense	p.P10Q	7	78	9.0%	.	.	.
GC13	8	142367776	C	A	GPR20	Missense	p.R83L	7	99	7.1%	.	.	.
GC13	8	143399906	G	T	TSNARE1	Missense	p.P109Q	8	84	9.5%	.	.	.
GC13	8	143412292	G	T	TSNARE1	Missense	p.P69Q	6	82	7.3%	.	.	.
GC13	8	143427121	G	T	TSNARE1	Missense	p.P74Q	6	49	12.2%	.	.	.
GC13	8	143558841	C	A	ADGRB1	Missense	p.Q440K	9	93	9.7%	.	.	.
GC13	8	144130643	G	T	C8orf31	Missense	p.G125W	9	95	9.5%	.	.	.
GC13	8	144296992	G	T	GPIHBP1	Missense	p.G96W	6	60	10.0%	.	.	.
GC13	8	144620233	G	T	ZC3H3	Missense	p.P435Q	7	72	9.7%	.	.	.
GC13	8	144681272	G	T	TIGD5	Missense	p.R400L	5	46	10.9%	.	.	.
GC13	8	144681407	C	A	TIGD5	Missense	p.P445Q	8	101	7.9%	.	.	.
GC13	8	144696506	C	A	TSTA3	Missense	p.K200N	8	122	6.6%	.	.	.
GC13	8	144696973	G	T	TSTA3	Missense	p.P131Q	7	105	6.7%	.	O	.
GC13	8	144732256	C	A	ZNF623	Missense	p.P72T	7	92	7.6%	.	.	.
GC13	8	144732257	C	A	ZNF623	Missense	p.P72H	8	93	8.6%	.	.	.
GC13	8	144800428	G	T	MAPK15	Missense	p.R81L	8	95	8.4%	.	.	.

GC13	8	144803964	C	A	MAPK15	Missense	p.Q458K	7	74	9.5%	.	.	.
GC13	8	144874682	G	T	SCRIB	Missense	p.P1434H	5	32	15.6%	.	.	.
GC13	8	144898887	G	T	PUF60	Missense	p.R449S	7	131	5.3%	.	O	.
GC13	8	144922156	G	T	NRBP2	Missense	p.P100Q	10	139	7.2%	.	.	.
GC13	8	144940683	G	T	EPPK1	Missense	p.Q2247K	14	232	6.0%	.	.	.
GC13	8	145066205	G	T	GRINA	Missense	p.G218W	10	137	7.3%	.	.	.
GC13	8	145066659	G	T	GRINA	Missense	p.M283I	6	73	8.2%	.	.	.
GC13	8	145095572	G	T	SPATC1	Missense	p.M290I	8	92	8.7%	.	.	.
GC13	8	145138381	C	A	GPAA1	Missense	p.P115Q	7	72	9.7%	.	.	.
GC13	8	145581310	G	T	FBXL6	Missense	p.L185M	6	60	10.0%	.	.	.
GC13	8	145582975	C	A	SLC52A2	Missense	p.R8S	7	85	8.2%	.	.	.
GC13	8	145584368	C	A	SLC52A2	Missense	p.L374I	8	56	14.3%	.	O	.
GC13	8	145616885	C	A	ADCK5	Missense	p.R302S	5	52	9.6%	.	.	.
GC13	8	145619361	G	T	CPSF1	Missense	p.R1276S	7	85	8.2%	.	.	.
GC13	8	145698796	G	T	KIFC2	Missense	p.W827L	6	70	8.6%	.	.	.
GC13	8	145746624	G	T	LRRC14	Missense	p.R415L	8	106	7.5%	.	.	.
GC13	8	145947163	C	A	ZNF251	Missense	p.G628W	9	103	8.7%	.	.	.
GC13	8	145948504	G	T	ZNF251	Missense	p.Q181K	17	193	8.8%	.	.	.
GC13	8	145998893	C	A	ZNF34	Missense	p.G441W	11	137	8.0%	.	.	.
GC13	8	146106962	C	A	ZNF250	Missense	p.G536W	7	88	8.0%	.	.	.
GC13	8	146107046	C	A	ZNF250	Missense	p.G508W	6	96	6.3%	.	.	.
GC13	8	146157862	G	T	ZNF16	Missense	p.P104H	8	119	6.7%	.	.	.
GC13	8	146157869	G	T	ZNF16	Missense	p.Q102K	8	126	6.3%	.	.	.
GC13	9	311993	G	T	DOCK8	Missense	p.G122W	6	62	9.7%	.	.	.
GC13	9	396825	G	T	DOCK8	Missense	p.R904L	7	98	7.1%	.	.	.
GC13	9	841913	G	T	DMRT1	Missense	p.Q25H	6	58	10.3%	.	.	.
GC13	9	1053740	G	T	DMRT2	Missense	p.G182W	8	95	8.4%	.	.	.
GC13	9	2086943	G	T	SMARCA2	Missense	p.G881W	8	95	8.4%	.	.	.
GC13	9	2097449	C	A	SMARCA2	Missense	p.P1019Q	8	103	7.8%	.	.	.
GC13	9	2635452	G	T	VLDLR	Splicing	c.83-1G>T;NM_003383	7	83	8.4%	.	O	.
GC13	9	2639970	C	A	VLDLR	Missense	p.P105Q	6	43	14.0%	.	.	.

GC13	9	2643340	G	T	VLDLR	Missense	p.W169L	7	91	7.7%	.	.	.
GC13	9	3247929	G	T	RFX3	Missense	p.Q691K	9	124	7.3%	.	.	.
GC13	9	3330405	G	T	RFX3	Missense	p.H110N	7	90	7.8%	.	.	.
GC13	9	4118709	C	A	GLIS3	Missense	p.G102W	9	123	7.3%	.	.	.
GC13	9	4604220	G	T	SPATA6L	Missense	p.P322H	8	134	6.0%	.	O	.
GC13	9	4793216	C	A	RCL1	Missense	p.P42Q	5	65	7.7%	.	.	.
GC13	9	4849446	G	T	RCL1	Splicing	c.868-1G>T;NM_001286700	8	117	6.8%	.	.	.
GC13	9	5064905	G	T	JAK2	Missense	p.R211M	8	117	6.8%	O	.	.
GC13	9	5534936	G	T	PDCD1LG2	Missense	p.G83W	6	50	12.0%	.	.	.
GC13	9	5745936	G	T	RIC1	Missense	p.W367C	9	112	8.0%	.	.	.
GC13	9	5763549	G	T	RIC1	Missense	p.G804V	10	106	9.4%	.	.	.
GC13	9	5765719	C	A	RIC1	Missense	p.Q983K	10	135	7.4%	.	.	.
GC13	9	5770123	C	A	RIC1	Missense	p.P1117Q	7	91	7.7%	.	.	.
GC13	9	5920085	C	A	KIAA2026	Missense	p.G1971W	10	120	8.3%	.	.	.
GC13	9	5922400	G	T	KIAA2026	Missense	p.P1199Q	9	142	6.3%	.	.	.
GC13	9	5922619	G	T	KIAA2026	Missense	p.P1126Q	8	127	6.3%	.	.	.
GC13	9	5969125	C	A	KIAA2026	Missense	p.W369L	9	165	5.5%	.	.	.
GC13	9	6007280	G	T	KIAA2026	Missense	p.R170S	7	111	6.3%	.	.	.
GC13	9	6007399	G	T	KIAA2026	Missense	p.P130Q	5	33	15.2%	.	.	.
GC13	9	6012738	G	T	RANBP6	Missense	p.P957Q	7	96	7.3%	.	.	.
GC13	9	6534712	G	T	GLDC	Missense	p.P972Q	8	94	8.5%	.	.	.
GC13	9	6553456	C	A	GLDC	Missense	p.R790L	7	113	6.2%	.	.	.
GC13	9	6558657	C	A	GLDC	Missense	p.G652W	9	129	7.0%	.	.	.
GC13	9	6888046	G	T	KDM4C	Missense	p.G75C	9	109	8.3%	.	.	.
GC13	9	8436690	C	A	PTPRD	Splicing	c.2741-1G>T;NM_130391	9	119	7.6%	.	.	.
GC13	9	8437216	C	A	PTPRD	Missense	p.G917C	7	72	9.7%	.	.	.
GC13	9	12698502	G	T	TYRP1	Missense	p.G254W	8	118	6.8%	.	O	.
GC13	9	13107029	G	T	MPDZ	Missense	p.H1988N	8	118	6.8%	.	.	.
GC13	9	13112058	G	T	MPDZ	Missense	p.P1835T	8	80	10.0%	.	.	.
GC13	9	13119550	C	A	MPDZ	Missense	p.G1744V	8	150	5.3%	.	.	.

GC13	9	13136796	C	A	MPDZ	Missense	p.G1370C	6	66	9.1%	.	.	.
GC13	9	14747383	G	T	FREM1	Missense	p.P499Q	8	103	7.8%	.	.	.
GC13	9	14789010	G	T	FREM1	Missense	p.Q1362K	8	119	6.7%	.	.	.
GC13	9	14801650	C	A	FREM1	Nonsense	p.G1232X	5	31	16.1%	.	.	.
GC13	9	14805043	C	A	FREM1	Missense	p.G1128W	7	96	7.3%	.	.	.
GC13	9	14823180	G	T	FREM1	Missense	p.P772Q	8	113	7.1%	.	.	.
GC13	9	14824072	G	T	FREM1	Missense	p.P707Q	6	86	7.0%	.	.	.
GC13	9	15564123	C	A	CCDC171	Missense	p.Q13K	8	100	8.0%	.	.	.
GC13	9	15784568	G	T	CCDC171	Missense	p.R1048L	7	125	5.6%	.	.	.
GC13	9	16419289	C	A	BNC2	Missense	p.G905W	7	81	8.6%	.	.	.
GC13	9	17793394	G	T	SH3GL2	Missense	p.R253M	7	87	8.0%	.	.	.
GC13	9	17795609	G	T	SH3GL2	Missense	p.L309F	7	97	7.2%	.	O	.
GC13	9	18777678	G	T	ADAMTSL1	Missense	p.G1151W	8	100	8.0%	.	.	.
GC13	9	18928688	C	A	SAXO1	Missense	p.G198W	8	111	7.2%	.	.	.
GC13	9	19049696	G	T	RRAGA	Missense	p.M13I	8	107	7.5%	.	.	.
GC13	9	19049697	G	T	RRAGA	Missense	p.G14W	8	107	7.5%	.	.	.
GC13	9	19050192	C	A	RRAGA	Missense	p.Q179K	8	120	6.7%	.	.	.
GC13	9	19050243	C	A	RRAGA	Missense	p.Q196K	8	130	6.2%	.	.	.
GC13	9	19120898	G	T	PLIN2	Missense	p.P192H	9	120	7.5%	.	.	.
GC13	9	19785943	G	T	SLC24A2	Missense	p.Q308K	8	128	6.3%	.	.	.
GC13	9	20881926	G	T	FOCAD	Missense	p.G792W	7	98	7.1%	.	O	.
GC13	9	20929501	G	T	FOCAD	Missense	p.G1075W	11	159	6.9%	.	.	.
GC13	9	21333493	G	T	KLHL9	Missense	p.H456N	11	148	7.4%	.	.	.
GC13	9	21334297	C	A	KLHL9	Missense	p.G188W	8	112	7.1%	.	.	.
GC13	9	21334668	G	T	KLHL9	Missense	p.P64H	11	164	6.7%	.	.	.
GC13	9	21409475	G	T	IFNA8	Missense	p.L100F	6	68	8.8%	.	.	.
GC13	9	21481074	G	T	IFNE	Missense	p.P207Q	11	153	7.2%	.	.	.
GC13	9	21837996	C	A	MTAP	Missense	p.P146H	7	70	10.0%	.	.	.
GC13	9	26919338	C	A	PLAA	Missense	p.G463W	7	89	7.9%	.	.	.
GC13	9	26984557	G	T	IFT74	Missense	p.M155I	9	104	8.7%	.	.	.
GC13	9	27172649	C	A	TEK	Missense	p.H118N	8	80	10.0%	.	.	.

GC13	9	27284846	C	A	EQTN	Missense	p.G225C	8	117	6.8%	.	.	.
GC13	9	27359125	C	A	MOB3B	Missense	p.M176I	9	136	6.6%	.	.	.
GC13	9	27949928	C	A	LINGO2	Missense	p.G248C	8	114	7.0%	.	.	.
GC13	9	32430486	G	T	ACO1	Missense	p.R547L	8	118	6.8%	.	.	.
GC13	9	32493824	G	T	DDX58	Missense	p.P120T	11	159	6.9%	.	.	.
GC13	9	32541879	G	T	TOPORS	Missense	p.H817N	10	109	9.2%	.	.	.
GC13	9	32543049	G	T	TOPORS	Missense	p.P427T	10	126	7.9%	.	.	.
GC13	9	32543276	C	A	TOPORS	Missense	p.W351L	11	134	8.2%	.	.	.
GC13	9	32630827	C	A	TAF1L	Missense	p.R1584L	10	131	7.6%	.	O	.
GC13	9	32631196	C	A	TAF1L	Missense	p.R1461L	8	129	6.2%	.	.	.
GC13	9	32633621	G	T	TAF1L	Missense	p.H653N	9	137	6.6%	.	.	.
GC13	9	32633875	C	A	TAF1L	Missense	p.R568M	9	172	5.2%	.	.	.
GC13	9	32634064	C	A	TAF1L	Missense	p.W505L	8	106	7.5%	.	.	.
GC13	9	32634082	C	A	TAF1L	Missense	p.W499L	9	103	8.7%	.	.	.
GC13	9	32634292	C	A	TAF1L	Missense	p.W429L	9	167	5.4%	.	.	.
GC13	9	32634554	G	T	TAF1L	Missense	p.Q342K	9	98	9.2%	.	.	.
GC13	9	33048190	G	T	SMU1	Missense	p.R453S	6	94	6.4%	.	.	.
GC13	9	33073797	G	T	SMU1	Missense	p.R12S	6	73	8.2%	.	.	.
GC13	9	33262796	G	T	BAG1	Missense	p.Q47K	8	113	7.1%	.	.	.
GC13	9	33264339	G	T	BAG1	Missense	p.Q41K	8	134	6.0%	.	.	.
GC13	9	33276502	C	A	CHMP5	Missense	p.Q146K	7	86	8.1%	.	.	.
GC13	9	33472247	C	A	NOL6	Missense	p.R73L	10	120	8.3%	.	.	.
GC13	9	33798599	G	T	PRSS3	Missense	p.E183D	6	61	9.8%	.	.	.
GC13	9	34098434	C	A	DCAF12	Missense	p.R228L	8	84	9.5%	.	.	.
GC13	9	34125268	C	A	DCAF12	Missense	p.W29L	8	124	6.5%	.	.	.
GC13	9	34257344	G	T	KIF24	Missense	p.P754Q	7	125	5.6%	.	.	.
GC13	9	34257423	G	T	KIF24	Missense	p.H728N	8	115	7.0%	.	.	.
GC13	9	34257702	C	A	KIF24	Missense	p.G635W	9	136	6.6%	.	.	.
GC13	9	34497170	C	A	DNAI1	Missense	p.Q296K	7	97	7.2%	.	.	.
GC13	9	34709623	C	A	CCL21	Missense	p.W82L	7	70	10.0%	.	.	.
GC13	9	34971425	C	A	PHF24	Missense	p.R44S	6	66	9.1%	.	.	.

GC13	9	34993328	C	A	DNAJB5	Missense	p.P105Q	9	110	8.2%	.	.	.
GC13	9	35042454	G	T	C9orf131	Missense	p.W68L	8	104	7.7%	.	.	.
GC13	9	35042865	C	A	C9orf131	Missense	p.P45Q	7	100	7.0%	.	.	.
GC13	9	35057410	G	T	VCP	Missense	p.Q760K	8	147	5.4%	.	.	.
GC13	9	35092586	C	A	PIGO	Missense	p.R433L	8	99	8.1%	.	.	.
GC13	9	35095528	C	A	PIGO	Missense	p.W12L	6	43	14.0%	.	O	.
GC13	9	35310565	G	T	UNC13B	Missense	p.R288L	9	164	5.5%	.	.	.
GC13	9	35547537	C	A	RUSC2	Missense	p.P340H	10	111	9.0%	.	.	.
GC13	9	35555982	G	T	RUSC2	Missense	p.R19L	9	98	9.2%	.	.	.
GC13	9	35556026	G	T	RUSC2	Missense	p.G34W	7	91	7.7%	.	.	.
GC13	9	35563172	C	A	FAM166B	Missense	p.G93W	7	84	8.3%	.	.	.
GC13	9	35607943	G	T	TESK1	Missense	p.G84W	9	155	5.8%	.	.	.
GC13	9	35609683	G	T	TESK1	Missense	p.G449W	7	91	7.7%	.	.	.
GC13	9	35679932	G	T	CA9	Missense	p.G383W	5	43	11.6%	.	.	.
GC13	9	35684749	G	T	TPM2	Missense	p.L207M	7	85	8.2%	.	O	.
GC13	9	35706785	G	T	TLN1	Missense	p.R1690S	9	130	6.9%	.	O	.
GC13	9	35707112	G	T	TLN1	Missense	p.R1638S	7	90	7.8%	.	.	.
GC13	9	35713235	G	T	TLN1	Missense	p.Q1104K	8	105	7.6%	.	.	.
GC13	9	35716452	G	T	TLN1	Missense	p.R854S	8	104	7.7%	.	.	.
GC13	9	35721788	C	A	TLN1	Missense	p.G321W	8	133	6.0%	.	.	.
GC13	9	35733117	G	T	CREB3	Missense	p.R85L	12	153	7.8%	.	.	.
GC13	9	35808680	G	T	NPR2	Missense	p.G963W	8	124	6.5%	.	.	.
GC13	9	35808805	G	T	NPR2	Nonsense	p.G981X	8	127	6.3%	.	.	.
GC13	9	36169840	C	A	CCIN	Missense	p.P114Q	9	135	6.7%	.	.	.
GC13	9	36170446	G	T	CCIN	Missense	p.R316L	7	92	7.6%	.	.	.
GC13	9	36651798	G	T	MELK	Missense	p.R195L	10	160	6.3%	.	.	.
GC13	9	37020744	G	T	PAX5	Missense	p.P34Q	5	40	12.5%	O	O	.
GC13	9	37426536	G	T	GRHPR	Missense	p.G97W	8	95	8.4%	.	.	.
GC13	9	37426537	G	T	GRHPR	Missense	p.G97V	8	96	8.3%	.	.	.
GC13	9	37486714	G	T	POLR1E	Missense	p.G31W	11	167	6.6%	.	.	.
GC13	9	37537181	G	T	FBXO10	Missense	p.H449N	9	97	9.3%	.	.	.

GC13	9	37537213	C	A	FBXO10	Missense	p.R438L	6	99	6.1%	.	.	.
GC13	9	37737150	G	T	FRMPD1	Missense	p.G487W	9	87	10.3%	.	.	.
GC13	9	37745021	G	T	FRMPD1	Missense	p.G998W	9	128	7.0%	.	.	.
GC13	9	37745492	G	T	FRMPD1	Missense	p.G1155C	9	100	9.0%	.	.	.
GC13	9	71395537	G	T	FAM122A	Missense	p.G195W	10	139	7.2%	.	.	.
GC13	9	71549833	G	T	PIP5K1B	Missense	p.R410L	8	109	7.3%	.	O	.
GC13	9	71628249	C	A	PRKACG	Missense	p.G254W	7	67	10.4%	.	.	.
GC13	9	71849386	G	T	TJP2	Missense	p.R572L	8	98	8.2%	.	.	.
GC13	9	72874056	C	A	SMC5	Missense	p.P21Q	8	109	7.3%	.	.	.
GC13	9	73205973	C	A	TRPM3	Missense	p.G891V	10	129	7.8%	.	.	.
GC13	9	73235187	G	T	TRPM3	Missense	p.P470H	9	120	7.5%	.	O	.
GC13	9	74337357	C	A	TMEM2	Missense	p.L688F	8	146	5.5%	.	.	.
GC13	9	74365120	C	A	TMEM2	Missense	p.R57L	15	181	8.3%	.	.	.
GC13	9	74828817	G	T	GDA	Missense	p.R163L	9	171	5.3%	.	.	.
GC13	9	74838098	G	T	GDA	Missense	p.M223I	10	126	7.9%	.	.	.
GC13	9	75545880	G	T	ALDH1A1	Missense	p.P76Q	7	120	5.8%	.	.	.
GC13	9	75783993	C	A	ANXA1	Missense	p.R303S	10	143	7.0%	.	.	.
GC13	9	77377836	G	T	TRPM6	Missense	p.H1246N	9	161	5.6%	.	.	.
GC13	9	77386680	G	T	TRPM6	Missense	p.H1154N	7	94	7.4%	.	.	.
GC13	9	77457189	C	A	TRPM6	Missense	p.G70C	10	142	7.0%	.	.	.
GC13	9	77457225	C	A	TRPM6	Missense	p.G58W	10	109	9.2%	.	.	.
GC13	9	77683920	C	A	NMRK1	Missense	p.W139L	7	91	7.7%	.	.	.
GC13	9	77692443	G	T	NMRK1	Missense	p.P28Q	13	154	8.4%	.	.	.
GC13	9	78804643	G	T	PCSK5	Missense	p.M869I	8	108	7.4%	.	.	.
GC13	9	79118045	C	A	GCNT1	Missense	p.H250N	9	116	7.8%	.	.	.
GC13	9	79118139	C	A	GCNT1	Missense	p.P281H	8	120	6.7%	.	.	.
GC13	9	79320350	C	A	PRUNE2	Missense	p.L2280F	8	146	5.5%	.	.	.
GC13	9	79321324	G	T	PRUNE2	Missense	p.Q1956K	8	113	7.1%	.	O	.
GC13	9	79321492	C	A	PRUNE2	Missense	p.G1900W	10	109	9.2%	.	.	.
GC13	9	79635403	G	T	FOXB2	Missense	p.R278L	5	57	8.8%	.	.	.
GC13	9	79852979	C	A	VPS13A	Missense	p.R553S	8	120	6.7%	.	.	.

GC13	9	79867192	C	A	VPS13A	Missense	p.Q738K	10	152	6.6%	.	.	.
GC13	9	79891052	G	T	VPS13A	Missense	p.L913F	11	184	6.0%	.	.	.
GC13	9	79933221	C	A	VPS13A	Missense	p.P1637Q	9	102	8.8%	.	.	.
GC13	9	79946979	G	T	VPS13A	Missense	p.L1976F	8	131	6.1%	.	.	.
GC13	9	84608307	G	T	SPATA31D1	Missense	p.L974F	7	95	7.4%	.	.	.
GC13	9	84609328	C	A	SPATA31D1	Missense	p.Q1315K	6	56	10.7%	.	.	.
GC13	9	85619480	C	A	RASEF	Missense	p.G379W	7	99	7.1%	.	.	.
GC13	9	85863059	G	T	FRMD3	Missense	p.P180Q	9	84	10.7%	.	.	.
GC13	9	86292870	C	A	UBQLN1	Missense	p.G293C	7	86	8.1%	.	.	.
GC13	9	86570427	C	A	C9orf64	Missense	p.G156W	9	151	6.0%	.	O	.
GC13	9	86616452	C	A	RMI1	Missense	p.P184Q	8	110	7.3%	.	.	.
GC13	9	87359975	G	T	NTRK2	Missense	p.R428L	6	80	7.5%	.	O	.
GC13	9	88247587	C	A	AGTPBP1	Missense	p.G721C	8	120	6.7%	.	.	.
GC13	9	88292407	G	T	AGTPBP1	Missense	p.P179Q	9	119	7.6%	.	.	.
GC13	9	88622329	G	T	NAA35	Missense	p.M391I	8	116	6.9%	.	.	.
GC13	9	88632419	C	A	NAA35	Missense	p.P571Q	9	110	8.2%	.	.	.
GC13	9	88633210	C	A	NAA35	Missense	p.P605Q	8	124	6.5%	.	.	.
GC13	9	88694217	C	A	GOLM1	Missense	p.G7W	5	31	16.1%	.	.	.
GC13	9	88943337	C	A	ZCCHC6	Missense	p.W386L	9	133	6.8%	.	.	.
GC13	9	90252982	C	A	DAPK1	Missense	p.H137N	10	147	6.8%	.	.	.
GC13	9	90296412	G	T	DAPK1	Missense	p.G699W	10	141	7.1%	.	.	.
GC13	9	90321842	G	T	DAPK1	Missense	p.G1286W	10	96	10.4%	.	.	.
GC13	9	90499934	G	T	SPATA31E1	Missense	p.G178W	8	85	9.4%	.	.	.
GC13	9	90500349	G	T	SPATA31E1	Missense	p.W316L	7	85	8.2%	.	O	.
GC13	9	90502053	G	T	SPATA31E1	Missense	p.R884L	8	76	10.5%	.	.	.
GC13	9	90589331	C	A	CDK20	Missense	p.G11W	6	97	6.2%	.	.	.
GC13	9	91606044	C	A	C9orf47	Missense	p.P45Q	9	133	6.8%	.	.	.
GC13	9	91616171	G	T	S1PR3	Missense	p.R19L	8	93	8.6%	.	.	.
GC13	9	91965679	G	T	SECISBP2	Missense	p.L674F	7	89	7.9%	.	.	.
GC13	9	92002367	G	T	SEMA4D	Missense	p.Q422K	10	108	9.3%	.	.	.
GC13	9	93376012	C	A	DIRAS2	Missense	p.R33L	8	97	8.2%	.	.	.

GC13	9	93376043	G	T	DIRAS2	Missense	p.L23M	9	101	8.9%	.	.	.
GC13	9	93639960	C	A	SYK	Missense	p.P407Q	9	108	8.3%	.	.	.
GC13	9	93978373	C	A	AUH	Missense	p.G275W	9	168	5.4%	.	.	.
GC13	9	94499781	C	A	ROR2	Missense	p.G172W	8	138	5.8%	.	.	.
GC13	9	94794759	G	T	SPTLC1	Missense	p.R460S	6	95	6.3%	.	.	.
GC13	9	95015658	G	T	IARS	Missense	p.R739S	8	122	6.6%	.	.	.
GC13	9	95076712	C	A	NOL8	Missense	p.R732L	10	117	8.5%	.	.	.
GC13	9	95081544	G	T	NOL8	Missense	p.H126N	7	101	6.9%	.	.	.
GC13	9	95155442	G	T	OGN	Missense	p.P118Q	8	132	6.1%	.	.	.
GC13	9	95179291	C	A	OMD	Missense	p.G184W	8	144	5.6%	.	.	.
GC13	9	95277180	G	T	ECM2	Missense	p.Q241K	12	145	8.3%	.	.	.
GC13	9	95279986	G	T	ECM2	Missense	p.P155Q	10	108	9.3%	.	.	.
GC13	9	95609733	C	A	ZNF484	Missense	p.G410W	8	99	8.1%	.	.	.
GC13	9	95609972	G	T	ZNF484	Missense	p.P330H	9	133	6.8%	.	.	.
GC13	9	96000572	G	T	WNK2	Missense	p.G431W	9	104	8.7%	.	.	.
GC13	9	96080257	G	T	WNK2	Missense	p.R2281L	7	95	7.4%	.	.	.
GC13	9	96080814	C	A	C9orf129	Missense	p.G153W	7	85	8.2%	.	.	.
GC13	9	96214507	C	A	FAM120A	Missense	p.R162L	8	153	5.2%	.	.	.
GC13	9	96214922	G	T	FAM120AOS	Missense	p.Q24K	6	47	12.8%	.	.	.
GC13	9	96291697	G	T	FAM120A	Missense	p.M522I	9	111	8.1%	.	.	.
GC13	9	96291920	G	T	FAM120A	Missense	p.G598W	10	111	9.0%	.	.	.
GC13	9	96420505	G	T	PHF2	Missense	p.W409L	5	40	12.5%	.	.	.
GC13	9	96428129	C	A	PHF2	Missense	p.P700Q	6	82	7.3%	.	.	.
GC13	9	96439914	G	T	PHF2	Missense	p.G1083W	6	63	9.5%	.	.	.
GC13	9	96860607	G	T	PTPDC1	Missense	p.G587W	8	107	7.5%	.	.	.
GC13	9	97062810	G	T	ZNF169	Missense	p.G133W	7	83	8.4%	.	.	.
GC13	9	97063062	G	T	ZNF169	Missense	p.G217W	9	118	7.6%	.	.	.
GC13	9	97563122	G	T	C9orf3	Missense	p.R401L	8	126	6.3%	.	.	.
GC13	9	98728934	G	T	ERCC6L2	Missense	p.G691W	8	118	6.8%	.	.	.
GC13	9	98774940	G	T	ERCC6L2	Missense	p.G1381W	11	157	7.0%	.	.	.
GC13	9	98775189	G	T	ERCC6L2	Missense	p.G1464W	9	150	6.0%	.	.	.

GC13	9	99114320	C	A	SLC35D2	Missense	p.G140V	9	136	6.6%	.	.	.
GC13	9	99114321	C	A	SLC35D2	Missense	p.G140W	10	135	7.4%	.	.	.
GC13	9	99233355	C	A	HABP4	Missense	p.P269Q	6	87	6.9%	.	.	.
GC13	9	99525402	G	T	ZNF510	Missense	p.P55Q	8	113	7.1%	.	.	.
GC13	9	99798888	C	A	CTSV	Missense	p.G180C	9	159	5.7%	.	.	.
GC13	9	99800259	G	T	CTSV	Missense	p.Q23K	10	143	7.0%	.	.	.
GC13	9	100128866	C	A	CCDC180	Missense	p.P1402Q	7	90	7.8%	.	.	.
GC13	9	100451847	G	T	XPA	Missense	p.H120N	7	65	10.8%	O	.	.
GC13	9	101068605	C	A	GABBR2	Missense	p.W676L	8	84	9.5%	.	.	.
GC13	9	101068621	C	A	GABBR2	Missense	p.G671C	6	81	7.4%	.	.	.
GC13	9	101125029	G	T	GABBR2	Missense	p.L621M	8	93	8.6%	.	.	.
GC13	9	101151191	C	A	GABBR2	Missense	p.G492W	7	123	5.7%	.	O	.
GC13	9	101540594	G	A	ANKS6	Missense	p.A494V	46	63	73.0%	.	.	.
GC13	9	101814715	G	T	COL15A1	Missense	p.G984C	10	119	8.4%	.	.	.
GC13	9	101984648	G	T	SEC61B	Missense	p.M1I	7	86	8.1%	.	.	.
GC13	9	102607028	G	T	NR4A3	Missense	p.W451L	7	80	8.8%	.	O	.
GC13	9	102888700	G	T	INVS	Missense	p.G48W	8	116	6.9%	.	.	.
GC13	9	103002508	G	T	INVS	Missense	p.W261L	9	132	6.8%	.	.	.
GC13	9	103055018	C	A	INVS	Missense	p.H501N	8	115	7.0%	.	.	.
GC13	9	103088600	C	A	TEX10	Missense	p.G658W	7	125	5.6%	.	O	.
GC13	9	103109354	G	T	TEX10	Missense	p.P175Q	8	144	5.6%	.	.	.
GC13	9	103109603	G	T	TEX10	Missense	p.P92Q	8	109	7.3%	.	.	.
GC13	9	104071540	G	T	PLPPR1	Nonsense	p.G145X	8	118	6.8%	.	.	.
GC13	9	104170397	G	T	ZNF189	Missense	p.W102L	9	161	5.6%	.	.	.
GC13	9	104171266	G	T	ZNF189	Nonsense	p.G392X	8	95	8.4%	.	.	.
GC13	9	104314720	C	A	RNF20	Missense	p.P529Q	10	109	9.2%	.	.	.
GC13	9	104314770	C	A	RNF20	Missense	p.Q546K	9	117	7.7%	.	.	.
GC13	9	104390604	G	T	GRIN3A	Missense	p.P811Q	9	105	8.6%	.	.	.
GC13	9	104433256	G	T	GRIN3A	Missense	p.R480S	6	91	6.6%	.	O	.
GC13	9	105757673	C	A	CYLC2	Missense	p.P4Q	6	72	8.3%	.	.	.
GC13	9	106889736	G	T	SMC2	Missense	p.R922L	8	91	8.8%	.	.	.

GC13	9	107298497	C	A	OR13C3	Missense	p.G200W	8	115	7.0%	.	O	.
GC13	9	107331834	C	A	OR13C8	Missense	p.P129Q	7	85	8.2%	.	.	.
GC13	9	107457306	G	T	OR13D1	Missense	p.G202W	6	78	7.7%	.	.	.
GC13	9	107624009	G	T	ABCA1	Missense	p.P165Q	8	108	7.4%	.	.	.
GC13	9	107646747	C	A	ABCA1	Missense	p.G88V	9	103	8.7%	.	.	.
GC13	9	108297381	G	T	FSD1L	Missense	p.W354L	8	126	6.3%	.	.	.
GC13	9	108308669	G	T	FSD1L	Missense	p.G467W	7	100	7.0%	.	.	.
GC13	9	108366571	G	T	FKTN	Missense	p.G149W	8	123	6.5%	.	.	.
GC13	9	108380368	G	T	FKTN	Missense	p.G347W	10	129	7.8%	.	.	.
GC13	9	109691577	C	A	ZNF462	Missense	p.P1795Q	8	98	8.2%	.	.	.
GC13	9	109691808	G	T	ZNF462	Missense	p.R1872L	9	135	6.7%	.	.	.
GC13	9	109771849	G	T	ZNF462	Missense	p.G1540W	8	134	6.0%	.	.	.
GC13	9	110249546	C	A	KLF4	Missense	p.G377W	9	129	7.0%	O	.	.
GC13	9	111624879	C	A	ACTL7A	Missense	p.H93N	11	86	12.8%	.	.	.
GC13	9	111641748	G	T	ELP1	Missense	p.H835N	10	110	9.1%	.	.	.
GC13	9	111662620	G	T	ELP1	Missense	p.H335N	7	76	9.2%	.	.	.
GC13	9	111706284	C	A	CTNNAL1	Missense	p.E633D	8	113	7.1%	.	.	.
GC13	9	111945064	G	T	EPB41L4B	Missense	p.P811Q	11	136	8.1%	.	.	.
GC13	9	112020520	C	A	EPB41L4B	Missense	p.R230L	6	59	10.2%	.	.	.
GC13	9	112694290	G	T	PALM2	Missense	p.G160W	6	47	12.8%	.	.	.
GC13	9	113166722	G	T	SVEP1	Missense	p.P3184Q	8	115	7.0%	.	.	.
GC13	9	113169675	C	A	SVEP1	Missense	p.M2735I	8	141	5.7%	.	.	.
GC13	9	113173389	G	T	SVEP1	Missense	p.P2201Q	5	53	9.4%	.	.	.
GC13	9	113449423	G	T	MUSK	Missense	p.R78L	7	110	6.4%	.	.	.
GC13	9	113550065	G	T	MUSK	Missense	p.R529M	8	95	8.4%	.	.	.
GC13	9	113563036	G	T	MUSK	Missense	p.W697L	8	102	7.8%	.	.	.
GC13	9	114135509	C	A	KIAA0368	Missense	p.R1604L	8	126	6.3%	.	.	.
GC13	9	114135596	C	A	KIAA0368	Missense	p.R1601L	8	136	5.9%	.	.	.
GC13	9	114178593	C	A	KIAA0368	Missense	p.G753W	6	83	7.2%	.	.	.
GC13	9	114332381	C	A	PTGR1	Missense	p.W290L	7	75	9.3%	.	.	.
GC13	9	114454293	G	T	C9orf84	Missense	p.R1219S	6	99	6.1%	.	.	.

GC13	9	114456095	G	T	C9orf84	Missense	p.P1047Q	6	88	6.8%	.	.	.
GC13	9	114864458	G	T	SUSD1	Missense	p.Q427K	6	59	10.2%	.	.	.
GC13	9	114904626	C	A	SUSD1	Missense	p.W227L	10	96	10.4%	.	.	.
GC13	9	114905777	G	T	SUSD1	Missense	p.P167Q	7	77	9.1%	.	.	.
GC13	9	115014983	G	T	PTBP3	Missense	p.P134Q	12	168	7.1%	.	.	.
GC13	9	115337162	G	T	KIAA1958	Missense	p.G268C	7	129	5.4%	.	.	.
GC13	9	115422066	G	T	KIAA1958	Missense	p.R623L	7	82	8.5%	.	.	.
GC13	9	115451827	G	T	INIP	Missense	p.Q37K	10	129	7.8%	.	.	.
GC13	9	115651925	C	A	SLC46A2	Missense	p.R346L	7	110	6.4%	.	.	.
GC13	9	115652127	G	T	SLC46A2	Missense	p.H279N	10	120	8.3%	.	.	.
GC13	9	115759561	C	A	ZNF883	Missense	p.G327W	8	116	6.9%	.	.	.
GC13	9	116059898	C	A	RNF183	Missense	p.W189C	9	125	7.2%	.	.	.
GC13	9	116085153	G	T	WDR31	Missense	p.L45M	8	114	7.0%	.	.	.
GC13	9	116130627	C	A	BSPRY	Missense	p.Q221K	7	64	10.9%	.	.	.
GC13	9	116136255	C	A	HDHD3	Missense	p.R127L	7	67	10.4%	.	.	.
GC13	9	116136270	C	A	HDHD3	Missense	p.R122M	7	66	10.6%	.	.	.
GC13	9	116172416	G	T	POLE3	Missense	p.P24Q	6	60	10.0%	.	.	.
GC13	9	116345883	G	T	RGS3	Missense	p.G52W	7	89	7.9%	.	.	.
GC13	9	116346614	G	T	RGS3	Missense	p.W295C	8	107	7.5%	.	.	.
GC13	9	116356307	G	T	RGS3	Missense	p.R36S	7	93	7.5%	.	.	.
GC13	9	116930117	G	T	COL27A1	Missense	p.L94F	7	84	8.3%	.	.	.
GC13	9	116931513	G	T	COL27A1	Missense	p.G560W	7	87	8.0%	.	.	.
GC13	9	116958266	G	T	COL27A1	Missense	p.G700W	6	88	6.8%	.	.	.
GC13	9	117062375	C	A	COL27A1	Missense	p.P1537Q	6	51	11.8%	.	.	.
GC13	9	117122051	G	T	AKNA	Missense	p.P653Q	9	90	10.0%	.	O	.
GC13	9	117797538	C	A	TNC	Missense	p.R1911L	6	70	8.6%	.	.	.
GC13	9	117822272	G	T	TNC	Missense	p.P1348Q	11	97	11.3%	.	.	.
GC13	9	118974130	G	T	PAPPA	Missense	p.G613C	10	113	8.8%	.	.	.
GC13	9	120474993	G	T	TLR4	Missense	p.R196L	10	152	6.6%	.	.	.
GC13	9	120475397	G	T	TLR4	Nonsense	p.G131X	8	107	7.5%	.	.	.
GC13	9	121929888	C	A	BRINP1	Missense	p.W587L	10	114	8.8%	.	.	.

GC13	9	123215846	C	A	CDK5RAP2	Missense	p.W894L	8	141	5.7%	.	.	.
GC13	9	123367581	G	T	MEGF9	Missense	p.H566N	8	130	6.2%	.	.	.
GC13	9	123533783	C	A	FBXW2	Missense	p.G307W	7	89	7.9%	.	O	.
GC13	9	123624951	C	A	PHF19	Missense	p.G140W	6	59	10.2%	.	.	.
GC13	9	123719563	C	A	C5	Missense	p.G1594W	10	171	5.8%	.	.	.
GC13	9	123768286	G	T	C5	Missense	p.L831M	9	144	6.3%	.	.	.
GC13	9	123903684	G	T	CNTRL	Missense	p.G285W	12	177	6.8%	.	.	.
GC13	9	123903780	C	A	CNTRL	Missense	p.Q317K	9	127	7.1%	.	.	.
GC13	9	123912674	G	T	CNTRL	Missense	p.M740I	9	85	10.6%	.	.	.
GC13	9	124074630	G	T	GSN	Missense	p.W227L	6	76	7.9%	.	.	.
GC13	9	124088886	G	T	GSN	Missense	p.G556W	5	71	7.0%	.	.	.
GC13	9	124091178	G	T	GSN	Missense	p.W642L	6	69	8.7%	.	.	.
GC13	9	124093681	G	T	GSN	Nonsense	p.G712X	8	83	9.6%	.	.	.
GC13	9	124528784	G	T	DAB2IP	Missense	p.R367L	10	103	9.7%	.	.	.
GC13	9	124528811	G	T	DAB2IP	Missense	p.W376L	7	99	7.1%	.	.	.
GC13	9	125075580	G	T	MRRF	Missense	p.R157L	8	127	6.3%	.	.	.
GC13	9	125145849	C	A	PTGS1	Missense	p.P227H	7	87	8.0%	.	.	.
GC13	9	125152544	G	T	PTGS1	Missense	p.R407L	5	57	8.8%	.	.	.
GC13	9	125282084	G	T	OR1J4	Missense	p.G222V	11	119	9.2%	.	.	.
GC13	9	125315620	G	T	OR1N2	Missense	p.G58W	8	123	6.5%	.	.	.
GC13	9	125315785	G	T	OR1N2	Missense	p.G113W	11	139	7.9%	.	.	.
GC13	9	125330468	C	A	OR1L8	Missense	p.G97W	10	100	10.0%	.	.	.
GC13	9	125330633	C	A	OR1L8	Missense	p.G42W	8	94	8.5%	.	.	.
GC13	9	125437411	G	T	OR1L3	Missense	p.M1I	13	143	9.1%	.	.	.
GC13	9	125582446	G	T	PDCL	Missense	p.P275Q	9	116	7.8%	.	.	.
GC13	9	125611926	C	A	RC3H2	Missense	p.G1186W	8	148	5.4%	.	.	.
GC13	9	125621349	C	A	RC3H2	Missense	p.G628C	7	75	9.3%	.	.	.
GC13	9	125642134	C	A	RC3H2	Missense	p.W371L	7	97	7.2%	.	.	.
GC13	9	125673305	C	A	ZBTB6	Missense	p.M349I	11	103	10.7%	.	.	.
GC13	9	125673547	C	A	ZBTB6	Missense	p.G269W	9	160	5.6%	.	.	.
GC13	9	125681375	C	A	ZBTB26	Missense	p.R280M	8	118	6.8%	.	.	.

GC13	9	125777851	G	T	RABGAP1	Missense	p.G527C	8	139	5.8%	.	.	.
GC13	9	125887936	C	A	STRBP	Missense	p.M639I	10	144	6.9%	.	.	.
GC13	9	126133374	G	T	CRB2	Missense	p.L651F	8	83	9.6%	.	.	.
GC13	9	126217019	C	A	DENND1A	Missense	p.R409L	6	77	7.8%	.	.	.
GC13	9	127265486	C	T	NR5A1	Missense	p.R39H	21	69	30.4%	.	.	.
GC13	9	127302370	G	T	NR6A1	Missense	p.P176T	6	68	8.8%	.	.	.
GC13	9	127572417	G	T	OLFML2A	Missense	p.R348L	6	97	6.2%	.	.	.
GC13	9	127572458	G	T	OLFML2A	Missense	p.G362W	8	105	7.6%	.	.	.
GC13	9	127764267	G	T	SCAI	Missense	p.P374H	8	92	8.7%	.	.	.
GC13	9	127920520	C	A	PPP6C	Missense	p.D105Y	7	60	11.7%	O	.	.
GC13	9	128001767	G	T	HSPA5	Missense	p.Q180K	9	92	9.8%	.	.	.
GC13	9	128002566	G	T	HSPA5	Missense	p.P126Q	6	78	7.7%	.	.	.
GC13	9	128305350	G	T	MAPKAP1	Missense	p.Q124K	13	139	9.4%	.	.	.
GC13	9	128419979	G	T	MAPKAP1	Missense	p.P150H	9	141	6.4%	.	.	.
GC13	9	129102865	G	T	MVB12B	Missense	p.G54W	7	76	9.2%	.	O	.
GC13	9	129595308	G	T	ZBTB43	Missense	p.G174C	8	98	8.2%	.	.	.
GC13	9	129595920	G	T	ZBTB43	Missense	p.G378W	10	138	7.2%	.	.	.
GC13	9	129642456	G	T	ZBTB34	Missense	p.G256W	7	80	8.8%	.	.	.
GC13	9	129642790	C	A	ZBTB34	Missense	p.P367Q	11	109	10.1%	.	.	.
GC13	9	129642906	G	T	ZBTB34	Missense	p.G406W	8	99	8.1%	.	.	.
GC13	9	130094595	G	T	GARNL3	Missense	p.G213W	7	99	7.1%	.	.	.
GC13	9	130104560	C	A	GARNL3	Missense	p.L400M	8	128	6.3%	.	.	.
GC13	9	130151355	G	T	GARNL3	Missense	p.M900I	7	83	8.4%	.	.	.
GC13	9	130230053	C	A	LRSAM1	Missense	p.P188Q	6	94	6.4%	.	.	.
GC13	9	130423471	C	A	STXBP1	Missense	p.P139Q	6	87	6.9%	.	.	.
GC13	9	130493047	G	T	TTC16	Missense	p.R649M	10	103	9.7%	.	.	.
GC13	9	130551560	C	A	CDK9	Missense	p.P286Q	7	92	7.6%	.	.	.
GC13	9	130648895	G	T	ST6GALNAC6	Missense	p.H295N	7	77	9.1%	.	.	.
GC13	9	130674659	G	T	ST6GALNAC4	Missense	p.R83S	5	68	7.4%	.	.	.
GC13	9	130687485	C	A	PIP5KL1	Missense	p.R70L	6	72	8.3%	.	.	.
GC13	9	131019418	C	A	GOLGA2	Missense	p.L979F	8	125	6.4%	.	.	.

GC13	9	131019506	C	A	GOLGA2	Missense	p.R950M	8	112	7.1%	.	.	.
GC13	9	131023457	G	T	GOLGA2	Missense	p.Q445K	7	79	8.9%	.	.	.
GC13	9	131028103	G	T	GOLGA2	Missense	p.L237M	8	91	8.8%	.	.	.
GC13	9	131118008	G	T	SLC27A4	Missense	p.L569F	7	83	8.4%	.	.	.
GC13	9	131186792	G	T	CERCAM	Missense	p.R144L	10	97	10.3%	.	.	.
GC13	9	131197961	G	T	CERCAM	Missense	p.R444L	4	23	17.4%	.	.	.
GC13	9	131198095	G	T	CERCAM	Missense	p.G489C	7	81	8.6%	.	.	.
GC13	9	131271207	G	T	GLE1	Missense	p.W51L	11	140	7.9%	.	.	.
GC13	9	131296113	G	T	GLE1	Missense	p.W510L	9	112	8.0%	.	.	.
GC13	9	131371550	C	A	SPTAN1	Missense	p.P1562H	7	96	7.3%	.	.	.
GC13	9	131394539	G	T	SPTAN1	Missense	p.W2274L	8	107	7.5%	.	.	.
GC13	9	131479202	G	T	PKN3	Missense	p.R662L	5	44	11.4%	.	.	.
GC13	9	131515072	C	A	ZER1	Missense	p.R268L	6	57	10.5%	.	.	.
GC13	9	131515534	G	T	ZER1	Missense	p.Q219K	7	63	11.1%	.	.	.
GC13	9	131591435	C	A	SPOUT1	Splicing	c.37-1G>T	12	103	11.7%	.	.	.
GC13	9	131605021	G	T	KYAT1	Missense	p.P38Q	6	69	8.7%	.	.	.
GC13	9	131670969	C	A	LRRC8A	Missense	p.P509Q	6	93	6.5%	.	.	.
GC13	9	131678502	G	T	LRRC8A	Missense	p.R762L	8	111	7.2%	.	.	.
GC13	9	131718676	C	A	NUP188	Missense	p.P70Q	8	133	6.0%	.	.	.
GC13	9	131757598	G	T	NUP188	Missense	p.K1052N	8	124	6.5%	.	.	.
GC13	9	131890313	C	A	PTPA	Missense	p.P96Q	9	115	7.8%	.	.	.
GC13	9	132084267	G	T	C9orf106	Missense	p.G59W	10	108	9.3%	.	.	.
GC13	9	132084535	G	T	C9orf106	Missense	p.W148L	6	72	8.3%	.	.	.
GC13	9	132382052	C	A	C9orf50	Missense	p.R189L	7	70	10.0%	.	.	.
GC13	9	132396387	G	T	NTMT1	Missense	p.G73W	7	100	7.0%	.	.	.
GC13	9	132400900	C	A	ASB6	Missense	p.G184W	7	58	12.1%	.	.	.
GC13	9	132689535	G	T	FNBP1	Missense	p.P243Q	9	137	6.6%	.	.	.
GC13	9	132741634	G	T	FNBP1	Missense	p.Q54K	7	99	7.1%	.	.	.
GC13	9	133499003	G	T	FUBP3	Missense	p.G294W	7	98	7.1%	.	.	.
GC13	9	133540176	G	T	PRDM12	Missense	p.G46W	6	70	8.6%	.	.	.
GC13	9	133589785	G	T	ABL1	Missense	p.G27W	7	94	7.4%	O	.	.

GC13	9	133753820	G	T	ABL1	Missense	p.W430L	8	127	6.3%	O	.	.
GC13	9	133760191	G	T	ABL1	Missense	p.K838N	9	107	8.4%	O	.	.
GC13	9	133914293	G	T	LAMC3	Missense	p.R340L	9	80	11.3%	.	.	.
GC13	9	133942439	G	T	LAMC3	Missense	p.G814W	6	86	7.0%	.	.	.
GC13	9	134004685	C	A	NUP214	Missense	p.P138Q	9	131	6.9%	.	.	.
GC13	9	134038431	G	T	NUP214	Missense	p.R855L	10	187	5.3%	.	.	.
GC13	9	134049695	G	T	NUP214	Missense	p.M1039I	6	54	11.1%	.	.	.
GC13	9	134067642	G	T	NUP214	Missense	p.G34W	14	153	9.2%	.	.	.
GC13	9	134070636	G	T	NUP214	Missense	p.G52W	16	134	11.9%	.	.	.
GC13	9	134072653	G	T	NUP214	Missense	p.G84C	6	65	9.2%	.	.	.
GC13	9	134072656	G	T	NUP214	Missense	p.G85W	6	67	9.0%	.	.	.
GC13	9	134072894	G	T	NUP214	Missense	p.R164L	7	114	6.1%	.	.	.
GC13	9	134073166	G	T	NUP214	Missense	p.G255W	8	133	6.0%	.	.	.
GC13	9	134074323	G	T	NUP214	Missense	p.Q640H	8	117	6.8%	.	.	.
GC13	9	134151463	C	A	FAM78A	Missense	p.R35L	9	110	8.2%	.	.	.
GC13	9	134308033	G	T	PRRC2B	Missense	p.G49W	8	126	6.3%	.	.	.
GC13	9	134351637	G	T	PRRC2B	Missense	p.W1374L	9	106	8.5%	.	.	.
GC13	9	134371199	C	A	PRRC2B	Missense	p.Q2210K	8	98	8.2%	.	.	.
GC13	9	134381561	G	T	POMT1	Missense	p.L7F	7	98	7.1%	.	.	.
GC13	9	134394800	G	T	POMT1	Missense	p.R387L	6	91	6.6%	.	.	.
GC13	9	134503411	G	T	RAPGEF1	Missense	p.R364S	6	76	7.9%	.	.	.
GC13	9	135073942	G	T	NTNG2	Missense	p.R268L	7	115	6.1%	.	.	.
GC13	9	135139786	G	T	SETX	Missense	p.P2625Q	8	92	8.7%	.	O	.
GC13	9	135140168	C	A	SETX	Nonsense	p.G2498X	7	81	8.6%	.	.	.
GC13	9	135202302	C	A	SETX	Missense	p.Q1561H	8	132	6.1%	.	.	.
GC13	9	135203084	G	T	SETX	Missense	p.Q1301K	10	153	6.5%	.	.	.
GC13	9	135203957	G	T	SETX	Missense	p.R1010S	10	128	7.8%	.	.	.
GC13	9	135203981	G	T	SETX	Missense	p.Q1002K	8	131	6.1%	.	.	.
GC13	9	135205538	G	T	SETX	Missense	p.Q483K	8	109	7.3%	.	.	.
GC13	9	135205713	C	A	SETX	Missense	p.L424F	11	161	6.8%	.	.	.
GC13	9	135218114	G	T	SETX	Missense	p.P154Q	10	152	6.6%	.	.	.

GC13	9	135251438	G	T	TTF1	Missense	p.P346Q	8	112	7.1%	.	.	.
GC13	9	135266132	G	T	TTF1	Missense	p.Q177K	10	172	5.8%	.	.	.
GC13	9	135458425	G	T	BARHL1	Missense	p.G81W	6	79	7.6%	.	.	.
GC13	9	135470264	G	T	DDX31	Missense	p.Q776K	7	98	7.1%	.	.	.
GC13	9	135487541	C	A	DDX31	Nonsense	p.G675X	7	108	6.5%	.	.	.
GC13	9	135507465	C	A	DDX31	Missense	p.R590L	7	110	6.4%	.	.	.
GC13	9	135546285	G	T	GTF3C4	Missense	p.Q100H	9	125	7.2%	.	.	.
GC13	9	135553611	G	T	GTF3C4	Missense	p.W202L	7	94	7.4%	.	.	.
GC13	9	135554802	C	A	GTF3C4	Missense	p.P599H	8	121	6.6%	.	.	.
GC13	9	135554967	C	A	GTF3C4	Missense	p.P654H	7	81	8.6%	.	.	.
GC13	9	135601139	G	T	AK8	Missense	p.P255Q	6	76	7.9%	.	.	.
GC13	9	135703427	C	A	AK8	Missense	p.G177W	7	80	8.8%	.	.	.
GC13	9	135772680	G	T	TSC1	Missense	p.Q905K	11	169	6.5%	O	.	.
GC13	9	135781031	G	T	TSC1	Missense	p.P594Q	8	106	7.5%	O	.	.
GC13	9	135977145	C	A	RALGDS	Missense	p.W684L	5	36	13.9%	.	.	.
GC13	9	136029121	C	A	GBGT1	Missense	p.R279L	8	84	9.5%	.	.	.
GC13	9	136029302	G	T	GBGT1	Missense	p.R219S	6	71	8.5%	.	O	.
GC13	9	136199436	G	T	SURF6	Missense	p.Q185K	7	97	7.2%	.	.	.
GC13	9	136199458	G	T	SURF6	Missense	p.Q178K	7	97	7.2%	.	.	.
GC13	9	136403493	G	T	ADAMTSL2	Missense	p.G86W	7	83	8.4%	.	.	.
GC13	9	136409616	C	A	ADAMTSL2	Missense	p.P236Q	5	62	8.1%	.	.	.
GC13	9	136501755	G	T	DBH	Missense	p.G88W	8	90	8.9%	.	.	.
GC13	9	136662918	C	A	VAV2	Missense	p.G279W	7	60	11.7%	.	.	.
GC13	9	136899943	C	A	BRD3	Missense	p.G649W	8	132	6.1%	.	.	.
GC13	9	136901285	C	A	BRD3	Missense	p.R602L	7	102	6.9%	.	.	.
GC13	9	137313586	G	T	RXRA	Missense	p.W185L	9	120	7.5%	.	.	.
GC13	9	138516108	C	A	GLT6D1	Missense	p.Q222H	8	90	8.9%	.	.	.
GC13	9	138703393	C	A	CAMSAP1	Missense	p.R1524M	10	135	7.4%	.	.	.
GC13	9	138714430	G	T	CAMSAP1	Missense	p.Q693K	8	100	8.0%	.	.	.
GC13	9	138837771	G	T	UBAC1	Missense	p.P206Q	10	80	12.5%	.	.	.
GC13	9	139100623	C	A	QSOX2	Missense	p.R683L	9	99	9.1%	.	O	.

GC13	9	139265835	G	T	CARD9	Missense	p.P88Q	8	77	10.4%	.	O	.
GC13	9	139333366	G	T	INPP5E	Missense	p.P169Q	4	35	11.4%	.	.	.
GC13	9	139370986	G	T	SEC16A	Missense	p.P361Q	8	117	6.8%	.	.	.
GC13	9	139391931	C	A	NOTCH1	Missense	p.R2087L	7	93	7.5%	O	.	.
GC13	9	139396799	G	T	NOTCH1	Missense	p.P1770H	8	85	9.4%	O	O	.
GC13	9	139400084	C	A	NOTCH1	Missense	p.G1422W	6	81	7.4%	O	.	.
GC13	9	139407916	G	T	NOTCH1	Missense	p.P761T	7	66	10.6%	O	O	.
GC13	9	139413909	G	T	NOTCH1	Missense	p.P284Q	5	54	9.3%	O	.	.
GC13	9	139636430	G	T	LCN10	Missense	p.Q54K	8	96	8.3%	.	.	.
GC13	9	139685875	C	A	TMEM141	Missense	p.P18Q	7	92	7.6%	.	.	.
GC13	9	139734871	C	A	RABL6	Missense	p.P697Q	5	45	11.1%	.	.	.
GC13	9	139741619	C	A	C9orf172	Missense	p.P918Q	6	74	8.1%	.	.	.
GC13	9	139743982	G	T	PHPT1	Missense	p.G34W	6	77	7.8%	.	.	.
GC13	9	139747909	G	T	MAMDC4	Missense	p.L109F	10	113	8.8%	.	.	.
GC13	9	139840965	G	T	C8G	Missense	p.R168L	7	116	6.0%	.	.	.
GC13	9	139909971	C	A	ABCA2	Missense	p.G1198W	10	92	10.9%	.	O	.
GC13	9	139944935	C	A	ENTPD2	Missense	p.G277V	7	68	10.3%	.	.	.
GC13	9	139973828	G	T	UAP1L1	Missense	p.G322W	7	117	6.0%	.	.	.
GC13	9	139990711	G	T	MAN1B1	Missense	p.R163L	6	79	7.6%	.	.	.
GC13	9	140070198	G	T	ANAPC2	Missense	p.P661Q	6	55	10.9%	.	.	.
GC13	9	140082083	G	T	ANAPC2	Missense	p.P197Q	8	96	8.3%	.	.	.
GC13	9	140109135	G	T	NDOR1	Missense	p.R245L	5	56	8.9%	.	.	.
GC13	9	140147339	G	T	STPG3	Missense	p.G240W	7	77	9.1%	.	.	.
GC13	9	140147755	G	T	STPG3	Missense	p.R334L	8	116	6.9%	.	.	.
GC13	9	140332506	G	T	ENTPD8	Missense	p.H53N	7	94	7.4%	.	.	.
GC13	9	140477532	C	A	ZMYND19	Missense	p.R148L	6	96	6.3%	.	.	.
GC13	9	140508099	G	T	ARRDC1	Missense	p.G105W	7	135	5.2%	.	.	.
GC13	9	140637878	G	T	EHMT1	Missense	p.M293I	11	109	10.1%	.	.	.
GC13	9	140674110	C	A	EHMT1	Missense	p.P739Q	7	80	8.8%	.	.	.
GC13	9	140954153	G	T	CACNA1B	Missense	p.L1544F	8	108	7.4%	.	.	.
GC13	10	1041879	C	A	GTPBP4	Missense	p.P77Q	6	90	6.7%	.	.	.

GC13	10	1065735	G	T	IDI2	Missense	p.H136N	7	98	7.1%	.	.	.
GC13	10	5442858	C	A	TUBAL3	Missense	p.G26W	8	102	7.8%	.	.	.
GC13	10	5468671	G	T	NET1	Missense	p.W61L	7	98	7.1%	.	.	.
GC13	10	5784191	C	A	FAM208B	Missense	p.P739Q	10	100	10.0%	.	.	.
GC13	10	6063507	C	A	IL2RA	Missense	p.G173W	6	65	9.2%	.	.	.
GC13	10	6255591	G	T	PFKFB3	Missense	p.G8W	7	84	8.3%	.	.	.
GC13	10	6257195	G	T	PFKFB3	Missense	p.G52W	5	42	11.9%	.	.	.
GC13	10	6553088	G	T	PRKCQ	Missense	p.H63N	6	54	11.1%	.	.	.
GC13	10	7605099	G	T	ITIH5	Missense	p.H712N	7	69	10.1%	.	.	.
GC13	10	7765511	G	T	ITIH2	Missense	p.W322L	8	65	12.3%	.	.	.
GC13	10	7820911	G	T	KIN	Missense	p.R150S	7	134	5.2%	.	.	.
GC13	10	7820958	G	T	KIN	Missense	p.P134Q	8	108	7.4%	.	.	.
GC13	10	7844366	G	T	ATP5C1	Missense	p.M210I	7	53	13.2%	.	.	.
GC13	10	7866333	G	T	TAF3	Missense	p.M73I	7	91	7.7%	.	.	.
GC13	10	11291164	G	T	CELF2	Missense	p.A17S	11	100	11.0%	.	.	.
GC13	10	11911503	G	T	PROSER2	Missense	p.G136W	6	32	18.8%	.	.	.
GC13	10	12288212	G	T	CDC123	Missense	p.W261L	8	104	7.7%	.	.	.
GC13	10	13040509	C	A	CCDC3	Missense	p.M126I	7	89	7.9%	.	.	.
GC13	10	13647731	C	A	PRPF18	Missense	p.P117Q	7	94	7.4%	.	.	.
GC13	10	13712485	C	A	FRMD4A	Missense	p.G123V	7	52	13.5%	.	.	.
GC13	10	13838535	G	T	FRMD4A	Missense	p.P103H	8	96	8.3%	.	.	.
GC13	10	16877147	C	A	CUBN	Nonsense	p.G3410X	7	96	7.3%	.	.	.
GC13	10	17271885	G	T	VIM	Missense	p.R155L	6	74	8.1%	.	.	.
GC13	10	17646044	C	A	HACD1	Missense	p.W87L	6	58	10.3%	.	.	.
GC13	10	17730156	C	A	STAM	Missense	p.P46Q	7	94	7.4%	.	.	.
GC13	10	17756720	G	T	STAM	Nonsense	p.G425X	8	89	9.0%	.	.	.
GC13	10	18289753	G	T	SLC39A12	Missense	p.M452I	7	76	9.2%	.	.	.
GC13	10	21785733	C	A	CASC10	Missense	p.R4L	9	117	7.7%	.	.	.
GC13	10	21804177	C	A	SKIDA1	Missense	p.G859W	7	100	7.0%	.	.	.
GC13	10	22055239	C	A	DNAJC1	Splicing	c.1099-1G>T	8	92	8.7%	.	.	.
GC13	10	22705604	C	A	SPAG6	Missense	p.P506Q	9	87	10.3%	.	.	.

GC13	10	22896890	C	A	PIP4K2A	Missense	p.R43L	6	80	7.5%	.	.	.
GC13	10	23270364	C	A	ARMC3	Missense	p.Q75K	8	108	7.4%	.	.	.
GC13	10	23295877	G	T	ARMC3	Missense	p.R336L	8	94	8.5%	.	.	.
GC13	10	23481490	G	T	PTF1A	Missense	p.G11W	7	63	11.1%	.	.	.
GC13	10	24816916	G	T	KIAA1217	Missense	p.G667W	7	78	9.0%	.	.	.
GC13	10	25279532	C	A	ENKUR	Missense	p.G90C	6	53	11.3%	.	.	.
GC13	10	25887581	G	T	GPR158	Missense	p.W1009L	7	81	8.6%	.	.	.
GC13	10	26785255	C	A	APBB1IP	Missense	p.P32H	7	87	8.0%	.	.	.
GC13	10	26856326	C	A	APBB1IP	Missense	p.P637Q	6	61	9.8%	.	.	.
GC13	10	27066058	G	T	ABI1	Missense	p.P133H	7	98	7.1%	.	.	.
GC13	10	28879696	C	A	WAC	Missense	p.P182Q	11	121	9.1%	.	.	.
GC13	10	29788078	G	T	SVIL	Missense	p.Q785K	8	77	10.4%	.	.	.
GC13	10	29821045	C	A	SVIL	Missense	p.R632L	6	43	14.0%	.	.	.
GC13	10	29840112	C	A	SVIL	Missense	p.G81C	8	87	9.2%	.	.	.
GC13	10	30315489	C	A	JCAD	Missense	p.L1196F	5	44	11.4%	.	.	.
GC13	10	30316958	C	A	JCAD	Missense	p.G707C	6	60	10.0%	.	.	.
GC13	10	30318506	G	T	JCAD	Missense	p.Q191K	9	121	7.4%	.	.	.
GC13	10	30318628	C	A	JCAD	Missense	p.R150M	7	99	7.1%	.	.	.
GC13	10	31815993	C	A	ZEB1	Missense	p.P985Q	8	124	6.5%	.	.	.
GC13	10	33015746	G	T	CCDC7	Missense	p.R885M	8	113	7.1%	.	.	.
GC13	10	33103379	C	A	CCDC7	Missense	p.P1060H	6	57	10.5%	.	O	.
GC13	10	33502592	G	T	NRP1	Missense	p.Q446K	6	90	6.7%	.	.	.
GC13	10	34400428	C	A	PARD3	Missense	p.G1135V	6	56	10.7%	.	.	.
GC13	10	34400459	G	T	PARD3	Missense	p.Q1125K	6	56	10.7%	.	.	.
GC13	10	34739351	G	T	PARD3	Missense	p.P203Q	6	76	7.9%	.	.	.
GC13	10	37431099	C	A	ANKRD30A	Missense	p.P369H	8	108	7.4%	.	.	.
GC13	10	38121178	G	T	ZNF248	Missense	p.Q369K	8	98	8.2%	.	O	.
GC13	10	38241413	C	A	ZNF25	Missense	p.G338V	6	55	10.9%	.	.	.
GC13	10	38306297	C	A	ZNF33A	Missense	p.P85Q	10	111	9.0%	.	.	.
GC13	10	38343809	G	T	ZNF33A	Missense	p.G259W	10	118	8.5%	.	.	.
GC13	10	38344727	G	T	ZNF33A	Missense	p.G565W	6	51	11.8%	.	.	.

GC13	10	38406546	C	A	ZNF37A	Missense	p.P156H	12	96	12.5%	.	.	.
GC13	10	43127394	G	T	ZNF33B	Missense	p.P85Q	8	104	7.7%	.	.	.
GC13	10	43607633	C	A	RET	Missense	p.P537T	6	40	15.0%	O	.	.
GC13	10	43612045	G	T	RET	Missense	p.W717L	7	66	10.6%	O	.	.
GC13	10	43882552	C	A	HNRNPF	Missense	p.G261W	5	68	7.4%	.	.	.
GC13	10	44111981	G	T	ZNF485	Missense	p.G73W	7	89	7.9%	.	.	.
GC13	10	44112065	G	T	ZNF485	Missense	p.G101W	9	104	8.7%	.	.	.
GC13	10	44112233	G	T	ZNF485	Missense	p.G157W	7	88	8.0%	.	.	.
GC13	10	44139995	G	T	ZNF32	Missense	p.H109N	9	86	10.5%	.	.	.
GC13	10	45799231	C	A	OR13A1	Missense	p.G214C	7	96	7.3%	.	.	.
GC13	10	46121548	C	A	ZFAND4	Missense	p.G575W	6	78	7.7%	.	.	.
GC13	10	46122421	C	A	ZFAND4	Missense	p.G284W	8	104	7.7%	.	.	.
GC13	10	46143814	G	T	ZFAND4	Missense	p.P166Q	6	91	6.6%	.	.	.
GC13	10	47087681	G	T	NPY4R	Missense	p.G300W	9	163	5.5%	.	O	.
GC13	10	48385920	G	T	RBP3	Missense	p.Q1058K	5	50	10.0%	.	.	.
GC13	10	50364200	C	A	C10orf128	Nonsense	p.G123X	7	98	7.1%	.	.	.
GC13	10	50533661	G	T	C10orf71	Missense	p.W1024L	10	106	9.4%	.	.	.
GC13	10	50534222	C	A	C10orf71	Missense	p.P1211Q	5	51	9.8%	.	.	.
GC13	10	52595930	G	T	A1CF	Missense	p.P170T	7	97	7.2%	.	.	.
GC13	10	52596007	G	T	A1CF	Missense	p.P144Q	8	106	7.5%	.	.	.
GC13	10	60124601	G	T	UBE2D1	Missense	p.R52M	8	124	6.5%	.	.	.
GC13	10	61829467	C	A	ANK3	Missense	p.M3724I	8	107	7.5%	.	.	.
GC13	10	61830483	C	A	ANK3	Missense	p.G3386W	10	81	12.3%	.	.	.
GC13	10	61833788	C	A	ANK3	Missense	p.R2284L	7	107	6.5%	.	.	.
GC13	10	61835138	G	T	ANK3	Missense	p.P1834Q	7	88	8.0%	.	.	.
GC13	10	61835226	C	A	ANK3	Missense	p.G1805W	8	111	7.2%	.	.	.
GC13	10	61958259	C	A	ANK3	Missense	p.G493W	7	96	7.3%	.	.	.
GC13	10	61967827	C	A	ANK3	Missense	p.L370F	6	40	15.0%	.	.	.
GC13	10	62547966	C	A	CDK1	Missense	p.P156H	9	124	7.3%	.	.	.
GC13	10	62551647	G	T	CDK1	Splicing	c.490-1G>T;NM_033379	6	47	12.8%	.	O	.
GC13	10	62648260	G	T	RHOBTB1	Missense	p.P269H	7	99	7.1%	.	.	.

GC13	10	63816921	C	A	ARID5B	Missense	p.H55N	7	74	9.5%	.	.	.
GC13	10	63851422	C	A	ARID5B	Missense	p.Q491K	7	94	7.4%	.	.	.
GC13	10	63852329	C	A	ARID5B	Missense	p.P793Q	9	76	11.8%	.	.	.
GC13	10	64968074	C	A	JMJD1C	Missense	p.G900C	6	87	6.9%	.	.	.
GC13	10	68280449	C	A	CTNNA3	Missense	p.W486L	8	123	6.5%	.	.	.
GC13	10	68687507	C	A	LRRTM3	Missense	p.P278Q	7	98	7.1%	.	.	.
GC13	10	69676334	C	A	SIRT1	Missense	p.P440Q	10	121	8.3%	.	.	.
GC13	10	69966595	G	T	MYPN	Missense	p.W1243L	7	66	10.6%	.	.	.
GC13	10	70174817	C	A	DNA2	Missense	p.L1054F	8	106	7.5%	.	.	.
GC13	10	70190304	C	A	DNA2	Missense	p.L699F	8	125	6.4%	.	.	.
GC13	10	70332532	C	A	TET1	Missense	p.P146H	8	120	6.7%	.	.	.
GC13	10	70405846	G	T	TET1	Missense	p.K1120N	9	102	8.8%	.	.	.
GC13	10	70506928	G	T	CCAR1	Missense	p.G162W	8	68	11.8%	.	.	.
GC13	10	70652357	G	T	STOX1	Missense	p.W159L	8	90	8.9%	.	.	.
GC13	10	70893363	G	T	VPS26A	Missense	p.K34N	7	82	8.5%	.	.	.
GC13	10	70968579	G	T	SUPV3L1	Missense	p.G388W	7	95	7.4%	.	.	.
GC13	10	71008179	G	T	HKDC1	Splicing	c.1266-1G>T	7	65	10.8%	.	.	.
GC13	10	72195373	G	T	NODAL	Missense	p.P54Q	6	79	7.6%	.	.	.
GC13	10	72358494	C	A	PRF1	Missense	p.W328L	7	70	10.0%	.	.	.
GC13	10	72462146	G	T	ADAMTS14	Missense	p.G201W	6	67	9.0%	.	.	.
GC13	10	73501668	C	A	CDH23	Missense	p.P1612Q	5	42	11.9%	.	.	.
GC13	10	73559336	G	T	CDH23	Nonsense	p.E198X	6	78	7.7%	.	.	.
GC13	10	73827445	C	A	SPOCK2	Missense	p.R212L	6	44	13.6%	.	.	.
GC13	10	73921405	G	T	ASCC1	Missense	p.Q173K	8	112	7.1%	.	.	.
GC13	10	74594123	C	A	MCU	Missense	p.Q53K	7	63	11.1%	.	.	.
GC13	10	74684198	C	A	OIT3	Missense	p.P388Q	8	100	8.0%	.	.	.
GC13	10	74684246	G	T	OIT3	Missense	p.R404L	11	104	10.6%	.	.	.
GC13	10	74831936	C	A	P4HA1	Missense	p.W59L	7	98	7.1%	.	.	.
GC13	10	74906091	C	A	ECD	Missense	p.R314L	7	89	7.9%	.	.	.
GC13	10	74916147	C	A	ECD	Missense	p.W159L	6	61	9.8%	.	.	.
GC13	10	75011670	G	T	MRPS16	Missense	p.P42H	8	90	8.9%	.	O	.

GC13	10	75276606	C	A	USP54	Missense	p.G1136V	7	88	8.0%	.	.	.
GC13	10	75301452	G	T	USP54	Missense	p.P206Q	9	139	6.5%	.	.	.
GC13	10	75548485	C	A	ZSWIM8	Missense	p.P89Q	6	62	9.7%	.	.	.
GC13	10	75563775	C	A	NDST2	Splicing	c.1844-1G>T	7	66	10.6%	.	.	.
GC13	10	75566868	G	A	NDST2	Missense	p.T364I	20	61	32.8%	.	.	.
GC13	10	76780901	G	T	KAT6B	Missense	p.R777L	6	51	11.8%	.	.	.
GC13	10	76978932	A	G	VDAC2	Missense	p.T88A	61	100	61.0%	.	.	.
GC13	10	78674754	C	A	KCNMA1	Missense	p.G878W	7	65	10.8%	.	O	.
GC13	10	79576809	G	T	DLG5	Missense	p.P1277Q	6	50	12.0%	.	.	.
GC13	10	79795321	G	T	RPS24	Missense	p.R41L	5	34	14.7%	.	.	.
GC13	10	81923162	C	A	ANXA11	Splicing	c.1030-1G>T;NM_001278407	7	71	9.9%	.	.	.
GC13	10	85936235	G	T	C10orf99	Splicing	c.71-1G>T	8	87	9.2%	.	.	.
GC13	10	85978834	G	T	CDHR1	Splicing	c.2041-1G>T	7	72	9.7%	.	.	.
GC13	10	88260360	G	T	WAPL	Missense	p.Q214K	8	85	9.4%	.	.	.
GC13	10	88459034	G	T	LDB3	Splicing	c.897-1G>T;NM_001171611	8	96	8.3%	.	.	.
GC13	10	88476287	G	T	LDB3	Missense	p.G369W	5	46	10.9%	.	.	.
GC13	10	88822499	C	A	GLUD1	Missense	p.G146W	7	76	9.2%	.	.	.
GC13	10	89473816	C	A	PAPSS2	Missense	p.R133S	6	82	7.3%	.	.	.
GC13	10	89503232	C	A	PAPSS2	Missense	p.P437Q	8	74	10.8%	.	.	.
GC13	10	90438371	G	T	LIPF	Missense	p.W344L	7	82	8.5%	.	.	.
GC13	10	90503041	G	T	LIPK	Missense	p.W308L	11	73	15.1%	.	.	.
GC13	10	90673059	G	T	STAMBPL1	Missense	p.G208W	9	126	7.1%	.	.	.
GC13	10	90762831	C	A	FAS	Missense	p.Q26K	6	81	7.4%	O	.	.
GC13	10	90770527	G	T	FAS	Missense	p.G175W	9	115	7.8%	O	.	.
GC13	10	91099526	G	T	IFIT3	Missense	p.G372W	9	118	7.6%	.	.	.
GC13	10	91198495	G	T	SLC16A12	Missense	p.S298R	8	74	10.8%	.	.	.
GC13	10	92509305	C	A	HTR7	Missense	p.G196W	7	60	11.7%	.	O	.
GC13	10	92672720	G	A	ANKRD1	Missense	p.P288L	17	87	19.5%	.	.	.
GC13	10	93390287	C	A	PPP1R3C	Missense	p.L117F	7	79	8.9%	.	.	.
GC13	10	93593750	G	T	TNKS2	Missense	p.M472I	7	65	10.8%	.	.	.

GC13	10	93702310	C	A	BTAF1	Missense	p.Q129K	7	79	8.9%	.	.	.
GC13	10	94109544	G	T	MARCH5	Missense	p.G224C	6	57	10.5%	.	.	.
GC13	10	94223509	G	T	IDE	Missense	p.Q359K	7	97	7.2%	.	.	.
GC13	10	94294478	C	A	IDE	Missense	p.L116F	7	82	8.5%	.	.	.
GC13	10	94669237	G	T	EXOC6	Missense	p.W171L	10	114	8.8%	.	.	.
GC13	10	95076549	G	T	MYOF	Missense	p.Q1861K	7	96	7.3%	.	.	.
GC13	10	95079675	G	T	MYOF	Missense	p.P1838Q	8	91	8.8%	.	.	.
GC13	10	95111459	G	T	MYOF	Missense	p.P1196H	7	74	9.5%	.	.	.
GC13	10	95134627	G	T	MYOF	Missense	p.Q719K	8	95	8.4%	.	.	.
GC13	10	95157179	G	T	MYOF	Missense	p.P419Q	7	88	8.0%	.	.	.
GC13	10	95279564	G	T	CEP55	Missense	p.L397F	7	50	14.0%	.	.	.
GC13	10	95347194	G	T	FFAR4	Missense	p.W305L	9	119	7.6%	.	.	.
GC13	10	95347299	C	A	FFAR4	Missense	p.P340Q	7	95	7.4%	.	.	.
GC13	10	95372568	G	T	PDE6C	Missense	p.R29L	6	82	7.3%	.	.	.
GC13	10	95557512	G	T	LGI1	Missense	p.K494N	9	129	7.0%	.	.	.
GC13	10	96012254	G	T	PLCE1	Missense	p.R785M	7	58	12.1%	.	.	.
GC13	10	96117005	G	T	NOC3L	Missense	p.P145Q	8	87	9.2%	.	.	.
GC13	10	96256920	G	T	TBC1D12	Missense	p.W468L	8	114	7.0%	.	.	.
GC13	10	96260033	C	A	TBC1D12	Missense	p.R490S	5	68	7.4%	.	.	.
GC13	10	96352013	G	T	HELLS	Missense	p.G501W	8	140	5.7%	.	.	.
GC13	10	96534948	C	A	CYP2C19	Missense	p.P101Q	10	92	10.9%	.	.	.
GC13	10	96741127	G	T	CYP2C9	Missense	p.K383N	7	100	7.0%	.	.	.
GC13	10	97096601	G	T	SORBS1	Missense	p.Q1106K	7	85	8.2%	.	.	.
GC13	10	97135809	C	A	SORBS1	Missense	p.R352L	7	108	6.5%	.	.	.
GC13	10	98078275	C	A	DNTT	Missense	p.Q124K	7	95	7.4%	.	.	.
GC13	10	98097962	C	A	DNTT	Missense	p.P503Q	8	119	6.7%	.	.	.
GC13	10	98388211	G	T	PIK3AP1	Missense	p.P472H	7	95	7.4%	.	.	.
GC13	10	98408525	G	T	PIK3AP1	Missense	p.P359Q	5	44	11.4%	.	.	.
GC13	10	98742144	G	T	LCOR	Missense	p.G643W	7	73	9.6%	.	.	.
GC13	10	98742646	C	A	LCOR	Missense	p.P810H	8	112	7.1%	.	.	.
GC13	10	98802735	G	T	SLIT1	Missense	p.P696Q	5	54	9.3%	.	.	.

GC13	10	99016089	G	T	ARHGAP19	Missense	p.Q296K	8	133	6.0%	.	.	.
GC13	10	99211530	G	T	ZDHHC16	Missense	p.R33L	7	50	14.0%	.	.	.
GC13	10	99213398	G	T	ZDHHC16	Missense	p.R184L	9	126	7.1%	.	.	.
GC13	10	99330045	C	A	UBTD1	Missense	p.P150Q	5	50	10.0%	.	O	.
GC13	10	99969480	G	T	R3HCC1L	Missense	p.G537W	7	92	7.6%	.	O	.
GC13	10	101090195	G	T	CNNM1	Missense	p.G351W	8	50	16.0%	.	.	.
GC13	10	101163380	C	A	GOT1	Missense	p.G269W	9	70	12.9%	.	.	.
GC13	10	101371063	C	A	SLC25A28	Missense	p.R213L	5	35	14.3%	.	.	.
GC13	10	101563990	C	A	ABCC2	Missense	p.P475Q	7	91	7.7%	.	.	.
GC13	10	101571329	G	T	ABCC2	Missense	p.W646L	6	75	8.0%	.	.	.
GC13	10	101643789	G	T	DNMBP	Missense	p.R958S	9	101	8.9%	.	.	.
GC13	10	101689818	G	T	DNMBP	Missense	p.H235N	9	111	8.1%	.	.	.
GC13	10	101823415	G	T	CPN1	Missense	p.P276Q	8	69	11.6%	.	.	.
GC13	10	101996666	G	T	CWF19L1	Missense	p.Q302K	8	112	7.1%	.	.	.
GC13	10	102020765	G	T	CWF19L1	Missense	p.Q49K	9	95	9.5%	.	.	.
GC13	10	102058400	C	A	PKD2L1	Missense	p.R170L	8	94	8.5%	.	.	.
GC13	10	102089764	C	A	PKD2L1	Missense	p.G33W	6	68	8.8%	.	.	.
GC13	10	102240761	G	T	WNT8B	Missense	p.R83L	7	132	5.3%	.	.	.
GC13	10	102267738	G	T	SEC31B	Missense	p.P189H	7	94	7.4%	.	.	.
GC13	10	102743202	C	A	SEMA4G	Missense	p.R616S	6	48	12.5%	.	.	.
GC13	10	102749460	C	A	TWNK	Missense	p.Q435K	7	65	10.8%	.	.	.
GC13	10	102762413	G	T	LZTS2	Missense	p.G40W	6	33	18.2%	.	O	.
GC13	10	102763862	G	T	LZTS2	Missense	p.R336L	4	27	14.8%	.	.	.
GC13	10	102822755	G	T	KAZALD1	Missense	p.G136W	8	65	12.3%	.	.	.
GC13	10	103291028	G	T	BTRC	Missense	p.G234W	6	97	6.2%	.	O	.
GC13	10	103294543	G	T	BTRC	Missense	p.W382L	9	93	9.7%	.	.	.
GC13	10	103294557	C	A	BTRC	Missense	p.P387T	7	90	7.8%	.	.	.
GC13	10	103354442	C	A	DPCD	Missense	p.P31Q	7	78	9.0%	.	.	.
GC13	10	103368643	G	T	DPCD	Missense	p.W166L	7	91	7.7%	.	.	.
GC13	10	103547174	G	T	MGEA5	Missense	p.P801Q	10	133	7.5%	.	.	.
GC13	10	103557898	C	A	MGEA5	Missense	p.R555L	6	86	7.0%	.	O	.

GC13	10	103563587	G	T	MGEA5	Missense	p.P314Q	8	109	7.3%	.	.	.
GC13	10	103649234	G	T	C10orf76	Missense	p.H599N	8	114	7.0%	.	.	.
GC13	10	103735007	C	A	C10orf76	Missense	p.R486L	8	87	9.2%	.	.	.
GC13	10	103761808	G	T	C10orf76	Missense	p.P357Q	6	78	7.7%	.	.	.
GC13	10	103826529	G	T	HPS6	Missense	p.R433L	7	79	8.9%	.	.	.
GC13	10	103899049	G	T	PPRC1	Missense	p.G262W	6	80	7.5%	.	.	.
GC13	10	103918976	G	T	NOLC1	Missense	p.G212C	8	93	8.6%	.	.	.
GC13	10	104136703	C	A	GBF1	Missense	p.Q1434K	7	77	9.1%	.	.	.
GC13	10	104136748	C	A	GBF1	Missense	p.R1449S	6	94	6.4%	.	.	.
GC13	10	104241913	C	A	ACTR1A	Missense	p.R257L	4	23	17.4%	.	.	.
GC13	10	104596915	C	A	CYP17A1	Missense	p.M68I	10	94	10.6%	.	.	.
GC13	10	104632231	C	A	AS3MT	Missense	p.P66H	7	98	7.1%	.	.	.
GC13	10	104679469	G	T	CNNM2	Missense	p.R411L	6	71	8.5%	.	.	.
GC13	10	105138125	C	A	TAF5	Missense	p.R311S	7	76	9.2%	.	.	.
GC13	10	105147277	G	T	TAF5	Splicing	c.2008-1G>T	7	98	7.1%	.	.	.
GC13	10	105147311	G	T	TAF5	Missense	p.G626W	9	94	9.6%	.	.	.
GC13	10	105330637	G	T	NEURL1	Missense	p.G32W	7	58	12.1%	.	.	.
GC13	10	105762518	C	A	SLK	Missense	p.Q528K	10	110	9.1%	.	.	.
GC13	10	106035008	C	A	GSTO2	Missense	p.P20Q	7	92	7.6%	.	O	.
GC13	10	106130692	G	T	CFAP58	Missense	p.G324W	7	81	8.6%	.	.	.
GC13	10	106214201	G	T	CFAP58	Missense	p.M844I	7	87	8.0%	.	O	.
GC13	10	106960877	G	T	SORCS3	Splicing	c.2128-1G>T	8	79	10.1%	.	.	.
GC13	10	112055123	G	T	SMNDC1	Missense	p.Q148K	7	81	8.6%	.	.	.
GC13	10	112269834	C	A	DUSP5	Missense	p.R269S	5	37	13.5%	.	.	.
GC13	10	112541009	G	T	RBM20	Missense	p.L214F	6	71	8.5%	.	.	.
GC13	10	112724409	G	T	SHOC2	Missense	p.R98L	7	88	8.0%	.	.	.
GC13	10	112838383	G	T	ADRA2A	Missense	p.W210L	7	66	10.6%	.	.	.
GC13	10	114164462	G	T	ACSL5	Splicing	c.331-1G>T;NM_016234	9	114	7.9%	.	.	.
GC13	10	114172974	G	T	ACSL5	Missense	p.G387V	8	91	8.8%	.	.	.
GC13	10	114711340	G	T	TCF7L2	Nonsense	p.G119X	5	34	14.7%	.	.	.
GC13	10	115612496	G	T	DCLRE1A	Missense	p.P149Q	7	71	9.9%	.	.	.

GC13	10	115966034	G	T	TDRD1	Missense	p.M443I	9	123	7.3%	.	.	.
GC13	10	115970459	C	A	TDRD1	Missense	p.H502N	8	118	6.8%	.	.	.
GC13	10	116050103	G	T	VWA2	Nonsense	p.E743X	7	47	14.9%	.	.	.
GC13	10	116073694	G	T	AFAP1L2	Missense	p.P243Q	8	81	9.9%	.	.	.
GC13	10	116247718	C	A	ABLIM1	Missense	p.R31L	7	96	7.3%	.	.	.
GC13	10	116595325	G	T	FAM160B1	Missense	p.L108F	7	80	8.8%	.	.	.
GC13	10	116614987	G	T	FAM160B1	Missense	p.W612L	9	126	7.1%	.	.	.
GC13	10	116735022	C	A	TRUB1	Missense	p.P312T	8	104	7.7%	.	.	.
GC13	10	116919911	G	T	ATRNL1	Missense	p.G314W	7	106	6.6%	.	.	.
GC13	10	117059596	G	T	ATRNL1	Missense	p.W823L	6	72	8.3%	.	.	.
GC13	10	117185792	C	A	ATRNL1	Missense	p.P1101Q	8	102	7.8%	.	.	.
GC13	10	118220722	C	A	PNLIPRP3	Missense	p.P243Q	9	83	10.8%	.	.	.
GC13	10	118306854	C	A	PNLIP	Missense	p.P32Q	7	74	9.5%	.	.	.
GC13	10	118368594	G	T	PNLIPRP1	Missense	p.R457L	9	79	11.4%	.	.	.
GC13	10	118969566	G	T	KCNK18	Missense	p.W304L	11	101	10.9%	.	.	.
GC13	10	119003628	G	T	SLC18A2	Missense	p.G90W	6	96	6.3%	.	.	.
GC13	10	119805635	G	T	RAB11FIP2	Missense	p.H14N	9	84	10.7%	.	.	.
GC13	10	120832497	C	A	EIF3A	Missense	p.W149L	7	88	8.0%	.	.	.
GC13	10	121203226	C	A	GRK5	Missense	p.H410N	6	27	22.2%	.	.	.
GC13	10	121338316	C	A	TIAL1	Missense	p.G177C	6	98	6.1%	.	.	.
GC13	10	121429421	G	T	BAG3	Missense	p.R80M	10	96	10.4%	.	.	.
GC13	10	121432041	G	T	BAG3	Missense	p.R261L	7	83	8.4%	.	.	.
GC13	10	121551553	C	A	INPP5F	Missense	p.P206Q	7	73	9.6%	.	.	.
GC13	10	121556378	C	A	INPP5F	Missense	p.P274Q	5	35	14.3%	.	.	.
GC13	10	121564967	G	T	INPP5F	Missense	p.W438L	8	96	8.3%	.	.	.
GC13	10	121693197	G	T	SEC23IP	Splicing	c.2922-1G>T	8	82	9.8%	.	.	.
GC13	10	123256051	G	T	FGFR2	Missense	p.Q508K	13	100	13.0%	O	.	.
GC13	10	123278195	C	A	FGFR2	Splicing	c.1087+1G>T; NM_001144919	15	62	24.2%	O	.	.
GC13	10	123662006	G	T	ATE1	Missense	p.P142Q	7	99	7.1%	.	.	.
GC13	10	123844706	G	T	TACC2	Missense	p.L897F	8	99	8.1%	.	.	.
GC13	10	123970587	C	A	TACC2	Missense	p.P294Q	5	56	8.9%	.	.	.

GC13	10	124089024	G	T	BTBD16	Missense	p.R315L	6	85	7.1%	.	.	.
GC13	10	124189152	G	T	PLEKHA1	Missense	p.G305C	5	59	8.5%	.	.	.
GC13	10	124755656	G	T	IKZF5	Missense	p.P57Q	7	78	9.0%	.	.	.
GC13	10	126097450	G	T	OAT	Missense	p.P95H	8	117	6.8%	.	.	.
GC13	10	126715376	G	T	CTBP2	Missense	p.P318Q	9	65	13.8%	.	.	.
GC13	10	126715535	G	T	CTBP2	Missense	p.P265Q	6	70	8.6%	.	.	.
GC13	10	127434447	G	T	EDRF1	Missense	p.G887V	7	72	9.7%	.	.	.
GC13	10	127526949	C	A	DHX32	Missense	p.R630L	4	37	10.8%	.	.	.
GC13	10	127708294	C	A	ADAM12	Missense	p.W877L	7	32	21.9%	.	.	.
GC13	10	127798394	G	T	ADAM12	Missense	p.L207I	7	96	7.3%	.	.	.
GC13	10	129207642	G	T	DOCK1	Nonsense	p.E1444X	5	42	11.9%	.	.	.
GC13	10	129682113	G	T	CLRN3	Missense	p.Q86K	6	52	11.5%	.	.	.
GC13	10	129854399	G	T	PTPRE	Nonsense	p.G87X	6	72	8.3%	.	.	.
GC13	10	129868613	G	T	PTPRE	Missense	p.G178W	5	55	9.1%	.	.	.
GC13	10	134219236	C	A	PWWP2B	Missense	p.P411H	8	62	12.9%	.	.	.
GC13	10	134591197	C	A	INPP5A	Missense	p.R270S	6	77	7.8%	.	.	.
GC13	10	135082280	G	T	ADAM8	Missense	p.R614S	6	59	10.2%	.	.	.
GC13	10	135095807	C	A	TUBGCP2	Missense	p.G647W	6	61	9.8%	.	.	.
GC13	10	135370617	G	T	SYCE1	Missense	p.L140M	8	78	10.3%	.	.	.
GC13	11	197351	G	T	ODF3	Missense	p.R16L	5	57	8.8%	.	.	.
GC13	11	205433	C	A	BET1L	Missense	p.G69W	8	85	9.4%	.	.	.
GC13	11	239056	C	A	PSMD13	Missense	p.Q52K	8	83	9.6%	.	.	.
GC13	11	280951	G	T	NLRP6	Missense	p.R406L	6	81	7.4%	.	.	.
GC13	11	281659	C	A	NLRP6	Missense	p.P642Q	7	78	9.0%	.	.	.
GC13	11	292048	C	A	PGGHG	Missense	p.R327S	5	49	10.2%	.	.	.
GC13	11	397444	G	T	PKP3	Missense	p.G315C	5	73	6.8%	.	.	.
GC13	11	418808	C	A	ANO9	Missense	p.R537M	7	60	11.7%	.	.	.
GC13	11	489733	C	A	PTDSS2	Missense	p.P163Q	6	52	11.5%	.	.	.
GC13	11	498104	G	T	RNH1	Missense	p.H332N	7	60	11.7%	.	.	.
GC13	11	608454	C	A	PHRF1	Missense	p.R1000S	11	61	18.0%	.	.	.
GC13	11	1025253	C	A	MUC6	Missense	p.G972W	6	73	8.2%	.	.	.

GC13	11	1075722	G	T	MUC2	Missense	p.G50W	5	51	9.8%	.	.	.
GC13	11	1097734	G	T	MUC2	Missense	p.G2276V	7	79	8.9%	.	.	.
GC13	11	1275432	G	T	MUC5B	Missense	p.G5110W	8	93	8.6%	.	.	.
GC13	11	2188129	G	T	TH	Missense	p.R277S	6	61	9.8%	.	.	.
GC13	11	3039957	C	A	CARS	Missense	p.W390L	5	35	14.3%	.	.	.
GC13	11	3123512	C	A	OSBPL5	Splicing	c.1327-1G>T;NM_001144063	7	54	13.0%	.	.	.
GC13	11	3125592	G	T	OSBPL5	Missense	p.Q291K	5	36	13.9%	.	.	.
GC13	11	3129001	G	T	OSBPL5	Missense	p.P221Q	6	65	9.2%	.	.	.
GC13	11	3714563	G	T	NUP98	Missense	p.Q1404K	7	79	8.9%	.	.	.
GC13	11	3844221	C	A	PGAP2	Missense	p.Q73K	6	44	13.6%	.	.	.
GC13	11	3877516	C	A	STIM1	Missense	p.R6S	6	83	7.2%	.	.	.
GC13	11	4142841	G	T	RRM1	Missense	p.G73V	8	80	10.0%	.	.	.
GC13	11	4154830	G	T	RRM1	Missense	p.R310L	9	103	8.7%	.	.	.
GC13	11	4389251	C	A	OR52B4	Missense	p.G92V	8	88	9.1%	.	.	.
GC13	11	4389399	C	A	OR52B4	Missense	p.G43W	11	89	12.4%	.	O	.
GC13	11	4510851	G	T	OR52K1	Missense	p.G241W	8	92	8.7%	.	O	.
GC13	11	4703117	C	A	OR51E2	Missense	p.M275I	9	92	9.8%	.	.	.
GC13	11	4791021	C	A	OR51F1	Missense	p.G43W	8	78	10.3%	.	.	.
GC13	11	4870112	C	A	OR51S1	Missense	p.M109I	7	67	10.4%	.	.	.
GC13	11	4903255	G	T	OR51T1	Missense	p.L69F	9	97	9.3%	.	.	.
GC13	11	4928869	G	T	OR51A7	Missense	p.M90I	8	97	8.2%	.	.	.
GC13	11	5067846	G	T	OR52J3	Missense	p.G31W	6	87	6.9%	.	O	.
GC13	11	5444201	G	T	OR51Q1	Missense	p.M257I	10	93	10.8%	.	.	.
GC13	11	5475487	G	T	OR51I2	Missense	p.G257W	8	100	8.0%	.	.	.
GC13	11	5686435	C	A	TRIM5	Missense	p.E362D	6	65	9.2%	.	.	.
GC13	11	5776644	G	T	OR52N4	Missense	p.R225L	7	84	8.3%	.	.	.
GC13	11	5862433	C	A	OR52E6	Missense	p.W232L	8	91	8.8%	.	.	.
GC13	11	5878359	G	T	OR52E8	Missense	p.R192S	6	81	7.4%	.	.	.
GC13	11	6129011	G	T	OR56B4	Missense	p.M1I	8	86	9.3%	.	.	.
GC13	11	6235707	G	T	FAM160A2	Missense	p.R831S	5	40	12.5%	.	.	.
GC13	11	6265376	C	A	CNGA4	Missense	p.L489M	8	88	9.1%	.	.	.

GC13	11	6292258	G	T	CCKBR	Missense	p.G193W	6	65	9.2%	.	.	.
GC13	11	6340715	G	T	CAVIN3	Missense	p.P155Q	6	68	8.8%	.	.	.
GC13	11	6566996	G	T	DNHD1	Missense	p.L1609F	8	90	8.9%	.	.	.
GC13	11	6578237	G	T	DNHD1	Missense	p.W2571L	6	62	9.7%	.	.	.
GC13	11	6585741	G	T	DNHD1	Missense	p.R3488L	6	67	9.0%	.	.	.
GC13	11	6638360	G	T	TPP1	Missense	p.P178Q	6	64	9.4%	.	.	.
GC13	11	6644137	G	T	DCHS1	Missense	p.H2924N	8	71	11.3%	.	.	.
GC13	11	6648896	C	A	DCHS1	Missense	p.G1792W	6	67	9.0%	.	.	.
GC13	11	6704034	C	A	MRPL17	Missense	p.G63W	7	70	10.0%	.	.	.
GC13	11	6867338	G	T	OR10A5	Missense	p.R142L	8	96	8.3%	.	O	.
GC13	11	6898001	G	T	OR10A4	Missense	p.M41I	7	96	7.3%	.	.	.
GC13	11	6898578	G	T	OR10A4	Missense	p.G234W	11	126	8.7%	.	.	.
GC13	11	7021760	G	T	ZNF214	Missense	p.P385Q	7	78	9.0%	.	.	.
GC13	11	7670872	G	T	PPFIBP2	Missense	p.R560L	7	85	8.2%	.	.	.
GC13	11	7723014	G	T	OVCH2	Missense	p.L190I	7	92	7.6%	.	.	.
GC13	11	7727884	C	A	OVCH2	Missense	p.G20C	10	95	10.5%	.	.	.
GC13	11	7847400	C	A	OR5P3	Missense	p.M40I	7	77	9.1%	.	.	.
GC13	11	7981797	C	A	NLRP10	Missense	p.L454F	8	88	9.1%	.	.	.
GC13	11	7982731	G	T	NLRP10	Missense	p.P143Q	7	75	9.3%	.	.	.
GC13	11	8060452	G	T	TUB	Missense	p.W11L	8	89	9.0%	.	.	.
GC13	11	8668068	G	T	TRIM66	Missense	p.Q150K	8	99	8.1%	.	.	.
GC13	11	8718139	G	T	ST5	Missense	p.L623I	7	77	9.1%	.	.	.
GC13	11	8752052	C	A	ST5	Missense	p.R262L	6	92	6.5%	.	.	.
GC13	11	8959479	G	T	ASCL3	Missense	p.P77Q	5	52	9.6%	.	.	.
GC13	11	9080952	G	T	SCUBE2	Missense	p.R330S	6	64	9.4%	.	.	.
GC13	11	9202421	C	A	DENND5A	Missense	p.G426W	7	72	9.7%	.	.	.
GC13	11	9530285	G	T	ZNF143	Missense	p.G392W	7	88	8.0%	.	.	.
GC13	11	9761798	G	T	SWAP70	Missense	p.R362L	6	99	6.1%	.	.	.
GC13	11	10050000	C	A	SBF2	Missense	p.L206F	7	79	8.9%	.	O	.
GC13	11	10064513	C	A	SBF2	Missense	p.G53W	6	63	9.5%	.	.	.
GC13	11	10647890	C	A	MRVI1	Missense	p.G16W	7	62	11.3%	.	.	.

GC13	11	10785273	G	T	CTR9	Missense	p.L347F	9	135	6.7%	.	.	.
GC13	11	11954575	C	A	USP47	Missense	p.P491Q	8	82	9.8%	.	.	.
GC13	11	12237807	C	A	MICAL2	Missense	p.H297N	7	98	7.1%	.	.	.
GC13	11	12530179	G	T	PARVA	Missense	p.G276C	8	131	6.1%	.	.	.
GC13	11	14496109	G	T	COPB1	Missense	p.L557I	7	69	10.1%	.	.	.
GC13	11	14901720	C	A	CYP2R1	Missense	p.W321L	7	89	7.9%	.	.	.
GC13	11	16208432	C	A	SOX6	Missense	p.R202L	10	97	10.3%	.	O	.
GC13	11	16340117	C	A	SOX6	Missense	p.R107L	6	86	7.0%	.	O	.
GC13	11	16847841	C	A	PLEKHA7	Missense	p.R390L	5	66	7.6%	.	.	.
GC13	11	17191000	G	T	PIK3C2A	Missense	p.Q97K	10	94	10.6%	.	.	.
GC13	11	17191039	G	T	PIK3C2A	Missense	p.Q84K	9	110	8.2%	.	.	.
GC13	11	17409490	C	A	KCNJ11	Missense	p.R50L	7	80	8.8%	.	.	.
GC13	11	17470168	C	A	ABCC8	Missense	p.M409I	6	49	12.2%	.	.	.
GC13	11	17482160	C	A	ABCC8	Missense	p.G296W	5	40	12.5%	.	.	.
GC13	11	17632066	C	A	OTOG	Missense	p.P1752Q	6	62	9.7%	.	.	.
GC13	11	18050759	C	A	TPH1	Missense	p.R207L	6	83	7.2%	.	.	.
GC13	11	18195342	C	A	MRGPRX4	Missense	p.P180Q	7	61	11.5%	.	.	.
GC13	11	18467861	C	A	LDHC	Missense	p.P272Q	8	81	9.9%	.	.	.
GC13	11	18591833	C	A	UEVLD	Missense	p.M73I	7	92	7.6%	.	.	.
GC13	11	18637150	G	T	SPTY2D1	Missense	p.P224H	7	87	8.0%	.	.	.
GC13	11	18637199	G	T	SPTY2D1	Missense	p.L208I	8	108	7.4%	.	.	.
GC13	11	18743140	G	T	IGSF22	Missense	p.P107H	7	70	10.0%	.	.	.
GC13	11	19173788	G	T	ZDHHC13	Missense	p.W93L	8	140	5.7%	.	.	.
GC13	11	19970388	G	T	NAV2	Missense	p.G739W	8	89	9.0%	.	.	.
GC13	11	20075679	G	T	NAV2	Missense	p.R576L	6	71	8.5%	.	.	.
GC13	11	20089886	G	T	NAV2	Missense	p.R706L	7	76	9.2%	.	.	.
GC13	11	20104580	G	T	NAV2	Missense	p.G849W	9	74	12.2%	.	.	.
GC13	11	22646678	G	T	FANCF	Missense	p.H227N	7	85	8.2%	O	.	.
GC13	11	25098952	G	T	LUZP2	Missense	p.Q270H	7	73	9.6%	.	.	.
GC13	11	27137054	G	T	BBOX1	Missense	p.G197W	11	110	10.0%	.	.	.
GC13	11	27406999	G	T	LGR4	Missense	p.H116N	7	60	11.7%	.	.	.

GC13	11	28045289	C	A	KIF18A	Missense	p.M871I	8	71	11.3%	.	.	.
GC13	11	30032413	G	T	KCNA4	Missense	p.L605M	10	108	9.3%	.	.	.
GC13	11	30032691	G	T	KCNA4	Missense	p.P512Q	7	91	7.7%	.	O	.
GC13	11	30032868	C	A	KCNA4	Missense	p.R453L	5	66	7.6%	.	.	.
GC13	11	30033620	C	A	KCNA4	Missense	p.L202F	7	94	7.4%	.	O	.
GC13	11	30938432	G	T	DCDC1	Missense	p.P253Q	9	114	7.9%	.	.	.
GC13	11	30953411	G	T	DCDC1	Missense	p.P42H	7	90	7.8%	.	.	.
GC13	11	31349697	G	T	DCDC1	Missense	p.P44Q	5	36	13.9%	.	.	.
GC13	11	32610260	C	A	EIF3M	Missense	p.P99Q	9	81	11.1%	.	.	.
GC13	11	32632747	C	A	CCDC73	Missense	p.K987N	7	73	9.6%	.	O	.
GC13	11	32635538	G	T	CCDC73	Missense	p.H776N	8	91	8.8%	.	.	.
GC13	11	33049291	G	T	DEPDC7	Missense	p.W175L	9	100	9.0%	.	.	.
GC13	11	33106637	C	A	CSTF3	Missense	p.R717L	6	97	6.2%	.	.	.
GC13	11	33596371	C	A	KIAA1549L	Missense	p.Q1155K	10	97	10.3%	.	.	.
GC13	11	33768695	C	A	FBXO3	Missense	p.R407M	9	102	8.8%	.	.	.
GC13	11	34474704	G	T	CAT	Missense	p.W183L	8	92	8.7%	.	.	.
GC13	11	34475370	G	T	CAT	Missense	p.R203L	5	64	7.8%	.	.	.
GC13	11	34477634	G	T	CAT	Missense	p.R263L	8	105	7.6%	.	.	.
GC13	11	34482913	C	A	CAT	Missense	p.P391Q	5	55	9.1%	.	.	.
GC13	11	34527239	G	T	ELF5	Missense	p.L20M	7	71	9.9%	.	.	.
GC13	11	34904959	G	T	APIP	Missense	p.P185Q	5	44	11.4%	.	.	.
GC13	11	34988186	G	T	PDHX	Splicing	c.642-1G>T	9	98	9.2%	.	.	.
GC13	11	36512098	C	A	TRAF6	Missense	p.G287W	6	56	10.7%	.	.	.
GC13	11	36595024	C	A	RAG1	Missense	p.P57H	7	85	8.2%	.	.	.
GC13	11	36595795	G	T	RAG1	Missense	p.R314L	7	104	6.7%	.	.	.
GC13	11	44079912	G	T	ACCSL	Missense	p.R458L	7	96	7.3%	.	.	.
GC13	11	44096167	G	T	ACCS	Missense	p.R142L	6	68	8.8%	.	.	.
GC13	11	44193174	G	T	EXT2	Missense	p.W429L	8	77	10.4%	O	.	.
GC13	11	44255699	G	T	EXT2	Missense	p.G647V	10	71	14.1%	O	.	.
GC13	11	45246290	G	T	PRDM11	Missense	p.R422L	8	93	8.6%	.	.	.
GC13	11	45671693	C	A	CHST1	Missense	p.G261W	8	70	11.4%	.	.	.

GC13	11	45926521	G	T	MAPK8IP1	Splicing	c.1894-1G>T	7	86	8.1%	.	.	.
GC13	11	45975094	C	A	PHF21A	Missense	p.R359L	7	90	7.8%	.	.	.
GC13	11	46564651	C	A	AMBRA1	Missense	p.G306W	9	67	13.4%	.	.	.
GC13	11	46667445	C	A	ATG13	Missense	p.P59Q	10	146	6.8%	.	.	.
GC13	11	46806184	G	T	CKAP5	Missense	p.Q670K	7	81	8.6%	.	.	.
GC13	11	46911858	C	A	LRP4	Missense	p.G629W	7	88	8.0%	.	.	.
GC13	11	47296524	G	T	MADD	Missense	p.R158L	9	106	8.5%	.	.	.
GC13	11	47303209	G	T	MADD	Missense	p.L458F	9	63	14.3%	.	.	.
GC13	11	47330853	G	T	MADD	Missense	p.W1215L	8	133	6.0%	.	.	.
GC13	11	47362690	C	A	MYBPC3	Missense	p.M632I	8	77	10.4%	.	.	.
GC13	11	47363552	G	T	MYBPC3	Missense	p.H594N	9	68	13.2%	.	.	.
GC13	11	47445676	C	A	PSMC3	Missense	p.R171L	6	98	6.1%	.	O	.
GC13	11	47446029	G	T	PSMC3	Missense	p.P135H	8	114	7.0%	.	.	.
GC13	11	47469537	G	T	RAPSN	Missense	p.Q120K	7	87	8.0%	.	.	.
GC13	11	47603938	C	A	NDUFS3	Missense	p.P182H	7	81	8.6%	.	.	.
GC13	11	47833856	G	T	NUP160	Missense	p.P693Q	8	107	7.5%	.	.	.
GC13	11	55339705	G	T	OR4C16	Missense	p.L34F	7	85	8.2%	.	O	.
GC13	11	55433246	G	T	OR4C6	Missense	p.G202W	7	85	8.2%	.	.	.
GC13	11	55541616	G	T	OR5D13	Missense	p.G235W	7	89	7.9%	.	O	.
GC13	11	55681381	C	A	OR5W2	Missense	p.L226F	8	98	8.2%	.	.	.
GC13	11	55927408	G	T	OR8K5	Missense	p.P129H	7	87	8.0%	.	.	.
GC13	11	56020686	G	T	OR5T3	Missense	p.L337F	7	85	8.2%	.	.	.
GC13	11	56043357	G	T	OR5T1	Missense	p.L81F	7	97	7.2%	.	.	.
GC13	11	56058221	C	A	OR8H1	Missense	p.L106F	7	75	9.3%	.	O	.
GC13	11	56309980	C	A	OR5M11	Missense	p.G252W	6	77	7.8%	.	.	.
GC13	11	57076149	G	T	TNKS1BP1	Missense	p.Q1346K	9	92	9.8%	.	.	.
GC13	11	57076896	G	T	TNKS1BP1	Missense	p.Q1097K	10	95	10.5%	.	.	.
GC13	11	57114657	C	A	P2RX3	Missense	p.P108Q	7	71	9.9%	.	.	.
GC13	11	57184133	C	A	SLC43A3	Missense	p.G228W	7	70	10.0%	.	.	.
GC13	11	57367658	G	T	SERPING1	Missense	p.G120W	11	127	8.7%	.	.	.
GC13	11	57466304	G	T	ZDHHC5	Nonsense	p.G466X	7	96	7.3%	.	.	.

GC13	11	57466437	G	T	ZDHHC5	Missense	p.R510L	9	101	8.9%	.	.	.
GC13	11	57466727	C	A	ZDHHC5	Missense	p.R607S	6	62	9.7%	.	.	.
GC13	11	57513174	G	T	BTBD18	Missense	p.Q191K	6	79	7.6%	.	.	.
GC13	11	57569480	C	A	CTNND1	Missense	p.P310Q	10	91	11.0%	.	.	.
GC13	11	57569503	C	A	CTNND1	Missense	p.H318N	8	81	9.9%	.	.	.
GC13	11	57573406	G	T	CTNND1	Missense	p.R491L	6	91	6.6%	.	.	.
GC13	11	57581798	G	T	CTNND1	Missense	p.R778L	7	107	6.5%	.	.	.
GC13	11	57799252	G	T	OR6Q1	Missense	p.K276N	8	93	8.6%	.	O	.
GC13	11	57971494	C	A	OR1S2	Missense	p.G54W	11	101	10.9%	.	.	.
GC13	11	57982601	G	T	OR1S1	Missense	p.G129W	7	89	7.9%	.	.	.
GC13	11	58206827	C	A	OR5B12	Missense	p.M266I	9	76	11.8%	.	.	.
GC13	11	58294996	C	A	LPXN	Missense	p.K344N	10	114	8.8%	.	.	.
GC13	11	58477601	G	T	GLYAT	Missense	p.H177N	8	94	8.5%	.	.	.
GC13	11	58491902	G	T	GLYAT	Missense	p.P23Q	9	92	9.8%	.	O	.
GC13	11	58892820	G	T	FAM111B	Missense	p.R387L	12	86	14.0%	.	.	.
GC13	11	58919901	C	A	FAM111A	Missense	p.Q254K	9	141	6.4%	.	.	.
GC13	11	58920702	C	A	FAM111A	Missense	p.Q521K	7	81	8.6%	.	.	.
GC13	11	58949857	G	T	DTX4	Missense	p.R180M	8	119	6.7%	.	.	.
GC13	11	58956654	G	T	DTX4	Missense	p.M233I	5	31	16.1%	.	.	.
GC13	11	59132255	G	T	OR5AN1	Missense	p.M108I	8	110	7.3%	.	.	.
GC13	11	59189667	C	A	OR5A2	Missense	p.G254C	7	71	9.9%	.	.	.
GC13	11	59282505	G	T	OR4D9	Missense	p.M40I	10	85	11.8%	.	.	.
GC13	11	59423111	C	A	PATL1	Missense	p.G306W	7	41	17.1%	.	.	.
GC13	11	59480501	C	A	OR10V1	Missense	p.R273L	8	86	9.3%	.	.	.
GC13	11	59575226	C	A	MRPL16	Missense	p.R73L	6	81	7.4%	.	O	.
GC13	11	60264923	G	T	MS4A12	Missense	p.Q44H	8	79	10.1%	.	.	.
GC13	11	60776122	C	A	CD6	Missense	p.H196N	6	57	10.5%	.	.	.
GC13	11	60778581	C	A	CD6	Missense	p.P375H	7	90	7.8%	.	.	.
GC13	11	60780934	G	T	CD6	Missense	p.R397L	6	76	7.9%	.	.	.
GC13	11	60885811	G	T	CD5	Missense	p.G30W	7	78	9.0%	.	.	.
GC13	11	60886925	G	T	CD5	Missense	p.R171L	6	83	7.2%	.	.	.

GC13	11	61048137	G	T	VWCE	Missense	p.P428Q	10	86	11.6%	.	.	.
GC13	11	61048141	G	T	VWCE	Missense	p.H427N	8	85	9.4%	.	.	.
GC13	11	61058976	C	A	VWCE	Missense	p.M61I	7	66	10.6%	.	.	.
GC13	11	61090494	G	T	DDB1	Missense	p.Q332K	8	92	8.7%	.	.	.
GC13	11	61133688	G	T	TMEM138	Missense	p.M42I	7	64	10.9%	.	.	.
GC13	11	61160750	G	T	TMEM216	Missense	p.G28W	4	37	10.8%	.	.	.
GC13	11	61249786	G	T	PPP1R32	Missense	p.R38L	5	65	7.7%	.	.	.
GC13	11	61254627	G	T	PPP1R32	Missense	p.G300W	7	86	8.1%	.	.	.
GC13	11	61498849	G	T	DAGLA	Missense	p.G304W	6	74	8.1%	.	.	.
GC13	11	61511243	G	T	DAGLA	Missense	p.R804L	5	46	10.9%	.	.	.
GC13	11	61562982	G	T	FEN1	Missense	p.G50V	6	53	11.3%	.	.	.
GC13	11	61563491	C	A	FEN1	Missense	p.Q220K	8	109	7.3%	.	O	.
GC13	11	61643404	G	T	FADS3	Missense	p.P402Q	5	29	17.2%	.	.	.
GC13	11	61917604	G	T	INCENP	Missense	p.G819W	7	68	10.3%	.	.	.
GC13	11	62288597	G	T	AHNAK	Missense	p.P4431H	8	105	7.6%	.	.	.
GC13	11	62294663	G	T	AHNAK	Missense	p.P2409H	7	91	7.7%	.	.	.
GC13	11	62297088	G	T	AHNAK	Missense	p.L1601I	9	88	10.2%	.	.	.
GC13	11	62363535	G	T	MTA2	Missense	p.P181H	8	134	6.0%	.	O	.
GC13	11	62363943	C	A	MTA2	Missense	p.R142L	6	76	7.9%	.	.	.
GC13	11	62371487	G	T	EML3	Missense	p.H701N	9	93	9.7%	.	.	.
GC13	11	62372624	G	T	EML3	Missense	p.P649Q	7	68	10.3%	.	.	.
GC13	11	62381034	C	A	ROM1	Missense	p.P94H	8	55	14.5%	.	.	.
GC13	11	62416506	G	T	INTS5	Missense	p.P349Q	6	60	10.0%	.	.	.
GC13	11	62430774	C	A	LBHD1	Missense	p.G66W	9	112	8.0%	.	.	.
GC13	11	62483073	G	T	HNRNPUL2	Missense	p.R688S	9	118	7.6%	.	.	.
GC13	11	62559970	G	T	NXF1	Missense	p.P613Q	8	82	9.8%	.	.	.
GC13	11	62653039	G	T	SLC3A2	Missense	p.G368W	6	95	6.3%	.	.	.
GC13	11	62850877	C	A	SLC22A24	Missense	p.G375W	7	91	7.7%	.	.	.
GC13	11	63487926	C	A	RTN3	Missense	p.P539Q	7	100	7.0%	.	.	.
GC13	11	63714474	G	T	NAA40	Missense	p.G31W	6	90	6.7%	.	.	.
GC13	11	63719920	G	T	NAA40	Missense	p.R77L	9	125	7.2%	.	.	.

GC13	11	63764080	C	A	OTUB1	Missense	p.H100N	7	80	8.8%	.	.	.
GC13	11	63883834	G	T	FLRT1	Missense	p.R32L	5	69	7.2%	.	.	.
GC13	11	64001440	G	T	DNAJC4	Missense	p.R202L	9	123	7.3%	.	.	.
GC13	11	64111779	C	A	CCDC88B	Missense	p.P589Q	5	58	8.6%	.	.	.
GC13	11	64360928	G	T	SLC22A12	Missense	p.M186I	5	35	14.3%	.	.	.
GC13	11	64402904	C	A	NRXN2	Missense	p.G96W	6	58	10.3%	.	.	.
GC13	11	64575505	C	A	MEN1	Missense	p.R176L	6	69	8.7%	O	.	.
GC13	11	64674997	C	A	ATG2A	Missense	p.A883S	7	96	7.3%	.	.	.
GC13	11	64889085	G	T	FAU	Missense	p.P36Q	6	60	10.0%	.	.	.
GC13	11	64899754	G	T	SYVN1	Missense	p.R166S	6	72	8.3%	.	O	.
GC13	11	64940258	G	T	SPDYC	Missense	p.R207L	7	70	10.0%	.	.	.
GC13	11	65043469	G	T	POLA2	Missense	p.S154I	7	88	8.0%	.	.	.
GC13	11	65124218	G	T	TIGD3	Missense	p.L313F	7	62	11.3%	.	.	.
GC13	11	65308675	G	T	LTBP3	Missense	p.Q939K	6	85	7.1%	.	.	.
GC13	11	65319733	G	T	LTBP3	Missense	p.P444Q	7	88	8.0%	.	O	.
GC13	11	65321193	C	A	LTBP3	Missense	p.Q281H	6	59	10.2%	.	.	.
GC13	11	65321573	G	T	LTBP3	Missense	p.Q204K	7	53	13.2%	.	.	.
GC13	11	65350178	C	A	EHBP1L1	Missense	p.Q679K	8	98	8.2%	.	.	.
GC13	11	65386079	G	T	PCNX3	Missense	p.G416W	7	95	7.4%	.	.	.
GC13	11	65386128	C	A	PCNX3	Missense	p.P432Q	7	86	8.1%	.	.	.
GC13	11	65401682	G	T	PCNX3	Missense	p.R1519L	5	36	13.9%	.	.	.
GC13	11	65480414	G	T	KAT5	Missense	p.W90L	7	83	8.4%	.	.	.
GC13	11	65545457	G	T	AP5B1	Missense	p.P836Q	4	25	16.0%	.	.	.
GC13	11	65793250	C	A	CATSPER1	Missense	p.G201W	6	44	13.6%	.	.	.
GC13	11	65824399	G	T	SF3B2	Missense	p.G214C	6	50	12.0%	.	.	.
GC13	11	65826422	C	A	SF3B2	Missense	p.P363Q	8	103	7.8%	.	.	.
GC13	11	66335043	C	A	CTSF	Missense	p.G135W	6	55	10.9%	.	.	.
GC13	11	66335084	C	A	CTSF	Missense	p.R121L	4	31	12.9%	.	.	.
GC13	11	66358464	G	T	CCDC87	Missense	p.H675N	8	83	9.6%	.	O	.
GC13	11	66359147	C	A	CCDC87	Missense	p.R447L	6	62	9.7%	.	.	.
GC13	11	66360200	C	A	CCDC87	Missense	p.R96L	7	84	8.3%	.	.	.

GC13	11	66373256	G	T	CCS	Missense	p.W252L	8	104	7.7%	.	.	.
GC13	11	66481107	G	T	SPTBN2	Missense	p.P256H	7	89	7.9%	.	.	.
GC13	11	66807406	G	T	SYT12	Missense	p.R3L	7	76	9.2%	.	.	.
GC13	11	66811160	C	A	SYT12	Missense	p.L110M	7	85	8.2%	.	.	.
GC13	11	67021847	G	T	KDM2A	Missense	p.G650W	8	108	7.4%	.	.	.
GC13	11	67077245	G	T	SSH3	Missense	p.Q405H	7	52	13.5%	.	O	.
GC13	11	67079197	C	A	SSH3	Missense	p.Q607K	6	63	9.5%	.	.	.
GC13	11	67159661	C	A	RAD9A	Missense	p.R22S	6	59	10.2%	.	.	.
GC13	11	67166032	G	T	PPP1CA	Missense	p.R279S	4	32	12.5%	.	.	.
GC13	11	67191665	C	A	CARNS1	Missense	p.R693S	6	71	8.5%	.	.	.
GC13	11	67812520	G	T	TCIRG1	Missense	p.Q156H	7	75	9.3%	.	.	.
GC13	11	67842185	G	T	CHKA	Missense	p.P192Q	6	80	7.5%	.	.	.
GC13	11	67947606	G	T	KMT5B	Missense	p.P124T	8	112	7.1%	.	.	.
GC13	11	67957426	G	T	KMT5B	Missense	p.L40M	8	92	8.7%	.	.	.
GC13	11	68154116	C	A	LRP5	Missense	p.R450S	7	80	8.8%	.	.	.
GC13	11	68514737	G	T	TESMIN	Missense	p.P190Q	7	96	7.3%	.	.	.
GC13	11	68571493	G	T	CPT1A	Missense	p.P177Q	7	85	8.2%	.	O	.
GC13	11	70222671	C	A	PPFIA1	Missense	p.L1117I	8	81	9.9%	.	O	.
GC13	11	70261817	C	A	CTTN	Missense	p.Q151K	7	62	11.3%	.	.	.
GC13	11	71195403	G	T	NADSYN1	Missense	p.M455I	8	110	7.3%	.	.	.
GC13	11	71944743	G	T	INPPL1	Missense	p.G723W	7	75	9.3%	.	.	.
GC13	11	72407630	C	A	ARAP1	Missense	p.R773L	6	50	12.0%	.	.	.
GC13	11	72712146	C	A	FCHSD2	Missense	p.E92D	7	87	8.0%	.	.	.
GC13	11	73022111	C	A	ARHGEF17	Missense	p.L810M	10	83	12.0%	.	.	.
GC13	11	73022732	G	T	ARHGEF17	Missense	p.G1017C	8	91	8.8%	.	.	.
GC13	11	73070900	G	T	ARHGEF17	Missense	p.R1370L	6	38	15.8%	.	.	.
GC13	11	73584383	C	A	COA4	Missense	p.R14L	6	66	9.1%	.	.	.
GC13	11	73681037	G	T	DNAJB13	Missense	p.G277W	7	88	8.0%	.	.	.
GC13	11	73809200	C	A	C2CD3	Missense	p.G945W	8	95	8.4%	.	O	.
GC13	11	73990472	C	A	P4HA3	Missense	p.G379V	7	66	10.6%	.	.	.
GC13	11	74054451	G	T	PGM2L1	Missense	p.P410Q	9	93	9.7%	.	.	.

GC13	11	74058332	C	A	PGM2L1	Missense	p.G267V	6	66	9.1%	.	.	.
GC13	11	75298635	C	A	MAP6	Missense	p.K637N	7	97	7.2%	.	.	.
GC13	11	75298785	C	A	MAP6	Missense	p.M587I	7	89	7.9%	.	.	.
GC13	11	75902624	G	T	WNT11	Missense	p.H292N	5	42	11.9%	.	.	.
GC13	11	75902735	G	T	WNT11	Missense	p.R255S	6	45	13.3%	.	.	.
GC13	11	75907554	G	T	WNT11	Missense	p.P98T	7	77	9.1%	.	.	.
GC13	11	76092948	C	A	LOC100506 127	Missense	p.H26N	8	120	6.7%	.	.	.
GC13	11	76893566	C	A	MYO7A	Missense	p.P1069H	11	89	12.4%	.	.	.
GC13	11	76954751	G	T	GDPD4	Missense	p.P410H	8	90	8.9%	.	.	.
GC13	11	77409731	G	T	RSF1	Missense	p.P839H	7	61	11.5%	.	.	.
GC13	11	77589768	G	T	AAMDC	Missense	p.G117C	7	99	7.1%	.	.	.
GC13	11	77885498	C	A	KCTD21	Missense	p.G35W	6	82	7.3%	.	.	.
GC13	11	77909030	C	A	USP35	Missense	p.P241Q	7	77	9.1%	.	.	.
GC13	11	77911811	C	A	USP35	Missense	p.P385Q	6	59	10.2%	.	.	.
GC13	11	78381364	C	A	TENM4	Missense	p.R2009M	8	114	7.0%	.	.	.
GC13	11	82564320	C	A	PRCP	Missense	p.G104W	7	91	7.7%	.	.	.
GC13	11	82698676	C	A	RAB30	Missense	p.R105L	7	141	5.0%	.	.	.
GC13	11	82874782	C	A	PCF11	Missense	p.P127H	8	124	6.5%	.	.	.
GC13	11	83243827	C	A	DLG2	Splicing	c.1803-1G>T;NM_001 142699	8	109	7.3%	.	.	.
GC13	11	85695017	C	A	PICALM	Splicing	c.1388-1G>T;NM_001 206947	7	79	8.9%	.	.	.
GC13	11	88911242	G	T	TYR	Missense	p.G41W	8	85	9.4%	.	.	.
GC13	11	92532442	C	A	FAT3	Missense	p.P2088Q	7	66	10.6%	.	.	.
GC13	11	92533858	G	T	FAT3	Missense	p.R2560L	6	90	6.7%	.	O	.
GC13	11	92534367	C	A	FAT3	Missense	p.L2730M	7	96	7.3%	.	.	.
GC13	11	93065441	C	A	DEUP1	Missense	p.H6N	7	88	8.0%	.	.	.
GC13	11	93543018	C	A	MED17	Missense	p.P574T	7	81	8.6%	.	.	.
GC13	11	94039725	G	T	IZUMO1R	Missense	p.W62L	9	103	8.7%	.	.	.
GC13	11	94040634	C	A	IZUMO1R	Missense	p.H177N	7	85	8.2%	.	O	.
GC13	11	94179033	G	T	MRE11	Missense	p.R603S	8	87	9.2%	.	O	.
GC13	11	94300771	G	T	PIWIL4	Missense	p.L29F	7	71	9.9%	.	.	.

GC13	11	94301949	G	T	PIWIL4	Missense	p.L47F	10	114	8.8%	.	.	.
GC13	11	94533240	C	A	AMOTL1	Missense	p.S245Y	10	78	12.8%	.	.	.
GC13	11	94862575	G	T	ENDOD1	Missense	p.M445I	8	106	7.5%	.	.	.
GC13	11	95519396	C	A	FAM76B	Missense	p.G84C	9	113	8.0%	.	.	.
GC13	11	100211888	G	T	CNTN5	Missense	p.W920L	8	87	9.2%	.	.	.
GC13	11	100221509	C	A	CNTN5	Missense	p.P962Q	8	92	8.7%	.	.	.
GC13	11	101762317	C	A	ANGPTL5	Missense	p.R287L	5	67	7.5%	.	.	.
GC13	11	101773359	G	T	ANGPTL5	Missense	p.P178Q	7	78	9.0%	.	.	.
GC13	11	101833477	G	T	CEP126	Missense	p.G571C	7	83	8.4%	.	.	.
GC13	11	102668161	G	T	MMP1	Missense	p.P59Q	11	133	8.3%	.	.	.
GC13	11	102816416	G	T	MMP13	Missense	p.P425Q	10	132	7.6%	.	.	.
GC13	11	102980386	G	T	DYNC2H1	Missense	p.W28L	8	100	8.0%	.	.	.
GC13	11	103025223	C	A	DYNC2H1	Missense	p.L1116M	8	99	8.1%	.	.	.
GC13	11	103068681	G	T	DYNC2H1	Missense	p.W2576C	7	86	8.1%	.	.	.
GC13	11	103116022	G	T	DYNC2H1	Missense	p.G3321W	8	86	9.3%	.	.	.
GC13	11	103325918	G	T	DYNC2H1	Missense	p.W4154L	10	107	9.3%	.	.	.
GC13	11	104900521	C	A	CASP1	Missense	p.G224W	8	103	7.8%	.	O	.
GC13	11	105483106	G	T	GRIA4	Missense	p.L64F	8	115	7.0%	.	.	.
GC13	11	105881502	C	A	MSANTD4	Missense	p.W48L	10	142	7.0%	.	.	.
GC13	11	106810238	G	T	GUCY1A2	Missense	p.P385Q	8	82	9.8%	.	.	.
GC13	11	107197748	G	T	CWF19L2	Missense	p.P858Q	9	120	7.5%	.	.	.
GC13	11	107286954	G	T	CWF19L2	Missense	p.P533Q	9	83	10.8%	.	.	.
GC13	11	107427561	G	T	ALKBH8	Missense	p.L103I	7	63	11.1%	.	.	.
GC13	11	107535826	G	T	ELMOD1	Missense	p.R295M	8	86	9.3%	.	.	.
GC13	11	107960224	G	T	CUL5	Missense	p.L439F	7	92	7.6%	.	.	.
GC13	11	108216571	G	T	ATM	Missense	p.L2840F	9	145	6.2%	O	.	.
GC13	11	108380728	C	A	EXPH5	Missense	p.G1648W	7	89	7.9%	.	.	.
GC13	11	108384645	C	A	EXPH5	Missense	p.W342L	10	108	9.3%	.	.	.
GC13	11	108409812	G	T	EXPH5	Missense	p.L52M	9	128	7.0%	.	.	.
GC13	11	108535927	C	A	DDX10	Missense	p.P16Q	9	113	8.0%	.	.	.
GC13	11	108811065	G	T	DDX10	Missense	p.W848L	7	95	7.4%	.	.	.

GC13	11	110102737	G	T	RDX	Missense	p.Q190K	8	100	8.0%	.	.	.
GC13	11	110450716	C	A	ARHGAP20	Missense	p.R962L	7	107	6.5%	.	.	.
GC13	11	111177170	G	T	COLCA2	Missense	p.R70L	8	118	6.8%	.	.	.
GC13	11	111368768	G	T	BTG4	Missense	p.P89Q	6	78	7.7%	.	.	.
GC13	11	111404495	C	A	C11orf88	Missense	p.Q122K	7	85	8.2%	.	.	.
GC13	11	111572181	G	T	SIK2	Missense	p.M203I	6	84	7.1%	.	.	.
GC13	11	111582954	C	A	SIK2	Missense	p.P374Q	7	76	9.2%	.	.	.
GC13	11	111624227	C	A	PPP2R1B	Missense	p.L241F	8	112	7.1%	.	.	.
GC13	11	111745984	G	T	FDXACB1	Missense	p.R513S	7	114	6.1%	.	.	.
GC13	11	111915953	G	T	DLAT	Missense	p.R430L	7	112	6.3%	.	.	.
GC13	11	111916586	G	T	DLAT	Splicing	c.1291-1G>T	8	109	7.3%	.	.	.
GC13	11	111916658	G	T	DLAT	Missense	p.M454I	7	91	7.7%	.	.	.
GC13	11	111953214	G	T	C11orf57	Missense	p.G134W	7	89	7.9%	.	.	.
GC13	11	113567678	C	A	TMPRSS5	Missense	p.K116N	7	71	9.9%	.	.	.
GC13	11	113576944	C	A	TMPRSS5	Missense	p.M1I	7	65	10.8%	.	.	.
GC13	11	113853852	G	T	HTR3A	Missense	p.G135W	7	87	8.0%	.	.	.
GC13	11	113853871	C	A	HTR3A	Missense	p.P141Q	6	87	6.9%	.	.	.
GC13	11	114441898	G	T	NXPE4	Missense	p.P466Q	8	124	6.5%	.	.	.
GC13	11	114569182	G	T	NXPE2	Missense	p.W183L	5	34	14.7%	.	.	.
GC13	11	114577267	C	A	NXPE2	Missense	p.P432Q	9	118	7.6%	.	.	.
GC13	11	116691822	G	T	APOA4	Missense	p.L318M	7	84	8.3%	.	.	.
GC13	11	117058165	G	T	SIDT2	Nonsense	p.E363X	8	111	7.2%	.	.	.
GC13	11	117152041	C	A	RNF214	Missense	p.P386Q	6	36	16.7%	.	.	.
GC13	11	117186258	G	T	BACE1	Missense	p.P85Q	5	40	12.5%	.	.	.
GC13	11	117267299	C	A	CEP164	Missense	p.Q1087K	9	98	9.2%	.	.	.
GC13	11	117787962	C	A	TMPRSS13	Missense	p.R160L	5	45	11.1%	.	.	.
GC13	11	117864009	G	T	IL10RA	Missense	p.G141W	7	72	9.7%	.	O	.
GC13	11	117870051	G	T	IL10RA	Missense	p.G478W	6	91	6.6%	.	.	.
GC13	11	117969753	G	T	TMPRSS4	Missense	p.G33W	8	79	10.1%	.	.	.
GC13	11	118133206	G	T	MPZL2	Missense	p.P128Q	11	112	9.8%	.	.	.
GC13	11	118343690	G	T	KMT2A	Missense	p.G606W	9	142	6.3%	.	.	.

GC13	11	118375289	G	T	KMT2A	Missense	p.M2894I	7	88	8.0%	.	.	.
GC13	11	118398212	G	T	TTC36	Missense	p.M1I	6	79	7.6%	.	.	.
GC13	11	118404785	G	T	TMEM25	Missense	p.R145L	9	108	8.3%	.	.	.
GC13	11	118498044	G	T	PHLDB1	Missense	p.G169W	7	94	7.4%	.	.	.
GC13	11	118498104	C	A	PHLDB1	Missense	p.L189M	8	95	8.4%	.	.	.
GC13	11	118656861	C	A	DDX6	Missense	p.G34W	7	93	7.5%	.	.	.
GC13	11	118772371	G	T	BCL9L	Missense	p.P694H	7	74	9.5%	O	.	.
GC13	11	118886130	G	T	CCDC84	Nonsense	p.G307X	6	73	8.2%	.	.	.
GC13	11	118960702	G	T	HMBS	Missense	p.R116L	6	84	7.1%	.	O	.
GC13	11	118970987	G	T	DPAGT1	Missense	p.L210M	8	128	6.3%	.	.	.
GC13	11	118971736	G	T	DPAGT1	Missense	p.H92N	7	84	8.3%	.	.	.
GC13	11	119004760	G	T	HINFP	Missense	p.G390W	8	102	7.8%	.	.	.
GC13	11	119228761	G	T	USP2	Missense	p.P208H	7	96	7.3%	.	.	.
GC13	11	120187958	G	T	POU2F3	Missense	p.G388W	7	86	8.1%	.	.	.
GC13	11	120340012	G	T	ARHGEF12	Missense	p.R938M	8	114	7.0%	.	.	.
GC13	11	120348933	G	T	ARHGEF12	Missense	p.G1182W	7	92	7.6%	.	.	.
GC13	11	120350747	C	A	ARHGEF12	Missense	p.P1263Q	6	70	8.6%	.	.	.
GC13	11	120916410	C	A	TBCEL	Missense	p.P4H	6	59	10.2%	.	.	.
GC13	11	121174128	C	A	SC5D	Missense	p.P15Q	7	87	8.0%	.	.	.
GC13	11	121403201	G	T	SORL1	Missense	p.W542L	5	41	12.2%	.	.	.
GC13	11	122647748	G	T	UBASH3B	Missense	p.G78C	7	109	6.4%	.	.	.
GC13	11	122775905	C	A	C11orf63	Missense	p.P297Q	7	85	8.2%	.	.	.
GC13	11	123465536	G	T	GRAMD1B	Missense	p.W105L	11	111	9.9%	.	.	.
GC13	11	123474179	G	T	GRAMD1B	Missense	p.G183W	8	70	11.4%	.	.	.
GC13	11	123814283	C	A	OR6T1	Missense	p.G88V	7	93	7.5%	.	.	.
GC13	11	123900674	G	T	OR10G8	Missense	p.R115S	6	66	9.1%	.	.	.
GC13	11	124310456	G	T	OR8B8	Missense	p.H176N	9	116	7.8%	.	.	.
GC13	11	124765403	C	A	ROBO4	Missense	p.R184L	7	74	9.5%	.	.	.
GC13	11	124857939	G	T	CCDC15	Missense	p.R606L	8	134	6.0%	.	.	.
GC13	11	125447466	G	T	EI24	Missense	p.G106C	5	73	6.8%	.	.	.
GC13	11	125499317	G	T	CHEK1	Missense	p.R145M	7	97	7.2%	.	.	.

GC13	11	125514045	G	T	CHEK1	Missense	p.W344L	7	81	8.6%	.	.	.
GC13	11	125778394	C	A	DDX25	Missense	p.P54Q	6	59	10.2%	.	.	.
GC13	11	126081370	C	A	RPUSD4	Missense	p.R55L	7	85	8.2%	.	.	.
GC13	11	126277155	G	T	ST3GAL4	Missense	p.R63L	7	117	6.0%	.	.	.
GC13	11	126294738	C	A	KIRREL3	Missense	p.G680W	6	60	10.0%	.	.	.
GC13	11	128840132	C	A	ARHGAP32	Missense	p.R1296L	8	99	8.1%	.	.	.
GC13	11	128842877	G	T	ARHGAP32	Missense	p.P812Q	7	96	7.3%	.	.	.
GC13	11	129734984	C	A	NFRKB	Missense	p.L1242F	5	42	11.9%	.	.	.
GC13	11	129814734	G	T	PRDM10	Missense	p.Q146K	7	98	7.1%	.	.	.
GC13	11	130007175	C	A	APLP2	Missense	p.P565Q	7	94	7.4%	.	.	.
GC13	11	130130856	G	T	ZBTB44	Missense	p.Q305K	8	134	6.0%	.	.	.
GC13	11	130131297	C	A	ZBTB44	Missense	p.G158W	7	87	8.0%	.	.	.
GC13	11	130131339	C	A	ZBTB44	Nonsense	p.G144X	7	92	7.6%	.	.	.
GC13	11	134046202	C	A	NCAPD3	Missense	p.S1034I	7	90	7.8%	.	.	.
GC13	11	134074831	G	T	NCAPD3	Missense	p.P351Q	8	77	10.4%	.	.	.
GC13	11	134183311	C	A	GLB1L3	Missense	p.L508M	7	84	8.3%	.	.	.
GC13	11	134244507	G	T	GLB1L2	Missense	p.K573N	6	66	9.1%	.	.	.
GC13	12	301744	C	A	SLC6A12	Missense	p.W534L	6	56	10.7%	.	.	.
GC13	12	416961	G	T	KDM5A	Missense	p.Q1197K	7	105	6.7%	.	.	.
GC13	12	993389	C	A	WNK1	Missense	p.P1028H	7	95	7.4%	.	.	.
GC13	12	994351	G	T	WNK1	Missense	p.G1214W	8	118	6.8%	.	.	.
GC13	12	1006822	G	T	WNK1	Missense	p.L1893F	7	64	10.9%	.	.	.
GC13	12	1137390	G	T	ERC1	Missense	p.M107I	7	81	8.6%	.	.	.
GC13	12	1289767	G	T	ERC1	Missense	p.R600L	6	69	8.7%	.	.	.
GC13	12	1882064	G	T	ADIPOR2	Missense	p.M76I	6	51	11.8%	.	.	.
GC13	12	2062409	C	A	DCP1B	Missense	p.G233W	8	79	10.1%	.	.	.
GC13	12	2705141	C	A	CACNA1C	Missense	p.P922Q	6	81	7.4%	.	.	.
GC13	12	2797817	G	T	CACNA1C	Nonsense	p.E2005X	18	56	32.1%	.	O	.
GC13	12	2968697	C	A	FOXM1	Missense	p.G452W	7	78	9.0%	.	.	.
GC13	12	2981324	G	T	FOXM1	Missense	p.R197S	7	105	6.7%	.	.	.
GC13	12	3701483	G	T	PRMT8	Missense	p.G347W	5	58	8.6%	.	O	.

GC13	12	3921402	C	A	PARP11	Missense	p.G221W	8	110	7.3%	.	.	.
GC13	12	4459055	G	T	TIGAR	Missense	p.R88L	8	132	6.1%	.	.	.
GC13	12	4479898	C	A	FGF23	Missense	p.G123W	5	46	10.9%	.	.	O
GC13	12	4609391	C	A	C12orf4	Missense	p.M235I	7	87	8.0%	.	.	.
GC13	12	4920496	C	A	KCNA6	Missense	p.P430H	6	65	9.2%	.	.	.
GC13	12	5021463	C	A	KCNA1	Missense	p.R307S	6	70	8.6%	.	.	.
GC13	12	5672682	G	T	ANO2	Missense	p.P932H	9	98	9.2%	.	.	.
GC13	12	5908721	C	A	ANO2	Missense	p.W337L	8	111	7.2%	.	.	.
GC13	12	6131073	G	T	VWF	Missense	p.Q1223K	7	74	9.5%	.	.	.
GC13	12	6342641	C	A	CD9	Missense	p.H44N	7	89	7.9%	.	.	.
GC13	12	6436548	G	T	PLEKHG6	Missense	p.G568V	8	100	8.0%	.	.	.
GC13	12	6443314	G	T	TNFRSF1A	Missense	p.Q46K	6	81	7.4%	.	.	.
GC13	12	6494221	G	T	LTBR	Missense	p.R57L	6	93	6.5%	.	O	.
GC13	12	6562666	G	T	TAPBPL	Missense	p.G117W	7	85	8.2%	.	.	.
GC13	12	6623525	G	T	NCAPD2	Missense	p.G228C	8	106	7.5%	.	.	.
GC13	12	6682336	G	T	CHD4	Missense	p.R1814S	5	60	8.3%	O	.	.
GC13	12	6687014	C	A	CHD4	Missense	p.K1759N	9	147	6.1%	O	.	.
GC13	12	6701976	C	A	CHD4	Missense	p.R880L	6	66	9.1%	O	.	.
GC13	12	6762396	C	A	ING4	Missense	p.M68I	7	68	10.3%	.	.	.
GC13	12	6860821	C	A	MLF2	Missense	p.G68W	8	88	9.1%	.	.	.
GC13	12	6968633	G	T	USP5	Splicing	c.1059-1G>T	8	90	8.9%	.	.	.
GC13	12	7025042	G	T	ENO2	Missense	p.G16W	6	68	8.8%	.	.	.
GC13	12	7050549	G	T	ATN1	Missense	p.R1124L	9	108	8.3%	.	.	.
GC13	12	7067095	G	T	PTPN6	Missense	p.R407L	6	74	8.1%	.	.	.
GC13	12	7252309	C	A	C1RL	Missense	p.G180W	7	86	8.1%	.	.	.
GC13	12	7473400	G	T	ACSM4	Missense	p.R334I	10	96	10.4%	.	.	.
GC13	12	7477099	C	A	ACSM4	Missense	p.R481S	7	85	8.2%	.	.	.
GC13	12	7480919	G	T	ACSM4	Missense	p.G565W	9	104	8.7%	.	.	.
GC13	12	7527236	G	T	CD163L1	Missense	p.H1081N	7	69	10.1%	.	.	.
GC13	12	7528075	G	T	CD163L1	Missense	p.R945S	6	88	6.8%	.	.	.
GC13	12	7528326	G	T	CD163L1	Missense	p.H896N	7	100	7.0%	.	.	.

GC13	12	7548861	G	T	CD163L1	Missense	p.P637Q	8	89	9.0%	.	.	.
GC13	12	7640108	G	T	CD163	Missense	p.P633T	8	97	8.2%	.	.	.
GC13	12	8211383	G	T	C3AR1	Missense	p.H467N	8	63	12.7%	.	O	.
GC13	12	8211554	C	A	C3AR1	Missense	p.G410W	12	102	11.8%	.	.	.
GC13	12	8689832	C	A	CLEC4E	Missense	p.W84L	8	107	7.5%	.	.	.
GC13	12	9007422	C	A	A2ML1	Missense	p.P429Q	6	83	7.2%	.	.	.
GC13	12	9221389	G	T	A2M	Missense	p.P1288Q	9	121	7.4%	.	.	.
GC13	12	9225057	G	T	A2M	Missense	p.P1184Q	6	79	7.6%	.	O	.
GC13	12	9242963	G	T	A2M	Missense	p.P712Q	8	120	6.7%	.	.	.
GC13	12	9345200	G	T	PZP	Missense	p.L464M	6	56	10.7%	.	.	.
GC13	12	10205321	G	T	CLEC9A	Missense	p.W12L	7	96	7.3%	.	.	.
GC13	12	10224083	G	T	CLEC1A	Missense	p.P139Q	7	80	8.8%	.	.	.
GC13	12	10335155	G	T	TMEM52B	Missense	p.G21W	9	96	9.4%	.	.	.
GC13	12	10525806	C	A	KLRK1	Missense	p.K186N	8	83	9.6%	.	.	.
GC13	12	10531223	C	A	KLRK1	Missense	p.W120L	8	101	7.9%	.	.	.
GC13	12	10766107	G	T	MAGOHB	Missense	p.L9M	8	97	8.2%	.	.	.
GC13	12	10961822	C	A	TAS2R9	Nonsense	p.G285X	7	89	7.9%	.	.	.
GC13	12	10978606	C	A	TAS2R10	Missense	p.W88L	8	79	10.1%	.	.	.
GC13	12	12232606	C	A	BCL2L14	Missense	p.Q123K	7	85	8.2%	.	.	.
GC13	12	12311850	G	T	LRP6	Missense	p.H902N	7	88	8.0%	.	.	.
GC13	12	13221330	G	T	FAM234B	Missense	p.R412L	6	93	6.5%	.	.	.
GC13	12	13828725	G	T	GRIN2B	Missense	p.P360Q	8	106	7.5%	.	.	.
GC13	12	14767878	C	A	GUCY2C	Splicing	c.2971-1G>T	9	95	9.5%	.	.	.
GC13	12	14976607	G	T	C12orf60	Missense	p.X246Y	8	98	8.2%	.	.	.
GC13	12	15095545	G	T	ARHGDIB	Missense	p.H173N	5	49	10.2%	.	.	.
GC13	12	15739935	G	T	PTPRO	Missense	p.M281I	7	60	11.7%	.	.	.
GC13	12	16036497	G	T	STRAP	Missense	p.Q45H	5	34	14.7%	.	.	.
GC13	12	16342618	C	A	SLC15A5	Missense	p.W575L	8	89	9.0%	.	.	.
GC13	12	18435517	C	A	PIK3C2G	Missense	p.H168N	7	87	8.0%	.	.	.
GC13	12	18793370	C	A	PIK3C2G	Missense	p.P1356Q	7	89	7.9%	.	.	.
GC13	12	18852764	C	A	PLCZ1	Missense	p.G187W	8	110	7.3%	.	.	.

GC13	12	19436303	C	A	PLEKHA5	Missense	p.P354Q	8	104	7.7%	.	.	.
GC13	12	21033866	G	T	SLCO1B3	Missense	p.W442L	7	92	7.6%	.	.	.
GC13	12	21608118	G	T	PYROXD1	Missense	p.G110W	6	43	14.0%	.	.	.
GC13	12	22028651	C	A	ABCC9	Nonsense	p.G677X	8	127	6.3%	.	.	.
GC13	12	22061091	C	A	ABCC9	Missense	p.G459C	9	106	8.5%	.	.	.
GC13	12	22677486	G	T	C2CD5	Missense	p.P174Q	8	109	7.3%	.	.	.
GC13	12	25347895	C	A	CASC1	Missense	p.L34F	8	84	9.5%	.	.	.
GC13	12	26580988	C	A	ITPR2	Missense	p.W2268L	7	91	7.7%	.	.	.
GC13	12	26648121	G	T	ITPR2	Missense	p.L1716I	9	106	8.5%	.	.	.
GC13	12	26752233	G	T	ITPR2	Missense	p.H1283N	7	86	8.1%	.	.	.
GC13	12	29614858	G	T	OVCH1	Missense	p.H737N	6	88	6.8%	.	.	.
GC13	12	29648293	G	T	OVCH1	Missense	p.H127N	7	97	7.2%	.	.	.
GC13	12	30869611	C	A	CAPRIN2	Splicing	c.2053-1G>T;NM_001206856	6	51	11.8%	.	O	.
GC13	12	31446819	G	T	FAM60A	Missense	p.P92Q	5	35	14.3%	.	.	.
GC13	12	31566451	C	A	DENND5B	Missense	p.R867L	9	103	8.7%	.	.	.
GC13	12	31604927	G	T	DENND5B	Missense	p.Q526K	7	100	7.0%	.	.	.
GC13	12	32138130	C	A	KIAA1551	Missense	p.P1414Q	9	115	7.8%	.	.	.
GC13	12	32487579	C	A	BICD1	Missense	p.L744M	7	95	7.4%	.	.	.
GC13	12	32903728	C	A	YARS2	Missense	p.R343L	6	93	6.5%	.	.	.
GC13	12	39726206	C	A	KIF21A	Missense	p.R918L	9	130	6.9%	.	O	.
GC13	12	39980073	C	A	ABCD2	Missense	p.R558L	8	92	8.7%	.	.	.
GC13	12	41900264	G	T	PDZRN4	Missense	p.G26W	8	85	9.4%	.	.	.
GC13	12	42839917	G	T	PPHLN1	Missense	p.R368L	5	41	12.2%	.	.	.
GC13	12	43748028	G	T	ADAMTS20	Missense	p.P1906Q	8	87	9.2%	.	.	.
GC13	12	43822472	G	T	ADAMTS20	Missense	p.P1207H	7	87	8.0%	.	.	.
GC13	12	43826580	G	T	ADAMTS20	Missense	p.Q919K	7	94	7.4%	.	.	.
GC13	12	44171518	G	T	IRAK4	Missense	p.G144C	8	125	6.4%	.	.	.
GC13	12	45761576	G	T	ANO6	Missense	p.K368N	7	81	8.6%	.	.	.
GC13	12	46244575	G	T	ARID2	Missense	p.R890M	8	129	6.2%	O	.	.
GC13	12	46246343	G	T	ARID2	Missense	p.Q1479H	8	106	7.5%	O	.	.
GC13	12	46285701	G	T	ARID2	Missense	p.Q1687H	8	109	7.3%	O	.	.

GC13	12	46320776	G	T	SCAF11	Missense	p.P903Q	8	132	6.1%	.	.	.
GC13	12	46320777	G	T	SCAF11	Missense	p.P903T	8	132	6.1%	.	.	.
GC13	12	46756306	G	T	SLC38A2	Missense	p.P332Q	8	98	8.2%	.	.	.
GC13	12	47181751	C	A	SLC38A4	Missense	p.G92W	8	100	8.0%	.	.	.
GC13	12	47471345	C	A	AMIGO2	Missense	p.G481W	7	108	6.5%	.	.	.
GC13	12	48073354	C	A	RPAP3	Missense	p.W244C	8	117	6.8%	.	.	.
GC13	12	48104635	C	A	ENDOU	Missense	p.G332W	7	93	7.5%	.	.	.
GC13	12	48143252	C	A	RAPGEF3	Missense	p.R279L	4	37	10.8%	.	.	.
GC13	12	48359672	G	T	TMEM106C	Missense	p.L101F	7	95	7.4%	.	.	.
GC13	12	49075378	C	A	KANSL2	Missense	p.R13L	7	103	6.8%	.	.	.
GC13	12	49087874	G	T	CCNT1	Missense	p.Q375K	8	125	6.4%	.	.	.
GC13	12	49168252	C	A	ADCY6	Missense	p.R739L	8	98	8.2%	.	.	.
GC13	12	49168545	C	A	ADCY6	Missense	p.G699W	7	76	9.2%	.	.	.
GC13	12	49391317	G	T	DDN	Missense	p.R448S	6	51	11.8%	.	.	.
GC13	12	49435458	G	T	KMT2D	Missense	p.R2072S	5	47	10.6%	O	O	.
GC13	12	49448334	G	T	KMT2D	Missense	p.P126H	8	82	9.8%	O	.	.
GC13	12	49483839	C	A	DHH	Missense	p.G332W	5	45	11.1%	.	.	.
GC13	12	49496711	G	T	LMBR1L	Missense	p.R219S	5	48	10.4%	.	O	.
GC13	12	49743434	G	T	DNAJC22	Missense	p.W260L	7	97	7.2%	.	.	.
GC13	12	49934708	C	A	KCNH3	Missense	p.Q35K	7	80	8.8%	.	.	.
GC13	12	49994284	C	A	FAM186B	Missense	p.R380L	6	85	7.1%	.	.	.
GC13	12	50029239	C	A	PRPF40B	Missense	p.L392M	7	73	9.6%	.	.	.
GC13	12	50031280	G	T	PRPF40B	Missense	p.G502W	6	43	14.0%	.	.	.
GC13	12	50368589	G	T	AQP6	Missense	p.G208W	8	53	15.1%	.	.	.
GC13	12	50388268	G	T	RACGAP1	Missense	p.Q299K	9	92	9.8%	.	.	.
GC13	12	50472707	G	T	ASIC1	Missense	p.G366V	7	85	8.2%	.	.	.
GC13	12	50528456	C	A	CERS5	Missense	p.W301L	8	81	9.9%	.	.	.
GC13	12	50615817	G	T	LIMA1	Missense	p.P46Q	7	81	8.6%	.	.	.
GC13	12	50724482	G	T	FAM186A	Missense	p.P2303Q	7	94	7.4%	.	.	.
GC13	12	50745518	C	A	FAM186A	Missense	p.L1699F	9	63	14.3%	.	.	.
GC13	12	50747827	G	T	FAM186A	Missense	p.Q930K	11	142	7.7%	.	.	.

GC13	12	51034615	G	T	DIP2B	Missense	p.R94M	7	61	11.5%	.	.	.
GC13	12	51072582	G	T	DIP2B	Missense	p.W346L	6	63	9.5%	.	.	.
GC13	12	51382125	G	T	SLC11A2	Missense	p.R553S	10	86	11.6%	.	.	.
GC13	12	51458274	G	T	CSRNP2	Missense	p.P296Q	8	89	9.0%	.	.	.
GC13	12	52182528	G	T	SCN8A	Missense	p.R1385L	6	96	6.3%	.	.	.
GC13	12	52385720	G	T	ACVR1B	Missense	p.K445N	8	119	6.7%	.	.	.
GC13	12	52946818	C	A	KRT71	Missense	p.G15V	7	95	7.4%	.	.	.
GC13	12	52967147	G	T	KRT74	Missense	p.Q139K	5	40	12.5%	.	.	.
GC13	12	53045487	G	T	KRT2	Missense	p.P147H	7	56	12.5%	.	.	.
GC13	12	53165693	G	T	KRT76	Missense	p.Q409K	6	47	12.8%	.	.	.
GC13	12	53170944	A	T	KRT76	Nonsense	p.C44X	17	74	23.0%	.	.	.
GC13	12	53189259	G	T	KRT3	Missense	p.Q190K	5	35	14.3%	.	O	.
GC13	12	53189793	C	A	KRT3	Missense	p.G12W	7	103	6.8%	.	.	.
GC13	12	53201456	G	T	KRT4	Missense	p.R440S	5	32	15.6%	.	.	.
GC13	12	53449006	C	A	TNS2	Missense	p.R197S	7	100	7.0%	.	.	.
GC13	12	53457566	G	T	TNS2	Missense	p.W1387L	7	89	7.9%	.	.	.
GC13	12	53459720	C	A	SPRYD3	Missense	p.G409W	7	72	9.7%	.	.	.
GC13	12	53468441	C	A	SPRYD3	Missense	p.G167W	9	117	7.7%	.	.	.
GC13	12	53646956	C	A	MFSD5	Missense	p.L220M	8	115	7.0%	.	.	.
GC13	12	53684721	C	A	ESPL1	Missense	p.R1821S	4	31	12.9%	.	.	.
GC13	12	53687080	G	T	ESPL1	Missense	p.W2062L	9	107	8.4%	.	.	.
GC13	12	53699743	C	A	C12orf10	Missense	p.Q181K	6	66	9.1%	.	.	.
GC13	12	53723125	C	A	SP7	Missense	p.R34L	6	65	9.2%	.	.	.
GC13	12	53776515	G	T	SP1	Missense	p.G214W	9	114	7.9%	.	.	.
GC13	12	53777109	G	T	SP1	Nonsense	p.G412X	8	95	8.4%	.	O	.
GC13	12	53862568	G	T	PCBP2	Missense	p.L234F	8	138	5.8%	.	.	.
GC13	12	53877738	G	T	MAP3K12	Missense	p.R439S	7	77	9.1%	.	.	.
GC13	12	53900571	C	A	NPFF	Missense	p.G114W	7	91	7.7%	.	.	.
GC13	12	53925665	G	T	ATF7	Missense	p.L243I	7	57	12.3%	.	.	.
GC13	12	54426979	G	T	HOXC5	Missense	p.G25W	9	80	11.3%	.	.	.
GC13	12	54427360	G	T	HOXC5	Nonsense	p.E152X	5	48	10.4%	.	.	.

GC13	12	54575311	G	T	SMUG1	Missense	p.Q176K	7	77	9.1%	.	.	.
GC13	12	54575959	G	T	SMUG1	Missense	p.P143Q	6	56	10.7%	.	.	.
GC13	12	54734340	C	A	COPZ1	Missense	p.L10M	7	92	7.6%	.	.	.
GC13	12	54764456	G	T	ZNF385A	Missense	p.P194Q	7	75	9.3%	.	.	.
GC13	12	54920429	G	T	NCKAP1L	Missense	p.L708F	7	81	8.6%	.	.	.
GC13	12	54963338	G	T	PDE1B	Missense	p.R120L	7	103	6.8%	.	.	.
GC13	12	54969334	G	T	PDE1B	Splicing	c.775-1G>T;NM_001288769	8	101	7.9%	.	.	.
GC13	12	55615262	G	T	OR10A7	Missense	p.G152C	8	101	7.9%	.	.	.
GC13	12	55641186	G	T	OR6C74	Missense	p.G39W	9	126	7.1%	.	.	.
GC13	12	56213208	G	T	ORMDL2	Missense	p.W86L	12	114	10.5%	.	.	.
GC13	12	56221036	C	A	DNAJC14	Missense	p.M469I	8	99	8.1%	.	.	.
GC13	12	56334137	G	T	DGKA	Missense	p.G280W	10	95	10.5%	.	.	.
GC13	12	56351153	C	A	PMEL	Missense	p.G226W	6	56	10.7%	.	.	.
GC13	12	56493811	G	T	ERBB3	Missense	p.G1043W	8	91	8.8%	O	.	.
GC13	12	56536817	G	T	ESYT1	Nonsense	p.G1012X	7	87	8.0%	.	.	.
GC13	12	56572811	G	T	SMARCC2	Missense	p.P371T	7	71	9.9%	.	.	.
GC13	12	56604114	G	T	RNF41	Missense	p.P110Q	7	41	17.1%	.	.	.
GC13	12	56630387	G	T	SLC39A5	Missense	p.R355M	7	69	10.1%	.	.	.
GC13	12	56718105	G	T	PAN2	Missense	p.P634Q	7	73	9.6%	.	.	.
GC13	12	56740261	C	A	STAT2	Missense	p.R670L	7	87	8.0%	.	.	.
GC13	12	56740351	G	T	STAT2	Missense	p.P640Q	7	63	11.1%	.	.	.
GC13	12	56846903	G	T	MIP	Missense	p.R187S	7	69	10.1%	.	.	.
GC13	12	56915835	G	T	RBMS2	Missense	p.G10W	6	83	7.2%	.	.	.
GC13	12	56992970	G	T	BAZ2A	Missense	p.P1782H	6	54	11.1%	.	.	.
GC13	12	56994205	G	T	BAZ2A	Missense	p.Q1558K	7	75	9.3%	.	.	.
GC13	12	57005633	C	A	BAZ2A	Missense	p.K511N	7	88	8.0%	.	.	.
GC13	12	57008915	G	T	BAZ2A	Missense	p.Q205K	8	105	7.6%	.	.	.
GC13	12	57036471	G	T	ATP5B	Missense	p.Q313K	12	106	11.3%	.	.	.
GC13	12	57036581	G	T	ATP5B	Missense	p.P276H	7	84	8.3%	.	.	.
GC13	12	57389526	G	T	GPR182	Missense	p.W178L	6	59	10.2%	.	.	.
GC13	12	57437136	C	A	MYO1A	Missense	p.R300L	8	107	7.5%	.	.	.

GC13	12	57487333	C	A	NAB2	Missense	p.R474S	4	27	14.8%	.	.	.
GC13	12	57577249	C	A	LRP1	Missense	p.P1917Q	8	39	20.5%	.	.	.
GC13	12	57666236	C	A	R3HDM2	Missense	p.M450I	7	79	8.9%	.	.	.
GC13	12	57864789	G	T	GLI1	Missense	p.G628C	6	44	13.6%	.	.	.
GC13	12	57920051	G	T	MBD6	Missense	p.G434W	9	84	10.7%	.	.	.
GC13	12	58001107	G	T	DTX3	Missense	p.R157L	6	47	12.8%	.	.	.
GC13	12	58001202	G	A	DTX3	Missense	p.E189K	19	68	27.9%	.	.	.
GC13	12	58009436	C	A	ARHGEF25	Missense	p.L364M	7	87	8.0%	.	.	.
GC13	12	58015179	C	A	SLC26A10	Missense	p.P175Q	6	58	10.3%	.	.	.
GC13	12	58023911	C	A	B4GALNT1	Missense	p.G246W	7	87	8.0%	.	.	.
GC13	12	58160775	C	A	CYP27B1	Missense	p.W17L	6	47	12.8%	.	.	.
GC13	12	58163383	G	T	METTL1	Missense	p.P118H	7	80	8.8%	.	.	.
GC13	12	58189999	G	T	TSFM	Missense	p.W204L	8	81	9.9%	.	.	.
GC13	12	59271431	C	A	LRIG3	Missense	p.G703W	8	116	6.9%	.	.	.
GC13	12	62783284	C	A	USP15	Missense	p.P267H	8	129	6.2%	.	.	.
GC13	12	62954758	G	T	MON2	Missense	p.L1299F	8	137	5.8%	.	.	.
GC13	12	64509673	G	T	SRGAP1	Nonsense	p.E719X	6	51	11.8%	.	.	.
GC13	12	64679748	C	A	C12orf56	Missense	p.R242S	8	141	5.7%	.	.	.
GC13	12	64828689	G	T	XPOT	Missense	p.L895F	8	137	5.8%	.	.	.
GC13	12	64891533	G	T	TBK1	Missense	p.G689C	9	108	8.3%	.	.	.
GC13	12	65260585	C	A	TBC1D30	Missense	p.P398Q	7	69	10.1%	.	.	.
GC13	12	65564766	C	A	LEMD3	Missense	p.P464T	8	120	6.7%	.	.	.
GC13	12	65856950	G	T	MSRB3	Missense	p.G143W	7	88	8.0%	.	.	.
GC13	12	66800204	C	A	GRIP1	Missense	p.G563W	6	80	7.5%	.	.	.
GC13	12	66859182	C	A	GRIP1	Missense	p.G249W	7	77	9.1%	.	.	.
GC13	12	68051239	G	T	DYRK2	Missense	p.L111F	6	67	9.0%	.	O	.
GC13	12	68720501	G	T	MDM1	Missense	p.P145Q	8	90	8.9%	.	.	.
GC13	12	69120332	G	T	NUP107	Nonsense	p.G515X	8	137	5.8%	.	.	.
GC13	12	69135730	G	T	NUP107	Missense	p.M851I	8	149	5.4%	.	.	.
GC13	12	69653964	G	T	CPSF6	Missense	p.G486C	8	83	9.6%	.	O	.
GC13	12	69964132	G	T	FRS2	Missense	p.G30W	7	87	8.0%	.	.	.

GC13	12	69964192	C	A	FRS2	Missense	p.R50S	8	105	7.6%	.	.	.
GC13	12	69991417	G	T	CCT2	Splicing	c.962-1G>T	9	118	7.6%	.	.	.
GC13	12	70049296	C	A	BEST3	Missense	p.M253I	6	56	10.7%	.	O	.
GC13	12	70729260	C	A	CNOT2	Missense	p.P231Q	7	80	8.8%	.	.	.
GC13	12	70989939	C	A	PTPRB	Missense	p.W165L	8	115	7.0%	O	.	.
GC13	12	71519125	C	A	TSPAN8	Missense	p.G235W	7	97	7.2%	.	.	.
GC13	12	71960215	G	T	LGR5	Missense	p.G273W	8	76	10.5%	.	.	.
GC13	12	72290515	G	T	TBC1D15	Missense	p.W348L	6	80	7.5%	.	.	.
GC13	12	75444464	C	A	KCNC2	Missense	p.G441W	7	85	8.2%	.	.	.
GC13	12	75692715	C	A	CAPS2	Missense	p.G83W	6	59	10.2%	.	.	.
GC13	12	75804218	G	T	GLIPR1L2	Missense	p.W80L	7	73	9.6%	.	.	.
GC13	12	75807421	C	A	GLIPR1L2	Missense	p.P175Q	8	136	5.9%	.	.	.
GC13	12	75824628	G	T	GLIPR1L2	Missense	p.W241L	7	76	9.2%	.	.	.
GC13	12	75900278	C	A	KRR1	Nonsense	p.G169X	7	71	9.9%	.	.	.
GC13	12	76741020	G	T	BBS10	Missense	p.R249S	7	83	8.4%	.	O	.
GC13	12	76741374	G	T	BBS10	Missense	p.Q131K	9	95	9.5%	.	.	.
GC13	12	77259947	G	T	CSRP2	Missense	p.R32S	4	35	11.4%	.	.	.
GC13	12	77423946	C	A	E2F7	Missense	p.G517C	7	97	7.2%	.	.	.
GC13	12	78401005	C	A	NAV3	Missense	p.Q563K	8	113	7.1%	.	.	.
GC13	12	78443862	G	T	NAV3	Missense	p.G705W	7	91	7.7%	.	.	.
GC13	12	78522645	G	T	NAV3	Missense	p.L1480F	7	91	7.7%	.	.	.
GC13	12	78583826	C	A	NAV3	Missense	p.Q2018K	7	89	7.9%	.	.	.
GC13	12	80672010	G	T	OTOGL	Missense	p.W906L	8	109	7.3%	.	.	.
GC13	12	80750685	C	A	OTOGL	Missense	p.P1995T	7	98	7.1%	.	.	.
GC13	12	80899878	C	A	PTPRQ	Missense	p.P611Q	8	92	8.7%	.	.	.
GC13	12	81062865	G	T	PTPRQ	Missense	p.W1919L	9	110	8.2%	.	.	.
GC13	12	81741445	G	T	PPFIA2	Missense	p.P267Q	6	85	7.1%	.	O	.
GC13	12	85279252	C	A	SLC6A15	Missense	p.W72L	7	62	11.3%	.	.	.
GC13	12	88380121	G	T	C12orf50	Missense	p.P297Q	7	88	8.0%	.	.	.
GC13	12	88535027	G	T	CEP290	Missense	p.R20S	6	97	6.2%	.	.	.
GC13	12	88570084	C	A	TMTC3	Missense	p.H474N	6	74	8.1%	.	.	.

GC13	12	89917252	G	T	GALNT4	Missense	p.H359N	7	86	8.1%	.	.	.
GC13	12	89917260	G	T	GALNT4	Missense	p.P356Q	8	86	9.3%	.	O	.
GC13	12	90036033	C	A	ATP2B1	Missense	p.W103L	6	74	8.1%	.	.	.
GC13	12	91540020	G	T	DCN	Missense	p.P74H	6	56	10.7%	.	.	.
GC13	12	92538076	G	T	BTG1	Missense	p.P99Q	8	105	7.6%	.	.	.
GC13	12	94772623	G	T	CEP83	Missense	p.Q249K	7	92	7.6%	.	.	.
GC13	12	95927684	C	A	USP44	Missense	p.G117W	9	113	8.0%	.	.	.
GC13	12	96181159	C	A	NTN4	Missense	p.W48L	7	73	9.6%	.	.	.
GC13	12	96371766	C	A	HAL	Missense	p.W329L	5	31	16.1%	.	.	.
GC13	12	96921087	C	A	CFAP54	Missense	p.Q408K	8	114	7.0%	.	.	.
GC13	12	97313892	G	T	NEDD1	Missense	p.G71C	7	68	10.3%	.	O	.
GC13	12	98909733	G	T	TMPO	Missense	p.G30W	5	62	8.1%	.	.	.
GC13	12	98987785	G	T	SLC25A3	Missense	p.R10L	6	54	11.1%	.	.	.
GC13	12	99076939	G	T	APAF1	Missense	p.G678W	7	96	7.3%	.	.	.
GC13	12	100169338	G	T	ANKS1B	Missense	p.Q317K	5	38	13.2%	.	.	.
GC13	12	100489540	G	T	UHRF1BP1L	Missense	p.Q249K	13	120	10.8%	.	.	.
GC13	12	100691871	G	T	SCYL2	Missense	p.W133L	8	99	8.1%	.	.	.
GC13	12	100711627	C	A	SCYL2	Missense	p.T440N	5	77	6.5%	.	.	.
GC13	12	100723013	G	T	SCYL2	Missense	p.L559F	11	119	9.2%	.	.	.
GC13	12	101493427	C	A	ANO4	Missense	p.P693Q	8	104	7.7%	.	.	.
GC13	12	101746885	G	T	UTP20	Missense	p.M1695I	9	118	7.6%	.	.	.
GC13	12	101779384	C	A	UTP20	Missense	p.Q2694K	8	103	7.8%	.	.	.
GC13	12	102107937	G	T	CHPT1	Missense	p.G115W	7	86	8.1%	.	.	.
GC13	12	102147211	G	T	GNPTAB	Missense	p.Q1181K	7	85	8.2%	.	.	.
GC13	12	102159046	G	T	GNPTAB	Missense	p.P550Q	8	122	6.6%	.	.	.
GC13	12	102471118	C	A	NUP37	Missense	p.W235L	7	100	7.0%	.	.	.
GC13	12	103246680	C	A	PAH	Missense	p.R252L	4	31	12.9%	.	.	.
GC13	12	104033928	C	A	STAB2	Missense	p.Q312K	7	74	9.5%	.	.	.
GC13	12	104054108	G	T	STAB2	Splicing	c.1735-1G>T	8	89	9.0%	.	.	.
GC13	12	104086623	G	T	STAB2	Missense	p.G1111W	8	75	10.7%	.	O	.
GC13	12	104096994	G	T	STAB2	Missense	p.L1261F	6	78	7.7%	.	.	.

GC13	12	104099451	G	T	STAB2	Missense	p.M1314I	6	73	8.2%	.	.	.
GC13	12	104333309	G	T	HSP90B1	Missense	p.W333L	7	83	8.4%	.	.	.
GC13	12	104514222	C	A	NFYB	Missense	p.G183C	5	36	13.9%	.	.	.
GC13	12	105255144	C	A	SLC41A2	Missense	p.G394W	6	64	9.4%	.	.	.
GC13	12	105569840	C	A	APPL2	Missense	p.L617F	8	92	8.7%	.	O	.
GC13	12	106461298	G	T	NUAK1	Missense	p.P423H	8	109	7.3%	.	.	.
GC13	12	106734671	C	A	TCP11L2	Missense	p.L404M	10	131	7.6%	.	.	.
GC13	12	106751719	C	A	POLR3B	Missense	p.P21Q	4	31	12.9%	.	.	.
GC13	12	107268284	C	A	RIC8B	Missense	p.Q533K	7	79	8.9%	.	.	.
GC13	12	107372188	G	T	MTERF2	Missense	p.P102Q	8	103	7.8%	.	.	.
GC13	12	107937868	G	T	BTBD11	Missense	p.W481L	7	99	7.1%	.	.	.
GC13	12	108133284	G	T	PRDM4	Missense	p.Q657K	7	87	8.0%	.	.	.
GC13	12	108618518	G	T	WSCD2	Nonsense	p.G229X	11	112	9.8%	.	.	.
GC13	12	108620899	G	T	WSCD2	Missense	p.G313W	7	63	11.1%	.	.	.
GC13	12	108685782	C	A	CMKLR1	Missense	p.G320C	7	97	7.2%	.	.	.
GC13	12	108686070	G	T	CMKLR1	Missense	p.R224S	6	50	12.0%	.	.	.
GC13	12	108925042	C	A	SART3	Missense	p.W562L	8	123	6.5%	.	.	.
GC13	12	109205037	C	A	SSH1	Missense	p.G168C	8	93	8.6%	.	.	.
GC13	12	109290863	G	T	DAO	Missense	p.G232W	8	88	9.1%	.	.	.
GC13	12	109294227	G	T	DAO	Missense	p.W320C	8	56	14.3%	.	.	.
GC13	12	109294258	G	T	DAO	Missense	p.G331W	7	63	11.1%	.	.	.
GC13	12	109629461	T	A	ACACB	Missense	p.F730I	10	47	21.3%	.	.	.
GC13	12	109835562	G	T	MYO1H	Missense	p.R156L	7	112	6.3%	.	.	.
GC13	12	109847428	G	T	MYO1H	Missense	p.G392C	11	113	9.7%	.	.	.
GC13	12	110231375	C	A	TRPV4	Nonsense	p.G432X	6	69	8.7%	.	.	.
GC13	12	110342585	C	A	TCHP	Missense	p.P148Q	8	99	8.1%	.	.	.
GC13	12	110480257	C	A	C12orf76	Missense	p.G103W	6	60	10.0%	.	.	.
GC13	12	110765754	G	T	ATP2A2	Missense	p.G343C	9	93	9.7%	.	.	.
GC13	12	110783085	C	A	ATP2A2	Missense	p.P880Q	6	91	6.6%	.	.	.
GC13	12	110783844	G	T	ATP2A2	Missense	p.W927L	7	82	8.5%	.	.	.
GC13	12	110895376	C	A	GPN3	Missense	p.W169L	8	66	12.1%	.	.	.

GC13	12	110924338	G	T	FAM216A	Splicing	c.437-1G>T	7	76	9.2%	.	.	.
GC13	12	112460232	C	A	ERP29	Missense	p.L188I	7	83	8.4%	.	.	.
GC13	12	112477124	C	A	NAA25	Missense	p.W853L	6	81	7.4%	.	.	.
GC13	12	112516028	G	T	NAA25	Missense	p.Q210K	8	94	8.5%	.	.	.
GC13	12	112622413	C	A	HECTD4	Missense	p.G3319W	6	61	9.8%	.	.	.
GC13	12	112673472	C	A	HECTD4	Missense	p.R1720L	8	97	8.2%	.	.	.
GC13	12	112747463	C	A	HECTD4	Missense	p.L165F	5	36	13.9%	.	.	.
GC13	12	113307588	C	A	RPH3A	Missense	p.P145Q	13	94	13.8%	.	.	.
GC13	12	113357251	G	T	OAS1	Missense	p.G366C	5	42	11.9%	.	.	.
GC13	12	113405759	G	T	OAS3	Missense	p.G962W	7	76	9.2%	.	.	.
GC13	12	113596771	G	T	DDX54	Missense	p.R854S	6	52	11.5%	.	.	.
GC13	12	113617798	G	T	DDX54	Missense	p.P106Q	7	79	8.9%	.	.	.
GC13	12	113623154	G	T	DDX54	Missense	p.Q35K	5	43	11.6%	.	.	.
GC13	12	113629399	G	T	RITA1	Missense	p.R220L	5	68	7.4%	.	.	.
GC13	12	113836362	G	T	SDS	Missense	p.P128Q	5	39	12.8%	.	.	.
GC13	12	114404004	G	T	RBM19	Missense	p.P10Q	8	98	8.2%	.	.	.
GC13	12	116445230	G	T	MED13L	Missense	p.L742M	7	86	8.1%	.	.	.
GC13	12	116445327	C	A	MED13L	Missense	p.L709F	7	81	8.6%	.	.	.
GC13	12	116675399	G	T	MED13L	Missense	p.R62S	8	107	7.5%	.	.	.
GC13	12	117013842	C	A	MAP1LC3B2	Missense	p.P32Q	6	75	8.0%	.	.	.
GC13	12	117583992	G	T	FBXO21	Missense	p.P589Q	7	59	11.9%	.	.	.
GC13	12	117914270	C	A	KSR2	Missense	p.G832C	7	64	10.9%	.	.	.
GC13	12	119617182	G	T	HSPB8	Missense	p.R22L	6	72	8.3%	.	.	.
GC13	12	119937948	G	T	CCDC60	Missense	p.W208L	7	55	12.7%	.	.	.
GC13	12	120173008	C	A	CIT	Missense	p.R996L	5	46	10.9%	.	.	.
GC13	12	120502562	G	T	BICDL1	Missense	p.R263L	7	87	8.0%	.	.	.
GC13	12	120509546	C	A	BICDL1	Missense	p.L344I	7	52	13.5%	.	.	.
GC13	12	120907373	C	A	SRSF9	Missense	p.G14W	6	34	17.6%	.	.	.
GC13	12	121880281	G	T	KDM2B	Missense	p.P919Q	4	29	13.8%	.	.	.
GC13	12	122064956	G	T	ORAI1	Missense	p.M103I	5	26	19.2%	.	.	.
GC13	12	122246177	C	A	SETD1B	Missense	p.P203Q	8	64	12.5%	.	.	.

GC13	12	122252612	G	T	SETD1B	Missense	p.G831W	6	72	8.3%	.	.	.
GC13	12	122622102	G	T	MLXIP	Missense	p.G707W	7	85	8.2%	.	.	.
GC13	12	122702926	G	T	DIABLO	Missense	p.L15I	9	91	9.9%	.	.	.
GC13	12	123068859	G	T	KNTC1	Missense	p.G1100W	8	67	11.9%	.	.	.
GC13	12	123424716	C	A	ABCB9	Missense	p.R499L	6	79	7.6%	.	.	.
GC13	12	123497160	C	A	PITPNM2	Missense	p.D139Y	5	43	11.6%	.	.	.
GC13	12	123825542	G	T	SBNO1	Missense	p.P215Q	9	86	10.5%	.	.	.
GC13	12	123960263	C	A	RILPL1	Nonsense	p.G368X	7	88	8.0%	.	.	.
GC13	12	124242473	G	T	ATP6V0A2	Splicing	c.2466-1G>T	6	39	15.4%	.	.	.
GC13	12	125270987	C	A	SCARB1	Missense	p.K439N	7	83	8.4%	.	.	.
GC13	12	126135237	G	T	TMEM132B	Missense	p.R58L	6	64	9.4%	.	.	.
GC13	12	129190764	G	T	TMEM132C	Missense	p.W1084L	6	53	11.3%	.	.	.
GC13	12	130015691	C	A	TMEM132D	Missense	p.W343L	5	29	17.2%	.	.	.
GC13	12	131453557	C	A	ADGRD1	Missense	p.R75S	6	75	8.0%	.	.	.
GC13	12	132204068	C	A	SFSWAP	Missense	p.P197Q	7	101	6.9%	.	.	.
GC13	12	132425846	G	T	PUS1	Missense	p.R157L	5	46	10.9%	.	.	.
GC13	12	132466984	G	T	EP400	Missense	p.K630N	7	73	9.6%	.	.	.
GC13	12	133198064	G	T	P2RX2	Missense	p.G262W	8	74	10.8%	.	.	.
GC13	12	133233834	G	T	POLE	Missense	p.P1157Q	7	80	8.8%	O	O	.
GC13	12	133250291	C	A	POLE	Missense	p.W410L	8	92	8.7%	O	.	.
GC13	12	133311085	G	T	ANKLE2	Missense	p.Q593K	7	56	12.5%	.	.	.
GC13	12	133351864	G	T	GOLGA3	Missense	p.Q1336K	7	70	10.0%	.	.	.
GC13	12	133360653	G	T	GOLGA3	Missense	p.R1122S	4	28	14.3%	.	O	.
GC13	12	133682782	G	T	ZNF140	Missense	p.G204W	7	73	9.6%	.	.	.
GC13	13	20221217	C	A	MPHOSPH8	Missense	p.P335Q	6	51	11.8%	.	.	.
GC13	13	20399026	C	A	ZMYM5	Missense	p.R534L	6	81	7.4%	.	.	.
GC13	13	20425560	G	T	ZMYM5	Missense	p.P174H	10	77	13.0%	.	.	.
GC13	13	20632844	G	T	ZMYM2	Missense	p.D875Y	9	102	8.8%	.	.	.
GC13	13	20797635	C	A	GJB6	Splicing	.	7	100	7.0%	.	.	.
GC13	13	21375050	G	T	XPO4	Missense	p.Q633K	9	100	9.0%	.	.	.
GC13	13	23824830	G	T	SGCG	Missense	p.G120V	9	95	9.5%	.	.	.

GC13	13	23904372	G	T	SACS	Missense	p.P4401Q	7	96	7.3%	.	.	.
GC13	13	23911620	G	T	SACS	Missense	p.P1985H	7	92	7.6%	.	.	.
GC13	13	23913156	G	T	SACS	Missense	p.P1473Q	7	81	8.6%	.	.	.
GC13	13	23914874	C	A	SACS	Missense	p.M900I	7	95	7.4%	.	.	.
GC13	13	23915694	G	T	SACS	Missense	p.P627Q	7	96	7.3%	.	.	.
GC13	13	25009356	C	A	PARP4	Missense	p.W1308L	7	68	10.3%	.	.	.
GC13	13	25457439	C	A	CENPJ	Missense	p.R1298L	6	78	7.7%	.	.	.
GC13	13	25826100	G	T	MTMR6	Missense	p.L457M	8	77	10.4%	.	.	.
GC13	13	25842028	C	A	MTMR6	Nonsense	p.G65X	7	96	7.3%	.	.	.
GC13	13	25882032	G	T	NUP58	Missense	p.G66W	6	81	7.4%	.	.	.
GC13	13	25914190	C	A	NUP58	Missense	p.P561H	9	150	6.0%	.	.	.
GC13	13	26043204	C	A	ATP8A2	Missense	p.R16S	6	65	9.2%	.	.	.
GC13	13	26789594	G	T	RNF6	Missense	p.P142Q	7	109	6.4%	.	.	.
GC13	13	26927998	G	T	CDK8	Missense	p.W146L	7	96	7.3%	.	.	.
GC13	13	26967533	G	T	CDK8	Nonsense	p.E53X	6	78	7.7%	.	O	.
GC13	13	27847197	C	A	RASL11A	Missense	p.L57M	7	80	8.8%	.	.	.
GC13	13	28622419	C	A	FLT3	Nonsense	p.G400X	8	124	6.5%	O	.	.
GC13	13	28752017	C	A	PAN3	Missense	p.P212H	8	106	7.5%	.	.	.
GC13	13	28771437	C	A	PAN3	Missense	p.P269H	7	74	9.5%	.	.	.
GC13	13	28844898	G	T	PAN3	Missense	p.W618L	7	92	7.6%	.	.	.
GC13	13	29001360	C	A	FLT1	Missense	p.G458C	9	89	10.1%	.	.	.
GC13	13	29599088	G	T	MTUS2	Missense	p.G95W	10	123	8.1%	.	.	.
GC13	13	29600588	C	A	MTUS2	Missense	p.P595T	8	82	9.8%	.	.	.
GC13	13	29674997	C	A	MTUS2	Missense	p.P855Q	6	78	7.7%	.	.	.
GC13	13	30110057	C	A	SLC7A1	Missense	p.R90L	6	67	9.0%	.	.	.
GC13	13	30423659	G	T	UBL3	Missense	p.P6Q	9	127	7.1%	.	.	.
GC13	13	30801554	G	T	KATNAL1	Missense	p.P381Q	7	80	8.8%	.	.	.
GC13	13	31231704	G	T	USPL1	Missense	p.W168L	7	85	8.2%	.	.	.
GC13	13	31233098	C	A	USPL1	Missense	p.L633M	8	105	7.6%	.	.	.
GC13	13	31725843	G	T	HSPH1	Missense	p.P189Q	7	74	9.5%	.	.	.
GC13	13	32360519	G	T	RXFP2	Splicing	c.930-1G>T	8	73	11.0%	.	.	.

GC13	13	32735351	G	T	FRY	Missense	p.G619W	12	142	8.5%	.	.	.
GC13	13	32783101	C	A	FRY	Missense	p.P1377Q	6	59	10.2%	.	.	.
GC13	13	32821604	G	T	FRY	Missense	p.G2325W	6	64	9.4%	.	.	.
GC13	13	32885843	G	T	ZAR1L	Missense	p.Q74K	6	47	12.8%	.	.	.
GC13	13	32910602	C	A	BRCA2	Missense	p.P704T	9	132	6.8%	O	.	.
GC13	13	32954181	G	T	BRCA2	Missense	p.R3052L	8	90	8.9%	O	.	O
GC13	13	33017125	G	T	N4BP2L2	Missense	p.L502M	8	100	8.0%	.	.	.
GC13	13	33252985	C	A	PDS5B	Missense	p.H326N	9	133	6.8%	.	.	.
GC13	13	33638000	G	T	KL	Missense	p.G906C	8	123	6.5%	.	.	.
GC13	13	33703646	G	T	STARD13	Missense	p.Q382K	8	78	10.3%	.	.	.
GC13	13	34404099	C	A	RFC3	Missense	p.P173Q	8	54	14.8%	.	.	.
GC13	13	35733843	G	T	NBEA	Missense	p.G1179C	8	122	6.6%	.	.	.
GC13	13	35738575	C	A	NBEA	Missense	p.Q1388K	10	95	10.5%	.	.	.
GC13	13	35751233	G	T	NBEA	Missense	p.R1552L	11	111	9.9%	.	.	.
GC13	13	36026288	C	A	NBEA	Missense	p.Q2122K	8	116	6.9%	.	.	.
GC13	13	36158093	G	T	NBEA	Missense	p.W158L	9	92	9.8%	.	.	.
GC13	13	36886507	C	A	SPG20	Missense	p.G531W	9	113	8.0%	.	.	.
GC13	13	36909756	C	A	SPG20	Missense	p.W71L	7	88	8.0%	.	.	.
GC13	13	39265113	C	A	FREM2	Missense	p.P1211H	8	96	8.3%	.	.	.
GC13	13	39430365	C	A	FREM2	Missense	p.P2343H	7	99	7.1%	.	.	.
GC13	13	39587222	C	A	PROSER1	Missense	p.G723W	8	117	6.8%	.	.	.
GC13	13	39587375	C	A	PROSER1	Missense	p.G672C	7	92	7.6%	.	.	.
GC13	13	39587513	C	A	PROSER1	Missense	p.G626W	9	82	11.0%	.	.	.
GC13	13	39613355	C	A	NHLRC3	Missense	p.P56Q	6	68	8.8%	.	.	.
GC13	13	40301652	C	A	COG6	Missense	p.P598Q	10	113	8.8%	.	.	.
GC13	13	41508037	C	A	ELF1	Nonsense	p.G438X	7	100	7.0%	.	.	.
GC13	13	41639390	C	A	WBP4	Missense	p.Q77K	7	65	10.8%	.	.	.
GC13	13	41704903	C	A	KBTBD6	Missense	p.R582L	6	91	6.6%	.	.	.
GC13	13	41706637	C	A	KBTBD6	Missense	p.R4L	7	89	7.9%	.	.	.
GC13	13	41767765	C	A	KBTBD7	Missense	p.R210L	9	114	7.9%	.	O	.
GC13	13	41768383	C	A	KBTBD7	Missense	p.R4L	6	70	8.6%	.	.	.

GC13	13	41949598	C	A	NAA16	Missense	p.Q819K	7	84	8.3%	.	.	.
GC13	13	42784763	G	T	DGKH	Missense	p.W714L	9	128	7.0%	.	.	.
GC13	13	43358206	G	T	FAM216B	Missense	p.M1I	6	59	10.2%	.	.	.
GC13	13	43360966	G	T	FAM216B	Missense	p.W56L	7	64	10.9%	.	O	.
GC13	13	43918740	G	T	ENOX1	Missense	p.H359N	8	117	6.8%	.	.	.
GC13	13	44456372	C	A	LACC1	Missense	p.P205Q	7	87	8.0%	.	.	.
GC13	13	44464265	G	T	LACC1	Missense	p.Q383H	8	101	7.9%	.	.	.
GC13	13	45147350	G	T	TSC22D1	Missense	p.P954Q	6	98	6.1%	.	O	.
GC13	13	45602014	C	A	GPALPP1	Missense	p.P300Q	8	83	9.6%	.	.	.
GC13	13	45694803	G	T	GTF2F2	Missense	p.G5W	7	71	9.9%	.	.	.
GC13	13	46099140	G	T	COG3	Missense	p.Q728H	9	89	10.1%	.	.	.
GC13	13	46170759	G	T	ERICH6B	Missense	p.L128M	10	152	6.6%	.	.	.
GC13	13	46287564	G	T	SPERT	Missense	p.W108L	10	89	11.2%	.	.	.
GC13	13	46594628	G	T	ZC3H13	Missense	p.P135Q	7	90	7.8%	.	.	.
GC13	13	46918908	G	T	RUBCNL	Missense	p.H564N	7	73	9.6%	.	.	.
GC13	13	47262113	G	T	LRCH1	Missense	p.G317C	7	89	7.9%	.	.	.
GC13	13	47409210	C	A	HTR2A	Missense	p.R309L	7	124	5.6%	.	O	.
GC13	13	48985739	G	T	LPAR6	Missense	p.P274Q	11	122	9.0%	.	.	.
GC13	13	50057599	G	T	SETDB2	Splicing	c.1419-1G>T;NM_001160308	12	124	9.7%	.	.	.
GC13	13	50100537	G	T	PHF11	Missense	p.G262W	12	121	9.9%	.	.	.
GC13	13	52511697	G	T	ATP7B	Missense	p.P1066Q	6	87	6.9%	.	.	.
GC13	13	52639587	C	A	NEK5	Missense	p.G695W	8	64	12.5%	.	.	.
GC13	13	53233327	C	A	SUGT1	Missense	p.H91N	8	131	6.1%	.	.	.
GC13	13	53307425	C	A	CNMD	Missense	p.G95W	9	108	8.3%	.	.	.
GC13	13	61057958	C	A	TDRD3	Missense	p.P182Q	6	83	7.2%	.	.	.
GC13	13	61084836	C	A	TDRD3	Missense	p.P363Q	8	128	6.3%	.	.	.
GC13	13	61988041	C	A	PCDH20	Missense	p.R64L	7	78	9.0%	.	.	.
GC13	13	67799666	C	A	PCDH9	Missense	p.L969F	7	74	9.5%	.	.	.
GC13	13	67801206	C	A	PCDH9	Missense	p.R456M	7	87	8.0%	.	.	.
GC13	13	73319205	G	T	BORA	Missense	p.G130W	7	68	10.3%	.	.	.
GC13	13	73335813	G	T	DIS3	Missense	p.Q705K	10	123	8.1%	.	.	.

GC13	13	73357812	C	A	PIBF1	Missense	p.Q69K	8	80	10.0%	.	.	.
GC13	13	76381744	C	A	LMO7	Missense	p.P209Q	7	97	7.2%	.	.	.
GC13	13	76414552	C	A	LMO7	Missense	p.P935Q	5	49	10.2%	.	O	.
GC13	13	77629770	C	A	MYCBP2	Missense	p.G4524C	8	124	6.5%	.	.	.
GC13	13	77817260	G	T	MYCBP2	Missense	p.L855I	8	108	7.4%	.	.	.
GC13	13	78335202	G	T	SLAIN1	Missense	p.G153W	8	99	8.1%	.	.	.
GC13	13	78492614	G	T	EDNRB	Missense	p.P32Q	7	109	6.4%	.	.	.
GC13	13	79190793	C	A	RNF219	Missense	p.W368L	7	91	7.7%	.	.	.
GC13	13	79927327	C	A	RBM26	Missense	p.L675F	7	103	6.8%	.	.	.
GC13	13	80910987	C	A	SPRY2	Missense	p.R285L	7	112	6.3%	.	.	.
GC13	13	84455005	G	C	SLITRK1	Missense	p.P213R	5	80	6.3%	.	.	.
GC13	13	88328163	G	T	SLITRK5	Missense	p.G174W	7	99	7.1%	.	O	.
GC13	13	88328266	G	T	SLITRK5	Missense	p.R208L	7	109	6.4%	.	.	.
GC13	13	88330378	G	T	SLITRK5	Missense	p.R912L	7	80	8.8%	.	.	.
GC13	13	94680044	G	T	GPC6	Missense	p.R258L	8	85	9.4%	.	.	.
GC13	13	96212653	G	T	CLDN10	Nonsense	p.G113X	7	90	7.8%	.	.	.
GC13	13	97999092	G	T	MBNL2	Missense	p.R192L	7	77	9.1%	.	.	.
GC13	13	99091389	G	T	FARP1	Missense	p.R791L	6	76	7.9%	.	.	.
GC13	13	99134542	C	A	STK24	Missense	p.G103C	6	73	8.2%	.	.	.
GC13	13	99339905	G	T	SLC15A1	Missense	p.P586Q	7	88	8.0%	.	.	.
GC13	13	100172355	G	T	TM9SF2	Missense	p.G102V	8	97	8.2%	.	.	.
GC13	13	100635325	C	A	ZIC2	Missense	p.P336H	8	103	7.8%	.	.	.
GC13	13	101257378	C	A	TMTC4	Missense	p.W588L	7	102	6.9%	.	.	.
GC13	13	101315435	C	A	TMTC4	Splicing	c.336-1G>T;NM_001350572	7	83	8.4%	.	.	.
GC13	13	101759925	G	T	NALCN	Missense	p.P802Q	8	78	10.3%	.	.	.
GC13	13	102235608	C	A	ITGBL1	Missense	p.P116Q	8	91	8.8%	.	.	.
GC13	13	103053826	G	T	FGF14	Missense	p.P68Q	7	97	7.2%	.	.	.
GC13	13	103384964	C	A	CCDC168	Missense	p.R6028M	8	110	7.3%	.	.	.
GC13	13	103389986	G	T	CCDC168	Missense	p.P4354Q	8	143	5.6%	.	.	.
GC13	13	103392606	G	T	CCDC168	Missense	p.H3481N	8	120	6.7%	.	.	.
GC13	13	103393247	G	T	CCDC168	Missense	p.P3267Q	9	127	7.1%	.	.	.

GC13	13	103718319	G	T	SLC10A2	Missense	p.P94Q	5	44	11.4%	.	.	.
GC13	13	106118671	C	A	DAOA	Missense	p.Q12K	6	65	9.2%	.	.	.
GC13	13	107220173	C	A	ARGLU1	Missense	p.R32L	7	94	7.4%	.	.	.
GC13	13	108862433	G	T	LIG4	Missense	p.P395Q	7	98	7.1%	.	.	.
GC13	13	109777616	G	T	MYO16	Missense	p.R1231L	6	98	6.1%	.	O	.
GC13	13	111099144	G	T	COL4A2	Splicing	c.1012-1G>T	7	88	8.0%	.	.	.
GC13	13	111099211	G	T	COL4A2	Missense	p.G360C	9	108	8.3%	.	.	.
GC13	13	111372101	G	T	ING1	Missense	p.G204V	7	72	9.7%	.	.	.
GC13	13	113158347	C	A	TUBGCP3	Missense	p.R759M	6	63	9.5%	.	.	.
GC13	13	113768196	G	T	F7	Missense	p.G34W	6	41	14.6%	.	.	.
GC13	13	113803622	G	T	F10	Missense	p.G376W	5	72	6.9%	.	.	.
GC13	13	114294457	G	T	TFDP1	Missense	p.G370W	8	86	9.3%	.	.	.
GC13	13	114304735	C	A	ATP4B	Missense	p.G234W	7	65	10.8%	.	.	.
GC13	13	114541138	C	A	GAS6	Missense	p.G165W	4	33	12.1%	.	.	.
GC13	13	114622504	G	T	LINC00452	Missense	p.R137L	7	80	8.8%	.	.	.
GC13	13	115047178	G	T	UPF3A	Missense	p.G22W	5	41	12.2%	.	.	.
GC13	13	115090734	C	A	CHAMP1	Missense	p.R473S	8	97	8.2%	.	.	.
GC13	14	20528813	G	T	OR4L1	Missense	p.G204W	8	107	7.5%	.	.	.
GC13	14	20585944	G	T	OR4K17	Missense	p.G127W	9	98	9.2%	.	O	.
GC13	14	20586352	G	T	OR4K17	Missense	p.G263W	7	94	7.4%	.	.	.
GC13	14	20666332	G	T	OR11G2	Missense	p.G280W	7	67	10.4%	.	.	.
GC13	14	20837585	C	A	TEP1	Missense	p.R2417M	6	57	10.5%	.	.	.
GC13	14	20864884	C	A	TEP1	Missense	p.G411W	6	43	14.0%	.	.	.
GC13	14	21360009	G	T	RNASE3	Missense	p.R55L	10	96	10.4%	.	.	.
GC13	14	21502242	G	T	RNASE13	Missense	p.P69Q	8	106	7.5%	.	.	.
GC13	14	21502314	G	T	RNASE13	Missense	p.P45Q	10	120	8.3%	.	.	.
GC13	14	21698493	C	A	HNRNPC	Missense	p.R130L	7	70	10.0%	.	.	.
GC13	14	21820879	G	T	SUPT16H	Missense	p.R1033S	7	88	8.0%	.	.	.
GC13	14	21821705	C	A	SUPT16H	Missense	p.L980F	8	62	12.9%	.	.	.
GC13	14	21862264	C	A	CHD8	Missense	p.R1897L	6	74	8.1%	.	.	.
GC13	14	21867844	G	T	CHD8	Missense	p.P1613Q	8	90	8.9%	.	.	.

GC13	14	21876914	C	A	CHD8	Missense	p.R812L	8	112	7.1%	.	.	.
GC13	14	21896266	G	T	CHD8	Missense	p.Q455K	6	60	10.0%	.	.	.
GC13	14	21969062	G	T	METTL3	Missense	p.P370Q	5	31	16.1%	.	.	.
GC13	14	22038660	C	A	OR10G3	Missense	p.M72I	6	64	9.4%	.	.	.
GC13	14	22102735	C	A	OR10G2	Missense	p.L88F	9	71	12.7%	.	.	.
GC13	14	23392348	C	A	PRMT5	Missense	p.G295V	7	56	12.5%	.	.	.
GC13	14	23450584	G	T	AJUBA	Missense	p.R298S	7	82	8.5%	.	.	.
GC13	14	23458978	G	T	C14orf93	Missense	p.H353N	7	83	8.4%	.	.	.
GC13	14	23519108	G	T	CDH24	Missense	p.L470M	8	62	12.9%	.	.	.
GC13	14	23533352	G	T	ACIN1	Missense	p.H184N	9	97	9.3%	.	.	.
GC13	14	23652077	G	T	SLC7A8	Missense	p.P16Q	9	94	9.6%	.	.	.
GC13	14	23739169	G	T	RNF212B	Missense	p.A258S	9	92	9.8%	.	.	.
GC13	14	23746048	C	A	HOMEZ	Missense	p.R130L	7	83	8.4%	.	.	.
GC13	14	23777116	G	T	BCL2L2	Missense	p.R47L	5	49	10.2%	.	.	.
GC13	14	23868203	G	T	MYH6	Missense	p.P542H	7	43	16.3%	.	.	.
GC13	14	23993714	C	A	ZFHX2	Missense	p.G1813W	7	64	10.9%	.	.	.
GC13	14	24004584	C	A	ZFHX2	Splicing	.	5	30	16.7%	.	.	.
GC13	14	24026061	G	T	THTPA	Missense	p.R32L	5	53	9.4%	.	.	.
GC13	14	24035799	G	T	AP1G2	Missense	p.H101N	7	98	7.1%	.	.	.
GC13	14	24543305	G	T	CPNE6	Missense	p.G132W	13	86	15.1%	.	.	.
GC13	14	24569243	G	T	PCK2	Missense	p.G352V	7	95	7.4%	.	.	.
GC13	14	24588974	C	A	DCAF11	Missense	p.Q321K	6	54	11.1%	.	.	.
GC13	14	24606367	G	T	PSME1	Missense	p.G30W	8	106	7.5%	.	.	.
GC13	14	24619889	G	T	RNF31	Missense	p.W276L	7	98	7.1%	.	.	.
GC13	14	24648638	C	A	REC8	Missense	p.R429S	5	65	7.7%	.	.	.
GC13	14	24677240	G	T	TSSK4	Missense	p.W302L	7	67	10.4%	.	.	.
GC13	14	24737165	C	A	RABGGTA	Splicing	c.1148-1G>T	6	56	10.7%	.	.	.
GC13	14	24769360	G	T	NOP9	Missense	p.R67L	6	90	6.7%	.	.	.
GC13	14	24780917	G	T	LTB4R2	Missense	p.M349I	6	50	12.0%	.	.	.
GC13	14	24798420	C	A	ADCY4	Missense	p.K457N	7	90	7.8%	.	.	.
GC13	14	24839226	C	A	NFATC4	Missense	p.R208S	7	80	8.8%	.	.	.

GC13	14	24868520	G	T	NYNRIN	Missense	p.R23L	8	81	9.9%	.	.	.
GC13	14	25043975	C	A	CTSG	Missense	p.R82L	7	42	16.7%	.	O	.
GC13	14	26917476	G	T	NOVA1	Missense	p.L381I	7	79	8.9%	.	.	.
GC13	14	30108047	C	A	PRKD1	Nonsense	p.G254X	7	81	8.6%	.	O	.
GC13	14	31354665	C	A	COCH	Missense	p.P267T	7	90	7.8%	.	.	.
GC13	14	31425403	G	T	STRN3	Missense	p.L110M	9	144	6.3%	.	.	.
GC13	14	31638629	C	A	HECTD1	Missense	p.G460W	8	108	7.4%	.	.	.
GC13	14	32031286	G	T	NUBPL	Missense	p.G41W	5	65	7.7%	.	.	.
GC13	14	32047231	G	T	NUBPL	Missense	p.M1I	7	83	8.4%	.	.	.
GC13	14	32563102	G	T	ARHGAP5	Missense	p.R1076L	8	94	8.5%	.	.	.
GC13	14	32615492	C	A	ARHGAP5	Missense	p.R1297S	6	74	8.1%	.	.	.
GC13	14	33290824	G	T	AKAP6	Missense	p.G1269W	6	65	9.2%	.	.	.
GC13	14	35546384	G	T	FAM177A1	Missense	p.W123L	8	130	6.2%	.	.	.
GC13	14	35596794	C	A	KIAA0391	Missense	p.Q10K	6	58	10.3%	.	.	.
GC13	14	35739650	G	T	KIAA0391	Missense	p.G118W	7	35	20.0%	.	.	.
GC13	14	36004446	G	T	INSM2	Missense	p.G330W	6	88	6.8%	.	.	.
GC13	14	36004480	G	T	INSM2	Missense	p.R341L	7	87	8.0%	.	.	.
GC13	14	36041913	C	A	RALGAPA1	Missense	p.L2360F	8	106	7.5%	.	O	.
GC13	14	37149867	C	A	SLC25A21	Missense	p.X299L	9	114	7.9%	.	.	.
GC13	14	37736151	C	A	MIPOL1	Missense	p.H10N	8	100	8.0%	.	.	.
GC13	14	38286819	G	T	TTC6	Missense	p.G1592W	6	74	8.1%	.	.	.
GC13	14	38293058	G	T	TTC6	Missense	p.G1640C	7	93	7.5%	.	.	.
GC13	14	38724311	G	T	CLEC14A	Missense	p.P306Q	6	87	6.9%	.	.	.
GC13	14	38724780	C	A	CLEC14A	Missense	p.G150C	7	71	9.9%	.	.	.
GC13	14	39650160	C	A	PNN	Missense	p.P416H	7	93	7.5%	.	.	.
GC13	14	39784921	G	T	MIA2	Missense	p.W435L	7	78	9.0%	.	.	.
GC13	14	39870664	C	A	FBXO33	Missense	p.R371L	8	112	7.1%	.	.	.
GC13	14	42356540	G	T	LRFN5	Missense	p.G238C	8	107	7.5%	.	.	.
GC13	14	44974496	C	A	FSCB	Missense	p.K565N	8	95	8.4%	.	.	.
GC13	14	45433603	C	A	TOGARAM 1	Missense	p.P660Q	8	106	7.5%	.	.	.
GC13	14	45473338	G	T	TOGARAM 1	Missense	p.G805C	7	90	7.8%	.	.	.

GC13	14	45581520	G	T	PRPF39	Splicing	c.1573-1G>T	6	59	10.2%	.	.	.
GC13	14	50246956	G	T	KLHDC2	Missense	p.G267C	9	132	6.8%	.	.	.
GC13	14	50360482	G	T	ARF6	Missense	p.G10W	8	99	8.1%	.	.	.
GC13	14	50360783	G	T	ARF6	Missense	p.R110L	7	87	8.0%	.	.	.
GC13	14	50788230	C	A	ATP5S	Missense	p.Q24K	7	88	8.0%	.	.	.
GC13	14	50889888	G	T	MAP4K5	Missense	p.Q802K	7	99	7.1%	.	.	.
GC13	14	50929421	G	T	MAP4K5	Missense	p.H300N	10	146	6.8%	.	.	.
GC13	14	51102082	C	A	SAV1	Missense	p.W324L	6	72	8.3%	.	.	.
GC13	14	51202317	G	T	NIN	Missense	p.L1219I	7	77	9.1%	.	.	.
GC13	14	51224023	G	T	NIN	Missense	p.P1242H	8	134	6.0%	.	.	.
GC13	14	51347190	G	T	ABHD12B	Missense	p.R42L	5	49	10.2%	.	.	.
GC13	14	51378513	G	T	PYGL	Missense	p.P601H	7	80	8.8%	.	.	.
GC13	14	51382184	G	T	PYGL	Missense	p.R391S	7	119	5.9%	.	.	.
GC13	14	51561414	C	A	TRIM9	Missense	p.G82W	5	42	11.9%	.	.	.
GC13	14	51561492	C	A	TRIM9	Missense	p.G56W	6	69	8.7%	.	.	.
GC13	14	53245108	G	T	GNPNAT1	Missense	p.P159Q	7	85	8.2%	.	O	.
GC13	14	53518562	C	A	DDHD1	Missense	p.V841L	7	94	7.4%	.	O	.
GC13	14	54416962	C	A	BMP4	Missense	p.G339W	5	44	11.4%	.	.	.
GC13	14	54882633	G	T	CDKN3	Missense	p.G105W	7	75	9.3%	.	.	.
GC13	14	55251063	C	A	SAMD4A	Missense	p.Q275K	9	91	9.9%	.	.	.
GC13	14	55310766	C	A	GCH1	Missense	p.R241L	9	116	7.8%	.	.	.
GC13	14	55817419	C	A	FBXO34	Missense	p.P104Q	7	98	7.1%	.	.	.
GC13	14	55817871	C	A	FBXO34	Missense	p.P255T	7	87	8.0%	.	.	.
GC13	14	55818375	C	A	FBXO34	Missense	p.Q423K	8	105	7.6%	.	.	.
GC13	14	55848689	G	T	ATG14	Missense	p.Q290K	9	94	9.6%	.	.	.
GC13	14	56763838	G	T	PELI2	Missense	p.G406V	9	98	9.2%	.	O	.
GC13	14	57713539	G	T	EXOC5	Missense	p.Q54K	5	41	12.2%	.	.	.
GC13	14	57755523	G	T	AP5M1	Missense	p.R465L	6	57	10.5%	.	.	.
GC13	14	58605136	C	A	C14orf37	Missense	p.W314L	7	86	8.1%	.	.	.
GC13	14	59965585	C	A	JKAMP	Missense	p.P194Q	7	97	7.2%	.	.	.
GC13	14	60574835	C	A	PCNX4	Missense	p.P160Q	8	107	7.5%	.	.	.

GC13	14	60591351	G	T	PCNX4	Missense	p.W587L	7	87	8.0%	.	.	.
GC13	14	61746977	C	A	TMEM30B	Missense	p.G297C	5	35	14.3%	.	.	.
GC13	14	61789181	G	T	PRKCH	Missense	p.W121L	7	52	13.5%	.	.	.
GC13	14	62014505	G	T	PRKCH	Missense	p.Q602H	8	111	7.2%	.	.	.
GC13	14	62188319	G	T	HIF1A	Missense	p.G131C	8	123	6.5%	O	.	.
GC13	14	62233715	G	T	SNAPC1	Nonsense	p.G84X	8	119	6.7%	.	.	.
GC13	14	62547940	C	A	SYT16	Missense	p.P461Q	10	72	13.9%	.	.	.
GC13	14	63246537	C	A	KCNH5	Missense	p.R643L	6	82	7.3%	.	.	.
GC13	14	64152956	G	T	SGPP1	Missense	p.P398Q	7	86	8.1%	.	.	.
GC13	14	64165372	G	T	SGPP1	Missense	p.P230H	7	80	8.8%	.	.	.
GC13	14	64457792	G	T	SYNE2	Missense	p.G869W	6	57	10.5%	.	.	.
GC13	14	64469855	C	A	SYNE2	Missense	p.L1402M	9	127	7.1%	.	O	.
GC13	14	64493412	G	T	SYNE2	Missense	p.W2123L	7	82	8.5%	.	.	.
GC13	14	64519884	C	A	SYNE2	Missense	p.Q3085K	8	118	6.8%	.	.	.
GC13	14	64608116	G	T	SYNE2	Missense	p.G5012W	10	128	7.8%	.	.	.
GC13	14	64898562	C	A	MTHFD1	Missense	p.Q493K	7	97	7.2%	.	.	.
GC13	14	65009200	C	A	HSPA2	Missense	p.L545M	7	88	8.0%	.	.	.
GC13	14	65208451	G	T	PLEKHG3	Missense	p.R739L	6	84	7.1%	.	.	.
GC13	14	65210173	C	A	PLEKHG3	Missense	p.L1138M	7	73	9.6%	.	.	.
GC13	14	67346713	G	T	GPHN	Missense	p.M117I	7	97	7.2%	.	.	.
GC13	14	67576891	G	T	GPHN	Missense	p.R410L	8	98	8.2%	.	.	.
GC13	14	67819727	C	A	ATP6V1D	Missense	p.K24N	9	119	7.6%	.	.	.
GC13	14	68053885	G	T	PLEKHH1	Missense	p.W1343L	7	97	7.2%	.	.	.
GC13	14	68053896	G	T	PLEKHH1	Nonsense	p.G1347X	8	100	8.0%	.	.	.
GC13	14	68112478	G	T	ARG2	Nonsense	p.G161X	7	83	8.4%	.	.	.
GC13	14	68252944	C	A	ZFYVE26	Missense	p.R1009L	10	79	12.7%	.	.	.
GC13	14	69908965	C	A	SLC39A9	Missense	p.H129N	5	36	13.9%	.	.	.
GC13	14	69925177	C	A	SLC39A9	Missense	p.P241H	8	94	8.5%	.	.	.
GC13	14	70990862	G	T	ADAM20	Missense	p.H255N	8	106	7.5%	.	.	.
GC13	14	71443755	G	T	PCNX1	Missense	p.R234M	9	147	6.1%	.	.	.
GC13	14	71444723	C	A	PCNX1	Missense	p.H557N	9	130	6.9%	.	.	.

GC13	14	72055586	C	A	SIPA1L1	Missense	p.H333N	8	110	7.3%	.	.	.
GC13	14	72941391	C	A	RGS6	Missense	p.P191Q	8	109	7.3%	.	.	.
GC13	14	73406952	C	A	DCAF4	Missense	p.P73H	8	86	9.3%	.	.	.
GC13	14	73422363	C	A	DCAF4	Missense	p.Q320K	7	96	7.3%	.	.	.
GC13	14	73566458	G	T	RBM25	Missense	p.K289N	10	140	7.1%	.	.	.
GC13	14	73822341	G	T	NUMB	Missense	p.P40Q	11	87	12.6%	.	.	.
GC13	14	74427915	G	T	COQ6	Missense	p.G311C	7	73	9.6%	.	.	.
GC13	14	74495932	C	A	BBOF1	Missense	p.Q111K	7	85	8.2%	.	.	.
GC13	14	75265095	G	T	YLPM1	Missense	p.R1032L	7	122	5.7%	.	.	.
GC13	14	75330302	G	T	PROX2	Missense	p.P79Q	6	72	8.3%	.	O	.
GC13	14	75475823	G	T	EIF2B2	Missense	p.G330W	9	69	13.0%	.	.	.
GC13	14	75500191	C	A	MLH3	Missense	p.G1216W	7	58	12.1%	.	.	.
GC13	14	75516181	C	A	MLH3	Missense	p.G60W	8	117	6.8%	.	.	.
GC13	14	75520357	C	A	ACYP1	Missense	p.E30D	7	90	7.8%	.	.	.
GC13	14	75537623	G	T	ZC2HC1C	Missense	p.W116L	9	99	9.1%	.	.	.
GC13	14	75551334	G	T	NEK9	Missense	p.P970Q	8	74	10.8%	.	.	.
GC13	14	75553774	G	T	NEK9	Missense	p.Q934K	8	110	7.3%	.	.	.
GC13	14	75745755	G	T	FOS	Missense	p.G24W	7	112	6.3%	.	.	.
GC13	14	76100038	C	A	FLVCR2	Missense	p.P135Q	6	51	11.8%	.	.	.
GC13	14	76201616	C	A	TTLL5	Missense	p.P422H	7	83	8.4%	.	.	.
GC13	14	76259340	G	T	TTLL5	Missense	p.R1023L	7	101	6.9%	.	.	.
GC13	14	77275789	G	T	ANGEL1	Missense	p.Q88K	6	53	11.3%	.	.	.
GC13	14	77492107	C	A	IRF2BPL	Missense	p.G677W	6	47	12.8%	.	.	.
GC13	14	77753118	C	A	POMT2	Missense	p.R434L	5	49	10.2%	.	.	.
GC13	14	77755153	G	T	POMT2	Missense	p.P402Q	7	85	8.2%	.	.	.
GC13	14	77845352	C	A	SAMD15	Missense	p.Q531K	7	97	7.2%	.	.	.
GC13	14	77872338	C	A	NOXRED1	Missense	p.G275W	7	97	7.2%	.	O	.
GC13	14	77872392	G	T	NOXRED1	Missense	p.Q257K	8	71	11.3%	.	.	.
GC13	14	78197445	G	T	SNW1	Missense	p.Q307K	9	107	8.4%	.	.	.
GC13	14	78365572	C	A	ADCK1	Missense	p.Q170K	6	57	10.5%	.	.	.
GC13	14	79746866	C	A	NRXN3	Missense	p.L78I	7	91	7.7%	.	.	.

GC13	14	80164166	G	T	NRXN3	Missense	p.L265F	11	73	15.1%	.	.	.
GC13	14	81965814	G	T	SEL1L	Missense	p.H294N	7	92	7.6%	.	.	.
GC13	14	86087892	G	T	FLRT2	Missense	p.G12W	7	100	7.0%	.	.	.
GC13	14	86088495	G	T	FLRT2	Missense	p.G213W	8	103	7.8%	.	.	.
GC13	14	86089090	G	T	FLRT2	Missense	p.W411L	7	81	8.6%	.	.	.
GC13	14	86089401	C	A	FLRT2	Missense	p.H515N	8	88	9.1%	.	.	.
GC13	14	88892838	G	T	SPATA7	Missense	p.R180L	6	85	7.1%	.	.	.
GC13	14	88892870	G	T	SPATA7	Missense	p.G191W	11	99	11.1%	.	.	.
GC13	14	88970813	C	A	PTPN21	Missense	p.L181F	7	93	7.5%	.	.	.
GC13	14	89069345	C	A	ZC3H14	Missense	p.P383Q	7	85	8.2%	.	.	.
GC13	14	89084996	G	T	EML5	Missense	p.Q1846K	5	39	12.8%	.	.	.
GC13	14	89110777	C	A	EML5	Missense	p.E1369D	7	88	8.0%	.	.	.
GC13	14	89116267	C	A	EML5	Missense	p.Q1336H	8	91	8.8%	.	.	.
GC13	14	89153631	C	A	EML5	Missense	p.W928L	7	89	7.9%	.	.	.
GC13	14	90420699	C	A	EFCAB11	Missense	p.M1I	6	42	14.3%	.	.	.
GC13	14	90782925	G	T	NRDE2	Missense	p.P135Q	6	87	6.9%	.	O	.
GC13	14	91386566	G	T	RPS6KA5	Missense	p.Q45K	13	120	10.8%	.	.	.
GC13	14	91925134	G	T	PPP4R3A	Missense	p.P585Q	7	87	8.0%	.	.	.
GC13	14	92074722	C	A	CATSPERB	Missense	p.M875I	7	83	8.4%	.	.	.
GC13	14	92258727	G	T	TC2N	Missense	p.P344Q	7	81	8.6%	.	.	.
GC13	14	92588066	G	T	NDUFB1	Missense	p.P19Q	7	69	10.1%	.	O	.
GC13	14	92604619	C	A	CPSF2	Missense	p.L44I	8	116	6.9%	.	.	.
GC13	14	93760677	G	T	BTBD7	Missense	p.P230Q	6	75	8.0%	.	.	.
GC13	14	94044401	C	A	UNC79	Missense	p.L809M	8	98	8.2%	.	.	.
GC13	14	94413728	G	T	ASB2	Missense	p.P292Q	5	54	9.3%	.	.	.
GC13	14	94528937	G	T	DDX24	Missense	p.P250Q	6	63	9.5%	.	.	.
GC13	14	95056384	G	T	SERPINA5	Missense	p.W209L	9	80	11.3%	.	.	.
GC13	14	95085601	G	T	SERPINA3	Missense	p.W238L	7	94	7.4%	.	.	.
GC13	14	95569883	C	A	DICER1	Missense	p.G1284W	7	88	8.0%	O	.	.
GC13	14	95670356	G	T	CLMN	Missense	p.L444I	8	123	6.5%	.	.	.
GC13	14	96706783	G	T	BDKRB2	Missense	p.G40W	7	95	7.4%	.	.	.

GC13	14	96792204	C	A	ATG2B	Missense	p.R740L	6	93	6.5%	.	.	.
GC13	14	96937908	C	A	AK7	Missense	p.P458Q	11	152	7.2%	.	.	.
GC13	14	97002317	G	T	PAPOLA	Missense	p.M87I	7	91	7.7%	.	.	.
GC13	14	97319196	G	T	VRK1	Missense	p.G135W	9	99	9.1%	.	.	.
GC13	14	99879344	G	T	SETD3	Missense	p.R265S	6	44	13.6%	.	.	.
GC13	14	100119141	G	T	HHIPL1	Missense	p.W279L	6	51	11.8%	.	.	.
GC13	14	100158157	C	A	CYP46A1	Missense	p.H81N	7	95	7.4%	.	O	.
GC13	14	101349484	C	A	RTL1	Missense	p.G548C	7	69	10.1%	.	.	.
GC13	14	102378761	G	T	PPP2R5C	Missense	p.R426L	6	80	7.5%	.	.	.
GC13	14	102442120	G	T	DYNC1H1	Missense	p.G110W	7	95	7.4%	.	.	.
GC13	14	102471252	C	A	DYNC1H1	Missense	p.P1735T	8	109	7.3%	.	.	.
GC13	14	102675196	C	A	WDR20	Missense	p.P169Q	7	92	7.6%	.	.	.
GC13	14	102675649	G	T	WDR20	Missense	p.W320L	7	80	8.8%	.	.	.
GC13	14	103173764	C	A	RCOR1	Missense	p.P192Q	9	103	8.7%	.	.	.
GC13	14	103396037	G	T	AMN	Missense	p.R269L	5	37	13.5%	.	.	.
GC13	14	103566857	C	A	EXOC3L4	Missense	p.Q101K	7	89	7.9%	.	.	.
GC13	14	104027192	G	T	BAG5	Missense	p.L104I	8	101	7.9%	.	.	.
GC13	14	104473537	C	A	TDRD9	Missense	p.P683Q	7	112	6.3%	.	.	.
GC13	14	104565275	C	A	ASPG	Missense	p.P200Q	4	39	10.3%	.	.	.
GC13	14	105268021	G	T	ZBTB42	Missense	p.G163W	7	67	10.4%	.	.	.
GC13	14	105353515	G	T	CEP170B	Missense	p.R910L	6	76	7.9%	.	.	.
GC13	14	105353560	C	A	CEP170B	Missense	p.P925Q	6	79	7.6%	.	.	.
GC13	14	105359375	G	T	CEP170B	Missense	p.G1279V	6	53	11.3%	.	.	.
GC13	14	105407345	C	A	AHNAK2	Missense	p.G4715W	7	71	9.9%	.	.	.
GC13	14	105518126	C	A	GPR132	Missense	p.K107N	8	86	9.3%	.	.	.
GC13	15	22990094	A	C	CYFIP1	Missense	p.Y474S	22	68	32.4%	.	.	.
GC13	15	23811004	G	T	MKRN3	Missense	p.E25D	6	52	11.5%	.	.	.
GC13	15	23811411	C	A	MKRN3	Missense	p.P161Q	7	76	9.2%	.	.	.
GC13	15	24921550	C	A	NPAP1	Missense	p.P179H	7	91	7.7%	.	O	.
GC13	15	24923205	G	T	NPAP1	Missense	p.G731W	8	102	7.8%	.	.	.
GC13	15	24924320	G	T	NPAP1	Missense	p.M1102I	7	84	8.3%	.	.	.

GC13	15	25947080	G	T	ATP10A	Missense	p.L915M	6	61	9.8%	.	.	.
GC13	15	25947166	C	A	ATP10A	Missense	p.W886L	8	97	8.2%	.	.	.
GC13	15	29390711	C	A	APBA2	Missense	p.Q412K	6	73	8.2%	.	.	.
GC13	15	30919102	C	A	ARHGAP11<br>B	Missense	p.R27S	5	54	9.3%	.	.	.
GC13	15	31222857	C	A	FAN1	Missense	p.Q967K	7	67	10.4%	.	.	.
GC13	15	31294249	C	A	TRPM1	Missense	p.G1569W	8	100	8.0%	.	.	.
GC13	15	32404058	C	A	CHRNA7	Missense	p.P103Q	7	89	7.9%	.	.	.
GC13	15	33835909	C	A	RYR3	Missense	p.Q245K	8	105	7.6%	.	.	.
GC13	15	33936680	C	A	RYR3	Missense	p.P1242Q	6	85	7.1%	.	.	.
GC13	15	34023715	G	T	RYR3	Missense	p.R2415M	6	62	9.7%	.	O	.
GC13	15	34119428	G	T	RYR3	Missense	p.M3753I	9	90	10.0%	.	.	.
GC13	15	34129979	C	A	RYR3	Missense	p.P3928Q	8	99	8.1%	.	.	.
GC13	15	34130286	G	T	RYR3	Missense	p.M4030I	8	103	7.8%	.	.	.
GC13	15	34130359	C	A	RYR3	Missense	p.Q4055K	6	80	7.5%	.	.	.
GC13	15	34131038	C	A	RYR3	Missense	p.P4281Q	9	68	13.2%	.	.	.
GC13	15	34157422	G	T	RYR3	Nonsense	p.G4865X	8	102	7.8%	.	.	.
GC13	15	34355001	G	T	CHRM5	Missense	p.W28L	7	100	7.0%	.	.	.
GC13	15	34355213	G	T	CHRM5	Missense	p.G99W	11	131	8.4%	.	.	.
GC13	15	34649195	C	A	NUTM1	Missense	p.Q986K	8	100	8.0%	.	.	.
GC13	15	35149085	C	A	AQR	Nonsense	p.E1456X	19	54	35.2%	.	O	.
GC13	15	35149272	C	A	AQR	Missense	p.E1393D	6	59	10.2%	.	.	.
GC13	15	35212617	C	A	AQR	Missense	p.Q379H	8	96	8.3%	.	.	.
GC13	15	38773632	G	T	FAM98B	Missense	p.R290L	7	67	10.4%	.	.	.
GC13	15	38786834	G	T	RASGRP1	Missense	p.H635N	5	43	11.6%	.	.	.
GC13	15	39882774	G	T	THBS1	Missense	p.G735C	7	101	6.9%	.	.	.
GC13	15	40031892	G	T	FSIP1	Missense	p.Q252K	11	149	7.4%	.	.	.
GC13	15	40094102	C	A	GPR176	Missense	p.R215L	5	68	7.4%	.	.	.
GC13	15	40477579	G	T	BUB1B	Missense	p.R322M	9	109	8.3%	O	.	.
GC13	15	40504844	C	A	BUB1B	Missense	p.L844I	6	66	9.1%	O	.	.
GC13	15	40565630	G	T	PAK6	Missense	p.R525L	5	57	8.8%	.	.	.
GC13	15	40764506	C	A	CHST14	Missense	p.P365Q	6	53	11.3%	.	.	.

GC13	15	40862006	G	T	RPUSD2	Missense	p.W157L	7	88	8.0%	.	.	.
GC13	15	40914809	G	T	KNL1	Missense	p.G783C	12	128	9.4%	.	.	.
GC13	15	40915730	C	A	KNL1	Missense	p.Q1090K	7	82	8.5%	.	.	.
GC13	15	40951649	G	T	KNL1	Missense	p.G2276W	8	114	7.0%	.	.	.
GC13	15	41001282	C	A	RAD51	Missense	p.Q136K	7	97	7.2%	.	.	.
GC13	15	41029502	C	A	RMDN3	Missense	p.R421M	7	94	7.4%	.	.	.
GC13	15	41099516	G	T	ZFYVE19	Missense	p.Q10H	6	51	11.8%	.	.	.
GC13	15	41102065	G	T	ZFYVE19	Missense	p.L156F	8	109	7.3%	.	.	.
GC13	15	41192067	G	T	VPS18	Missense	p.G351W	7	83	8.4%	.	.	.
GC13	15	41226836	C	A	DLL4	Missense	p.P314Q	7	46	15.2%	.	.	.
GC13	15	41247809	G	T	CHAC1	Missense	p.R211L	6	87	6.9%	.	.	.
GC13	15	41337102	C	A	INO80	Missense	p.K969N	8	92	8.7%	.	.	.
GC13	15	41340434	G	T	INO80	Missense	p.Q873K	8	113	7.1%	.	.	.
GC13	15	41476551	C	A	EXD1	Missense	p.G375W	7	101	6.9%	.	.	.
GC13	15	41562799	C	A	CHP1	Missense	p.R132S	6	68	8.8%	.	.	.
GC13	15	41796336	G	T	LTK	Missense	p.P688Q	6	55	10.9%	.	.	.
GC13	15	41813936	G	T	RPAP1	Missense	p.P1013Q	6	67	9.0%	.	.	.
GC13	15	42041587	G	T	MGA	Nonsense	p.G1719X	8	89	9.0%	.	.	.
GC13	15	42067505	G	T	MAPKBP1	Missense	p.R11L	6	79	7.6%	.	.	.
GC13	15	42104751	G	T	MAPKBP1	Missense	p.R179L	7	106	6.6%	.	.	.
GC13	15	42457995	G	T	VPS39	Missense	p.P567Q	6	64	9.4%	.	.	.
GC13	15	42458373	G	T	VPS39	Missense	p.P555Q	8	86	9.3%	.	.	.
GC13	15	42742304	C	A	ZNF106	Missense	p.L699F	7	98	7.1%	.	.	.
GC13	15	42839602	C	A	LRRC57	Missense	p.G117W	7	63	11.1%	.	.	.
GC13	15	42967078	G	T	STARD9	Missense	p.G535C	6	61	9.8%	.	.	.
GC13	15	42977419	G	T	STARD9	Missense	p.G1215W	7	99	7.1%	.	.	.
GC13	15	42980002	C	A	STARD9	Missense	p.H2076N	8	112	7.1%	.	.	.
GC13	15	42981606	G	T	STARD9	Missense	p.E2610D	7	83	8.4%	.	.	.
GC13	15	42981778	C	A	STARD9	Missense	p.H2668N	8	98	8.2%	.	.	.
GC13	15	42982086	G	T	STARD9	Missense	p.E2770D	7	93	7.5%	.	.	.
GC13	15	42982191	G	T	STARD9	Missense	p.Q2805H	8	78	10.3%	.	.	.

GC13	15	42984440	G	T	STARD9	Missense	p.W3555L	9	96	9.4%	.	.	.
GC13	15	42984484	C	A	STARD9	Missense	p.H3570N	7	88	8.0%	.	.	.
GC13	15	42984862	G	T	STARD9	Missense	p.G3696W	10	112	8.9%	.	.	.
GC13	15	43022829	C	A	CDAN1	Missense	p.R714L	5	43	11.6%	.	.	.
GC13	15	43262718	C	A	UBR1	Missense	p.G1486V	8	106	7.5%	.	.	.
GC13	15	43461795	G	T	TMEM62	Missense	p.R229L	6	99	6.1%	.	.	.
GC13	15	43574117	C	A	TGM7	Missense	p.G426W	6	71	8.5%	.	.	.
GC13	15	43641162	G	T	ADAL	Missense	p.G231W	7	83	8.4%	.	.	.
GC13	15	43653816	G	T	ZSCAN29	Missense	p.H672N	7	71	9.9%	.	.	.
GC13	15	43699720	G	T	TP53BP1	Missense	p.P1930Q	7	87	8.0%	.	.	.
GC13	15	43767862	C	A	TP53BP1	Missense	p.R329M	6	60	10.0%	.	.	.
GC13	15	43815575	C	A	MAP1A	Missense	p.P635Q	8	101	7.9%	.	.	.
GC13	15	43815715	C	A	MAP1A	Missense	p.Q682K	7	68	10.3%	.	.	.
GC13	15	43817353	G	T	MAP1A	Missense	p.G1228W	7	83	8.4%	.	.	.
GC13	15	43819399	C	A	MAP1A	Missense	p.R1910S	6	64	9.4%	.	.	.
GC13	15	43893733	C	A	STRC	Missense	p.R1521L	6	98	6.1%	.	.	.
GC13	15	43927973	C	A	CATSPER2	Missense	p.R364L	5	65	7.7%	.	.	.
GC13	15	43990227	C	A	CKMT1A	Missense	p.Q298K	8	84	9.5%	.	.	.
GC13	15	44065346	G	T	ELL3	Missense	p.L388M	8	110	7.3%	.	.	.
GC13	15	44067732	C	A	ELL3	Missense	p.W187L	7	83	8.4%	.	.	.
GC13	15	44091188	G	T	SERINC4	Missense	p.P143T	7	87	8.0%	.	.	.
GC13	15	44105297	G	T	MFAP1	Missense	p.L259M	8	144	5.6%	.	.	.
GC13	15	44143427	G	T	WDR76	Missense	p.R328M	7	99	7.1%	.	.	.
GC13	15	44865774	C	A	SPG11	Missense	p.R2059L	9	87	10.3%	.	.	O
GC13	15	44876390	C	A	SPG11	Missense	p.G1830C	8	133	6.0%	.	.	.
GC13	15	44887583	C	A	SPG11	Missense	p.M1503I	7	86	8.1%	.	.	.
GC13	15	45360387	G	T	SORD	Missense	p.G185W	7	89	7.9%	.	.	.
GC13	15	48056102	G	T	SEMA6D	Missense	p.R268L	6	51	11.8%	.	.	.
GC13	15	48058844	G	T	SEMA6D	Missense	p.G573C	7	101	6.9%	.	O	.
GC13	15	48063451	G	T	SEMA6D	Missense	p.M835I	7	82	8.5%	.	.	.
GC13	15	48063534	G	T	SEMA6D	Missense	p.R863L	6	67	9.0%	.	.	.

GC13	15	48539588	G	T	SLC12A1	Missense	p.G539W	7	95	7.4%	.	.	.
GC13	15	48595027	C	A	SLC12A1	Missense	p.P1082Q	8	107	7.5%	.	.	.
GC13	15	48779335	C	A	FBN1	Missense	p.G1176W	8	93	8.6%	.	.	.
GC13	15	49048540	G	T	CEP152	Missense	p.H969N	7	96	7.3%	.	.	.
GC13	15	49217086	G	T	SHC4	Missense	p.Q216K	10	108	9.3%	.	.	.
GC13	15	49293206	G	T	SECISBP2L	Missense	p.Q661K	8	123	6.5%	.	.	.
GC13	15	49575874	C	A	GALK2	Missense	p.H228N	11	67	16.4%	.	.	.
GC13	15	50494723	G	T	SLC27A2	Missense	p.W243L	8	90	8.9%	.	.	.
GC13	15	50515174	C	A	SLC27A2	Missense	p.R276S	7	48	14.6%	.	.	.
GC13	15	50782605	G	T	USP8	Missense	p.R600L	7	102	6.9%	O	.	.
GC13	15	50867211	C	A	TRPM7	Missense	p.M1618I	11	151	7.3%	.	.	.
GC13	15	50884215	G	T	TRPM7	Missense	p.P1406Q	8	128	6.3%	.	.	.
GC13	15	50906395	C	A	TRPM7	Missense	p.M520I	7	81	8.6%	.	.	.
GC13	15	50920401	C	A	TRPM7	Missense	p.W417L	7	97	7.2%	.	.	.
GC13	15	50929721	G	T	TRPM7	Missense	p.H244N	9	138	6.5%	.	.	.
GC13	15	51000031	G	T	SPPL2A	Missense	p.P510H	8	112	7.1%	.	.	.
GC13	15	51293251	G	T	AP4E1	Missense	p.G967W	7	71	9.9%	.	.	.
GC13	15	51988185	C	A	SCG3	Missense	p.L96I	8	104	7.7%	.	.	.
GC13	15	52486235	C	A	MYO5C	Missense	p.R1698L	6	59	10.2%	.	.	.
GC13	15	52564020	G	T	MYO5C	Missense	p.H258N	9	96	9.4%	.	.	.
GC13	15	52605900	C	A	MYO5A	Missense	p.R1827L	8	108	7.4%	.	.	.
GC13	15	52671899	G	T	MYO5A	Missense	p.R711S	6	93	6.5%	.	.	.
GC13	15	52702625	C	A	MYO5A	Missense	p.G221W	9	92	9.8%	.	.	.
GC13	15	54003119	C	A	WDR72	Nonsense	p.G297X	9	82	11.0%	.	.	.
GC13	15	55839067	C	A	PYGO1	Missense	p.M138I	7	77	9.1%	.	.	.
GC13	15	55970236	C	A	PRTG	Missense	p.L380F	5	41	12.2%	.	.	.
GC13	15	57555435	C	A	TCF12	Missense	p.Q310K	10	123	8.1%	.	.	.
GC13	15	57820987	C	A	CGNL1	Missense	p.R1059S	4	33	12.1%	.	.	.
GC13	15	58834838	G	T	LIPC	Missense	p.G188W	7	80	8.8%	.	.	.
GC13	15	59146726	G	T	MINDY2	Missense	p.G595W	7	90	7.8%	.	.	.
GC13	15	59500942	C	A	MYO1E	Missense	p.G490W	8	113	7.1%	.	.	.

GC13	15	62161754	G	T	VPS13C	Missense	p.R3522S	7	87	8.0%	.	.	.
GC13	15	62209712	G	T	VPS13C	Missense	p.P2585Q	8	97	8.2%	.	.	.
GC13	15	63030462	C	A	TLN2	Missense	p.P1206H	7	84	8.3%	.	.	.
GC13	15	63053889	G	T	TLN2	Missense	p.G1550W	8	53	15.1%	.	.	.
GC13	15	63075992	C	A	TLN2	Missense	p.P1880Q	8	106	7.5%	.	.	.
GC13	15	63638872	G	T	CA12	Missense	p.P48Q	6	77	7.8%	.	.	.
GC13	15	63904617	G	T	HERC1	Missense	p.R4745S	7	83	8.4%	.	.	.
GC13	15	63915078	C	A	HERC1	Missense	p.G4542C	10	91	11.0%	.	.	.
GC13	15	63978692	G	T	HERC1	Missense	p.Q2031K	9	94	9.6%	.	.	.
GC13	15	64967186	G	T	ZNF609	Missense	p.M711I	11	112	9.8%	.	.	.
GC13	15	65214123	G	T	ANKDD1A	Missense	p.G91W	8	90	8.9%	.	.	.
GC13	15	65223757	G	T	ANKDD1A	Missense	p.G248W	9	90	10.0%	.	.	.
GC13	15	65257701	G	T	SPG21	Missense	p.P230Q	11	83	13.3%	.	.	.
GC13	15	65489713	G	T	CILP	Missense	p.R971S	7	79	8.9%	.	.	.
GC13	15	65555583	C	A	PARP16	Missense	p.G84W	7	69	10.1%	.	.	.
GC13	15	65624397	C	A	IGDCC3	Missense	p.G344W	7	61	11.5%	.	.	.
GC13	15	65756176	C	A	DPP8	Missense	p.G632W	6	72	8.3%	.	.	.
GC13	15	65855167	G	T	HACD3	Missense	p.L159F	10	104	9.6%	.	.	.
GC13	15	65871769	C	A	INTS14	Missense	p.G455W	7	75	9.3%	.	.	.
GC13	15	65982925	C	A	DENND4A	Missense	p.R1292M	8	72	11.1%	.	.	.
GC13	15	65994219	G	T	DENND4A	Missense	p.R814S	7	125	5.6%	.	O	.
GC13	15	65994758	C	A	DENND4A	Missense	p.W772L	7	94	7.4%	.	.	.
GC13	15	66604101	C	A	DIS3L	Missense	p.H74N	8	111	7.2%	.	.	.
GC13	15	66625210	G	T	DIS3L	Missense	p.G814W	9	114	7.9%	.	.	.
GC13	15	66737019	G	T	MAP2K1	Missense	p.R181M	7	66	10.6%	O	O	.
GC13	15	66853526	C	A	LCTL	Missense	p.R30L	8	72	11.1%	.	.	.
GC13	15	67430393	G	T	SMAD3	Missense	p.W10L	7	77	9.1%	O	.	.
GC13	15	67524226	G	T	AAGAB	Missense	p.P45Q	7	94	7.4%	.	.	.
GC13	15	68117307	C	A	SKOR1	Missense	p.P202Q	6	50	12.0%	.	.	.
GC13	15	68583146	G	T	FEM1B	Missense	p.G484C	9	129	7.0%	.	.	.
GC13	15	68643044	G	T	ITGA11	Missense	p.P324H	6	50	12.0%	.	.	.

GC13	15	68937547	G	T	CORO2B	Missense	p.G17W	6	57	10.5%	.	.	.
GC13	15	70980118	G	T	UACA	Missense	p.H143N	9	128	7.0%	.	.	.
GC13	15	71185243	G	T	LRRC49	Missense	p.G4W	7	89	7.9%	.	.	.
GC13	15	71188227	G	T	LRRC49	Missense	p.G54C	6	86	7.0%	.	.	.
GC13	15	72558332	C	A	PARP6	Missense	p.G28W	6	53	11.3%	.	.	.
GC13	15	72874482	C	A	ARIH1	Missense	p.Q515K	8	113	7.1%	.	.	.
GC13	15	73528724	G	T	NEO1	Missense	p.R443L	8	93	8.6%	.	.	.
GC13	15	73547143	G	T	NEO1	Missense	p.G689W	6	71	8.5%	.	.	.
GC13	15	73660007	G	T	HCN4	Missense	p.P202Q	5	38	13.2%	.	.	.
GC13	15	73994896	G	T	CD276	Missense	p.R127L	5	50	10.0%	.	.	.
GC13	15	74238796	G	T	LOXL1	Missense	p.R417L	4	35	11.4%	.	.	.
GC13	15	74427071	C	A	ISLR2	Missense	p.P659Q	6	87	6.9%	.	.	.
GC13	15	74481540	G	T	STRA6	Missense	p.L336M	6	38	15.8%	.	.	.
GC13	15	75012939	C	A	CYP1A1	Missense	p.R448L	6	89	6.7%	.	.	.
GC13	15	75042708	C	A	CYP1A2	Missense	p.P210H	7	78	9.0%	.	.	.
GC13	15	75042776	G	T	CYP1A2	Missense	p.G233W	10	94	10.6%	.	.	.
GC13	15	75499534	G	T	C15orf39	Missense	p.R382L	5	55	9.1%	.	.	.
GC13	15	75651720	G	T	MAN2C1	Missense	p.H670N	6	50	12.0%	.	.	.
GC13	15	75688768	G	T	SIN3A	Missense	p.R642S	6	75	8.0%	.	.	.
GC13	15	75968898	C	A	CSPG4	Missense	p.G1988W	8	50	16.0%	.	.	.
GC13	15	76496087	G	T	TMEM266	Missense	p.G343C	4	35	11.4%	.	.	.
GC13	15	76633523	C	A	ISL2	Missense	p.R282S	5	36	13.9%	.	.	.
GC13	15	77473962	C	A	PEAK1	Missense	p.G103W	8	127	6.3%	.	.	.
GC13	15	78337398	G	T	TBC1D2B	Missense	p.P176Q	6	51	11.8%	.	.	.
GC13	15	78456076	C	A	IDH3A	Missense	p.Q245K	8	93	8.6%	.	.	.
GC13	15	78755402	C	A	IREB2	Missense	p.P25H	8	143	5.6%	.	.	.
GC13	15	78783017	G	T	IREB2	Missense	p.L496F	9	126	7.1%	.	.	.
GC13	15	81046568	C	A	ABHD17C	Missense	p.H283N	8	104	7.7%	.	O	.
GC13	15	81271565	G	T	MESD	Missense	p.L234M	8	93	8.6%	.	.	.
GC13	15	81429001	C	A	CFAP161	Missense	p.H102N	6	65	9.2%	.	.	.
GC13	15	81625140	C	A	TMC3	Missense	p.G975W	6	54	11.1%	.	.	.

GC13	15	81628962	G	T	TMC3	Missense	p.Q731K	8	95	8.4%	.	.	.
GC13	15	83433991	C	A	FSD2	Nonsense	p.G538X	6	49	12.2%	.	.	.
GC13	15	83674445	C	A	C15orf40	Splicing	c.367-1G>T	8	101	7.9%	.	.	.
GC13	15	83687508	G	T	BTBD1	Missense	p.P385T	8	104	7.7%	.	.	.
GC13	15	84159649	C	A	SH3GL3	Missense	p.Q22K	7	64	10.9%	.	.	.
GC13	15	85164276	G	T	ZSCAN2	Missense	p.G284W	9	70	12.9%	.	.	.
GC13	15	85333944	G	T	ZNF592	Missense	p.Q743H	7	78	9.0%	.	.	.
GC13	15	85652349	C	A	PDE8A	Missense	p.R322S	5	55	9.1%	.	O	.
GC13	15	86123979	C	A	AKAP13	Missense	p.L894M	7	90	7.8%	.	.	.
GC13	15	86124636	C	A	AKAP13	Missense	p.Q1113K	8	114	7.0%	.	.	.
GC13	15	86198756	C	A	AKAP13	Missense	p.Q135K	10	122	8.2%	.	.	.
GC13	15	86253819	G	T	AKAP13	Missense	p.G469W	9	138	6.5%	.	.	.
GC13	15	86262336	C	A	AKAP13	Missense	p.R632S	6	79	7.6%	.	.	.
GC13	15	89074298	C	A	DET1	Missense	p.K213N	7	86	8.1%	.	.	.
GC13	15	89392852	C	A	ACAN	Missense	p.P639Q	7	65	10.8%	.	.	.
GC13	15	89728678	G	T	ABHD2	Missense	p.W249L	6	73	8.2%	.	.	.
GC13	15	89850731	G	T	FANCI	Nonsense	p.G1128X	6	69	8.7%	.	.	.
GC13	15	89870509	C	A	POLG	Missense	p.W441L	7	77	9.1%	.	.	.
GC13	15	90164715	G	T	TICRR	Missense	p.K1081N	7	78	9.0%	.	.	.
GC13	15	90167427	G	T	TICRR	Missense	p.G1295W	9	114	7.9%	.	.	.
GC13	15	90168564	G	T	TICRR	Missense	p.G1674W	8	44	18.2%	.	.	.
GC13	15	90191983	C	A	KIF7	Missense	p.G316W	5	39	12.8%	.	.	.
GC13	15	90336284	G	T	ANPEP	Missense	p.P744Q	7	68	10.3%	.	.	.
GC13	15	90342527	C	A	ANPEP	Missense	p.R658L	6	78	7.7%	.	.	.
GC13	15	90432317	C	A	AP3S2	Missense	p.R42L	9	97	9.3%	.	.	.
GC13	15	90631676	G	T	IDH2	Missense	p.P68Q	7	91	7.7%	O	.	.
GC13	15	91017836	C	A	IQGAP1	Missense	p.L899I	7	88	8.0%	.	.	.
GC13	15	91184404	G	T	CRTC3	Missense	p.G542W	7	99	7.1%	.	.	.
GC13	15	91292842	C	A	BLM	Missense	p.P115Q	7	123	5.7%	O	.	.
GC13	15	91304165	C	A	BLM	Missense	p.P521Q	8	116	6.9%	O	.	.
GC13	15	91454635	C	A	MAN2A2	Missense	p.P655Q	7	88	8.0%	.	.	.

GC13	15	91517927	C	A	PRC1	Missense	p.W372L	8	124	6.5%	.	.	.
GC13	15	91549930	G	T	VPS33B	Missense	p.Q217K	7	64	10.9%	.	.	.
GC13	15	93557996	G	T	CHD2	Missense	p.R1588L	5	50	10.0%	.	.	.
GC13	15	93567760	G	T	CHD2	Missense	p.G1771V	7	97	7.2%	.	.	.
GC13	15	93567865	G	T	CHD2	Missense	p.R1806M	8	138	5.8%	.	.	.
GC13	15	93567875	G	T	CHD2	Missense	p.L1809F	10	136	7.4%	.	.	.
GC13	15	99491823	G	T	IGF1R	Missense	p.W1203L	5	37	13.5%	.	O	.
GC13	15	100871202	G	T	ADAMTS17	Missense	p.Q170K	6	43	14.0%	.	.	.
GC13	15	101152540	C	A	ASB7	Missense	p.P40Q	8	121	6.6%	.	.	.
GC13	15	101718672	C	A	CHSY1	Missense	p.G444W	6	66	9.1%	.	.	.
GC13	15	102346042	G	T	OR4F6	Missense	p.M40I	8	90	8.9%	.	.	.
GC13	15	102346507	G	T	OR4F6	Missense	p.L195F	8	101	7.9%	.	.	.
GC13	16	256056	G	T	LUC7L	Missense	p.Q153K	11	135	8.1%	.	.	.
GC13	16	323476	G	T	RGS11	Missense	p.P185Q	5	43	11.6%	.	.	.
GC13	16	335638	G	T	PDIA2	Missense	p.G352W	7	95	7.4%	.	.	.
GC13	16	396787	G	T	AXIN1	Missense	p.P80Q	7	77	9.1%	O	.	.
GC13	16	681389	C	A	WFIKKN1	Missense	p.Q46K	6	33	18.2%	.	.	.
GC13	16	683378	G	T	WFIKKN1	Missense	p.R323L	7	66	10.6%	.	.	.
GC13	16	715978	C	A	WDR90	Missense	p.P1488Q	8	50	16.0%	.	.	.
GC13	16	727011	G	T	RHBDL1	Missense	p.W81L	6	89	6.7%	.	.	.
GC13	16	733402	C	A	JMJD8	Missense	p.W135L	6	68	8.8%	.	.	.
GC13	16	737700	C	A	WDR24	Missense	p.R174L	5	73	6.8%	.	.	.
GC13	16	772379	C	A	FAM173A	Missense	p.P160Q	7	76	9.2%	.	.	.
GC13	16	775480	C	A	CCDC78	Missense	p.R123L	5	48	10.4%	.	.	.
GC13	16	814941	C	A	MSLN	Missense	p.R139S	7	70	10.0%	.	.	.
GC13	16	816976	G	T	MSLN	Missense	p.G497W	8	56	14.3%	.	.	.
GC13	16	837446	C	A	RPUSD1	Missense	p.G72W	5	68	7.4%	.	.	.
GC13	16	842300	G	T	CHTF18	Missense	p.G427W	6	59	10.2%	.	O	.
GC13	16	1248662	G	T	CACNA1H	Missense	p.G231W	6	70	8.6%	.	.	.
GC13	16	1370518	G	T	UBE2I	Missense	p.C138F	6	71	8.5%	.	.	.
GC13	16	1536082	C	A	PTX4	Missense	p.R427L	6	92	6.5%	.	.	.

GC13	16	1555456	G	T	TELO2	Missense	p.G630W	6	78	7.7%	.	O	.
GC13	16	1995913	C	A	RPL3L	Missense	p.G324W	7	102	6.9%	.	.	.
GC13	16	2030539	G	T	NOXO1	Missense	p.P81Q	7	53	13.2%	.	.	.
GC13	16	2049944	C	A	ZNF598	Missense	p.G536W	6	41	14.6%	.	.	.
GC13	16	2070555	C	A	NPW	Missense	p.R145S	5	55	9.1%	.	.	.
GC13	16	2140689	G	T	PKD1	Missense	p.Q4041K	6	58	10.3%	.	.	.
GC13	16	2258560	C	A	MLST8	Missense	p.R270S	5	53	9.4%	.	.	.
GC13	16	2264016	G	T	PGP	Missense	p.Q227K	6	46	13.0%	.	.	.
GC13	16	2282297	C	A	E4F1	Missense	p.Q181K	6	69	8.7%	.	.	.
GC13	16	2511008	G	T	C16orf59	Missense	p.G130C	7	78	9.0%	.	.	.
GC13	16	2808479	G	T	SRRM2	Missense	p.R175L	7	118	5.9%	.	.	.
GC13	16	2811824	G	T	SRRM2	Missense	p.R432L	6	99	6.1%	.	.	.
GC13	16	2814712	G	T	SRRM2	Missense	p.G1395W	8	96	8.3%	.	.	.
GC13	16	2817554	G	T	SRRM2	Missense	p.R2342L	7	96	7.3%	.	.	.
GC13	16	3076526	C	A	THOC6	Missense	p.L141I	7	98	7.1%	.	O	.
GC13	16	3140365	G	T	ZSCAN10	Missense	p.P220Q	6	64	9.4%	.	.	.
GC13	16	3254253	G	T	OR1F1	Missense	p.G3W	6	84	7.1%	.	.	.
GC13	16	3274043	G	T	ZNF200	Missense	p.P345Q	9	125	7.2%	.	.	.
GC13	16	3333840	C	A	ZNF263	Missense	p.Q8K	7	44	15.9%	.	.	.
GC13	16	3339962	G	T	ZNF263	Missense	p.G486W	7	76	9.2%	.	.	.
GC13	16	3340415	G	T	ZNF263	Nonsense	p.G637X	7	90	7.8%	.	.	.
GC13	16	3366987	C	A	ZNF75A	Missense	p.P59Q	6	76	7.9%	.	.	.
GC13	16	3434470	G	T	ZSCAN32	Missense	p.P119Q	6	60	10.0%	.	.	.
GC13	16	3458689	G	T	ZNF174	Missense	p.G332W	8	94	8.5%	.	.	.
GC13	16	3534810	G	T	NAA60	Missense	p.G153W	6	79	7.6%	.	.	.
GC13	16	3586207	C	A	CLUAP1	Missense	p.P227Q	7	94	7.4%	.	.	.
GC13	16	3614938	C	A	NLRC3	Missense	p.G34W	6	70	8.6%	.	.	.
GC13	16	3725362	G	T	TRAP1	Missense	p.P231H	7	95	7.4%	.	.	.
GC13	16	3817880	G	T	CREBBP	Missense	p.Q993K	10	154	6.5%	O	.	.
GC13	16	4242560	C	A	SRL	Missense	p.R339L	7	90	7.8%	.	.	.
GC13	16	4242776	C	A	SRL	Missense	p.R267L	8	60	13.3%	.	.	.

GC13	16	4312563	G	T	TFAP4	Missense	p.H77N	7	82	8.5%	.	O	.
GC13	16	4312643	C	A	TFAP4	Missense	p.R50L	7	64	10.9%	.	.	.
GC13	16	4516180	G	A	NMRAL1	Missense	p.A168V	20	67	29.9%	.	.	.
GC13	16	4558134	C	A	HMOX2	Missense	p.L180M	7	90	7.8%	.	.	.
GC13	16	4751032	C	A	ANKS3	Missense	p.R235M	9	93	9.7%	.	.	.
GC13	16	4777134	G	T	ANKS3	Missense	p.P72Q	9	76	11.8%	.	.	.
GC13	16	4786570	C	A	C16orf71	Missense	p.Q19K	6	67	9.0%	.	.	.
GC13	16	4810525	G	T	ZNF500	Missense	p.P243Q	9	105	8.6%	.	.	.
GC13	16	4920230	G	T	UBN1	Missense	p.R400L	6	73	8.2%	.	.	.
GC13	16	4921237	C	A	UBN1	Missense	p.D547E	32	51	62.7%	.	.	.
GC13	16	5075540	C	A	NAGPA	Missense	p.G496V	7	76	9.2%	.	O	.
GC13	16	8862101	G	T	ABAT	Missense	p.G219W	6	48	12.5%	.	.	.
GC13	16	8988910	G	T	USP7	Missense	p.P990Q	6	85	7.1%	.	.	.
GC13	16	8997226	C	A	USP7	Missense	p.G564W	7	81	8.6%	.	.	.
GC13	16	10274266	C	A	GRIN2A	Missense	p.M1I	5	29	17.2%	O	.	.
GC13	16	11001145	G	T	CIITA	Missense	p.R599L	6	77	7.8%	.	.	.
GC13	16	11981492	G	T	GSPT1	Missense	p.P297T	7	92	7.6%	.	.	.
GC13	16	12450101	C	A	SNX29	Missense	p.Q622K	5	49	10.2%	.	.	.
GC13	16	12571619	G	T	SNX29	Missense	p.R694L	7	84	8.3%	.	.	.
GC13	16	12662379	G	T	SNX29	Nonsense	p.G779X	8	117	6.8%	.	.	.
GC13	16	14020425	G	T	ERCC4	Missense	p.L132F	9	129	7.0%	O	.	O
GC13	16	14031682	G	T	ERCC4	Missense	p.R624L	9	166	5.4%	O	.	.
GC13	16	14355187	G	T	MKL2	Missense	p.L1062F	8	113	7.1%	.	.	.
GC13	16	14723465	C	A	PARN	Missense	p.G29V	9	98	9.2%	.	.	.
GC13	16	15131916	C	A	NTAN1	Missense	p.W197L	8	95	8.4%	.	.	.
GC13	16	15727637	C	A	MARF1	Missense	p.W357L	9	139	6.5%	.	.	.
GC13	16	15788067	G	T	NDE1	Missense	p.R250L	6	77	7.8%	.	.	.
GC13	16	15813531	G	T	MYH11	Missense	p.R1665S	7	100	7.0%	.	.	.
GC13	16	15892541	C	A	MYH11	Missense	p.R169L	11	115	9.6%	.	.	.
GC13	16	16278886	C	A	ABCC6	Missense	p.G625W	6	46	13.0%	.	O	.
GC13	16	18856777	C	A	SMG1	Missense	p.G2065W	8	130	6.2%	.	.	.

GC13	16	18870983	C	A	SMG1	Missense	p.R1283M	7	91	7.7%	.	.	.
GC13	16	19020566	G	T	TMC7	Missense	p.R47L	8	144	5.6%	.	.	.
GC13	16	19126463	G	T	ITPRIPL2	Missense	p.R227L	6	80	7.5%	.	.	.
GC13	16	19127249	G	T	ITPRIPL2	Missense	p.R489L	6	93	6.5%	.	.	.
GC13	16	19127314	G	T	ITPRIPL2	Missense	p.G511W	7	94	7.4%	.	.	.
GC13	16	19127345	C	A	ITPRIPL2	Missense	p.P521Q	8	103	7.8%	.	.	.
GC13	16	19468044	C	A	TMC5	Missense	p.H6N	8	95	8.4%	.	.	.
GC13	16	19481056	G	T	TMC5	Missense	p.G318V	8	107	7.5%	.	.	.
GC13	16	19516203	G	T	GDE1	Missense	p.P252Q	9	98	9.2%	.	.	.
GC13	16	19516342	C	A	GDE1	Missense	p.G206W	8	108	7.4%	.	.	.
GC13	16	19554006	G	T	CCP110	Missense	p.W764L	10	147	6.8%	.	.	.
GC13	16	19619578	G	T	C16orf62	Missense	p.R377M	8	119	6.7%	.	.	.
GC13	16	19653744	C	A	C16orf62	Missense	p.P626Q	12	125	9.6%	.	.	.
GC13	16	19726320	G	T	KNOP1	Missense	p.P13Q	11	99	11.1%	.	.	.
GC13	16	19883326	C	A	GPRC5B	Missense	p.W412L	8	87	9.2%	.	.	.
GC13	16	20348001	G	T	UMOD	Missense	p.R597S	7	92	7.6%	.	.	.
GC13	16	20371965	C	A	PDILT	Missense	p.K477N	7	83	8.4%	.	.	.
GC13	16	20482995	C	A	ACSM2A	Missense	p.P214Q	8	135	5.9%	.	.	.
GC13	16	20748443	G	T	THUMPD1	Missense	p.P274Q	7	123	5.7%	.	.	.
GC13	16	20792142	C	A	ACSM3	Missense	p.H249N	10	138	7.2%	.	.	.
GC13	16	20856399	C	A	REXO5	Missense	p.Q623K	7	79	8.9%	.	.	.
GC13	16	20944667	C	A	DNAH3	Missense	p.G4008W	7	81	8.6%	.	O	.
GC13	16	20970695	C	A	DNAH3	Missense	p.M3498I	8	119	6.7%	.	.	.
GC13	16	20999070	C	A	DNAH3	Missense	p.R2230L	4	22	18.2%	.	.	.
GC13	16	21042492	G	T	DNAH3	Missense	p.L1726I	8	131	6.1%	.	.	.
GC13	16	21069433	G	T	DNAH3	Missense	p.Q1254K	8	99	8.1%	.	.	.
GC13	16	21185408	G	T	TMEM159	Missense	p.G115W	8	91	8.8%	.	.	.
GC13	16	21215479	C	A	ZP2	Missense	p.G282W	9	141	6.4%	.	.	.
GC13	16	21666602	G	T	METTL9	Missense	p.W268L	8	110	7.3%	.	.	.
GC13	16	21693087	C	A	OTOA	Missense	p.H70N	8	128	6.3%	.	.	.
GC13	16	21985277	C	A	UQCRC2	Missense	p.Q343K	8	124	6.5%	.	.	.

GC13	16	22128131	G	T	VWA3A	Missense	p.M289I	8	83	9.6%	.	.	.
GC13	16	22269054	G	T	EEF2K	Missense	p.R331L	6	41	14.6%	.	O	.
GC13	16	22337412	G	T	POLR3E	Missense	p.R524L	7	91	7.7%	.	.	.
GC13	16	23464225	C	A	COG7	Missense	p.G31W	9	77	11.7%	.	.	.
GC13	16	23563511	G	T	EARS2	Missense	p.P85H	8	94	8.5%	.	.	.
GC13	16	23578390	G	T	UBFD1	Missense	p.M273I	8	117	6.8%	.	.	.
GC13	16	23581891	G	T	UBFD1	Missense	p.G304W	8	116	6.9%	.	.	.
GC13	16	23649399	G	T	PALB2	Missense	p.R34S	8	146	5.5%	O	.	.
GC13	16	23706242	C	A	ERN2	Missense	p.R584L	6	83	7.2%	.	.	.
GC13	16	23716409	C	A	ERN2	Missense	p.G217W	6	36	16.7%	.	.	.
GC13	16	24043538	G	T	PRKCB	Nonsense	p.G124X	8	77	10.4%	.	.	.
GC13	16	24268094	G	T	CACNG3	Missense	p.G7C	8	98	8.2%	.	.	.
GC13	16	24373087	G	T	CACNG3	Missense	p.R284L	9	92	9.8%	.	O	.
GC13	16	24567756	C	A	RBBP6	Missense	p.P221Q	8	96	8.3%	.	.	.
GC13	16	24570842	G	T	RBBP6	Missense	p.G231W	6	76	7.9%	.	.	.
GC13	16	24580197	G	T	RBBP6	Missense	p.R695L	6	95	6.3%	.	.	.
GC13	16	24800837	C	A	TNRC6A	Missense	p.Q292K	10	123	8.1%	.	.	.
GC13	16	24801473	C	A	TNRC6A	Missense	p.L504I	9	114	7.9%	.	.	.
GC13	16	24828245	G	T	TNRC6A	Missense	p.R1598L	6	84	7.1%	.	O	.
GC13	16	24946914	C	A	ARHGAP17	Missense	p.G513W	7	56	12.5%	.	.	.
GC13	16	24963670	C	A	ARHGAP17	Missense	p.R329L	8	137	5.8%	.	.	.
GC13	16	25255588	C	A	ZKSCAN2	Missense	p.W500L	7	96	7.3%	.	.	.
GC13	16	25704369	C	A	HS3ST4	Missense	p.R211S	5	50	10.0%	.	.	.
GC13	16	27226238	C	A	KDM8	Missense	p.P274Q	7	65	10.8%	.	.	.
GC13	16	27549238	C	A	GTF3C1	Missense	p.G207W	10	78	12.8%	.	.	.
GC13	16	27556794	G	T	GTF3C1	Missense	p.P91Q	7	89	7.9%	.	.	.
GC13	16	27715279	G	T	KIAA0556	Missense	p.R450M	7	94	7.4%	.	.	.
GC13	16	27751863	G	T	KIAA0556	Missense	p.G749W	7	103	6.8%	.	.	.
GC13	16	28123200	C	A	XPO6	Missense	p.R760L	6	75	8.0%	.	.	.
GC13	16	28508073	C	A	APOBR	Missense	p.Q571K	7	98	7.1%	.	.	.
GC13	16	28508080	C	A	APOBR	Missense	p.P573Q	9	96	9.4%	.	.	.

GC13	16	28515303	C	A	IL27	Missense	p.G34W	7	93	7.5%	.	.	.
GC13	16	28854454	C	A	TUFM	Missense	p.G404W	5	39	12.8%	.	.	.
GC13	16	28855606	C	A	TUFM	Missense	p.K289N	8	126	6.3%	.	.	.
GC13	16	28856682	G	T	TUFM	Missense	p.R123S	6	70	8.6%	.	.	.
GC13	16	28898986	G	T	ATP2A1	Missense	p.G166W	7	86	8.1%	.	.	.
GC13	16	28899043	G	T	ATP2A1	Missense	p.G185C	10	122	8.2%	.	.	.
GC13	16	29675269	G	T	SPN	Nonsense	p.G74X	8	108	7.4%	.	.	.
GC13	16	29708685	C	A	QPRT	Missense	p.L138I	7	97	7.2%	.	.	.
GC13	16	29828299	G	T	PAGR1	Missense	p.E151D	34	91	37.4%	.	.	.
GC13	16	29830900	G	T	PAGR1	Missense	p.R197L	7	100	7.0%	.	.	.
GC13	16	29907064	G	T	SEZ6L2	Missense	p.P166H	5	36	13.9%	.	.	.
GC13	16	29912545	G	T	ASPHD1	Missense	p.G85W	8	93	8.6%	.	.	.
GC13	16	29974725	G	T	TMEM219	Missense	p.W57L	12	147	8.2%	.	.	.
GC13	16	29974775	G	T	TMEM219	Missense	p.G74W	8	103	7.8%	.	.	.
GC13	16	29992988	G	T	TAOK2	Missense	p.R221L	11	163	6.7%	.	.	.
GC13	16	30078929	G	T	ALDOA	Missense	p.G91W	8	87	9.2%	.	.	.
GC13	16	30080222	G	T	ALDOA	Missense	p.G155W	9	103	8.7%	.	.	.
GC13	16	30100331	C	A	TBX6	Missense	p.W185L	9	106	8.5%	.	.	.
GC13	16	30128492	G	T	MAPK3	Missense	p.P297H	10	131	7.6%	.	.	.
GC13	16	30369843	G	T	TBC1D10B	Missense	p.Q617K	9	108	8.3%	.	.	.
GC13	16	30409133	G	T	ZNF48	Missense	p.G65C	7	76	9.2%	.	.	.
GC13	16	30673906	G	T	FBRS	Missense	p.G261W	7	92	7.6%	.	.	.
GC13	16	30723266	C	A	SRCAP	Missense	p.Q535K	8	122	6.6%	.	.	.
GC13	16	30723623	G	T	SRCAP	Missense	p.R619L	8	147	5.4%	.	O	.
GC13	16	30740893	G	T	SRCAP	Nonsense	p.G2043X	8	123	6.5%	.	.	.
GC13	16	30745101	G	T	SRCAP	Missense	p.R2159L	7	101	6.9%	.	.	.
GC13	16	30748996	G	T	SRCAP	Missense	p.L2545F	8	116	6.9%	.	O	.
GC13	16	30780717	C	A	RNF40	Missense	p.Q720K	6	66	9.1%	.	.	.
GC13	16	30793926	C	A	ZNF629	Nonsense	p.G575X	7	82	8.5%	.	.	.
GC13	16	30794679	C	A	ZNF629	Missense	p.G324W	10	74	13.5%	.	.	.
GC13	16	30938960	C	A	FBXL19	Missense	p.Q150K	7	62	11.3%	.	.	.

GC13	16	30976229	G	T	SETD1A	Missense	p.R389L	9	103	8.7%	.	.	.
GC13	16	30980680	C	A	SETD1A	Missense	p.P942Q	6	91	6.6%	.	.	.
GC13	16	31008825	G	T	STX1B	Missense	p.Q118K	6	50	12.0%	.	.	.
GC13	16	31072858	G	T	ZNF668	Missense	p.P464Q	7	84	8.3%	.	.	.
GC13	16	31090064	C	A	ZNF646	Missense	p.H807N	7	90	7.8%	.	.	.
GC13	16	31104164	G	T	VKORC1	Missense	p.Q103K	6	46	13.0%	.	.	.
GC13	16	31123564	G	T	BCKDK	Missense	p.R406L	7	119	5.9%	.	O	.
GC13	16	31141660	G	T	KAT8	Missense	p.G329W	6	57	10.5%	.	.	.
GC13	16	31195262	G	T	FUS	Missense	p.G91W	6	59	10.2%	.	.	.
GC13	16	31195561	G	T	FUS	Missense	p.G122W	8	118	6.8%	.	.	.
GC13	16	31199652	G	T	FUS	Missense	p.R268L	8	107	7.5%	.	.	.
GC13	16	31201083	C	A	FUS	Missense	p.P344H	6	61	9.8%	.	.	.
GC13	16	31273087	G	T	ITGAM	Missense	p.G35W	8	111	7.2%	.	.	.
GC13	16	31388171	G	T	ITGAX	Missense	p.G854W	12	92	13.0%	.	.	.
GC13	16	31418987	G	T	ITGAD	Missense	p.G286W	8	98	8.2%	.	.	.
GC13	16	31501468	G	T	SLC5A2	Missense	p.R570L	4	34	11.8%	.	.	.
GC13	16	46696897	G	T	VPS35	Missense	p.Q609K	6	60	10.0%	.	.	.
GC13	16	46766377	G	T	MYLK3	Missense	p.P61Q	8	90	8.9%	.	.	.
GC13	16	46766545	C	A	MYLK3	Missense	p.G5V	6	32	18.8%	.	.	.
GC13	16	47196509	C	A	ITFG1	Missense	p.R394L	6	86	7.0%	.	.	.
GC13	16	47536930	C	A	PHKB	Missense	p.H112N	8	121	6.6%	.	.	.
GC13	16	47703222	C	A	PHKB	Missense	p.H842N	7	87	8.0%	.	.	.
GC13	16	47723012	G	T	PHKB	Missense	p.L897F	7	56	12.5%	.	.	.
GC13	16	47733139	G	T	PHKB	Splicing	c.3124-1G>T	10	120	8.3%	.	.	.
GC13	16	48119604	G	T	ABCC12	Missense	p.P1243Q	7	85	8.2%	.	.	.
GC13	16	48234270	G	T	ABCC11	Missense	p.H667N	6	51	11.8%	.	.	.
GC13	16	48595473	G	T	N4BP1	Missense	p.Q361K	8	113	7.1%	.	.	.
GC13	16	50261858	G	T	PAPD5	Missense	p.G622W	6	69	8.7%	.	.	.
GC13	16	50325743	C	A	ADCY7	Missense	p.H158N	6	47	12.8%	.	.	.
GC13	16	51173161	G	T	SALL1	Missense	p.P894H	8	91	8.8%	.	.	.
GC13	16	51173591	G	T	SALL1	Missense	p.Q751K	8	85	9.4%	.	.	.

GC13	16	51175472	C	A	SALL1	Missense	p.G124W	6	72	8.3%	.	.	.
GC13	16	51175744	G	T	SALL1	Missense	p.P33Q	8	62	12.9%	.	.	.
GC13	16	52484297	C	A	TOX3	Missense	p.L190F	8	72	11.1%	.	.	.
GC13	16	53190124	G	T	CHD9	Missense	p.L41F	7	93	7.5%	.	.	.
GC13	16	53321868	G	T	CHD9	Splicing	c.5190-1G>T	7	87	8.0%	.	.	.
GC13	16	53348922	C	A	CHD9	Missense	p.P2517Q	9	75	12.0%	.	.	.
GC13	16	53358278	C	A	CHD9	Missense	p.P2722Q	9	81	11.1%	.	.	.
GC13	16	53480995	G	T	RBL2	Missense	p.W205L	8	64	12.5%	.	.	.
GC13	16	53709023	C	A	RPGRIP1L	Missense	p.R263L	7	107	6.5%	.	.	.
GC13	16	55585013	G	T	LPCAT2	Missense	p.R405M	7	78	9.0%	.	.	.
GC13	16	55732462	G	T	SLC6A2	Nonsense	p.G386X	6	54	11.1%	.	.	.
GC13	16	55739143	G	T	SLC6A2	Missense	p.G616W	7	73	9.6%	.	.	.
GC13	16	56518743	C	A	BBS2	Missense	p.R699L	7	126	5.6%	.	.	.
GC13	16	56519504	C	A	BBS2	Missense	p.R686L	8	132	6.1%	.	.	.
GC13	16	57059900	G	T	NLRC5	Missense	p.G349W	7	71	9.9%	.	.	.
GC13	16	57188309	C	A	FAM192A	Missense	p.G220W	6	52	11.5%	.	.	.
GC13	16	57500138	G	T	POLR2C	Missense	p.R86L	8	87	9.2%	.	O	.
GC13	16	57608843	G	T	ADGRG5	Missense	p.G403W	5	42	11.9%	.	.	.
GC13	16	57707274	G	T	ADGRG3	Missense	p.G34W	7	82	8.5%	.	.	.
GC13	16	57784755	G	T	KATNB1	Missense	p.M102I	9	115	7.8%	.	.	.
GC13	16	57786790	C	T	KATNB1	Missense	p.L269F	30	77	39.0%	.	.	.
GC13	16	57945717	C	A	CNGB1	Missense	p.W805L	7	62	11.3%	.	.	.
GC13	16	58565909	G	T	CNOT1	Missense	p.H2039N	9	116	7.8%	.	.	.
GC13	16	58608624	C	A	CNOT1	Missense	p.R623L	6	74	8.1%	.	.	.
GC13	16	58615292	G	T	CNOT1	Missense	p.P391Q	8	114	7.0%	.	.	.
GC13	16	58701275	C	A	SLC38A7	Missense	p.G334W	7	67	10.4%	.	.	.
GC13	16	66527114	C	A	BEAN1	Missense	p.H133N	6	70	8.6%	.	.	.
GC13	16	66804004	G	T	TERB1	Missense	p.P494Q	8	147	5.4%	.	.	.
GC13	16	66811208	G	T	TERB1	Missense	p.H295N	7	84	8.3%	.	.	.
GC13	16	66966176	C	A	FAM96B	Missense	p.R141L	4	27	14.8%	.	.	.
GC13	16	67183596	G	T	B3GNT9	Missense	p.R265S	5	67	7.5%	.	.	.

GC13	16	67200243	G	T	HSF4	Missense	p.R169L	5	47	10.6%	.	.	.
GC13	16	67203586	G	T	HSF4	Missense	p.L459F	5	45	11.1%	.	.	.
GC13	16	67234153	G	T	ELMO3	Missense	p.R154L	6	75	8.0%	.	.	.
GC13	16	67265541	C	A	FHOD1	Missense	p.R795L	6	81	7.4%	.	.	.
GC13	16	67291314	C	A	SLC9A5	Missense	p.L367M	7	83	8.4%	.	.	.
GC13	16	67325336	C	A	KCTD19	Missense	p.W814L	7	73	9.6%	.	.	.
GC13	16	67333438	C	A	KCTD19	Missense	p.G272W	5	49	10.2%	.	.	.
GC13	16	67424205	G	T	TPPP3	Missense	p.H135N	7	79	8.9%	.	.	.
GC13	16	67440206	G	T	ZDHHC1	Missense	p.P50Q	7	73	9.6%	.	.	.
GC13	16	67575669	G	T	RIPOR1	Missense	p.R359L	7	93	7.5%	.	.	.
GC13	16	67655458	C	A	CTCF	Missense	p.H113N	8	120	6.7%	O	.	.
GC13	16	67662309	G	T	CTCF	Missense	p.G191W	8	70	11.4%	O	.	.
GC13	16	67709765	G	T	GFOD2	Missense	p.R151S	8	82	9.8%	.	.	.
GC13	16	67719557	G	T	GFOD2	Missense	p.P21Q	8	100	8.0%	.	.	.
GC13	16	67860371	G	T	TSNAXIP1	Missense	p.R90L	6	85	7.1%	.	.	.
GC13	16	67912648	C	A	EDC4	Missense	p.P398Q	8	104	7.7%	.	.	.
GC13	16	67919882	G	T	NRN1L	Missense	p.W73L	7	91	7.7%	.	.	.
GC13	16	67942803	G	T	PSKH1	Missense	p.G51W	6	68	8.8%	.	.	.
GC13	16	67970362	G	T	PSMB10	Missense	p.P28Q	7	87	8.0%	.	.	.
GC13	16	68055593	G	T	DDX28	Missense	p.H505N	9	75	12.0%	.	.	.
GC13	16	68156259	G	T	NFATC3	Missense	p.R158L	8	104	7.7%	.	.	.
GC13	16	68156892	G	T	NFATC3	Missense	p.R369L	7	75	9.3%	.	O	.
GC13	16	68225521	G	T	NFATC3	Missense	p.Q983H	8	105	7.6%	.	.	.
GC13	16	68710295	G	T	CDH3	Missense	p.M1I	6	68	8.8%	.	.	.
GC13	16	68714917	G	T	CDH3	Missense	p.G250V	6	64	9.4%	.	.	.
GC13	16	68718664	C	A	CDH3	Missense	p.P399H	7	55	12.7%	.	.	.
GC13	16	68842406	G	T	CDH1	Missense	p.W156L	8	99	8.1%	O	.	.
GC13	16	68934449	G	T	TANGO6	Missense	p.R497M	8	83	9.6%	.	.	.
GC13	16	69148573	C	A	HAS3	Missense	p.R356S	5	70	7.1%	.	.	.
GC13	16	69171746	C	A	UTP4	Missense	p.P49T	7	89	7.9%	.	.	.
GC13	16	69373760	C	A	NIP7	Missense	p.R10S	6	75	8.0%	.	.	.

GC13	16	69385655	A	T	TMED6	Missense	p.M1K	10	27	37.0%	.	.	.
GC13	16	69726927	G	T	NFAT5	Missense	p.G1049W	10	121	8.3%	.	.	.
GC13	16	69744910	G	T	NQO1	Missense	p.P193Q	8	100	8.0%	.	.	.
GC13	16	69748881	T	G	NQO1	Missense	p.K135Q	23	83	27.7%	.	.	.
GC13	16	69832663	C	A	WWP2	Missense	p.P50H	7	69	10.1%	.	.	.
GC13	16	69963428	C	A	WWP2	Missense	p.Q322K	5	38	13.2%	.	.	.
GC13	16	69964065	G	T	WWP2	Missense	p.W11L	6	61	9.8%	.	.	.
GC13	16	70166132	C	A	PDPR	Missense	p.P209Q	9	147	6.1%	.	.	.
GC13	16	70711769	G	T	MTSS1L	Missense	p.H220N	6	76	7.9%	.	.	.
GC13	16	70815823	C	A	VAC14	Missense	p.G65W	7	94	7.4%	.	.	.
GC13	16	70902522	C	A	HYDIN	Missense	p.W3754L	7	60	11.7%	.	.	.
GC13	16	71509507	G	T	ZNF19	Missense	p.H315N	8	106	7.5%	.	.	.
GC13	16	71570957	G	T	CHST4	Missense	p.W126L	8	84	9.5%	.	.	.
GC13	16	71668456	C	A	MARVELD3	Missense	p.P319Q	6	86	7.0%	.	.	.
GC13	16	71686785	C	A	PHLPP2	Missense	p.G842W	8	51	15.7%	.	.	.
GC13	16	71712680	G	T	PHLPP2	Missense	p.H416N	9	94	9.6%	.	.	.
GC13	16	72137071	C	A	DHX38	Missense	p.P533H	5	38	13.2%	.	.	.
GC13	16	72992410	C	A	ZFHX3	Missense	p.R545S	8	114	7.0%	O	O	.
GC13	16	74511424	C	A	GLG1	Missense	p.R601L	7	90	7.8%	.	.	.
GC13	16	75148776	G	T	LDHD	Missense	p.R140S	7	89	7.9%	.	.	.
GC13	16	75204164	G	T	ZFP1	Missense	p.G353W	8	82	9.8%	.	.	.
GC13	16	75563384	C	A	CHST5	Missense	p.R300L	4	27	14.8%	.	.	.
GC13	16	75563732	C	A	CHST5	Missense	p.R184L	5	48	10.4%	.	.	.
GC13	16	75646256	C	A	ADAT1	Nonsense	p.G223X	6	91	6.6%	.	.	.
GC13	16	76572114	G	T	CNTNAP4	Missense	p.G904C	7	67	10.4%	.	.	.
GC13	16	77228316	G	T	MON1B	Missense	p.R41L	6	83	7.2%	.	.	.
GC13	16	77325290	G	T	ADAMTS18	Missense	p.P920Q	9	104	8.7%	.	O	.
GC13	16	81094965	C	A	C16orf46	Missense	p.R330L	7	101	6.9%	.	.	.
GC13	16	81209319	G	T	PKD1L2	Missense	p.P140Q	7	83	8.4%	.	.	.
GC13	16	81396124	G	T	GAN	Missense	p.G332W	7	94	7.4%	.	.	.
GC13	16	83948579	G	T	MLYCD	Missense	p.G323W	8	92	8.7%	.	.	.

GC13	16	84012054	G	T	NECAB2	Missense	p.G78W	8	42	19.0%	.	.	.
GC13	16	84215834	G	T	TAF1C	Missense	p.P232H	8	96	8.3%	.	.	.
GC13	16	84256037	G	T	KCNG4	Missense	p.P449Q	5	46	10.9%	.	O	.
GC13	16	85012850	G	T	ZDHHC7	Missense	p.P161Q	7	90	7.8%	.	.	.
GC13	16	85667552	G	T	GSE1	Missense	p.G14W	6	44	13.6%	.	.	.
GC13	16	87451094	G	T	ZCCHC14	Missense	p.P315H	8	90	8.9%	.	.	.
GC13	16	88500663	G	T	ZNF469	Missense	p.W2234L	7	86	8.1%	.	.	.
GC13	16	88664598	G	T	ZC3H18	Missense	p.W234L	6	54	11.1%	.	.	.
GC13	16	88790368	C	A	PIEZO1	Missense	p.G1416W	6	79	7.6%	.	.	.
GC13	16	88808494	C	A	PIEZO1	Missense	p.W98L	6	54	11.1%	.	.	.
GC13	16	89169081	C	A	ACSF3	Missense	p.H246N	7	42	16.7%	.	.	.
GC13	16	89293342	C	A	ZNF778	Missense	p.Q188K	7	98	7.1%	.	.	.
GC13	16	89293358	G	T	ZNF778	Missense	p.R193L	6	97	6.2%	.	.	.
GC13	16	89293975	G	T	ZNF778	Missense	p.G399W	7	90	7.8%	.	.	.
GC13	16	89294731	G	T	ZNF778	Missense	p.G651W	8	50	16.0%	.	.	.
GC13	16	89348299	C	A	ANKRD11	Missense	p.G1551W	6	87	6.9%	.	.	.
GC13	16	89350431	C	A	ANKRD11	Missense	p.R840L	8	98	8.2%	.	.	.
GC13	16	89715875	G	T	CHMP1A	Missense	p.P39Q	6	80	7.5%	.	.	.
GC13	16	89757872	G	T	CDK10	Missense	p.R14L	6	63	9.5%	.	.	.
GC13	16	89971386	G	T	TCF25	Missense	p.G504W	6	49	12.2%	.	.	.
GC13	16	90038324	C	A	CENPBD1	Missense	p.G3W	5	50	10.0%	.	.	.
GC13	16	90104195	G	T	GAS8	Missense	p.R125M	11	151	7.3%	.	.	.
GC13	17	69464	G	T	RPH3AL	Missense	p.P197Q	7	80	8.8%	.	.	.
GC13	17	650390	C	A	GEMIN4	Missense	p.R298L	6	72	8.3%	.	.	.
GC13	17	679062	G	T	GLOD4	Missense	p.P120Q	6	81	7.4%	.	.	.
GC13	17	1028546	G	T	ABR	Missense	p.P27Q	8	54	14.8%	.	.	.
GC13	17	1340315	C	A	CRK	Nonsense	p.G126X	7	82	8.5%	.	.	.
GC13	17	1385804	C	A	MYO1C	Missense	p.G199W	7	86	8.1%	.	.	.
GC13	17	1586908	G	T	PRPF8	Missense	p.P63Q	7	79	8.9%	.	.	.
GC13	17	1655955	C	A	SERPINF2	Missense	p.Q248K	8	61	13.1%	.	O	.
GC13	17	1792131	C	A	RPA1	Missense	p.R513S	6	64	9.4%	.	.	.

GC13	17	1946126	C	A	OVCA2	Missense	p.R138S	6	81	7.4%	.	.	.
GC13	17	1968449	C	A	SMG6	Splicing	c.4048-1G>T;NM_001282326	6	48	12.5%	.	.	.
GC13	17	1989074	G	T	SMG6	Missense	p.P71Q	6	55	10.9%	.	.	.
GC13	17	2227563	G	T	TSR1	Missense	p.P781Q	8	99	8.1%	.	.	.
GC13	17	2227590	C	A	TSR1	Missense	p.R772L	6	98	6.1%	.	.	.
GC13	17	2234371	C	A	TSR1	Missense	p.R510L	6	98	6.1%	.	.	.
GC13	17	2237998	C	A	TSR1	Missense	p.R250L	6	75	8.0%	.	.	.
GC13	17	2323349	C	A	METTL16	Missense	p.W535L	6	83	7.2%	.	.	.
GC13	17	2995845	C	A	OR1D2	Missense	p.W149L	8	98	8.2%	.	.	.
GC13	17	3101073	G	T	OR1A2	Missense	p.L87F	8	130	6.2%	.	.	.
GC13	17	3101095	G	T	OR1A2	Missense	p.G95W	8	123	6.5%	.	.	.
GC13	17	3119360	G	T	OR1A1	Missense	p.W149L	8	81	9.9%	.	.	.
GC13	17	3181473	G	T	OR3A2	Missense	p.H253N	6	74	8.1%	.	.	.
GC13	17	3343619	G	T	SPATA22	Missense	p.P262Q	6	88	6.8%	.	O	.
GC13	17	3558394	G	T	CTNS	Missense	p.G110C	6	85	7.1%	.	.	.
GC13	17	3629297	C	A	HASPIN	Missense	p.L690M	8	102	7.8%	.	.	.
GC13	17	3631314	C	A	ITGAE	Missense	p.G995W	9	143	6.3%	.	.	.
GC13	17	3728289	G	T	NCBP3	Missense	p.P248Q	8	109	7.3%	.	.	.
GC13	17	3992137	C	A	ZZEF1	Missense	p.L692F	7	91	7.7%	.	.	.
GC13	17	4186168	G	T	UBE2G1	Missense	p.R159S	8	103	7.8%	.	.	.
GC13	17	4446305	C	A	MYBBP1A	Missense	p.R932L	5	60	8.3%	.	.	.
GC13	17	4457340	G	T	MYBBP1A	Missense	p.L143I	8	95	8.4%	.	.	.
GC13	17	4619480	C	A	ARRB2	Missense	p.R52S	4	25	16.0%	.	.	.
GC13	17	4642189	C	A	CXCL16	Nonsense	p.G55X	5	50	10.0%	.	.	.
GC13	17	4722380	G	T	PLD2	Missense	p.M725I	5	41	12.2%	.	.	.
GC13	17	4784551	G	T	MINK1	Missense	p.W102L	6	53	11.3%	.	.	.
GC13	17	4804410	C	A	CHRNE	Missense	p.G226V	7	69	10.1%	.	.	.
GC13	17	4883087	C	A	CAMTA2	Missense	p.M512I	7	74	9.5%	.	.	.
GC13	17	4936561	G	T	SLC52A1	Missense	p.L377I	7	71	9.9%	.	.	.
GC13	17	4995013	G	T	ZFP3	Missense	p.G72W	7	93	7.5%	.	.	.
GC13	17	4996186	C	A	ZFP3	Missense	p.Q463K	7	87	8.0%	.	.	.

GC13	17	5009124	G	T	ZNF232	Missense	p.H435N	7	66	10.6%	.	.	.
GC13	17	5012229	G	T	ZNF232	Missense	p.P206Q	11	87	12.6%	.	.	.
GC13	17	5087368	G	T	ZNF594	Missense	p.Q62K	11	111	9.9%	.	.	.
GC13	17	5341522	G	T	C1QBP	Missense	p.L102I	6	60	10.0%	.	.	.
GC13	17	5463043	G	T	NLRP1	Missense	p.Q325K	9	129	7.0%	.	.	.
GC13	17	5991399	C	A	WSCD1	Missense	p.H173N	7	66	10.6%	.	.	.
GC13	17	6553690	G	T	MED31	Missense	p.P31Q	8	81	9.9%	.	.	.
GC13	17	6596458	G	T	SLC13A5	Missense	p.P351T	6	43	14.0%	.	.	.
GC13	17	6909220	G	T	ALOX12	Missense	p.R431L	6	43	14.0%	.	.	.
GC13	17	6941957	C	A	SLC16A13	Missense	p.P277Q	7	78	9.0%	.	.	.
GC13	17	7096389	G	T	DLG4	Missense	p.H521N	6	74	8.1%	.	.	.
GC13	17	7129903	C	A	DVL2	Missense	p.Q533H	6	52	11.5%	.	.	.
GC13	17	7139548	G	T	PHF23	Missense	p.P166Q	9	72	12.5%	.	.	.
GC13	17	7226990	G	T	NEURL4	Missense	p.R772S	7	86	8.1%	.	.	.
GC13	17	7306996	G	T	TMEM256	Missense	p.P33Q	7	66	10.6%	.	.	.
GC13	17	7324485	G	T	SPEM1	Missense	p.W164L	5	46	10.9%	.	.	.
GC13	17	7324809	G	T	SPEM1	Missense	p.R272L	8	69	11.6%	.	.	.
GC13	17	7330329	G	T	C17orf74	Missense	p.R340L	9	74	12.2%	.	.	.
GC13	17	7367039	C	A	ZBTB4	Missense	p.R421L	5	51	9.8%	.	.	.
GC13	17	7412910	C	A	POLR2A	Missense	p.R1258S	6	75	8.0%	.	.	.
GC13	17	7463773	C	A	TNFSF13	Missense	p.P177Q	5	40	12.5%	.	O	.
GC13	17	7497323	C	A	FXR2	Splicing	c.1021-1G>T	7	86	8.1%	.	.	.
GC13	17	7592169	G	T	WRAP53	Missense	p.R68L	6	97	6.2%	.	.	.
GC13	17	7636457	G	T	DNAH2	Missense	p.W151L	9	76	11.8%	.	.	.
GC13	17	7640548	G	T	DNAH2	Missense	p.R381L	6	46	13.0%	.	O	.
GC13	17	7696440	C	A	DNAH2	Missense	p.Q2496K	8	96	8.3%	.	.	.
GC13	17	7722377	G	T	DNAH2	Missense	p.R3604L	6	61	9.8%	.	.	.
GC13	17	7726770	G	T	DNAH2	Missense	p.R3718L	9	115	7.8%	.	.	.
GC13	17	7736432	G	T	DNAH2	Missense	p.W4341L	6	59	10.2%	.	.	.
GC13	17	7752601	C	A	KDM6B	Missense	p.R999S	5	48	10.4%	.	.	.
GC13	17	8052884	C	A	PER1	Missense	p.R250L	6	76	7.9%	.	.	.

GC13	17	8092431	C	A	BORCS6	Missense	p.R343L	8	123	6.5%	.	.	.
GC13	17	8093445	C	A	BORCS6	Missense	p.R5L	6	88	6.8%	.	.	.
GC13	17	8135445	C	A	CTC1	Missense	p.G721C	6	62	9.7%	.	.	.
GC13	17	8159189	C	A	PFAS	Missense	p.P214Q	7	78	9.0%	.	O	.
GC13	17	8218449	G	T	ARHGEF15	Missense	p.G372W	8	64	12.5%	.	.	.
GC13	17	8219405	C	A	ARHGEF15	Missense	p.P548Q	6	79	7.6%	.	.	.
GC13	17	8243401	C	A	ODF4	Missense	p.P11H	7	72	9.7%	.	.	.
GC13	17	8272651	C	A	KRBA2	Missense	p.R345L	8	115	7.0%	.	.	.
GC13	17	8387470	G	T	MYH10	Missense	p.L1690I	10	120	8.3%	.	.	.
GC13	17	8508216	C	A	MYH10	Missense	p.G144W	8	102	7.8%	.	.	.
GC13	17	8661675	G	T	SPDYE4	Missense	p.P9Q	6	59	10.2%	.	.	.
GC13	17	8784256	G	T	PIK3R5	Missense	p.Q435K	7	85	8.2%	.	.	.
GC13	17	9490031	G	T	CFAP52	Missense	p.W28L	8	106	7.5%	.	.	.
GC13	17	9631600	G	T	USP43	Missense	p.G884W	6	74	8.1%	.	.	.
GC13	17	10236391	C	A	MYH13	Missense	p.R725L	5	60	8.3%	.	.	.
GC13	17	10304684	G	T	MYH8	Missense	p.H1006N	9	100	9.0%	.	O	.
GC13	17	10400624	C	A	MYH1	Missense	p.R1504L	9	101	8.9%	.	.	.
GC13	17	10418209	C	A	MYH1	Missense	p.R170L	9	109	8.3%	.	.	.
GC13	17	10424643	C	A	MYH2	Missense	p.R1927L	6	99	6.1%	.	.	.
GC13	17	10538301	C	A	MYH3	Missense	p.Q1404H	7	98	7.1%	.	.	.
GC13	17	11572435	C	A	DNAH9	Missense	p.P929Q	8	74	10.8%	.	.	.
GC13	17	11837351	C	A	DNAH9	Missense	p.P463Q	6	76	7.9%	.	.	.
GC13	17	11865466	C	A	DNAH9	Missense	p.L688M	7	76	9.2%	.	.	.
GC13	17	12647511	G	T	MYOCD	Missense	p.L243F	7	88	8.0%	.	.	.
GC13	17	12860015	G	T	ARHGAP44	Missense	p.G232W	7	88	8.0%	.	.	.
GC13	17	12860036	C	A	ARHGAP44	Missense	p.Q239K	7	86	8.1%	.	.	.
GC13	17	15965099	G	T	NCOR1	Missense	p.Q1849K	8	85	9.4%	O	.	.
GC13	17	16137346	G	T	PIGL	Missense	p.L99F	7	74	9.5%	.	.	.
GC13	17	16327069	G	T	TRPV2	Missense	p.E304D	7	66	10.6%	.	.	.
GC13	17	16456288	G	T	ZNF287	Missense	p.H342N	9	121	7.4%	.	.	.
GC13	17	16467100	G	T	ZNF287	Missense	p.Q133K	7	83	8.4%	.	.	.

GC13	17	16526368	G	T	ZNF624	Missense	p.P611Q	7	86	8.1%	.	.	.
GC13	17	16526600	C	A	ZNF624	Missense	p.G534W	7	78	9.0%	.	.	.
GC13	17	17174239	C	A	COPS3	Missense	p.G23V	8	132	6.1%	.	.	.
GC13	17	17696282	G	T	RAI1	Missense	p.R7M	7	91	7.7%	.	.	.
GC13	17	17701198	G	T	RAI1	Missense	p.G1646W	7	89	7.9%	.	.	.
GC13	17	17719673	C	A	SREBF1	Missense	p.G664W	6	41	14.6%	.	.	.
GC13	17	18136029	G	T	LLGL1	Missense	p.W102L	6	68	8.8%	.	.	.
GC13	17	18155017	G	T	FLII	Missense	p.Q399K	8	85	9.4%	.	.	.
GC13	17	18218074	G	T	TOP3A	Missense	p.R7S	7	54	13.0%	.	.	.
GC13	17	18219251	G	T	SMCR8	Missense	p.G50W	7	74	9.5%	.	.	.
GC13	17	18220100	C	A	SMCR8	Missense	p.Q333K	6	80	7.5%	.	.	.
GC13	17	18638564	G	T	TRIM16L	Missense	p.G280W	6	61	9.8%	.	.	.
GC13	17	18874740	C	A	FAM83G	Missense	p.G802C	7	55	12.7%	.	.	.
GC13	17	19186506	G	T	EPN2	Missense	p.R25L	7	69	10.1%	.	.	.
GC13	17	19216592	G	T	EPN2	Missense	p.G98C	7	89	7.9%	.	.	.
GC13	17	19284025	G	T	MAPK7	Missense	p.R29L	8	77	10.4%	.	.	.
GC13	17	19458973	G	T	SLC47A1	Missense	p.G237W	7	80	8.8%	.	.	.
GC13	17	19470152	C	A	SLC47A1	Missense	p.H386N	7	95	7.4%	.	.	.
GC13	17	19561069	G	T	ALDH3A2	Missense	p.W231L	7	98	7.1%	.	.	.
GC13	17	19748627	G	T	ULK2	Missense	p.P178Q	6	98	6.1%	.	.	.
GC13	17	20109031	C	A	SPECC1	Missense	p.H476N	8	102	7.8%	.	.	.
GC13	17	21318834	G	T	KCNJ12	Missense	p.M60I	14	261	5.4%	.	O	.
GC13	17	26369954	G	T	NLK	Missense	p.G19C	6	77	7.8%	.	.	.
GC13	17	26370357	G	T	NLK	Missense	p.W153L	6	84	7.1%	.	.	.
GC13	17	26652572	C	A	TMEM97	Missense	p.P57Q	6	75	8.0%	.	.	.
GC13	17	26729334	C	A	SLC46A1	Missense	p.G363W	7	81	8.6%	.	.	.
GC13	17	26919001	C	A	SPAG5	Missense	p.G421C	6	71	8.5%	.	.	.
GC13	17	26919238	G	T	SPAG5	Missense	p.P342T	10	89	11.2%	.	.	.
GC13	17	26919310	G	T	SPAG5	Missense	p.Q318K	7	97	7.2%	.	.	.
GC13	17	26940303	G	T	SGK494	Missense	p.Q129K	7	93	7.5%	.	.	.
GC13	17	27065727	G	T	NEK8	Missense	p.G421W	7	70	10.0%	.	.	.

GC13	17	27076506	C	A	TRAF4	Missense	p.H442N	8	90	8.9%	.	.	.
GC13	17	27209435	G	T	FLOT2	Missense	p.L167M	8	106	7.5%	.	.	.
GC13	17	27238143	C	A	PHF12	Missense	p.M734I	7	80	8.8%	.	.	.
GC13	17	27493600	C	A	MYO18A	Missense	p.R120L	6	91	6.6%	.	.	.
GC13	17	27939745	G	T	ANKRD13B	Missense	p.R496L	4	26	15.4%	.	.	.
GC13	17	27963027	G	T	SSH2	Missense	p.H714N	8	102	7.8%	.	.	.
GC13	17	27977771	G	T	SSH2	Missense	p.P349Q	10	104	9.6%	.	.	.
GC13	17	27994194	C	A	SSH2	Missense	p.R259M	8	115	7.0%	.	.	.
GC13	17	28011678	G	T	SSH2	Missense	p.Q108K	7	97	7.2%	.	.	.
GC13	17	28296073	C	A	EFCAB5	Missense	p.P96H	10	144	6.9%	.	.	.
GC13	17	28512180	C	A	NSRP1	Missense	p.Q335K	9	92	9.8%	.	.	.
GC13	17	28512565	C	A	NSRP1	Missense	p.P463H	9	118	7.6%	.	.	.
GC13	17	28754532	G	T	CPD	Missense	p.W411L	8	131	6.1%	.	.	.
GC13	17	28754547	C	A	CPD	Missense	p.P416Q	9	118	7.6%	.	.	.
GC13	17	28776764	C	A	CPD	Missense	p.Q776K	5	39	12.8%	.	.	.
GC13	17	28811257	G	T	GOSR1	Nonsense	p.G58X	7	100	7.0%	.	.	.
GC13	17	29161335	G	T	ATAD5	Missense	p.G79V	8	115	7.0%	.	.	.
GC13	17	29206444	C	A	ATAD5	Missense	p.P1339Q	8	70	11.4%	.	.	.
GC13	17	29220871	G	T	ATAD5	Missense	p.R1667M	10	141	7.1%	.	.	.
GC13	17	29231160	C	A	TEFM	Missense	p.R140L	10	75	13.3%	.	.	.
GC13	17	29664506	G	T	NF1	Missense	p.R2162L	8	92	8.7%	O	.	.
GC13	17	30221750	C	A	UTP6	Nonsense	p.G76X	7	97	7.2%	.	.	.
GC13	17	30685550	C	A	ZNF207	Missense	p.P66Q	7	90	7.8%	.	.	.
GC13	17	31618778	C	A	ASIC2	Missense	p.R119L	6	59	10.2%	.	.	.
GC13	17	32953254	G	T	TMEM132E	Missense	p.R59L	4	27	14.8%	.	.	.
GC13	17	33279029	G	T	CCT6B	Missense	p.P140H	7	93	7.5%	.	.	.
GC13	17	33319614	G	T	LIG3	Missense	p.R453L	6	93	6.5%	.	.	.
GC13	17	33462351	C	A	NLE1	Missense	p.Q85H	7	85	8.2%	.	.	.
GC13	17	33513568	C	A	UNC45B	Missense	p.P848Q	7	82	8.5%	.	.	.
GC13	17	33769198	G	T	SLFN13	Missense	p.Q436K	7	96	7.3%	.	.	.
GC13	17	33902949	C	A	PEX12	Missense	p.R311L	9	102	8.8%	.	.	.

GC13	17	33904286	G	T	PEX12	Missense	p.R151S	6	91	6.6%	.	.	.
GC13	17	34073384	C	A	GAS2L2	Missense	p.G378W	5	30	16.7%	.	.	.
GC13	17	34257715	G	T	RDM1	Missense	p.P6H	9	97	9.3%	.	.	.
GC13	17	34340940	C	A	CCL23	Missense	p.R49M	6	61	9.8%	.	.	.
GC13	17	34893437	G	T	PIGW	Missense	p.G163W	8	119	6.7%	.	.	.
GC13	17	34893834	G	T	PIGW	Missense	p.R295L	8	103	7.8%	.	.	.
GC13	17	35445877	G	T	ACACA	Missense	p.R2247S	7	88	8.0%	.	.	.
GC13	17	35634796	G	T	ACACA	Missense	p.P143Q	12	107	11.2%	.	.	.
GC13	17	35802721	G	T	TADA2A	Missense	p.G167W	9	114	7.9%	.	.	.
GC13	17	35913378	G	T	SYNRG	Missense	p.P655H	7	96	7.3%	.	.	.
GC13	17	35986077	G	T	DDX52	Missense	p.Q334K	7	96	7.3%	.	.	.
GC13	17	36002220	G	T	DDX52	Missense	p.Q69K	7	94	7.4%	.	.	.
GC13	17	36476512	G	T	MRPL45	Missense	p.W174L	7	98	7.1%	.	.	.
GC13	17	36483573	C	A	GPR179	Missense	p.W1960L	10	108	9.3%	.	.	.
GC13	17	36483889	G	T	GPR179	Missense	p.L1855M	10	105	9.5%	.	.	.
GC13	17	36485244	G	T	GPR179	Missense	p.P1403H	7	94	7.4%	.	.	.
GC13	17	36623044	C	A	ARHGAP23	Missense	p.R374S	4	37	10.8%	.	.	.
GC13	17	36623240	G	T	ARHGAP23	Missense	p.R439L	6	88	6.8%	.	.	.
GC13	17	37107836	G	T	FBXO47	Missense	p.P205Q	9	98	9.2%	.	.	.
GC13	17	37316930	C	A	ARL5C	Missense	p.M135I	6	65	9.2%	.	.	.
GC13	17	37566835	C	A	MED1	Missense	p.G547W	7	78	9.0%	.	.	.
GC13	17	37571506	G	T	MED1	Missense	p.L462M	7	91	7.7%	.	O	.
GC13	17	37619285	G	T	CDK12	Missense	p.G321W	7	67	10.4%	O	O	.
GC13	17	37627997	G	T	CDK12	Nonsense	p.G638X	7	97	7.2%	O	.	.
GC13	17	37882874	C	A	ERBB2	Missense	p.R978S	7	69	10.1%	O	.	.
GC13	17	37902195	G	T	GRB7	Missense	p.G434W	6	66	9.1%	.	.	.
GC13	17	38146041	G	T	PSMD3	Missense	p.G246W	7	90	7.8%	.	.	.
GC13	17	38251792	C	A	NR1D1	Missense	p.G385W	7	35	20.0%	.	.	.
GC13	17	38289911	G	T	MSL1	Missense	p.V298F	7	99	7.1%	.	.	.
GC13	17	38320300	G	T	CASC3	Missense	p.W451L	7	82	8.5%	.	.	.
GC13	17	38348474	G	T	RAPGEFL1	Missense	p.G388W	7	89	7.9%	.	O	.

GC13	17	38447480	C	A	CDC6	Missense	p.Q117K	9	138	6.5%	.	.	.
GC13	17	38569206	C	A	TOP2A	Missense	p.M198I	7	96	7.3%	.	.	.
GC13	17	38938700	C	A	KRT27	Missense	p.G16W	7	67	10.4%	.	.	.
GC13	17	38990785	C	A	TMEM99	Missense	p.P6Q	7	74	9.5%	.	.	.
GC13	17	39672439	G	T	KRT15	Missense	p.R273S	6	85	7.1%	.	.	.
GC13	17	39890639	G	T	HAP1	Missense	p.P83Q	6	69	8.7%	.	.	.
GC13	17	40332903	G	T	KCNH4	Missense	p.R21S	4	26	15.4%	.	.	.
GC13	17	40486033	G	T	STAT3	Missense	p.R278S	7	114	6.1%	O	.	.
GC13	17	40665973	G	T	ATP6V0A1	Missense	p.W743L	7	89	7.9%	.	.	.
GC13	17	40673073	G	T	ATP6V0A1	Missense	p.G818W	8	87	9.2%	.	.	.
GC13	17	40692998	G	T	NAGLU	Missense	p.M265I	7	81	8.6%	.	.	.
GC13	17	40822620	C	A	PLEKHH3	Missense	p.G509C	5	46	10.9%	.	.	.
GC13	17	41001637	C	A	AOC2	Missense	p.Q605K	8	96	8.3%	.	.	.
GC13	17	41245455	G	T	BRCA1	Missense	p.P651Q	8	123	6.5%	O	.	.
GC13	17	41597503	G	T	DHX8	Missense	p.M844I	7	80	8.8%	.	.	.
GC13	17	41610248	C	A	ETV4	Missense	p.R163L	7	63	11.1%	.	.	.
GC13	17	41956695	C	A	MPP2	Missense	p.R466L	6	75	8.0%	.	.	.
GC13	17	41959741	G	T	MPP2	Missense	p.R187S	5	48	10.4%	.	.	.
GC13	17	42225968	G	T	C17orf53	Missense	p.W266L	8	113	7.1%	.	.	.
GC13	17	42334783	G	T	SLC4A1	Missense	p.Q521K	8	106	7.5%	.	.	.
GC13	17	42430123	G	T	GRN	Missense	p.W580L	6	65	9.2%	.	.	.
GC13	17	42461475	A	T	ITGA2B	Missense	p.L308Q	8	45	17.8%	.	.	.
GC13	17	42476249	G	T	GPATCH8	Missense	p.Q988K	7	97	7.2%	.	.	.
GC13	17	42477331	C	A	GPATCH8	Missense	p.G627V	9	134	6.7%	.	.	.
GC13	17	42478162	G	T	GPATCH8	Missense	p.P350Q	8	118	6.8%	.	.	.
GC13	17	42744265	C	A	MEIOC	Missense	p.P329Q	8	105	7.6%	.	.	.
GC13	17	42756345	C	A	CCDC43	Missense	p.R185L	8	108	7.4%	.	.	.
GC13	17	42854140	G	T	ADAM11	Missense	p.G332C	6	52	11.5%	.	.	.
GC13	17	42882443	C	A	GJC1	Missense	p.W248L	9	119	7.6%	.	.	.
GC13	17	42979976	G	T	CCDC103	Missense	p.G174W	7	87	8.0%	.	.	.
GC13	17	43322192	C	A	FMNL1	Missense	p.L842M	7	85	8.2%	.	.	.

GC13	17	43474054	C	A	ARHGAP27	Missense	p.R685L	6	62	9.7%	.	O	.
GC13	17	43923312	G	T	SPPL2C	Missense	p.W347L	7	67	10.4%	.	O	.
GC13	17	44791233	G	T	NSF	Missense	p.G548W	6	75	8.0%	.	.	.
GC13	17	44803981	G	T	NSF	Missense	p.D610Y	8	110	7.3%	.	.	.
GC13	17	44952517	C	A	WNT9B	Missense	p.H129N	9	88	10.2%	.	.	.
GC13	17	45299107	C	A	MYL4	Missense	p.R125S	7	114	6.1%	.	.	.
GC13	17	45452246	C	A	EFCAB13	Missense	p.P333Q	9	126	7.1%	.	.	.
GC13	17	45481978	C	A	EFCAB13	Missense	p.Q642K	7	91	7.7%	.	.	.
GC13	17	45745700	G	T	KPNB1	Missense	p.R238L	7	92	7.6%	.	.	.
GC13	17	45911832	G	T	LRRC46	Missense	p.R53L	11	101	10.9%	.	.	.
GC13	17	45913431	C	A	LRRC46	Missense	p.L139M	7	87	8.0%	.	.	.
GC13	17	46620639	C	A	HOXB2	Missense	p.G288W	4	37	10.8%	.	.	.
GC13	17	46847332	G	A	TTLL6	Missense	p.S416F	20	81	24.7%	.	.	.
GC13	17	46868906	C	A	TTLL6	Missense	p.W46L	7	84	8.3%	.	O	.
GC13	17	46878729	C	A	TTLL6	Missense	p.G167W	9	110	8.2%	.	.	.
GC13	17	46928966	G	T	CALCOCO2	Missense	p.M184I	8	107	7.5%	.	.	.
GC13	17	46990358	G	T	UBE2Z	Missense	p.G185W	10	152	6.6%	.	.	.
GC13	17	47489163	G	T	PHB	Missense	p.R43S	5	52	9.6%	.	.	.
GC13	17	47656430	G	T	NXPH3	Missense	p.R176L	6	68	8.8%	.	.	.
GC13	17	48267077	G	T	COL1A1	Missense	p.P877H	8	74	10.8%	.	.	.
GC13	17	48539638	C	A	ACSF2	Missense	p.Q41K	7	73	9.6%	.	.	.
GC13	17	48618379	G	T	EPN3	Missense	p.G402V	6	58	10.3%	.	.	.
GC13	17	48628194	G	T	SPATA20	Missense	p.L417F	6	56	10.7%	.	.	.
GC13	17	48650036	G	T	CACNA1G	Missense	p.G290W	7	55	12.7%	.	.	.
GC13	17	48735442	G	T	ABCC3	Splicing	c.487-1G>T	7	64	10.9%	.	.	.
GC13	17	48765027	C	A	ABCC3	Missense	p.Q1471K	7	80	8.8%	.	.	.
GC13	17	49067123	G	T	SPAG9	Missense	p.Q753K	9	97	9.3%	.	O	.
GC13	17	49302425	G	T	MBTD1	Missense	p.P33Q	6	91	6.6%	.	.	.
GC13	17	49354497	G	T	UTP18	Missense	p.G282W	4	20	20.0%	.	.	.
GC13	17	49357405	C	A	UTP18	Missense	p.P351Q	7	72	9.7%	.	.	.
GC13	17	51901556	G	T	KIF2B	Missense	p.G388W	7	85	8.2%	.	O	.

GC13	17	54925341	C	A	DGKE	Missense	p.P268Q	9	99	9.1%	.	.	.
GC13	17	55028049	G	T	COIL	Missense	p.P185Q	9	134	6.7%	.	.	.
GC13	17	56083740	G	T	SRSF1	Missense	p.P115T	8	114	7.0%	.	.	.
GC13	17	56294085	G	T	MKS1	Missense	p.P58Q	8	97	8.2%	.	.	.
GC13	17	56296029	C	A	MKS1	Missense	p.G38W	6	83	7.2%	.	.	.
GC13	17	56296830	G	T	MKS1	Missense	p.R15S	5	65	7.7%	.	.	.
GC13	17	56327861	G	T	LPO	Missense	p.W137L	8	76	10.5%	.	.	.
GC13	17	56356929	G	T	MPO	Missense	p.P168Q	6	82	7.3%	.	O	.
GC13	17	56598465	G	T	SEPT4	Missense	p.H383N	6	62	9.7%	.	O	.
GC13	17	56620233	G	T	C17orf47	Missense	p.Q439K	8	77	10.4%	.	.	.
GC13	17	57049557	G	T	PPM1E	Missense	p.W346L	6	74	8.1%	.	.	.
GC13	17	57287809	C	A	SMG8	Missense	p.Q133K	7	88	8.0%	.	.	.
GC13	17	57351022	G	T	GDPD1	Missense	p.W278L	6	89	6.7%	.	.	.
GC13	17	57679896	C	A	DHX40	Missense	p.P530Q	8	104	7.7%	.	.	.
GC13	17	58040355	C	A	RNFT1	Missense	p.R116L	7	92	7.6%	.	.	.
GC13	17	58121175	C	A	HEATR6	Missense	p.G1099W	8	91	8.8%	.	.	.
GC13	17	58123420	C	A	HEATR6	Missense	p.M980I	8	106	7.5%	.	.	.
GC13	17	58267932	G	T	USP32	Missense	p.Q1212K	10	95	10.5%	.	.	.
GC13	17	58533708	G	T	APPBP2	Missense	p.H295N	8	111	7.2%	.	.	.
GC13	17	58678202	C	A	PPM1D	Missense	p.R143S	9	124	7.3%	O	.	.
GC13	17	58946044	G	T	BCAS3	Missense	p.R195L	8	96	8.3%	.	.	.
GC13	17	59946698	G	T	INTS2	Missense	p.P1025Q	8	114	7.0%	.	.	.
GC13	17	59946742	C	A	INTS2	Splicing	c.3031-1G>T;NM_020748	7	92	7.6%	.	.	.
GC13	17	59967256	C	A	INTS2	Splicing	c.1876-1G>T;NM_020748	7	76	9.2%	.	.	.
GC13	17	60503680	C	A	METTL2A	Missense	p.H75N	7	93	7.5%	.	.	.
GC13	17	60679541	G	T	TLK2	Missense	p.W588L	5	31	16.1%	.	.	.
GC13	17	60813634	G	T	MARCH10	Missense	p.P532Q	10	107	9.3%	.	.	.
GC13	17	60865926	G	T	MARCH10	Missense	p.P42Q	8	122	6.6%	.	.	.
GC13	17	61417589	G	T	TANC2	Missense	p.R494L	7	92	7.6%	.	.	.
GC13	17	61457113	C	A	TANC2	Missense	p.R799S	7	107	6.5%	.	.	.

GC13	17	61557214	C	A	ACE	Missense	p.P199Q	6	86	7.0%	.	.	.
GC13	17	61566111	C	A	ACE	Missense	p.P229Q	6	79	7.6%	.	O	.
GC13	17	61894290	G	T	DDX42	Missense	p.M692I	9	88	10.2%	.	.	.
GC13	17	61895718	C	A	DDX42	Missense	p.P926Q	6	71	8.5%	.	.	.
GC13	17	62018771	C	A	SCN4A	Missense	p.W1624L	7	86	8.1%	.	.	.
GC13	17	62272310	G	T	TEX2	Missense	p.P597H	6	61	9.8%	.	.	.
GC13	17	63010673	C	A	GNA13	Missense	p.R184L	7	130	5.4%	.	.	.
GC13	17	63200351	G	T	RGS9	Missense	p.G376W	5	37	13.5%	.	.	.
GC13	17	65185705	C	A	HELZ	Missense	p.K288N	11	107	10.3%	.	.	.
GC13	17	65907317	C	A	BPTF	Missense	p.P1106Q	10	100	10.0%	.	.	.
GC13	17	65942244	C	A	BPTF	Missense	p.Q2474K	8	107	7.5%	.	.	.
GC13	17	65989060	C	A	C17orf58	Missense	p.W68L	6	72	8.3%	.	.	.
GC13	17	66274455	G	T	SLC16A6	Missense	p.Q3K	6	61	9.8%	.	.	.
GC13	17	66430768	C	A	WIPI1	Splicing	c.376-1G>T	7	91	7.7%	.	.	.
GC13	17	66871534	C	A	ABCA8	Missense	p.G1460C	8	106	7.5%	.	.	.
GC13	17	66883632	G	T	ABCA8	Missense	p.Q1014K	8	91	8.8%	.	.	.
GC13	17	66898940	C	A	ABCA8	Missense	p.W853L	6	74	8.1%	.	.	.
GC13	17	66928550	G	T	ABCA8	Missense	p.L226I	8	95	8.4%	.	.	.
GC13	17	66987031	G	T	ABCA9	Missense	p.Q1262K	8	110	7.3%	.	O	.
GC13	17	67109785	C	A	ABCA6	Missense	p.G626W	9	122	7.4%	.	.	.
GC13	17	67144984	G	T	ABCA10	Missense	p.P1539Q	7	89	7.9%	.	.	.
GC13	17	67181704	C	A	ABCA10	Missense	p.W804L	9	97	9.3%	.	.	.
GC13	17	67246634	G	T	ABCA5	Missense	p.P1555Q	8	100	8.0%	.	.	.
GC13	17	67256510	G	T	ABCA5	Missense	p.P1203Q	6	72	8.3%	.	.	.
GC13	17	70120491	G	T	SOX9	Missense	p.W498L	6	68	8.8%	.	.	.
GC13	17	71380095	C	A	SDK2	Missense	p.R1542L	5	46	10.9%	.	.	.
GC13	17	71397335	C	A	SDK2	Missense	p.W932L	5	34	14.7%	.	.	.
GC13	17	72356293	C	A	BTBD17	Missense	p.Q59H	7	52	13.5%	.	.	.
GC13	17	72745199	C	A	SLC9A3R1	Missense	p.H72N	6	44	13.6%	.	.	.
GC13	17	72916656	C	A	USH1G	Missense	p.W92L	8	93	8.6%	.	.	.
GC13	17	73102026	G	T	SLC16A5	Missense	p.W472C	8	75	10.7%	.	.	.

GC13	17	73560455	G	T	LLGL2	Missense	p.Q301H	6	49	12.2%	.	.	.
GC13	17	73750807	G	T	ITGB4	Missense	p.R1420L	7	80	8.8%	.	.	.
GC13	17	73775204	G	T	H3F3B	Missense	p.R18S	8	96	8.3%	O	.	.
GC13	17	73839105	C	A	UNC13D	Missense	p.R104M	5	34	14.7%	.	.	.
GC13	17	74084583	C	A	EXOC7	Missense	p.G380C	5	40	12.5%	.	.	.
GC13	17	74163789	C	A	RNF157	Missense	p.R129L	6	70	8.6%	.	.	.
GC13	17	74262032	-	CCACCA	UBALD2	Nonframeshift	p.S55delinsSPP	22	69	31.9%	.	.	.
GC13	17	74287775	C	A	QRICH2	Missense	p.M845I	8	109	7.3%	.	.	.
GC13	17	74395935	C	A	UBE2O	Missense	p.R408L	8	89	9.0%	.	.	.
GC13	17	74622154	C	A	ST6GALNAC1	Missense	p.R480L	6	84	7.1%	.	.	.
GC13	17	74737055	G	T	MFSD11	Nonsense	p.G57X	7	52	13.5%	.	.	.
GC13	17	74737133	G	T	MFSD11	Missense	p.G83C	7	100	7.0%	.	.	.
GC13	17	74738337	G	T	MFSD11	Missense	p.W140L	7	92	7.6%	.	.	.
GC13	17	75209402	C	A	SEC14L1	Missense	p.H590N	7	75	9.3%	.	.	.
GC13	17	76046465	C	A	TNRC6C	Missense	p.P441Q	7	91	7.7%	.	.	.
GC13	17	76121049	G	T	TMC6	Missense	p.P185Q	6	34	17.6%	.	.	.
GC13	17	76167021	G	T	SYNGR2	Missense	p.G75W	7	102	6.9%	.	.	.
GC13	17	76201202	G	T	AFMID	Missense	p.G166W	5	51	9.8%	.	.	.
GC13	17	76547628	G	T	DNAH17	Missense	p.Q794K	12	88	13.6%	.	.	.
GC13	17	76817074	C	A	USP36	Missense	p.R276L	6	94	6.4%	.	O	.
GC13	17	76887822	G	T	CEP295NL	Missense	p.P255Q	9	132	6.8%	.	.	.
GC13	17	76888095	G	T	CEP295NL	Missense	p.P164Q	6	87	6.9%	.	.	.
GC13	17	76991201	G	T	CANT1	Missense	p.P245Q	5	38	13.2%	.	.	.
GC13	17	77809031	C	A	CBX4	Missense	p.R137L	9	109	8.3%	.	.	.
GC13	17	77915957	C	A	TBC1D16	Missense	p.G278W	5	50	10.0%	.	.	.
GC13	17	78013861	C	A	CCDC40	Missense	p.P115Q	9	70	12.9%	.	.	.
GC13	17	78086394	G	T	GAA	Missense	p.R591L	5	29	17.2%	.	.	.
GC13	17	78185914	G	T	SGSH	Missense	p.P302Q	7	72	9.7%	.	.	.
GC13	17	78247136	C	A	RNF213	Missense	p.P65Q	6	46	13.0%	.	.	.
GC13	17	78306186	C	A	RNF213	Missense	p.L1300I	8	120	6.7%	.	.	.
GC13	17	79203150	C	A	TEPSIN	Missense	p.G386W	5	41	12.2%	.	.	.

GC13	17	79496064	G	T	FSCN2	Missense	p.W169C	6	39	15.4%	.	.	.
GC13	17	79534506	C	A	NPLOC4	Missense	p.L501F	8	59	13.6%	.	.	.
GC13	17	79857117	G	T	ANAPC11	Splicing	c.110-1G>T	8	73	11.0%	.	.	.
GC13	17	79860559	G	T	NPB	Missense	p.G105W	5	43	11.6%	.	.	.
GC13	17	80043130	C	A	FASN	Missense	p.W1424L	7	56	12.5%	.	.	.
GC13	17	80130634	G	T	CCDC57	Missense	p.Q537K	6	63	9.5%	.	.	.
GC13	17	80525959	G	T	FOXK2	Missense	p.R215L	6	60	10.0%	.	.	.
GC13	17	80708458	G	T	FN3K	Missense	p.G253W	6	74	8.1%	.	.	.
GC13	17	80790311	C	A	ZNF750	Missense	p.R7L	7	83	8.4%	.	.	.
GC13	17	80992946	G	T	B3GNTL1	Missense	p.Q103K	7	93	7.5%	.	.	.
GC13	18	163365	C	A	USP14	Missense	p.P25H	7	93	7.5%	.	.	.
GC13	18	196724	C	A	USP14	Missense	p.P149Q	8	133	6.0%	.	O	.
GC13	18	223473	G	T	THOC1	Missense	p.P446H	8	73	11.0%	.	.	.
GC13	18	260270	C	A	THOC1	Missense	p.L97F	9	116	7.8%	.	.	.
GC13	18	657895	G	T	TYMS	Missense	p.P118Q	4	29	13.8%	.	.	.
GC13	18	2585164	G	T	NDC80	Missense	p.W211L	8	94	8.5%	.	.	.
GC13	18	2666886	G	T	SMCHD1	Nonsense	p.G94X	7	94	7.4%	.	.	.
GC13	18	2740724	G	T	SMCHD1	Nonsense	p.G1180X	6	74	8.1%	.	.	.
GC13	18	2891845	C	A	EMILIN2	Missense	p.R574S	6	86	7.0%	.	.	.
GC13	18	2934355	G	T	LPIN2	Missense	p.P421H	7	94	7.4%	.	.	.
GC13	18	3126892	G	T	MYOM1	Missense	p.P837Q	7	88	8.0%	.	.	.
GC13	18	3456507	C	A	TGIF1	Missense	p.R61S	8	118	6.8%	.	.	.
GC13	18	3457453	C	A	TGIF1	Missense	p.R115S	7	83	8.4%	.	.	.
GC13	18	3457766	C	A	TGIF1	Missense	p.P219Q	10	127	7.9%	.	.	.
GC13	18	5423510	C	A	EPB41L3	Missense	p.L402F	8	100	8.0%	.	.	.
GC13	18	5891894	G	T	TMEM200C	Missense	p.L57I	8	82	9.8%	.	.	.
GC13	18	6982565	C	A	LAMA1	Missense	p.G1941W	6	70	8.6%	.	.	.
GC13	18	7002327	C	A	LAMA1	Missense	p.G1440W	6	48	12.5%	.	O	.
GC13	18	7008557	G	T	LAMA1	Missense	p.P1351Q	7	84	8.3%	.	.	.
GC13	18	7774243	C	A	PTPRM	Missense	p.P57Q	7	124	5.6%	.	.	.
GC13	18	8069855	G	T	PTPRM	Missense	p.W435L	6	78	7.7%	.	.	.

GC13	18	8777837	C	A	MTCL1	Missense	p.L122I	7	64	10.9%	.	.	.
GC13	18	9256381	T	A	ANKRD12	Missense	p.I1016K	5	93	5.4%	.	.	.
GC13	18	9257950	C	A	ANKRD12	Missense	p.P1539Q	8	107	7.5%	.	.	.
GC13	18	9258460	C	A	ANKRD12	Missense	p.P1709H	7	90	7.8%	.	.	.
GC13	18	10748485	G	T	PIEZO2	Missense	p.Q1445K	5	43	11.6%	.	.	.
GC13	18	10787073	G	T	PIEZO2	Missense	p.P760Q	8	78	10.3%	.	.	.
GC13	18	12262839	G	T	CIDEA	Missense	p.M18I	8	92	8.7%	.	.	.
GC13	18	12506573	C	A	SPIRE1	Missense	p.R292L	6	63	9.5%	.	.	.
GC13	18	12674535	C	A	CEP76	Missense	p.R539L	6	76	7.9%	.	.	.
GC13	18	12720580	G	T	PSMG2	Missense	p.W160L	7	95	7.4%	.	.	.
GC13	18	12999487	G	T	CEP192	Missense	p.G22C	9	99	9.1%	.	.	.
GC13	18	13040940	C	A	CEP192	Missense	p.H641N	8	121	6.6%	.	.	.
GC13	18	13056080	C	A	CEP192	Missense	p.P1164Q	6	91	6.6%	.	.	.
GC13	18	14105880	G	T	ZNF519	Missense	p.P220Q	6	70	8.6%	.	.	.
GC13	18	18608803	G	T	ROCK1	Missense	p.P382H	10	121	8.3%	.	.	.
GC13	18	19021402	G	T	GREB1L	Nonsense	p.G371X	9	137	6.6%	.	.	.
GC13	18	19153975	G	T	ESCO1	Missense	p.P277Q	9	102	8.8%	.	.	.
GC13	18	19763002	G	T	GATA6	Missense	p.G540W	10	101	9.9%	.	.	.
GC13	18	20572878	C	A	RBBP8	Missense	p.P363H	8	120	6.7%	.	.	.
GC13	18	21107839	C	A	C18orf8	Missense	p.L267M	5	37	13.5%	.	.	.
GC13	18	21119384	C	A	NPC1	Missense	p.W949L	6	64	9.4%	.	.	.
GC13	18	21131617	G	T	NPC1	Missense	p.P543Q	7	82	8.5%	.	.	.
GC13	18	21214077	C	A	ANKRD29	Missense	p.G123W	5	68	7.4%	.	.	.
GC13	18	21218881	G	T	ANKRD29	Missense	p.Q88K	6	56	10.7%	.	.	.
GC13	18	21329484	C	A	LAMA3	Missense	p.R220S	7	86	8.1%	.	.	.
GC13	18	21438711	C	A	LAMA3	Missense	p.P1447Q	7	105	6.7%	.	.	.
GC13	18	21494744	G	T	LAMA3	Missense	p.G857C	8	92	8.7%	.	.	.
GC13	18	21912936	C	A	OSBPL1A	Nonsense	p.G199X	6	77	7.8%	.	.	.
GC13	18	22806234	C	A	ZNF521	Missense	p.G330W	8	92	8.7%	.	.	.
GC13	18	23738188	G	T	PSMA8	Missense	p.G153C	7	98	7.1%	.	.	.
GC13	18	23866347	G	T	TAF4B	Missense	p.G492W	8	109	7.3%	.	.	.

GC13	18	24496801	C	A	CHST9	Missense	p.G252W	13	127	10.2%	.	.	.
GC13	18	25565726	C	A	CDH2	Splicing	c.1742-1G>T	7	92	7.6%	.	.	.
GC13	18	28935223	C	T	DSG1	Missense	p.H1022Y	21	74	28.4%	.	.	.
GC13	18	29111203	C	A	DSG2	Missense	p.P423Q	7	94	7.4%	.	.	.
GC13	18	29126486	T	C	DSG2	Missense	p.V1046A	25	63	39.7%	.	.	.
GC13	18	29848037	C	A	GAREM1	Nonsense	p.G810X	7	86	8.1%	.	.	.
GC13	18	29867353	G	T	GAREM1	Missense	p.L403I	7	80	8.8%	.	.	.
GC13	18	30260465	G	T	KLHL14	Missense	p.L446I	7	71	9.9%	.	.	.
GC13	18	32834235	G	T	ZNF397	Missense	p.Q35K	9	107	8.4%	.	.	.
GC13	18	34289066	G	T	FHOD3	Missense	p.G557W	7	108	6.5%	.	O	.
GC13	18	34647444	C	A	KIAA1328	Missense	p.Q282K	5	44	11.4%	.	.	.
GC13	18	39593421	C	A	PIK3C3	Missense	p.L333I	7	88	8.0%	.	.	.
GC13	18	39618782	C	A	PIK3C3	Missense	p.P606H	5	38	13.2%	.	.	.
GC13	18	39620658	C	A	PIK3C3	Missense	p.Q623K	10	122	8.2%	.	.	.
GC13	18	42456558	G	T	SETBP1	Missense	p.W190L	5	44	11.4%	O	.	.
GC13	18	42531065	G	T	SETBP1	Missense	p.R587L	11	107	10.3%	O	.	.
GC13	18	43316462	G	T	SLC14A1	Missense	p.W39L	10	99	10.1%	.	.	.
GC13	18	43493739	G	T	EPG5	Missense	p.Q1250K	7	58	12.1%	.	.	.
GC13	18	43534763	G	T	EPG5	Missense	p.P202Q	7	90	7.8%	.	O	.
GC13	18	43671710	G	T	ATP5A1	Missense	p.R33S	9	93	9.7%	.	.	.
GC13	18	44102192	C	A	LOXHD1	Missense	p.G446W	6	74	8.1%	.	.	.
GC13	18	45396918	C	A	SMAD2	Missense	p.W85L	6	84	7.1%	O	.	.
GC13	18	45566856	G	T	ZBTB7C	Missense	p.P208H	7	49	14.3%	.	.	.
GC13	18	46284448	G	T	CTIF	Missense	p.R248L	6	64	9.4%	.	.	.
GC13	18	46448031	C	A	SMAD7	Missense	p.W143L	6	79	7.6%	.	.	.
GC13	18	46798622	G	T	DYM	Missense	p.H393N	6	69	8.7%	.	.	.
GC13	18	47108778	G	T	LIPG	Missense	p.M287I	8	77	10.4%	.	.	.
GC13	18	47405449	G	T	MYO5B	Missense	p.Q1048K	11	119	9.2%	.	.	.
GC13	18	47799078	G	T	MBD1	Missense	p.R481S	6	70	8.6%	.	.	.
GC13	18	47799772	G	T	MBD1	Missense	p.Q427K	7	88	8.0%	.	.	.
GC13	18	48510501	G	T	ELAC1	Nonsense	p.G65X	8	113	7.1%	.	.	.

GC13	18	48510525	G	T	ELAC1	Missense	p.G73W	8	104	7.7%	.	.	.
GC13	18	48584797	C	A	SMAD4	Missense	p.P292Q	7	114	6.1%	O	.	.
GC13	18	50451625	G	T	DCC	Missense	p.L290F	8	103	7.8%	.	.	.
GC13	18	50731655	G	T	DCC	Missense	p.W548L	6	74	8.1%	.	.	.
GC13	18	51851099	G	T	STARD6	Missense	p.P209Q	9	125	7.2%	.	.	.
GC13	18	52262266	G	T	DYNAP	Missense	p.G78W	8	76	10.5%	.	.	.
GC13	18	52556460	C	A	RAB27B	Missense	p.P158Q	7	88	8.0%	.	.	.
GC13	18	53303039	C	A	TCF4	Missense	p.E30D	7	82	8.5%	.	.	.
GC13	18	55104078	C	A	ONECUT2	Missense	p.P377Q	6	79	7.6%	.	.	.
GC13	18	55273849	G	T	NARS	Missense	p.P379Q	6	97	6.2%	.	.	.
GC13	18	55352342	G	T	ATP8B1	Missense	p.H479N	7	81	8.6%	.	.	.
GC13	18	56033319	C	A	NEDD4L	Missense	p.P500Q	8	119	6.7%	.	.	.
GC13	18	56203445	C	A	ALPK2	Missense	p.W1325L	7	73	9.6%	.	.	.
GC13	18	56940360	C	A	RAX	Missense	p.G27W	4	38	10.5%	.	.	.
GC13	18	58039561	C	A	MC4R	Missense	p.G8W	7	76	9.2%	.	.	.
GC13	18	59942648	G	T	KIAA1468	Missense	p.W970L	8	111	7.2%	.	.	.
GC13	18	60036249	G	T	TNFRSF11A	Nonsense	p.G353X	7	86	8.1%	.	.	.
GC13	18	61064379	C	A	VPS4B	Missense	p.R327L	8	127	6.3%	.	.	.
GC13	18	63547960	C	A	CDH7	Missense	p.L730M	7	81	8.6%	.	O	.
GC13	18	64172450	G	T	CDH19	Missense	p.Q640K	8	131	6.1%	.	O	.
GC13	18	65181439	G	T	DSEL	Missense	p.P146Q	7	100	7.0%	.	.	.
GC13	18	66354946	G	T	TMX3	Missense	p.L91I	10	89	11.2%	.	.	.
GC13	18	66504466	C	A	CCDC102B	Missense	p.Q156K	9	97	9.3%	.	.	.
GC13	18	67672481	G	T	RTTN	Missense	p.P1271Q	8	89	9.0%	.	.	.
GC13	18	67718691	G	T	RTTN	Missense	p.P848Q	6	85	7.1%	.	.	.
GC13	18	67759941	G	T	RTTN	Missense	p.H423N	8	88	9.1%	.	.	.
GC13	18	67872416	G	T	RTTN	Missense	p.P56Q	9	128	7.0%	.	.	.
GC13	18	67993292	G	T	SOCS6	Missense	p.R463M	8	95	8.4%	.	.	.
GC13	18	71740752	G	T	FBXO15	Missense	p.L493M	8	119	6.7%	.	.	.
GC13	18	72103926	C	A	FAM69C	Missense	p.W357L	5	37	13.5%	.	.	.
GC13	18	72109305	C	A	FAM69C	Missense	p.R308M	9	87	10.3%	.	.	.

GC13	18	72343358	C	A	ZNF407	Missense	p.P128Q	7	88	8.0%	.	.	.
GC13	18	74091420	C	A	ZNF516	Missense	p.G884W	7	50	14.0%	.	.	.
GC13	18	76757027	G	T	SALL3	Missense	p.R1203L	6	67	9.0%	.	.	.
GC13	18	76757194	G	T	SALL3	Missense	p.G1259W	7	65	10.8%	.	.	.
GC13	18	77896313	C	A	ADNP2	Missense	p.P1006Q	7	83	8.4%	.	.	.
GC13	18	77918234	G	T	PARD6G	Missense	p.P184Q	5	40	12.5%	.	.	.
GC13	19	282778	C	A	PLPP2	Missense	p.G172W	6	56	10.7%	.	.	.
GC13	19	805022	G	T	PTBP1	Missense	p.G243W	5	57	8.8%	.	.	.
GC13	19	814733	G	T	PLPPR3	Missense	p.Q206K	6	47	12.8%	.	.	.
GC13	19	1047291	G	T	ABCA7	Missense	p.G661C	6	74	8.1%	.	.	.
GC13	19	1066155	C	A	ARHGAP45	Missense	p.P44Q	8	53	15.1%	.	.	.
GC13	19	1068674	C	A	ARHGAP45	Missense	p.H134N	9	63	14.3%	.	.	.
GC13	19	1109182	C	A	SBNO2	Missense	p.W1069L	5	43	11.6%	.	.	.
GC13	19	1817250	G	T	REXO1	Missense	p.H1057N	6	40	15.0%	.	.	.
GC13	19	1828036	C	A	REXO1	Missense	p.R251L	5	38	13.2%	.	.	.
GC13	19	2185874	C	A	DOT1L	Missense	p.P49Q	7	109	6.4%	.	.	.
GC13	19	2408530	G	T	TMPRSS9	Missense	p.R306L	7	96	7.3%	.	.	.
GC13	19	2416612	G	T	TMPRSS9	Missense	p.G574W	5	48	10.4%	.	.	.
GC13	19	2427271	G	T	TIMM13	Missense	p.L58M	7	77	9.1%	.	.	.
GC13	19	2438223	G	T	LMNB2	Missense	p.L208M	7	98	7.1%	.	.	.
GC13	19	2477072	G	T	GADD45B	Missense	p.E64D	7	95	7.4%	.	.	.
GC13	19	2827738	C	A	ZNF554	Missense	p.L84M	5	26	19.2%	.	O	.
GC13	19	2853267	G	T	ZNF555	Missense	p.G401W	7	78	9.0%	.	.	.
GC13	19	2933569	G	T	ZNF77	Missense	p.P519Q	8	105	7.6%	.	.	.
GC13	19	2933606	G	T	ZNF77	Missense	p.L507I	7	98	7.1%	.	.	.
GC13	19	2934098	C	A	ZNF77	Missense	p.G343W	9	109	8.3%	.	.	.
GC13	19	2939366	G	T	ZNF77	Missense	p.P15T	7	74	9.5%	.	.	.
GC13	19	2991912	C	A	TLE6	Missense	p.P316Q	6	50	12.0%	.	.	.
GC13	19	3533332	G	T	FZR1	Missense	p.W339L	5	39	12.8%	.	.	.
GC13	19	3750591	G	T	TJP3	Missense	p.R890L	6	90	6.7%	.	.	.
GC13	19	3964846	C	A	DAPK3	Missense	p.R69L	7	85	8.2%	.	.	.

GC13	19	4024037	C	A	PIAS4	Missense	p.P153Q	7	94	7.4%	.	.	.
GC13	19	4102439	G	T	MAP2K2	Missense	p.L155M	6	56	10.7%	O	.	.
GC13	19	4216784	C	A	ANKRD24	Missense	p.H543N	5	39	12.8%	.	.	.
GC13	19	4511361	C	A	PLIN4	Missense	p.G857C	6	80	7.5%	.	.	.
GC13	19	4512582	C	A	PLIN4	Missense	p.G450C	7	93	7.5%	.	.	.
GC13	19	4704217	C	A	DPP9	Nonsense	p.E176X	6	84	7.1%	.	.	.
GC13	19	5211707	G	T	PTPRS	Missense	p.R1263S	6	85	7.1%	.	.	.
GC13	19	5456170	G	T	ZNRF4	Missense	p.R223L	6	63	9.5%	.	.	.
GC13	19	5616210	A	G	SAFB2	Missense	p.F159S	4	84	4.8%	.	.	.
GC13	19	5787373	C	A	DUS3L	Splicing	c.1213-1G>T	7	47	14.9%	.	.	.
GC13	19	5914970	G	T	CAPS	Missense	p.R180L	6	50	12.0%	.	.	.
GC13	19	6380585	C	A	GTF2F1	Missense	p.G450C	6	61	9.8%	.	.	.
GC13	19	6734209	G	T	GPR108	Missense	p.R162S	6	57	10.5%	.	.	.
GC13	19	6743040	G	T	TRIP10	Missense	p.R87L	6	91	6.6%	.	.	.
GC13	19	7083492	G	T	ZNF557	Nonsense	p.G344X	6	58	10.3%	.	.	.
GC13	19	7505169	G	T	ARHGEF18	Missense	p.G115W	6	95	6.3%	.	.	.
GC13	19	7535080	G	T	ARHGEF18	Missense	p.G1140W	6	65	9.2%	.	.	.
GC13	19	7584289	C	A	ZNF358	Missense	p.P54Q	7	80	8.8%	.	.	.
GC13	19	7685450	G	T	XAB2	Missense	p.Q693K	6	62	9.7%	.	.	.
GC13	19	7809954	C	A	CD209	Missense	p.W122L	8	83	9.6%	.	.	.
GC13	19	7963814	G	T	LRRC8E	Missense	p.W7L	8	80	10.0%	.	.	.
GC13	19	8130952	G	T	FBN3	Missense	p.L2761M	7	80	8.8%	.	.	.
GC13	19	8369999	G	T	CD320	Missense	p.R62S	5	47	10.6%	.	.	.
GC13	19	8563869	G	T	PRAM1	Missense	p.Q275K	7	84	8.3%	.	.	.
GC13	19	8657693	G	T	ADAMTS10	Missense	p.P514Q	5	47	10.6%	.	.	.
GC13	19	9013905	C	A	MUC16	Missense	p.G12829W	8	68	11.8%	.	.	.
GC13	19	9046562	C	A	MUC16	Missense	p.W11690L	7	86	8.1%	.	.	.
GC13	19	9047652	C	A	MUC16	Missense	p.G11327W	8	99	8.1%	.	.	.
GC13	19	9047826	G	T	MUC16	Missense	p.H11269N	6	86	7.0%	.	.	.
GC13	19	9057046	C	A	MUC16	Missense	p.G10134C	8	100	8.0%	.	.	.
GC13	19	9059535	T	G	MUC16	Missense	p.E9304A	36	110	32.7%	.	.	.

GC13	19	9059581	C	A	MUC16	Missense	p.G9289W	8	113	7.1%	.	.	.
GC13	19	9060186	C	A	MUC16	Missense	p.G9087V	7	78	9.0%	.	.	.
GC13	19	9062282	C	A	MUC16	Missense	p.M8388I	8	78	10.3%	.	.	.
GC13	19	9062772	C	A	MUC16	Missense	p.W8225L	7	88	8.0%	.	.	.
GC13	19	9064741	C	A	MUC16	Missense	p.G7569C	8	107	7.5%	.	.	.
GC13	19	9066514	C	A	MUC16	Missense	p.G6978C	9	106	8.5%	.	.	.
GC13	19	9067164	C	A	MUC16	Missense	p.R6761M	7	86	8.1%	.	O	.
GC13	19	9068356	G	T	MUC16	Missense	p.L6364M	9	116	7.8%	.	.	.
GC13	19	9073722	C	A	MUC16	Missense	p.G4575V	7	81	8.6%	.	.	.
GC13	19	9087227	C	A	MUC16	Missense	p.G1530W	9	105	8.6%	.	.	.
GC13	19	9088985	C	A	MUC16	Missense	p.G944W	10	85	11.8%	.	O	.
GC13	19	9090314	C	A	MUC16	Missense	p.G501W	8	89	9.0%	.	.	.
GC13	19	9296660	G	T	OR7D2	Missense	p.W68L	7	88	8.0%	.	O	.
GC13	19	9297151	G	T	OR7D2	Missense	p.G232W	7	84	8.3%	.	.	.
GC13	19	9407395	C	A	ZNF699	Missense	p.G229W	7	81	8.6%	.	.	.
GC13	19	9491818	C	A	ZNF177	Missense	p.L271I	7	56	12.5%	.	.	.
GC13	19	9581155	G	T	ZNF560	Missense	p.Q15K	6	69	8.7%	.	.	.
GC13	19	9639193	C	A	ZNF426	Missense	p.G472W	6	60	10.0%	.	.	.
GC13	19	9727808	G	T	ZNF561	Missense	p.P52T	7	77	9.1%	.	.	.
GC13	19	9868837	C	A	ZNF846	Nonsense	p.G306X	7	85	8.2%	.	.	.
GC13	19	9869373	G	T	ZNF846	Missense	p.P127Q	8	126	6.3%	.	.	.
GC13	19	10132044	G	T	RDH8	Missense	p.R237L	5	57	8.8%	.	.	.
GC13	19	10225209	G	T	P2RY11	Missense	p.R307L	6	75	8.0%	.	.	.
GC13	19	10254460	C	A	DNMT1	Missense	p.R1017L	8	105	7.6%	.	.	.
GC13	19	10291143	C	A	DNMT1	Missense	p.G110W	7	99	7.1%	.	.	.
GC13	19	10398732	G	T	ICAM4	Missense	p.G231W	8	84	9.5%	.	O	.
GC13	19	10403411	C	A	ICAM5	Missense	p.P362Q	6	88	6.8%	.	.	.
GC13	19	10426068	G	T	FDX1L	Missense	p.L104M	9	84	10.7%	.	.	.
GC13	19	10599886	C	A	KEAP1	Missense	p.G564W	7	95	7.4%	O	.	.
GC13	19	10781852	C	A	ILF3	Missense	p.P69Q	7	81	8.6%	.	.	.
GC13	19	11031171	G	T	CARM1	Missense	p.R419L	4	38	10.5%	.	.	.

GC13	19	11031607	G	T	CARM1	Missense	p.G508W	6	65	9.2%	.	O	.
GC13	19	11034191	G	T	YIPF2	Missense	p.L233I	7	81	8.6%	.	.	.
GC13	19	11114030	C	A	SMARCA4	Missense	p.P653Q	6	83	7.2%	O	.	.
GC13	19	11354335	G	T	DOCK6	Missense	p.R386S	6	70	8.6%	.	.	.
GC13	19	11548772	G	T	PRKCSH	Missense	p.R91L	8	112	7.1%	.	.	.
GC13	19	11597919	G	T	ZNF653	Missense	p.P409Q	6	87	6.9%	.	.	.
GC13	19	11891828	G	T	ZNF441	Missense	p.G397W	7	52	13.5%	.	O	.
GC13	19	12127420	G	T	ZNF433	Missense	p.Q88K	9	130	6.9%	.	.	.
GC13	19	12223383	G	T	ZNF788	Missense	p.G341W	8	118	6.8%	.	.	.
GC13	19	12463889	G	T	ZNF442	Missense	p.Q40K	6	62	9.7%	.	.	.
GC13	19	12691424	G	T	ZNF490	Missense	p.L489M	8	107	7.5%	.	.	.
GC13	19	12691571	G	T	ZNF490	Missense	p.H440N	9	93	9.7%	.	.	.
GC13	19	12774537	G	T	MAN2B1	Missense	p.P248Q	5	30	16.7%	.	.	.
GC13	19	12807082	C	A	FBXW9	Missense	p.R105L	6	43	14.0%	.	.	.
GC13	19	12874385	C	A	HOOK2	Missense	p.R654L	9	107	8.4%	.	.	.
GC13	19	12918276	G	T	RNASEH2A	Missense	p.G123W	6	81	7.4%	.	.	.
GC13	19	12945599	C	A	RTBDN	Missense	p.E26D	7	49	14.3%	.	.	.
GC13	19	12979952	G	T	MAST1	Missense	p.R949L	7	102	6.9%	.	.	.
GC13	19	12984846	C	A	MAST1	Missense	p.P1292Q	5	60	8.3%	.	O	.
GC13	19	13051220	G	T	CALR	Missense	p.W219L	9	96	9.4%	O	.	.
GC13	19	13051443	C	A	CALR	Missense	p.P264Q	7	86	8.1%	O	.	.
GC13	19	13227167	C	A	TRMT1	Missense	p.R16L	7	77	9.1%	.	.	.
GC13	19	13246188	G	T	NACC1	Missense	p.R56L	7	85	8.2%	.	.	.
GC13	19	13260664	C	A	STX10	Missense	p.W28L	5	30	16.7%	.	.	.
GC13	19	13941743	G	T	ZSWIM4	Missense	p.R950L	6	79	7.6%	.	.	.
GC13	19	13991270	G	T	NANOS3	Missense	p.G178W	7	60	11.7%	.	.	.
GC13	19	14000350	G	T	C19orf57	Missense	p.P322Q	6	85	7.1%	.	.	.
GC13	19	14037685	G	T	CC2D1A	Nonsense	p.E709X	7	70	10.0%	.	.	.
GC13	19	14153551	G	T	IL27RA	Missense	p.G240W	7	100	7.0%	.	.	.
GC13	19	14165312	G	T	PALM3	Missense	p.P376Q	7	92	7.6%	.	.	.
GC13	19	14165800	T	G	PALM3	Missense	p.K213N	24	76	31.6%	.	.	.

GC13	19	14194390	G	T	C19orf67	Missense	p.L170M	7	91	7.7%	.	.	.
GC13	19	14232369	G	T	ASF1B	Missense	p.P126Q	6	59	10.2%	.	.	.
GC13	19	14262009	C	A	ADGRL1	Missense	p.E1362D	7	43	16.3%	.	.	.
GC13	19	14267834	G	T	ADGRL1	Missense	p.R957S	6	57	10.5%	.	.	.
GC13	19	14273766	C	A	ADGRL1	Missense	p.G283W	5	53	9.4%	.	.	.
GC13	19	14804262	G	T	ZNF333	Splicing	.	7	56	12.5%	.	.	.
GC13	19	14910487	C	A	OR7C1	Missense	p.M154I	8	103	7.8%	.	.	.
GC13	19	14938933	C	A	OR7A5	Missense	p.G41W	5	50	10.0%	.	.	.
GC13	19	15198571	G	T	OR1I1	Missense	p.R232L	7	108	6.5%	.	.	.
GC13	19	15290250	G	T	NOTCH3	Missense	p.Q1129K	7	70	10.0%	.	.	.
GC13	19	15383860	C	A	BRD4	Missense	p.M17I	8	83	9.6%	.	.	.
GC13	19	15752380	C	A	CYP4F3	Missense	p.P52Q	7	84	8.3%	.	.	.
GC13	19	16003202	G	T	CYP4F2	Missense	p.R148S	5	32	15.6%	.	.	.
GC13	19	16338993	C	A	AP1M1	Missense	p.H288N	8	103	7.8%	.	.	.
GC13	19	16860832	C	A	NWD1	Missense	p.P254Q	6	100	6.0%	.	.	.
GC13	19	17283575	G	T	MYO9B	Missense	p.R648L	7	60	11.7%	.	.	.
GC13	19	17339096	G	T	OCEL1	Missense	p.R217M	7	89	7.9%	.	.	.
GC13	19	17370187	C	A	USHBP1	Missense	p.Q255H	7	88	8.0%	.	O	.
GC13	19	17394502	C	A	ANKLE1	Missense	p.P353Q	7	89	7.9%	.	.	.
GC13	19	17438281	G	T	ANO8	Missense	p.P839Q	6	54	11.1%	.	.	.
GC13	19	17451908	C	T	GTPBP3	Missense	p.P376S	18	56	32.1%	.	.	.
GC13	19	17955151	G	T	JAK3	Missense	p.L26M	5	37	13.5%	O	.	.
GC13	19	18234043	G	T	MAST3	Missense	p.R110L	7	81	8.6%	.	.	.
GC13	19	18368664	G	T	KIAA1683	Missense	p.Q1144K	6	74	8.1%	.	.	.
GC13	19	18650363	G	T	FKBP8	Missense	p.Q154K	6	58	10.3%	.	.	.
GC13	19	18971146	G	T	UPF1	Missense	p.K744N	8	76	10.5%	.	.	.
GC13	19	18995049	C	A	CERS1	Missense	p.R48L	4	33	12.1%	.	.	.
GC13	19	19232118	G	T	TMEM161A	Missense	p.L202M	7	68	10.3%	.	.	.
GC13	19	19232400	G	T	TMEM161A	Missense	p.P142Q	6	51	11.8%	.	.	.
GC13	19	19338863	C	A	NCAN	Missense	p.P812T	7	60	11.7%	.	.	.
GC13	19	19379467	C	A	TM6SF2	Missense	p.R194L	6	92	6.5%	.	.	.

GC13	19	19763429	C	A	ATP13A1	Missense	p.R734L	6	59	10.2%	.	.	.
GC13	19	19767656	C	A	ATP13A1	Missense	p.G326C	5	48	10.4%	.	.	.
GC13	19	19790219	C	A	ZNF101	Missense	p.R21S	8	88	9.1%	.	.	.
GC13	19	21281645	C	A	ZNF714	Missense	p.P24Q	6	91	6.6%	.	.	.
GC13	19	24289443	C	A	ZNF254	Missense	p.P11Q	6	51	11.8%	.	.	.
GC13	19	31040183	G	T	ZNF536	Missense	p.Q1219H	7	63	11.1%	.	.	.
GC13	19	31040284	G	T	ZNF536	Missense	p.R1253L	7	73	9.6%	.	.	.
GC13	19	31767688	C	A	TSHZ3	Missense	p.R1004L	7	85	8.2%	.	.	.
GC13	19	31767881	C	A	TSHZ3	Missense	p.G940W	6	72	8.3%	.	.	.
GC13	19	33324150	C	A	SLC7A9	Missense	p.W435L	9	64	14.1%	.	.	.
GC13	19	33602687	G	T	GPATCH1	Missense	p.R548L	5	23	21.7%	.	O	.
GC13	19	33623179	G	T	WDR88	Missense	p.W35L	7	49	14.3%	.	.	.
GC13	19	33703477	C	A	SLC7A10	Missense	p.G193W	5	52	9.6%	.	.	.
GC13	19	34263583	G	T	CHST8	Missense	p.R297L	7	75	9.3%	.	.	.
GC13	19	34856188	G	T	GPI	Missense	p.R6L	5	33	15.2%	.	.	.
GC13	19	35258236	G	T	ZNF599	Missense	p.Q76K	5	27	18.5%	.	.	.
GC13	19	35715765	G	T	FAM187B	Missense	p.P358Q	5	71	7.0%	.	.	.
GC13	19	35719088	G	T	FAM187B	Missense	p.R166S	8	86	9.3%	.	.	.
GC13	19	35940705	G	T	FFAR2	Missense	p.R30L	6	97	6.2%	.	.	.
GC13	19	36018781	G	T	SBSN	Missense	p.H135N	8	99	8.1%	.	.	.
GC13	19	36018889	G	T	SBSN	Missense	p.H99N	7	97	7.2%	.	.	.
GC13	19	36120132	C	A	RBM42	Missense	p.P26Q	5	41	12.2%	.	.	.
GC13	19	36149538	G	T	COX6B1	Missense	p.G84W	4	32	12.5%	.	.	.
GC13	19	36271839	G	T	ARHGAP33	Missense	p.G147W	12	94	12.8%	.	.	.
GC13	19	36273408	G	T	ARHGAP33	Missense	p.G271W	7	68	10.3%	.	.	.
GC13	19	36279108	G	T	ARHGAP33	Missense	p.W1050L	5	48	10.4%	.	.	.
GC13	19	36330464	G	T	NPHS1	Missense	p.P954Q	7	68	10.3%	.	.	.
GC13	19	36349612	C	A	KIRREL2	Missense	p.P73Q	6	50	12.0%	.	.	.
GC13	19	36602295	C	A	OVOL3	Missense	p.P40Q	5	51	9.8%	.	.	.
GC13	19	36673847	C	A	ZNF565	Nonsense	p.G341X	6	56	10.7%	.	.	.
GC13	19	36674351	C	A	ZNF565	Missense	p.G173W	7	69	10.1%	.	.	.

GC13	19	36728162	G	T	ZNF146	Missense	p.G274W	7	88	8.0%	.	.	.
GC13	19	37005960	C	A	ZNF260	Missense	p.G61C	8	133	6.0%	.	.	.
GC13	19	37117844	G	T	ZNF382	Missense	p.G348W	8	95	8.4%	.	.	.
GC13	19	37147352	G	T	ZNF461	Missense	p.P77Q	5	39	12.8%	.	.	.
GC13	19	37210336	C	A	ZNF567	Missense	p.P206Q	8	91	8.8%	.	.	.
GC13	19	37239487	C	A	ZNF850	Missense	p.G787W	8	91	8.8%	.	.	.
GC13	19	37239655	C	A	ZNF850	Missense	p.G731W	5	39	12.8%	.	.	.
GC13	19	37487878	G	T	ZNF568	Missense	p.G365W	6	51	11.8%	.	.	.
GC13	19	37487893	C	A	ZNF568	Missense	p.R370S	6	51	11.8%	.	.	.
GC13	19	37643723	C	A	ZNF585A	Missense	p.G360W	8	89	9.0%	.	.	.
GC13	19	37644380	G	T	ZNF585A	Missense	p.Q141K	9	123	7.3%	.	.	.
GC13	19	37677445	C	A	ZNF585B	Missense	p.G332W	7	97	7.2%	.	.	.
GC13	19	37733880	C	A	ZNF383	Missense	p.H248N	6	69	8.7%	.	.	.
GC13	19	37853250	G	T	HKR1	Missense	p.G125W	9	98	9.2%	.	.	.
GC13	19	37853820	C	A	HKR1	Missense	p.H315N	7	93	7.5%	.	.	.
GC13	19	37854204	G	T	HKR1	Missense	p.G443W	7	82	8.5%	.	.	.
GC13	19	37903588	C	A	ZNF569	Missense	p.G499C	7	93	7.5%	.	.	.
GC13	19	37974849	C	A	ZNF570	Missense	p.Q67K	7	100	7.0%	.	.	.
GC13	19	38028662	G	T	ZNF793	Missense	p.G368W	7	89	7.9%	.	.	.
GC13	19	38103494	C	A	ZNF540	Missense	p.P406Q	6	71	8.5%	.	.	.
GC13	19	38189417	C	A	ZNF607	Missense	p.G538W	7	116	6.0%	.	.	.
GC13	19	38377568	G	T	WDR87	Missense	p.P2248Q	10	162	6.2%	.	.	.
GC13	19	38377622	G	T	WDR87	Missense	p.P2230Q	8	136	5.9%	.	.	.
GC13	19	38378652	G	T	WDR87	Missense	p.Q1887K	8	106	7.5%	.	.	.
GC13	19	38379321	G	T	WDR87	Missense	p.Q1664K	9	136	6.6%	.	.	.
GC13	19	38379680	G	T	WDR87	Missense	p.P1544Q	7	144	4.9%	.	.	.
GC13	19	38380343	C	A	WDR87	Missense	p.W1323L	7	58	12.1%	.	.	.
GC13	19	38572812	G	T	SIPA1L3	Missense	p.G203W	5	46	10.9%	.	.	.
GC13	19	38573229	C	A	SIPA1L3	Missense	p.H342N	6	55	10.9%	.	.	.
GC13	19	38633292	G	T	SIPA1L3	Missense	p.G1159W	6	61	9.8%	.	.	.
GC13	19	38655377	G	A	SIPA1L3	Missense	p.G1347S	5	34	14.7%	.	.	.

GC13	19	38901539	C	A	RASGRP4	Missense	p.W462L	5	38	13.2%	.	.	.
GC13	19	38954094	G	T	RYR1	Missense	p.R870L	4	37	10.8%	.	.	.
GC13	19	38996584	C	A	RYR1	Missense	p.Q2847K	6	49	12.2%	.	.	.
GC13	19	39002250	G	T	RYR1	Missense	p.G3058W	8	125	6.4%	.	.	.
GC13	19	39034423	G	T	RYR1	Missense	p.G3969W	6	75	8.0%	.	.	.
GC13	19	39061278	G	T	RYR1	Missense	p.R4559L	8	84	9.5%	.	.	.
GC13	19	39589776	C	A	ACP7	Missense	p.H167N	6	52	11.5%	.	.	.
GC13	19	39591381	G	T	ACP7	Missense	p.W232L	6	53	11.3%	.	.	.
GC13	19	39664433	G	T	PAK4	Missense	p.R141L	5	35	14.3%	.	.	.
GC13	19	39882256	C	A	MED29	Missense	p.P86Q	9	97	9.3%	.	.	.
GC13	19	39950535	G	T	SUPT5H	Missense	p.G183W	8	116	6.9%	.	.	.
GC13	19	39950545	G	T	SUPT5H	Missense	p.R186L	7	108	6.5%	.	.	.
GC13	19	39964664	G	T	SUPT5H	Missense	p.G848W	8	84	9.5%	.	.	.
GC13	19	39971441	C	A	TIMM50	Missense	p.P86Q	4	36	11.1%	.	.	.
GC13	19	40321374	C	A	DYRK1B	Missense	p.R38L	4	30	13.3%	.	.	.
GC13	19	40360876	G	T	FCGBP	Missense	p.H5178N	7	83	8.4%	.	.	.
GC13	19	40480240	G	T	PSMC4	Missense	p.R121L	7	49	14.3%	.	.	.
GC13	19	40520852	G	T	ZNF546	Missense	p.G533W	8	100	8.0%	.	.	.
GC13	19	40521020	G	T	ZNF546	Missense	p.G589W	6	49	12.2%	.	.	.
GC13	19	40590695	G	T	ZNF780A	Missense	p.P5Q	6	77	7.8%	.	.	.
GC13	19	40698507	G	T	MAP3K10	Missense	p.R190L	6	61	9.8%	.	.	.
GC13	19	40745986	C	A	AKT2	Missense	p.R140L	7	111	6.3%	.	.	.
GC13	19	40880517	C	A	PLD3	Missense	p.H337N	6	69	8.7%	.	.	.
GC13	19	40902016	G	T	PRX	Missense	p.P748Q	7	92	7.6%	.	.	.
GC13	19	40929425	C	A	SERTAD1	Missense	p.R10L	8	92	8.7%	.	.	.
GC13	19	41265412	G	T	SNRPA	Missense	p.R108L	6	83	7.2%	.	.	.
GC13	19	41600280	G	T	CYP2A13	Missense	p.M368I	6	45	13.3%	.	.	.
GC13	19	41709379	G	T	CYP2S1	Missense	p.R334L	7	38	18.4%	.	.	.
GC13	19	41711966	G	T	CYP2S1	Missense	p.R423L	5	68	7.4%	.	.	.
GC13	19	41778032	C	A	HNRNPUL1	Missense	p.P66Q	8	84	9.5%	.	.	.
GC13	19	41785075	C	A	HNRNPUL1	Missense	p.Q205K	5	34	14.7%	.	.	.

GC13	19	41800503	G	T	HNRNPUL1	Missense	p.W388L	7	96	7.3%	.	.	.
GC13	19	41811610	G	T	HNRNPUL1	Missense	p.Q675H	11	91	12.1%	.	.	.
GC13	19	41825668	G	T	CCDC97	Missense	p.R166L	5	64	7.8%	.	.	.
GC13	19	41950092	C	A	ERICH4	Missense	p.P119Q	7	59	11.9%	.	.	.
GC13	19	42225021	G	T	CEACAM5	Missense	p.G651W	7	85	8.2%	.	.	.
GC13	19	42407472	G	T	ARHGEF1	Missense	p.R552L	7	63	11.1%	.	.	.
GC13	19	42485904	C	A	ATP1A3	Missense	p.K435N	9	110	8.2%	.	.	.
GC13	19	42583947	G	T	ZNF574	Missense	p.G487W	7	99	7.1%	.	.	.
GC13	19	42729020	G	T	ZNF526	Missense	p.W155C	7	65	10.8%	.	.	.
GC13	19	42754613	G	T	ERF	Missense	p.Q43K	7	58	12.1%	.	.	.
GC13	19	42806137	C	A	PAFAH1B3	Missense	p.G45W	6	70	8.6%	.	.	.
GC13	19	42824575	C	A	TMEM145	Missense	p.H394N	7	42	16.7%	.	.	.
GC13	19	42839222	G	T	MEGF8	Missense	p.L198F	7	80	8.8%	.	.	.
GC13	19	42862352	G	T	MEGF8	Missense	p.G1623W	6	45	13.3%	.	.	.
GC13	19	42930562	G	T	LIPE	Missense	p.P247Q	7	73	9.6%	.	.	.
GC13	19	43026120	G	T	CEACAM1	Missense	p.P220Q	5	40	12.5%	.	.	.
GC13	19	43965675	C	A	LYPD3	Missense	p.R290L	5	43	11.6%	.	.	.
GC13	19	44002008	C	A	PHLDB3	Missense	p.Q185H	6	40	15.0%	.	.	.
GC13	19	44039454	C	A	ZNF575	Missense	p.P118Q	4	32	12.5%	.	.	.
GC13	19	44352212	C	A	ZNF283	Missense	p.H348N	10	82	12.2%	.	.	.
GC13	19	44352482	G	T	ZNF283	Missense	p.G438W	7	67	10.4%	.	.	.
GC13	19	44377893	G	T	ZNF404	Missense	p.P155H	7	98	7.1%	.	.	.
GC13	19	44423040	G	T	ZNF45	Missense	p.Q73K	6	38	15.8%	.	.	.
GC13	19	44470165	C	A	ZNF221	Missense	p.R171S	6	94	6.4%	.	.	.
GC13	19	44470936	G	T	ZNF221	Missense	p.G428W	9	90	10.0%	.	.	.
GC13	19	44590079	G	T	ZNF284	Missense	p.G150W	7	91	7.7%	.	.	.
GC13	19	44605369	G	T	ZNF224	Missense	p.G76W	8	87	9.2%	.	.	.
GC13	19	44612153	C	A	ZNF224	Missense	p.H614N	7	81	8.6%	.	.	.
GC13	19	44681508	C	A	ZNF226	Missense	p.P698Q	5	38	13.2%	.	.	.
GC13	19	44771056	G	T	ZNF233	Missense	p.L60F	7	100	7.0%	.	.	.
GC13	19	44791805	C	A	ZNF235	Missense	p.G595W	7	67	10.4%	.	.	.

GC13	19	44803014	C	A	ZNF235	Missense	p.R62M	7	98	7.1%	.	.	.
GC13	19	44832821	C	A	ZNF112	Missense	p.G436W	7	99	7.1%	.	.	.
GC13	19	44934177	G	T	ZNF229	Missense	p.P254H	10	111	9.0%	.	.	.
GC13	19	44934373	G	T	ZNF229	Missense	p.Q189K	7	70	10.0%	.	.	.
GC13	19	44946776	G	T	ZNF229	Missense	p.Q22K	8	102	7.8%	.	.	.
GC13	19	44981752	G	T	ZNF180	Missense	p.H291N	6	84	7.1%	.	.	.
GC13	19	45028102	C	A	CEACAM20	Missense	p.R130L	7	85	8.2%	.	.	.
GC13	19	45028189	C	A	CEACAM20	Missense	p.W101L	8	102	7.8%	.	.	.
GC13	19	45209063	G	T	CEACAM16	Missense	p.G289W	7	70	10.0%	.	.	.
GC13	19	45296780	G	T	CBLC	Missense	p.W350L	7	56	12.5%	.	.	.
GC13	19	45375236	G	T	NECTIN2	Missense	p.W202L	6	67	9.0%	.	.	.
GC13	19	45377238	C	A	NECTIN2	Missense	p.L281M	8	97	8.2%	.	.	.
GC13	19	45451783	G	T	APOC2	Missense	p.L16F	8	82	9.8%	.	.	.
GC13	19	45452045	G	T	APOC2	Missense	p.W48L	9	111	8.1%	.	.	.
GC13	19	45774952	G	T	MARK4	Missense	p.G258W	5	30	16.7%	.	.	.
GC13	19	46299338	C	A	RSPH6A	Missense	p.W648L	6	67	9.0%	.	.	.
GC13	19	46319221	C	A	SYMPK	Missense	p.R1192L	6	66	9.1%	.	.	.
GC13	19	46375847	C	A	FOXA3	Missense	p.P195H	7	74	9.5%	.	.	.
GC13	19	46417744	C	A	NANOS2	Missense	p.G70W	5	43	11.6%	.	.	.
GC13	19	46464343	C	A	NOVA2	Missense	p.G52W	6	46	13.0%	.	.	.
GC13	19	47423095	G	T	ARHGAP35	Missense	p.W388L	11	103	10.7%	.	.	.
GC13	19	47425269	C	A	ARHGAP35	Missense	p.Q1113K	8	104	7.7%	.	.	.
GC13	19	47492897	C	A	ARHGAP35	Missense	p.P1334Q	8	88	9.1%	.	.	.
GC13	19	47597262	G	T	ZC3H4	Missense	p.R153S	7	78	9.0%	.	.	.
GC13	19	47763593	C	A	CCDC9	Missense	p.R42S	5	46	10.9%	.	.	.
GC13	19	47844939	G	T	C5AR2	Missense	p.G295W	9	82	11.0%	.	.	.
GC13	19	47856385	C	A	DHX34	Missense	p.P33Q	7	62	11.3%	.	.	.
GC13	19	48058876	C	A	ZNF541	Missense	p.G80W	6	69	8.7%	.	.	.
GC13	19	48239677	G	T	EHD2	Missense	p.G323W	8	112	7.1%	.	.	.
GC13	19	48244309	C	A	EHD2	Missense	p.H418N	6	56	10.7%	.	.	.
GC13	19	48253495	G	T	NOP53	Missense	p.R117L	6	71	8.5%	.	.	.

GC13	19	48374722	G	T	SULT2A1	Missense	p.P283Q	8	88	9.1%	.	.	.
GC13	19	48715004	G	T	CARD8	Missense	p.P526H	7	99	7.1%	.	.	.
GC13	19	48876954	C	A	SYNGR4	Missense	p.R92S	5	51	9.8%	.	.	.
GC13	19	48953937	G	T	GRWD1	Missense	p.G233C	5	43	11.6%	.	.	.
GC13	19	49001336	G	T	LMTK3	Missense	p.P1026Q	7	94	7.4%	.	.	.
GC13	19	49001561	G	T	LMTK3	Missense	p.P951Q	7	83	8.4%	.	.	.
GC13	19	49001624	G	T	LMTK3	Missense	p.P930Q	7	86	8.1%	.	.	.
GC13	19	49004615	C	A	LMTK3	Missense	p.W338L	6	75	8.0%	.	.	.
GC13	19	49005839	C	A	LMTK3	Splicing	c.733-1G>T	7	70	10.0%	.	.	.
GC13	19	49013735	C	A	LMTK3	Missense	p.R93L	8	101	7.9%	.	.	.
GC13	19	49207078	G	T	FUT2	Missense	p.G289W	9	95	9.5%	.	.	.
GC13	19	49245075	G	T	IZUMO1	Missense	p.P129Q	6	57	10.5%	.	.	.
GC13	19	49253713	G	T	FUT1	Missense	p.Q276K	7	63	11.1%	.	.	.
GC13	19	49254006	C	A	FUT1	Missense	p.R178L	6	74	8.1%	.	.	.
GC13	19	49316572	C	A	HSD17B14	Missense	p.G225W	6	46	13.0%	.	.	.
GC13	19	49388742	G	T	TULP2	Missense	p.L342I	8	125	6.4%	.	.	.
GC13	19	49488804	C	A	GYS1	Missense	p.R182M	7	43	16.3%	.	.	.
GC13	19	49593727	G	T	SNRNP70	Missense	p.R72L	7	109	6.4%	.	.	.
GC13	19	49639985	G	T	PPFIA3	Missense	p.W557L	7	70	10.0%	.	.	.
GC13	19	49658164	G	T	HRC	Missense	p.H111N	7	72	9.7%	.	.	.
GC13	19	49658233	G	T	HRC	Missense	p.R88S	6	77	7.8%	.	.	.
GC13	19	49675364	G	T	TRPM4	Missense	p.K268N	8	79	10.1%	.	.	.
GC13	19	49691898	G	T	TRPM4	Missense	p.G228C	8	104	7.7%	.	.	.
GC13	19	49813335	C	A	SLC6A16	Missense	p.W221L	8	124	6.5%	.	.	.
GC13	19	49951149	C	A	PIH1D1	Missense	p.R139L	7	87	8.0%	.	.	.
GC13	19	49954821	G	T	PIH1D1	Missense	p.P4Q	6	74	8.1%	.	.	.
GC13	19	50031914	G	T	RCN3	Missense	p.R62L	6	72	8.3%	.	.	.
GC13	19	50156069	C	A	SCAF1	Missense	p.P808Q	8	67	11.9%	.	.	.
GC13	19	50169283	C	A	BCL2L12	Missense	p.P68Q	5	42	11.9%	.	.	.
GC13	19	50210846	G	T	CPT1C	Missense	p.W472L	6	49	12.2%	.	.	.
GC13	19	50247498	G	T	TSKS	Missense	p.R451S	4	23	17.4%	.	.	.

GC13	19	50295273	G	T	AP2A1	Missense	p.M185I	8	83	9.6%	.	.	.
GC13	19	50302893	G	T	AP2A1	Missense	p.R381L	7	67	10.4%	.	.	.
GC13	19	50303274	G	T	AP2A1	Missense	p.W441L	5	22	22.7%	.	.	.
GC13	19	50304773	G	T	AP2A1	Missense	p.Q560H	8	84	9.5%	.	.	.
GC13	19	50322432	G	T	MED25	Missense	p.G62W	6	66	9.1%	.	O	.
GC13	19	50363342	C	A	PTOV1	Missense	p.H381N	11	112	9.8%	.	.	.
GC13	19	50365074	C	A	PNKP	Missense	p.R418L	8	62	12.9%	.	.	.
GC13	19	50882398	G	T	NR1H2	Missense	p.R199L	5	66	7.6%	.	O	.
GC13	19	51021966	G	A	LRRC4B	Missense	p.T335M	21	96	21.9%	.	.	.
GC13	19	51135628	C	A	SYT3	Missense	p.G197W	8	83	9.6%	.	.	.
GC13	19	51537841	C	A	KLK12	Missense	p.G13W	6	53	11.3%	.	.	.
GC13	19	51585980	C	A	KLK14	Missense	p.G9W	7	59	11.9%	.	.	.
GC13	19	51628419	G	T	SIGLEC9	Missense	p.R63L	6	82	7.3%	.	.	.
GC13	19	51628482	G	T	SIGLEC9	Missense	p.W84L	7	70	10.0%	.	.	.
GC13	19	51630300	G	T	SIGLEC9	Missense	p.L254F	8	99	8.1%	.	.	.
GC13	19	51728621	G	T	CD33	Missense	p.R62L	6	76	7.9%	.	O	.
GC13	19	51892392	C	A	C19orf84	Missense	p.R136L	4	39	10.3%	.	.	.
GC13	19	52004719	C	A	SIGLEC12	Missense	p.R90L	7	82	8.5%	.	.	.
GC13	19	52031501	G	T	SIGLEC6	Missense	p.P288Q	8	81	9.9%	.	.	.
GC13	19	52090911	G	T	ZNF175	Missense	p.G443W	8	117	6.8%	.	.	.
GC13	19	52327549	G	T	FPR3	Missense	p.W183L	8	94	8.5%	.	.	.
GC13	19	52394839	C	A	ZNF649	Missense	p.G184W	8	120	6.7%	.	.	.
GC13	19	52468111	G	T	ZNF350	Missense	p.P532Q	6	68	8.8%	.	.	.
GC13	19	52497761	G	T	ZNF615	Missense	p.Q195K	8	130	6.2%	.	.	.
GC13	19	52497845	C	A	ZNF615	Missense	p.G167W	9	124	7.3%	.	.	.
GC13	19	52521357	C	A	ZNF614	Splicing	c.143-1G>T	5	41	12.2%	.	O	.
GC13	19	52569022	C	A	ZNF841	Missense	p.G589W	9	134	6.7%	.	.	.
GC13	19	52658823	G	T	ZNF836	Missense	p.Q705K	8	127	6.3%	.	.	.
GC13	19	52659278	G	T	ZNF836	Missense	p.P553H	8	102	7.8%	.	.	.
GC13	19	52659807	G	T	ZNF836	Missense	p.Q377K	6	70	8.6%	.	.	.
GC13	19	52918650	G	T	ZNF528	Missense	p.R182M	9	109	8.3%	.	.	.

GC13	19	52934734	C	A	ZNF534	Missense	p.L3I	6	67	9.0%	.	.	.
GC13	19	53573479	G	T	ZNF160	Missense	p.P103Q	6	64	9.4%	.	.	.
GC13	19	53611710	C	A	ZNF415	Missense	p.G300W	8	100	8.0%	.	.	.
GC13	19	53612486	G	T	ZNF415	Missense	p.P41Q	7	74	9.5%	.	.	.
GC13	19	54307230	G	T	NLRP12	Missense	p.P855Q	8	102	7.8%	.	.	.
GC13	19	54308627	C	A	NLRP12	Missense	p.R775M	7	73	9.6%	.	.	.
GC13	19	54314371	C	A	NLRP12	Missense	p.R181L	6	73	8.2%	.	.	.
GC13	19	54444776	G	T	CACNG7	Missense	p.E159D	6	55	10.9%	.	.	.
GC13	19	54606487	G	T	NDUFA3	Missense	p.G26W	6	65	9.2%	.	.	.
GC13	19	54649689	G	T	CNOT3	Missense	p.M249I	6	38	15.8%	O	.	.
GC13	19	54969664	G	T	LENG8	Missense	p.R735L	9	85	10.6%	.	.	.
GC13	19	55450418	G	T	NLRP7	Missense	p.P590Q	8	77	10.4%	.	.	.
GC13	19	55450836	C	A	NLRP7	Nonsense	p.G451X	6	81	7.4%	.	.	.
GC13	19	55451405	G	T	NLRP7	Missense	p.P261Q	8	91	8.8%	.	.	.
GC13	19	55493756	G	T	NLRP2	Missense	p.W208C	6	60	10.0%	.	.	.
GC13	19	55677200	G	T	DNAAF3	Missense	p.Q6K	6	77	7.8%	.	.	.
GC13	19	56011641	C	A	SSC5D	Missense	p.Q722K	7	64	10.9%	.	.	.
GC13	19	56113521	G	T	ZNF524	Missense	p.G15W	6	68	8.8%	.	.	.
GC13	19	56156155	G	T	ZNF581	Missense	p.R73M	7	72	9.7%	.	.	.
GC13	19	56156298	G	T	ZNF581	Missense	p.G121W	8	85	9.4%	.	.	.
GC13	19	56173935	C	A	U2AF2	Missense	p.P185Q	7	91	7.7%	.	.	.
GC13	19	56180864	C	A	U2AF2	Missense	p.Q363K	6	56	10.7%	.	.	.
GC13	19	56223314	G	T	NLRP9	Missense	p.R899S	7	72	9.7%	.	.	.
GC13	19	56244542	G	T	NLRP9	Missense	p.Q219K	7	75	9.3%	.	.	.
GC13	19	56320262	G	T	NLRP11	Missense	p.L473I	8	121	6.6%	.	.	.
GC13	19	56369159	C	A	NLRP4	Missense	p.H134N	9	122	7.4%	.	.	.
GC13	19	56466995	C	A	NLRP8	Missense	p.P524Q	7	87	8.0%	.	.	.
GC13	19	56544984	C	A	NLRP5	Missense	p.L842I	8	84	9.5%	.	.	.
GC13	19	57035696	G	T	ZNF471	Missense	p.W13L	7	100	7.0%	.	.	.
GC13	19	57065445	C	A	ZFP28	Missense	p.Q431K	7	88	8.0%	.	.	.
GC13	19	57085812	G	T	ZNF470	Missense	p.W37L	7	91	7.7%	.	.	.

GC13	19	57088887	G	T	ZNF470	Missense	p.G364W	7	82	8.5%	.	.	.
GC13	19	57133188	G	T	ZNF71	Missense	p.R178L	6	81	7.4%	.	.	.
GC13	19	57175027	C	A	ZNF835	Missense	p.G514W	7	82	8.5%	.	.	.
GC13	19	57175129	C	A	ZNF835	Missense	p.G480W	7	81	8.6%	.	.	.
GC13	19	57328815	C	A	PEG3	Missense	p.R332M	7	92	7.6%	.	O	.
GC13	19	57328905	C	A	PEG3	Missense	p.R302L	7	89	7.9%	.	O	.
GC13	19	57329128	G	T	PEG3	Missense	p.P283Q	8	71	11.3%	.	O	.
GC13	19	57641715	G	T	USP29	Missense	p.G558W	7	69	10.1%	.	.	.
GC13	19	57642549	C	A	USP29	Missense	p.P836T	8	104	7.7%	.	.	.
GC13	19	57672072	G	T	DUXA	Missense	p.P40Q	7	88	8.0%	.	.	.
GC13	19	57722904	C	A	ZNF264	Missense	p.Q147K	11	119	9.2%	.	.	.
GC13	19	57724236	G	T	ZNF264	Missense	p.G591W	7	93	7.5%	.	.	.
GC13	19	57802513	G	T	ZNF460	Missense	p.G161W	7	89	7.9%	.	O	.
GC13	19	57802933	G	T	ZNF460	Missense	p.G301W	9	71	12.7%	.	.	.
GC13	19	57803200	C	A	ZNF460	Missense	p.R390S	7	97	7.2%	.	O	.
GC13	19	57839611	G	T	ZNF543	Missense	p.G261W	12	107	11.2%	.	.	.
GC13	19	57868054	C	A	ZNF304	Missense	p.H273N	7	88	8.0%	.	.	.
GC13	19	57868846	G	T	ZNF304	Missense	p.G537W	6	62	9.7%	.	.	.
GC13	19	57869122	C	A	ZNF304	Missense	p.H629N	5	40	12.5%	.	.	.
GC13	19	57931866	G	T	ZNF17	Missense	p.G336W	10	93	10.8%	.	.	.
GC13	19	57967497	C	A	VN1R1	Nonsense	p.G120X	7	85	8.2%	.	.	.
GC13	19	58049129	G	T	ZNF549	Missense	p.G240W	7	95	7.4%	.	.	.
GC13	19	58049348	C	A	ZNF549	Missense	p.H313N	7	85	8.2%	.	.	.
GC13	19	58101579	C	A	ZIK1	Missense	p.Q31K	7	95	7.4%	.	.	.
GC13	19	58117626	G	T	ZNF530	Missense	p.G245W	7	96	7.3%	.	.	.
GC13	19	58118565	C	A	ZNF530	Missense	p.R558S	5	53	9.4%	.	.	.
GC13	19	58152254	G	T	ZNF211	Missense	p.G73W	7	84	8.3%	.	.	.
GC13	19	58152641	G	T	ZNF211	Missense	p.G202W	9	112	8.0%	.	.	.
GC13	19	58196666	C	A	ZNF551	Missense	p.Q12K	6	47	12.8%	.	.	.
GC13	19	58213091	C	A	ZNF154	Missense	p.G409V	7	86	8.1%	.	.	.
GC13	19	58384495	G	T	ZNF814	Missense	p.H755N	7	53	13.2%	.	.	.

GC13	19	58578846	G	T	ZNF135	Missense	p.G344W	5	38	13.2%	.	.	.
GC13	19	58579062	C	A	ZNF135	Missense	p.H428N	6	61	9.8%	.	.	.
GC13	19	58928378	G	T	ZNF584	Missense	p.G165W	7	96	7.3%	.	.	.
GC13	19	58948456	G	T	ZNF132	Missense	p.H64N	8	92	8.7%	.	.	.
GC13	19	59012009	C	A	SLC27A5	Missense	p.W332L	8	81	9.9%	.	.	.
GC13	19	59022291	C	A	SLC27A5	Missense	p.R148L	6	49	12.2%	.	.	.
GC13	19	59059737	G	T	TRIM28	Missense	p.W393L	8	90	8.9%	.	.	.
GC13	20	168664	C	A	DEFB128	Missense	p.G49W	8	121	6.6%	.	.	.
GC13	20	278820	C	A	ZCCHC3	Missense	p.P198Q	6	91	6.6%	.	.	.
GC13	20	372055	G	T	TRIB3	Missense	p.R139L	7	78	9.0%	.	.	.
GC13	20	419782	C	A	TBC1D20	Missense	p.R309L	6	74	8.1%	.	.	.
GC13	20	1234927	G	T	RAD21L1	Missense	p.M499I	8	133	6.0%	.	.	.
GC13	20	1903262	C	A	SIRPA	Missense	p.P353Q	6	29	20.7%	.	.	.
GC13	20	2465026	G	T	ZNF343	Missense	p.P104H	8	107	7.5%	.	.	.
GC13	20	2591226	G	T	TMC2	Missense	p.M525I	6	64	9.4%	.	.	.
GC13	20	2819532	G	T	PCED1A	Missense	p.P83Q	6	64	9.4%	.	.	.
GC13	20	2846918	C	A	VPS16	Missense	p.R634S	6	95	6.3%	.	.	.
GC13	20	3128852	C	A	FASTKD5	Missense	p.G289C	7	74	9.5%	.	.	.
GC13	20	3147604	G	T	LZTS3	Missense	p.P69H	6	49	12.2%	.	.	.
GC13	20	3844932	G	T	MAVS	Missense	p.G78W	6	61	9.8%	.	.	.
GC13	20	3954991	G	T	RNF24	Missense	p.P15Q	6	66	9.1%	.	.	.
GC13	20	4158045	G	T	SMOX	Missense	p.G86W	7	45	15.6%	.	.	.
GC13	20	5559060	G	T	GPCPD1	Missense	p.P224Q	6	78	7.7%	.	.	.
GC13	20	5924640	G	T	TRMT6	Missense	p.R166S	8	112	7.1%	.	.	.
GC13	20	5966577	G	T	MCM8	Nonsense	p.G608X	8	93	8.6%	.	.	.
GC13	20	6096655	C	A	FERMT1	Missense	p.W63L	9	66	13.6%	.	.	.
GC13	20	9318679	C	A	PLCB4	Missense	p.L64I	10	79	12.7%	.	O	.
GC13	20	10620222	G	T	JAG1	Missense	p.P1194Q	9	121	7.4%	.	.	.
GC13	20	10633202	G	T	JAG1	Missense	p.P267Q	6	61	9.8%	.	.	.
GC13	20	13071839	C	A	SPTLC3	Missense	p.P239Q	6	54	11.1%	.	.	.
GC13	20	13561576	C	A	TASP1	Missense	p.K152N	7	57	12.3%	.	.	.

GC13	20	13789516	C	A	NDUFAF5	Missense	p.P221H	6	55	10.9%	.	.	.
GC13	20	13847431	C	A	SEL1L2	Missense	p.G441W	7	95	7.4%	.	.	.
GC13	20	14307371	C	A	FLRT3	Missense	p.R261L	7	82	8.5%	.	O	.
GC13	20	16492096	G	T	KIF16B	Missense	p.R175S	7	118	5.9%	.	O	.
GC13	20	16729075	C	A	OTOR	Missense	p.P10Q	12	100	12.0%	.	.	.
GC13	20	17968809	G	T	MGME1	Missense	p.Q244H	10	95	10.5%	.	.	.
GC13	20	17970663	C	A	MGME1	Missense	p.P198Q	8	110	7.3%	.	.	.
GC13	20	18038211	G	T	OVOL2	Missense	p.P23Q	5	32	15.6%	.	.	.
GC13	20	18142649	C	A	KAT14	Missense	p.P290T	7	94	7.4%	.	.	.
GC13	20	18296481	G	T	ZNF133	Missense	p.R310L	8	98	8.2%	.	.	.
GC13	20	18429646	C	A	DZANK1	Missense	p.R204M	9	125	7.2%	.	.	.
GC13	20	18429689	C	A	DZANK1	Missense	p.G190C	7	111	6.3%	.	.	.
GC13	20	18440944	G	T	DZANK1	Missense	p.P39Q	7	93	7.5%	.	.	.
GC13	20	18477751	G	T	RBBP9	Missense	p.H21N	8	80	10.0%	.	O	.
GC13	20	18523665	G	T	SEC23B	Missense	p.W505L	9	122	7.4%	.	.	.
GC13	20	19955699	G	T	RIN2	Missense	p.G344W	9	95	9.5%	.	.	.
GC13	20	20591985	G	T	RALGAPA2	Missense	p.Q592K	10	117	8.5%	.	.	.
GC13	20	21695335	G	T	PAX1	Missense	p.R500L	4	21	19.0%	.	.	.
GC13	20	21695433	G	T	PAX1	Missense	p.G533W	9	83	10.8%	.	.	.
GC13	20	23346167	G	T	GZF1	Missense	p.G383W	7	77	9.1%	.	.	.
GC13	20	23548945	G	T	CST9L	Missense	p.P48H	8	99	8.1%	.	O	.
GC13	20	24959480	G	T	APMAP	Missense	p.P84Q	5	49	10.2%	.	.	.
GC13	20	25193915	G	T	ENTPD6	Missense	p.R140L	6	90	6.7%	.	.	.
GC13	20	25657292	C	A	ZNF337	Missense	p.R211M	8	85	9.4%	.	.	.
GC13	20	29960930	G	T	DEFB118	Missense	p.R110M	7	86	8.1%	.	.	.
GC13	20	29976920	G	T	DEFB119	Missense	p.Q58K	7	87	8.0%	.	.	.
GC13	20	30359437	C	A	TPX2	Missense	p.P187Q	8	113	7.1%	.	.	.
GC13	20	30380537	G	T	TPX2	Splicing	c.1414-1G>T	7	86	8.1%	.	.	.
GC13	20	30605622	G	T	CCM2L	Missense	p.W73L	8	71	11.3%	.	.	.
GC13	20	30730828	G	T	TM9SF4	Missense	p.R191L	6	58	10.3%	.	.	.
GC13	20	31024521	G	T	ASXL1	Missense	p.G1336W	7	70	10.0%	O	.	.

GC13	20	31024839	G	T	ASXL1	Missense	p.G1442W	9	87	10.3%	O	.	.
GC13	20	31372613	C	A	DNMT3B	Missense	p.P85Q	6	47	12.8%	.	.	.
GC13	20	32212691	G	T	CBFA2T2	Missense	p.G272W	7	98	7.1%	.	.	.
GC13	20	32255579	G	T	ACTL10	Missense	p.E92D	7	95	7.4%	.	.	.
GC13	20	32332984	G	T	ZNF341	Missense	p.R73L	6	96	6.3%	.	.	.
GC13	20	32371589	G	T	ZNF341	Missense	p.G501W	6	58	10.3%	.	.	.
GC13	20	32436360	G	T	CHMP4B	Missense	p.R93L	6	39	15.4%	.	.	.
GC13	20	32661658	C	G	RALY	Missense	p.D121E	12	34	35.3%	.	.	.
GC13	20	32878142	G	T	AHCY	Missense	p.P323Q	6	75	8.0%	.	.	.
GC13	20	33000393	G	T	ITCH	Missense	p.L95F	8	138	5.8%	.	.	.
GC13	20	33329769	G	T	NCOA6	Missense	p.Q1431K	9	105	8.6%	.	.	.
GC13	20	33330745	C	A	NCOA6	Missense	p.L1105F	8	109	7.3%	.	.	.
GC13	20	33500939	C	A	ACSS2	Missense	p.H139N	7	81	8.6%	.	.	.
GC13	20	33502170	G	T	ACSS2	Missense	p.R255L	7	100	7.0%	.	.	.
GC13	20	33585435	C	A	MYH7B	Missense	p.R1289S	6	71	8.5%	.	.	.
GC13	20	33857680	G	T	MMP24	Missense	p.G438W	7	90	7.8%	.	.	.
GC13	20	33874618	G	T	FAM83C	Missense	p.P655Q	8	86	9.3%	.	O	.
GC13	20	33875006	C	A	FAM83C	Missense	p.G526W	5	40	12.5%	.	.	.
GC13	20	33875258	C	A	FAM83C	Missense	p.G442W	6	55	10.9%	.	.	.
GC13	20	34064316	C	A	CEP250	Missense	p.L587M	8	111	7.2%	.	O	.
GC13	20	34082393	C	A	CEP250	Missense	p.Q394K	8	58	13.8%	.	.	.
GC13	20	34089684	G	T	CEP250	Missense	p.W672L	8	106	7.5%	.	.	.
GC13	20	34092646	G	T	CEP250	Missense	p.R1518L	7	78	9.0%	.	.	.
GC13	20	34220447	G	T	CPNE1	Missense	p.L101I	7	78	9.0%	.	.	.
GC13	20	34242188	G	T	RBM12	Missense	p.P353T	7	88	8.0%	.	.	.
GC13	20	34459681	G	T	PHF20	Missense	p.M404I	7	99	7.1%	.	.	.
GC13	20	34526758	G	T	PHF20	Missense	p.G814W	7	58	12.1%	.	.	.
GC13	20	34541839	C	A	SCAND1	Missense	p.R186L	7	90	7.8%	.	.	.
GC13	20	34583011	C	A	CNBD2	Missense	p.R303S	6	72	8.3%	.	.	.
GC13	20	34778704	C	A	EPB41L1	Missense	p.R367S	6	57	10.5%	.	.	.
GC13	20	35060262	G	T	DLGAP4	Missense	p.G48W	6	72	8.3%	.	.	.

GC13	20	35545172	G	T	SAMHD1	Missense	p.R339S	7	128	5.5%	.	O	.
GC13	20	35555601	G	T	SAMHD1	Missense	p.P227Q	8	96	8.3%	.	.	.
GC13	20	35555613	G	T	SAMHD1	Missense	p.P223Q	8	93	8.6%	.	.	.
GC13	20	36611914	C	A	TTI1	Missense	p.G1072W	5	42	11.9%	.	.	.
GC13	20	37126094	G	T	RALGAPB	Missense	p.W163L	9	146	6.2%	.	.	.
GC13	20	37154079	C	A	RALGAPB	Missense	p.P607Q	8	136	5.9%	.	.	.
GC13	20	37202914	G	T	RALGAPB	Missense	p.G1422W	7	74	9.5%	.	.	.
GC13	20	37353692	G	T	SLC32A1	Missense	p.G109W	6	55	10.9%	.	.	.
GC13	20	37356291	C	A	SLC32A1	Missense	p.P196Q	7	79	8.9%	.	.	.
GC13	20	37657067	C	A	DHX35	Missense	p.P572Q	5	36	13.9%	.	.	.
GC13	20	39791139	G	T	PLCG1	Missense	p.R187L	5	62	8.1%	O	.	.
GC13	20	39833516	G	T	ZHX3	Missense	p.P14Q	7	66	10.6%	.	.	.
GC13	20	40162008	G	T	CHD6	Missense	p.H79N	8	99	8.1%	.	.	.
GC13	20	42142535	G	T	L3MBTL1	Missense	p.R43L	7	79	8.9%	.	O	.
GC13	20	42265804	G	T	IFT52	Missense	p.R344L	10	86	11.6%	.	.	.
GC13	20	42331253	C	A	MYBL2	Missense	p.R335S	7	77	9.1%	.	.	.
GC13	20	42935779	C	A	FITM2	Missense	p.R92L	6	53	11.3%	.	.	.
GC13	20	42965954	C	A	R3HDML	Missense	p.R53S	6	71	8.5%	.	.	.
GC13	20	42966058	G	T	R3HDML	Missense	p.M87I	7	54	13.0%	.	O	.
GC13	20	43108920	G	T	TTPAL	Missense	p.R94L	7	60	11.7%	.	.	.
GC13	20	43547680	C	A	PABPC1L	Missense	p.Q213K	6	56	10.7%	.	.	.
GC13	20	43836326	C	A	SEMG1	Missense	p.H130N	9	111	8.1%	.	.	.
GC13	20	44005933	C	A	TP53TG5	Missense	p.R58L	7	81	8.6%	.	.	.
GC13	20	44037157	G	T	DBNDD2	Missense	p.R119L	7	77	9.1%	.	.	.
GC13	20	44045217	G	T	PIGT	Missense	p.R83L	7	69	10.1%	.	.	.
GC13	20	44469471	G	T	SNX21	Missense	p.R214L	7	73	9.6%	.	.	.
GC13	20	44507260	C	A	ZSWIM3	Missense	p.P688H	7	69	10.1%	.	.	.
GC13	20	44534908	C	A	PLTP	Missense	p.R140L	7	79	8.9%	.	.	.
GC13	20	44598239	C	A	ZNF335	Missense	p.G98V	6	60	10.0%	.	.	.
GC13	20	44683658	G	T	SLC12A5	Missense	p.R951L	5	67	7.5%	.	.	.
GC13	20	45003071	C	A	ELMO2	Missense	p.L393F	6	68	8.8%	.	.	.

GC13	20	45017855	C	A	ELMO2	Missense	p.R83L	4	27	14.8%	.	.	.
GC13	20	45130078	C	A	ZNF334	Missense	p.G634W	8	89	9.0%	.	.	.
GC13	20	45700877	G	T	EYA2	Missense	p.G157W	6	54	11.1%	.	.	.
GC13	20	45874880	G	T	ZMYND8	Missense	p.P647Q	8	72	11.1%	.	.	.
GC13	20	46256335	A	T	NCOA3	Missense	p.Q188L	31	94	33.0%	.	.	.
GC13	20	46264415	G	T	NCOA3	Missense	p.G488W	9	140	6.4%	.	.	.
GC13	20	46331406	C	A	SULF2	Missense	p.G142W	9	79	11.4%	.	.	.
GC13	20	47297776	G	T	PREX1	Missense	p.H478N	5	32	15.6%	.	.	.
GC13	20	47361582	C	A	PREX1	Missense	p.G132W	7	78	9.0%	.	.	.
GC13	20	47587678	G	T	ARFGEF2	Missense	p.K404N	8	82	9.8%	.	.	.
GC13	20	47607718	G	T	ARFGEF2	Missense	p.G986W	9	109	8.3%	.	.	.
GC13	20	47645178	G	T	ARFGEF2	Missense	p.W1679L	7	90	7.8%	.	.	.
GC13	20	48479509	G	T	SLC9A8	Missense	p.G269W	11	132	8.3%	.	.	.
GC13	20	48600460	G	T	SNAI1	Missense	p.W61L	9	78	11.5%	.	.	.
GC13	20	49195011	G	T	PTPN1	Nonsense	p.G110X	8	115	7.0%	.	.	.
GC13	20	49366427	G	T	PARD6B	Missense	p.R174L	8	104	7.7%	.	.	.
GC13	20	49366502	C	A	PARD6B	Missense	p.P199Q	7	90	7.8%	.	.	.
GC13	20	49509393	C	A	ADNP	Missense	p.G620W	8	96	8.3%	.	.	.
GC13	20	50342368	C	A	ATP9A	Missense	p.W106L	6	67	9.0%	.	.	.
GC13	20	50769424	C	A	ZFP64	Missense	p.R382L	6	90	6.7%	.	.	.
GC13	20	52188302	G	T	ZNF217	Missense	p.P1043Q	8	136	5.9%	.	.	.
GC13	20	52192758	G	T	ZNF217	Missense	p.P849T	7	92	7.6%	.	.	.
GC13	20	55101082	C	A	FAM209A	Missense	p.H158N	10	116	8.6%	.	.	.
GC13	20	57036436	C	A	APCDD1L	Missense	p.G306W	4	23	17.4%	.	.	.
GC13	20	57430169	G	T	GNAS	Missense	p.G617W	6	96	6.3%	O	.	.
GC13	20	57564685	C	A	NELFCD	Missense	p.P234H	8	96	8.3%	.	.	.
GC13	20	57896229	C	A	EDN3	Missense	p.Q175K	8	84	9.5%	.	.	.
GC13	20	58381189	C	A	PHACTR3	Missense	p.P312H	7	95	7.4%	.	.	.
GC13	20	58547143	C	A	CDH26	Missense	p.R120S	6	92	6.5%	.	.	.
GC13	20	60448840	G	T	CDH4	Missense	p.G275W	5	67	7.5%	.	.	.
GC13	20	60572680	G	T	TAF4	Missense	p.L1006I	7	80	8.8%	.	.	.

GC13	20	60904260	C	A	LAMA5	Missense	p.R1390L	6	63	9.5%	.	.	.
GC13	20	61467272	G	T	COL9A3	Splicing	c.1369-1G>T	8	93	8.6%	.	.	.
GC13	20	61833775	C	A	YTHDF1	Missense	p.R506L	7	110	6.4%	.	.	.
GC13	20	61834631	C	A	YTHDF1	Missense	p.G221W	6	85	7.1%	.	.	.
GC13	20	61982320	C	A	CHRNA4	Missense	p.R148L	6	47	12.8%	.	O	.
GC13	20	62153068	G	T	PPDPF	Missense	p.G61W	8	107	7.5%	.	.	.
GC13	20	62168657	C	A	PTK6	Missense	p.R4L	6	26	23.1%	O	.	.
GC13	20	62198582	C	A	HELZ2	Missense	p.R141L	5	29	17.2%	.	.	.
GC13	20	62594561	C	A	ZNF512B	Missense	p.G619W	4	20	20.0%	.	.	.
GC13	20	62597969	C	A	ZNF512B	Missense	p.G187W	6	63	9.5%	.	.	.
GC13	20	62616275	G	T	PRPF6	Missense	p.G86W	9	98	9.2%	.	.	.
GC13	20	62904691	C	A	PCMTD2	Missense	p.P248H	8	106	7.5%	.	.	.
GC13	21	16337127	C	A	NRIP1	Missense	p.M1129I	7	80	8.8%	.	.	.
GC13	21	16337529	C	A	NRIP1	Missense	p.M995I	8	106	7.5%	.	.	.
GC13	21	16337662	G	T	NRIP1	Missense	p.P951Q	8	120	6.7%	.	.	.
GC13	21	19629017	G	T	CHODL	Missense	p.G72W	7	120	5.8%	.	.	.
GC13	21	22782636	G	T	NCAM2	Missense	p.W413L	11	99	11.1%	.	O	.
GC13	21	27121428	C	A	GABPA	Missense	p.Q102K	9	88	10.2%	.	.	.
GC13	21	27840936	C	A	CYYR1	Missense	p.G226C	7	80	8.8%	.	.	.
GC13	21	28212680	G	T	ADAMTS1	Missense	p.P527Q	8	99	8.1%	.	.	.
GC13	21	28296416	G	T	ADAMTS5	Missense	p.Q917K	8	126	6.3%	.	.	.
GC13	21	30309015	C	A	LTN1	Missense	p.R1528L	5	42	11.9%	.	.	.
GC13	21	30332968	C	A	LTN1	Missense	p.G774C	7	96	7.3%	.	.	.
GC13	21	30409751	G	T	USP16	Missense	p.L200F	11	153	7.2%	.	.	.
GC13	21	30419120	C	A	USP16	Missense	p.H496N	9	141	6.4%	.	.	.
GC13	21	30419499	G	T	USP16	Missense	p.R622M	8	117	6.8%	.	.	.
GC13	21	30699645	G	T	BACH1	Missense	p.L500F	7	81	8.6%	.	.	.
GC13	21	31538653	G	T	CLDN17	Missense	p.L95M	8	98	8.2%	.	.	.
GC13	21	31655055	C	A	KRTAP24-1	Missense	p.G66C	8	87	9.2%	.	.	.
GC13	21	31864070	G	T	KRTAP19-3	Missense	p.P69Q	6	85	7.1%	.	.	.
GC13	21	31864074	G	T	KRTAP19-3	Missense	p.R68S	6	80	7.5%	.	.	.

GC13	21	31986064	G	T	KRTAP6-1	Missense	p.R54S	6	83	7.2%	.	.	.
GC13	21	33040886	C	A	SOD1	Missense	p.Q154K	8	108	7.4%	.	.	.
GC13	21	33064748	G	T	SCAF4	Missense	p.L495I	8	102	7.8%	.	.	.
GC13	21	33331183	G	T	HUNK	Missense	p.G259W	6	46	13.0%	.	.	.
GC13	21	33723038	C	A	URB1	Missense	p.R936L	7	61	11.5%	.	.	.
GC13	21	33723134	C	A	URB1	Missense	p.R904L	5	62	8.1%	.	.	.
GC13	21	33982200	G	T	C21orf59	Missense	p.P9Q	11	108	10.2%	.	.	.
GC13	21	34134564	C	A	PAXBP1	Missense	p.L238F	9	116	7.8%	.	.	.
GC13	21	34725973	G	T	IFNAR1	Nonsense	p.G442X	6	62	9.7%	.	.	.
GC13	21	34954362	C	A	DONSON	Splicing	c.1047-1G>T	9	131	6.9%	.	.	.
GC13	21	34969611	C	A	CRYZL1	Missense	p.W258L	9	89	10.1%	.	.	.
GC13	21	34971468	G	T	CRYZL1	Missense	p.L222I	10	119	8.4%	.	.	.
GC13	21	35743007	G	T	KCNE2	Missense	p.R77L	7	74	9.5%	.	.	.
GC13	21	35897651	C	A	RCAN1	Missense	p.W21L	9	107	8.4%	.	.	.
GC13	21	36231876	C	A	RUNX1	Splicing	c.428-1G>T;NM_001122607	7	68	10.3%	.	.	.
GC13	21	37605307	G	T	DOPEY2	Missense	p.M852I	7	91	7.7%	.	.	.
GC13	21	37609603	G	T	DOPEY2	Missense	p.R889L	6	90	6.7%	.	.	.
GC13	21	37617994	G	T	DOPEY2	Missense	p.R1239L	7	94	7.4%	.	.	.
GC13	21	38390221	G	T	RIPPLY3	Missense	p.G12V	8	118	6.8%	.	.	.
GC13	21	38390245	C	A	RIPPLY3	Missense	p.P20Q	8	109	7.3%	.	.	.
GC13	21	38525559	C	A	TTC3	Missense	p.Q598K	7	92	7.6%	.	.	.
GC13	21	38529167	C	A	TTC3	Missense	p.P674Q	8	109	7.3%	.	.	.
GC13	21	38537932	C	A	TTC3	Missense	p.P829Q	10	117	8.5%	.	.	.
GC13	21	38564456	G	T	TTC3	Missense	p.R1410L	5	53	9.4%	.	.	.
GC13	21	38884559	G	T	DYRK1A	Missense	p.G635C	8	110	7.3%	.	.	.
GC13	21	39671754	G	T	KCNJ15	Missense	p.G191W	10	86	11.6%	.	.	.
GC13	21	39755585	C	A	ERG	Missense	p.G302W	7	58	12.1%	.	O	.
GC13	21	40191535	G	T	ETS2	Missense	p.R307L	6	75	8.0%	.	.	.
GC13	21	40871822	C	A	SH3BGR	Missense	p.P81H	7	43	16.3%	.	.	.
GC13	21	41142847	G	T	IGSF5	Missense	p.M141I	7	85	8.2%	.	.	.
GC13	21	41300911	G	T	PCP4	Nonsense	p.G22X	6	57	10.5%	.	.	.

GC13	21	41516526	G	T	DSCAM	Missense	p.L1051M	6	56	10.7%	.	.	.
GC13	21	43327232	G	T	C2CD2	Missense	p.P241H	7	72	9.7%	.	.	.
GC13	21	43711229	G	T	ABCG1	Missense	p.R486L	6	80	7.5%	.	.	.
GC13	21	43716318	G	T	ABCG1	Missense	p.R618L	7	46	15.2%	.	.	.
GC13	21	43716431	G	T	ABCG1	Missense	p.G656W	6	95	6.3%	.	.	.
GC13	21	44117587	G	T	PDE9A	Missense	p.W94L	6	50	12.0%	.	.	.
GC13	21	44329088	G	T	NDUFV3	Missense	p.R95M	8	107	7.5%	.	.	.
GC13	21	44339057	C	A	ERVH48-1	Missense	p.M20I	8	75	10.7%	.	.	.
GC13	21	44590502	G	T	LOC102724652	Missense	p.G21W	6	56	10.7%	.	.	.
GC13	21	45194631	G	T	CSTB	Missense	p.Q26K	8	105	7.6%	.	.	.
GC13	21	45494215	C	A	TRAPPC10	Missense	p.P350Q	5	23	21.7%	.	.	.
GC13	21	45509814	G	T	TRAPPC10	Missense	p.R490L	6	62	9.7%	.	.	.
GC13	21	45540909	G	T	PWP2	Missense	p.W521L	8	79	10.1%	.	.	.
GC13	21	45858957	G	T	TRPM2	Missense	p.R73L	6	45	13.3%	.	.	.
GC13	21	46309221	G	T	ITGB2	Missense	p.P616H	6	39	15.4%	.	.	.
GC13	21	46624592	G	T	ADARB1	Missense	p.R563L	5	60	8.3%	.	.	.
GC13	21	46888440	G	T	COL18A1	Missense	p.G311W	5	65	7.7%	.	.	.
GC13	21	47361603	G	T	PCBP3	Missense	p.G341W	7	73	9.6%	.	.	.
GC13	21	47545402	C	A	COL6A2	Missense	p.L614M	7	77	9.1%	.	.	.
GC13	21	47545963	G	T	COL6A2	Missense	p.R745L	6	75	8.0%	.	.	.
GC13	21	47633718	C	A	LSS	Missense	p.W268L	7	82	8.5%	.	.	.
GC13	21	47773930	C	A	PCNT	Missense	p.P452H	6	39	15.4%	.	.	.
GC13	21	47918539	G	T	DIP2A	Missense	p.G150W	6	71	8.5%	.	.	.
GC13	21	47978274	G	T	DIP2A	Missense	p.G1309W	6	51	11.8%	.	.	.
GC13	21	48080822	C	A	PRMT2	Missense	p.P303Q	7	119	5.9%	.	.	.
GC13	21	48083454	C	A	PRMT2	Missense	p.H272N	6	54	11.1%	.	.	.
GC13	22	18894210	-	GCGCCT	DGCR6	Nonframeshift	p.Q81delinsQAP	9	30	30.0%	.	.	.
GC13	22	19119316	G	T	TSSK2	Missense	p.R135L	7	93	7.5%	.	.	.
GC13	22	19766919	C	A	TBX1	Missense	p.R396S	6	56	10.7%	.	.	.
GC13	22	20077261	C	A	DGCR8	Missense	p.P317Q	6	61	9.8%	.	.	.
GC13	22	20077333	G	T	DGCR8	Missense	p.R341L	6	78	7.7%	.	.	.

GC13	22	20094854	C	A	DGCR8	Missense	p.P653Q	9	90	10.0%	.	.	.
GC13	22	20819391	G	T	KLHL22	Missense	p.P289Q	6	47	12.8%	.	.	.
GC13	22	21331211	G	T	AIFM3	Missense	p.R401L	6	66	9.1%	.	.	.
GC13	22	21336833	C	A	LZTR1	Missense	p.P58Q	6	70	8.6%	O	.	.
GC13	22	21347092	G	T	LZTR1	Missense	p.G387W	6	66	9.1%	O	.	.
GC13	22	21354981	G	T	THAP7	Missense	p.P111Q	6	64	9.4%	.	.	.
GC13	22	21384184	C	A	SLC7A4	Missense	p.W480L	7	66	10.6%	.	.	.
GC13	22	21991327	C	A	CCDC116	Missense	p.Q604K	8	114	7.0%	.	.	.
GC13	22	22035587	G	T	PPIL2	Splicing	c.296-1G>T;NM_014337	7	56	12.5%	.	.	.
GC13	22	24122806	G	T	MMP11	Missense	p.G174W	8	64	12.5%	.	.	.
GC13	22	24159017	C	A	SMARCB1	Missense	p.P221Q	6	97	6.2%	O	O	.
GC13	22	24579506	G	T	SUSD2	Missense	p.G111W	7	79	8.9%	.	.	.
GC13	22	24584215	G	T	SUSD2	Missense	p.L788F	7	74	9.5%	.	.	.
GC13	22	24717846	C	A	SPECC1L	Missense	p.P300T	9	125	7.2%	.	.	.
GC13	22	24982137	C	A	LRRC75B	Missense	p.R222L	6	73	8.2%	.	.	.
GC13	22	26107060	G	T	GRK3	Missense	p.R474L	7	109	6.4%	.	.	.
GC13	22	26695065	G	T	SEZ6L	Missense	p.Q426H	5	36	13.9%	.	.	.
GC13	22	26736565	G	T	SEZ6L	Nonsense	p.G727X	7	95	7.4%	.	.	.
GC13	22	26761462	G	T	SEZ6L	Missense	p.M844I	6	72	8.3%	.	.	.
GC13	22	26829879	G	T	ASPHD2	Missense	p.G100W	8	92	8.7%	.	.	.
GC13	22	26868809	G	T	HPS4	Missense	p.L120I	8	82	9.8%	.	.	.
GC13	22	26884183	G	T	SRRD	Missense	p.G147W	11	120	9.2%	.	.	.
GC13	22	26892784	G	T	TFIP11	Missense	p.P472Q	6	58	10.3%	.	.	.
GC13	22	27026438	G	A	CRYBA4	Missense	p.R193K	16	63	25.4%	.	.	.
GC13	22	28385895	G	T	TTC28	Missense	p.P1893Q	9	70	12.9%	.	.	.
GC13	22	28492288	G	T	TTC28	Missense	p.P1219Q	6	57	10.5%	.	.	.
GC13	22	29446186	C	A	ZNRF3	Missense	p.L673M	7	72	9.7%	.	.	.
GC13	22	29456650	C	A	C22orf31	Missense	p.W62L	8	86	9.3%	.	.	.
GC13	22	29521353	G	T	KREMEN1	Missense	p.G194W	6	63	9.5%	.	.	.
GC13	22	29924099	G	T	THOC5	Missense	p.P345Q	7	98	7.1%	.	.	.
GC13	22	30000043	C	A	NF2	Missense	p.P19H	6	59	10.2%	O	.	.

GC13	22	30200649	G	T	ASCC2	Missense	p.P368Q	6	72	8.3%	.	.	.
GC13	22	30200730	G	T	ASCC2	Missense	p.P341Q	7	96	7.3%	.	.	.
GC13	22	30202271	C	A	ASCC2	Missense	p.R289L	5	44	11.4%	.	.	.
GC13	22	30416142	C	A	MTMR3	Missense	p.Q832K	7	73	9.6%	.	.	.
GC13	22	30518133	G	T	HORMAD2	Missense	p.R162L	7	109	6.4%	.	.	.
GC13	22	30730597	C	A	SF3A1	Missense	p.G790W	5	56	8.9%	.	.	.
GC13	22	30737719	G	T	SF3A1	Missense	p.Q345K	8	71	11.3%	.	.	.
GC13	22	30811946	G	T	SEC14L2	Missense	p.G178W	6	77	7.8%	.	.	.
GC13	22	30857669	C	A	SEC14L3	Missense	p.G262W	6	63	9.5%	.	.	.
GC13	22	30862353	G	T	SEC14L3	Missense	p.P183Q	8	81	9.9%	.	.	.
GC13	22	30934885	C	A	SEC14L6	Missense	p.R20L	7	68	10.3%	.	.	.
GC13	22	31330985	C	A	MORC2	Missense	p.R659L	5	43	11.6%	.	.	.
GC13	22	31338126	G	T	MORC2	Missense	p.Q187K	7	87	8.0%	.	.	.
GC13	22	31486986	G	T	SMTN	Missense	p.R382L	8	89	9.0%	.	.	.
GC13	22	31487756	G	T	SMTN	Missense	p.A575S	23	59	39.0%	.	.	.
GC13	22	31524488	C	A	INPP5J	Missense	p.R246S	6	62	9.7%	.	.	.
GC13	22	31644755	G	T	LIMK2	Missense	p.R18L	5	46	10.9%	.	.	.
GC13	22	31654389	C	A	LIMK2	Missense	p.L56M	6	56	10.7%	.	.	.
GC13	22	31667153	G	T	LIMK2	Missense	p.R429L	7	62	11.3%	.	.	.
GC13	22	31723110	C	A	PATZ1	Missense	p.G565W	7	83	8.4%	.	.	.
GC13	22	31851241	G	T	EIF4ENIF1	Missense	p.P224Q	7	93	7.5%	.	.	.
GC13	22	32099690	G	T	PRR14L	Missense	p.P1949H	8	94	8.5%	.	.	.
GC13	22	32110478	G	T	PRR14L	Missense	p.P1116Q	11	125	8.8%	.	.	.
GC13	22	32112974	G	T	PRR14L	Missense	p.P284Q	7	91	7.7%	.	.	.
GC13	22	32626984	G	T	SLC5A4	Missense	p.P367H	6	61	9.8%	.	.	.
GC13	22	32633312	C	A	SLC5A4	Splicing	c.584-1G>T	7	66	10.6%	.	.	.
GC13	22	33255245	G	T	TIMP3	Missense	p.G173C	6	99	6.1%	.	.	.
GC13	22	33828179	G	T	LARGE1	Missense	p.P287Q	6	52	11.5%	.	.	.
GC13	22	35947586	G	T	RASD2	Missense	p.R103L	4	26	15.4%	.	.	.
GC13	22	36661720	G	T	APOL1	Missense	p.G262W	9	93	9.7%	.	.	.
GC13	22	37531477	C	A	IL2RB	Missense	p.G237W	6	39	15.4%	.	O	.

GC13	22	37535207	C	A	IL2RB	Missense	p.G113V	6	58	10.3%	.	.	.
GC13	22	37540145	C	A	IL2RB	Missense	p.W23L	8	84	9.5%	.	.	.
GC13	22	37578652	G	T	C1QTNF6	Missense	p.P138Q	6	92	6.5%	.	.	.
GC13	22	37769233	C	A	ELFN2	Missense	p.R781L	6	84	7.1%	.	.	.
GC13	22	37769315	C	A	ELFN2	Missense	p.G754W	7	78	9.0%	.	.	.
GC13	22	37770206	G	T	ELFN2	Missense	p.Q457K	6	65	9.2%	.	O	.
GC13	22	38038979	G	T	SH3BP1	Missense	p.R121M	6	59	10.2%	.	.	.
GC13	22	38120161	G	T	TRIOBP	Missense	p.R533L	5	44	11.4%	.	.	.
GC13	22	38120671	G	T	TRIOBP	Missense	p.R703L	6	38	15.8%	.	.	.
GC13	22	38121564	G	T	TRIOBP	Missense	p.G1001W	7	85	8.2%	.	.	.
GC13	22	38121906	G	T	TRIOBP	Missense	p.G1115W	9	90	10.0%	.	O	.
GC13	22	38621508	C	A	TMEM184B	Missense	p.R237L	7	67	10.4%	.	.	.
GC13	22	38823048	G	T	KCNJ4	Missense	p.P364T	7	84	8.3%	.	.	.
GC13	22	38875643	G	T	KDELR3	Missense	p.G80W	9	96	9.4%	.	.	.
GC13	22	39222573	C	A	NPTXR	Missense	p.G344W	6	67	9.0%	.	.	.
GC13	22	39530534	G	T	CBX7	Missense	p.P157Q	4	23	17.4%	.	.	.
GC13	22	39773628	G	T	SYNGR1	Missense	p.R170L	6	65	9.2%	.	.	.
GC13	22	39883504	G	T	MGAT3	Missense	p.W51L	8	97	8.2%	.	.	.
GC13	22	39909891	G	T	MIEF1	Missense	p.G319C	7	125	5.6%	.	.	.
GC13	22	40060095	G	T	CACNA1I	Splicing	c.3493-1G>T	8	95	8.4%	.	.	.
GC13	22	40257905	G	T	ENTHD1	Missense	p.H153N	7	76	9.2%	.	.	.
GC13	22	40661699	C	A	TNRC6B	Missense	p.Q489K	7	82	8.5%	.	.	.
GC13	22	40801780	G	T	SGSM3	Missense	p.R186L	5	23	21.7%	.	.	.
GC13	22	40804997	C	A	SGSM3	Missense	p.R566S	5	47	10.6%	.	.	.
GC13	22	41513381	G	T	EP300	Missense	p.M95I	7	92	7.6%	O	.	.
GC13	22	41742130	C	A	ZC3H7B	Missense	p.P528Q	5	39	12.8%	.	.	.
GC13	22	41790216	G	T	TEF	Missense	p.G168W	6	77	7.8%	.	.	.
GC13	22	41864641	G	T	PHF5A	Missense	p.P6H	7	73	9.6%	.	O	.
GC13	22	41922272	G	T	ACO2	Missense	p.G590W	7	65	10.8%	.	.	.
GC13	22	42190392	C	A	MEI1	Missense	p.Q1149K	7	83	8.4%	.	.	.
GC13	22	42205910	G	T	CCDC134	Missense	p.R44L	7	92	7.6%	.	O	.

GC13	22	42262874	C	A	SREBF2	Missense	p.P43H	7	83	8.4%	.	.	.
GC13	22	42269886	G	T	SREBF2	Missense	p.G318W	6	65	9.2%	.	.	.
GC13	22	42341311	G	T	CENPM	Missense	p.Q71K	8	108	7.4%	.	.	.
GC13	22	42464497	C	A	NAGA	Missense	p.W33L	6	70	8.6%	.	.	.
GC13	22	42607228	G	T	TCF20	Missense	p.P1362T	8	85	9.4%	.	.	.
GC13	22	42610840	C	A	TCF20	Missense	p.G158W	8	75	10.7%	.	.	.
GC13	22	44368202	G	T	SAMM50	Nonsense	p.G137X	8	117	6.8%	.	.	.
GC13	22	45927181	C	A	FBLN1	Missense	p.P174Q	7	74	9.5%	.	.	.
GC13	22	46096218	C	A	ATXN10	Missense	p.Q86K	8	112	7.1%	.	.	.
GC13	22	46653728	G	T	PKDREJ	Missense	p.P1831Q	8	116	6.9%	.	.	.
GC13	22	46654866	G	T	PKDREJ	Missense	p.Q1452K	8	107	7.5%	.	.	.
GC13	22	46656908	C	A	PKDREJ	Missense	p.W771L	8	92	8.7%	.	.	.
GC13	22	46704680	G	T	GTSE1	Missense	p.R201L	9	79	11.4%	.	.	.
GC13	22	46777743	C	A	CELSR1	Missense	p.R2363L	6	47	12.8%	.	.	.
GC13	22	50278316	C	A	ZBED4	Missense	p.R336S	9	76	11.8%	.	.	.
GC13	22	50280312	G	T	ZBED4	Missense	p.R1001L	8	104	7.7%	.	.	.
GC13	22	50580531	C	A	MOV10L1	Missense	p.Q698K	7	88	8.0%	.	.	.
GC13	22	50682534	C	A	TUBGCP6	Missense	p.G119W	8	87	9.2%	.	.	.
GC13	22	50688386	C	A	HDAC10	Missense	p.G133W	5	70	7.1%	.	.	.
GC13	22	50695546	G	T	MAPK12	Missense	p.Q131K	7	74	9.5%	.	.	.
GC13	22	50724284	G	T	PLXNB2	Missense	p.P678H	9	74	12.2%	.	.	.
GC13	22	50752847	C	A	DENND6B	Missense	p.G352W	6	79	7.6%	.	.	.
GC13	22	51010636	C	A	CPT1B	Missense	p.W451L	6	66	9.1%	.	.	.
GC13	22	51160327	G	T	SHANK3	Missense	p.G1342W	5	43	11.6%	.	.	.
GC13	22	51160703	G	T	SHANK3	Missense	p.R1467L	5	51	9.8%	.	.	.
GC13	X	2839965	C	A	ARSD	Missense	p.G99W	7	67	10.4%	.	.	.
GC13	X	2933094	G	T	ARSH	Missense	p.G142W	6	76	7.9%	.	O	.
GC13	X	2945467	A	G	ARSH	Missense	p.I384V	19	41	46.3%	.	.	.
GC13	X	2994615	G	T	ARSF	Missense	p.R63M	6	32	18.8%	.	.	.
GC13	X	3238497	C	A	MXRA5	Missense	p.L1743F	5	35	14.3%	.	.	.
GC13	X	3239217	C	A	MXRA5	Missense	p.L1503F	7	62	11.3%	.	.	.

GC13	X	3241352	C	A	MXRA5	Missense	p.G792W	9	67	13.4%	.	.	.
GC13	X	3248294	C	A	MXRA5	Missense	p.L158F	6	55	10.9%	.	.	.
GC13	X	7268021	G	T	STS	Missense	p.G491W	7	82	8.5%	.	.	.
GC13	X	8556011	G	T	ANOS1	Missense	p.L184M	5	26	19.2%	.	.	.
GC13	X	10535076	C	A	MID1	Missense	p.R171L	6	66	9.1%	.	.	.
GC13	X	11130183	G	T	HCCS	Missense	p.M1I	5	39	12.8%	.	.	.
GC13	X	12708329	C	A	FRMPD4	Missense	p.L233I	8	63	12.7%	.	.	.
GC13	X	12938648	G	T	TLR8	Missense	p.G497W	9	89	10.1%	.	.	.
GC13	X	13752241	C	A	TRAPPC2	Missense	p.W4L	6	57	10.5%	.	.	.
GC13	X	13792688	C	A	GPM6B	Missense	p.R239L	6	53	11.3%	.	.	.
GC13	X	14748374	G	T	GLRA2	Missense	p.G376W	6	79	7.6%	.	O	.
GC13	X	16881099	G	T	RBBP7	Missense	p.H140N	8	76	10.5%	.	.	.
GC13	X	18779659	G	T	PPEF1	Missense	p.G175W	7	71	9.9%	.	.	.
GC13	X	19002018	C	A	PHKA2	Missense	p.L11F	6	58	10.3%	.	.	.
GC13	X	20029138	G	T	MAP7D2	Missense	p.P616Q	6	71	8.5%	.	.	.
GC13	X	21579649	C	A	CNKSR2	Missense	p.P406Q	5	45	11.1%	.	.	.
GC13	X	21761905	G	T	SMPX	Missense	p.P32H	5	33	15.2%	.	.	.
GC13	X	21875557	G	T	YY2	Missense	p.G319W	6	62	9.7%	.	.	.
GC13	X	22003324	G	T	SMS	Missense	p.G257W	5	37	13.5%	.	.	.
GC13	X	22208560	G	T	PHEX	Splicing	c.1587-1G>T	9	74	12.2%	.	.	.
GC13	X	22291782	G	T	ZNF645	Missense	p.W225L	6	46	13.0%	.	.	.
GC13	X	23018985	G	T	DDX53	Missense	p.G271W	9	74	12.2%	.	.	.
GC13	X	23412273	C	A	PTCHD1	Missense	p.R880S	7	61	11.5%	.	.	.
GC13	X	23803490	C	A	SAT1	Missense	p.P83Q	7	93	7.5%	.	.	.
GC13	X	24330952	G	T	SUPT20HL2	Missense	p.Q161K	7	71	9.9%	.	.	.
GC13	X	24735711	C	A	POLA1	Missense	p.P308Q	7	74	9.5%	.	.	.
GC13	X	26212687	C	A	MAGEB6	Missense	p.Q242K	7	68	10.3%	.	.	.
GC13	X	27479012	G	T	PPP4R3CP	Missense	p.P801Q	6	73	8.2%	.	.	.
GC13	X	27839617	G	T	MAGEB10	Missense	p.R65L	5	49	10.2%	.	.	.
GC13	X	29973821	G	T	IL1RAPL1	Missense	p.G659W	6	80	7.5%	.	.	.
GC13	X	30254395	G	T	MAGEB3	Missense	p.L118F	6	55	10.9%	.	.	.

GC13	X	30714800	G	T	GK	Missense	p.M243I	7	75	9.3%	.	.	.
GC13	X	30849590	G	T	TAB3	Missense	p.P698Q	7	76	9.2%	.	.	.
GC13	X	31164440	C	A	DMD	Missense	p.R549L	5	60	8.3%	.	.	.
GC13	X	32305698	G	T	DMD	Missense	p.Q739K	8	75	10.7%	.	.	.
GC13	X	38271221	C	A	OTC	Missense	p.P325Q	7	73	9.6%	.	.	.
GC13	X	38525409	G	T	TSPAN7	Missense	p.W39L	5	31	16.1%	.	.	.
GC13	X	39922048	C	A	BCOR	Missense	p.R1323L	6	46	13.0%	O	.	.
GC13	X	39933726	C	A	BCOR	Missense	p.M291I	7	52	13.5%	O	.	.
GC13	X	40540115	C	A	MED14	Missense	p.L835F	7	73	9.6%	.	.	.
GC13	X	40551992	C	A	MED14	Missense	p.G605W	6	73	8.2%	.	.	.
GC13	X	41029813	C	A	USP9X	Missense	p.H990N	6	72	8.3%	.	.	.
GC13	X	41055609	G	T	USP9X	Missense	p.L1361F	7	73	9.6%	.	.	.
GC13	X	41202015	G	T	DDX3X	Missense	p.G141W	7	81	8.6%	O	.	.
GC13	X	41203050	C	A	DDX3X	Missense	p.P231Q	6	62	9.7%	O	.	.
GC13	X	41334009	G	T	NYX	Missense	p.G435W	5	27	18.5%	.	.	.
GC13	X	41495910	C	A	CASK	Missense	p.R279L	6	82	7.3%	.	.	.
GC13	X	41586364	G	T	GPR82	Missense	p.G29C	8	96	8.3%	.	.	.
GC13	X	46857529	G	T	JADE3	Missense	p.R45L	5	63	7.9%	.	.	.
GC13	X	46857622	G	T	JADE3	Missense	p.W76L	6	37	16.2%	.	.	.
GC13	X	47070489	G	T	UBA1	Missense	p.G777W	5	58	8.6%	.	.	.
GC13	X	47084248	G	T	CDK16	Missense	p.G234W	7	73	9.6%	.	.	.
GC13	X	47918759	G	T	ZNF630	Missense	p.Q358K	7	53	13.2%	.	.	.
GC13	X	48776072	C	A	PIM2	Missense	p.G14W	5	29	17.2%	.	O	.
GC13	X	49067838	C	A	CACNA1F	Missense	p.G1402C	6	38	15.8%	.	O	.
GC13	X	49143008	C	A	PPP1R3F	Missense	p.P273Q	6	39	15.4%	.	.	.
GC13	X	49645590	G	T	USP27X	Missense	p.W227L	7	76	9.2%	.	.	.
GC13	X	49645667	G	T	USP27X	Missense	p.G253W	6	69	8.7%	.	.	.
GC13	X	49806932	G	T	CLCN5	Missense	p.M8I	5	29	17.2%	.	.	.
GC13	X	51641690	C	A	MAGED1	Missense	p.L599I	6	51	11.8%	.	.	.
GC13	X	53279551	C	A	IQSEC2	Missense	p.R736L	5	36	13.9%	.	.	.
GC13	X	54020315	C	A	PHF8	Missense	p.G485W	7	44	15.9%	.	.	.

GC13	X	54569689	C	A	GNL3L	Missense	p.P147Q	7	64	10.9%	.	.	.
GC13	X	54578726	G	T	GNL3L	Missense	p.G395W	6	50	12.0%	.	.	.
GC13	X	62898230	G	T	ARHGEF9	Missense	p.Q209K	6	32	18.8%	.	.	.
GC13	X	66765613	G	T	AR	Missense	p.G209W	6	64	9.4%	O	.	.
GC13	X	67731773	G	T	YIPF6	Missense	p.R47L	6	58	10.3%	.	O	.
GC13	X	67885348	C	A	STARD8	Missense	p.P18Q	6	48	12.5%	.	.	.
GC13	X	68060283	G	T	EFNB1	Missense	p.R276L	5	46	10.9%	.	.	.
GC13	X	70464283	C	A	ZMYM3	Missense	p.R1038L	6	55	10.9%	.	.	.
GC13	X	70643066	C	A	TAF1	Missense	p.Q1538K	6	72	8.3%	.	.	.
GC13	X	70680544	C	A	TAF1	Missense	p.R1786S	6	72	8.3%	.	.	.
GC13	X	70680628	C	A	TAF1	Missense	p.H1814N	10	70	14.3%	.	.	.
GC13	X	70774372	C	A	OGT	Missense	p.L220I	7	95	7.4%	.	.	.
GC13	X	70836264	C	A	CXCR3	Missense	p.R400L	7	65	10.8%	.	.	.
GC13	X	71363366	G	T	NHSL2	Missense	p.R1207L	6	64	9.4%	.	.	.
GC13	X	71428398	C	A	ERCC6L	Missense	p.Q73H	7	75	9.3%	.	.	.
GC13	X	73962795	G	T	KIAA2022	Missense	p.R533S	6	67	9.0%	.	.	.
GC13	X	73963095	G	T	KIAA2022	Missense	p.L433M	8	71	11.3%	.	.	.
GC13	X	75397602	C	A	PBDC1	Missense	p.P150Q	7	93	7.5%	.	.	.
GC13	X	77528536	C	A	CYSLTR1	Missense	p.M236I	6	60	10.0%	.	.	.
GC13	X	84349981	C	A	SATL1	Missense	p.G572V	6	52	11.5%	.	.	.
GC13	X	84586048	G	T	POF1B	Missense	p.P254H	7	90	7.8%	.	.	.
GC13	X	88008758	G	T	CPXCR1	Missense	p.G115W	6	57	10.5%	.	O	.
GC13	X	100240761	G	T	ARL13A	Missense	p.R79L	5	46	10.9%	.	.	.
GC13	X	100603603	G	T	TIMM8A	Missense	p.P17Q	7	45	15.6%	.	O	.
GC13	X	100745877	G	T	ARMCX4	Missense	p.K767N	6	54	11.1%	.	.	.
GC13	X	100910906	G	T	ARMCX2	Missense	p.L557I	9	68	13.2%	.	.	.
GC13	X	101858503	G	T	ARMCX5	Missense	p.L478F	6	55	10.9%	.	.	.
GC13	X	101909422	G	T	GPRASP1	Missense	p.W194L	7	64	10.9%	.	.	.
GC13	X	102931507	G	T	MORF4L2	Missense	p.P150H	7	57	12.3%	.	.	.
GC13	X	106185933	G	T	MORC4	Missense	p.P730T	8	69	11.6%	.	.	.
GC13	X	107018414	C	A	TSC22D3	Missense	p.R79L	5	63	7.9%	.	.	.

GC13	X	107310202	G	T	VSIG1	Missense	p.G84W	6	61	9.8%	.	.	.
GC13	X	108718666	C	A	GUCY2F	Missense	p.R167L	6	74	8.1%	.	.	.
GC13	X	110439145	G	T	PAK3	Missense	p.G396W	6	54	11.1%	.	.	.
GC13	X	115593059	C	A	CT83	Missense	p.R64L	7	76	9.2%	.	.	.
GC13	X	118140115	G	T	LONRF3	Missense	p.G442W	6	88	6.8%	.	.	.
GC13	X	118284413	G	T	KIAA1210	Missense	p.Q44K	8	68	11.8%	.	.	.
GC13	X	119072711	G	T	NKAP	Missense	p.P150Q	6	65	9.2%	.	.	.
GC13	X	119678395	C	A	CUL4B	Missense	p.G342C	6	65	9.2%	.	.	.
GC13	X	123179142	G	T	STAG2	Missense	p.M197I	6	59	10.2%	O	.	.
GC13	X	123184159	G	T	STAG2	Missense	p.K339N	6	56	10.7%	O	.	.
GC13	X	123185062	G	T	STAG2	Missense	p.R370L	4	33	12.1%	O	.	.
GC13	X	123637458	G	T	TENM1	Missense	p.L1133I	7	91	7.7%	.	.	.
GC13	X	125955475	G	T	PRR32	Missense	p.G285V	7	78	9.0%	.	.	.
GC13	X	128878047	G	T	XPNPEP2	Missense	p.M78I	5	30	16.7%	.	.	.
GC13	X	128945385	G	T	ZDHHC9	Missense	p.P293H	9	70	12.9%	.	.	.
GC13	X	129155011	G	A	BCORL1	Missense	p.G1165R	15	52	28.8%	O	.	.
GC13	X	129185846	G	T	BCORL1	Missense	p.G1570W	7	65	10.8%	O	.	.
GC13	X	129190017	C	A	BCORL1	Missense	p.P1681Q	6	49	12.2%	O	.	.
GC13	X	129283476	G	T	AIFM1	Missense	p.P106Q	7	78	9.0%	.	.	.
GC13	X	129289202	C	A	AIFM1	Missense	p.G56W	7	49	14.3%	.	.	.
GC13	X	129318269	G	T	RAB33A	Missense	p.W90L	5	27	18.5%	.	.	.
GC13	X	129318622	C	A	RAB33A	Missense	p.L208M	7	64	10.9%	.	.	.
GC13	X	130215642	G	T	ARHGAP36	Missense	p.M1I	5	41	12.2%	.	O	.
GC13	X	131212286	G	T	FRMD7	Missense	p.P572T	7	59	11.9%	.	.	.
GC13	X	133923638	G	T	FAM122B	Missense	p.P69H	6	57	10.5%	.	.	.
GC13	X	135081125	C	A	SLC9A6	Missense	p.P264Q	8	69	11.6%	.	.	.
GC13	X	135432100	G	T	ADGRG4	Missense	p.G2079C	9	72	12.5%	.	.	.
GC13	X	135574281	G	T	BRS3	Missense	p.R316L	8	98	8.2%	.	.	.
GC13	X	135579967	G	T	HTATSF1	Missense	p.G42W	5	49	10.2%	.	.	.
GC13	X	135593348	C	A	HTATSF1	Missense	p.Q482K	6	59	10.2%	.	.	.
GC13	X	135593642	G	A	HTATSF1	Missense	p.E580K	33	74	44.6%	.	.	.

GC13	X	138845487	G	T	ATP11C	Missense	p.H831N	8	66	12.1%	.	.	.
GC13	X	140985039	G	T	MAGEC3	Missense	p.G201W	5	67	7.5%	.	.	.
GC13	X	140995340	G	T	MAGEC1	Missense	p.W717L	5	40	12.5%	.	.	.
GC13	X	140995388	G	T	MAGEC1	Missense	p.G733V	7	52	13.5%	.	.	.
GC13	X	144905207	C	A	SLITRK2	Missense	p.L422M	7	69	10.1%	.	.	.
GC13	X	148037698	G	T	AFF2	Missense	p.R349L	5	53	9.4%	.	.	.
GC13	X	152088913	G	T	ZNF185	Missense	p.R191L	4	28	14.3%	.	.	.
GC13	X	152482290	G	T	MAGEA1	Missense	p.Q241K	5	35	14.3%	.	.	.
GC13	X	153248285	G	T	TMEM187	Missense	p.G258W	8	88	9.1%	.	.	.
GC13	X	153578548	C	A	FLNA	Missense	p.R2387L	4	34	11.8%	.	.	.
GC13	X	153581708	C	A	FLNA	Missense	p.R1985L	5	43	11.6%	.	.	.
GC13	X	153716478	C	A	SLC10A3	Missense	p.G268W	5	38	13.2%	.	O	.
GC13	X	153880843	C	A	CTAG2	Missense	p.R111L	5	47	10.6%	.	.	.
GC13	X	154159778	G	T	F8	Missense	p.Q763K	7	75	9.3%	.	.	.
GC15HM	1	23966895	-	AAG	MDS2	Nonframeshift	p.V93delinsVK	105	132	79.5%	.	.	.
GC15HM	1	155004134	T	C	DCST2	Missense	p.S219G	145	150	96.7%	.	.	.
GC15HM	1	207265087	T	C	C4BPB	Missense	p.Y111H	228	237	96.2%	.	.	.
GC15HM	1	210273457	C	T	SYT14	Missense	p.S272L	44	47	93.6%	.	O	.
GC15HM	2	26696071	T	C	OTOF	Missense	p.H531R	59	62	95.2%	.	.	.
GC15HM	2	37398631	G	T	SULT6B1	Missense	p.R205S	54	55	98.2%	.	.	.
GC15HM	2	216283996	C	A	FN1	Missense	p.E596D	52	58	89.7%	.	.	.
GC15HM	3	57108066	A	G	SPATA12	Missense	p.Q115R	115	125	92.0%	.	O	.
GC15HM	4	88580628	C	G	DMP1	Missense	p.Q61E	39	40	97.5%	.	.	.
GC15HM	4	110932390	-	C	EGF	Frameshift	p.P1094fs	134	148	90.5%	.	.	.
GC15HM	4	186321550	A	G	UFSP2	Missense	p.I469T	96	103	93.2%	.	.	.
GC15HM	5	132358582	G	A	ZCCHC10	Nonsense	p.Q20X	117	120	97.5%	.	.	.
GC15HM	6	13365007	T	C	GFOD1	Missense	p.M278V	78	80	97.5%	.	.	.
GC15HM	6	100061625	-	CCG	PRDM13	Nonframeshift	p.P372delinsPA	35	56	62.5%	.	.	.
GC15HM	6	135787046	A	G	AHI1	Missense	p.F219L	130	132	98.5%	.	.	.
GC15HM	6	151936807	A	G	CCDC170	Missense	p.E647G	83	87	95.4%	.	.	.
GC15HM	7	96635565	G	C	DLX6	Missense	p.Q92H	5	71	7.0%	.	.	.

GC15HM	7	99686616	-	GGC	COPS6	Nonframeshift	p.M1delinsMG	88	141	62.4%	.	.	.
GC15HM	8	10480388	-	A	RP1L1	Frameshift	p.K108fs	39	43	90.7%	.	.	.
GC15HM	8	113277768	T	C	CSMD3	Missense	p.D3018G	87	91	95.6%	.	.	.
GC15HM	9	135171331	C	A	SETX	Missense	p.A2012S	153	161	95.0%	.	.	.
GC15HM	9	136269966	C	A	STKLD1	Missense	p.L596I	32	34	94.1%	.	.	.
GC15HM	10	50820200	G	T	SLC18A3	Missense	p.V472L	93	96	96.9%	.	.	.
GC15HM	10	135213072	G	A	MTG1	Missense	p.V154M	100	109	91.7%	.	.	.
GC15HM	11	1090908	T	C	MUC2	Missense	p.F1268S	5	83	6.0%	.	.	.
GC15HM	11	58602147	AA	-	GLYATL2	Frameshift	p.S213fs	150	158	94.9%	.	.	.
GC15HM	11	119206247	A	G	RNF26	Missense	p.I139V	87	91	95.6%	.	.	.
GC15HM	12	6972523	C	T	USP5	Missense	p.H646Y	224	232	96.6%	.	.	.
GC15HM	12	55714407	-	A	OR6C1	Frameshift	p.T8fs	199	228	87.3%	.	.	.
GC15HM	12	108986001	G	C	TMEM119	Missense	p.S53R	35	39	89.7%	.	.	.
GC15HM	13	39264083	C	T	FREM2	Missense	p.L868F	150	161	93.2%	.	.	.
GC15HM	13	101707716	-	AAC	NALCN	Nonframeshift	p.V1687delins VF	177	223	79.4%	.	.	.
GC15HM	13	103492129	G	T	BIVM	Missense	p.D254Y	146	153	95.4%	.	.	.
GC15HM	13	112723112	-	GGGCGT	SOX1	Nonframeshift	p.A380delinsA GR	81	101	80.2%	.	O	.
GC15HM	13	113909086	A	G	CUL4A	Missense	p.E611G	110	113	97.3%	.	.	.
GC15HM	14	23817146	T	C	SLC22A17	Missense	p.N82D	30	141	21.3%	.	.	.
GC15HM	14	75376389	C	A	RPS6KL1	Missense	p.G376V	84	87	96.6%	.	.	.
GC15HM	14	91931647	G	A	PPP4R3A	Missense	p.R354C	7	148	4.7%	.	.	.
GC15HM	18	55269615	G	T	NARS	Missense	p.P496H	56	57	98.2%	.	.	.
GC15HM	19	12791921	T	C	DHPS	Missense	p.I3V	5	41	12.2%	.	.	.
GC15HM	19	54872805	T	A	LAIR1	Nonsense	p.R10X	121	129	93.8%	.	.	.
GC15HM	19	56125808	A	G	ZNF865	Missense	p.K275R	32	34	94.1%	.	.	.
GC15HM	19	57803519	T	C	ZNF460	Missense	p.M496T	106	112	94.6%	.	.	.
GC15HM	20	3651955	C	G	ADAM33	Missense	p.G648A	5	89	5.6%	.	.	.
GC15HM	22	23437952	C	T	GNAZ	Missense	p.R24C	80	83	96.4%	.	.	.
GC15HM	22	32588945	A	T	RFPL2	Missense	p.L106Q	22	22	100.0%	.	.	.
GC15HM	X	25031396	T	C	ARX	Missense	p.E239G	81	85	95.3%	.	.	.
GC15HM	X	152994726	G	A	ABCD1	Missense	p.A314T	47	48	97.9%	.	.	.

GC15IM	1	23966895	-	AAG	MDS2	Nonframeshift	p.V93delinsVK	66	90	73.3%	.	.	.
GC15IM	1	29651761	C	T	PTPRU	Missense	p.R1388C	5	65	7.7%	.	.	.
GC15IM	1	155004134	T	C	DCST2	Missense	p.S219G	67	74	90.5%	.	.	.
GC15IM	1	156244456	G	C	SMG5	Missense	p.A159G	5	83	6.0%	.	.	.
GC15IM	1	207265087	T	C	C4BPB	Missense	p.Y111H	83	97	85.6%	.	.	.
GC15IM	1	210273457	C	T	SYT14	Missense	p.S272L	26	33	78.8%	.	O	.
GC15IM	2	26696071	T	C	OTOF	Missense	p.H531R	46	51	90.2%	.	.	.
GC15IM	2	37398631	G	T	SULT6B1	Missense	p.R205S	17	20	85.0%	.	.	.
GC15IM	2	131128143	C	T	PTPN18	Nonsense	p.R128X	4	43	9.3%	.	.	.
GC15IM	3	57108066	A	G	SPATA12	Missense	p.Q115R	55	65	84.6%	.	O	.
GC15IM	4	88580628	C	G	DMP1	Missense	p.Q61E	24	29	82.8%	.	.	.
GC15IM	4	110932390	-	C	EGF	Frameshift	p.P1094fs	80	96	83.3%	.	.	.
GC15IM	4	138452196	G	T	PCDH18	Missense	p.D349E	8	25	32.0%	.	.	.
GC15IM	4	186321550	A	G	UFSP2	Missense	p.I469T	29	36	80.6%	.	.	.
GC15IM	5	132358582	G	A	ZCCHC10	Nonsense	p.Q20X	43	56	76.8%	.	.	.
GC15IM	6	13365007	T	C	GFOD1	Missense	p.M278V	43	50	86.0%	.	.	.
GC15IM	6	100061625	-	CCG	PRDM13	Nonframeshift	p.P372delinsPA	38	62	61.3%	.	.	.
GC15IM	6	135787046	A	G	AHI1	Missense	p.F219L	35	48	72.9%	.	.	.
GC15IM	6	151936807	A	G	CCDC170	Missense	p.E647G	48	57	84.2%	.	.	.
GC15IM	6	152730215	G	T	SYNE1	Missense	p.D2183E	7	74	9.5%	.	.	.
GC15IM	7	99686616	-	GGC	COPS6	Nonframeshift	p.M1delinsMG	50	80	62.5%	.	.	.
GC15IM	8	10480388	-	A	RP1L1	Frameshift	p.K108fs	40	52	76.9%	.	.	.
GC15IM	8	113277768	T	C	CSMD3	Missense	p.D3018G	34	41	82.9%	.	.	.
GC15IM	9	135171331	C	A	SETX	Missense	p.A2012S	53	62	85.5%	.	.	.
GC15IM	9	136269966	C	A	STKLD1	Missense	p.L596I	22	24	91.7%	.	.	.
GC15IM	10	50820200	G	T	SLC18A3	Missense	p.V472L	44	51	86.3%	.	.	.
GC15IM	10	135213072	G	A	MTG1	Missense	p.V154M	28	39	71.8%	.	.	.
GC15IM	11	58602147	AA	-	GLYATL2	Frameshift	p.S213fs	86	108	79.6%	.	.	.
GC15IM	11	119206247	A	G	RNF26	Missense	p.I139V	44	51	86.3%	.	.	.
GC15IM	12	6972523	C	T	USP5	Missense	p.H646Y	88	99	88.9%	.	.	.
GC15IM	12	55714407	-	A	OR6C1	Frameshift	p.T8fs	102	126	81.0%	.	.	.

GC15IM	12	108986001	G	C	TMEM119	Missense	p.S53R	18	21	85.7%	.	.	.
GC15IM	13	39264083	C	T	FREM2	Missense	p.L868F	53	62	85.5%	.	.	.
GC15IM	13	101707716	-	AAC	NALCN	Nonframeshift	p.V1687delins VF	104	140	74.3%	.	.	.
GC15IM	13	103492129	G	T	BIVM	Missense	p.D254Y	55	62	88.7%	.	.	.
GC15IM	13	112723112	-	GGGCGT	SOX1	Nonframeshift	p.A380delinsA GR	26	44	59.1%	.	O	.
GC15IM	13	113909086	A	G	CUL4A	Missense	p.E611G	31	37	83.8%	.	.	.
GC15IM	14	75376389	C	A	RPS6KL1	Missense	p.G376V	58	63	92.1%	.	.	.
GC15IM	19	17449428	G	A	GTPBP3	Missense	p.E157K	5	69	7.2%	.	O	.
GC15IM	19	54872805	T	A	LAIR1	Nonsense	p.R10X	56	61	91.8%	.	.	.
GC15IM	19	57803519	T	C	ZNF460	Missense	p.M496T	51	59	86.4%	.	.	.
GC15IM	21	31852554	C	T	KRTAP19-1	Missense	p.G28E	6	62	9.7%	.	.	.
GC15IM	22	23437952	C	T	GNAZ	Missense	p.R24C	48	55	87.3%	.	.	.
GC15IM	X	25031396	T	C	ARX	Missense	p.E239G	36	40	90.0%	.	.	.
GC15IM	X	152994726	G	A	ABCD1	Missense	p.A314T	28	34	82.4%	.	.	.
GC15	1	23966895	-	AAG	MDS2	Nonframeshift	p.V93delinsV K	71	119	59.7%	.	.	.
GC15	1	155004134	T	C	DCST2	Missense	p.S219G	84	94	89.4%	.	.	.
GC15	1	207265087	T	C	C4BPB	Missense	p.Y111H	120	155	77.4%	.	.	.
GC15	1	210273457	C	T	SYT14	Missense	p.S272L	88	108	81.5%	.	O	.
GC15	2	26696071	T	C	OTOF	Missense	p.H531R	21	30	70.0%	.	.	.
GC15	2	37398631	G	T	SULT6B1	Missense	p.R205S	32	43	74.4%	.	.	.
GC15	2	97532079	C	T	SEMA4C	Missense	p.V67M	3	21	14.3%	.	O	.
GC15	2	216283996	C	A	FN1	Missense	p.E596D	17	21	81.0%	.	.	.
GC15	3	52475032	G	T	SEMA3G	Missense	p.N282K	9	43	20.9%	.	.	.
GC15	3	57108066	A	G	SPATA12	Missense	p.Q115R	57	78	73.1%	.	O	.
GC15	3	58156481	G	C	FLNB	Missense	p.V2577L	9	48	18.8%	.	.	.
GC15	3	101298579	A	T	PCNP	Missense	p.I9F	20	118	16.9%	.	.	.
GC15	3	125282643	G	T	OSBPL11	Missense	p.H305N	19	82	23.2%	.	.	.
GC15	4	88580628	C	G	DMP1	Missense	p.Q61E	37	60	61.7%	.	.	.
GC15	4	110932390	-	C	EGF	Frameshift	p.P1094fs	66	116	56.9%	.	.	.
GC15	4	186321550	A	G	UFSP2	Missense	p.I469T	47	63	74.6%	.	.	.
GC15	5	132358582	G	A	ZCCHC10	Nonsense	p.Q20X	44	67	65.7%	.	.	.

GC15	5	140589409	A	C	PCDHB12	Missense	p.E310D	31	95	32.6%	.	.	.
GC15	6	13365007	T	C	GFOD1	Missense	p.M278V	20	30	66.7%	.	.	.
GC15	6	100061625	-	CCG	PRDM13	Nonframeshift	p.P372delinsPA	7	23	30.4%	.	.	.
GC15	6	135787046	A	G	AHI1	Missense	p.F219L	56	95	58.9%	.	.	.
GC15	6	151936807	A	G	CCDC170	Missense	p.E647G	49	72	68.1%	.	.	.
GC15	7	99686616	-	GGC	COPS6	Nonframeshift	p.M1delinsMG	14	34	41.2%	.	.	.
GC15	7	149129574	G	A	ZNF777	Missense	p.R597C	3	43	7.0%	.	.	.
GC15	8	10480388	-	A	RP1L1	Frameshift	p.K108fs	13	27	48.1%	.	.	.
GC15	8	113277768	T	C	CSMD3	Missense	p.D3018G	38	62	61.3%	.	.	.
GC15	9	77377979	G	T	TRPM6	Missense	p.S1198Y	10	35	28.6%	.	.	.
GC15	9	135171331	C	A	SETX	Missense	p.A2012S	68	96	70.8%	.	.	.
GC15	9	136269966	C	A	STKLD1	Missense	p.L596I	7	20	35.0%	.	.	.
GC15	10	50820200	G	T	SLC18A3	Missense	p.V472L	25	38	65.8%	.	.	.
GC15	10	135213072	G	A	MTG1	Missense	p.V154M	17	33	51.5%	.	.	.
GC15	11	58602147	AA	-	GLYATL2	Frameshift	p.S213fs	76	116	65.5%	.	.	.
GC15	11	63398903	A	T	ATL3	Missense	p.L365Q	5	90	5.6%	.	.	.
GC15	11	66029348	C	T	KLC2	Missense	p.R122C	5	23	21.7%	.	.	.
GC15	11	66816218	G	A	SYT12	Missense	p.R304Q	4	38	10.5%	.	O	.
GC15	11	70332119	G	A	SHANK2	Missense	p.R839W	4	45	8.9%	.	.	.
GC15	11	83177774	T	-	DLG2	Frameshift	p.K261fs	34	101	33.7%	.	.	.
GC15	11	119206247	A	G	RNF26	Missense	p.I139V	34	57	59.6%	.	.	.
GC15	12	6972523	C	T	USP5	Missense	p.H646Y	74	98	75.5%	.	.	.
GC15	12	49424391	A	T	KMT2D	Missense	p.L4611H	4	73	5.5%	O	.	.
GC15	12	55714407	-	A	OR6C1	Frameshift	p.T8fs	136	172	79.1%	.	.	.
GC15	12	56862454	C	G	SPRYD4	Missense	p.Q27E	5	81	6.2%	.	.	.
GC15	12	57627860	G	A	SHMT2	Missense	p.V442I	44	102	43.1%	.	O	.
GC15	13	39264083	C	T	FREM2	Missense	p.L868F	56	74	75.7%	.	.	.
GC15	13	101707716	-	AAC	NALCN	Nonframeshift	p.V1687delinsVF	60	113	53.1%	.	.	.
GC15	13	103492129	G	T	BIVM	Missense	p.D254Y	42	71	59.2%	.	.	.
GC15	13	113909086	A	G	CUL4A	Missense	p.E611G	28	43	65.1%	.	.	.
GC15	14	75376389	C	A	RPS6KL1	Missense	p.G376V	44	58	75.9%	.	.	.

GC15	16	71487209	C	T	ZNF23	Missense	p.G27R	16	52	30.8%	.	.	.
GC15	16	86544646	G	A	FOXF1	Missense	p.M157I	6	46	13.0%	.	.	.
GC15	18	55269615	G	T	NARS	Missense	p.P496H	24	27	88.9%	.	.	.
GC15	19	41597807	G	A	CYP2A13	Missense	p.M275I	8	26	30.8%	.	.	.
GC15	19	54872805	T	A	LAIR1	Nonsense	p.R10X	39	70	55.7%	.	.	.
GC15	19	57803519	T	C	ZNF460	Missense	p.M496T	33	50	66.0%	.	.	.
GC15	22	23437952	C	T	GNAZ	Missense	p.R24C	22	37	59.5%	.	.	.
GC15	X	25031396	T	C	ARX	Missense	p.E239G	16	21	76.2%	.	.	.
GC15	X	152994726	G	A	ABCD1	Missense	p.A314T	10	20	50.0%	.	.	.
GC16	1	27100862	A	T	ARID1A	Nonsense	p.K1382X	17	45	37.8%	O	O	.
GC16	1	45253068	G	A	BEST4	Missense	p.L75F	38	98	38.8%	.	O	.
GC16	1	90398922	T	C	LRRC8D	Missense	p.S99P	62	156	39.7%	.	.	.
GC16	1	118479450	G	A	WDR3	Missense	p.G147E	50	153	32.7%	.	.	.
GC16	1	152484120	C	T	LCE5A	Missense	p.P37L	79	118	66.9%	.	.	.
GC16	1	153233489	-	GGCGGT	LOR	Nonframeshift	p.G22delinsGRC	65	172	37.8%	.	.	.
GC16	1	156704269	G	C	RRNAD1	Missense	p.E369Q	31	216	14.4%	.	.	.
GC16	1	200377711	C	G	ZNF281	Missense	p.A375P	188	292	64.4%	.	.	.
GC16	1	201184238	T	C	IGFN1	Missense	p.F2984S	5	36	13.9%	.	.	.
GC16	1	201263070	G	A	PKP1	Missense	p.G68D	66	91	72.5%	.	.	.
GC16	1	201459355	G	A	CSRP1	Missense	p.A77V	11	55	20.0%	.	.	.
GC16	1	215824059	C	T	USH2A	Missense	p.A4740T	50	82	61.0%	.	.	.
GC16	1	242035372	C	T	EXO1	Missense	p.L436F	28	141	19.9%	.	.	.
GC16	2	21364443	G	T	TDRD15	Missense	p.L1368F	28	83	33.7%	.	.	.
GC16	2	39187224	G	A	ARHGEF33	Missense	p.R593H	36	76	47.4%	.	.	.
GC16	2	74043115	C	A	C2orf78	Missense	p.Q589K	23	90	25.6%	.	.	.
GC16	2	169830314	C	T	ABCB11	Missense	p.E449K	25	53	47.2%	.	.	.
GC16	2	186658128	C	T	FSIP2	Nonsense	p.Q2089X	42	111	37.8%	.	.	.
GC16	2	201722507	C	A	CLK1	Missense	p.G298C	21	54	38.9%	.	.	.
GC16	3	44847419	A	G	KIF15	Missense	p.K638E	93	113	82.3%	.	.	.
GC16	3	50232040	AGA	-	GNAT1	Nonframeshift	p.272_273del	64	87	73.6%	.	.	.
GC16	3	52837999	G	T	ITIH3	Missense	p.R613M	21	79	26.6%	.	.	.

GC16	3	73433631	C	T	PDZRN3	Missense	p.E394K	32	79	40.5%	.	O	.
GC16	3	130683840	A	T	ATP2C1	Missense	p.N353I	58	231	25.1%	.	.	.
GC16	3	170856122	A	G	TNIK	Missense	p.L460P	50	102	49.0%	.	.	.
GC16	3	184910121	T	A	EHHADH	Missense	p.I593F	31	136	22.8%	.	.	.
GC16	4	2916652	G	C	ADD1	Missense	p.E549D	10	24	41.7%	.	.	.
GC16	4	24914468	C	T	CCDC149	Missense	p.E12K	24	63	38.1%	.	.	.
GC16	4	38933232	A	C	FAM114A1	Missense	p.E234A	33	104	31.7%	.	.	.
GC16	4	144621405	-	GCAGCA	FREM3	Nonframeshift	p.Q142delinsCCQ	40	86	46.5%	.	.	.
GC16	5	3599732	G	A	IRX1	Missense	p.D224N	15	236	6.4%	.	O	.
GC16	5	15936988	C	A	FBXL7	Missense	p.T343K	43	99	43.4%	.	.	.
GC16	5	152871789	-	C	GRIA1	Frameshift	p.L11fs	157	213	73.7%	.	.	.
GC16	5	171341391	C	T	FBXW11	Missense	p.D22N	17	43	39.5%	.	.	.
GC16	6	599147	A	G	EXOC2	Missense	p.L274P	70	150	46.7%	.	.	.
GC16	6	1612018	-	CGG	FOXC1	Nonframeshift	p.H446delinsHR	30	59	50.8%	.	.	.
GC16	6	46976856	G	T	ADGRF1	Missense	p.P772Q	40	103	38.8%	.	.	.
GC16	6	119327747	C	G	FAM184A	Missense	p.R440S	22	74	29.7%	.	.	.
GC16	7	5385419	G	C	TNRC18	Missense	p.D1831E	3	27	11.1%	.	.	.
GC16	7	11871470	-	GCAGCG	THSD7A	Nonframeshift	p.L35delinsRCL	21	38	55.3%	.	.	.
GC16	7	31697914	-	T	CCDC129	Frameshift	p.F1033fs	14	32	43.8%	.	.	.
GC16	7	100677902	A	C	MUC17	Missense	p.T1069P	12	176	6.8%	.	.	.
GC16	9	6605200	G	C	GLDC	Missense	p.F264L	52	66	78.8%	.	O	.
GC16	9	8341736	A	G	PTPRD	Missense	p.I1228T	46	164	28.0%	.	O	.
GC16	9	139634449	C	T	LCN10	Missense	p.C176Y	33	97	34.0%	.	.	.
GC16	9	140094484	G	A	TPRN	Missense	p.A227V	13	33	39.4%	.	.	.
GC16	10	3208544	T	C	PITRM1	Missense	p.S67G	19	53	35.8%	.	.	.
GC16	10	21805467	-	CCTCCT	SKIDA1	Nonframeshift	p.G429delinsRRG	34	85	40.0%	.	.	.
GC16	10	27687535	-	T	PTCHD3	Frameshift	p.K664fs	165	245	67.3%	.	.	.
GC16	10	92678964	A	G	ANKRD1	Missense	p.I90T	20	40	50.0%	.	.	.
GC16	10	101295141	G	A	NKX2-3	Missense	p.G253D	56	128	43.8%	.	.	.
GC16	11	57414511	G	A	YPEL4	Missense	p.R24C	10	36	27.8%	.	.	.
GC16	11	64802996	-	C	SNX15	Frameshift	p.D175fs	40	115	34.8%	.	.	.

GC16	11	65414220	G	C	SIPA1	Missense	p.G572A	41	83	49.4%	.	.	.
GC16	11	66838952	-	GCTCG	RHOD	Frameshift	p.C105fs	36	101	35.6%	.	.	.
GC16	11	68216518	C	T	LRP5	Missense	p.P1029S	12	25	48.0%	.	.	.
GC16	11	76954789	-	A	GDPD4	Frameshift	p.N397fs	113	161	70.2%	.	.	.
GC16	11	93103238	G	T	DEUP1	Splicing	c.433-1G>T	24	50	48.0%	.	.	.
GC16	12	6762460	G	C	ING4	Missense	p.A47G	103	124	83.1%	.	.	.
GC16	12	8374782	-	ACG	FAM90A1	Nonframeshift	p.T344delinsTS	61	76	80.3%	.	.	.
GC16	12	9098996	-	AAC	M6PR	Nonframeshift	p.F2delinsCF	24	40	60.0%	.	.	.
GC16	12	27627797	C	G	SMCO2	Missense	p.P105A	76	126	60.3%	.	.	.
GC16	12	47472230	C	T	AMIGO2	Missense	p.V186I	82	144	56.9%	.	.	.
GC16	12	49745107	G	C	DNAJC22	Missense	p.G283A	39	66	59.1%	.	.	.
GC16	12	49958600	T	C	MCRS1	Missense	p.K136E	13	39	33.3%	.	.	.
GC16	12	55714407	-	A	OR6C1	Frameshift	p.T8fs	102	194	52.6%	.	.	.
GC16	12	57884376	G	-	MARS	Frameshift	p.W240fs	25	70	35.7%	.	.	.
GC16	14	24002039	C	A	ZFHX2	Missense	p.G766C	142	160	88.8%	.	.	.
GC16	14	103999051	G	T	TRMT61A	Missense	p.R155L	10	60	16.7%	.	O	.
GC16	15	63631068	C	A	CA12	Missense	p.R215L	24	56	42.9%	.	.	.
GC16	16	50347963	A	G	ADCY7	Missense	p.H949R	31	68	45.6%	.	.	.
GC16	17	7130436	C	A	DVL2	Nonsense	p.G506X	8	25	32.0%	.	.	.
GC16	17	17701072	C	G	RAI1	Missense	p.R1604G	23	53	43.4%	.	.	.
GC16	17	36623380	G	A	ARHGAP23	Missense	p.G486S	19	41	46.3%	.	.	.
GC16	17	36872024	G	T	MLLT6	Missense	p.A327S	7	95	7.4%	.	.	.
GC16	17	38100291	C	G	LRRC3C	Missense	p.S44R	24	56	42.9%	.	.	.
GC16	17	46669675	G	A	HOXB5	Missense	p.R236C	24	63	38.1%	.	.	.
GC16	17	56281630	C	T	EPX	Missense	p.A665V	46	115	40.0%	.	.	.
GC16	17	71380097	G	T	SDK2	Nonsense	p.Y1541X	16	50	32.0%	.	.	.
GC16	18	12701000	C	G	CEP76	Missense	p.R59P	45	115	39.1%	.	.	.
GC16	18	34854378	C	T	CELF4	Missense	p.D233N	30	68	44.1%	.	.	.
GC16	18	44139442	G	A	LOXHD1	Missense	p.S1062L	18	49	36.7%	.	.	.
GC16	18	44774683	GGC	-	SKOR2	Nonframeshift	p.290_291del	39	122	32.0%	.	.	.
GC16	18	52921877	G	A	TCF4	Missense	p.R241W	42	92	45.7%	.	O	.

GC16	18	70417417	T	C	NETO1	Missense	p.N474S	42	131	32.1%	.	.	.
GC16	18	77170993	A	C	NFATC1	Missense	p.T240P	4	34	11.8%	.	.	.
GC16	19	1468360	G	T	APC2	Missense	p.R1686L	48	111	43.2%	.	.	.
GC16	19	6916313	G	A	ADGRE1	Missense	p.D311N	56	137	40.9%	.	.	.
GC16	19	8400652	G	A	KANK3	Missense	p.P20L	7	25	28.0%	.	.	.
GC16	19	13318717	G	A	CACNA1A	Missense	p.P2311S	24	57	42.1%	.	.	.
GC16	19	16178486	G	A	TPM4	Missense	p.A18T	15	42	35.7%	.	.	.
GC16	19	22271309	A	G	ZNF257	Missense	p.R177G	6	41	14.6%	.	O	.
GC16	19	49920723	T	A	CCDC155	Missense	p.W549R	5	69	7.2%	.	.	.
GC16	19	56012015	C	T	SSC5D	Nonsense	p.R821X	59	70	84.3%	.	.	.
GC16	20	43926628	G	T	MATN4	Missense	p.H421Q	26	35	74.3%	.	.	.
GC16	20	61597909	C	A	SLC17A9	Missense	p.P365Q	14	49	28.6%	.	.	.
GC16	22	24434880	G	-	CABIN1	Frameshift	p.E61fs	43	60	71.7%	.	.	.
GC16	X	49082517	C	T	CACNA1F	Missense	p.R502Q	24	89	27.0%	.	.	.
GC16	X	70321277	-	G	FOXO4	Frameshift	p.L399fs	17	43	39.5%	.	.	.
GC16	X	70469925	C	G	ZMYM3	Missense	p.G401A	8	20	40.0%	.	.	.
GC16	X	129271076	C	T	AIFM1	Nonsense	p.W12X	41	118	34.7%	.	.	.
GC16	X	129271094	G	T	AIFM1	Missense	p.P6H	41	120	34.2%	.	O	.
GC16	X	152936286	C	T	PNCK	Nonsense	p.W352X	13	41	31.7%	.	.	.
GC18	1	881898	C	A	NOC2L	Missense	p.V563L	4	40	10.0%	.	.	.
GC18	1	1916846	T	A	CFAP74	Missense	p.E160V	20	74	27.0%	.	.	.
GC18	1	8927240	C	T	ENO1	Missense	p.G34E	9	91	9.9%	.	.	.
GC18	1	12475158	G	A	VPS13D	Missense	p.G3992R	38	75	50.7%	.	.	.
GC18	1	23395062	C	A	KDM1A	Missense	p.Q380K	19	85	22.4%	.	.	.
GC18	1	23966895	-	AAG	MDS2	Nonframeshift	p.V93delinsVK	47	59	79.7%	.	.	.
GC18	1	24019172	G	A	RPL11	Missense	p.G27E	16	36	44.4%	.	.	.
GC18	1	26349835	G	A	EXTL1	Missense	p.R233K	9	55	16.4%	.	.	.
GC18	1	33235357	G	A	KIAA1522	Missense	p.G165R	9	32	28.1%	.	.	.
GC18	1	43021991	G	T	CCDC30	Missense	p.R197M	11	102	10.8%	.	.	.
GC18	1	43708934	C	T	CFAP57	Missense	p.R1181C	59	64	92.2%	.	.	.
GC18	1	43826871	-	TG	CDC20	Frameshift	p.N386fs	49	133	36.8%	.	.	.

GC18	1	43893067	C	G	SZT2	Missense	p.I1126M	57	60	95.0%	.	.	.
GC18	1	44160542	C	T	KDM4A	Missense	p.R883W	6	132	4.5%	.	.	.
GC18	1	52246838	T	A	OSBPL9	Missense	p.I242N	32	81	39.5%	.	.	.
GC18	1	55474249	G	A	BSND	Missense	p.G304E	9	120	7.5%	.	.	.
GC18	1	57170000	G	T	PRKAA2	Missense	p.C382F	9	178	5.1%	.	.	.
GC18	1	103491363	C	T	COL11A1	Missense	p.G309E	28	111	25.2%	.	.	.
GC18	1	110125127	C	G	GNAI3	Missense	p.T177R	27	64	42.2%	.	.	.
GC18	1	111061319	C	G	KCNA10	Missense	p.D31H	20	57	35.1%	.	.	.
GC18	1	111216219	G	T	KCNA3	Missense	p.L405I	8	86	9.3%	.	.	.
GC18	1	111494065	C	T	LRIF1	Missense	p.G481R	10	80	12.5%	.	.	.
GC18	1	114354752	C	T	RSBN1	Missense	p.G95R	12	61	19.7%	.	.	.
GC18	1	114399237	T	A	PTPN22	Missense	p.K138M	19	159	11.9%	.	.	.
GC18	1	114483547	A	T	HIPK1	Missense	p.E181V	7	93	7.5%	.	.	.
GC18	1	117552513	G	A	CD101	Missense	p.G29R	9	88	10.2%	.	.	.
GC18	1	120468054	G	T	NOTCH2	Missense	p.P1462H	39	106	36.8%	O	.	.
GC18	1	154987396	-	GT	ZBTB7B	Frameshift	p.G87fs	19	52	36.5%	.	.	.
GC18	1	156046507	T	A	MEX3A	Missense	p.E474V	12	61	19.7%	.	.	.
GC18	1	156288671	T	A	CCT3	Missense	p.K211N	6	78	7.7%	.	.	.
GC18	1	162335338	A	C	NOS1AP	Missense	p.K67Q	5	74	6.8%	.	.	.
GC18	1	168105582	-	G	GPR161	Frameshift	p.P18fs	57	65	87.7%	.	.	.
GC18	1	169364364	G	A	CCDC181	Missense	p.A484V	42	124	33.9%	.	.	.
GC18	1	169511791	C	A	F5	Missense	p.G846V	9	145	6.2%	.	.	.
GC18	1	181701916	G	T	CACNA1E	Missense	p.E879D	5	64	7.8%	.	.	.
GC18	1	197070411	C	T	ASPM	Missense	p.R2657K	6	94	6.4%	.	.	.
GC18	1	200823989	A	T	CAMSAP2	Missense	p.S1274C	7	95	7.4%	.	.	.
GC18	1	202711581	G	A	KDM5B	Missense	p.R798W	48	87	55.2%	.	O	.
GC18	1	203452702	G	T	PRELP	Missense	p.W130C	8	114	7.0%	.	.	.
GC18	1	203452703	A	T	PRELP	Missense	p.I131F	8	114	7.0%	.	.	.
GC18	1	211952387	C	T	LPGAT1	Splicing	c.728-1G>A	5	36	13.9%	.	.	.
GC18	1	215802305	G	C	USH2A	Missense	p.L5124V	10	84	11.9%	.	.	.
GC18	1	233134980	A	T	PCNX2	Missense	p.L1825Q	15	62	24.2%	.	.	.

GC18	1	233134981	G	T	PCNX2	Missense	p.L1825M	15	64	23.4%	.	.	.
GC18	2	21230321	G	T	APOB	Missense	p.T3140K	5	73	6.8%	.	.	.
GC18	2	27560866	C	T	GTF3C2	Missense	p.G293R	6	92	6.5%	.	.	.
GC18	2	31560582	C	A	XDH	Missense	p.K1292N	12	56	21.4%	.	.	.
GC18	2	54850707	G	T	SPTBN1	Missense	p.G373W	7	97	7.2%	.	.	.
GC18	2	60679787	G	A	BCL11A	Missense	p.P749S	6	66	9.1%	.	.	.
GC18	2	61019312	A	T	PAPOLG	Nonsense	p.R523X	13	73	17.8%	.	.	.
GC18	2	70188180	C	T	ASPRV1	Missense	p.G214E	8	103	7.8%	.	.	.
GC18	2	80874862	G	T	CTNNA2	Missense	p.K540N	6	87	6.9%	.	O	.
GC18	2	97020090	G	A	NCAPH	Missense	p.R255K	11	49	22.4%	.	.	.
GC18	2	130832581	T	C	POTEF	Missense	p.I822V	4	21	19.0%	.	.	.
GC18	2	141609232	T	C	LRP1B	Missense	p.K1567R	21	37	56.8%	O	.	.
GC18	2	152320382	C	T	RIF1	Missense	p.P1450S	81	87	93.1%	.	.	.
GC18	2	152321135	G	T	RIF1	Missense	p.G1701W	54	63	85.7%	.	O	.
GC18	2	167129122	C	T	SCN9A	Missense	p.M1035I	23	114	20.2%	.	.	.
GC18	2	167168134	C	T	SCN9A	Missense	p.E45K	15	86	17.4%	.	.	.
GC18	2	179592981	T	A	TTN	Missense	p.S5280C	5	60	8.3%	.	.	.
GC18	2	186653493	C	T	FSIP2	Missense	p.P544S	8	82	9.8%	.	.	.
GC18	2	196771388	G	T	DNAH7	Missense	p.L1444M	5	53	9.4%	.	.	.
GC18	2	206872088	T	G	INO80D	Missense	p.D613A	6	85	7.1%	.	.	.
GC18	2	210642089	G	T	UNC80	Missense	p.D136Y	9	27	33.3%	.	.	.
GC18	2	219544797	C	A	STK36	Missense	p.A377E	16	84	19.0%	.	.	.
GC18	2	227661440	G	A	IRS1	Missense	p.S672F	4	38	10.5%	.	.	.
GC18	2	227942792	C	T	COL4A4	Missense	p.G602E	6	62	9.7%	.	O	.
GC18	2	231042927	G	A	SP110	Missense	p.P465S	5	44	11.4%	.	.	.
GC18	3	1367563	G	T	CNTN6	Missense	p.W233C	12	125	9.6%	.	.	.
GC18	3	38012946	G	A	CTDSPL	Missense	p.G148R	7	83	8.4%	.	.	.
GC18	3	38888955	T	G	SCN11A	Missense	p.T1536P	9	88	10.2%	.	.	.
GC18	3	39116406	G	T	WDR48	Missense	p.V206L	22	87	25.3%	.	.	.
GC18	3	42735176	C	A	HHATL	Missense	p.C394F	19	89	21.3%	.	.	.
GC18	3	44670740	G	T	ZNF197	Nonsense	p.G32X	6	62	9.7%	.	.	.

GC18	3	47382106	G	A	KLHL18	Missense	p.G389E	5	86	5.8%	.	.	.
GC18	3	49662725	C	A	BSN	Missense	p.T181N	12	165	7.3%	.	.	.
GC18	3	51315088	A	G	DOCK3	Missense	p.D909G	7	51	13.7%	.	O	.
GC18	3	52256340	G	T	TLR9	Nonsense	p.Y664X	25	63	39.7%	.	.	.
GC18	3	53531262	C	A	CACNA1D	Missense	p.L51M	6	84	7.1%	O	.	.
GC18	3	53531263	T	C	CACNA1D	Missense	p.L51P	6	86	7.0%	O	.	.
GC18	3	53769523	G	A	CACNA1D	Missense	p.R915Q	4	36	11.1%	O	O	.
GC18	3	53911248	T	A	ACTR8	Missense	p.D226V	12	35	34.3%	.	.	.
GC18	3	57132056	A	C	IL17RD	Missense	p.W559G	15	106	14.2%	.	.	.
GC18	3	72897347	G	A	SHQ1	Splicing	c.143+2C>T	8	90	8.9%	.	.	.
GC18	3	100413682	G	A	ADGRG7	Missense	p.R449K	29	136	21.3%	.	.	.
GC18	3	101284469	T	C	TRMT10C	Missense	p.F282L	25	225	11.1%	.	.	.
GC18	3	115738395	G	T	LSAMP	Missense	p.P161T	24	98	24.5%	.	.	.
GC18	3	122414437	G	A	PARP14	Missense	p.G255R	5	68	7.4%	.	.	.
GC18	3	122631061	G	T	SEMA5B	Missense	p.L858M	7	58	12.1%	.	.	.
GC18	3	124732420	-	GAAGAG	HEG1	Nonframeshift	p.S668delinsS LP	158	390	40.5%	.	.	.
GC18	3	126291065	G	T	TXNRD3NB	Missense	p.P108T	5	73	6.8%	.	.	.
GC18	3	129695755	C	T	TRH	Missense	p.P142L	7	86	8.1%	.	.	.
GC18	3	135978057	T	C	PCCB	Missense	p.S138P	7	111	6.3%	.	.	.
GC18	3	170736319	C	A	SLC2A2	Splicing	c.14+1G>T;N M_001278659	7	113	6.2%	.	.	.
GC18	3	170736320	C	G	SLC2A2	Missense	p.Q36H	6	111	5.4%	.	.	.
GC18	4	1816277	T	A	LETM1	Missense	p.E698D	6	54	11.1%	.	.	.
GC18	4	6087293	C	A	JAKMIP1	Missense	p.A65S	7	59	11.9%	.	.	.
GC18	4	17826621	C	A	NCAPG	Missense	p.P472T	10	72	13.9%	.	.	.
GC18	4	40440136	C	A	RBM47	Nonsense	p.E259X	6	76	7.9%	.	.	.
GC18	4	71232514	C	A	SMR3A	Missense	p.P70T	17	219	7.8%	.	.	.
GC18	4	71389610	C	A	AMTN	Missense	p.P54Q	18	61	29.5%	.	.	.
GC18	4	74364860	C	T	AFM	Missense	p.P440L	6	101	5.9%	.	.	.
GC18	4	81791190	T	C	C4orf22	Missense	p.I126T	40	44	90.9%	.	.	.
GC18	4	126336573	C	T	FAT4	Missense	p.P2152L	13	122	10.7%	O	.	.
GC18	4	146823779	G	C	ZNF827	Missense	p.A211G	7	63	11.1%	.	.	.

GC18	4	148787962	C	G	ARHGAP10	Missense	p.Q233E	19	21	90.5%	.	.	.
GC18	5	5464830	G	A	ICE1	Missense	p.G1795R	6	81	7.4%	.	.	.
GC18	5	36257119	G	T	RANBP3L	Missense	p.S252Y	5	79	6.3%	.	.	.
GC18	5	52204858	A	G	ITGA1	Missense	p.Y529C	28	72	38.9%	.	.	.
GC18	5	73930860	T	G	ENC1	Missense	p.Y411S	92	98	93.9%	.	.	.
GC18	5	76249468	G	A	CRHBP	Missense	p.A42T	12	34	35.3%	.	O	.
GC18	5	89979890	C	A	ADGRV1	Missense	p.A2051D	15	42	35.7%	.	.	.
GC18	5	90074313	G	A	ADGRV1	Missense	p.G4246R	34	113	30.1%	.	.	.
GC18	5	115298697	C	A	LVRN	Missense	p.T128N	8	56	14.3%	.	.	.
GC18	5	128796157	T	G	ADAMTS19	Missense	p.S25A	8	102	7.8%	.	.	.
GC18	5	140166399	G	T	PCDHA1	Missense	p.S175I	5	78	6.4%	.	.	.
GC18	5	148596547	C	A	ABLIM3	Missense	p.T232N	27	80	33.8%	.	.	.
GC18	5	148930510	GCC	-	CSNK1A1	Nonframeshift	p.6_6del	61	141	43.3%	.	.	.
GC18	5	153054186	C	G	GRIA1	Missense	p.R196G	5	64	7.8%	.	.	.
GC18	5	153085398	C	A	GRIA1	Missense	p.L452I	33	92	35.9%	.	.	.
GC18	5	156747701	A	T	CYFIP2	Missense	p.E495V	5	74	6.8%	.	.	.
GC18	5	159781793	C	T	C1QTNF2	Missense	p.G121R	5	26	19.2%	.	.	.
GC18	5	170308860	C	G	RANBP17	Missense	p.S72R	6	53	11.3%	.	.	.
GC18	5	172353510	A	G	ERGIC1	Splicing	c.481-2A>G	11	132	8.3%	.	.	.
GC18	5	180651649	G	A	TRIM41	Missense	p.R217Q	5	72	6.9%	.	.	.
GC18	6	12161874	G	T	HIVEP1	Missense	p.E2230D	73	80	91.3%	.	.	.
GC18	6	17102510	C	A	STMND1	Missense	p.P8T	12	77	15.6%	.	.	.
GC18	6	27277594	G	A	POM121L2	Missense	p.P786S	16	86	18.6%	.	.	.
GC18	6	29274626	C	T	OR14J1	Missense	p.H54Y	21	194	10.8%	.	.	.
GC18	6	30918925	T	C	DPCR1	Missense	p.L895P	6	92	6.5%	.	.	.
GC18	6	30955864	G	A	MUC21	Missense	p.G532R	6	49	12.2%	.	.	.
GC18	6	31000117	C	A	MUC22	Missense	p.A1605E	8	106	7.5%	.	.	.
GC18	6	31127278	G	A	TCF19	Missense	p.G11E	4	29	13.8%	.	.	.
GC18	6	31735401	G	T	VWA7	Missense	p.A545E	5	76	6.6%	.	.	.
GC18	6	32944054	A	C	BRD2	Missense	p.H213P	11	158	7.0%	.	.	.
GC18	6	43041692	G	T	KLC4	Missense	p.A523S	30	134	22.4%	.	.	.

GC18	6	43415665	G	A	ABCC10	Missense	p.A1289T	4	37	10.8%	.	O	.
GC18	6	49427119	C	A	MUT	Nonsense	p.E21X	27	48	56.3%	.	.	.
GC18	6	54002657	C	A	MLIP	Missense	p.P586H	36	119	30.3%	.	.	.
GC18	6	64422249	A	G	PHF3	Missense	p.K1589E	35	37	94.6%	.	.	.
GC18	6	83748163	C	A	UBE3D	Missense	p.K26N	7	45	15.6%	.	.	.
GC18	6	96971098	G	A	UFL1	Missense	p.D52N	5	92	5.4%	.	.	.
GC18	6	134349608	T	A	SLC2A12	Missense	p.D452V	28	116	24.1%	.	.	.
GC18	6	134349609	C	G	SLC2A12	Missense	p.D452H	28	116	24.1%	.	.	.
GC18	6	144086512	G	A	PHACTR2	Missense	p.G190E	7	85	8.2%	.	.	.
GC18	6	150001123	T	A	LATS1	Missense	p.R547S	7	92	7.6%	.	O	.
GC18	6	150174199	G	C	LRP11	Missense	p.D237E	4	25	16.0%	.	.	.
GC18	6	152646301	C	T	SYNE1	Nonsense	p.W5121X	10	94	10.6%	.	.	.
GC18	7	883016	G	A	SUN1	Missense	p.G173R	18	188	9.6%	.	.	.
GC18	7	1526583	T	A	INTS1	Missense	p.K934M	12	101	11.9%	.	.	.
GC18	7	5385278	G	C	TNRC18	Missense	p.S1878R	10	66	15.2%	.	.	.
GC18	7	19765385	G	T	TMEM196	Missense	p.L71I	8	49	16.3%	.	.	.
GC18	7	21469619	G	A	SP4	Missense	p.G262E	22	172	12.8%	.	.	.
GC18	7	44714808	A	T	OGDH	Missense	p.R323W	11	109	10.1%	.	.	.
GC18	7	45124058	G	T	NACAD	Missense	p.P574H	12	94	12.8%	.	.	.
GC18	7	48237852	A	T	ABCA13	Missense	p.D61V	8	193	4.1%	.	.	.
GC18	7	49842337	G	T	VWC2	Missense	p.A243S	20	231	8.7%	.	.	.
GC18	7	65425922	-	CTA	GUSB	Nonsense	p.A494delinsX	102	156	65.4%	.	.	.
GC18	7	75601730	G	A	POR	Splicing	c.189-1G>A	7	79	8.9%	.	.	.
GC18	7	81350078	G	C	HGF	Missense	p.N418K	26	56	46.4%	.	.	.
GC18	7	81372668	C	A	HGF	Splicing	c.850+1G>T;NM_001010933	10	76	13.2%	.	.	.
GC18	7	82583467	C	A	PCLO	Missense	p.D2268Y	7	69	10.1%	.	.	.
GC18	7	82584024	G	T	PCLO	Missense	p.P2082Q	19	94	20.2%	.	.	.
GC18	7	96653794	A	T	DLX5	Missense	p.Y48N	27	76	35.5%	.	.	.
GC18	7	100683295	A	T	MUC17	Missense	p.E2866D	13	106	12.3%	.	.	.
GC18	7	105189109	G	T	RINT1	Missense	p.R238S	31	113	27.4%	.	.	.
GC18	7	116146092	G	T	CAV2	Missense	p.V53L	132	143	92.3%	.	.	.

GC18	7	130021990	C	A	CPA1	Missense	p.H141Q	44	103	42.7%	.	.	.
GC18	7	143632509	T	A	OR2F2	Missense	p.F62I	8	122	6.6%	.	.	.
GC18	7	151680215	G	T	GALNTL5	Missense	p.L171F	117	286	40.9%	.	.	.
GC18	8	1817393	G	A	ARHGEF10	Missense	p.R220K	23	97	23.7%	.	.	.
GC18	8	3038694	A	T	CSMD1	Missense	p.L1888H	12	41	29.3%	.	.	.
GC18	8	10467496	C	T	RP1L1	Missense	p.G1371E	47	429	11.0%	.	.	.
GC18	8	35579796	C	A	UNC5D	Missense	p.L391M	6	45	13.3%	.	.	.
GC18	8	35579797	T	C	UNC5D	Missense	p.L391P	6	46	13.0%	.	.	.
GC18	8	56711766	G	T	TGS1	Missense	p.Q519H	5	30	16.7%	.	.	.
GC18	8	56711767	G	T	TGS1	Missense	p.A520S	5	30	16.7%	.	.	.
GC18	8	69129930	G	T	PREX2	Missense	p.A1562S	8	136	5.9%	O	.	.
GC18	8	103572961	C	T	ODF1	Missense	p.P201L	20	188	10.6%	.	.	.
GC18	8	107782266	C	A	ABRA	Missense	p.W51C	9	114	7.9%	.	.	.
GC18	8	144663438	T	G	EEF1D	Missense	p.I60L	6	67	9.0%	.	.	.
GC18	8	145001607	C	A	PLEC	Missense	p.A1229S	5	49	10.2%	.	.	.
GC18	9	15666182	G	A	CCDC171	Missense	p.G313R	29	84	34.5%	.	.	.
GC18	9	17332663	G	A	CNTLN	Missense	p.E527K	6	51	11.8%	.	.	.
GC18	9	19290785	G	A	DENND4C	Missense	p.G238R	6	98	6.1%	.	.	.
GC18	9	21077381	G	T	IFNB1	Missense	p.A163D	12	154	7.8%	.	O	.
GC18	9	98278952	C	T	PTCH1	Missense	p.G51R	8	65	12.3%	O	O	.
GC18	9	99006652	C	A	HSD17B3	Missense	p.A211S	18	144	12.5%	.	.	.
GC18	9	100892082	C	A	CORO2A	Missense	p.G321W	11	231	4.8%	.	.	.
GC18	9	107568584	C	T	ABCA1	Missense	p.D1468N	4	37	10.8%	.	O	.
GC18	9	112705052	G	A	PALM2	Missense	p.G163R	7	97	7.2%	.	.	.
GC18	9	113163241	C	G	SVEP1	Missense	p.V3239L	86	95	90.5%	.	.	.
GC18	9	115759577	G	C	ZNF883	Nonsense	p.Y321X	8	167	4.8%	.	.	.
GC18	9	133570917	C	T	EXOSC2	Missense	p.A54V	36	164	22.0%	.	.	.
GC18	9	139910496	G	A	ABCA2	Missense	p.R1079W	4	36	11.1%	.	.	.
GC18	10	15713602	C	A	ITGA8	Nonsense	p.E283X	6	67	9.0%	.	.	.
GC18	10	25887494	G	T	GPR158	Missense	p.R980M	9	86	10.5%	.	.	.
GC18	10	71124614	G	A	HK1	Missense	p.G151R	29	93	31.2%	.	.	.

GC18	10	103354478	A	T	DPCD	Missense	p.K43M	12	129	9.3%	.	.	.
GC18	10	120070379	C	A	FAM204A	Missense	p.G231V	6	72	8.3%	.	.	.
GC18	10	124399626	G	A	DMBT1	Missense	p.R1581K	7	102	6.9%	.	.	.
GC18	10	125506423	C	T	CPXM2	Missense	p.G710R	5	95	5.3%	.	.	.
GC18	10	134649649	C	T	CFAP46	Missense	p.G2204R	6	57	10.5%	.	.	.
GC18	11	1888077	C	T	LSP1	Missense	p.P125S	6	23	26.1%	.	.	.
GC18	11	4104509	G	T	STIM1	Missense	p.V419L	4	23	17.4%	.	.	.
GC18	11	5255309	A	G	HBD	Missense	p.L76P	7	72	9.7%	.	.	.
GC18	11	6129504	C	A	OR56B4	Missense	p.P166T	23	121	19.0%	.	.	.
GC18	11	6417350	G	T	APBB1	Missense	p.L370I	8	62	12.9%	.	.	.
GC18	11	7981505	T	C	NLRP10	Missense	p.M552V	45	51	88.2%	.	.	.
GC18	11	8670087	C	T	TRIM66	Missense	p.G56S	75	86	87.2%	.	.	.
GC18	11	46637237	C	T	HARBI1	Missense	p.G184E	6	91	6.6%	.	.	.
GC18	11	56114370	C	A	OR8K1	Missense	p.L286M	7	104	6.7%	.	O	.
GC18	11	59271094	G	T	OR4D11	Nonsense	p.G16X	5	60	8.3%	.	.	.
GC18	11	63884665	T	C	FLRT1	Missense	p.L309P	6	87	6.9%	.	.	.
GC18	11	65481249	G	A	KAT5	Missense	p.G155E	11	97	11.3%	.	.	.
GC18	11	66833398	T	C	RHOD	Missense	p.M55T	57	59	96.6%	.	.	.
GC18	11	92532579	C	A	FAT3	Missense	p.P2134T	10	80	12.5%	.	.	.
GC18	11	100863137	C	T	TMEM133	Missense	p.P33L	7	122	5.7%	.	.	.
GC18	11	102195412	A	C	BIRC3	Missense	p.T58P	9	151	6.0%	O	.	.
GC18	11	104763134	A	T	CASP12	Missense	p.L119H	11	75	14.7%	.	.	.
GC18	11	117301554	G	T	DSCAML1	Missense	p.S1917Y	27	112	24.1%	.	.	.
GC18	11	120769316	T	A	GRIK4	Missense	p.F414I	7	105	6.7%	.	.	.
GC18	11	128773360	C	G	C11orf45	Missense	p.W61C	10	72	13.9%	.	.	.
GC18	12	4919536	T	C	KCNA6	Missense	p.V110A	4	34	11.8%	.	.	.
GC18	12	8374782	-	ACG	FAM90A1	Nonframeshift	p.T344delinsTS	28	36	77.8%	.	.	.
GC18	12	9303282	A	T	PZP	Missense	p.L1448M	34	100	34.0%	.	.	.
GC18	12	12419665	C	T	LRP6	Missense	p.G2E	4	37	10.8%	.	.	.
GC18	12	14018784	A	T	GRIN2B	Missense	p.L120H	17	50	34.0%	.	O	.
GC18	12	21807559	T	A	LDHB	Missense	p.E16V	21	55	38.2%	.	.	.

GC18	12	23999091	G	A	SOX5	Missense	p.P93S	6	38	15.8%	.	.	.
GC18	12	32974325	C	T	PKP2	Missense	p.G660R	8	109	7.3%	.	.	.
GC18	12	41337514	C	T	CNTN1	Missense	p.L488F	5	44	11.4%	.	.	.
GC18	12	46246192	G	A	ARID2	Missense	p.S1429N	8	93	8.6%	O	.	.
GC18	12	56600529	G	A	RNF41	Missense	p.S219L	21	63	33.3%	.	.	.
GC18	12	57849479	G	A	INHBE	Missense	p.G54R	7	45	15.6%	.	.	.
GC18	12	58140853	A	G	TSPAN31	Missense	p.K83R	44	55	80.0%	.	.	.
GC18	12	60098635	G	A	SLC16A7	Nonsense	p.W18X	6	34	17.6%	.	.	.
GC18	12	62784759	A	T	USP15	Missense	p.S405C	18	39	46.2%	.	.	.
GC18	12	63543712	A	C	AVPR1A	Missense	p.V302G	12	203	5.9%	.	.	.
GC18	12	95914934	C	T	USP44	Missense	p.G593D	9	139	6.5%	.	.	.
GC18	12	104063352	G	C	STAB2	Missense	p.D736H	45	52	86.5%	.	.	.
GC18	12	108145734	T	A	PRDM4	Missense	p.E195V	13	154	8.4%	.	.	.
GC18	12	112148161	A	G	ACAD10	Missense	p.R259G	12	116	10.3%	.	.	.
GC18	12	112186280	G	T	ACAD10	Splicing	c.2737+1G>T	23	24	95.8%	.	.	.
GC18	12	112187086	G	T	ACAD10	Missense	p.R918S	4	38	10.5%	.	.	.
GC18	12	118610419	C	T	TAOK3	Missense	p.R121Q	14	50	28.0%	.	O	.
GC18	12	119909830	G	A	CCDC60	Missense	p.G68R	26	33	78.8%	.	O	.
GC18	12	122361604	T	C	WDR66	Missense	p.L152P	6	67	9.0%	.	.	.
GC18	13	32366944	C	A	RXFP2	Missense	p.A478D	11	125	8.8%	.	O	.
GC18	13	40261859	T	A	COG6	Missense	p.M311K	10	65	15.4%	.	.	.
GC18	13	52952180	C	T	THSD1	Missense	p.R589K	7	97	7.2%	.	.	.
GC18	13	95114417	G	T	DCT	Missense	p.T297N	12	92	13.0%	.	.	.
GC18	13	103400933	G	T	CCDC168	Missense	p.P705H	9	78	11.5%	.	.	.
GC18	13	108882192	G	A	ABHD13	Missense	p.R209K	6	74	8.1%	.	.	.
GC18	14	21250270	C	T	RNASE6	Missense	p.P138S	9	158	5.7%	.	.	.
GC18	14	58583144	A	G	C14orf37	Missense	p.V638A	7	47	14.9%	.	.	.
GC18	14	75514563	C	T	MLH3	Missense	p.G599E	9	105	8.6%	.	.	.
GC18	14	92537355	-	CTGCTGCTG CTGCTG	ATXN3	Nonframeshift	p.G23delinsQ QQQQG	69	150	46.0%	.	.	.
GC18	14	102452229	G	A	DYNC1H1	Missense	p.R556K	11	124	8.9%	.	.	.
GC18	15	25924786	G	T	ATP10A	Missense	p.A1401D	9	75	12.0%	.	.	.

GC18	15	25959194	C	A	ATP10A	Missense	p.R657S	10	34	29.4%	.	.	.
GC18	15	50555577	T	A	HDC	Missense	p.Q20L	25	141	17.7%	.	.	.
GC18	15	52662569	C	A	MYO5A	Nonsense	p.G955X	7	85	8.2%	.	.	.
GC18	15	53997219	T	G	WDR72	Missense	p.K438N	45	107	42.1%	.	.	.
GC18	15	55919223	T	C	PRTG	Missense	p.I970M	102	111	91.9%	.	.	.
GC18	15	65994132	G	A	DENND4A	Missense	p.P843S	5	50	10.0%	.	.	.
GC18	15	102346330	G	T	OR4F6	Missense	p.M136I	12	127	9.4%	.	.	.
GC18	16	11001549	C	T	CIITA	Missense	p.P734S	6	71	8.5%	.	.	.
GC18	16	21216790	C	T	ZP2	Missense	p.R215K	5	30	16.7%	.	.	.
GC18	16	22111573	C	T	VWA3A	Missense	p.S95L	13	49	26.5%	.	.	.
GC18	16	23223356	T	A	SCNN1G	Missense	p.I393N	8	115	7.0%	.	.	.
GC18	16	29851543	A	T	MVP	Missense	p.Q318H	13	48	27.1%	.	.	.
GC18	16	30583494	G	A	ZNF688	Missense	p.L35F	5	60	8.3%	.	O	.
GC18	16	31470281	G	A	ARMC5	Missense	p.R71Q	24	26	92.3%	.	.	.
GC18	16	55516973	C	G	MMP2	Missense	p.C52W	10	93	10.8%	.	.	.
GC18	16	57559999	T	C	CCDC102A	Missense	p.E209G	4	31	12.9%	.	.	.
GC18	16	58018245	C	T	TEPP	Missense	p.P143S	6	123	4.9%	.	.	.
GC18	16	58296376	A	T	CCDC113	Missense	p.R185W	6	69	8.7%	.	.	.
GC18	16	66802033	T	C	TERB1	Missense	p.N553S	5	53	9.4%	.	.	.
GC18	16	66998349	G	T	CES3	Missense	p.G217V	5	79	6.3%	.	.	.
GC18	16	67354563	A	T	KCTD19	Missense	p.F77I	13	117	11.1%	.	.	.
GC18	16	76556101	C	T	CNTNAP4	Missense	p.P772L	4	36	11.1%	.	O	.
GC18	17	2203129	C	A	SMG6	Missense	p.E306D	7	111	6.3%	.	.	.
GC18	17	5058842	A	T	USP6	Missense	p.Q923H	9	96	9.4%	.	.	.
GC18	17	5462963	C	T	NLRP1	Nonsense	p.W351X	12	89	13.5%	.	.	.
GC18	17	6610457	C	A	SLC13A5	Missense	p.V41F	7	93	7.5%	.	.	.
GC18	17	7232438	T	A	NEURL4	Missense	p.Q65L	5	39	12.8%	.	.	.
GC18	17	7534054	G	A	SHBG	Missense	p.G87E	6	90	6.7%	.	.	.
GC18	17	7726904	C	A	DNAH2	Missense	p.L3763M	6	94	6.4%	.	.	.
GC18	17	7945706	G	C	ALOX15B	Missense	p.G157R	6	69	8.7%	.	.	.
GC18	17	10728632	C	A	PIRT	Missense	p.V111L	9	133	6.8%	.	.	.

GC18	17	27030630	-	G	PROCA1	Frameshift	p.A203fs	127	158	80.4%	.	.	.
GC18	17	27613264	C	T	NUFIP2	Missense	p.G583E	12	114	10.5%	.	.	.
GC18	17	37649072	A	T	CDK12	Missense	p.K726M	20	66	30.3%	O	.	.
GC18	17	37649073	G	T	CDK12	Missense	p.K726N	19	64	29.7%	O	.	.
GC18	17	39723973	A	T	KRT9	Missense	p.L475H	8	98	8.2%	.	.	.
GC18	17	40842226	G	T	CNTNAP1	Splicing	c.1855+1G>T	85	94	90.4%	.	.	.
GC18	17	42289110	C	T	UBTF	Missense	p.S267N	7	45	15.6%	.	.	.
GC18	17	43333169	C	T	SPATA32	Nonsense	p.W127X	43	100	43.0%	.	.	.
GC18	17	45821876	C	T	TBX21	Missense	p.P319L	11	59	18.6%	.	.	.
GC18	17	48557408	G	A	RSAD1	Missense	p.G146E	7	68	10.3%	.	.	.
GC18	17	48701807	C	T	CACNA1G	Missense	p.H1979Y	5	47	10.6%	.	.	.
GC18	17	55026747	C	A	COIL	Splicing	c.1488+1G>T	10	48	20.8%	.	.	.
GC18	17	66985239	C	T	ABCA9	Missense	p.G1326R	14	66	21.2%	.	.	.
GC18	17	79826767	C	T	ARHGDIA	Missense	p.R163K	4	44	9.1%	.	.	.
GC18	17	80320376	C	T	TEX19	Missense	p.P117L	6	46	13.0%	.	.	.
GC18	18	2697869	A	G	SMCHD1	Missense	p.N391S	8	97	8.2%	.	.	.
GC18	18	2769723	G	A	SMCHD1	Missense	p.R1584K	10	95	10.5%	.	.	.
GC18	18	11886777	C	T	MPPE1	Missense	p.A227T	6	69	8.7%	.	.	.
GC18	18	21451479	A	T	LAMA3	Missense	p.I1618F	66	73	90.4%	.	.	.
GC18	18	46784808	C	T	DYM	Missense	p.G436E	6	81	7.4%	.	.	.
GC18	18	52546651	C	A	RAB27B	Missense	p.H69N	10	132	7.6%	.	.	.
GC18	18	63548105	C	A	CDH7	Missense	p.T778N	14	37	37.8%	.	.	.
GC18	19	6396460	G	-	LOC390877	Frameshift	p.V24fs	55	71	77.5%	.	.	.
GC18	19	8841866	A	G	OR2Z1	Missense	p.Q159R	29	99	29.3%	.	.	.
GC18	19	9237444	G	T	OR7G3	Missense	p.F61L	102	120	85.0%	.	.	.
GC18	19	9361978	G	C	OR7E24	Missense	p.A87P	4	37	10.8%	.	O	.
GC18	19	13419318	C	A	CACNA1A	Missense	p.V566F	7	75	9.3%	.	.	.
GC18	19	13934229	C	A	ZSWIM4	Missense	p.S593R	19	70	27.1%	.	.	.
GC18	19	18546423	C	T	ISYNA1	Missense	p.E274K	29	115	25.2%	.	.	.
GC18	19	36122308	G	T	RBM42	Splicing	c.442+1G>T	16	68	23.5%	.	.	.
GC18	19	36245360	G	T	LIN37	Missense	p.M242I	5	90	5.6%	.	.	.

GC18	19	43922449	C	T	TEX101	Missense	p.P217L	7	80	8.8%	.	.	.
GC18	19	44570228	C	T	ZNF223	Nonsense	p.Q83X	20	83	24.1%	.	.	.
GC18	19	44832312	T	A	ZNF112	Missense	p.K605N	8	105	7.6%	.	.	.
GC18	19	44833023	C	A	ZNF112	Missense	p.R368S	6	74	8.1%	.	.	.
GC18	19	47549415	C	T	TMEM160	Missense	p.G133R	7	109	6.4%	.	.	.
GC18	19	53993685	G	A	ZNF813	Missense	p.E67K	5	63	7.9%	.	.	.
GC18	19	54818759	C	T	LILRA5	Missense	p.G268E	5	89	5.6%	.	.	.
GC18	19	54969660	G	C	LENG8	Missense	p.D734H	18	119	15.1%	.	.	.
GC18	19	56041515	C	T	SBK2	Missense	p.G211E	14	106	13.2%	.	.	.
GC18	19	56481943	G	C	NLRP8	Missense	p.W805C	47	105	44.8%	.	.	.
GC18	20	2290435	G	T	TGM3	Missense	p.G47V	14	145	9.7%	.	.	.
GC18	20	20453509	C	A	RALGAPA2	Missense	p.R1820M	24	70	34.3%	.	.	.
GC18	20	23546634	G	A	CST9L	Nonsense	p.Q111X	30	73	41.1%	.	.	.
GC18	20	25297713	C	T	ABHD12	Missense	p.G182R	5	80	6.3%	.	.	.
GC18	20	30959968	T	C	ASXL1	Missense	p.V85A	7	91	7.7%	O	.	.
GC18	20	42089349	G	T	SRSF6	Missense	p.R227S	6	83	7.2%	.	.	.
GC18	20	43836958	C	G	SEMG1	Missense	p.S340R	8	64	12.5%	.	.	.
GC18	20	44141421	G	T	SPINT3	Missense	p.T47N	8	78	10.3%	.	.	.
GC18	20	47707283	G	T	CSE1L	Missense	p.G673V	7	66	10.6%	.	.	.
GC18	20	57088901	G	C	APCDD1L	Missense	p.I18M	67	72	93.1%	.	.	.
GC18	20	62707901	C	G	RGS19	Missense	p.W43C	4	39	10.3%	.	.	.
GC18	21	11098727	C	A	BAGE	Missense	p.G4V	37	291	12.7%	.	.	.
GC18	21	31768748	G	A	KRTAP13-1	Missense	p.R115K	7	59	11.9%	.	.	.
GC18	21	41423929	G	A	DSCAM	Missense	p.A1714V	13	172	7.6%	.	.	.
GC18	21	43522342	G	A	UMODL1	Missense	p.R418H	25	110	22.7%	.	.	.
GC18	21	47349870	C	T	PCBP3	Missense	p.P227S	13	210	6.2%	.	.	.
GC18	21	48081739	A	G	PRMT2	Missense	p.I228V	88	94	93.6%	.	.	.
GC18	22	19455498	C	A	UFD1	Missense	p.G107V	8	104	7.7%	.	.	.
GC18	22	25124224	G	A	PIWIL3	Nonsense	p.Q618X	63	300	21.0%	.	O	.
GC18	22	32097697	T	C	PRR14L	Missense	p.T2018A	12	174	6.9%	.	.	.
GC18	22	32242898	G	A	DEPDC5	Missense	p.G956R	5	64	7.8%	.	.	.

GC18	22	32644775	C	A	SLC5A4	Missense	p.L109F	7	137	5.1%	.	.	.
GC18	22	36688221	C	A	MYH9	Missense	p.E1385D	7	119	5.9%	.	.	.
GC18	22	38710116	C	A	CSNK1E	Missense	p.G16V	21	67	31.3%	.	.	.
GC18	22	38710117	C	A	CSNK1E	Missense	p.G16W	22	69	31.9%	.	.	.
GC18	22	40045778	C	G	CACNA1I	Missense	p.Q579E	8	133	6.0%	.	.	.
GC18	22	40661730	G	A	TNRC6B	Missense	p.G499E	9	112	8.0%	.	.	.
GC18	22	40662480	G	A	TNRC6B	Nonsense	p.W749X	8	147	5.4%	.	.	.
GC18	22	41573710	G	A	EP300	Missense	p.G1999R	6	87	6.9%	O	O	.
GC18	22	46447720	T	C	PRR34	Missense	p.E135G	9	100	9.0%	.	.	.
GC18	22	46654620	G	A	PKDREJ	Missense	p.L1534F	9	128	7.0%	.	.	.
GC18	22	50753116	G	A	DENND6B	Missense	p.L299F	9	49	18.4%	.	.	.
GC18	X	9900258	C	T	SHROOM2	Nonsense	p.Q979X	55	125	44.0%	.	.	.
GC18	X	14891842	C	T	MOSPD2	Missense	p.S14F	8	126	6.3%	.	.	.
GC18	X	16701350	C	A	CTPS2	Splicing	c.873-1G>T;NM_001144002	7	62	11.3%	.	.	.
GC18	X	16870199	G	T	RBBP7	Missense	p.T381N	4	40	10.0%	.	.	.
GC18	X	17818681	C	T	RAI2	Missense	p.G484R	5	71	7.0%	.	.	.
GC18	X	39922953	A	T	BCOR	Missense	p.I1200N	6	87	6.9%	O	.	.
GC18	X	47101645	C	G	USP11	Missense	p.S491R	13	26	50.0%	.	.	.
GC18	X	50350729	-	TCC	SHROOM4	Nonframeshift	p.E1138delinsGK	41	126	32.5%	.	.	.
GC18	X	69500077	G	T	ARR3	Missense	p.G313V	12	79	15.2%	.	.	.
GC18	X	83128981	-	A	CYLC1	Frameshift	p.E422fs	13	42	31.0%	.	.	.
GC18	X	96136711	C	G	DIAPH2	Missense	p.P194R	6	61	9.8%	.	O	.
GC18	X	104464709	C	T	TEX13A	Missense	p.A125T	9	107	8.4%	.	.	.
GC18	X	117960088	C	T	ZCCHC12	Missense	p.P294L	6	67	9.0%	.	.	.
GC18	X	129148232	C	G	BCORL1	Missense	p.A495G	6	87	6.9%	O	.	.
GC18	X	129505574	G	A	SLC25A14	Missense	p.G263R	8	87	9.2%	.	.	.
GC18	X	139038842	T	A	CXorf66	Missense	p.K100M	21	98	21.4%	.	.	.
GC18	X	140996408	C	T	MAGEC1	Missense	p.P1073L	6	74	8.1%	.	.	.
GC18	X	154132718	C	A	F8	Missense	p.V1890L	9	90	10.0%	.	.	.
GC18	X	154528182	G	A	CLIC2	Missense	p.P70L	6	38	15.8%	.	.	.

GC19	1	39854175	G	C	MACF1	Missense	p.E3159Q	8	52	15.4%	.	.	.
GC19	1	113232178	G	T	MOV10	Missense	p.Q161H	5	68	7.4%	.	.	.
GC19	1	113456713	T	C	SLC16A1	Missense	p.I435V	59	80	73.8%	.	.	.
GC19	1	154744640	C	T	KCNN3	Missense	p.R420Q	4	40	10.0%	.	O	.
GC19	1	155236565	G	T	CLK2	Missense	p.H37N	18	24	75.0%	.	.	.
GC19	1	171605306	G	A	MYOC	Missense	p.S425L	7	79	8.9%	.	.	.
GC19	1	200801870	G	T	CAMSAP2	Missense	p.S275I	42	54	77.8%	.	.	.
GC19	1	202130568	C	T	PTPN7	Missense	p.A41T	4	44	9.1%	.	.	.
GC19	1	223567488	G	T	CCDC185	Missense	p.R224L	4	23	17.4%	.	.	.
GC19	1	248263039	G	A	OR2L13	Missense	p.R121H	6	59	10.2%	.	O	.
GC19	1	249142680	T	G	ZNF672	Missense	p.C403G	5	29	17.2%	.	.	.
GC19	2	10192617	A	T	KLF11	Missense	p.M491L	4	39	10.3%	.	.	.
GC19	2	74655403	C	T	RTKN	Missense	p.V360I	4	43	9.3%	.	.	.
GC19	2	97215175	C	A	ARID5A	Missense	p.P80T	4	37	10.8%	.	.	.
GC19	2	99438617	T	C	KIAA1211L	Missense	p.K707E	6	41	14.6%	.	.	.
GC19	3	32933286	C	T	TRIM71	Nonsense	p.R864X	9	133	6.8%	.	O	.
GC19	3	51746675	G	A	GRM2	Missense	p.E213K	4	41	9.8%	.	.	.
GC19	3	52800330	C	T	NEK4	Missense	p.R52K	5	37	13.5%	.	.	.
GC19	3	62189206	A	T	PTPRG	Missense	p.E579D	5	31	16.1%	.	.	.
GC19	3	81630383	A	G	GBE1	Missense	p.I460T	45	60	75.0%	.	.	.
GC19	3	145914421	C	A	PLSCR4	Nonsense	p.E157X	7	73	9.6%	.	.	.
GC19	3	151090460	C	T	MED12L	Missense	p.S1222L	5	62	8.1%	.	.	.
GC19	3	160150818	C	T	SMC4	Nonsense	p.R1179X	4	37	10.8%	.	.	.
GC19	3	180320986	C	T	TTC14	Nonsense	p.R121X	5	60	8.3%	.	O	.
GC19	3	183883223	G	A	DVL3	Missense	p.S236N	4	36	11.1%	.	.	.
GC19	4	6380186	C	A	PPP2R2C	Missense	p.K94N	21	23	91.3%	.	.	.
GC19	4	15780150	T	A	CD38	Missense	p.V38E	5	31	16.1%	.	.	.
GC19	4	16215508	C	A	TAPT1	Missense	p.L71F	5	50	10.0%	.	.	.
GC19	4	57244315	G	A	AASDH	Missense	p.R123W	5	64	7.8%	.	O	.
GC19	4	88536490	T	A	DSPP	Missense	p.S892R	6	76	7.9%	.	.	.
GC19	4	90169494	C	G	GPRIN3	Missense	p.E590Q	5	68	7.4%	.	.	.

GC19	4	106588778	G	A	ARHGEF38	Missense	p.S689N	8	57	14.0%	.	.	.
GC19	4	106766645	G	A	GSTCD	Missense	p.E605K	14	28	50.0%	.	.	.
GC19	5	15937245	C	T	FBXL7	Missense	p.R429C	4	37	10.8%	.	O	.
GC19	5	112174008	C	G	APC	Missense	p.S888C	6	55	10.9%	O	.	.
GC19	5	135207290	G	A	SLC25A48	Missense	p.D188N	4	32	12.5%	.	.	.
GC19	5	140182638	G	A	PCDHA3	Missense	p.R619H	4	27	14.8%	.	O	.
GC19	5	176026112	C	G	GPRIN1	Missense	p.D242H	4	42	9.5%	.	.	.
GC19	5	176026114	A	T	GPRIN1	Missense	p.V241E	4	41	9.8%	.	.	.
GC19	5	177987725	G	A	COL23A1	Missense	p.R107W	4	28	14.3%	.	.	.
GC19	6	3324195	T	A	SLC22A23	Missense	p.M38L	30	42	71.4%	.	.	.
GC19	6	18122419	C	T	NHLRC1	Missense	p.R140H	4	34	11.8%	.	.	.
GC19	6	30154034	G	C	TRIM26	Missense	p.D413E	6	117	5.1%	.	.	.
GC19	6	43738463	A	C	VEGFA	Missense	p.D7A	5	22	22.7%	.	.	.
GC19	6	79735867	A	C	PHIP	Missense	p.C205W	4	41	9.8%	.	.	.
GC19	6	151121889	A	C	PLEKHG1	Missense	p.K183Q	38	63	60.3%	.	.	.
GC19	6	155450539	C	T	TIAM2	Missense	p.A61V	4	42	9.5%	.	.	.
GC19	7	70249985	T	G	AUTS2	Missense	p.L711R	5	44	11.4%	.	.	.
GC19	7	99818816	-	CT	PVRIG	Frameshift	p.T308fs	25	59	42.4%	.	.	.
GC19	7	128034510	C	T	IMPDH1	Missense	p.R455Q	8	49	16.3%	.	O	.
GC19	7	141857807	G	A	MGAM2	Missense	p.R665H	5	35	14.3%	.	.	.
GC19	7	148876246	C	T	ZNF398	Missense	p.P428S	10	69	14.5%	.	.	.
GC19	8	10465172	C	T	RP1L1	Missense	p.E2146K	4	31	12.9%	.	.	.
GC19	8	10466047	G	T	RP1L1	Missense	p.P1854Q	4	40	10.0%	.	.	.
GC19	8	22865004	A	G	RHOBTB2	Missense	p.K423E	4	43	9.3%	.	.	.
GC19	8	109796441	A	T	TMEM74	Missense	p.V296E	5	46	10.9%	.	.	.
GC19	9	36211727	C	T	CLTA	Missense	p.R153C	10	57	17.5%	.	.	.
GC19	10	24832439	G	A	KIAA1217	Missense	p.V1414I	5	77	6.5%	.	.	.
GC19	10	61829240	T	C	ANK3	Missense	p.D3800G	5	30	16.7%	.	.	.
GC19	10	71164620	C	A	TACR2	Missense	p.G387C	5	46	10.9%	.	.	.
GC19	10	72492084	G	T	ADAMTS14	Missense	p.A393S	6	39	15.4%	.	.	.
GC19	10	88696669	C	T	MMRN2	Missense	p.R894Q	5	46	10.9%	.	.	.

GC19	10	95557399	G	A	LGI1	Missense	p.D457N	5	55	9.1%	.	.	.
GC19	10	101553692	C	T	ABCC2	Missense	p.S171F	6	58	10.3%	.	.	.
GC19	10	102050227	C	A	PKD2L1	Missense	p.S639I	4	41	9.8%	.	.	.
GC19	10	105215196	C	A	CALHM1	Missense	p.E288D	4	31	12.9%	.	.	.
GC19	11	1093337	C	G	MUC2	Missense	p.P1719R	11	175	6.3%	.	.	.
GC19	11	8947204	G	A	C11orf16	Missense	p.S337F	4	43	9.3%	.	.	.
GC19	11	18731925	G	A	IGSF22	Missense	p.P884S	4	40	10.0%	.	.	.
GC19	11	18787376	C	T	PTPN5	Missense	p.M1I	19	23	82.6%	.	.	.
GC19	11	47376924	T	G	SPI1	Missense	p.M224L	4	37	10.8%	.	.	.
GC19	11	69063657	G	C	MYEOV	Missense	p.C189S	4	35	11.4%	.	.	.
GC19	11	73811627	T	C	C2CD3	Missense	p.Q892R	5	41	12.2%	.	.	.
GC19	11	92577101	C	A	FAT3	Missense	p.A3523E	7	56	12.5%	.	.	.
GC19	11	124624213	C	T	ESAM	Missense	p.G252R	4	29	13.8%	.	.	.
GC19	11	124624227	G	C	ESAM	Missense	p.A247G	4	22	18.2%	.	.	.
GC19	11	126145306	G	A	FOXRED1	Missense	p.C239Y	4	21	19.0%	.	.	.
GC19	11	126145307	C	A	FOXRED1	Nonsense	p.C239X	4	21	19.0%	.	.	.
GC19	12	992592	G	C	WNK1	Missense	p.R927T	5	39	12.8%	.	.	.
GC19	12	8374782	-	ACG	FAM90A1	Nonframeshift	p.T344delinsTS	16	28	57.1%	.	.	.
GC19	12	49426604	G	A	KMT2D	Nonsense	p.Q3962X	4	43	9.3%	O	.	.
GC19	12	100904630	G	C	NR1H4	Missense	p.V62L	5	39	12.8%	.	.	.
GC19	12	105582049	A	G	APPL2	Splicing	c.1634+2T>C; NM_001251904	6	50	12.0%	.	.	.
GC19	12	111885876	C	T	SH2B3	Missense	p.H298Y	4	32	12.5%	O	.	.
GC19	12	113812810	G	A	PLBD2	Nonsense	p.W286X	4	27	14.8%	.	.	.
GC19	13	32783104	G	T	FRY	Missense	p.G1378V	6	27	22.2%	.	.	.
GC19	13	88329622	C	A	SLITRK5	Nonsense	p.S660X	4	22	18.2%	.	.	.
GC19	14	21992083	C	G	SALL2	Missense	p.Q593H	5	38	13.2%	.	.	.
GC19	14	24029026	C	T	AP1G2	Missense	p.D383N	5	33	15.2%	.	.	.
GC19	14	24808714	C	T	RIPK3	Missense	p.R37Q	4	38	10.5%	.	.	.
GC19	14	38061411	A	C	FOXA1	Missense	p.L193R	4	27	14.8%	O	.	.
GC19	14	74727408	A	G	VSX2	Missense	p.D291G	4	24	16.7%	.	.	.

GC19	15	41961861	G	T	MGA	Missense	p.G257C	5	51	9.8%	.	.	.
GC19	15	55838667	C	A	PYGO1	Nonsense	p.E272X	5	45	11.1%	.	.	.
GC19	15	79750435	T	C	KIAA1024	Missense	p.L649S	4	46	8.7%	.	.	.
GC19	16	1033825	C	T	SOX8	Missense	p.R174W	4	31	12.9%	.	.	.
GC19	16	2580435	G	T	AMDHD2	Missense	p.P214T	5	33	15.2%	.	.	.
GC19	16	2817578	T	A	SRRM2	Missense	p.V2350E	4	37	10.8%	.	.	.
GC19	16	2819035	C	T	SRRM2	Nonsense	p.R2591X	5	40	12.5%	.	O	.
GC19	16	4016009	C	T	ADCY9	Missense	p.E1277K	6	56	10.7%	.	O	.
GC19	16	28118977	G	A	XPO6	Missense	p.T788I	37	52	71.2%	.	.	.
GC19	16	30975532	C	T	SETD1A	Nonsense	p.R253X	5	54	9.3%	.	.	.
GC19	17	7406540	G	A	POLR2A	Missense	p.E953K	4	36	11.1%	.	.	.
GC19	17	16005048	C	T	NCOR1	Missense	p.D643N	32	39	82.1%	O	.	.
GC19	17	18148885	A	T	FLII	Missense	p.L1143Q	6	58	10.3%	.	.	.
GC19	17	18832211	A	C	PRPSAP2	Missense	p.T258P	9	70	12.9%	.	.	.
GC19	17	40870055	C	T	EZH1	Missense	p.R327H	5	38	13.2%	.	O	.
GC19	17	48678513	C	T	CACNA1G	Missense	p.P1275L	4	43	9.3%	.	.	.
GC19	17	59560312	C	A	TBX4	Missense	p.P358H	4	39	10.3%	.	.	.
GC19	17	73567182	G	T	LLGL2	Splicing	c.2176+1G>T	4	21	19.0%	.	.	.
GC19	17	77073747	A	T	ENGASE	Nonsense	p.R73X	4	27	14.8%	.	.	.
GC19	18	5416032	G	A	EPB41L3	Missense	p.P618S	7	56	12.5%	.	.	.
GC19	18	9257872	A	G	ANKRD12	Missense	p.D1513G	24	36	66.7%	.	.	.
GC19	18	47809054	G	T	CXXC1	Missense	p.T631K	7	50	14.0%	.	.	.
GC19	18	70417423	C	T	NETO1	Missense	p.R472K	5	61	8.2%	.	.	.
GC19	19	1080098	C	T	ARHGAP45	Missense	p.H197Y	4	38	10.5%	.	.	.
GC19	19	2852550	A	G	ZNF555	Missense	p.I162V	34	41	82.9%	.	.	.
GC19	19	5706000	C	A	LONP1	Nonsense	p.E188X	4	35	11.4%	.	.	.
GC19	19	14876139	G	T	ADGRE2	Missense	p.T300N	5	52	9.6%	.	.	.
GC19	19	32843776	A	T	ZNF507	Missense	p.I14F	7	69	10.1%	.	.	.
GC19	19	39086627	C	T	MAP4K1	Missense	p.V711M	4	41	9.8%	.	.	.
GC19	19	41622108	-	C	CYP2F1	Frameshift	p.S5fs	51	70	72.9%	.	.	.
GC19	19	44661727	G	A	ZNF234	Missense	p.V520I	5	40	12.5%	.	.	.

GC19	19	44661728	T	G	ZNF234	Missense	p.V520G	5	40	12.5%	.	.	.
GC19	19	49442850	-	G	DHDH	Frameshift	p.G171fs	58	67	86.6%	.	.	.
GC19	19	52004792	-	C	SIGLEC12	Frameshift	p.A66fs	54	80	67.5%	.	.	.
GC19	19	52448481	T	A	ZNF613	Missense	p.F449I	5	68	7.4%	.	.	.
GC19	19	55449446	C	T	NLRP7	Missense	p.V699I	9	57	15.8%	.	.	O
GC19	19	55526104	-	CAGA	GP6	Frameshift	p.L403fs	42	110	38.2%	.	.	.
GC19	19	55870636	C	G	FAM71E2	Missense	p.A534P	4	40	10.0%	.	.	.
GC19	19	56952721	C	A	ZNF667	Missense	p.S548I	7	48	14.6%	.	.	.
GC19	20	30729348	T	C	TM9SF4	Missense	p.L93P	9	51	17.6%	.	.	.
GC19	20	34091857	A	C	CEP250	Missense	p.E1255A	5	30	16.7%	.	.	.
GC19	20	34135229	G	T	ERGIC3	Missense	p.C145F	4	37	10.8%	.	.	.
GC19	20	48894028	T	C	SMIM25	Missense	p.W23R	23	33	69.7%	.	.	.
GC19	21	43187096	C	A	RIPK4	Missense	p.V36L	19	24	79.2%	.	.	.
GC19	22	21099004	G	A	PI4KA	Missense	p.T1123I	11	77	14.3%	.	.	.
GC19	22	21348036	T	A	LZTR1	Missense	p.L449Q	4	35	11.4%	O	.	.
GC19	22	32111445	T	G	PRR14L	Missense	p.N794H	6	82	7.3%	.	.	.
GC19	X	51640054	A	C	MAGED1	Missense	p.I435L	5	66	7.6%	.	.	.
GC19	X	51640060	C	A	MAGED1	Missense	p.P437T	5	63	7.9%	.	.	.
GC19	X	53245306	T	C	KDM5C	Missense	p.K177R	4	43	9.3%	O	.	.
GC19	X	100746581	T	C	ARMCX4	Missense	p.L1002P	4	42	9.5%	.	.	.
GC19	X	102005115	C	T	BHLHB9	Nonsense	p.Q398X	4	37	10.8%	.	.	.
GC19	X	102529469	T	C	TCEAL5	Missense	p.N8S	7	69	10.1%	.	.	.
GC19	X	131540375	G	A	MBNL3	Missense	p.H25Y	4	34	11.8%	.	.	.
GC19	X	135431011	G	A	ADGRG4	Missense	p.G1716S	6	66	9.1%	.	O	.
GC20	1	20517154	A	G	UBXN10	Missense	p.M34V	77	109	70.6%	.	.	.
GC20	1	33945070	G	C	ZSCAN20	Missense	p.D61H	16	70	22.9%	.	.	.
GC20	1	85634765	T	C	SYDE2	Missense	p.T939A	65	82	79.3%	.	.	.
GC20	1	117564462	-	AA	CD101	Frameshift	p.Q762fs	83	135	61.5%	.	.	.
GC20	1	146658617	G	C	FMO5	Missense	p.I488M	16	91	17.6%	.	.	.
GC20	1	156287220	T	A	CCT3	Missense	p.E255V	12	113	10.6%	.	.	.
GC20	1	214549638	G	T	PTPN14	Missense	p.P944H	5	91	5.5%	.	.	.

GC20	1	248059780	-	A	OR2W3	Frameshift	p.K298fs	61	120	50.8%	.	.	.
GC20	1	248525329	-	TA	OR2T4	Frameshift	p.T149fs	20	42	47.6%	.	.	.
GC20	2	96943673	G	A	SNRNP200	Missense	p.P1876S	74	90	82.2%	.	.	.
GC20	2	160205739	C	T	BAZ2B	Missense	p.G1603D	61	91	67.0%	.	.	.
GC20	2	174829212	-	CCGCCA	SP3	Nonframeshift	p.G26delinsVAG	22	34	64.7%	.	.	.
GC20	2	220396565	G	C	ASIC4	Missense	p.G350A	12	74	16.2%	.	.	.
GC20	3	12458635	G	A	PPARG	Missense	p.V390I	19	77	24.7%	.	.	.
GC20	3	38040435	-	CAC	VILL	Nonframeshift	p.Y325delinsYH	50	74	67.6%	.	.	.
GC20	3	38739717	C	A	SCN10A	Missense	p.G1567V	10	76	13.2%	.	.	.
GC20	3	45127486	G	A	CDCP1	Missense	p.P719S	13	63	20.6%	.	.	.
GC20	3	48725855	C	T	IP6K2	Missense	p.D378N	13	85	15.3%	.	.	.
GC20	3	56674058	T	C	FAM208A	Missense	p.N511S	41	61	67.2%	.	.	.
GC20	3	73111482	-	A	EBLN2	Frameshift	p.R84fs	110	173	63.6%	.	O	.
GC20	3	128625022	G	A	ACAD9	Missense	p.G403D	58	75	77.3%	.	.	.
GC20	3	167742331	C	T	GOLIM4	Missense	p.E587K	17	88	19.3%	.	.	.
GC20	3	194168709	G	A	ATP13A3	Missense	p.R394C	5	72	6.9%	.	O	.
GC20	4	8589028	G	C	GPR78	Missense	p.A344P	63	83	75.9%	.	.	.
GC20	4	41983892	T	C	DCAF4L1	Missense	p.M28T	75	105	71.4%	.	.	.
GC20	4	71067991	A	T	ODAM	Missense	p.Q198L	16	96	16.7%	.	.	.
GC20	5	90398108	A	G	ADGRV1	Missense	p.Y6128C	17	105	16.2%	.	.	.
GC20	5	98204215	T	A	CHD1	Missense	p.Q1411L	6	58	10.3%	.	.	.
GC20	5	140076960	G	A	HARS2	Missense	p.R245Q	5	97	5.2%	.	.	.
GC20	6	13160475	A	T	PHACTR1	Missense	p.E60V	69	88	78.4%	.	.	.
GC20	6	27419264	G	A	ZNF184	Missense	p.H692Y	14	94	14.9%	.	.	.
GC20	6	160174524	G	C	WTAP	Missense	p.R162P	33	108	30.6%	.	O	.
GC20	7	1132462	G	C	GPER1	Missense	p.Q366H	9	50	18.0%	.	.	.
GC20	7	100678878	G	C	MUC17	Missense	p.G1394A	70	91	76.9%	.	.	.
GC20	7	142919770	T	C	TAS2R40	Missense	p.M200T	9	115	7.8%	.	.	.
GC20	8	10480296	-	G	RP1L1	Frameshift	p.P139fs	23	62	37.1%	.	.	.
GC20	8	107460258	A	G	OXR1	Missense	p.K3R	5	43	11.6%	.	.	.
GC20	8	145267938	G	A	MROH1	Missense	p.V395I	73	104	70.2%	.	.	.

GC20	9	20929393	G	T	FOCAD	Missense	p.A1039S	13	92	14.1%	.	.	.
GC20	9	111670675	G	C	ELP1	Missense	p.P108R	9	43	20.9%	.	.	.
GC20	10	12867629	A	G	CAMK1D	Missense	p.S327G	12	113	10.6%	.	.	.
GC20	10	13698924	-	CGC	FRMD4A	Nonframeshift	p.D580delinsAD	206	361	57.1%	.	.	.
GC20	10	75408164	C	T	SYNPO2L	Missense	p.G192R	4	45	8.9%	.	O	.
GC20	11	36595215	G	T	RAG1	Missense	p.D121Y	121	156	77.6%	.	.	.
GC20	11	67196646	C	T	RPS6KB2	Missense	p.P59S	5	43	11.6%	.	.	.
GC20	11	82877306	A	T	PCF11	Missense	p.K456I	10	103	9.7%	.	.	.
GC20	11	126143277	A	C	FOXRED1	Missense	p.N155T	32	51	62.7%	.	.	.
GC20	11	130785408	G	A	SNX19	Missense	p.R143W	14	83	16.9%	.	O	.
GC20	12	7476100	G	A	ACSM4	Missense	p.E418K	7	50	14.0%	.	O	.
GC20	12	8374782	-	ACG	FAM90A1	Nonframeshift	p.T344delinsTS	32	42	76.2%	.	.	.
GC20	12	48596876	-	A	OR10AD1	Frameshift	p.L67fs	190	234	81.2%	.	.	.
GC20	14	23745518	G	T	HOMEZ	Missense	p.Q307K	12	67	17.9%	.	.	.
GC20	14	60585335	G	C	PCNX4	Missense	p.D389H	8	52	15.4%	.	.	.
GC20	14	96178622	G	A	TCL1A	Missense	p.L78F	8	83	9.6%	.	.	.
GC20	15	45444206	T	G	DUOX1	Missense	p.V1050G	49	69	71.0%	.	.	.
GC20	15	77472230	C	A	PEAK1	Missense	p.S680I	18	177	10.2%	.	.	.
GC20	16	726792	G	T	RHBDL1	Missense	p.V173L	6	85	7.1%	.	.	.
GC20	16	3487148	T	C	ZNF597	Missense	p.H184R	20	144	13.9%	.	.	.
GC20	16	10775875	G	-	TEKT5	Frameshift	p.H280fs	134	180	74.4%	.	.	.
GC20	16	15967362	-	CTA	FOPNL	Nonframeshift	p.R79delinsIG	88	111	79.3%	.	.	.
GC20	16	31091862	G	A	ZNF646	Missense	p.S1406N	40	54	74.1%	.	.	.
GC20	16	31770697	-	A	ZNF720	Frameshift	p.X127delinsX	85	126	67.5%	.	.	.
GC20	16	67237617	G	A	ELMO3	Missense	p.G720D	4	44	9.1%	.	.	.
GC20	17	7606776	C	G	WRAP53	Missense	p.T540R	4	35	11.4%	.	.	.
GC20	17	26958903	C	T	KIAA0100	Missense	p.G1344D	34	96	35.4%	.	.	.
GC20	17	73626919	-	TG	RECQL5	Splicing	c.1586-2->CA	15	42	35.7%	.	O	.
GC20	18	31685020	ATCTGTTCC	-	NOL4	Nonframeshift	p.171_173del	70	132	53.0%	.	.	.
GC20	19	9086533	T	G	MUC16	Missense	p.E1761A	17	128	13.3%	.	O	.
GC20	19	10202947	T	A	C19orf66	Missense	p.V246E	5	53	9.4%	.	.	.

GC20	19	16977308	G	A	SIN3B	Missense	p.D173N	6	110	5.5%	.	.	.
GC20	19	19296867	A	G	BORCS8	Missense	p.I64T	14	22	63.6%	.	.	.
GC20	19	36128169	C	G	RBM42	Missense	p.D386E	5	37	13.5%	.	.	.
GC20	19	40363059	G	C	FCGBP	Missense	p.A5004G	10	71	14.1%	.	.	.
GC20	19	45296847	-	C	CBLC	Frameshift	p.D372fs	26	38	68.4%	.	.	.
GC20	19	52004792	-	C	SIGLEC12	Frameshift	p.A66fs	94	154	61.0%	.	.	.
GC20	19	55146175	A	C	LILRB1	Missense	p.I466L	5	86	5.8%	.	.	.
GC20	20	36852008	G	T	KIAA1755	Missense	p.Q385K	4	46	8.7%	.	.	.
GC20	20	57273794	C	A	NPEPL1	Missense	p.P188T	10	91	11.0%	.	.	.
GC20	21	43913137	G	T	RSPH1	Missense	p.A36E	21	99	21.2%	.	.	.
GC20	22	41601373	A	G	L3MBTL2	Missense	p.K3E	12	87	13.8%	.	.	.
GC20	X	3238516	G	A	MXRA5	Missense	p.T1737I	15	92	16.3%	.	.	.
GC20	X	12903892	G	C	TLR7	Missense	p.D89H	27	138	19.6%	.	.	.
GC20	X	16778432	G	A	SYAP1	Missense	p.D337N	10	71	14.1%	.	.	.
GC20	X	34150322	C	A	FAM47A	Missense	p.R25L	12	67	17.9%	.	.	.
GC20	X	73745612	G	T	SLC16A2	Nonsense	p.E352X	5	79	6.3%	.	.	.
GC20	X	139866017	G	C	CDR1	Missense	p.S172C	6	127	4.7%	.	.	.
GC20	X	139866018	A	C	CDR1	Missense	p.S172A	6	128	4.7%	.	.	.
GC20	X	153880883	A	G	CTAG2	Missense	p.S98P	5	68	7.4%	.	.	.
GC21	1	25678127	G	T	TMEM50A	Missense	p.V73L	21	63	33.3%	.	.	.
GC21	1	29475079	C	T	SRSF4	Missense	p.R443K	6	101	5.9%	.	.	.
GC21	1	38482041	G	A	UTP11	Missense	p.R25Q	4	39	10.3%	.	O	.
GC21	1	43887808	T	A	SZT2	Missense	p.Y561N	4	35	11.4%	.	.	.
GC21	1	46663447	C	T	POMGNT1	Missense	p.R16Q	4	37	10.8%	.	.	.
GC21	1	52705111	A	T	ZFYVE9	Missense	p.R674S	6	62	9.7%	.	.	.
GC21	1	75172014	C	T	CRYZ	Missense	p.S182N	22	53	41.5%	.	.	.
GC21	1	90058573	C	T	LRRC8B	Missense	p.R795C	15	69	21.7%	.	.	.
GC21	1	91178062	C	A	BARHL2	Missense	p.S324I	4	25	16.0%	.	.	.
GC21	1	115282408	G	C	CSDE1	Missense	p.T35S	39	88	44.3%	.	.	.
GC21	1	152127289	A	T	RPTN	Missense	p.H762Q	17	114	14.9%	.	.	.
GC21	1	152327800	C	T	FLG2	Missense	p.G821E	5	77	6.5%	.	.	.

GC21	1	158725455	G	A	OR6K6	Missense	p.V284I	6	55	10.9%	.	.	.
GC21	1	159176204	G	T	ACKR1	Missense	p.W327C	18	70	25.7%	.	.	.
GC21	1	160395043	A	C	VANGL2	Missense	p.K481Q	4	43	9.3%	.	.	.
GC21	1	161161211	A	G	ADAMTS4	Missense	p.L744P	14	34	41.2%	.	.	.
GC21	1	167096117	C	A	DUSP27	Missense	p.D583E	8	35	22.9%	.	.	.
GC21	1	169677611	C	T	SELL	Missense	p.G153D	16	43	37.2%	.	.	.
GC21	1	200877935	G	A	C1orf106	Missense	p.G218S	7	29	24.1%	.	O	.
GC21	1	205138741	A	G	DSTYK	Missense	p.S292P	19	54	35.2%	.	.	.
GC21	1	247588549	C	T	NLRP3	Nonsense	p.Q602X	4	35	11.4%	.	O	.
GC21	1	248525329	-	TA	OR2T4	Frameshift	p.T149fs	18	39	46.2%	.	.	.
GC21	2	49217721	G	C	FSHR	Missense	p.L144V	4	47	8.5%	.	.	.
GC21	2	51255041	C	T	NRXN1	Missense	p.R124H	4	38	10.5%	.	.	.
GC21	2	54483162	G	T	TSPYL6	Missense	p.R43S	5	30	16.7%	.	.	.
GC21	2	99012988	A	G	CNGA3	Missense	p.D434G	17	45	37.8%	.	.	.
GC21	2	141812700	C	T	LRP1B	Missense	p.G513S	5	49	10.2%	O	.	.
GC21	2	159533310	G	A	PKP4	Missense	p.D1062N	20	58	34.5%	.	O	.
GC21	2	173292609	C	A	ITGA6	Missense	p.D31E	5	47	10.6%	.	.	.
GC21	2	186653458	C	T	FSIP2	Missense	p.T532I	15	39	38.5%	.	.	.
GC21	2	203055022	G	A	KIAA2012	Missense	p.E873K	9	43	20.9%	.	.	.
GC21	2	207310243	A	G	ADAM23	Missense	p.N143D	16	45	35.6%	.	.	.
GC21	2	223389693	G	C	SGPP2	Missense	p.V69L	6	68	8.8%	.	.	.
GC21	2	232393316	A	T	NMUR1	Missense	p.L139Q	4	33	12.1%	.	.	.
GC21	3	36779310	G	T	DCLK3	Missense	p.Q281K	4	46	8.7%	.	.	.
GC21	3	48921471	C	A	SLC25A20	Missense	p.L95F	4	39	10.3%	.	.	.
GC21	3	49831367	C	T	CDHR4	Missense	p.R451Q	4	35	11.4%	.	.	.
GC21	3	50154781	G	A	RBM5	Missense	p.R764Q	4	32	12.5%	.	.	.
GC21	3	52395681	G	A	DNAH1	Missense	p.G1627R	16	42	38.1%	.	.	.
GC21	3	73111482	-	A	EBLN2	Frameshift	p.R84fs	88	116	75.9%	.	O	.
GC21	3	119263646	C	T	CD80	Missense	p.E57K	8	83	9.6%	.	.	.
GC21	3	126707877	C	A	PLXNA1	Missense	p.F147L	4	36	11.1%	.	.	.
GC21	3	130110459	G	A	COL6A5	Missense	p.G952S	4	46	8.7%	.	.	.

GC21	3	133099529	C	A	TMEM108	Missense	p.P325Q	15	39	38.5%	.	.	.
GC21	3	159711557	A	C	IL12A	Missense	p.K178Q	9	41	22.0%	.	.	.
GC21	4	40440616	C	T	RBM47	Missense	p.E99K	4	38	10.5%	.	.	.
GC21	4	47565783	G	C	ATP10D	Splicing	c.2853+1G>C	8	39	20.5%	.	.	.
GC21	4	55962456	T	C	KDR	Missense	p.I890V	15	35	42.9%	O	.	.
GC21	4	95539218	G	C	PDLIM5	Missense	p.E3D	13	42	31.0%	.	.	.
GC21	4	186231815	G	C	SNX25	Missense	p.D233H	6	60	10.0%	.	.	.
GC21	5	1085537	C	T	SLC12A7	Missense	p.E243K	5	46	10.9%	.	.	.
GC21	5	63257014	G	C	HTR1A	Missense	p.P178R	18	37	48.6%	.	.	.
GC21	5	80626713	G	T	ACOT12	Missense	p.P480T	9	21	42.9%	.	.	.
GC21	5	95119539	G	A	RHOBTB3	Missense	p.E499K	7	42	16.7%	.	.	.
GC21	5	123984417	C	T	ZNF608	Missense	p.D554N	5	86	5.8%	.	.	.
GC21	5	127609653	G	C	FBN2	Missense	p.N2573K	7	64	10.9%	.	.	.
GC21	5	137666752	T	C	CDC25C	Missense	p.T40A	10	67	14.9%	.	.	.
GC21	5	148206786	G	A	ADRB2	Missense	p.R131H	18	56	32.1%	.	.	.
GC21	5	152871789	-	C	GRIA1	Frameshift	p.L11fs	58	149	38.9%	.	.	.
GC21	5	169812331	G	A	KCNMB1	Missense	p.L41F	4	24	16.7%	.	O	.
GC21	6	10586752	-	T	GCNT2	Frameshift	p.L177fs	50	137	36.5%	.	.	.
GC21	6	26235133	G	A	HIST1H1D	Missense	p.T10I	10	36	27.8%	.	.	.
GC21	6	43550061	C	T	POLH	Missense	p.A2V	33	70	47.1%	.	.	.
GC21	6	56483049	C	G	DST	Missense	p.R1928P	20	54	37.0%	.	.	.
GC21	6	90660498	T	A	BACH2	Missense	p.T443S	5	56	8.9%	.	.	.
GC21	6	138745629	G	C	NHSL1	Missense	p.S1474R	4	40	10.0%	.	.	.
GC21	6	152653025	T	A	SYNE1	Missense	p.R4194S	11	55	20.0%	.	.	.
GC21	7	12391269	-	A	VWDE	Frameshift	p.N1157fs	42	76	55.3%	.	.	.
GC21	7	24756895	G	T	DFNA5	Nonsense	p.Y61X	10	47	21.3%	.	.	.
GC21	7	48619914	G	A	ABCA13	Missense	p.E4817K	5	45	11.1%	.	.	.
GC21	7	91794414	C	T	LRRD1	Missense	p.E35K	5	77	6.5%	.	.	.
GC21	7	106508233	T	G	PIK3CG	Missense	p.L76R	4	31	12.9%	.	.	.
GC21	7	130135300	C	T	MEST	Missense	p.L40F	4	45	8.9%	.	.	.
GC21	7	139107067	G	T	LUC7L2	Missense	p.R387L	9	72	12.5%	.	.	.

GC21	8	2820777	C	A	CSMD1	Missense	p.G3141W	14	31	45.2%	.	.	.
GC21	8	144356945	G	A	GLI4	Missense	p.E66K	8	50	16.0%	.	.	.
GC21	9	34637391	G	A	SIGMAR1	Missense	p.R60C	4	41	9.8%	.	.	.
GC21	9	35807141	C	T	NPR2	Nonsense	p.Q881X	4	20	20.0%	.	.	.
GC21	9	75366803	C	A	TMC1	Missense	p.F191L	6	47	12.8%	.	.	.
GC21	9	96214545	C	T	FAM120A	Nonsense	p.W149X	4	36	11.1%	.	.	.
GC21	9	115422234	G	A	KIAA1958	Missense	p.R679Q	4	41	9.8%	.	.	.
GC21	9	120475682	C	T	TLR4	Missense	p.H226Y	23	50	46.0%	.	.	.
GC21	9	125391771	-	A	OR1B1	Frameshift	p.L15fs	66	123	53.7%	.	.	.
GC21	9	125437831	-	TT	OR1L3	Frameshift	p.C141fs	31	101	30.7%	.	.	.
GC21	9	132631160	C	G	USP20	Missense	p.H385Q	4	43	9.3%	.	.	.
GC21	9	136330529	G	A	CACFD1	Missense	p.D94N	5	46	10.9%	.	.	.
GC21	10	5540972	C	T	CALML5	Missense	p.A144T	5	42	11.9%	.	.	.
GC21	10	18276530	T	A	SLC39A12	Missense	p.L273M	11	30	36.7%	.	.	.
GC21	10	27702257	-	C	PTCHD3	Frameshift	p.G308fs	24	76	31.6%	.	.	.
GC21	10	105793793	A	T	COL17A1	Missense	p.Y1356N	4	46	8.7%	.	.	.
GC21	10	115537269	C	A	PLEKHS1	Missense	p.A423E	5	53	9.4%	.	.	.
GC21	10	121663665	G	A	SEC23IP	Missense	p.R326Q	6	76	7.9%	.	O	.
GC21	11	5989316	A	-	OR56A5	Frameshift	p.S137fs	42	125	33.6%	.	.	.
GC21	11	6477838	C	T	TRIM3	Missense	p.R254H	4	35	11.4%	.	.	.
GC21	11	6646557	C	A	DCHS1	Nonsense	p.E2340X	4	38	10.5%	.	.	.
GC21	11	8951039	G	T	C11orf16	Missense	p.A70E	17	46	37.0%	.	.	.
GC21	11	21581765	C	T	NELL1	Missense	p.T549I	6	59	10.2%	.	.	.
GC21	11	55927322	T	C	OR8K5	Missense	p.M158V	6	54	11.1%	.	.	.
GC21	11	56230165	G	A	OR5M9	Missense	p.T238I	16	42	38.1%	.	.	.
GC21	11	65373417	C	A	MAP3K11	Missense	p.R580M	5	40	12.5%	.	.	.
GC21	11	66114304	C	T	B4GAT1	Missense	p.R238K	6	61	9.8%	.	.	.
GC21	11	66262882	G	A	DPP3	Missense	p.S470N	4	32	12.5%	.	.	.
GC21	11	75136490	C	A	KLHL35	Missense	p.G441V	4	37	10.8%	.	.	.
GC21	11	86160992	A	T	ME3	Missense	p.V357E	5	30	16.7%	.	.	.
GC21	11	96125119	A	G	JRKL	Missense	p.S436G	26	60	43.3%	.	.	.

GC21	11	118983197	C	A	C2CD2L	Missense	p.L364I	4	42	9.5%	.	.	.
GC21	11	119044648	G	A	NLRX1	Missense	p.M230I	4	43	9.3%	.	.	.
GC21	11	123597070	G	T	ZNF202	Missense	p.Q304K	5	60	8.3%	.	.	.
GC21	12	8374782	-	ACG	FAM90A1	Nonframeshift	p.T344delinsTS	15	46	32.6%	.	.	.
GC21	12	42854131	C	T	PRICKLE1	Missense	p.R659H	22	55	40.0%	.	.	.
GC21	12	48596876	-	A	OR10AD1	Frameshift	p.L67fs	54	156	34.6%	.	.	.
GC21	12	49722825	G	T	TROAP	Missense	p.G336V	4	41	9.8%	.	.	.
GC21	12	53185150	C	T	KRT3	Missense	p.D459N	11	34	32.4%	.	.	.
GC21	12	57389129	G	A	GPR182	Missense	p.E46K	4	44	9.1%	.	.	.
GC21	12	92821895	-	TAAGTTCG	CLLU1OS	Nonsense	p.K10_E11delinsRTX	21	60	35.0%	.	.	.
GC21	12	111078188	G	T	TCTN1	Missense	p.V282F	5	71	7.0%	.	.	.
GC21	12	122247761	C	T	SETD1B	Missense	p.P304S	25	47	53.2%	.	.	.
GC21	12	123472421	C	T	PITPNM2	Missense	p.D1003N	5	54	9.3%	.	.	.
GC21	12	132471306	C	T	EP400	Missense	p.S726L	5	66	7.6%	.	O	.
GC21	13	97484868	C	G	HS6ST3	Missense	p.P278A	21	40	52.5%	.	.	.
GC21	13	100511164	G	A	CLYBL	Missense	p.G100D	10	81	12.3%	.	.	.
GC21	13	107145556	G	T	EFNB2	Missense	p.S278R	5	50	10.0%	.	.	.
GC21	14	24655501	C	T	IPO4	Missense	p.A333T	5	77	6.5%	.	.	.
GC21	14	39650372	C	T	PNN	Nonsense	p.Q487X	5	69	7.2%	.	.	.
GC21	14	47120647	C	T	RPL10L	Missense	p.R98H	4	39	10.3%	.	.	.
GC21	14	74823839	A	T	VRTN	Missense	p.Y118F	5	49	10.2%	.	.	.
GC21	14	95657991	C	G	CLMN	Missense	p.Q973H	4	35	11.4%	.	.	.
GC21	15	34648986	T	C	NUTM1	Missense	p.I916T	16	54	29.6%	.	O	.
GC21	15	72192100	T	C	MYO9A	Missense	p.K1133R	6	63	9.5%	.	.	.
GC21	15	85164351	G	C	ZSCAN2	Missense	p.A309P	4	49	8.2%	.	.	.
GC21	15	90127681	C	A	TICRR	Missense	p.P366T	11	44	25.0%	.	.	.
GC21	16	1570172	G	A	IFT140	Missense	p.A1278V	5	44	11.4%	.	.	.
GC21	16	18848720	T	G	SMG1	Missense	p.K2487Q	19	48	39.6%	.	.	.
GC21	16	24817902	C	T	TNRC6A	Missense	p.P1397L	22	55	40.0%	.	.	.
GC21	16	31770697	-	A	ZNF720	Frameshift	p.X127delinsX	40	84	47.6%	.	.	.
GC21	16	47005365	ATC	-	DNAJA2	Nonframeshift	p.86_86del	61	165	37.0%	.	.	.

GC21	16	67183716	C	T	B3GNT9	Missense	p.V225I	7	71	9.9%	.	O	.
GC21	16	67919966	A	C	NRN1L	Missense	p.E101A	4	31	12.9%	.	.	.
GC21	16	68328673	G	A	SLC7A6	Missense	p.R363H	6	113	5.3%	.	.	.
GC21	16	68598266	G	C	ZFP90	Missense	p.E526Q	6	59	10.2%	.	.	.
GC21	16	69728052	A	C	NFAT5	Missense	p.N1424H	13	45	28.9%	.	.	.
GC21	16	72110547	T	A	HPR	Missense	p.M205K	30	61	49.2%	.	.	.
GC21	16	75269596	C	A	BCAR1	Missense	p.D191Y	4	37	10.8%	.	.	.
GC21	16	88873690	T	G	CDT1	Missense	p.I426S	13	27	48.1%	.	.	.
GC21	17	3662802	A	T	ITGAE	Missense	p.F254I	5	36	13.9%	.	.	.
GC21	17	7221221	C	G	NEURL4	Missense	p.S1362T	20	50	40.0%	.	.	.
GC21	17	7827747	C	T	KCNAB3	Missense	p.A233T	20	52	38.5%	.	.	.
GC21	17	7834415	C	T	TRAPPC1	Missense	p.E75K	5	69	7.2%	.	.	.
GC21	17	18221109	A	G	SMCR8	Missense	p.D669G	5	61	8.2%	.	.	.
GC21	17	19186736	G	A	EPN2	Missense	p.A102T	4	44	9.1%	.	O	.
GC21	17	28811300	T	C	GOSR1	Missense	p.I9T	34	73	46.6%	.	.	.
GC21	17	36485427	C	T	GPR179	Missense	p.R1342K	4	42	9.5%	.	.	.
GC21	17	39635962	G	C	KRT35	Missense	p.D181E	5	31	16.1%	.	.	.
GC21	17	45747099	C	A	KPNB1	Missense	p.T282K	5	64	7.8%	.	.	.
GC21	17	74288904	C	G	QRICH2	Missense	p.R469T	10	58	17.2%	.	.	.
GC21	17	75209468	G	C	SEC14L1	Missense	p.A612P	6	42	14.3%	.	.	.
GC21	18	31226306	A	G	ASXL3	Missense	p.E115G	23	75	30.7%	.	.	.
GC21	18	42533114	G	A	SETBP1	Missense	p.G1270D	16	42	38.1%	O	.	.
GC21	18	47809049	G	A	CXXC1	Missense	p.R633C	5	55	9.1%	.	.	.
GC21	19	9949219	C	T	PIN1	Missense	p.R56C	4	37	10.8%	.	.	.
GC21	19	18474275	C	A	PGPEP1	Missense	p.P94Q	5	45	11.1%	.	.	.
GC21	19	19656361	C	T	CILP2	Missense	p.R1003W	4	30	13.3%	.	O	.
GC21	19	41622108	-	C	CYP2F1	Frameshift	p.S5fs	21	61	34.4%	.	.	.
GC21	19	41808776	C	T	HNRNPUL1	Nonsense	p.R543X	7	50	14.0%	.	.	.
GC21	19	44116527	G	A	SRRM5	Missense	p.R85H	15	37	40.5%	.	O	.
GC21	19	44590632	G	C	ZNF284	Missense	p.C334S	5	67	7.5%	.	.	.
GC21	19	44591019	G	C	ZNF284	Missense	p.S463T	5	60	8.3%	.	.	.

GC21	19	45888861	G	A	PPP1R13L	Missense	p.P736L	4	29	13.8%	.	.	.
GC21	19	49442850	-	G	DHDH	Frameshift	p.G171fs	54	58	93.1%	.	.	.
GC21	19	52004792	-	C	SIGLEC12	Frameshift	p.A66fs	34	81	42.0%	.	.	.
GC21	19	56185355	C	T	U2AF2	Missense	p.T446M	4	33	12.1%	.	O	.
GC21	19	57802841	G	A	ZNF460	Missense	p.S270N	5	71	7.0%	.	.	.
GC21	19	58048797	C	T	ZNF549	Missense	p.S129L	6	67	9.0%	.	.	.
GC21	19	58319773	G	C	ZNF552	Missense	p.Q287E	9	35	25.7%	.	.	.
GC21	19	58490885	G	A	ZNF606	Missense	p.T298I	5	73	6.8%	.	.	.
GC21	20	3735140	C	A	C20orf27	Missense	p.A135S	5	38	13.2%	.	.	.
GC21	20	25457073	G	T	NINL	Missense	p.Q952K	4	34	11.8%	.	.	.
GC21	20	33328503	C	T	NCOA6	Missense	p.E1853K	5	65	7.7%	.	.	.
GC21	20	37580541	A	G	FAM83D	Missense	p.D379G	14	42	33.3%	.	.	.
GC21	20	45927466	A	T	ZMYND8	Missense	p.S154T	11	64	17.2%	.	.	.
GC21	21	15592017	G	A	RBM11	Missense	p.G77E	5	76	6.6%	.	O	.
GC21	22	18370108	G	A	MICAL3	Missense	p.S662F	5	44	11.4%	.	.	.
GC21	22	21989525	T	G	CCDC116	Missense	p.S391R	19	38	50.0%	.	.	.
GC21	22	22842565	C	T	ZNF280B	Missense	p.V387I	4	44	9.1%	.	.	.
GC21	22	23524102	C	T	BCR	Missense	p.R319W	6	52	11.5%	.	.	.
GC21	22	29885568	-	AAGTCCCCTGAGAAGGCC	NEFH	Nonsense	p.K647delinsKVPX	34	89	38.2%	.	.	.
GC21	22	39498016	G	A	APOBEC3H	Missense	p.R171Q	6	53	11.3%	.	O	.
GC21	X	12939818	G	A	TLR8	Missense	p.D887N	5	69	7.2%	.	.	.
GC21	X	16773139	C	T	SYAP1	Missense	p.T216M	8	55	14.5%	.	.	.
GC21	X	38145992	C	T	RPGR	Missense	p.E754K	4	37	10.8%	.	.	.
GC21	X	47074037	G	A	UBA1	Splicing	c.3041+1G>A	5	70	7.1%	.	.	.
GC21	X	50654022	C	T	BMP15	Missense	p.S80L	4	45	8.9%	.	.	.
GC21	X	70465648	G	T	ZMYM3	Missense	p.D898E	4	37	10.8%	.	.	.
GC21	X	77276512	C	T	ATP7A	Missense	p.A873V	10	60	16.7%	.	.	.
GC21	X	111145315	G	A	TRPC5OS	Missense	p.E80K	5	41	12.2%	.	.	.
GC21	X	111145356	G	A	TRPC5OS	Missense	p.M93I	6	41	14.6%	.	.	.
GC21	X	111698776	C	A	RTL4	Missense	p.Q274K	22	56	39.3%	.	.	.
GC21	X	111698777	A	C	RTL4	Missense	p.Q274P	22	55	40.0%	.	.	.

GC21	X	135432121	A	G	ADGRG4	Missense	p.T2086A	23	60	38.3%	.	.	.
GC21	X	153053560	G	A	IDH3G	Missense	p.P126L	5	46	10.9%	.	O	.
GC22	1	3732024	A	T	CEP104	Missense	p.I907N	23	71	32.4%	.	.	.
GC22	1	11017722	G	A	C1orf127	Missense	p.T215I	20	48	41.7%	.	.	.
GC22	1	11129736	T	C	EXOSC10	Missense	p.T765A	39	114	34.2%	.	.	.
GC22	1	20066382	C	-	TMCO4	Frameshift	p.V372fs	28	91	30.8%	.	O	.
GC22	1	40092291	T	C	HEYL	Missense	p.Y292C	5	81	6.2%	.	.	.
GC22	1	43784966	A	G	TIE1	Missense	p.I950V	8	75	10.7%	.	.	.
GC22	1	44137412	C	T	KDM4A	Nonsense	p.R534X	27	86	31.4%	.	.	.
GC22	1	45298002	-	C	PTCH2	Frameshift	p.V93fs	16	48	33.3%	.	.	.
GC22	1	53556389	G	A	SLC1A7	Missense	p.A302V	22	62	35.5%	.	.	.
GC22	1	64022857	T	-	EFCAB7	Frameshift	p.N429fs	61	194	31.4%	.	O	.
GC22	1	67147729	A	G	SGIP1	Missense	p.E335G	39	101	38.6%	.	.	.
GC22	1	78478908	G	T	DNAJB4	Missense	p.G14C	72	211	34.1%	.	.	.
GC22	1	85724270	C	T	C1orf52	Missense	p.D138N	37	99	37.4%	.	.	.
GC22	1	91404039	A	C	ZNF644	Missense	p.L958V	66	147	44.9%	.	.	.
GC22	1	93170274	T	A	EVI5	Missense	p.L103F	49	141	34.8%	.	.	.
GC22	1	145075853	T	A	PDE4DIP	Missense	p.T4S	23	528	4.4%	.	.	.
GC22	1	150240400	G	T	APH1A	Missense	p.L81I	27	152	17.8%	.	.	.
GC22	1	155223513	A	G	FAM189B	Missense	p.F79S	53	267	19.9%	.	.	.
GC22	1	156256123	T	A	TMEM79	Missense	p.V277E	33	147	22.4%	.	.	.
GC22	1	168250471	C	T	TBX19	Missense	p.A48V	38	237	16.0%	.	.	.
GC22	1	179853883	-	TCA	TOR1AIP1	Nonframeshift	p.R178delinsIR	74	218	33.9%	.	.	.
GC22	1	192129557	C	T	RGS18	Missense	p.L91F	35	160	21.9%	.	O	.
GC22	1	202736181	T	C	KDM5B	Missense	p.Q195R	25	154	16.2%	.	O	.
GC22	1	202746164	T	A	KDM5B	Missense	p.Q88L	62	334	18.6%	.	.	.
GC22	1	210522395	T	A	HHAT	Missense	p.Y26N	26	195	13.3%	.	.	.
GC22	1	214791950	G	A	CENPF	Missense	p.A132T	31	162	19.1%	.	.	.
GC22	1	224922396	A	C	CNIH3	Missense	p.Y132S	15	173	8.7%	.	.	.
GC22	1	226180254	A	G	SDE2	Missense	p.V122A	11	87	12.6%	.	.	.
GC22	1	232574980	T	C	SIPA1L2	Missense	p.D1302G	18	137	13.1%	.	.	.

GC22	1	247695126	T	C	OR2C3	Missense	p.R230G	87	240	36.3%	.	.	.
GC22	1	248525329	-	TA	OR2T4	Frameshift	p.T149fs	60	109	55.0%	.	.	.
GC22	2	3278191	C	T	EIPR1	Splicing	c.260-1G>A	35	118	29.7%	.	.	.
GC22	2	11706696	A	G	GREB1	Missense	p.K123R	21	92	22.8%	.	.	.
GC22	2	17962136	A	G	GEN1	Missense	p.I553V	93	249	37.3%	.	.	.
GC22	2	29456492	C	A	ALK	Missense	p.R809I	25	72	34.7%	O	.	.
GC22	2	54093345	T	-	PSME4	Frameshift	p.T1805fs	49	159	30.8%	.	O	.
GC22	2	111666425	A	G	ACOXL	Missense	p.Q283R	11	82	13.4%	.	.	.
GC22	2	170127494	T	A	LRP2	Missense	p.D747V	38	120	31.7%	.	.	.
GC22	2	172951443	A	-	DLX1	Frameshift	p.G125fs	44	140	31.4%	.	.	.
GC22	2	182394343	A	G	ITGA4	Missense	p.T836A	54	163	33.1%	.	.	.
GC22	2	186662103	A	-	FSIP2	Frameshift	p.K3414fs	55	151	36.4%	.	O	.
GC22	2	219114132	T	A	ARPC2	Missense	p.I241N	47	135	34.8%	.	.	.
GC22	2	239002571	G	A	SCLY	Missense	p.E339K	23	41	56.1%	.	.	.
GC22	3	10251315	C	A	IRAK2	Missense	p.P156H	24	80	30.0%	.	.	.
GC22	3	12626691	G	A	RAF1	Missense	p.S533F	79	257	30.7%	.	.	.
GC22	3	12977930	C	T	IQSEC1	Missense	p.V196M	35	93	37.6%	.	.	.
GC22	3	39118673	T	C	WDR48	Missense	p.V232A	27	90	30.0%	.	.	.
GC22	3	50414911	-	GTCA	CACNA2D2	Frameshift	p.T538fs	38	124	30.6%	.	.	.
GC22	3	52004098	-	G	ABHD14B	Frameshift	p.P67fs	29	86	33.7%	.	.	.
GC22	3	54603860	T	C	CACNA2D3	Missense	p.F239L	53	163	32.5%	.	.	.
GC22	3	58089685	G	A	FLNB	Splicing	c.1484-1G>A;NM_001457	20	70	28.6%	.	.	.
GC22	3	58135590	A	G	FLNB	Missense	p.I2011M	34	114	29.8%	.	.	.
GC22	3	115395200	-	C	GAP43	Frameshift	p.A124fs	30	95	31.6%	.	.	.
GC22	3	122423539	A	-	PARP14	Frameshift	p.K1162fs	93	283	32.9%	.	.	.
GC22	4	1019074	A	T	FGFRL1	Missense	p.H485L	52	191	27.2%	.	O	.
GC22	4	6826217	C	T	KIAA0232	Missense	p.P13S	46	158	29.1%	.	.	.
GC22	4	6863968	G	A	KIAA0232	Missense	p.S620N	89	226	39.4%	.	.	.
GC22	4	10105577	C	T	WDR1	Missense	p.A58T	17	58	29.3%	.	O	.
GC22	4	15004879	-	GCCGCC	CPEB2	Nonframeshift	p.K194delinsKAA	49	89	55.1%	.	O	.

GC22	4	73013281	A	C	NPFFR2	Missense	p.N441H	31	177	17.5%	.	.	.
GC22	4	100047746	T	-	ADH4	Frameshift	p.S373fs	48	145	33.1%	.	O	.
GC22	4	106604431	G	A	INTS12	Missense	p.S283F	23	95	24.2%	.	.	.
GC22	4	110402932	A	G	SEC24B	Missense	p.E387G	19	99	19.2%	.	.	.
GC22	4	141548640	C	T	TBC1D9	Missense	p.A952T	34	88	38.6%	.	.	.
GC22	5	14871543	G	T	ANKH	Missense	p.P5Q	23	63	36.5%	.	.	.
GC22	5	65372201	G	T	ERBIN	Missense	p.G1231W	28	101	27.7%	.	.	.
GC22	5	75581011	C	T	SV2C	Missense	p.A313V	12	103	11.7%	.	.	.
GC22	5	80932421	T	-	SSBP2	Frameshift	p.N51fs	21	61	34.4%	.	O	.
GC22	5	89757135	C	A	MBLAC2	Missense	p.G230V	7	196	3.6%	.	.	.
GC22	5	140719668	G	T	PCDHGA2	Missense	p.G377V	8	209	3.8%	.	.	.
GC22	5	140753871	T	-	PCDHGA6	Frameshift	p.L74fs	37	108	34.3%	.	.	.
GC22	5	149420391	G	A	HMGXB3	Missense	p.D832N	67	160	41.9%	.	.	.
GC22	5	151784497	C	-	NMUR2	Frameshift	p.V60fs	44	109	40.4%	.	.	.
GC22	5	171523494	A	G	STK10	Missense	p.V314A	15	61	24.6%	.	.	.
GC22	5	175394992	A	T	THOC3	Missense	p.S74T	5	41	12.2%	.	.	.
GC22	6	12164370	A	-	HIVEP1	Frameshift	p.A2611fs	67	219	30.6%	.	O	.
GC22	6	20649568	G	A	CDKAL1	Missense	p.V111I	23	101	22.8%	.	.	.
GC22	6	32097318	-	AGACTTATG AGA	FKBPL	Nonframeshift	p.S80delinsSLI SL	47	128	36.7%	.	.	.
GC22	6	41162407	A	G	TREML2	Missense	p.S181P	22	63	34.9%	.	.	.
GC22	6	64422132	ATTT	-	PHF3	Frameshift	p.I1550fs	49	155	31.6%	.	.	.
GC22	6	100838424	T	A	SIM1	Missense	p.Y705F	51	173	29.5%	.	.	.
GC22	6	109560663	C	T	C6orf183	Missense	p.L85F	14	59	23.7%	.	.	.
GC22	6	112392640	A	T	TUBE1	Nonsense	p.L468X	55	175	31.4%	.	.	.
GC22	6	112393994	A	G	TUBE1	Missense	p.V354A	33	103	32.0%	.	.	.
GC22	6	138539444	C	G	PBOV1	Missense	p.S30T	65	198	32.8%	.	.	.
GC22	7	12391269	-	A	VWDE	Frameshift	p.N1157fs	72	145	49.7%	.	.	.
GC22	7	42950024	T	C	C7orf25	Missense	p.Q217R	65	259	25.1%	.	.	.
GC22	7	44801338	G	T	ZMIZ2	Missense	p.R451L	43	132	32.6%	.	.	.
GC22	7	48314853	C	A	ABCA13	Missense	p.L1864M	49	198	24.7%	.	.	.
GC22	7	50611653	G	A	DDC	Missense	p.A44V	24	107	22.4%	.	.	.

GC22	7	56049217	G	A	NIPSNAP2	Nonsense	p.W95X	30	82	36.6%	.	.	.
GC22	7	64166756	C	A	ZNF107	Missense	p.S62Y	30	118	25.4%	.	O	.
GC22	7	77408300	G	A	RSBN1L	Missense	p.V786I	92	262	35.1%	.	.	.
GC22	7	98530985	T	A	TRRAP	Missense	p.M1325K	18	58	31.0%	O	.	.
GC22	7	98988542	T	C	ARPC1B	Missense	p.V176A	35	120	29.2%	.	.	.
GC22	7	99129797	C	-	ZKSCAN5	Frameshift	p.S742fs	50	163	30.7%	.	.	.
GC22	7	99704407	C	T	AP4M1	Nonsense	p.R422X	33	133	24.8%	.	.	.
GC22	7	100086417	C	A	NYAP1	Missense	p.P358H	7	137	5.1%	.	.	.
GC22	7	122303392	C	T	CADPS2	Missense	p.V229M	61	276	22.1%	.	.	.
GC22	7	134849377	C	A	TMEM140	Missense	p.L62I	51	198	25.8%	.	.	.
GC22	7	139305221	T	C	HIPK2	Missense	p.T570A	8	153	5.2%	.	O	.
GC22	7	150737985	G	A	ABCB8	Missense	p.A399T	30	126	23.8%	.	.	.
GC22	8	12952709	C	A	DLC1	Missense	p.K560N	8	27	29.6%	.	.	.
GC22	8	12968261	C	A	DLC1	Missense	p.A95S	22	114	19.3%	.	.	.
GC22	8	17612449	G	A	MTUS1	Nonsense	p.Q290X	77	267	28.8%	.	.	.
GC22	8	22291446	C	A	SLC39A14	Missense	p.H459N	6	82	7.3%	.	.	.
GC22	8	24249853	G	T	ADAMDEC<br>1	Missense	p.R56I	42	130	32.3%	.	.	.
GC22	8	53084709	T	C	ST18	Missense	p.T238A	67	193	34.7%	.	.	.
GC22	8	54870893	A	T	RGS20	Missense	p.I112L	36	152	23.7%	.	.	.
GC22	8	54870906	C	T	RGS20	Missense	p.A116V	34	144	23.6%	.	.	.
GC22	8	55537537	T	G	RP1	Missense	p.S365R	55	202	27.2%	.	.	.
GC22	8	55537559	A	T	RP1	Missense	p.T373S	60	210	28.6%	.	.	.
GC22	8	73979596	G	C	SBSPON	Missense	p.H259D	21	119	17.6%	.	.	.
GC22	8	81412122	C	T	ZBTB10	Nonsense	p.R456X	38	149	25.5%	.	.	.
GC22	8	99961253	C	T	OSR2	Nonsense	p.Q25X	24	70	34.3%	.	.	.
GC22	8	126448477	A	G	TRIB1	Missense	p.K129E	65	182	35.7%	.	.	.
GC22	8	133854842	-	A	PHF20L1	Frameshift	p.K798fs	45	147	30.6%	.	.	.
GC22	8	144940749	A	C	EPPK1	Missense	p.C2225G	12	265	4.5%	.	.	.
GC22	9	14113024	C	A	NFIB	Missense	p.D229Y	16	51	31.4%	.	.	.
GC22	9	15447195	G	A	SNAPC3	Missense	p.G229S	28	108	25.9%	.	.	.
GC22	9	18950845	G	C	SAXO1	Missense	p.F43L	15	135	11.1%	.	.	.

GC22	9	19049740	T	A	RRAGA	Missense	p.F28Y	51	146	34.9%	.	.	.
GC22	9	33986833	T	C	UBAP2	Missense	p.R149G	32	95	33.7%	.	.	.
GC22	9	82333765	T	C	TLE4	Missense	p.V396A	12	30	40.0%	.	.	.
GC22	9	95009806	A	G	IARS	Missense	p.S882P	24	151	15.9%	.	.	.
GC22	9	125391771	-	A	OR1B1	Frameshift	p.L15fs	144	234	61.5%	.	.	.
GC22	9	125909274	G	A	STRBP	Missense	p.P386S	35	107	32.7%	.	.	.
GC22	9	130674697	C	T	ST6GALNA C4	Missense	p.G70D	46	115	40.0%	.	.	.
GC22	9	134070640	T	A	NUP214	Missense	p.I53K	33	126	26.2%	.	.	.
GC22	9	135277154	C	G	TTF1	Missense	p.S352T	23	224	10.3%	.	.	.
GC22	9	136379780	G	A	MYMK	Missense	p.T215I	24	80	30.0%	.	.	.
GC22	9	140123320	G	C	RNF224	Missense	p.D85H	7	56	12.5%	.	.	.
GC22	10	27687535	-	T	PTCHD3	Frameshift	p.K664fs	92	174	52.9%	.	.	.
GC22	10	70947457	-	T	SUPV3L1	Frameshift	p.L42fs	55	180	30.6%	.	.	.
GC22	10	120809407	T	A	EIF3A	Missense	p.E855V	84	227	37.0%	.	.	.
GC22	11	704597	A	G	TMEM80	Missense	p.Q268R	28	90	31.1%	.	.	.
GC22	11	6567219	A	G	DNHD1	Missense	p.T1684A	17	87	19.5%	.	.	.
GC22	11	7111391	A	T	RBMXL2	Missense	p.D347V	28	110	25.5%	.	.	.
GC22	11	30352754	A	-	ARL14EP	Frameshift	p.K87fs	57	174	32.8%	.	O	.
GC22	11	34515214	T	G	ELF5	Missense	p.K56T	18	106	17.0%	.	.	.
GC22	11	44940810	G	T	TSPAN18	Missense	p.G127C	38	112	33.9%	.	.	.
GC22	11	55340393	C	A	OR4C16	Missense	p.P264T	78	151	51.7%	.	.	.
GC22	11	58170407	C	A	OR5B3	Missense	p.G159V	7	194	3.6%	.	O	.
GC22	11	62562465	G	A	NXF1	Missense	p.R507W	8	223	3.6%	.	.	.
GC22	11	64577170	C	T	MEN1	Missense	p.A138T	54	157	34.4%	O	.	.
GC22	11	77413116	T	-	RSF1	Frameshift	p.K386fs	46	131	35.1%	.	O	.
GC22	11	112064647	A	T	BCO2	Missense	p.D83V	29	98	29.6%	.	.	.
GC22	11	128651852	G	T	FLI1	Splicing	c.590-1G>T;NM_001271010	9	100	9.0%	.	.	.
GC22	12	8673845	T	A	CLEC4D	Missense	p.I209K	32	105	30.5%	.	.	.
GC22	12	9317935	T	C	PZP	Missense	p.T763A	34	139	24.5%	.	.	.
GC22	12	26592096	G	T	ITPR2	Missense	p.L2203I	42	152	27.6%	.	.	.

GC22	12	46602878	A	G	SLC38A1	Missense	p.I116T	37	97	38.1%	.	.	.
GC22	12	48596876	-	A	OR10AD1	Frameshift	p.L67fs	181	279	64.9%	.	.	.
GC22	12	49445469	G	T	KMT2D	Missense	p.P666H	27	84	32.1%	O	.	.
GC22	12	53045627	-	GCTGCCGCC TCCAAAGCC	KRT2	Nonframeshift	p.S100delinsR ALEAAA	34	65	52.3%	.	.	.
GC22	12	53819280	G	C	AMHR2	Missense	p.V182L	40	109	36.7%	.	.	.
GC22	12	54925990	A	C	NCKAP1L	Missense	p.I890L	31	78	39.7%	.	.	.
GC22	12	55028599	C	A	LACRT	Missense	p.L9F	26	70	37.1%	.	.	.
GC22	12	65564877	T	C	LEMD3	Missense	p.S501P	67	203	33.0%	.	.	.
GC22	12	70938393	A	T	PTPRB	Missense	p.F1505Y	39	141	27.7%	O	.	.
GC22	12	78400610	A	G	NAV3	Missense	p.N431S	20	113	17.7%	.	.	.
GC22	12	95603511	C	T	FGD6	Missense	p.E517K	27	146	18.5%	.	.	.
GC22	12	101779878	A	C	UTP20	Missense	p.K2779Q	16	83	19.3%	.	.	.
GC22	12	109924283	A	T	UBE3B	Missense	p.Y117F	32	88	36.4%	.	.	.
GC22	12	111891541	C	T	ATXN2	Missense	p.A978T	25	91	27.5%	.	.	.
GC22	12	120876266	A	T	COX6A1	Missense	p.H63L	74	230	32.2%	.	.	.
GC22	12	120907315	A	T	SRSF9	Missense	p.L33Q	57	164	34.8%	.	.	.
GC22	12	122691429	G	A	B3GNT4	Missense	p.A186T	32	116	27.6%	.	.	.
GC22	12	123187604	A	T	HCAR2	Missense	p.L76Q	26	80	32.5%	.	.	.
GC22	12	124856638	G	-	NCOR2	Frameshift	p.Q895fs	45	129	34.9%	O	.	.
GC22	13	21742491	C	T	SKA3	Missense	p.E127K	49	158	31.0%	.	.	.
GC22	13	38144790	T	-	POSTN	Frameshift	p.K675fs	33	91	36.3%	.	.	.
GC22	13	48916810	T	C	RB1	Missense	p.S114P	66	190	34.7%	O	.	.
GC22	13	49089392	T	-	RCBTB2	Nonsense	p.I76X	42	139	30.2%	.	O	.
GC22	14	20857778	A	G	TEP1	Missense	p.V711A	20	96	20.8%	.	.	.
GC22	14	21543635	C	T	ARHGEF40	Missense	p.A532V	49	190	25.8%	.	.	.
GC22	14	23991572	T	C	ZFHX2	Missense	p.T2440A	26	102	25.5%	.	.	.
GC22	14	33292100	G	A	AKAP6	Missense	p.S1694N	60	279	21.5%	.	.	.
GC22	14	35783650	T	A	PSMA6	Missense	p.N145K	50	189	26.5%	.	.	.
GC22	14	75583914	C	A	NEK9	Missense	p.R249M	32	183	17.5%	.	.	.
GC22	14	77319649	C	T	LRRC74A	Missense	p.R302C	116	204	56.9%	.	.	.
GC22	14	91747821	A	G	CCDC88C	Missense	p.V1560A	28	125	22.4%	.	.	.

GC22	14	93995636	C	A	UNC79	Missense	p.H353N	53	216	24.5%	.	.	.
GC22	15	25932930	C	A	ATP10A	Nonsense	p.E1071X	14	85	16.5%	.	.	.
GC22	15	25953367	G	A	ATP10A	Missense	p.R809C	44	136	32.4%	.	O	.
GC22	15	41339618	C	T	INO80	Missense	p.G908E	24	56	42.9%	.	.	.
GC22	15	72557484	A	T	PARP6	Missense	p.L89H	34	96	35.4%	.	.	.
GC22	15	75122663	-	A	CPLX3	Frameshift	p.K149fs	44	123	35.8%	.	.	.
GC22	15	75185509	A	G	MPI	Missense	p.D123G	7	52	13.5%	.	.	.
GC22	15	75969068	A	G	CSPG4	Missense	p.M1931T	22	52	42.3%	.	.	.
GC22	15	85383729	C	T	ALPK3	Missense	p.P609S	14	42	33.3%	.	.	.
GC22	15	90904229	C	T	ZNF774	Missense	p.A389V	57	152	37.5%	.	.	.
GC22	15	92397208	C	T	SLCO3A1	Nonsense	p.Q24X	41	119	34.5%	.	.	.
GC22	16	348185	C	T	AXIN1	Missense	p.A441T	12	38	31.6%	O	.	.
GC22	16	21213141	T	C	ZP2	Missense	p.K464E	26	143	18.2%	.	.	.
GC22	16	21987549	A	G	UQCRC2	Missense	p.D370G	20	96	20.8%	.	.	.
GC22	16	29810597	G	A	KIF22	Missense	p.E190K	46	160	28.8%	.	.	.
GC22	16	30002542	C	T	TAOK2	Missense	p.P935S	31	37	83.8%	.	.	.
GC22	16	31121043	G	A	BCKDK	Missense	p.R105H	41	108	38.0%	.	.	.
GC22	16	31770697	-	A	ZNF720	Frameshift	p.X127delinsX	114	195	58.5%	.	.	.
GC22	16	50188066	G	T	PAPD5	Missense	p.Q163H	7	74	9.5%	.	.	.
GC22	16	67914661	G	C	EDC4	Missense	p.D767H	19	76	25.0%	.	.	.
GC22	16	71012887	T	C	HYDIN	Missense	p.Y1523C	25	106	23.6%	.	.	.
GC22	16	72991758	-	C	ZFHX3	Frameshift	p.E763fs	66	177	37.3%	O	.	.
GC22	16	72993668	A	G	ZFHX3	Missense	p.V126A	25	71	35.2%	O	.	.
GC22	16	88501955	C	T	ZNF469	Missense	p.P2665S	10	79	12.7%	.	.	.
GC22	17	4200094	A	-	UBE2G1	Frameshift	p.F55fs	35	86	40.7%	.	.	.
GC22	17	7330230	T	C	C17orf74	Missense	p.L307P	26	103	25.2%	.	.	.
GC22	17	26971123	A	-	KIAA0100	Frameshift	p.W51fs	31	88	35.2%	.	O	.
GC22	17	27251246	G	-	PHF12	Frameshift	p.P132fs	81	215	37.7%	.	.	.
GC22	17	29250076	C	G	ADAP2	Missense	p.D71E	64	196	32.7%	.	.	.
GC22	17	30207595	T	C	UTP6	Missense	p.T322A	145	222	65.3%	.	.	.
GC22	17	34062261	A	G	RASL10B	Missense	p.I20V	21	79	26.6%	.	.	.

GC22	17	39550339	C	A	KRT31	Missense	p.V394F	9	38	23.7%	.	.	.
GC22	17	40720492	TTTTGTA	-	MLX	Frameshift	p.L82fs	26	49	53.1%	.	.	.
GC22	17	40843928	G	A	CNTNAP1	Missense	p.A817T	40	101	39.6%	.	.	.
GC22	17	43181233	C	A	NMT1	Missense	p.L441I	42	121	34.7%	.	.	.
GC22	17	48192979	G	-	SAMD14	Frameshift	p.P257fs	45	134	33.6%	.	.	.
GC22	17	56389513	A	G	TSPOAP1	Missense	p.V830A	16	53	30.2%	.	.	.
GC22	17	58499967	A	G	C17orf64	Missense	p.D5G	7	24	29.2%	.	.	.
GC22	17	78032656	G	T	CCDC40	Splicing	c.1318-1G>T;NM_001330508	23	78	29.5%	.	.	.
GC22	17	80146102	T	A	CCDC57	Missense	p.I349F	10	26	38.5%	.	.	.
GC22	17	80438961	C	A	NARF	Missense	p.L167M	19	134	14.2%	.	.	.
GC22	18	46474795	G	-	SMAD7	Frameshift	p.P209fs	29	83	34.9%	.	O	.
GC22	18	55217961	T	C	FECH	Missense	p.T419A	35	96	36.5%	.	.	.
GC22	19	1796198	C	T	ATP8B3	Missense	p.G560D	26	90	28.9%	.	.	.
GC22	19	2987138	-	G	TLE6	Frameshift	p.W25fs	35	99	35.4%	.	.	.
GC22	19	6753506	C	T	SH2D3A	Missense	p.A511T	25	78	32.1%	.	O	.
GC22	19	10227826	-	G	EIF3G	Frameshift	p.P113fs	22	70	31.4%	.	.	.
GC22	19	10230366	T	C	EIF3G	Missense	p.S11G	39	136	28.7%	.	.	.
GC22	19	12186287	C	A	ZNF844	Missense	p.L118I	52	168	31.0%	.	.	.
GC22	19	12224150	T	-	ZNF788	Frameshift	p.S596fs	55	172	32.0%	.	.	.
GC22	19	14952569	C	A	OR7A10	Missense	p.G41W	5	96	5.2%	.	.	.
GC22	19	17627053	G	T	PGLS	Missense	p.E120D	6	45	13.3%	.	.	.
GC22	19	18960950	G	-	UPF1	Frameshift	p.L176fs	38	108	35.2%	.	.	.
GC22	19	31040124	G	A	ZNF536	Missense	p.D1200N	49	150	32.7%	.	.	.
GC22	19	36940069	C	G	ZNF566	Missense	p.R252T	14	78	17.9%	.	.	.
GC22	19	37382758	A	T	ZNF829	Missense	p.I312N	32	109	29.4%	.	.	.
GC22	19	37975675	G	A	ZNF570	Missense	p.R342K	6	130	4.6%	.	O	.
GC22	19	38996976	C	-	RYR1	Frameshift	p.P2859fs	28	90	31.1%	.	.	.
GC22	19	39230774	A	G	CAPN12	Missense	p.Y216H	16	45	35.6%	.	.	.
GC22	19	40357568	C	-	FCGBP	Frameshift	p.A5249fs	52	168	31.0%	.	.	.
GC22	19	40902740	C	T	PRX	Missense	p.V507M	29	102	28.4%	.	.	.
GC22	19	44096985	C	A	IRGQ	Missense	p.K355N	97	270	35.9%	.	.	.

GC22	19	49442850	-	G	DHDH	Frameshift	p.G171fs	102	126	81.0%	.	.	.
GC22	19	50435804	G	A	ATF5	Missense	p.A102T	17	32	53.1%	.	.	.
GC22	19	51645844	G	A	SIGLEC7	Missense	p.S73N	47	147	32.0%	.	.	.
GC22	19	52004792	-	C	SIGLEC12	Frameshift	p.A66fs	93	189	49.2%	.	.	.
GC22	19	55526104	-	CAGA	GP6	Frameshift	p.L403fs	77	210	36.7%	.	.	.
GC22	19	59028805	G	T	ZBTB45	Missense	p.T79K	44	144	30.6%	.	.	.
GC22	20	18794615	G	T	SCP2D1	Missense	p.R52S	22	173	12.7%	.	O	.
GC22	20	31041483	C	G	NOL4L	Missense	p.A157P	36	107	33.6%	.	.	.
GC22	20	35421908	C	T	SOGA1	Missense	p.G1526D	51	169	30.2%	.	O	.
GC22	20	36640999	T	A	TTI1	Missense	p.Y407F	55	190	28.9%	.	.	.
GC22	20	43036077	G	T	HNF4A	Missense	p.R116M	16	53	30.2%	.	.	.
GC22	20	44506629	C	T	ZSWIM3	Nonsense	p.Q478X	14	169	8.3%	.	.	.
GC22	20	48500416	T	C	SLC9A8	Missense	p.L451P	19	55	34.5%	.	.	.
GC22	20	50255893	T	C	ATP9A	Missense	p.I553V	83	138	60.1%	.	.	.
GC22	20	54824540	C	T	MC3R	Missense	p.A214V	25	148	16.9%	.	.	.
GC22	20	54963248	G	C	AURKA	Missense	p.D2E	23	164	14.0%	.	.	.
GC22	20	61940691	A	G	COL20A1	Missense	p.K316R	8	45	17.8%	.	.	.
GC22	21	43711642	A	G	ABCG1	Missense	p.Y522C	50	148	33.8%	.	.	.
GC22	22	26242204	C	T	MYO18B	Missense	p.A1170V	21	57	36.8%	.	.	.
GC22	22	26247515	T	C	MYO18B	Missense	p.M1286T	49	170	28.8%	.	.	.
GC22	22	29885568	-	AAGTCCCCTGAGAAGGCC	NEFH	Nonsense	p.K647delinsKVPX	26	57	45.6%	.	.	.
GC22	22	37326777	G	A	CSF2RB	Missense	p.C306Y	43	121	35.5%	.	.	.
GC22	22	39132297	C	A	SUN2	Missense	p.R731I	32	93	34.4%	.	.	.
GC22	22	40055768	T	C	CACNA1I	Missense	p.S804P	26	90	28.9%	.	.	.
GC22	22	45732267	A	-	FAM118A	Frameshift	p.S269fs	28	61	45.9%	.	.	.
GC22	X	12939470	G	-	TLR8	Frameshift	p.G771fs	113	240	47.1%	.	.	.
GC22	X	35985745	C	A	CFAP47	Missense	p.P537H	19	43	44.2%	.	O	.
GC22	X	35993324	A	G	CFAP47	Missense	p.H772R	25	53	47.2%	.	.	.
GC22	X	46491052	G	T	SLC9A7	Missense	p.P570H	33	65	50.8%	.	O	.
GC22	X	47920299	G	A	ZNF630	Missense	p.A14V	41	88	46.6%	.	.	.
GC22	X	70469315	T	G	ZMYM3	Missense	p.K489T	11	131	8.4%	.	.	.

GC22	X	70617264	G	A	TAF1	Missense	p.A1210T	85	153	55.6%	.	.	.
GC22	X	70774430	G	A	OGT	Missense	p.R239H	52	104	50.0%	.	O	.
GC22	X	114425278	A	C	RBMXL3	Missense	p.E425A	30	66	45.5%	.	.	.
GC22	X	123210247	G	A	STAG2	Missense	p.A867T	39	87	44.8%	O	.	.
GC22	X	133378875	T	-	CCDC160	Frameshift	p.P15fs	28	76	36.8%	.	O	.
GC22	X	140270995	G	A	LDOC1	Missense	p.T71M	29	55	52.7%	.	O	.
GC23	1	11102958	C	T	MASP2	Missense	p.G288D	8	72	11.1%	.	.	.
GC23	1	92647615	T	C	KIAA1107	Missense	p.F946L	73	104	70.2%	.	.	.
GC23	1	152084547	-	CTC	TCHH	Nonframeshift	p.Q382delinsQR	30	60	50.0%	.	.	.
GC23	1	159900577	C	G	IGSF9	Missense	p.G573A	15	52	28.8%	.	.	.
GC23	1	204438248	T	C	PIK3C2B	Missense	p.Y228C	127	220	57.7%	.	.	.
GC23	1	248525329	-	TA	OR2T4	Frameshift	p.T149fs	44	104	42.3%	.	.	.
GC23	2	28828802	-	T	PLB1	Frameshift	p.L1004fs	45	140	32.1%	.	.	.
GC23	2	71650749	C	G	ZNF638	Missense	p.Q1369E	80	148	54.1%	.	.	.
GC23	2	109527498	G	A	EDAR	Missense	p.A187V	15	116	12.9%	.	.	.
GC23	2	168041073	C	G	XIRP2	Missense	p.A195G	10	98	10.2%	.	.	.
GC23	2	219140248	T	C	TMBIM1	Missense	p.I185V	78	133	58.6%	.	.	.
GC23	2	236761394	C	-	AGAP1	Frameshift	p.A372fs	71	158	44.9%	.	.	.
GC23	2	241465167	G	A	ANKMY1	Missense	p.L194F	9	101	8.9%	.	.	.
GC23	3	9798484	C	T	OGG1	Missense	p.A311V	22	55	40.0%	.	.	.
GC23	3	39230108	G	T	XIRP1	Missense	p.P277T	6	97	6.2%	.	.	.
GC23	3	73111482	-	A	EBLN2	Frameshift	p.R84fs	90	202	44.6%	.	O	.
GC23	3	132411562	T	C	NPHP3	Missense	p.H804R	14	151	9.3%	.	.	.
GC23	4	6864334	T	C	KIAA0232	Missense	p.I742T	9	90	10.0%	.	.	.
GC23	4	10444739	T	G	ZNF518B	Missense	p.K1072Q	9	91	9.9%	.	.	.
GC23	4	80329103	G	T	GK2	Missense	p.N84K	5	96	5.2%	.	.	.
GC23	4	85642639	C	T	WDFY3	Missense	p.A2510T	6	131	4.6%	.	.	.
GC23	4	110370660	G	T	SEC24B	Missense	p.S74I	8	100	8.0%	.	.	.
GC23	4	151829594	G	C	LRBA	Missense	p.S462C	24	116	20.7%	.	O	.
GC23	5	35861055	C	G	IL7R	Missense	p.P62A	54	105	51.4%	O	.	.
GC23	5	36976177	A	T	NIPBL	Missense	p.I390F	79	159	49.7%	.	.	.

GC23	5	37064909	C	T	NIPBL	Missense	p.A2777V	41	78	52.6%	.	.	.
GC23	5	54529090	-	GGGCC	CCNO	Frameshift	p.Q88fs	20	66	30.3%	.	.	.
GC23	5	78421961	C	A	BHMT	Missense	p.L240M	9	137	6.6%	.	.	.
GC23	5	101597654	A	G	SLCO4C1	Missense	p.L328S	19	115	16.5%	.	O	.
GC23	5	137680912	T	C	FAM53C	Missense	p.S179P	44	127	34.6%	.	.	.
GC23	5	152871789	-	C	GRIA1	Frameshift	p.L11fs	100	284	35.2%	.	.	.
GC23	5	154394982	G	C	KIF4B	Missense	p.Q521H	96	263	36.5%	.	.	.
GC23	7	20782519	T	C	ABCB5	Missense	p.L570S	45	110	40.9%	.	.	.
GC23	7	89887443	T	G	CFAP69	Missense	p.V71G	8	60	13.3%	.	.	.
GC23	7	96115626	C	G	SEM1	Missense	p.R39P	8	100	8.0%	.	.	.
GC23	7	99077367	C	G	ZNF789	Nonsense	p.Y19X	13	163	8.0%	.	.	.
GC23	7	101921332	G	T	CUX1	Missense	p.G543V	6	70	8.6%	O	.	.
GC23	7	139798731	C	A	KDM7A	Missense	p.E622D	37	75	49.3%	.	.	.
GC23	7	150938681	C	G	SMARCD3	Missense	p.S279T	30	73	41.1%	.	.	.
GC23	8	10480296	-	G	RP1L1	Frameshift	p.P139fs	25	48	52.1%	.	.	.
GC23	8	104897566	T	A	RIMS2	Missense	p.Y55N	10	70	14.3%	.	.	.
GC23	8	139165205	C	A	FAM135B	Missense	p.G505C	15	86	17.4%	.	.	.
GC23	8	143746836	G	C	JRK	Missense	p.D214E	14	43	32.6%	.	.	.
GC23	9	14125706	G	A	NFIB	Missense	p.P77S	31	72	43.1%	.	.	.
GC23	9	125391771	-	A	OR1B1	Frameshift	p.L15fs	98	181	54.1%	.	.	.
GC23	9	131477658	T	C	PKN3	Missense	p.V576A	19	46	41.3%	.	.	.
GC23	10	48370812	C	T	ZNF488	Missense	p.R94C	22	47	46.8%	.	.	.
GC23	10	102267275	A	G	SEC31B	Missense	p.V230A	43	92	46.7%	.	.	.
GC23	11	556570	G	A	LMNTD2	Missense	p.S332L	30	37	81.1%	.	.	.
GC23	11	1092847	C	G	MUC2	Missense	p.Q1556E	7	154	4.5%	.	.	.
GC23	11	19259442	CAAA	-	E2F8	Frameshift	p.F84fs	181	260	69.6%	.	.	.
GC23	11	66472378	C	T	SPTBN2	Missense	p.R790Q	24	42	57.1%	.	O	.
GC23	11	71723485	C	G	NUMA1	Missense	p.E1538Q	32	67	47.8%	.	.	.
GC23	11	92901210	A	G	SLC36A4	Missense	p.L88S	8	71	11.3%	.	.	.
GC23	12	8374782	-	ACG	FAM90A1	Nonframeshift	p.T344delinsTS	25	41	61.0%	.	.	.
GC23	12	20864396	C	G	SLCO1C1	Missense	p.Q43E	9	83	10.8%	.	.	.

GC23	12	56872836	T	G	GLS2	Missense	p.K178N	14	88	15.9%	.	.	.
GC23	12	106773903	G	A	POLR3B	Missense	p.V179I	28	85	32.9%	.	.	.
GC23	12	110385192	G	T	GIT2	Missense	p.R453S	41	125	32.8%	.	.	.
GC23	12	121880356	T	G	KDM2B	Missense	p.N894T	5	93	5.4%	.	.	.
GC23	14	24809020	T	G	RIPK3	Missense	p.K5Q	29	55	52.7%	.	.	.
GC23	15	63620314	G	T	CA12	Missense	p.A297E	63	106	59.4%	.	O	.
GC23	15	63972938	G	A	HERC1	Missense	p.T2088M	64	107	59.8%	.	.	.
GC23	15	64017505	T	A	HERC1	Missense	p.D1185V	12	125	9.6%	.	.	.
GC23	16	2523456	C	A	NTN3	Missense	p.R449S	15	94	16.0%	.	.	.
GC23	16	21272626	T	A	CRYM	Nonsense	p.K277X	6	88	6.8%	.	.	.
GC23	17	10350437	C	T	MYH4	Missense	p.E1688K	58	119	48.7%	.	.	.
GC23	18	10704642	T	C	PIEZO2	Missense	p.H1890R	84	184	45.7%	.	.	.
GC23	19	1510247	-	G	ADAMTSL5	Frameshift	p.P88fs	33	85	38.8%	.	.	.
GC23	19	4511763	C	T	PLIN4	Missense	p.V723M	36	181	19.9%	.	.	.
GC23	19	4652281	C	A	TNFAIP8L1	Missense	p.H134N	45	87	51.7%	.	.	.
GC23	19	39915936	C	A	PLEKHG2	Missense	p.P1211T	7	102	6.9%	.	.	.
GC23	19	50945567	G	C	MYBPC2	Missense	p.S300T	42	70	60.0%	.	.	.
GC23	19	52004792	-	C	SIGLEC12	Frameshift	p.A66fs	55	179	30.7%	.	.	.
GC23	19	52084691	-	G	ZNF175	Frameshift	p.E40fs	50	131	38.2%	.	.	.
GC23	19	55085981	A	G	LILRA2	Missense	p.Y83C	9	132	6.8%	.	.	.
GC23	20	1546855	-	C	SIRPB1	Frameshift	p.L164fs	42	108	38.9%	.	.	.
GC23	20	8862280	G	C	PLCB1	Missense	p.E1145D	39	83	47.0%	.	O	.
GC23	20	30438476	C	T	DUSP15	Missense	p.A144T	33	67	49.3%	.	.	.
GC23	20	33330455	G	A	NCOA6	Missense	p.A1202V	27	77	35.1%	.	O	.
GC23	20	43851325	C	T	SEMG2	Missense	p.S351L	14	149	9.4%	.	.	.
GC23	20	43929777	C	T	MATN4	Missense	p.G244D	44	100	44.0%	.	.	.
GC23	21	34926299	G	T	SON	Missense	p.D1588Y	55	118	46.6%	.	.	.
GC23	21	34927515	G	A	SON	Missense	p.R1993H	45	112	40.2%	.	O	.
GC23	22	29885568	-	AAGTCCCCTGAGAAGGCC	NEFH	Nonsense	p.K647delinsKVPX	29	91	31.9%	.	.	.
GC23	22	50191596	G	A	BRD1	Missense	p.A647V	16	167	9.6%	.	.	.
GC23	X	41333851	C	A	NYX	Missense	p.S382Y	7	40	17.5%	.	.	.

GC24	1	12170214	G	A	TNFRSF8	Missense	p.R99K	5	98	5.1%	.	.	.
GC24	1	17914014	G	T	ARHGEF10L	Nonsense	p.E33X	11	79	13.9%	.	.	.
GC24	1	23966895	-	AAG	MDS2	Nonframeshift	p.V93delinsVK	34	92	37.0%	.	.	.
GC24	1	32681065	C	T	DCDC2B	Missense	p.P292L	14	51	27.5%	.	.	.
GC24	1	36299676	G	A	AGO4	Missense	p.G489S	10	48	20.8%	.	.	.
GC24	1	40769613	T	C	COL9A2	Missense	p.E452G	16	45	35.6%	.	.	.
GC24	1	55251232	T	C	TTC22	Missense	p.E369G	3	22	13.6%	.	.	.
GC24	1	62740408	C	A	KANK4	Missense	p.S123I	11	48	22.9%	.	.	.
GC24	1	78327005	G	A	MIGA1	Missense	p.G458R	9	149	6.0%	.	.	.
GC24	1	85487770	C	T	MCOLN3	Missense	p.G379R	6	73	8.2%	.	.	.
GC24	1	91403943	C	A	ZNF644	Nonsense	p.G990X	9	175	5.1%	.	.	.
GC24	1	94502907	C	G	ABCA4	Splicing	c.3608-1G>C	7	92	7.6%	.	.	.
GC24	1	98015214	C	A	DPYD	Missense	p.V476L	26	209	12.4%	.	.	.
GC24	1	111145955	A	T	KCNA2	Missense	p.L484M	15	224	6.7%	.	.	.
GC24	1	152059034	T	G	TCHHL1	Missense	p.Q375P	16	124	12.9%	.	.	.
GC24	1	152059035	G	T	TCHHL1	Missense	p.Q375K	17	121	14.0%	.	.	.
GC24	1	153177359	T	A	LELP1	Missense	p.L59Q	25	141	17.7%	.	.	.
GC24	1	153665647	G	A	NPR1	Missense	p.E1033K	14	46	30.4%	.	.	.
GC24	1	154296870	C	A	AQP10	Missense	p.P274T	6	85	7.1%	.	.	.
GC24	1	158326623	A	C	CD1E	Missense	p.Q167H	8	130	6.2%	.	.	.
GC24	1	160535386	C	T	CD84	Missense	p.G66R	7	182	3.8%	.	.	.
GC24	1	168012363	G	A	DCAF6	Missense	p.G386R	28	169	16.6%	.	.	.
GC24	1	190067812	A	C	BRINP3	Missense	p.L444R	19	186	10.2%	.	.	.
GC24	1	197479823	C	T	DENND1B	Missense	p.G699R	13	213	6.1%	.	.	.
GC24	1	200817819	A	G	CAMSAP2	Missense	p.D625G	43	442	9.7%	.	.	.
GC24	1	201181918	G	A	IGFN1	Missense	p.G2633R	10	166	6.0%	.	.	.
GC24	1	204403709	C	T	PIK3C2B	Missense	p.A1182T	15	191	7.9%	.	O	.
GC24	1	207110931	G	A	PIGR	Missense	p.P185L	18	511	3.5%	.	.	.
GC24	1	223284972	T	A	TLR5	Missense	p.T468S	32	72	44.4%	.	.	.
GC24	1	226127137	A	T	LEFTY2	Missense	p.F221I	5	30	16.7%	.	.	.
GC24	1	228403450	A	T	OBSCN	Missense	p.E672V	20	92	21.7%	.	.	.

GC24	1	245848783	T	A	KIF26B	Missense	p.F833Y	15	84	17.9%	.	.	.
GC24	1	248059780	-	A	OR2W3	Frameshift	p.K298fs	91	189	48.1%	.	.	.
GC24	2	11280671	C	A	C2orf50	Missense	p.P98Q	5	77	6.5%	.	.	.
GC24	2	11750909	A	T	GREB1	Missense	p.K921M	13	166	7.8%	.	.	.
GC24	2	26174707	C	A	KIF3C	Missense	p.D653Y	16	74	21.6%	.	.	.
GC24	2	29296395	G	A	C2orf71	Nonsense	p.Q245X	12	70	17.1%	.	.	.
GC24	2	31167767	G	A	GALNT14	Missense	p.P262S	7	111	6.3%	.	O	.
GC24	2	113675281	G	A	IL37	Missense	p.G51E	11	126	8.7%	.	.	.
GC24	2	153533156	-	GCTGCT	PRPF40A	Nonframeshift	p.A265delinsEQP	60	147	40.8%	.	.	.
GC24	2	169825893	C	T	ABCB11	Missense	p.G660R	11	83	13.3%	.	O	.
GC24	2	179416981	G	A	TTN	Missense	p.P21151S	12	146	8.2%	.	.	.
GC24	2	202492051	T	A	TMEM237	Missense	p.S344C	22	198	11.1%	.	.	.
GC24	2	215843080	G	C	ABCA12	Missense	p.D1378E	71	124	57.3%	.	.	.
GC24	2	220361591	-	A	LOC100996693	Frameshift	p.V63fs	35	64	54.7%	.	.	.
GC24	2	223559932	C	A	MOGAT1	Missense	p.P260T	15	124	12.1%	.	.	.
GC24	2	230456534	C	T	DNER	Missense	p.S116N	10	135	7.4%	.	.	.
GC24	2	234602090	T	A	UGT1A6	Missense	p.L147H	15	133	11.3%	.	.	.
GC24	2	234891832	C	A	TRPM8	Missense	p.L909M	13	76	17.1%	.	.	.
GC24	2	241987736	C	A	SNED1	Missense	p.D426E	4	42	9.5%	.	.	.
GC24	3	3084060	G	A	CNTN4	Nonsense	p.W493X	10	194	5.2%	.	.	.
GC24	3	8669418	T	A	SSUH2	Nonsense	p.R214X	10	130	7.7%	.	.	.
GC24	3	33453106	C	T	UBP1	Nonsense	p.W174X	11	88	12.5%	.	.	.
GC24	3	48463794	T	A	PLXNB1	Missense	p.R455S	8	35	22.9%	.	.	.
GC24	3	48699475	C	G	CELSR3	Missense	p.R198T	51	91	56.0%	.	.	.
GC24	3	52643338	C	T	PBRM1	Missense	p.R853K	6	81	7.4%	O	.	.
GC24	3	107885824	C	A	IFT57	Missense	p.L286F	4	41	9.8%	.	.	.
GC24	3	124731989	TTG	-	HEG1	Nonframeshift	p.811_812del	184	358	51.4%	.	.	.
GC24	3	127390292	T	A	PODXL2	Missense	p.Y481N	12	75	16.0%	.	.	.
GC24	3	128856004	G	A	ISY1	Missense	p.L152F	4	49	8.2%	.	.	.
GC24	3	130282482	G	C	COL6A6	Missense	p.G212A	33	139	23.7%	.	.	.
GC24	3	165547602	T	C	BCHE	Missense	p.D407G	28	149	18.8%	.	.	.

GC24	3	193335599	G	A	OPA1	Missense	p.G159R	10	144	6.9%	.	.	.
GC24	4	983738	G	A	SLC26A1	Missense	p.P330L	3	21	14.3%	.	.	.
GC24	4	7435231	G	A	PSAPL1	Missense	p.P459L	21	99	21.2%	.	.	.
GC24	4	24541875	C	A	DHX15	Missense	p.A548S	5	43	11.6%	.	.	.
GC24	4	24801811	G	A	SOD3	Missense	p.R223Q	18	51	35.3%	.	.	.
GC24	4	40351224	C	T	CHRNA9	Missense	p.L231F	10	196	5.1%	.	.	.
GC24	4	88231393	-	A	HSD17B13	Splicing	c.704+2->T	38	79	48.1%	.	.	.
GC24	4	110932390	-	C	EGF	Frameshift	p.P1094fs	38	80	47.5%	.	.	.
GC24	4	122766831	T	A	BBS7	Missense	p.Q353L	7	108	6.5%	.	.	.
GC24	4	151829913	T	C	LRBA	Missense	p.T420A	32	70	45.7%	.	.	.
GC24	4	164506963	G	C	MARCH1	Missense	p.R104G	18	68	26.5%	.	O	.
GC24	4	184366024	A	G	CDKN2AIP	Missense	p.D25G	21	47	44.7%	.	.	.
GC24	4	186611767	T	C	SORBS2	Splicing	.	28	92	30.4%	.	.	.
GC24	4	187549386	C	A	FAT1	Missense	p.G1578C	20	128	15.6%	O	.	.
GC24	5	5306786	C	T	ADAMTS16	Missense	p.P1119L	16	84	19.0%	.	.	.
GC24	5	16675143	T	A	MYO10	Missense	p.T1595S	17	117	14.5%	.	.	.
GC24	5	32263340	G	T	MTMR12	Missense	p.P198T	5	77	6.5%	.	.	.
GC24	5	35875619	A	T	IL7R	Missense	p.K269M	8	122	6.6%	O	.	.
GC24	5	35875620	G	T	IL7R	Missense	p.K269N	8	125	6.4%	O	.	.
GC24	5	35965519	A	C	UGT3A1	Missense	p.L271W	5	36	13.9%	.	.	.
GC24	5	36168467	A	T	SKP2	Missense	p.T197S	19	181	10.5%	.	.	.
GC24	5	36168468	C	T	SKP2	Missense	p.T197I	20	185	10.8%	.	.	.
GC24	5	38933056	A	G	OSMR	Missense	p.K817R	9	55	16.4%	.	.	.
GC24	5	90050880	C	A	ADGRV1	Missense	p.P3820T	8	119	6.7%	.	.	.
GC24	5	95124464	A	T	RHOBTB3	Splicing	c.1624-2A>T	14	113	12.4%	.	.	.
GC24	5	112824049	-	GCC	MCC	Nonframeshift	p.G21delinsGA	35	83	42.2%	.	.	.
GC24	5	141053257	T	A	ARAP3	Missense	p.I273F	5	86	5.8%	.	.	.
GC24	5	147024488	T	A	JAKMIP2	Missense	p.R294S	8	124	6.5%	.	.	.
GC24	5	149011711	G	A	ARHGEF37	Missense	p.R662K	8	63	12.7%	.	.	.
GC24	5	156590485	C	T	FAM71B	Missense	p.R264K	15	120	12.5%	.	O	.
GC24	5	173534501	G	C	HMP19	Missense	p.G170A	4	44	9.1%	.	.	.

GC24	6	30124715	C	A	TRIM10	Splicing	c.895+1G>T	6	68	8.8%	.	.	.
GC24	6	33263858	C	A	RGL2	Missense	p.V157F	8	120	6.7%	.	.	.
GC24	6	83075817	G	A	TPBG	Missense	p.G380E	15	147	10.2%	.	.	.
GC24	6	109703562	G	A	CD164	Missense	p.S7L	59	107	55.1%	.	.	.
GC24	6	112499428	G	A	LAMA4	Nonsense	p.Q362X	11	127	8.7%	.	.	.
GC24	6	126199472	G	T	NCOA7	Missense	p.V35L	43	121	35.5%	.	.	.
GC24	6	127768482	C	A	KIAA0408	Missense	p.G328W	10	139	7.2%	.	.	.
GC24	7	1477735	C	A	MICALL2	Missense	p.G770V	4	36	11.1%	.	.	.
GC24	7	2611828	A	G	IQCE	Missense	p.S23G	96	167	57.5%	.	.	.
GC24	7	18687613	T	A	HDAC9	Missense	p.L334H	11	151	7.3%	.	.	.
GC24	7	48467383	A	T	ABCA13	Missense	p.Q4160H	13	98	13.3%	.	.	.
GC24	7	76062805	A	G	ZP3	Missense	p.K185R	15	39	38.5%	.	.	.
GC24	7	80300418	-	A	CD36	Frameshift	p.E276fs	116	194	59.8%	.	.	.
GC24	7	100006202	G	A	ZCWPW1	Missense	p.P318S	8	89	9.0%	.	.	.
GC24	7	101801865	A	G	CUX1	Missense	p.T229A	16	99	16.2%	O	.	.
GC24	7	116146124	G	C	CAV2	Missense	p.E84Q	8	168	4.8%	.	.	.
GC24	7	124386740	T	C	GPR37	Missense	p.M561V	8	187	4.3%	.	.	.
GC24	7	132193005	C	T	PLXNA4	Missense	p.G150R	17	117	14.5%	.	.	.
GC24	7	151705028	G	T	GALNTL5	Missense	p.R342M	15	150	10.0%	.	.	.
GC24	8	10466021	C	G	RP1L1	Missense	p.A1863P	10	97	10.3%	.	.	.
GC24	8	24813510	G	T	NEFL	Missense	p.L174M	15	53	28.3%	.	.	.
GC24	8	27824015	C	T	SCARA5	Missense	p.G53R	14	69	20.3%	.	O	.
GC24	8	42163903	G	T	IKBKB	Missense	p.D172Y	11	92	12.0%	O	.	.
GC24	8	53574279	C	T	RB1CC1	Missense	p.G392R	5	80	6.3%	.	O	.
GC24	8	103293724	C	A	UBR5	Missense	p.R1907L	6	96	6.3%	O	.	.
GC24	8	110099899	C	A	TRHR	Missense	p.T53N	10	133	7.5%	.	.	.
GC24	8	135533158	C	A	ZFAT	Missense	p.V1006L	10	165	6.1%	.	.	.
GC24	8	135622779	T	A	ZFAT	Nonsense	p.R178X	21	148	14.2%	.	.	.
GC24	8	139774678	T	G	COL22A1	Missense	p.K612T	8	68	11.8%	.	.	.
GC24	8	143960500	G	A	CYP11B1	Missense	p.P115S	17	107	15.9%	.	.	.
GC24	8	144892972	G	T	SCRIB	Missense	p.Q430K	12	85	14.1%	.	.	.

GC24	8	145161300	G	T	MAF1	Missense	p.D145Y	13	126	10.3%	.	.	.
GC24	9	371451	T	C	DOCK8	Missense	p.V563A	68	100	68.0%	.	.	.
GC24	9	100070358	G	A	CCDC180	Missense	p.G49R	16	98	16.3%	.	.	.
GC24	9	100372652	T	G	TSTD2	Missense	p.K271Q	60	111	54.1%	.	.	.
GC24	9	113169583	G	A	SVEP1	Missense	p.A2766V	37	151	24.5%	.	.	.
GC24	9	123367581	G	T	MEGF9	Missense	p.H566N	64	147	43.5%	.	.	.
GC24	9	132662331	C	A	FNBP1	Missense	p.V534L	16	139	11.5%	.	.	.
GC24	9	139235480	C	T	GPSM1	Missense	p.L413F	20	38	52.6%	.	.	.
GC24	10	27352964	C	T	ANKRD26	Missense	p.G439E	11	84	13.1%	.	.	.
GC24	10	55587198	-	GGCGGC	PCDH15	Nonframeshift	p.P1370delins RRP	25	50	50.0%	.	.	.
GC24	10	78708944	C	A	KCNMA1	Nonsense	p.E781X	23	147	15.6%	.	.	.
GC24	10	99667803	C	A	CRTAC1	Missense	p.D273Y	40	100	40.0%	.	.	.
GC24	10	101977752	C	A	CHUK	Missense	p.K311N	5	81	6.2%	.	.	.
GC24	10	105107055	C	T	PCGF6	Splicing	c.614-1G>A	4	34	11.8%	.	.	.
GC24	10	115389426	T	G	NRAP	Missense	p.K619T	15	79	19.0%	.	.	.
GC24	10	135000027	G	A	KNDC1	Missense	p.S392N	30	58	51.7%	.	.	.
GC24	10	135044764	A	T	UTF1	Missense	p.T282S	4	43	9.3%	.	.	.
GC24	11	4842750	T	G	OR51F2	Missense	p.F45L	13	259	5.0%	.	.	.
GC24	11	5799458	C	A	OR52N5	Missense	p.C136F	30	148	20.3%	.	O	.
GC24	11	5878068	G	A	OR52E8	Missense	p.P289S	13	100	13.0%	.	O	.
GC24	11	18332332	C	G	HPS5	Missense	p.G31R	20	158	12.7%	.	.	.
GC24	11	27722549	C	A	BDNF	Missense	p.R12L	6	85	7.1%	.	.	.
GC24	11	56380636	G	T	OR5M1	Missense	p.L115I	5	93	5.4%	.	.	.
GC24	11	59210718	T	C	OR5A1	Missense	p.L26P	7	131	5.3%	.	.	.
GC24	11	62189762	A	G	SCGB1A1	Missense	p.Y42C	70	118	59.3%	.	.	.
GC24	11	66488563	G	A	SPTBN2	Missense	p.A50V	7	116	6.0%	.	.	.
GC24	11	70332701	C	T	SHANK2	Missense	p.G645R	10	85	11.8%	.	O	.
GC24	11	70336380	C	A	SHANK2	Splicing	c.2360+1G>T	9	76	11.8%	.	.	.
GC24	11	73020376	-	CTC	ARHGEF17	Nonframeshift	p.C231delinsC L	23	56	41.1%	.	.	.
GC24	11	84245620	G	A	DLG2	Missense	p.P66L	12	171	7.0%	.	O	.
GC24	11	101762080	-	T	ANGPTL5	Frameshift	p.N366fs	167	300	55.7%	.	.	.

GC24	11	114568771	C	A	NXPE2	Nonsense	p.S46X	5	46	10.9%	.	.	.
GC24	11	121016483	C	A	TECTA	Missense	p.P1255T	9	197	4.6%	.	.	.
GC24	11	128839691	A	T	ARHGAP32	Missense	p.L1443Q	8	210	3.8%	.	.	.
GC24	12	6155967	A	G	VWF	Missense	p.F735L	68	139	48.9%	.	.	.
GC24	12	7473384	C	A	ACSM4	Missense	p.Q329K	17	125	13.6%	.	.	.
GC24	12	20833102	C	T	PDE3A	Missense	p.P786L	6	124	4.8%	.	.	.
GC24	12	27845780	A	T	PPFIBP1	Missense	p.S848C	6	33	18.2%	.	.	.
GC24	12	29617575	C	G	OVCH1	Missense	p.D664H	8	124	6.5%	.	O	.
GC24	12	40748244	G	C	LRRK2	Missense	p.K2240N	5	84	6.0%	.	.	.
GC24	12	53453557	G	T	TNS2	Missense	p.R721M	7	35	20.0%	.	.	.
GC24	12	57960908	G	T	KIF5A	Splicing	c.502-1G>T	11	95	11.6%	.	.	.
GC24	12	91371990	G	A	EPYC	Missense	p.P72L	4	41	9.8%	.	.	.
GC24	12	97112279	G	T	CFAP54	Missense	p.E2261D	16	80	20.0%	.	.	.
GC24	12	123800138	T	C	SBNO1	Missense	p.E1002G	11	174	6.3%	.	.	.
GC24	13	24871853	T	G	SPATA13	Missense	p.V423G	4	68	5.9%	.	.	.
GC24	13	49688847	C	A	FNDC3A	Missense	p.P22T	8	124	6.5%	.	.	.
GC24	13	77651418	T	A	MYCBP2	Missense	p.R3863S	11	92	12.0%	.	.	.
GC24	13	103275273	G	T	TPP2	Missense	p.V223L	13	61	21.3%	.	.	.
GC24	13	108922423	C	A	TNFSF13B	Nonsense	p.C60X	12	72	16.7%	.	.	.
GC24	14	21424199	C	T	RNASE2	Missense	p.P90L	9	146	6.2%	.	.	.
GC24	14	23374332	C	T	RBM23	Missense	p.G199R	11	128	8.6%	.	.	.
GC24	14	102477186	G	A	DYNC1H1	Missense	p.R2172K	19	79	24.1%	.	.	.
GC24	15	42439888	C	A	PLA2G4F	Missense	p.G378W	9	55	16.4%	.	.	.
GC24	15	51984480	A	G	SCG3	Missense	p.E40G	5	38	13.2%	.	.	.
GC24	15	67430373	C	A	SMAD3	Nonsense	p.C3X	12	90	13.3%	O	.	.
GC24	15	69739255	G	T	KIF23	Nonsense	p.G754X	12	95	12.6%	.	.	.
GC24	15	80847455	G	A	ARNT2	Missense	p.S380N	9	61	14.8%	.	.	.
GC24	15	89401502	G	T	ACAN	Missense	p.G1896C	7	110	6.4%	.	.	.
GC24	15	96880655	G	A	NR2F2	Missense	p.R217K	23	169	13.6%	.	.	.
GC24	16	424253	A	G	TMEM8A	Missense	p.S575P	7	68	10.3%	.	.	.
GC24	16	3119298	-	G	IL32	Frameshift	p.R161fs	78	223	35.0%	.	O	.

GC24	16	29820957	T	G	MAZ	Missense	p.V459G	6	32	18.8%	.	.	.
GC24	16	30370512	C	T	TBC1D10B	Nonsense	p.W541X	10	72	13.9%	.	.	.
GC24	16	53301847	G	A	CHD9	Missense	p.R1509Q	7	50	14.0%	.	.	.
GC24	16	53720346	T	A	RPGRIP1L	Missense	p.R259W	15	58	25.9%	.	.	.
GC24	16	57060539	C	T	NLRC5	Missense	p.P562S	6	105	5.7%	.	.	.
GC24	16	66972125	G	C	CES2	Missense	p.G152R	17	89	19.1%	.	.	.
GC24	16	67860643	G	T	TSNAXIP1	Missense	p.G138W	23	155	14.8%	.	.	.
GC24	16	72821594	-	GCCGCC	ZFHX3	Nonframeshift	p.G2613delins GAA	79	221	35.7%	O	.	.
GC24	16	87637078	G	A	JPH3	Missense	p.G109E	8	39	20.5%	.	.	.
GC24	17	7106636	C	T	DLG4	Missense	p.S113N	19	94	20.2%	.	.	.
GC24	17	7637799	C	T	DNAH2	Missense	p.H251Y	9	76	11.8%	.	.	.
GC24	17	10427940	A	T	MYH2	Missense	p.L1673Q	16	94	17.0%	.	.	.
GC24	17	32964355	G	A	TMEM132E	Missense	p.E777K	15	83	18.1%	.	.	.
GC24	17	36623266	A	G	ARHGAP23	Missense	p.T448A	57	104	54.8%	.	.	.
GC24	17	40049424	T	A	ACLY	Missense	p.K532M	9	62	14.5%	.	.	.
GC24	17	40328176	A	G	KCNH4	Missense	p.V242A	11	131	8.4%	.	.	.
GC24	17	41342724	G	A	NBR1	Missense	p.G244D	8	146	5.5%	.	.	.
GC24	17	45367573	G	A	ITGB3	Missense	p.G353E	16	120	13.3%	.	.	.
GC24	17	46673860	C	T	HOXB6	Missense	p.R197Q	6	142	4.2%	.	.	.
GC24	17	79632258	G	C	OXLD1	Missense	p.I139M	60	95	63.2%	.	.	.
GC24	18	43459127	G	C	EPG5	Missense	p.S1907C	17	24	70.8%	.	.	.
GC24	18	44118253	C	A	LOXHD1	Missense	p.G114W	14	118	11.9%	.	.	.
GC24	18	72247484	C	A	CNDP1	Missense	p.A429E	7	66	10.6%	.	.	.
GC24	19	1122492	C	A	SBNO2	Missense	p.G270V	5	96	5.2%	.	.	.
GC24	19	1122493	C	A	SBNO2	Missense	p.G270C	5	96	5.2%	.	.	.
GC24	19	11556280	G	C	PRKCSH	Missense	p.M225I	28	72	38.9%	.	.	.
GC24	19	17746888	G	A	UNC13A	Missense	p.L1054F	5	81	6.2%	.	.	.
GC24	19	18466771	C	A	PGPEP1	Missense	p.Q52K	23	119	19.3%	.	.	.
GC24	19	42221387	A	T	CEACAM5	Missense	p.K324N	30	164	18.3%	.	.	.
GC24	19	47152124	G	A	DACT3	Missense	p.P277L	7	39	17.9%	.	.	.
GC24	19	50060471	C	G	NOSIP	Missense	p.E98D	6	24	25.0%	.	.	.

GC24	19	50161567	C	A	SCAF1	Missense	p.P1284T	6	46	13.0%	.	.	.
GC24	19	51455899	T	G	KLK5	Missense	p.R5S	7	42	16.7%	.	.	.
GC24	19	57723595	G	A	ZNF264	Missense	p.G377E	6	149	4.0%	.	.	.
GC24	20	31022902	G	T	ASXL1	Missense	p.W796L	10	113	8.8%	O	.	.
GC24	20	50286535	C	A	ATP9A	Splicing	c.1293+1G>T	14	132	10.6%	.	.	.
GC24	20	54824051	T	A	MC3R	Missense	p.V51D	97	146	66.4%	.	O	.
GC24	20	61833878	C	T	YTHDF1	Missense	p.G472R	7	167	4.2%	.	.	.
GC24	20	61833879	C	A	YTHDF1	Missense	p.K471N	7	165	4.2%	.	.	.
GC24	22	23523372	G	A	BCR	Nonsense	p.W75X	14	125	11.2%	.	.	.
GC24	22	50894695	T	A	SBF1	Missense	p.S1406C	7	39	17.9%	.	.	.
GC24	X	12809726	A	C	PRPS2	Missense	p.N37T	5	41	12.2%	.	.	.
GC24	X	16871874	T	C	RBBP7	Missense	p.E274G	10	76	13.2%	.	.	.
GC24	X	41555098	G	A	GPR34	Missense	p.G71E	14	142	9.9%	.	.	.
GC24	X	44035550	G	C	EFHC2	Missense	p.S677C	11	128	8.6%	.	.	.
GC24	X	44949163	G	T	KDM6A	Missense	p.G1163C	10	58	17.2%	O	.	.
GC24	X	48673989	G	T	HDAC6	Missense	p.A422S	5	47	10.6%	.	.	.
GC24	X	65392282	T	A	HEPH	Missense	p.S88T	20	184	10.9%	.	.	.
GC24	X	70145691	G	A	SLC7A3	Missense	p.P611L	43	342	12.6%	.	.	.
GC24	X	106312596	C	T	RBM41	Missense	p.E322K	50	112	44.6%	.	.	.
GC24	X	108641794	C	T	GUCY2F	Missense	p.M753I	47	113	41.6%	.	.	.
GC24	X	114082583	C	A	HTR2C	Missense	p.P123T	5	78	6.4%	.	.	.
GC24	X	129279527	G	T	AIFM1	Missense	p.P208H	15	106	14.2%	.	.	.
GC24	X	134685920	C	A	INTS6L	Missense	p.P303H	17	100	17.0%	.	.	.
GC24	X	139586549	G	T	SOX3	Missense	p.P226H	7	117	6.0%	.	.	.
GC24	X	153236132	C	T	HCFC1	Missense	p.G54R	21	97	21.6%	.	.	.
GC25	1	1900107	-	CTC	CFAP74	Nonframeshift	p.K404delinsKR	68	180	37.8%	.	.	.
GC25	1	92642765	A	C	KIAA1107	Missense	p.Q159P	36	96	37.5%	.	.	.
GC25	1	203054687	G	C	MYOG	Missense	p.L135V	23	51	45.1%	.	.	.
GC25	1	248525329	-	TA	OR2T4	Frameshift	p.T149fs	27	72	37.5%	.	.	.
GC25	3	10146080	-	A	FANCD2OS	Frameshift	p.C127fs	84	223	37.7%	.	.	.
GC25	3	38134321	C	G	DLEC1	Missense	p.T569S	57	152	37.5%	.	.	.

GC25	3	169566040	A	C	LRRC31	Missense	p.F343V	41	119	34.5%	.	.	.
GC25	4	15004879	-	GCCGCC	CPEB2	Nonframeshift	p.K194delinsKAA	34	113	30.1%	.	O	.
GC25	4	68472023	C	T	STAP1	Missense	p.P279L	19	64	29.7%	.	.	.
GC25	4	92519785	T	G	CCSER1	Missense	p.H760Q	40	115	34.8%	.	.	.
GC25	4	123161162	C	G	KIAA1109	Missense	p.T1442S	40	102	39.2%	.	.	.
GC25	4	185583039	-	G	PRIMPOL	Frameshift	p.M1fs	28	67	41.8%	.	.	.
GC25	5	3600321	C	T	IRX1	Missense	p.P420L	32	87	36.8%	.	.	.
GC25	5	68599757	C	T	CCDC125	Splicing	c.615-1G>A;NM_001297697	15	45	33.3%	.	.	.
GC25	5	101599470	T	C	SLCO4C1	Missense	p.M273V	47	108	43.5%	.	.	.
GC25	5	112420955	C	G	MCC	Missense	p.S294T	32	72	44.4%	.	.	.
GC25	5	122426202	C	T	PRDM6	Missense	p.R165C	53	141	37.6%	.	.	.
GC25	6	26199299	T	A	HIST1H2AD	Missense	p.Y58F	8	33	24.2%	.	.	.
GC25	6	41001747	G	T	UNC5CL	Missense	p.Q187K	7	91	7.7%	.	.	.
GC25	7	107602123	T	C	LAMB1	Splicing	c.1858-2A>G	37	112	33.0%	.	.	.
GC25	7	107717470	A	C	LAMB4	Missense	p.D681E	32	81	39.5%	.	.	.
GC25	8	75227468	G	A	JPH1	Missense	p.T256M	15	370	4.1%	.	.	.
GC25	9	5746015	G	A	RIC1	Missense	p.V394I	59	122	48.4%	.	.	.
GC25	9	139943437	C	G	ENTPD2	Missense	p.G391R	12	70	17.1%	.	.	.
GC25	11	12901298	T	C	TEAD1	Missense	p.M125T	41	125	32.8%	.	.	.
GC25	11	92531736	G	T	FAT3	Missense	p.V1853L	98	353	27.8%	.	.	.
GC25	12	16425553	G	T	SLC15A5	Missense	p.L176M	20	121	16.5%	.	.	.
GC25	14	20666176	-	A	OR11G2	Frameshift	p.K228fs	116	346	33.5%	.	.	.
GC25	15	23685802	G	T	GOLGA6L2	Missense	p.A607E	6	47	12.8%	.	.	.
GC25	16	20376758	G	T	PDILT	Missense	p.D407E	55	143	38.5%	.	.	.
GC25	16	21123340	T	C	DNAH3	Missense	p.N572D	6	149	4.0%	.	.	.
GC25	16	51173771	G	A	SALL1	Missense	p.H691Y	52	150	34.7%	.	.	.
GC25	16	84075615	A	T	SLC38A8	Missense	p.S50T	8	30	26.7%	.	.	.
GC25	17	39969287	A	G	FKBP10	Missense	p.M1V	23	84	27.4%	.	.	.
GC25	18	32917293	C	T	ZNF24	Missense	p.C337Y	7	120	5.8%	.	.	.
GC25	19	44833199	T	-	ZNF112	Frameshift	p.I310fs	107	281	38.1%	.	.	.

GC25	19	48956215	A	G	GRWD1	Missense	p.Q425R	62	150	41.3%	.	.	.
GC25	19	56012551	G	C	SSC5D	Missense	p.R923T	15	26	57.7%	.	O	.
GC25	20	62197308	G	T	HELZ2	Missense	p.A387E	16	114	14.0%	.	.	.
GC26	1	26156202	C	G	MTFR1L	Missense	p.H218Q	55	174	31.6%	.	.	.
GC26	1	115231266	T	C	AMPD1	Missense	p.D73G	45	198	22.7%	.	.	.
GC26	1	153233489	-	GGCGGT	LOR	Nonframeshift	p.G22delinsGRC	68	155	43.9%	.	.	.
GC26	1	156020296	G	A	UBQLN4	Missense	p.A156V	27	138	19.6%	.	O	.
GC26	1	232940824	G	A	MAP10	Missense	p.E19K	22	75	29.3%	.	.	.
GC26	2	25057721	C	T	ADCY3	Missense	p.E583K	55	98	56.1%	.	.	.
GC26	2	136718999	T	C	DARS	Missense	p.H96R	16	92	17.4%	.	.	.
GC26	2	161030533	G	C	ITGB6	Missense	p.D142E	21	123	17.1%	.	.	.
GC26	2	179586600	A	T	TTN	Missense	p.M6353K	78	130	60.0%	.	.	.
GC26	2	179588409	C	T	TTN	Missense	p.V5896M	67	120	55.8%	.	.	.
GC26	2	186661344	G	A	FSIP2	Missense	p.A3161T	64	107	59.8%	.	.	.
GC26	2	186661345	C	G	FSIP2	Missense	p.A3161G	65	108	60.2%	.	.	.
GC26	2	219878264	C	G	CFAP65	Missense	p.V1275L	44	81	54.3%	.	.	.
GC26	3	7620203	A	G	GRM7	Missense	p.K537R	55	207	26.6%	.	.	.
GC26	3	121351316	-	GGCTCAGGCTCA	HCLS1	Nonframeshift	p.P331delinsLSLSP	116	202	57.4%	.	.	.
GC26	5	13701426	-	A	DNAH5	Frameshift	p.F4486fs	24	46	52.2%	.	O	.
GC26	5	75427742	A	G	SV2C	Missense	p.D56G	39	141	27.7%	.	.	.
GC26	5	131924565	A	C	RAD50	Missense	p.Q413P	13	83	15.7%	.	.	O
GC26	5	140048565	C	T	WDR55	Missense	p.T217I	31	157	19.7%	.	.	.
GC26	6	34512263	T	C	SPDEF	Splicing	.	9	55	16.4%	.	.	.
GC26	6	105733421	C	T	PREP	Missense	p.G497S	16	119	13.4%	.	.	.
GC26	6	109763746	C	G	SMPD2	Missense	p.L137V	19	135	14.1%	.	.	.
GC26	6	167549870	T	C	CCR6	Missense	p.I51T	46	163	28.2%	.	.	.
GC26	7	12391269	-	A	VWDE	Frameshift	p.N1157fs	52	104	50.0%	.	.	.
GC26	7	45121259	C	T	NACAD	Missense	p.G1400S	13	82	15.9%	.	.	.
GC26	7	127224279	G	C	GCC1	Missense	p.L320V	52	228	22.8%	.	.	.
GC26	8	12592906	C	A	LONRF1	Missense	p.L474F	8	56	14.3%	.	.	.
GC26	8	52384754	G	A	PXDNL	Missense	p.H269Y	28	109	25.7%	.	.	.

GC26	8	142161937	-	GTTA	DENND3	Splicing	c.834+1->GTTA	39	86	45.3%	.	.	.
GC26	8	145161333	G	A	MAF1	Missense	p.E156K	22	136	16.2%	.	.	.
GC26	9	19372061	G	A	DENND4C	Missense	p.A1874T	54	80	67.5%	.	.	.
GC26	9	95078405	G	A	NOL8	Missense	p.P168S	30	176	17.0%	.	.	.
GC26	10	8100466	G	A	GATA3	Missense	p.S147N	35	135	25.9%	O	.	.
GC26	10	21823676	G	A	MLLT10	Missense	p.A35T	58	359	16.2%	.	.	.
GC26	11	6592463	G	A	DNHD1	Missense	p.S4574N	421	676	62.3%	.	.	.
GC26	11	11959806	G	T	USP47	Missense	p.V626L	20	110	18.2%	.	.	.
GC26	12	56537089	T	C	ESYT1	Missense	p.M1076T	26	175	14.9%	.	.	.
GC26	12	93163939	T	C	PLEKHG7	Missense	p.F370L	27	132	20.5%	.	.	.
GC26	12	113618756	-	CTT	DDX54	Nonframeshift	p.K94delinsKR	91	196	46.4%	.	.	.
GC26	13	29284976	C	G	SLC46A3	Missense	p.R355S	27	134	20.1%	.	.	.
GC26	14	50807836	C	T	CDKL1	Missense	p.C191Y	194	285	68.1%	.	.	.
GC26	14	53521209	G	A	DDHD1	Missense	p.T795I	74	109	67.9%	.	.	.
GC26	15	40866377	C	T	RPUSD2	Missense	p.H458Y	23	124	18.5%	.	.	.
GC26	15	42005575	C	T	MGA	Missense	p.A1104V	28	104	26.9%	.	.	.
GC26	15	45409859	G	C	DUOXA1	Missense	p.P391A	8	188	4.3%	.	.	.
GC26	15	50154518	G	A	ATP8B4	Missense	p.T1074I	24	96	25.0%	.	.	.
GC26	15	60919508	C	T	RORA	Missense	p.M22I	46	196	23.5%	.	.	.
GC26	16	424292	T	G	TMEM8A	Missense	p.I562L	21	92	22.8%	.	.	.
GC26	16	450141	-	AG	NME4	Frameshift	p.R158fs	49	75	65.3%	.	.	.
GC26	16	2024636	T	A	TBL3	Missense	p.V112E	7	95	7.4%	.	.	.
GC26	16	58073786	A	C	MMP15	Missense	p.N150H	30	184	16.3%	.	.	.
GC26	16	66413328	C	T	CDH5	Missense	p.P30S	36	110	32.7%	.	.	.
GC26	16	68026516	A	G	DPEP2	Missense	p.L96P	20	80	25.0%	.	.	.
GC26	16	72832239	G	A	ZFHX3	Missense	p.L534F	34	151	22.5%	O	.	.
GC26	17	26966624	A	G	KIAA0100	Missense	p.V351A	51	257	19.8%	.	.	.
GC26	17	48157641	C	T	ITGA3	Missense	p.R908C	28	130	21.5%	.	.	.
GC26	17	73626919	-	TG	RECQL5	Splicing	c.1586-2->CA	10	31	32.3%	.	O	.
GC26	17	74288577	C	G	QRICH2	Missense	p.R578P	99	465	21.3%	.	.	.
GC26	18	59195207	G	A	CDH20	Missense	p.S342N	17	83	20.5%	.	.	.

GC26	19	10218274	G	A	PPAN	Missense	p.V42I	22	151	14.6%	.	.	.
GC26	19	10244922	A	G	DNMT1	Missense	p.M1599T	23	86	26.7%	.	.	.
GC26	19	12776190	C	A	MAN2B1	Missense	p.V138F	27	78	34.6%	.	.	.
GC26	19	37005149	G	C	ZNF260	Missense	p.P331R	22	145	15.2%	.	.	.
GC26	19	41622108	-	C	CYP2F1	Frameshift	p.S5fs	34	113	30.1%	.	.	.
GC26	19	44376906	C	A	ZNF404	Missense	p.R484I	34	171	19.9%	.	O	.
GC26	19	44681726	-	A	ZNF226	Frameshift	p.T771fs	38	126	30.2%	.	.	.
GC26	19	45662311	C	T	NKPD1	Missense	p.G47R	7	32	21.9%	.	.	.
GC26	19	48059030	G	T	ZNF541	Missense	p.N28K	7	47	14.9%	.	.	.
GC26	19	49442850	-	G	DHDH	Frameshift	p.G171fs	86	133	64.7%	.	.	.
GC26	19	55453077	C	T	NLRP7	Missense	p.M1I	17	92	18.5%	.	.	.
GC26	19	56549455	C	G	NLRP5	Missense	p.P894A	50	185	27.0%	.	.	.
GC26	20	21492799	G	A	NKX2-2	Missense	p.T195M	51	394	12.9%	.	O	.
GC26	20	25655745	A	G	ZNF337	Missense	p.Y727H	53	182	29.1%	.	.	.
GC26	20	62632565	C	G	PRPF6	Missense	p.R387G	24	112	21.4%	.	.	.
GC26	21	43298955	-	G	PRDM15	Frameshift	p.R88fs	49	52	94.2%	.	.	.
GC26	22	29885568	-	AAGTCCCCTGAGAAGGCC	NEFH	Nonsense	p.K647delinsKVPX	31	58	53.4%	.	.	.
GC26	22	50750620	G	A	DENND6B	Missense	p.T569M	23	123	18.7%	.	.	.
GC26	X	50052128	C	T	CCNB3	Missense	p.T320I	74	172	43.0%	.	.	.
GC26	X	55479451	G	T	MAGEH1	Missense	p.G215V	47	107	43.9%	.	.	.
GC26	X	70149808	C	T	SLC7A3	Missense	p.V14I	27	100	27.0%	.	.	.
GC26	X	84362724	C	T	SATL1	Nonsense	p.W417X	56	194	28.9%	.	O	.
GC27	1	14108837	G	T	PRDM2	Missense	p.R1315L	8	100	8.0%	.	.	.
GC27	1	16069122	T	G	TMEM82	Missense	p.L23R	11	50	22.0%	.	.	.
GC27	1	19553945	C	T	EMC1	Splicing	c.1999-1G>A;NM_001271427	7	83	8.4%	.	.	.
GC27	1	44461695	G	A	CCDC24	Missense	p.A129T	48	124	38.7%	.	.	.
GC27	1	47130998	C	A	ATPAF1	Missense	p.R125L	6	106	5.7%	.	.	.
GC27	1	152281144	T	G	FLG	Missense	p.K2073T	5	70	7.1%	.	.	.
GC27	1	156844722	G	A	NTRK1	Missense	p.V420I	8	170	4.7%	.	.	.
GC27	1	169838111	C	T	SCYL3	Missense	p.C232Y	8	161	5.0%	.	.	.

GC27	1	179989575	T	C	CEP350	Missense	p.F889S	96	279	34.4%	.	.	.
GC27	1	201751946	C	A	NAV1	Missense	p.A378E	7	71	9.9%	.	.	.
GC27	1	228362434	G	C	IBA57	Missense	p.C128S	5	65	7.7%	.	.	.
GC27	2	37455263	A	G	CEBPZ	Missense	p.L358S	12	129	9.3%	.	.	.
GC27	2	105472266	G	A	POU3F3	Missense	p.A100T	31	121	25.6%	.	.	.
GC27	2	163174562	T	G	IFIH1	Missense	p.T86P	11	173	6.4%	.	.	.
GC27	2	173916485	G	C	RAPGEF4	Missense	p.R789P	12	187	6.4%	.	.	.
GC27	2	179472649	T	G	TTN	Missense	p.K8557T	26	342	7.6%	.	.	.
GC27	3	38127819	G	T	DLEC1	Missense	p.G508V	8	64	12.5%	.	.	.
GC27	3	73111482	-	A	EBLN2	Frameshift	p.R84fs	92	257	35.8%	.	O	.
GC27	4	37448106	C	T	NWD2	Missense	p.S1499L	17	189	9.0%	.	O	.
GC27	4	106156172	G	A	TET2	Missense	p.S358N	7	150	4.7%	O	.	.
GC27	4	147561952	G	A	POU4F2	Missense	p.G408S	21	182	11.5%	.	.	.
GC27	5	33944842	C	A	SLC45A2	Missense	p.G502W	7	150	4.7%	.	.	.
GC27	5	154181853	G	A	LARP1	Missense	p.R591H	29	231	12.6%	.	.	.
GC27	6	10687339	C	T	C6orf52	Missense	p.G44S	23	70	32.9%	.	.	.
GC27	6	43184105	T	A	CUL9	Missense	p.L2049Q	39	152	25.7%	.	.	.
GC27	6	132874845	T	G	TAAR8	Missense	p.S338R	4	48	8.3%	.	.	.
GC27	7	105904029	C	T	NAMPT	Missense	p.D260N	38	152	25.0%	.	.	.
GC27	8	3257045	C	T	CSMD1	Missense	p.C758Y	7	78	9.0%	.	.	.
GC27	8	41832331	T	C	KAT6A	Missense	p.D458G	38	118	32.2%	.	.	.
GC27	9	114484859	T	C	C9orf84	Missense	p.Q551R	9	49	18.4%	.	.	.
GC27	10	19981449	T	-	MALRD1	Frameshift	p.L2094fs	53	133	39.8%	.	.	.
GC27	10	21074707	A	C	NEBL	Missense	p.L261R	9	185	4.9%	.	.	.
GC27	10	56138558	C	A	PCDH15	Missense	p.R79I	7	79	8.9%	.	.	.
GC27	10	62648433	T	G	RHOBTB1	Missense	p.E211D	11	165	6.7%	.	.	.
GC27	11	17428283	C	A	ABCC8	Missense	p.S1072I	12	166	7.2%	.	.	.
GC27	11	47372976	C	T	MYBPC3	Missense	p.A36T	17	51	33.3%	.	.	.
GC27	11	47510458	C	G	CELF1	Missense	p.E37Q	9	136	6.6%	.	.	.
GC27	11	125465823	G	A	STT3A	Missense	p.G5R	26	134	19.4%	.	.	.
GC27	12	7528464	C	A	CD163L1	Missense	p.D850Y	17	174	9.8%	.	.	.

GC27	12	10959131	A	C	TAS2R8	Missense	p.I150R	10	168	6.0%	.	.	.
GC27	12	19511243	A	G	PLEKHA5	Missense	p.R890G	17	83	20.5%	.	.	.
GC27	12	54803360	G	A	ITGA5	Missense	p.S124L	18	36	50.0%	.	.	.
GC27	12	120574407	C	G	GCN1	Missense	p.V2303L	10	84	11.9%	.	.	.
GC27	13	33628349	C	T	KL	Missense	p.T422I	41	182	22.5%	.	.	.
GC27	15	40588795	C	A	PLCB2	Missense	p.E505D	28	114	24.6%	.	.	.
GC27	15	48780687	A	G	FBN1	Missense	p.I1029T	23	86	26.7%	.	.	.
GC27	16	732473	G	A	STUB1	Nonsense	p.W227X	8	149	5.4%	.	.	.
GC27	16	47630365	G	T	PHKB	Missense	p.R429L	46	176	26.1%	.	.	.
GC27	16	88600221	C	A	ZFPM1	Missense	p.P619T	52	96	54.2%	.	.	.
GC27	17	4794812	C	T	MINK1	Missense	p.S581F	19	70	27.1%	.	.	.
GC27	18	44184123	C	T	LOXHD1	Missense	p.E277K	14	57	24.6%	.	.	.
GC27	18	60191240	C	A	ZCCHC2	Missense	p.L195I	16	48	33.3%	.	.	.
GC27	19	9088787	C	G	MUC16	Missense	p.E1010Q	17	225	7.6%	.	.	.
GC27	19	51413987	G	A	KLK4	Missense	p.T3I	39	130	30.0%	.	.	.
GC27	19	53304181	T	C	ZNF28	Missense	p.K306R	29	56	51.8%	.	.	.
GC27	19	58850435	C	G	ZSCAN22	Missense	p.P407A	71	204	34.8%	.	.	.
GC27	20	31480051	C	T	EFCAB8	Missense	p.R236W	65	163	39.9%	.	.	.
GC27	20	62562361	A	G	DNAJC5	Missense	p.Q160R	30	85	35.3%	.	.	.
GC27	21	41385067	T	G	DSCAM	Missense	p.E1960A	21	77	27.3%	.	.	.
GC27	X	18631376	C	T	CDKL5	Nonsense	p.Q753X	9	146	6.2%	.	.	.
GC27	X	135954479	C	G	RBMX	Missense	p.R189P	9	114	7.9%	.	.	.
GC27	X	149681321	C	T	MAMLD1	Missense	p.T992I	15	51	29.4%	.	.	.
GC28	1	27190191	C	T	SFN	Missense	p.P163L	53	204	26.0%	.	O	.
GC28	1	158597425	A	C	SPTA1	Missense	p.I1885S	18	138	13.0%	.	.	.
GC28	1	205888115	T	C	SLC26A9	Splicing	c.2111-2A>G	24	110	21.8%	.	.	.
GC28	1	225273391	T	G	DNAH14	Missense	p.I1158S	81	172	47.1%	.	.	.
GC28	1	231064741	T	G	TTC13	Missense	p.N414H	12	65	18.5%	.	.	.
GC28	2	32897370	A	G	TTC27	Missense	p.N274S	80	150	53.3%	.	.	.
GC28	2	37410588	T	G	SULT6B1	Missense	p.I90L	22	101	21.8%	.	.	.
GC28	2	73678624	C	T	ALMS1	Missense	p.T1656I	57	231	24.7%	.	.	.

GC28	2	103149006	T	G	SLC9A4	Missense	p.H752Q	54	101	53.5%	.	.	.
GC28	2	170038038	C	G	LRP2	Missense	p.K3363N	20	119	16.8%	.	.	.
GC28	2	219209243	G	A	PNKD	Missense	p.E288K	11	50	22.0%	.	.	.
GC28	2	242741293	C	T	GAL3ST2	Missense	p.R73C	28	103	27.2%	.	.	.
GC28	3	9880768	A	T	RPUSD3	Nonsense	p.C280X	15	76	19.7%	.	.	.
GC28	3	33060047	C	T	GLB1	Missense	p.G283R	24	91	26.4%	.	.	.
GC28	3	38040435	-	CAC	VILL	Nonframeshift	p.Y325delinsYH	39	116	33.6%	.	.	.
GC28	3	73111482	-	A	EBLN2	Frameshift	p.R84fs	103	251	41.0%	.	O	.
GC28	3	121489268	T	A	IQCB1	Missense	p.D441V	97	224	43.3%	.	.	.
GC28	3	122437263	A	G	PARP14	Missense	p.K1422R	70	268	26.1%	.	.	.
GC28	3	132036319	C	T	ACPP	Missense	p.L7F	5	49	10.2%	.	.	.
GC28	3	133526592	G	C	SRPRB	Missense	p.L84F	30	140	21.4%	.	.	.
GC28	3	185775226	G	C	ETV5	Missense	p.P342R	75	166	45.2%	.	.	.
GC28	4	39409252	T	C	KLB	Missense	p.M228T	67	150	44.7%	.	.	.
GC28	4	144621405	-	GCAGCA	FREM3	Nonframeshift	p.Q142delinsCCQ	45	136	33.1%	.	.	.
GC28	4	146770521	T	A	ZNF827	Missense	p.Q725L	21	175	12.0%	.	.	.
GC28	5	5464131	G	A	ICE1	Missense	p.D1562N	29	137	21.2%	.	.	.
GC28	5	94845314	C	T	TTC37	Missense	p.A1000T	44	108	40.7%	.	.	.
GC28	6	83848049	G	A	DOPEY1	Missense	p.V1421I	57	181	31.5%	.	.	.
GC28	6	97553321	G	C	KLHL32	Missense	p.K210N	17	100	17.0%	.	.	.
GC28	6	131971286	T	C	ENPP3	Missense	p.S92P	37	82	45.1%	.	.	.
GC28	7	50143996	G	T	C7orf72	Missense	p.A214S	46	88	52.3%	.	.	.
GC28	7	151874362	C	T	KMT2C	Missense	p.D2726N	120	250	48.0%	O	.	.
GC28	8	26505209	T	G	DPYSL2	Missense	p.F497V	43	88	48.9%	.	.	.
GC28	8	28635354	C	G	INTS9	Missense	p.E442Q	28	103	27.2%	.	.	.
GC28	8	35425625	T	G	UNC5D	Missense	p.V106G	29	163	17.8%	.	.	.
GC28	8	103250840	-	G	RRM2B	Frameshift	p.R71fs	30	90	33.3%	.	.	.
GC28	9	13217254	A	G	MPDZ	Missense	p.F376L	21	83	25.3%	.	.	.
GC28	9	35809997	A	G	SPAG8	Missense	p.S466P	52	123	42.3%	.	.	.
GC28	9	97216345	C	G	MFSD14B	Missense	p.Q341E	48	206	23.3%	.	.	.
GC28	9	114466202	C	T	C9orf84	Missense	p.V873I	28	121	23.1%	.	.	.

GC28	10	64973719	A	T	JMJD1C	Missense	p.H517Q	56	203	27.6%	.	.	.
GC28	11	1092940	A	G	MUC2	Missense	p.T1587A	10	229	4.4%	.	.	.
GC28	11	44616232	A	G	CD82	Missense	p.K7R	50	150	33.3%	.	.	.
GC28	11	47521006	C	A	CELF1	Missense	p.V24F	36	134	26.9%	.	.	.
GC28	11	55872830	T	G	OR8H2	Missense	p.F104L	59	156	37.8%	.	.	.
GC28	11	76954789	-	A	GDPD4	Frameshift	p.N397fs	48	116	41.4%	.	.	.
GC28	11	77333674	C	T	CLNS1A	Missense	p.G103R	22	76	28.9%	.	.	.
GC28	12	48596876	-	A	OR10AD1	Frameshift	p.L67fs	189	347	54.5%	.	.	.
GC28	13	25912821	A	C	NUP58	Missense	p.T516P	49	104	47.1%	.	.	.
GC28	13	26043219	A	C	ATP8A2	Missense	p.N21H	33	74	44.6%	.	.	.
GC28	13	41705168	T	A	KBTBD6	Missense	p.M494L	34	108	31.5%	.	.	.
GC28	15	56723621	G	C	MNS1	Missense	p.L449V	45	92	48.9%	.	.	.
GC28	16	450141	-	AG	NME4	Frameshift	p.R158fs	42	90	46.7%	.	.	.
GC28	16	15967362	-	CTA	FOPNL	Nonframeshift	p.R79delinsIG	64	141	45.4%	.	.	.
GC28	16	23503027	C	G	GGA2	Missense	p.R149P	11	136	8.1%	.	.	.
GC28	16	25175981	A	G	LCMT1	Missense	p.N156S	35	114	30.7%	.	.	.
GC28	16	71482783	T	C	ZNF23	Missense	p.H324R	29	114	25.4%	.	.	.
GC28	16	87637894	-	CTG	JPH3	Nonframeshift	p.P144delinsP C	10	30	33.3%	.	.	.
GC28	17	1611407	A	G	TLCD2	Missense	p.L151P	12	63	19.0%	.	.	.
GC28	17	33592116	C	A	SLFN5	Missense	p.S388R	9	199	4.5%	.	.	.
GC28	17	35932033	C	T	SYNRG	Missense	p.A241T	24	97	24.7%	.	.	.
GC28	17	56296868	G	T	MKS1	Missense	p.A2D	19	127	15.0%	.	.	.
GC28	17	56677015	T	C	TEX14	Missense	p.H570R	35	144	24.3%	.	.	.
GC28	17	74003666	G	C	EVPL	Missense	p.R1896G	123	223	55.2%	.	.	.
GC28	18	19079962	C	T	GREB1L	Missense	p.R1222W	47	95	49.5%	.	.	.
GC28	18	30847237	C	G	CCDC178	Missense	p.D401H	22	99	22.2%	.	.	.
GC28	19	7621351	G	A	PNPLA6	Missense	p.V1036M	141	269	52.4%	.	.	.
GC28	19	11545633	A	T	CCDC151	Missense	p.S69T	87	165	52.7%	.	.	.
GC28	19	36303074	G	T	PRODH2	Missense	p.Q234K	33	151	21.9%	.	.	.
GC28	19	44039501	-	A	ZNF575	Frameshift	p.L134fs	38	109	34.9%	.	.	.
GC28	19	45296847	-	C	CBLC	Frameshift	p.D372fs	25	61	41.0%	.	.	.

GC28	19	49442850	-	G	DHDH	Frameshift	p.G171fs	159	172	92.4%	.	.	.
GC28	19	52084691	-	G	ZNF175	Frameshift	p.E40fs	67	143	46.9%	.	.	.
GC28	19	58213549	T	G	ZNF154	Missense	p.Q256H	51	201	25.4%	.	.	.
GC28	20	52198281	T	A	ZNF217	Missense	p.D362V	57	224	25.4%	.	.	.
GC28	22	21133650	C	T	SERPIND1	Missense	p.A17V	42	155	27.1%	.	.	.
GC28	22	22868331	T	C	ZNF280A	Missense	p.S542G	72	136	52.9%	.	.	.
GC28	22	29885568	-	AAGTCCCCT GAGAAGGC C	NEFH	Nonsense	p.K647delinsK VPX	25	79	31.6%	.	.	.
GC28	X	38546864	G	C	TSPAN7	Missense	p.M231I	46	73	63.0%	.	.	.
GC28	X	47422678	G	C	ARAF	Missense	p.K50N	57	82	69.5%	.	.	.
GC28	X	123519706	G	T	TENM1	Missense	p.T1959N	44	116	37.9%	.	.	.
GC29	1	22173982	G	A	HSPG2	Missense	p.H2678Y	35	48	72.9%	.	.	.
GC29	1	26608812	CCAGGACA GGGACTGG GGCCGGGA	-	UBXN11	Nonframeshift	p.386_394del	10	25	40.0%	.	O	.
GC29	1	112305347	G	A	DDX20	Missense	p.D385N	117	165	70.9%	.	.	.
GC29	1	232144716	A	G	DISC1	Missense	p.K621R	117	202	57.9%	.	.	.
GC29	1	248525329	-	TA	OR2T4	Frameshift	p.T149fs	50	88	56.8%	.	.	.
GC29	2	73520603	A	G	EGR4	Missense	p.L51P	77	105	73.3%	.	.	.
GC29	2	179595702	A	G	TTN	Missense	p.V4653A	105	153	68.6%	.	.	.
GC29	2	189910579	C	T	COL5A2	Missense	p.G1086R	77	102	75.5%	.	.	.
GC29	2	202357951	A	G	C2CD6	Missense	p.L1038P	50	73	68.5%	.	.	.
GC29	2	210880950	A	G	RPE	Missense	p.E54G	6	102	5.9%	.	O	.
GC29	3	9852060	-	GAT	TTLL3	Nonframeshift	p.D124delinsG Y	84	124	67.7%	.	.	.
GC29	3	77681675	T	A	ROBO2	Missense	p.S1275T	67	103	65.0%	.	.	.
GC29	4	110370651	T	G	SEC24B	Missense	p.I71S	5	43	11.6%	.	.	.
GC29	4	110932390	-	C	EGF	Frameshift	p.P1094fs	76	129	58.9%	.	.	.
GC29	5	70308519	G	A	NAIP	Missense	p.S75L	51	308	16.6%	.	.	.
GC29	6	43400950	C	T	ABCC10	Missense	p.T368I	86	112	76.8%	.	.	.
GC29	6	83772870	A	G	UBE3D	Splicing	.	4	43	9.3%	.	.	.
GC29	6	154731509	G	A	CNKSR3	Missense	p.P446L	55	82	67.1%	.	.	.
GC29	6	155757611	C	A	NOX3	Missense	p.A247S	37	50	74.0%	.	.	.
GC29	7	80276111	C	A	CD36	Missense	p.L19M	112	162	69.1%	.	.	.

GC29	7	90894460	-	CCG	FZD1	Nonframeshift	p.P89delinsPA	97	186	52.2%	.	.	.
GC29	7	100485668	C	T	SRRT	Missense	p.P777S	128	169	75.7%	.	.	.
GC29	7	122338521	A	G	RNF133	Missense	p.V151A	155	247	62.8%	.	.	.
GC29	8	10623165	C	A	PINX1	Missense	p.A245S	104	152	68.4%	.	.	.
GC29	8	133107972	C	G	HHLA1	Missense	p.K114N	59	89	66.3%	.	.	.
GC29	9	100092969	-	GAGGAG	CCDC180	Nonframeshift	p.E776delinsGGE	23	56	41.1%	.	.	.
GC29	10	4877892	G	C	AKR1E2	Missense	p.S117T	58	91	63.7%	.	.	.
GC29	10	13224982	A	T	MCM10	Missense	p.Q327L	92	137	67.2%	.	.	.
GC29	10	21805467	-	CCTCCT	SKIDA1	Nonframeshift	p.G429delinsRRG	62	135	45.9%	.	.	.
GC29	11	57466384	C	G	ZDHHC5	Missense	p.D492E	177	257	68.9%	.	.	.
GC29	11	66136653	C	T	SLC29A2	Missense	p.R98H	33	47	70.2%	.	.	.
GC29	12	9098996	-	AAC	M6PR	Nonframeshift	p.F2delinsCF	33	62	53.2%	.	.	.
GC29	12	31820908	-	G	ETFBKMT	Frameshift	p.W258fs	124	180	68.9%	.	.	.
GC29	12	57493637	T	C	STAT6	Missense	p.N443D	114	164	69.5%	.	.	.
GC29	12	64712547	-	GTT	C12orf56	Nonframeshift	p.N234delinsKT	133	227	58.6%	.	.	.
GC29	13	48611919	-	GGAGTC	NUDT15	Nonframeshift	p.G13delinsGSR	28	85	32.9%	.	.	.
GC29	13	52718051	-	T	NEK3	Frameshift	p.K292fs	34	46	73.9%	.	.	.
GC29	14	68270903	G	C	ZFYVE26	Missense	p.H450Q	90	138	65.2%	.	.	.
GC29	14	75537322	-	A	ZC2HC1C	Frameshift	p.M16fs	59	87	67.8%	.	.	.
GC29	15	23686546	T	C	GOLGA6L2	Missense	p.E359G	5	87	5.7%	.	O	.
GC29	15	41099900	-	GGGGC	ZFYVE19	Frameshift	p.W38fs	16	29	55.2%	.	O	.
GC29	15	99511805	-	C	PGPEP1L	Frameshift	p.V165fs	59	105	56.2%	.	.	.
GC29	16	1419970	G	C	UNKL	Missense	p.P13R	10	264	3.8%	.	.	.
GC29	16	5094586	A	G	C16orf89	Missense	p.X403Q	9	91	9.9%	.	.	.
GC29	16	30913698	-	CGGGCCCCGGGC	CTF1	Nonframeshift	p.R147delinsRRAPG	19	46	41.3%	.	.	.
GC29	16	68835797	GTA	-	CDH1	Splicing	.	58	73	79.5%	O	.	.
GC29	17	7989499	C	A	ALOX12B	Nonsense	p.E63X	35	47	74.5%	.	.	.
GC29	17	11666804	T	C	DNAH9	Missense	p.V2348A	102	166	61.4%	.	.	.
GC29	17	27958005	G	C	SSH2	Missense	p.Q1376E	26	147	17.7%	.	.	.
GC29	18	60036021	G	A	TNFRSF11A	Missense	p.E277K	152	221	68.8%	.	.	.
GC29	18	61326746	T	C	SERPINB3	Missense	p.N80D	56	83	67.5%	.	.	.

GC29	19	4513064	G	T	PLIN4	Missense	p.T289N	6	99	6.1%	.	.	.
GC29	19	51769036	A	G	SIGLECL1	Missense	p.S10G	146	203	71.9%	.	.	.
GC29	20	5953323	G	C	MCM8	Missense	p.C442S	6	82	7.3%	.	.	.
GC29	X	63412109	C	T	AMER1	Missense	p.R353Q	75	238	31.5%	O	O	.
GC29	X	86919848	C	A	KLHL4	Missense	p.D670E	22	77	28.6%	.	.	.
GC29	X	106184845	G	C	MORC4	Nonsense	p.Y880X	38	70	54.3%	.	.	.
